# Diretriz de Tomografia Computadorizada e Ressonância Magnética Cardiovascular da Sociedade Brasileira de Cardiologia e do Colégio Brasileiro de Radiologia – 2024

**DOI:** 10.36660/abc.20240608

**Published:** 2024-10-17

**Authors:** Tiago Augusto Magalhães, Adriano Camargo de Castro Carneiro, Valéria de Melo Moreira, Henrique Simão Trad, Marly Maria Uellendahl Lopes, Rodrigo Julio Cerci, Marcelo Souto Nacif, Paulo R. Schvartzman, Antônio Carlos Palandrini Chagas, Isabela Bispo Santos da Silva Costa, André Schmidt, Afonso Akio Shiozaki, Sérgio Tavares Montenegro, Leopoldo Soares Piegas, Marcelo Zapparoli, José Carlos Nicolau, Fabio Fernandes, Marcelo Souza Hadlich, Nabil Ghorayeb, Evandro Tinoco Mesquita, Luiz Flávio Galvão Gonçalves, Felix José Alvarez Ramires, Juliano de Lara Fernandes, Pedro Vellosa Schwartzmann, Salvador Rassi, Jorge Andion Torreão, José Carlos Pachón Mateos, Luiz Beck-da-Silva, Marly Conceição Silva, Gabriela Liberato, Gláucia Maria Moraes de Oliveira, Gilson Soares Feitosa, Hilka dos Santos Moraes de Carvalho, Brivaldo Markman, Ricardo Paulo de Sousa Rocha, Clerio Francisco de Azevedo, Flávio Taratsoutchi, Otavio Rizzi Coelho-Filho, Roberto Kalil, Ludhmila Abrahão Hajjar, Walther Yoshiharu Ishikawa, Cíntia Acosta Melo, Ieda Biscegli Jatene, Andrei Skromov de Albuquerque, Carolina de Medeiros Rimkus, Paulo Savoia Dias da Silva, Thiago Dieb Ristum Vieira, Fabio Biscegli Jatene, Guilherme Sant Anna Antunes de Azevedo, Raul D. Santos, Guilherme Urpia Monte, José Antonio Franchini Ramires, Marcio Sommer Bittencourt, Alvaro Avezum, Leonardo Sara da Silva, Alexandre Abizaid, Ilan Gottlieb, Dalton Bertolim Precoma, Gilberto Szarf, Antônio Carlos Sobral Sousa, Ibraim Masciarelli Francisco Pinto, Fábio de Morais Medeiros, Bruno Caramelli, José Rodrigues Parga, Tiago Senra Garcia dos Santos, Carlos Eduardo Elias dos Prazeres, Marcelo Antonio Cartaxo Queiroga Lopes, Luiz Francisco Rodrigues de Avila, Mauricio Ibrahim Scanavacca, Luis Henrique Wolff Gowdak, Silvio Henrique Barberato, Cesar Higa Nomura, Carlos Eduardo Rochitte

**Affiliations:** 1 Complexo Hospital de Clínicas da Universidade Federal do Paraná Curitiba PR Brasil Complexo Hospital de Clínicas da Universidade Federal do Paraná (CHC-UFPR), Curitiba, PR – Brasil; 2 Hospital do Coração São Paulo SP Brasil Hospital do Coração (HCOR), São Paulo, SP – Brasil; 3 Hospital Sírio Libanês São Paulo SP Brasil Hospital Sírio Libanês, SP, São Paulo, SP – Brasil; 4 Hospital Alemão Oswaldo Cruz São Paulo SP Brasil Hospital Alemão Oswaldo Cruz, São Paulo, SP – Brasil; 5 Hospital das Clínicas da Faculdade de Medicina da Universidade de São Paulo Instituto do Coração São Paulo SP Brasil Instituto do Coração (Incor) do Hospital das Clínicas da Faculdade de Medicina da Universidade de São Paulo (HCFMUSP), São Paulo SP – Brasil; 6 Lotus Radiologia Ltda Ribeirão Preto SP Brasil Lotus Radiologia Ltda, Ribeirão Preto, SP – Brasil; 7 Universidade Federal de São Paulo São Paulo SP Brasil Universidade Federal de São Paulo (UNIFESP), São Paulo, SP – Brasil; 8 Diagnósticos da América S/A São Paulo SP Brasil DASA – Diagnósticos da América S/A, São Paulo, SP – Brasil; 9 Quanta Diagnóstico por Imagem Curitiba PR Brasil Quanta Diagnóstico por Imagem, Curitiba, PR – Brasil; 10 Universidade Federal Fluminense Niterói RJ Brasil Universidade Federal Fluminense, Niterói, RJ – Brasil; 11 Hospital Universitário Antonio Pedro Niterói RJ Brasil Hospital Universitário Antonio Pedro, Niterói, RJ – Brasil; 12 Hospital Moinhos de Vento Porto Alegre RS Brasil Hospital Moinhos de Vento, Porto Alegre, RS – Brasil; 13 Faculdade de Medicina do ABC Santo André SP Brasil Faculdade de Medicina do ABC, Santo André, SP – Brasil; 14 Instituto do Câncer do Estado de São Paulo São Paulo SP Brasil Instituto do Câncer do Estado de São Paulo, São Paulo, SP – Brasil; 15 Universidade de São Paulo Ribeirão Preto SP Brasil Universidade de São Paulo (USP), Ribeirão Preto, SP – Brasil; 16 ND Núcleo Diagnóstico Maringá PR Brasil ND Núcleo Diagnóstico, Maringá, PR – Brasil; 17 Ômega Diagnóstico Maringá PR Brasil Ômega Diagnóstico, Maringá, PR – Brasil; 18 Hospital Paraná Maringá PR Brasil Hospital Paraná, Maringá, PR – Brasil; 19 Universidade de Pernambuco Recife PE Brasil PROCAPE – Universidade de Pernambuco, Recife, PE – Brasil; 20 DAPI Curitiba PR Brasil DAPI, Curitiba, PR – Brasil; 21 Fleury Medicina e Saúde Rio de Janeiro RJ Brasil Fleury Medicina e Saúde, Rio de Janeiro, RJ – Brasil; 22 Rede D'Or RJ Rio de Janeiro RJ Brasil Rede D'Or RJ, Rio de Janeiro, RJ – Brasil; 23 Unimed Rio de Janeiro RJ Brasil Unimed, Rio de Janeiro, RJ – Brasil; 24 Instituto Nacional de Cardiologia Rio de Janeiro RJ Brasil Instituto Nacional de Cardiologia (INC), Rio de Janeiro, RJ – Brasil; 25 Instituto Dante Pazzanese de Cardiologia São Paulo SP Brasil Instituto Dante Pazzanese de Cardiologia, São Paulo, SP – Brasil; 26 Inspirali Educação São Paulo SP Brasil Inspirali Educação, São Paulo, SP – Brasil; 27 Anhanguera Educacional São Paulo SP Brasil Anhanguera Educacional, São Paulo, SP – Brasil; 28 Universidade Federal Fluminense Rio de Janeiro RJ Brasil Universidade Federal Fluminense (UFF), Rio de Janeiro, RJ – Brasil; 29 Hospital São Lucas Aracaju SE Brasil Hospital São Lucas, Rede D'Or SE, Aracaju, SE – Brasil; 30 Hospital Universitário da Universidade Federal de Sergipe Aracaju SE Brasil Hospital Universitário da Universidade Federal de Sergipe, Aracaju, SE – Brasil; 31 Clínica Climedi Aracaju SE Brasil Clínica Climedi, Aracaju, SE – Brasil; 32 Radiologia Clinica de Campinas Campinas SP Brasil Radiologia Clinica de Campinas, Campinas, SP – Brasil; 33 Hospital Unimed Ribeirão Preto Ribeirão Preto SP Brasil Hospital Unimed Ribeirão Preto, Ribeirão Preto, SP – Brasil; 34 Centro Avançado de Pesquisa, Ensino e Diagnóstico Ribeirão Preto SP Brasil Centro Avançado de Pesquisa, Ensino e Diagnóstico (CAPED), Ribeirão Preto, SP – Brasil; 35 Universidade Federal de Goiás Goiânia GO Brasil Universidade Federal de Goiás, Goiânia, GO – Brasil; 36 Santa Casa da Bahia Salvador BA Brasil Santa Casa da Bahia, Salvador, BA – Brasil; 37 Universidade Federal do Rio Grande do Sul Porto Alegre RS Brasil Universidade Federal do Rio Grande do Sul (UFRGS), Porto Alegre, RS – Brasil; 38 Axial Medicina Diagnóstica Belo Horizonte MG Brasil Axial Medicina Diagnóstica, Belo Horizonte, MG – Brasil; 39 Universidade Federal do Rio de Janeiro Rio de Janeiro RJ Brasil Universidade Federal do Rio de Janeiro, Rio de Janeiro, RJ – Brasil; 40 Escola Bahiana de Medicina e Saúde Pública Salvador BA Brasil Escola Bahiana de Medicina e Saúde Pública, Salvador, BA – Brasil; 41 Universidade Federal de Pernambuco Hospital das Clínicas de Pernambuco Recife PE Brasil Hospital das Clínicas de Pernambuco da Universidade Federal de Pernambuco (UFPE), Recife, PE – Brasil; 42 Real Hospital Português de Pernambuco Recife PE Brasil Real Hospital Português de Pernambuco, Recife, PE – Brasil; 43 Hospital Monteklinikum Fortaleza CE Brasil Hospital Monteklinikum, Fortaleza, CE – Brasil; 44 Hospital Oto Kora Fortaleza CE Brasil Hospital Oto Kora, Fortaleza, CE – Brasil; 45 Abbott Northwestern Hospital Allina Health Minneapolis Heart Institute Minneapolis EUA Minneapolis Heart Institute, Abbott Northwestern Hospital Allina Health, Minneapolis – EUA; 46 Universidade Estadual de Campinas Campinas SP Brasil Universidade Estadual de Campinas (UNICAMP), Campinas, SP – Brasil; 47 Hospital Beneficência Portuguesa de São Paulo São Paulo SP Brasil Hospital Beneficência Portuguesa de São Paulo, São Paulo, SP – Brasil; 48 Hospital Infantil Sabará São Paulo SP Brasil Hospital Infantil Sabará, São Paulo, SP – Brasil; 49 Hospital das Clínicas da Faculdade de Medicina da Universidade de São Paulo São Paulo SP Brasil Hospital das Clínicas da Faculdade de Medicina da Universidade de São Paulo (HCFMUSP), São Paulo SP – Brasil; 50 Instituto D'Or de Pesquisa e Ensino São Paulo SP Brasil Instituto D'Or de Pesquisa e Ensino (IDOR), São Paulo SP – Brasil; 51 University of Iowa Hospitals and Clinics Iowa City EUA University of Iowa Hospitals and Clinics, Iowa City – EUA; 52 ECOMAX Blumenau SC Brasil ECOMAX, Blumenau, SC – Brasil; 53 Hospital Unimed Blumenau Blumenau SC Brasil Hospital Unimed Blumenau, Blumenau, SC – Brasil; 54 Hospital São José de Jaraguá do Sul Blumenau SC Brasil Hospital São José de Jaraguá do Sul, Blumenau, SC – Brasil; 55 Cliniimagem Criciúma Blumenau SC Brasil Cliniimagem Criciúma, Blumenau, SC – Brasil; 56 Instituto de Cardiologia e Transplantes do Distrito Federal Brasília DF Brasil Instituto de Cardiologia e Transplantes do Distrito Federal, Brasília, DF – Brasil; 57 University of Pittsburgh Pittsburgh EUA EUA University of Pittsburgh, Pittsburgh – EUA; 58 Clínica diagnóstica CDI Premium Goiânia GO Brasil CDI Premium – Clínica diagnóstica, Goiânia, GO – Brasil; 59 Fonte Imagem Medicina Diagnostica Rio de Janeiro RJ Brasil Fonte Imagem Medicina Diagnostica, Rio de Janeiro, RJ – Brasil; 60 Sociedade Hospitalar Angelina Caron Curitiba PR Brasil Sociedade Hospitalar Angelina Caron, Curitiba, PR – Brasil; 61 Universidade Federal de Sergipe Aracaju SE Brasil Universidade Federal de Sergipe, Aracaju, SE – Brasil; 62 Hospital São Lucas Aracaju SE Brasil Hospital São Lucas, Aracaju, SE – Brasil; 63 Rede D'Or de Aracaju Aracaju SE Brasil Rede D'Or de Aracaju, Aracaju, SE – Brasil; 64 Laboratório Sabin Centro de Imagem VEJA Brasília DF Brasil Laboratório Sabin Centro de Imagem VEJA, Brasília, DF – Brasil; 65 Hospital Alberto Urquiza Wanderley João Pessoa PB Brasil Hospital Alberto Urquiza Wanderley, João Pessoa, PB – Brasil; 66 Centro de Diagnóstico Cardiovascular Cardioeco Curitiba PR Brasil Cardioeco, Centro de Diagnóstico Cardiovascular, Curitiba, PR – Brasil

**Table:** 

Diretriz de Tomografia Computadorizada e Ressonância Magnética Cardiovascular da Sociedade Brasileira de Cardiologia e do Colégio Brasileiro de Radiologia – 2024	
O relatório abaixo lista as declarações de interesse conforme relatadas à SBC pelos especialistas durante o período de desenvolvimento deste posicionamento, 2021-2024.	
Especialista	Tipo de relacionamento com a indústria
Adriano Camargo de Castro Carneiro	Nada a ser declarado
Afonso Akio Shiozaki	Nada a ser declarado
Alexandre Abizaid	Nada a ser declarado
Alvaro Avezum	Outros relacionamentos Financiamento de atividades de educação médica continuada, incluindo viagens, hospedagens e inscrições para congressos e cursos, provenientes da indústria farmacêutica, de órteses, próteses, equipamentos e implantes, brasileiras ou estrangeiras: - EMS: Simpósios SBC Espiritualidade - DEMCA.
André Schmidt	Declaração financeira B - Financiamento de pesquisas sob sua responsabilidade direta/pessoal (direcionado ao departamento ou instituição) provenientes da indústria farmacêutica, de órteses, próteses, equipamentos e implantes, brasileiras ou estrangeiras: - Janssen: Milvexiana; AMGEN: Olpasirana.
Andrei Skromov de Albuquerque	Nada a ser declarado
Antonnio Carlos Palandri Chagas	Declaração financeira A - Pagamento de qualquer espécie e desde que economicamente apreciáveis, feitos a (i) você, (ii) ao seu cônjuge/ companheiro ou a qualquer outro membro que resida com você, (iii) a qualquer pessoa jurídica em que qualquer destes seja controlador, sócio, acionista ou participante, de forma direta ou indireta, recebimento por palestras, aulas, atuação como proctor de treinamentos, remunerações, honorários pagos por participações em conselhos consultivos, de investigadores, ou outros comitês, etc. Provenientes da indústria farmacêutica, de órteses, próteses, equipamentos e implantes, brasileiras ou estrangeiras: - Novo Nordisk: Semaglutida; Instituto De Vita: Conselho Consultivo. Outros relacionamentos Financiamento de atividades de educação médica continuada, incluindo viagens, hospedagens e inscrições para congressos e cursos, provenientes da indústria farmacêutica, de órteses, próteses, equipamentos e implantes, brasileiras ou estrangeiras: - Novo Nordisk: Semaglutida.
Antônio Carlos Sobral Sousa	Declaração financeira A - Pagamento de qualquer espécie e desde que economicamente apreciáveis, feitos a (i) você, (ii) ao seu cônjuge/ companheiro ou a qualquer outro membro que resida com você, (iii) a qualquer pessoa jurídica em que qualquer destes seja controlador, sócio, acionista ou participante, de forma direta ou indireta, recebimento por palestras, aulas, atuação como proctor de treinamentos, remunerações, honorários pagos por participações em conselhos consultivos, de investigadores, ou outros comitês, etc. Provenientes da indústria farmacêutica, de órteses, próteses, equipamentos e implantes, brasileiras ou estrangeiras: - Viatris: Inspra. Outros relacionamentos Financiamento de atividades de educação médica continuada, incluindo viagens, hospedagens e inscrições para congressos e cursos, provenientes da indústria farmacêutica, de órteses, próteses, equipamentos e implantes, brasileiras ou estrangeiras: - Pfizer: Sybrava.
Brivaldo Markman Filho	Nada a ser declarado
Bruno Caramelli	Nada a ser declarado
Carlos Eduardo Elias dos Prazeres	Nada a ser declarado
Carlos Eduardo Rochitte	Nada a ser declarado
Carolina de Medeiros Rimkus	Declaração financeira A - Pagamento de qualquer espécie e desde que economicamente apreciáveis, feitos a (i) você, (ii) ao seu cônjuge/ companheiro ou a qualquer outro membro que resida com você, (iii) a qualquer pessoa jurídica em que qualquer destes seja controlador, sócio, acionista ou participante, de forma direta ou indireta, recebimento por palestras, aulas, atuação como proctor de treinamentos, remunerações, honorários pagos por participações em conselhos consultivos, de investigadores, ou outros comitês, etc. Provenientes da indústria farmacêutica, de órteses, próteses, equipamentos e implantes, brasileiras ou estrangeiras: - Guerbet: inteligência artificial; Biogen: imunomoduladores; Roche: imunomoduladores.
Cesar Higa Nomura	Nada a ser declarado
Cíntia Acosta Melo	Nada a ser declarado
Clerio Francisco de Azevedo Filho	Nada a ser declarado
Dalton Bertolim Precoma	Declaração financeira A - Pagamento de qualquer espécie e desde que economicamente apreciáveis, feitos a (i) você, (ii) ao seu cônjuge/ companheiro ou a qualquer outro membro que resida com você, (iii) a qualquer pessoa jurídica em que qualquer destes seja controlador, sócio, acionista ou participante, de forma direta ou indireta, recebimento por palestras, aulas, atuação como proctor de treinamentos, remunerações, honorários pagos por participações em conselhos consultivos, de investigadores, ou outros comitês, etc. Provenientes da indústria farmacêutica, de órteses, próteses, equipamentos e implantes, brasileiras ou estrangeiras: - Novo Nordisk: Ozempic; Astrazeneca: Forxiga; Novartis: Entresto; Daiichi Sankyo: Lixiana; Lilly: Tirzetapibe. B - Financiamento de pesquisas sob sua responsabilidade direta/pessoal (direcionado ao departamento ou instituição) provenientes da indústria farmacêutica, de órteses, próteses, equipamentos e implantes, brasileiras ou estrangeiras: - Novo Nordisk: insuficiência cardíaca, obesidade; Lilly: insuficiência cardíaca, obesidade; Astrazeneca: insuficiência renal, hipertensão, insuficiência cardíaca; MSD: dislipidemia; Bayer: insuficiência cardíaca; Janssen-BMS: anticoagulação e prevenção de cardioembolismo. Outros relacionamentos Financiamento de atividades de educação médica continuada, incluindo viagens, hospedagens e inscrições para congressos e cursos, provenientes da indústria farmacêutica, de órteses, próteses, equipamentos e implantes, brasileiras ou estrangeiras: - AstraZeneca: insuficiência cardíaca; Lilly: insuficiência cardíaca e obesidade. Participação societária de qualquer natureza e qualquer valor economicamente apreciável de empresas na área de saúde, de ensino ou em empresas concorrentes ou fornecedoras da SBC: - Área da Saúde.
Evandro Tinoco Mesquita	Declaração financeira A - Pagamento de qualquer espécie e desde que economicamente apreciáveis, feitos a (i) você, (ii) ao seu cônjuge/ companheiro ou a qualquer outro membro que resida com você, (iii) a qualquer pessoa jurídica em que qualquer destes seja controlador, sócio, acionista ou participante, de forma direta ou indireta, recebimento por palestras, aulas, atuação como proctor de treinamentos, remunerações, honorários pagos por participações em conselhos consultivos, de investigadores, ou outros comitês, etc. Provenientes da indústria farmacêutica, de órteses, próteses, equipamentos e implantes, brasileiras ou estrangeiras: - Ache: material educacional e aulas Astra. Outros relacionamentos Financiamento de atividades de educação médica continuada, incluindo viagens, hospedagens e inscrições para congressos e cursos, provenientes da indústria farmacêutica, de órteses, próteses, equipamentos e implantes, brasileiras ou estrangeiras: - Pfizer: amiloidose.
Fabio Biscegli Jatene	Nada a ser declarado
Fábio de Morais Medeiros	Nada a ser declarado
Fabio Fernandes	Nada a ser declarado
Felix José Alvarez Ramires	Declaração financeira A - Pagamento de qualquer espécie e desde que economicamente apreciáveis, feitos a (i) você, (ii) ao seu cônjuge/companheiro ou a qualquer outro membro que resida com você, (iii) a qualquer pessoa jurídica em que qualquer destes seja controlador, sócio, acionista ou participante, de forma direta ou indireta, recebimento por palestras, aulas, atuação como proctor de treinamentos, remunerações, honorários pagos por participações em conselhos consultivos, de investigadores, ou outros comitês, etc. Provenientes da indústria farmacêutica, de órteses, próteses, equipamentos e implantes, brasileiras ou estrangeiras: - Novartis; Pfizer; AstraZeneca; Amgen.
Flávio Tarasoutchi	Outros relacionamentos Financiamento de atividades de educação médica continuada, incluindo viagens, hospedagens e inscrições para congressos e cursos, provenientes da indústria farmacêutica, de órteses, próteses, equipamentos e implantes, brasileiras ou estrangeiras: - Aula da Edwards.
Gabriela Liberato	Nada a ser declarado
Gilberto Szarf	Nada a ser declarado
Gilson Soares Feitosa Filho	Nada a ser declarado
Gláucia Maria Moraes de Oliveira	Nada a ser declarado
Guilherme Sant Anna Antunes de Azevedo	Declaração financeira A - Pagamento de qualquer espécie e desde que economicamente apreciáveis, feitos a (i) você, (ii) ao seu cônjuge/ companheiro ou a qualquer outro membro que resida com você, (iii) a qualquer pessoa jurídica em que qualquer destes seja controlador, sócio, acionista ou participante, de forma direta ou indireta, recebimento por palestras, aulas, atuação como proctor de treinamentos, remunerações, honorários pagos por participações em conselhos consultivos, de investigadores, ou outros comitês, etc. Provenientes da indústria farmacêutica, de órteses, próteses, equipamentos e implantes, brasileiras ou estrangeiras: - Canon: tomografia computadorizada.
Guilherme Urpia Monte	Nada a ser declarado
Henrique Simão Trad	Nada a ser declarado
Hilka dos Santos Moraes de Carvalho	Nada a ser declarado
Ibraim Masciarelli Francisco Pinto	Nada a ser declarado
Ieda Biscegli Jatene	Nada a ser declarado
Ilan Gottlieb	Nada a ser declarado
Isabela Bispo Santos da Silva Costa	Nada a ser declarado
Jorge Andion Torreão	Nada a ser declarado
José Antonio Franchini Ramires	Nada a ser declarado
José Carlos Nicolau	Declaração financeira A - Pagamento de qualquer espécie e desde que economicamente apreciáveis, feitos a (i) você, (ii) ao seu cônjuge/companheiro ou a qualquer outro membro que resida com você, (iii) a qualquer pessoa jurídica em que qualquer destes seja controlador, sócio, acionista ou participante, de forma direta ou indireta, recebimento por palestras, aulas, atuação como proctor de treinamentos, remunerações, honorários pagos por participações em conselhos consultivos, de investigadores, ou outros comitês, etc. Provenientes da indústria farmacêutica, de órteses, próteses, equipamentos e implantes, brasileiras ou estrangeiras: - Amgen; AstraZeneca; Bayer; CSL Behring; Daiichi Sankyo; Dalcor; Esperion; Janssen; Novartis; Novo Nordisk; Sanofi; Vifor; Anthos; Libbs. Outros relacionamentos Financiamento de atividades de educação médica continuada, incluindo viagens, hospedagens e inscrições para congressos e cursos, provenientes da indústria farmacêutica, de órteses, próteses, equipamentos e implantes, brasileiras ou estrangeiras: - Novo Nordisk.
José Carlos Pachón Mateos	Nada a ser declarado
José Rodrigues Parga Filho	Nada a ser declarado
Juliano de Lara Fernandes	Nada a ser declarado
Leonardo Sara da Silva	Nada a ser declarado
Leopoldo Soares Piegas	Nada a ser declarado
Ludhmila Abrahão Hajjar	Nada a ser declarado
Luis Beck-da-Silva	Declaração financeira A - Pagamento de qualquer espécie e desde que economicamente apreciáveis, feitos a (i) você, (ii) ao seu cônjuge/companheiro ou a qualquer outro membro que resida com você, (iii) a qualquer pessoa jurídica em que qualquer destes seja controlador, sócio, acionista ou participante, de forma direta ou indireta, recebimento por palestras, aulas, atuação como proctor de treinamentos, remunerações, honorários pagos por participações em conselhos consultivos, de investigadores, ou outros comitês, etc. Provenientes da indústria farmacêutica, de órteses, próteses, equipamentos e implantes, brasileiras ou estrangeiras: - Viatris; Pfizer; AstraZeneca; NovoNordisk. B - Financiamento de pesquisas sob sua responsabilidade direta/pessoal (direcionado ao departamento ou instituição) provenientes da indústria farmacêutica, de órteses, próteses, equipamentos e implantes, brasileiras ou estrangeiras: - CSL Vifor. Outros relacionamentos Financiamento de atividades de educação médica continuada, incluindo viagens, hospedagens e inscrições para congressos e cursos, provenientes da indústria farmacêutica, de órteses, próteses, equipamentos e implantes, brasileiras ou estrangeiras: - Novo Nordisk; Viatris.
Luis Henrique Wolff Gowdak	Declaração financeira A - Pagamento de qualquer espécie e desde que economicamente apreciáveis, feitos a (i) você, (ii) ao seu cônjuge/ companheiro ou a qualquer outro membro que resida com você, (iii) a qualquer pessoa jurídica em que qualquer destes seja controlador, sócio, acionista ou participante, de forma direta ou indireta, recebimento por palestras, aulas, atuação como proctor de treinamentos, remunerações, honorários pagos por participações em conselhos consultivos, de investigadores, ou outros comitês, etc. Provenientes da indústria farmacêutica, de órteses, próteses, equipamentos e implantes, brasileiras ou estrangeiras: - Servier: síndrome coronariana crônica; Novartis: hipercolesterolemia. B - Financiamento de pesquisas sob sua responsabilidade direta/pessoal (direcionado ao departamento ou instituição) provenientes da indústria farmacêutica, de órteses, próteses, equipamentos e implantes, brasileiras ou estrangeiras: - Servier: síndrome coronariana crônica. Outros relacionamentos Financiamento de atividades de educação médica continuada, incluindo viagens, hospedagens e inscrições para congressos e cursos, provenientes da indústria farmacêutica, de órteses, próteses, equipamentos e implantes, brasileiras ou estrangeiras: - Servier: síndrome coronariana crônica.
Luiz Flávio Galvão Gonçalves	Nada a ser declarado
Luiz Francisco Rodrigues de Avila	Nada a ser declarado
Marcello Zapparoli	Outros relacionamentos Participação societária de qualquer natureza e qualquer valor economicamente apreciável de empresas na área de saúde, de ensino ou em empresas concorrentes ou fornecedoras da SBC: - Sócio de empresa de diagnóstico por imagem.
Marcelo Antonio Cartaxo Queiroga Lopes	Nada a ser declarado
Marcelo Souto Nacif	Nada a ser declarado
Marcelo Souza Hadlich	Nada a ser declarado
Marcio Sommer Bittencourt	Declaração financeira A - Pagamento de qualquer espécie e desde que economicamente apreciáveis, feitos a (i) você, (ii) ao seu cônjuge/companheiro ou a qualquer outro membro que resida com você, (iii) a qualquer pessoa jurídica em que qualquer destes seja controlador, sócio, acionista ou participante, de forma direta ou indireta, recebimento por palestras, aulas, atuação como proctor de treinamentos, remunerações, honorários pagos por participações em conselhos consultivos, de investigadores, ou outros comitês, etc. Provenientes da indústria farmacêutica, de órteses, próteses, equipamentos e implantes, brasileiras ou estrangeiras: - Cleerly Health; Elucid.
Marly Conceição Silva	Nada a ser declarado
Marly Maria Uellendahl Lopes	Nada a ser declarado
Mauricio Ibrahim Scanavacca	Declaração financeira A - Pagamento de qualquer espécie e desde que economicamente apreciáveis, feitos a (i) você, (ii) ao seu cônjuge/ companheiro ou a qualquer outro membro que resida com você, (iii) a qualquer pessoa jurídica em que qualquer destes seja controlador, sócio, acionista ou participante, de forma direta ou indireta, recebimento por palestras, aulas, atuação como proctor de treinamentos, remunerações, honorários pagos por participações em conselhos consultivos, de investigadores, ou outros comitês, etc. Provenientes da indústria farmacêutica, de órteses, próteses, equipamentos e implantes, brasileiras ou estrangeiras: - Daichii Sankyo: anticoagulação. B - Financiamento de pesquisas sob sua responsabilidade direta/pessoal (direcionado ao departamento ou instituição) provenientes da indústria farmacêutica, de órteses, próteses, equipamentos e implantes, brasileiras ou estrangeiras: - J&J: ablação de taquicardia ventricular. C - Financiamento de pesquisa (pessoal), cujas receitas tenham sido provenientes da indústria farmacêutica, de órteses, próteses, equipamentos e implantes, brasileiras ou estrangeiras: - ABBOT: ablação por cateter da cindam vaso vagal. Outros relacionamentos Financiamento de atividades de educação médica continuada, incluindo viagens, hospedagens e inscrições para congressos e cursos, provenientes da indústria farmacêutica, de órteses, próteses, equipamentos e implantes, brasileiras ou estrangeiras: - J&J: simpósio patrocinado pela indústria.
Nabil Ghorayeb	Nada a ser declarado
Otavio Rizzi Coelho-Filho	Declaração financeira A - Pagamento de qualquer espécie e desde que economicamente apreciáveis, feitos a (i) você, (ii) ao seu cônjuge/ companheiro ou a qualquer outro membro que resida com você, (iii) a qualquer pessoa jurídica em que qualquer destes seja controlador, sócio, acionista ou participante, de forma direta ou indireta, recebimento por palestras, aulas, atuação como proctor de treinamentos, remunerações, honorários pagos por participações em conselhos consultivos, de investigadores, ou outros comitês, etc. Provenientes da indústria farmacêutica, de órteses, próteses, equipamentos e implantes, brasileiras ou estrangeiras: - Pfizer: amiloidose/Tafamides; AstraZeneca: insuficiência cardíaca/Forxiga; Bayer: Firialta; Norvartis: insuficiência cardíaca/dislipidemia; EMS: insuficiência cardíaca. Outros relacionamentos Financiamento de atividades de educação médica continuada, incluindo viagens, hospedagens e inscrições para congressos e cursos, provenientes da indústria farmacêutica, de órteses, próteses, equipamentos e implantes, brasileiras ou estrangeiras: - Bayer; Pfizer; AstraZeneca.
Paulo R. Schvartzman	Nada a ser declarado
Paulo Savoia Dias da Silva	Nada a ser declarado
Pedro Vellosa Schwartzmann	Declaração financeira B - Financiamento de pesquisas sob sua responsabilidade direta/pessoal (direcionado ao departamento ou instituição) provenientes da indústria farmacêutica, de órteses, próteses, equipamentos e implantes, brasileiras ou estrangeiras: - MSD; Novartis; AstraZeneca; Alnylam; BridgeBio; Ionis; Lilly. Outros relacionamentos Financiamento de atividades de educação médica continuada, incluindo viagens, hospedagens e inscrições para congressos e cursos, provenientes da indústria farmacêutica, de órteses, próteses, equipamentos e implantes, brasileiras ou estrangeiras: - Pfizer; AstraZeneca; Novartis; Alnylam; NovoNordisk.
Raul D. Santos	Declaração financeira A - Pagamento de qualquer espécie e desde que economicamente apreciáveis, feitos a (i) você, (ii) ao seu cônjuge/companheiro ou a qualquer outro membro que resida com você, (iii) a qualquer pessoa jurídica em que qualquer destes seja controlador, sócio, acionista ou participante, de forma direta ou indireta, recebimento por palestras, aulas, atuação como proctor de treinamentos, remunerações, honorários pagos por participações em conselhos consultivos, de investigadores, ou outros comitês, etc. Provenientes da indústria farmacêutica, de órteses, próteses, equipamentos e implantes, brasileiras ou estrangeiras: - Libbs: dislipidemias; Novartis: dislipidemias; Novo-Nordisk: diabetes, Sanofi: dislipidemias; Eli-Lilly: diabetes. B - Financiamento de pesquisas sob sua responsabilidade direta/pessoal (direcionado ao departamento ou instituição) provenientes da indústria farmacêutica, de órteses, próteses, equipamentos e implantes, brasileiras ou estrangeiras: - Amgen: dislipidemias; Novartis: dislipidemias; Esperion: dislipidemias; Sanofi: dislipidemias; Ionis: dislipidemias; Eli-Lilly: dislipidemias; Kowa: dislipidemias; Amrit: dislipidemias. Outros relacionamentos Financiamento de atividades de educação médica continuada, incluindo viagens, hospedagens e inscrições para congressos e cursos, provenientes da indústria farmacêutica, de órteses, próteses, equipamentos e implantes, brasileiras ou estrangeiras: - Sanofi.
Ricardo Paulo de Sousa Rocha	Nada a ser declarado
Roberto Kalil Filho	Nada a ser declarado
Rodrigo Julio Cerci	Nada a ser declarado
Salvador Rassi	Nada a ser declarado
Sérgio Tavares Montenegro	Outros relacionamentos Participação em órgãos governamentais de regulação, ou de defesa de direitos na área de cardiologia: - Membro da Câmara Técnica da Cardiologia do Ministério da Saúde.
Silvio Henrique Barberato	Declaração financeira A - Pagamento de qualquer espécie e desde que economicamente apreciáveis, feitos a (i) você, (ii) ao seu cônjuge/ companheiro ou a qualquer outro membro que resida com você, (iii) a qualquer pessoa jurídica em que qualquer destes seja controlador, sócio, acionista ou participante, de forma direta ou indireta, recebimento por palestras, aulas, atuação como proctor de treinamentos, remunerações, honorários pagos por participações em conselhos consultivos, de investigadores, ou outros comitês, etc. Provenientes da indústria farmacêutica, de órteses, próteses, equipamentos e implantes, brasileiras ou estrangeiras: - Pfizer: amiloidose; Bristol: Camzyos; Boston: oclusão de apêndice atrial esquerdo.
Thiago Dieb Ristum Vieira	Nada a ser declarado
Tiago Augusto Magalhães	Declaração financeira A - Pagamento de qualquer espécie e desde que economicamente apreciáveis, feitos a (i) você, (ii) ao seu cônjuge/ companheiro ou a qualquer outro membro que resida com você, (iii) a qualquer pessoa jurídica em que qualquer destes seja controlador, sócio, acionista ou participante, de forma direta ou indireta, recebimento por palestras, aulas, atuação como proctor de treinamentos, remunerações, honorários pagos por participações em conselhos consultivos, de investigadores, ou outros comitês, etc. Provenientes da indústria farmacêutica, de órteses, próteses, equipamentos e implantes, brasileiras ou estrangeiras: - Conjuge - Novartis; Eli-Lilly; BMS; Knight; MSD; Daichii-Sankyo; AstraZeneca; Pint Pharma; GSK; Roche; Gilead; Adium; Pfizer: câncer de mama.
Tiago Senra Garcia dos Santos	Nada a ser declarado
Valéria de Melo Moreira	Nada a ser declarado
Walther Yoshiharu Ishikawa	Nada a ser declarado

**Sumário**

**1. Introdução** 12

1.1. Definição das Recomendações e Evidências 13

1.2. Definições de Escore de Risco Clínico e de Probabilidade Pré-teste 13

**1.2.1. Escore de Risco Clínico** 13

**1.2.2. Probabilidade Pré-teste** 13

**2. Tomografia Computadorizada Cardiovascular** 14

2.1. Escore de Cálcio Coronariano 17

**2.1.1. Evidências Atuais na Estratificação de Risco Cardiovascular pelo Escore de Cálcio Coronariano (EC)** 17

**2.1.2. Papel do Escore de Cálcio Coronariano na Reestratificação do Risco Definido pelos Escores Clínicos Tradicionais** 20

**2.1.3. Uso do Escore de Cálcio no Suporte na Decisão de Terapia Farmacológica** 21

2.2. Angiotomografia de Coronárias na Suspeita de Angina Estável sem DAC Conhecida 23

**2.2.1. Como Opção de Primeira Escolha na Avaliação de Dor Torácica Não Aguda** 23

**2.2.2. Em Pacientes de Baixo Risco com Testes Funcionais Positivos** 24

2.3. Na Pesquisa de Etiologia Isquêmica de Insuficiência Cardíaca 25

2.4. Angiotomografia de Coronárias na Suspeita de Angina Estável com DAC Conhecida 25

**2.4.1. Portadores de Stents** 25

**2.4.2. Revascularizados** 25

2.5. Seguimento de Coronariopatas em Tratamento Clínico 25

2.6. Angiotomografia das Artérias Coronárias na Avaliação de Outros Cenários Relacionados à Doença Arterial Coronariana 27

2.7. Anomalias de Artérias Coronárias 27

2.8. Angiotomografia de Coronárias na Suspeita de Dor Torácica Aguda 28

**2.8.1. Descarte Triplo** 29

2.9. Angiotomografia de Coronárias na Avaliação Pré-operatória 30

2.10. Avaliação de Valvopatias pela Angiotomografia 30

2.11. Avaliação Pré-implante Percutâneo de Valva Aórtica (TAVI/ViV) 31

2.12. Planejamento Percutâneo de Outras Alterações Estruturais 32

2.13. Avaliação das Veias Cardíacas, Átrio Esquerdo e Avaliação de Veias Pulmonares (Incluindo Planejamento de Ablação de Fibrilação Atrial/Oclusão Apêndice Atrial) 32

2.14. Avaliação Funcional por Tomografia Computadorizada 33

**2.14.1. Perfusão Miocárdica por Tomografia Computadorizada** 33

**2.14.2. Reserva de Fluxo Fracionado por Tomografia Computadorizada (FFR-TC)** 35

2.15. Tomografia na Avaliação das Cardiomiopatias Não Isquêmicas 36

**2.15.1. Tomografia na Avaliação de Função Ventricular** 36

**2.15.2. Avaliação de Caracterização Tecidual Miocárdica pela Técnica de Realce Tardio** 36

**2.15.3. Avaliação de Volume Extracelular Miocárdico pela Tomografia** 37

2.16. Tomografia na Avaliação das Doenças Pericárdicas 37

2.17. Tomografia na Avaliação de Massas/Trombos Cardíacos 39

2.18. Doenças Vasculares 40

**2.18.1. Aorta** 40

**2.18.2. Carótidas Extracranianas** 42

**2.18.3. Artérias Renais** 42

**2.18.4. Doença Vascular Periférica** 42

**2.18.5. Artérias Pulmonares** 43

**2.18.6. Artérias Viscerais** 43

**3. Ressonância Magnética Cardiovascular** 43

3.1. Uso dos Mapas Multiparamétricos no Diagnóstico Diferencial das Miocardiopatias 45

3.2. Pesquisa de DAC pela Ressonância Magnética – Isquemia Miocárdica 48

3.3. Pesquisa de DAC pela Ressonância Magnética – Viabilidade Miocárdica 51

3.4. Angiorressonância das Artérias Coronárias 54

3.5. Diagnóstico Diferencial de Troponina Positiva com Coronárias Normais (TP-NOCA/MINOCA) 55

3.6. Cardiomiopatia Induzida por Estresse (Takotsubo) 55

3.7. Miocardites/Cardiomiopatias Inflamatórias 56

3.8. Coração de Atleta 57

3.9. Cardiomiopatia Hipertrófica 57

3.10. Endomiocardiofibrose 58

3.11. Amiloidose Cardíaca 59

3.12. Hemossiderose Cardíaca 60

3.13. Outras Doenças de Depósito Miocárdico 61

3.14. Cardiomiopatia Chagásica 62

3.15. Cardiomiopatia Arritmogênica do Ventrículo Direito 62

3.16. Sarcoidose 64

3.17. Miocárdio Não Compactado/Trabeculação Excessiva do Ventrículo Esquerdo 65

3.18. Distrofias Musculares 65

3.19. Cardiomiopatia Periparto 66

3.20. Cardiomiopatia Associada a Doenças Sistêmicas 66

3.21. Alterações Cardíacas Associadas ao Transplante Cardíaco 67

3.22. Doenças do Pericárdio 67

3.23. Massas Cardíacas e Trombo 70

3.24. Doenças Valvares 72

3.25. Cardio-oncologia 74

3.26. Doenças Vasculares 75

**3.26.1. Aorta** 75

**3.26.2. Carótidas Extracranianas** 76

**3.26.3. Artérias Renais** 76

**3.26.4. Artérias Pulmonares** 77

**3.26.5. Artérias Viscerais** 77

**4. Cardiopatias Congênitas** 77

**4.1. Tomografia Computadorizada na Avaliação de Cardiopatias Congênitas** 77

**4.1.1. Avaliação de *Shunts* Intra e Extracardíacos** 77

***4.1.1.1. Comunicação Interatrial e Interventricular*** 77

***4.1.1.2. Conexão Venosa Anômala Parcial e Total*** 78

**4.1.2. Lesões Congênitas Valvares** 78

*4.1.2.1. Valva Tricúspide/Anomalia de Ebstein* 78

*4.1.2.2. Valva Pulmonar* 79

*4.1.2.3. Valva Mitral* 80

*4.1.2.4. Valva Aórtica* 80

*4.1.3. Anomalias Conotruncais* 81

*4.1.3.1. Tetralogia de Fallot* 81

*4.1.3.2. Dupla Via de Saída de Ventrículo Direito* 81

*4.1.3.3. Tronco Arterial Comum* 81

***4.1.3.4. Transposição das Grandes Artérias*** 82

***4.1.3.5. Transposição Corrigida das Grandes Artérias*** 82

**4.1.4. Anomalias da Aorta Torácica** 82

***4.1.4.1. Coarctação e Outras Anormalidades da Aorta*** 82

**4.1.5. Coração Univentricular** 83

**4.1.6. Miscelânea** 85

4.2. Ressonância Magnética Cardiovascular em Cardiopatias Congênitas 85

**4.2.1. Avaliação de Shunts Intra e Extracardíacos** 86

***4.2.1.1. Comunicação Interatrial*** 86

***4.2.1.2. Forame Oval Patente*** 86

***4.2.1.3. Conexão Anômala de Veias Pulmonares Parcial e Total*** 86

***4.2.1.4. Comunicação Interventricular (CIV)*** 87

***4.2.1.5. Defeito de Septo Atrioventricular*** 87

***4.2.1.6. Persistência do Canal Arterial*** 87

**4.2.2. Lesões Congênitas Valvares** 87

***4.2.2.1. Valva Tricúspide/Anomalia de Ebstein*** 87

***4.2.2.2. Valva Mitral*** 88

***4.2.2.3. Estenose Mitral*** 89

***4.2.2.4. Valva Pulmonar*** 89

***4.2.2.5. Valva Aórtica*** 89

**4.2.3. Anomalias Conotruncais** 90

***4.2.3.1. Tetralogia de Fallot*** 90

***4.2.3.2. Dupla Via de Saída do Ventrículo Direito*** 90

***4.2.3.3. Tronco Arterioso Comum*** 91

***4.2.3.4. Transposição das Grandes Artérias*** 91

***4.2.3.5. Transposição Corrigida das Grandes Artérias*** 91

**4.2.4. Anomalias da Aorta Torácica** 92

***4.2.4.1. Coarctação de Aorta*** 92

***4.2.4.2. Outras Anomalias da Aorta*** 93

**4.2.5. Coração Univentricular** 93

**4.2.6. Miscelânea** 94

**Referências** 95

## Prefácio

No Brasil, as doenças cardiovasculares representam uma causa importante de mortalidade. No período entre 2010 e 2019, 28% das fatalidades registradas pelo Departamento de Informação e Informática do Sistema Único de Saúde (DATASUS)^1^ decorreram desse grupo de patologias. Dentro das medidas envolvidas no enfrentamento a esse problema de saúde pública, as estratégias de utilização racional de recursos vêm recebendo contribuição importante com o desenvolvimento de técnicas de diagnóstico por imagem. Notadamente, a tomografia computadorizada e a ressonância magnética têm ampliado o seu potencial dentro do arsenal diagnóstico e prognóstico na doença cardiovascular, bem como vêm oferecendo bases para o planejamento de diferentes modalidades de procedimentos terapêuticos (cirúrgicos e/ou minimamente invasivos).

A área da imagem cardiovascular encontra-se em evolução exponencial. Nos últimos anos, o surgimento e o aprimoramento de novas técnicas relacionadas à detecção de doença arterial coronariana, bem como aquelas relacionadas ao estudo anatômico e funcional do miocárdio, permitiram a expansão das indicações de tomografia computadorizada e ressonância magnética cardíaca no manejo do paciente com cardiopatia. Paralelamente, resultados de grandes estudos multicêntricos permitiram definições mais assertivas na utilização desses métodos em cenários específicos, ratificando e retificando indicações anteriormente inconclusivas.

Acompanhando os desenvolvimentos no campo diagnóstico, os procedimentos terapêuticos (percutâneo e/ou cirúrgicos) em cardiologia também contaram com o desenvolvimento de técnicas que ampliaram as suas indicações no tratamento de diversas patologias. A indicação de tais procedimentos veio acompanhada da necessidade de um maior detalhamento anatômico e exatidão no diagnóstico para o seu emprego adequado. Esse cenário também foi agente potencializador do uso da tomografia computadorizada e ressonância magnética cardíaca como recursos auxiliares no manejo clínico dos pacientes.

A II Diretriz de Tomografia e Ressonância Cardiovascular,^2^ publicada em 2014 pela Sociedade Brasileira de Cardiologia em conjunto com o Colégio Brasileiro de Radiologia, trouxe as evidências mais robustas disponíveis para a aplicação de ambos os métodos em diferentes cenários clínicos. Na ocasião, também lançou luz sobre as técnicas em desenvolvimento nas diferentes áreas, mesmo que ainda não amplamente validadas por grandes estudos. Dessa forma, essa atualização visa revisitar as indicações propostas pelo documento anterior, bem como contextualizar os avanços dessas modalidades, qualificando-os com os respectivos níveis de evidência e graus de recomendação nas variadas aplicações. O objetivo final deste documento é ser uma fonte de consulta ao cardiologista, fornecendo informação atualizada e pautada na melhor evidência disponível para ser utilizada de forma prática e dirigida aos questionamentos da rotina clínica.

## 1. Introdução

Desde a última diretriz sobre tomografia computadorizada (TC) e ressonância magnética cardíaca (RMC) da Sociedade Brasileira de Cardiologia (SBC), novas tecnologias e diversos estudos científicos envolvendo esses métodos diagnósticos, incluindo estudos multicêntricos e randomizados, contribuíram para reforçar as indicações previamente existentes, assim como mostrar novas contribuições desses exames dentro da cardiologia.

A TC cardiovascular e a RMC, métodos relativamente recentes na cardiologia, causaram uma revolução no entendimento e no tratamento das cardiopatias. A TC do coração permitiu a detecção da aterosclerose coronariana em seus estágios mais precoces, mostrando o importante valor prognóstico da doença arterial coronariana (DAC) não obstrutiva, antes subestimada pelas diversas sociedades de cardiologia em todo o mundo, e reforçando o valor da anatomia para guiar o tratamento da DAC obstrutiva, sendo a opção inicial na investigação de pacientes sintomáticos sem DAC conhecida. Apesar de inicialmente ser um exame anatômico, a TC vem mostrando ser cada vez mais um exame completo na avaliação das cardiopatias. A análise da isquemia pela TC através da perfusão miocárdica sob estresse farmacológico e/ou pela reserva de fluxo fracionada (FFR, de *fractional flow reserve* ) por TC pode ser uma alternativa validada aos outros exames de isquemia ou complementar à informação anatômica nas estenoses com repercussão funcional indeterminada.^3 – 8^ Outra área de avanço da TC foi na avaliação das cardiopatias estruturais, como nas valvopatias, possibilitando aos cardiologistas melhor seleção dos pacientes para procedimentos terapêuticos menos invasivos com maiores taxas de sucesso e menores riscos de complicações.^9^

A RMC tem a vantagem de ser um exame sem radiação ionizante e abrangente nas diversas análises morfológicas e funcionais cardíacas, tendo ampliado o arsenal diagnóstico na avaliação da DAC, com alta acurácia diagnóstica para detecção de isquemia miocárdica e considerada o padrão-ouro na análise da função ventricular, do infarto e da viabilidade miocárdica, capaz de avaliar todos esses parâmetros em um único exame.^2 , 10 – 13^ Já na avaliação das cardiomiopatias não isquêmicas, a RM se tornou um exame fundamental, auxiliando no diagnóstico e no prognóstico dessas doenças e fornecendo informações para o manejo terapêutico de diversas delas.^2 , 14 , 15^

Com o objetivo de ser uma referência para o uso desses métodos na rotina clínica e baseada nas melhores evidências científicas disponíveis, a SBC e o Colégio Brasileiro de Radiologia (CBR) elaboraram este documento a fim de auxiliar os médicos na indicação desses exames para uma melhor decisão clínica em benefício dos pacientes.

### 1.1. Definição das Recomendações e Evidências

Em linha com os demais documentos elaborados por diferentes entidades e sociedades médicas nacionais e internacionais, as informações contidas neste documento são pautadas em indicações baseadas em Classes de Recomendação e Níveis de Evidência. Representados pela indicação em diferentes cenários clínicos e/ou patologia específica, a utilização de cada um dos métodos é individualizada para cada tema proposto neste documento.

De maneira simplificada, a Classe de Recomendação trata do posicionamento consensual sobre utilidade e benefício de determinado procedimento, observando-se a segurança e eficácia de seu emprego com base nas melhores evidências disponíveis. O Nível de Evidência define a qualidade dos estudos que subsidiaram tais recomendações, incluindo desde opinião de especialistas a ensaios clínicos randomizados.

**Classes de Recomendação** ^2^ **:**

**Classe I:** Condições para as quais há evidências conclusivas ou, na sua falta, consenso geral de que o procedimento é seguro e útil/eficaz.
**Classe II:** Condições para as quais há evidências conflitantes e/ou divergência de opinião sobre segurança e utilidade/eficácia do procedimento.
  - **Classe IIa:** Peso ou evidência/opinião a favor do procedimento. A maioria aprova.
  - **Classe IIb:** Segurança e utilidade/eficácia menos bem estabelecida, não havendo predomínio de opiniões a favor.
**Classe III:** Condições para as quais há evidências e/ou consenso de que o procedimento não é útil/eficaz e, em alguns casos, pode ser prejudicial.

**Níveis de Evidência** ^2^ **:**

**Nível A:** Dados obtidos a partir de múltiplos estudos randomizados de bom porte concordantes e/ou de metanálise robusta de estudos clínicos randomizados.
**Nível B:** Dados obtidos a partir de metanálise menos robusta, a partir de um único estudo randomizado ou de estudos não randomizados (observacionais).
**Nível C:** Dados obtidos a partir de opiniões consensuais de especialistas.

### 1.2. Definições de Escore de Risco Clínico e de Probabilidade Pré-teste

#### 1.2.1. Escore de Risco Clínico

Os escores de risco clínico são ferramentas que ajudam a avaliar a probabilidade de um indivíduo assintomático desenvolver uma doença aterosclerótica cardiovascular em um período determinado (geralmente 10 anos) e são calculados através dos fatores de risco (FR) apresentados e baseados em análises populacionais. O uso dos escores de risco é importante na escolha da terapia preventiva apropriada, ajustando a intensidade da terapia prescrita ao risco estimado do paciente, o que permite, assim, potencializar o benefício das medicações nos pacientes de maior risco e evitar o uso desnecessário e/ou excessivo naqueles de menor risco.

O escore de risco de Framingham foi usado durante muitos anos em diversos países e estima o risco de infarto e morte coronariana em 10 anos, porém, atualmente, vem sendo substituído por outros escores de risco, como o Escore de Risco Global, o Escore de Risco para Doença Aterosclerótica Cardiovascular (ASCVD Risk Estimator), o Escore de Risco de Reynolds ou o SCORE (Systematic COronary Risk Evaluation), por exemplo.^16 – 19^ A SBC recomenda a utilização do Escore de Risco Global, que estima o risco de infarto do miocárdio, acidente vascular encefálico, insuficiência cardíaca (IC) ou insuficiência vascular periférica em 10 anos.^16 , 17^

#### 1.2.2. Probabilidade Pré-teste

A capacidade de um exame confirmar ou excluir corretamente uma doença vai depender da sua acurácia diagnóstica e da prevalência dessa doença na população investigada. Dessa forma, a escolha do exame adequado para determinada população é fundamental para evitar resultados falso-negativos e falso-positivos.

No caso da investigação da DAC, diversos exames podem ser utilizados e têm sua acurácia diagnóstica já bem estabelecida na literatura, sendo a escolha de cada um deles realizada levando em consideração a prevalência da DAC, além da disponibilidade e experiência locais e características específicas dos pacientes que podem limitar a análise de algum exame específico.^8 , 20 , 21^

Para avaliar a prevalência da DAC na população investigada, usamos os dados dos pacientes, seus antecedentes pessoais e exames anteriores, o exame físico e principalmente as características dos sintomas relatados, estimando, assim, a probabilidade pré-teste da DAC. Essa probabilidade pode ser também analisada de forma mais objetiva por escores já validados para auxiliar os médicos na decisão clínica sobre o exame a ser solicitado. Um escore antigo bastante utilizado é o modelo proposto por Diamond-Forrester; porém, estudos recentes mostraram sua superestimação da probabilidade pré-teste da DAC, podendo outros escores mais atuais como o CAD Consortium (Coronary Artery Disease Consortium) e suas variantes fornecer estimativas mais apropriadas dessa prevalência.^8 , 21 – 24^

Apesar dessas discrepâncias entre os diferentes escores nas estimativas de probabilidades pré-teste, uma alternativa de estratificação de suspeita de DAC pode ser utilizada da seguinte forma:^2 , 25^

- **Probabilidade pré-teste baixa:** < 10% de probabilidade de DAC
- **Probabilidade pré-teste intermediária:** entre 10 e 90% de probabilidade de DAC
- **Probabilidade pré-teste alta:** > 90% de probabilidade de DAC

## 2. Tomografia Computadorizada Cardiovascular

A TC do coração tem ganhado cada vez mais aplicações e indicações com o surgimento de novas tecnologias, a publicação de estudos científicos e a experiência dos médicos envolvidos. Inicialmente, era um exame realizado sem contraste para avaliação do escore de cálcio coronariano e, posteriormente, com os tomógrafos de 64 detectores, houve a expansão da avaliação anatômica não invasiva das artérias coronárias. Hoje, é um método que oferece uma avaliação multimodalidade na Cardiologia, com possibilidade da análise de diferentes estruturas e parâmetros anatômicos e fisiológicos, como avaliação de função e volumes das câmaras cardíacas, identificação de isquemia miocárdica através perfusão miocárdica (sob estresse farmacológico ou pela FFR, ferramenta que avalia a repercussão funcional de estenoses coronarianas) por tomografia, avaliação de infarto e viabilidade miocárdica pelo realce tardio (RT) por tomografia, análise das veias cardíacas e das veias pulmonares, avaliação de valvopatias, cardiopatias congênitas e análise de cardiomiopatias não isquêmicas como alternativas a outros métodos diagnósticos.^2 , 3 , 8 , 25^

Essa expansão do uso da TC do coração é um dos motivos da atualização desta nova Diretriz, sendo discutida nos tópicos específicos ao longo desta publicação e de forma introdutória a seguir para ajudar os médicos solicitantes sobre suas aplicações e limitações.

**Figura 1:**
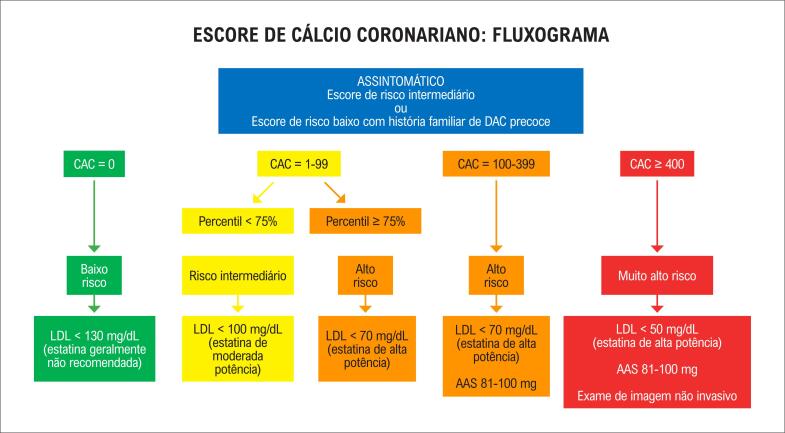
Utilização do escore de cálcio coronariano (CAC) como ferramenta de estratificação de risco e suporte em conduta clínica. DAC: doença arterial coronariana; LDL: lipoproteína de baixa densidade; AAS: ácido acetilsalicílico.

Uma das aplicações da TC do coração é o escore de cálcio (EC) coronariano, método validado em pacientes assintomáticos para a reestratificação do risco cardiovascular. O EC é um exame não invasivo, rápido, sem o uso de contraste iodado e com baixa dose de radiação ionizante (cerca de 1 mSv), que tem como objetivo detectar e quantificar a calcificação arterial coronariana (CAC), sendo um preditor independente de mortalidade, de eventos coronarianos e de isquemia miocárdica.^2 , 26 – 30^ As informações fornecidas pelo EC sobre a carga de aterosclerose coronariana permitem individualizar o risco cardiovascular fornecido pelos escores de risco clínico baseados em dados populacionais, reestratificando o risco cardiovascular melhor do que qualquer outro método com esse objetivo em pacientes assintomáticos e possibilitando aos clínicos adequarem a terapia preventiva e aumentarem a aderência medicamentosa nos pacientes com indicação do uso de medicações.^31 – 34^ Uma sugestão de como o CAC pode auxiliar na reestratificação de risco é ilustrada no fluxograma da Figura 1 .^26 – 34^

O principal exame incluído dentro da definição de TC do coração é a angiotomografia (angio-TC) das artérias coronárias, exame bem validado na investigação da DAC com alta acurácia diagnóstica e valor prognóstico. Em relação à sua realização, é um exame que utiliza contraste iodado, preferencialmente não iônico pelo menor risco de complicações, necessitando de um acesso venoso periférico para a injeção em alto fluxo (4 a 6 mL/s) de um baixo volume de contraste (cerca de 60 a 70 mL) em comparação a outras tomografias.

O exame de angio-TC coronária também usa radiação ionizante, tendo nas últimas décadas havido redução significativa da dose de radiação com tomógrafos mais modernos e avanços tecnológicos em tomógrafos convencionais. A dose média de radiação em uma aquisição retrospectiva com modulação de dose é de cerca de 9 mSv em tomógrafos de 64 detectores; porém, em tomógrafos mais modernos, esse mesmo tipo de aquisição tem dose média de 5 mSv, sendo ainda menores quando usados outros tipos de aquisição como a prospectiva (< 3 mSv) e a com alto *pitch* (< 1 mSv).^2 , 35^

Nos protocolos para a realização da angio-TC coronária, o uso de medicações cronotrópicas negativas para a redução da frequência cardíaca (< 60 bpm) e o uso de nitratos sublinguais para a vasodilatação coronária são estratégias que aumentam a qualidade e a acurácia diagnóstica dos exames e permitem reduzir ainda mais a dose de radiação na escolha de formas de aquisição que possam ser utilizadas com frequências cardíacas mais baixas.^2 , 36^ Como o exame é sincronizado ao eletrocardiograma para formação das imagens, pacientes com arritmias ou alta frequência cardíaca podem ter imagens não diagnósticas, principalmente em tomógrafos convencionais. Outro ponto fundamental na aquisição das imagens da angio-TC coronária é a realização de apneia inspiratória pelos pacientes (< 15 segundos), orientação necessária para manter o diafragma e a topografia do coração inalteradas no tórax durante o exame.^2 , 36^

Entre os exames utilizados no diagnóstico da DAC, a angio-TC coronária é uma alternativa como um exame de imagem não invasivo em pacientes com probabilidade pré-teste baixa ou intermediária que vem ganhando cada vez mais indicações com a publicação de estudos científicos, inclusive de estudos prospectivos, randomizados e multicêntricos.^2 , 3 , 8 , 23 , 25 , 37 – 39^ No estudo PROMISE, publicado em 2015, 10.003 pacientes com suspeita de DAC estável foram randomizados para realizar angio-TC coronária ou testes de isquemia, mostrando um número similar de desfecho primário (morte, infarto, internação por angina instável e complicação importante relacionada ao procedimento) nos dois grupos ao final dos 2 anos de seguimento (3,3% vs. 3,0%, respectivamente). Apesar do número parecido de exames positivos em ambos os grupos (10,7% vs. 11,7%), o grupo da angio-TC coronária indicou mais pacientes à cinecoronariografia dentro de 90 dias (12,2% vs. 8,1%) e à revascularização miocárdica (6,2% vs. 3,2%, respectivamente). Dados interessantes do estudo PROMISE mostram uma menor porcentagem de cinecoronariografia sem estenoses significativas no grupo angio-TC (27,9% vs. 52,5%). Embora tenha sido considerado um estudo neutro (desfecho primário combinado não foi diferente entre os grupos), observou-se uma redução significativa (34%) no desfecho composto de morte e infarto no final dos primeiros 12 meses de seguimento no grupo da angio-TC coronária ( *hazard ratio* [HR] 0,66 e p = 0,049).^23^

Outro estudo comparando a angio-TC coronária e os testes de isquemia foi o SCOT-HEART, em que 4.146 pacientes com suspeita de angina estável foram randomizados para realizar angio-TC ou teste ergométrico. Uma orientação importante no protocolo deste estudo foi que, no grupo da angio-TC, os pacientes que apresentavam DAC obstrutiva e não obstrutiva teriam que receber terapia medicamentosa, enquanto no grupo dos testes de isquemia, não adequados na detecção da DAC não obstrutiva, a terapia medicamentosa nos pacientes com resultados negativos foi orientada pelo escore de risco clínico local. Apesar de um número semelhante de pacientes indicados à cinecoronariografia e à revascularização, o grupo da angio-TC detectou uma maior porcentagem de pacientes com DAC obstrutiva, introduziu mais medicação preventiva e antianginosa e reduziu de forma significativa (50%) o número de infartos fatal e não fatal nos primeiros 20 meses de seguimento e o número de morte cardiovascular e infarto (41%) no final do seguimento de 5 anos.^37 , 38^

Um estudo muito esperado no tratamento da DAC estável foi o ISCHEMIA, em que 5.179 pacientes com angina estável com isquemia moderada ou grave por exames funcionais foram randomizados para uma estratégia invasiva (cinecoronariografia e revascularização quando possível) ou uma estratégia conservadora (tratamento clínico inicial e cinecoronariografia se falha da terapia medicamentosa). Os pacientes do estudo realizaram angio-TC coronária para avaliar estenose obstrutiva (≥ 50%) do tronco de coronária esquerda, o qual era um fator de exclusão do estudo, e o seu resultado era cego aos cardiologistas que acompanhavam os pacientes remanescentes. O estudo mostrou que o desfecho primário de morte cardiovascular, infarto e internação por angina instável, IC ou morte súbita abortada foi semelhante em ambos os grupos, mostrando a segurança do tratamento clínico nesta população sem estenose obstrutiva do tronco de coronária esquerda pela tomografia.^39^ Uma análise posterior dos dados do estudo ISCHEMIA mostrou que a gravidade das estenoses coronárias pela tomografia foram associados a um maior risco clínico, mas não à gravidade da isquemia após o ajuste para a gravidade da anatomia.^40^

**Figura 2:**
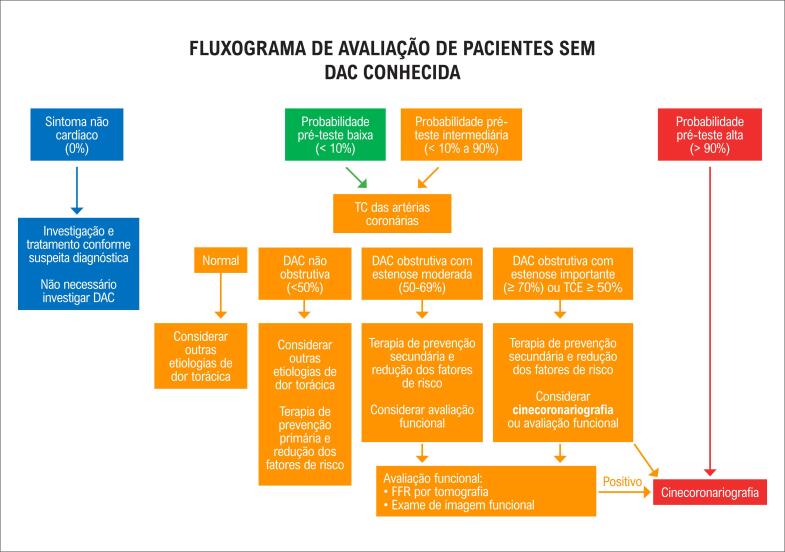
Fluxograma para avaliação de pacientes sem DAC conhecida. TC: tomografia computadorizada; TCE: tronco da coronária esquerda; FFR: reserva de fluxo fracionada.

Diante das evidências atuais, a angio-TC coronária é um exame bem validado não apenas pelo seu valor diagnóstico e prognóstico na DAC, mas também por uma melhor reestratificação do risco cardiovascular e orientação das decisões clínicas nos pacientes com DAC, sendo uma opção inicial adequada nesta avaliação, principalmente nos pacientes sem DAC conhecida, como indicado em outras diretrizes internacionais.^3 , 8^ Sugestões de fluxogramas na investigação de pacientes sem DAC conhecida e com DAC conhecida podem ser visualizados nas Figuras 2 e 3 , respectivamente.^3 , 8 , 41 , 42^

**Figura 3:**
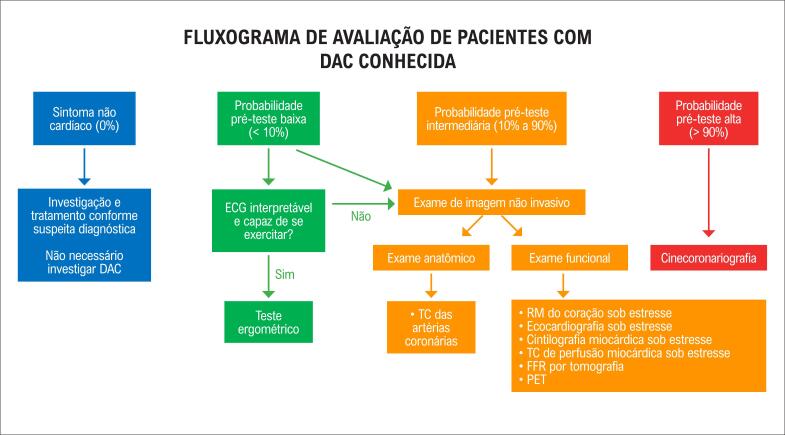
Fluxograma de avaliação de doença arterial coronariana (DAC) em pacientes com DAC conhecida.

Modalidades da TC do coração que também podem ser usadas na avaliação da DAC são a detecção de isquemia miocárdica pela perfusão miocárdica sob estresse farmacológico ou pela FFR por tomografia.^3^ A perfusão miocárdica sob estresse farmacológico, geralmente realizada por vasodilatadores como a adenosina e o dipiridamol, é validada há cerca de duas décadas, tanto com valor diagnóstico como prognóstico. Em um estudo do grupo CORE320, a perfusão miocárdica por tomografia (sem associação aos dados anatômicos da angio-TC coronária) em relação à cintilografia miocárdica (tomografia computadorizada por emissão de fóton único [SPECT]) mostrou uma melhor acurácia diagnóstica quando comparada à cinecoronariografia invasiva, apresentando sensibilidades superiores no diagnóstico da estenose significativa do tronco de coronária esquerda (TC 92% vs. SPECT 75%), DAC triarterial (TC 92% vs. SPECT 79%), DAC biarterial (TC 89% vs. SPECT 68%) e DAC uniarterial (TC 83% vs. SPECT 41%).^43^

A avaliação de isquemia miocárdica pela FFR por tomografia (FFR-TC), método mais recente na cardiologia e validado em estudos clínicos, tem a vantagem de avaliar o significado funcional das estenoses coronarianas sem a necessidade de estresse farmacológico ou doses extras de contraste iodado e radiação, porém exigindo o emprego de *softwares* direcionados para esta análise adicional. A FFR-TC pode, assim, auxiliar na tomada de decisão em pacientes, por exemplo, com estenoses moderadas, mantendo-os em tratamento clínico quando negativo ou solicitando uma cinecoronariografia invasiva quando positivo para prosseguir a investigação diagnóstica.^5 – 7^

Os outros diferentes usos da TC do coração estão mais bem detalhados em sessões específicas desta Diretriz, mostrando sua característica de multimodalidade dentro da Cardiologia.

### 2.1. Escore de Cálcio Coronariano

#### 2.1.1. Evidências Atuais na Estratificação de Risco Cardiovascular pelo Escore de Cálcio Coronariano (EC)

Em 1990, Arthur Agatston utilizou um tomógrafo de feixe de raios de elétrons EBCT ( *electron beam computed tomography* ) para identificar e quantificar aterosclerose coronariana calcificada.^44^ Desde então, após mais de três décadas de publicações e seguimento de várias coortes populacionais numerosas, a quantificação de carga aterosclerótica, através do EC, consolidou-se como a melhor ferramenta adicional para predição de risco de eventos cardiovasculares maiores entre as atualmente disponíveis de maneira ampla na prática clínica.^45^

#### 2.1.1.1. Técnica

- O exame de EC é realizado sem a utilização de contraste, habitualmente em tomógrafos helicoidais com múltiplas fileiras de detectores, que apresentam utilidade clínica mais ampla que os tomógrafos EBCT. Imagens axiais são adquiridas cobrindo toda a área cardíaca, no sentido crânio-caudal, com sincronização com o eletrocardiograma (ECG). A duração de todo o exame é de cerca de 10 minutos, e a dose atual de radiação é extremamente baixa, ficando em torno de 0,8 a 1 mSv.^46^
- O exame detecta o componente calcificado das placas de ateroma, e a sua quantificação pode ser realizada de diversas maneiras (escores de Agatston, volume, densidade, entre outros). O escore de Agatston é o mais utilizado na prática clínica pois é referência para bancos de dados populacionais como os estudos *Multi-Ethnic Study of Atherosclerosis* (MESA) e *Framingham Heart Study* (FHS).^47^

#### 2.1.1.2. Marcador de Risco Independente para Eventos Cardiovasculares

Estudos iniciais ajudaram a determinar o papel do EC, como o *South Bay Heart Watch Study* ,^46^ que mostrou que o EC reestratificava pacientes de risco intermediário pelo escore de risco de Framingham e o *St. Francis Heart Study* que, em 2005, mostrava risco cardiovascular bem mais elevado quando comparou-se grupos com EC > 400 *versus* EC zero.^48^

Estudos de coorte maiores, com pacientes assintomáticos em prevenção primária, foram realizados nos últimos 15 anos. Estudos como os norte-americanos MESA,^49^ que seguiu de maneira prospectiva 6.814 pacientes com idade entre 45-84 anos; CAC Consortium (maior coorte da literatura com 66.363 pacientes);^50^ *Dallas Heart Study* (DHS);^51^ o alemão *Heinz Nixdorf Recall Study* (HNR), com 4.814 pacientes entre 45-74 anos^52^; e o holandês Rotterdam Study,^53^ com 7.983 pacientes um pouco mais velhos (idade > 55 anos) reforçaram conceitos como o EC ser mais elevado em homens, haver diferenças entre etnias e aumentar com a idade, contribuindo para um melhor entendimento do processo de aterosclerose coronariana.

Esses estudos também ajudaram a estabelecer a relação entre o EC e o risco cardiovascular. Por exemplo, Detrano et al., na população do MESA, mostraram que o EC é relacionado, de maneira independente, à incidência de eventos cardiovasculares, e o fato de o EC dobrar representou um aumento de 25% na probabilidade de eventos cardiovasculares maiores, em um período de seguimento de 3,8 anos.^54^

#### 2.1.1.3. Conceitos Básicos

1. Quanto maior a carga de aterosclerose detectada pelo EC, maior o risco de existência de DAC obstrutiva e de eventos cardiovasculares maiores. Isso ocorre de maneira semelhante em todas as populações estudadas.
2. Interpretação de um EC positivo: escores positivos podem ser classificados em EC baixos (1-100), moderados (101-400), altos (> 400) e muito altos (> 1.000) ( Tabela 1 ).

**Tabela 1:** Riscos relativos (RR) para eventos cardiovasculares e grau de calcificação de acordo com valores absolutos do escore de cálcio (EC)

Valores absolutos do EC	RR para eventos cardiovasculares	Grau de calcificação
0		Ausência de calcificação
1-100	1,9 (1,3-2,8)	Discreto
101-400	4,3 (3,1-6,1)	Moderado
401-1.000	7,2 (5,2-9,9)	Alto
> 1.000	10,8 (4,2-27,7)	Muito alto

* Adaptado de Azevedo, Rochitte e Lima^55^ e Greenland et al.^56^

Tem interesse clínico especialmente um escore de Agatston acima de 100 ou um percentil acima de 75 do esperado para sexo, idade e etnia (útil em pacientes jovens, com valores absolutos ainda abaixo de 100), configurando-se como fator agravante de risco cardiovascular e reestratificando o paciente para uma categoria de risco acima da obtida com a utilização da ferramenta clínica utilizada (Escore de Risco Global, "10- year atherosclerotic cardiovascular disease", ASCVD, Framingham etc).^16^ Cabe ressaltar que, em pacientes com diabete melito, o limiar de corte é menor, com EC acima de 10 já sendo considerado como fator de risco adicional.^57^ O valor absoluto tem uma associação mais forte com eventos cardiovasculares maiores do que o percentil, pelo menos a curto/médio prazo.^58^ Já EC bem elevados, acima de 400 de Agatston, estão relacionados a uma maior incidência de isquemia detectável e a um risco de eventos semelhante ao de pacientes sintomáticos.^59^

#### 2.1.1.4. População-alvo

Pacientes assintomáticos, em prevenção primária e com risco clínico considerado limítrofe ou intermediário são os que mais se beneficiam da realização do exame.^60 , 61^

Deve-se lembrar aqui que os escores que utilizam os FR tradicionais tendem a superestimar o risco cardiovascular quando comparados com a incidência de eventos na população incluída em estudos mais recentes,^62^ podendo levar a uma categorização em que haverá maior utilização de fármacos e exames complementares, com benefício clínico questionável.

Populações com FR não contempladas nos escores tradicionais, como diabetes melito (DM), hipercolesterolemia familiar (HF) e com antecedente familiar positivo para DAC precoce, também apresentam potenciais benefícios clínicos na mensuração da carga de placas através do EC.^63 – 67^

Pacientes sintomáticos, no serviço de emergência, não devem utilizar de maneira habitual o exame, devido ao potencial mecanismo envolvido (ruptura de placa, trombose, placa não calcificada) não poder ser devidamente caracterizado nesse exame. Além disso, mesmo no cenário ambulatorial, também não se recomenda o uso do EC em indivíduos sintomáticos. Apesar de sabermos que um EC zero tem valor preditivo negativo muito alto e bom prognóstico a médio prazo, sabemos também que até 10% desses pacientes tem DAC não calcificada, com cerca de 2% deles apresentando lesões > 50% em uma coorte com 1.753 pacientes.^68 , 69^

De maneira similar, pacientes em prevenção secundária, em que o risco cardiovascular já é considerado muito alto, também não são candidatos ao exame de EC.

#### 2.1.1.5. Distribuição

Estudos têm demonstrado que, de maneira análoga à cineangiocoronariografia e à angio-TC de coronárias, em que pode-se calcular o escore de segmentos envolvidos ( *Segment Involvement Score* [SIS]),^70^ a localização e a distribuição das placas calcificadas no EC têm implicação prognóstica. Assim, uma concentração de carga de placa majoritariamente em tronco de coronária esquerda (especialmente se acima de 25% do total do EC) está independentemente associada a aumento de 6 a 9% na mortalidade cardiovascular, depois de ajustada para carga de placas no restante das artérias coronárias. Pacientes "uni, bi e triarteriais" de cálcio também têm uma contínua e progressiva piora na taxa de eventos cardiovasculares, conforme demonstrado no *Framingham Heart Study* , com seguimento de 7 anos.^71 , 72^ Um estudo com população do CAC Consortium mostrou que integrar a distribuição regional ao EC tradicional resultou em melhor reestratificação de risco.^73^

#### 2.1.1.6. Idade para Início da Realização do EC

Não há uma determinação padrão, devendo ser considerado os dados clínicos obtidos com a história e o exame físico do paciente.

Um interessante estudo recente utilizando dados do CAC Consortium, com 22.346 pacientes com idade entre 30 e 50 anos, buscou determinar a idade ideal em que iniciar a pesquisa de aterosclerose subclínica com o EC seria mais útil, de acordo com a presença de FR do escore de risco ASCVD. Comparando com aqueles pacientes sem FR, indivíduos com diabetes apresentavam EC positivo 6,4 anos antes e os pacientes com os demais FR tradicionais desenvolviam EC > 0 em média 3,3 a 4,3 anos antes. O modelo utilizado no estudo apontou como idade ideal para um potencial exame de EC aproximadamente 37 anos em homens e 50 anos em mulheres com DM. No outro extremo, em pacientes sem nenhum FR, a idade ideal seria de 42 anos nos homens e 58 anos nas mulheres.^74^

#### 2.1.1.7. Periodicidade

Para pacientes com EC positivo (> 0), a recomendação geral é de não repetir o exame, principalmente se já houve reestratificação do risco cardiovascular no exame em questão. Deve-se lembrar que há uma variabilidade *inter-scans* de 15%^56^ e progressão natural da aterosclerose de 15 a 20% ao ano^75^ que também pode sofrer interferência com o tratamento com estatinas^76^ e com atividade física regular,^77^ já que ambas as situações levam a um aumento do componente calcificado das placas de ateroma. Além disso, nenhum algoritmo clínico utiliza a progressão do EC para definição do tratamento, uma vez que tal informação não é claramente superior ao EC basal como indicador de prognóstico.

Quando o EC é zero, reporta-se que 20 a 25% dos pacientes convertem para EC positivo (em geral, valores baixos) em um intervalo de 4 a 5 anos. Um estudo recente mostrou que, em pacientes de baixo risco (< 5 % do escore ASCVD), o intervalo de conversão de um EC zero foi de 5 a 7 anos. Em pacientes com risco baixo a moderado (5 a 10% do estimado pelo escore ASCVD), o intervalo de conversão foi de 3 a 5 anos e, em pacientes de alto risco e com diabetes, o intervalo foi de 3 anos. Então, de maneira geral, parece ser de 3 a 5 anos o intervalo recomendado para se repetir um EC zero.^78 , 79^

Pacientes com dois EC zero têm o melhor perfil de sobrevida livre de eventos (1,4% em 10 anos).^28^

#### 2.1.1.8. O Poder do EC Zero

Ao longo dos anos, após a sedimentação do conceito da carga de aterosclerose como o principal marcador de risco cardiovascular, o foco de estudo passou a ser o efeito protetor que a ausência de aterosclerose calcificada confere, já que, desde o início, chamava atenção a baixíssima taxa de eventos naqueles pacientes portadores de um EC zero (aproximadamente 0,1%/ano). Essa população representa aproximadamente 1/3 dos pacientes, mesmo em cenários em que a prevalência esperada de aterosclerose seria maior, como nos pacientes com dor torácica e diabéticos.^69 , 80^

Uma metanálise publicada com 29.312 pacientes com EC zero em 13 estudos evidenciou taxa de eventos de 0,47% em 4 anos nessa população.^81^

Um trabalho recente reforçou que um EC zero tem papel de fator de risco "negativo" com a ausência de aterosclerose calcificada detectável ao escore de Agatston superando os FR clínicos para predição de mortalidade. Nesse estudo com 44.052 pacientes, os indivíduos sem FR tradicionais, mas com EC > 400 tiveram risco cardiovascular bem maior que pacientes com três ou mais FR, porém com EC zero (taxa de eventos por 1.000 pacientes de 16,89 x 2,72).^82^

Na população do CAC Consortium, que é o estudo com maior número de pacientes disponível na literatura (66.363 pacientes, com idade média de 54 anos e com 33% de mulheres), 45% tinham EC zero (idade média de 45 anos) e apresentaram baixos índices de eventos cardiovasculares maiores (0,32 a 0,43 por 1.000 pessoas/ano). Inclusive, apesar de ainda raro, o câncer foi a causa predominante de morte nessa população.^83^ Esse conceito consolidou-se de tal maneira que, de forma inédita, o consenso americano de dislipidemia de 2018 contemplou a possibilidade de reclassificar para menos o risco cardiovascular daqueles portadores de EC zero, inclusive discutindo-se a retirada de medicação hipolipemiante (com exceção nos casos de pacientes tabagistas, diabéticos, com HF e antecedente familiar positivo para DAC precoce).^60^

#### 2.1.1.9. EC Muito Elevado (Acima de 1.000 Unidades Agatston)

A população com expressiva carga de aterosclerose calcificada também vem sendo estudada com mais atenção nos últimos anos.

Recentemente, uma coorte com pacientes portadores de EC > 1.000 evidenciou que o risco de eventos cardiovasculares nessa população suplanta até mesmo o de pacientes em prevenção secundária e com perfil lipídico fora da meta, como os que foram avaliados no estudo FOURIER ( *Further Cardiac Outcomes Research With PCSK9 Inhibition in Subjects with Elevated Risk* ).^84^ De maneira semelhante, um estudo com pacientes do CAC Consortium encontrou resultados parecidos,^85^ levando a proposições para um tratamento mais agressivo e um melhor custo/benefício na utilização de provas de isquemia nesse subgrupo de pacientes.

#### 2.1.1.10. Populações Especiais

#### 2.1.1.10.1. Diabetes Melito (DM)

Mesmo na população de indivíduos com DM, que teoricamente seriam de alto risco, o risco cardiovascular é heterogêneo. Essa população pode se beneficiar de individualização através do uso do EC.^65 , 66 , 80^ Um estudo de Raggi et al. com 10.377 pacientes, sendo 903 com DM, mostrou que a sobrevida em seguimento de 5 anos foi igual em pacientes com e sem DM quando o EC era 0.^26^ No estudo PREDICT ( *Prospective Evaluation of Diabetic Ischemic Disease by Computed Tomography* ), com 589 pacientes com DM e sem DAC, foi evidenciado que quanto maior o EC, maior o risco de desfechos cardiovasculares desfavoráveis.^86^ Seu uso é previsto pela Sociedade Americana de Diabetes nas recomendações de 2021, a partir dos 40 anos.^63^

#### 2.1.1.10.2. Antecedente Familiar de Doença Coronária Precoce

Pacientes com antecedente familiar positivo para DAC precoce (evento em parente de primeiro grau antes dos 55 anos em homens ou antes dos 65 anos em mulheres) apresentam maior risco cardiovascular e não costumam ser contemplados nos principais escores clínicos.

No estudo CARDIA ( *Coronary Risk Development in Young Adults* ), com pacientes de 32 a 46 anos, observou-se que CAC > 0 não é incomum, especialmente em vigência de algum FR.^87^ Adicionalmente, um estudo de Miedema et al. mostrou que o EC pode ter valor prognóstico em pacientes jovens de baixo risco (< 40 anos).^64^

#### 2.1.1.10.3. Pacientes Jovens

Outro cenário em que a utilização do EC pode ser considerada é em pacientes jovens (especialmente abaixo de 40 anos), supostamente de baixo risco cardiovascular por escores clínicos tradicionais. Como a idade tem um peso grande nesses escores, a estimativa do risco de pacientes jovens pode ficar subestimada, abaixo do limiar de recomendação do uso de estatina. Dois estudos prospectivos em pacientes jovens (CARDIA^87^ e PACC^88^), com médias de idade de 40,3 e 42,9 anos, respectivamente, evidenciaram a associação de calcificações coronarianas com um aumento de 3 a 12 vezes do risco de eventos coronarianos comparados aos pacientes com EC zero.

#### 2.1.1.10.4. Hipercolesterolemia Familiar (HF)

Mesmo em populações com risco basal elevado, como os portadores de HF, o EC parece ajudar a discernir melhor o risco cardiovascular. Miname et al., em estudo com 206 pacientes com HF com diagnóstico molecular comprovado e em uso de estatinas, com média de lipoproteína de baixa densidade (LDL) residual de 150 ± 56 mg/dL, mostrou que um EC Zero associou-se a baixo risco de eventos cardiovasculares maiores em seguimento mediano de 4 anos.^67^ Os eventos ocorreram apenas naqueles com EC > 0 (incidência respectivamente de 2,6% e 4,4% por ano naqueles com EC 1-100 e > 100). Mais recentemente, Gallo et al. encontraram resultados similares em 1.624 portadores de HF com terapia hipolipemiante (LDL-C sob tratamento 170 mg/dL) com estatinas e/ou ezetimiba seguidos por uma mediana de 2,7 anos com taxas de eventos de 0,47%, 2,1% e 14,2%, respectivamente para EC = 0, 1-100 e > 100 durante o seguimento.^89^ De forma semelhante, o estudo de Sandesara et al. com pacientes da coorte do MESA que apresentavam LDL > 190 mg/dL (LDL-C médio = 215 ± 27 mg/dL) observou que indivíduos com EC zero tinham menor número de eventos (incidência absoluta anual de eventos de 0,4%) do que aqueles com EC > 100 (2% de eventos por ano), após 14 anos de seguimento.^90^

#### 2.1.1.11. Comparação do Uso de EC com a Angiotomografia de Coronárias na Estratificação de Risco de Eventos Cardiovasculares

Com o advento de tomógrafos com novas tecnologias, a dose de radiação dos exames diminuiu de maneira substancial, chegando a 78% de redução no estudo Protection VI em relação ao estudo original,^91^ levando a proposições de se extrapolar o uso da angio-TC de coronárias para populações assintomáticas. Isso objetiva uma melhor reestratificação de risco cardiovascular, uma vez que placas não calcificadas passariam a ser visibilizadas, além de pequenas placas calcificadas não detectadas no EC, em que a resolução espacial proposta no escore de Agatston (3 mm) é bem inferior à da angio-TC de coronárias (0,5-0,625 mm). Porém, apesar de um limiar menor para detecção de aterosclerose, os resultados dos estudos são controversos, ora neutros, ora favoráveis à nova estratégia,^92 , 93^ não mostrando superioridade evidente na estratificação de risco na população assintomática de uma maneira geral. O estudo de Senoner et al., com 6.439 pacientes, mostrou que, naqueles com EC zero, mesmo quando ocorre o achado de apenas placas não calcificadas, a taxa de eventos continua sendo baixa.^94 , 95^

#### 2.1.1.12. Uso em Diretrizes Clínicas

O EC apresenta indicação IIA na Diretriz Americana de Manejo do Colesterol do Sangue para pacientes de risco cardiovascular limítrofe e intermediário (risco calculado de morte, infarto do miocárdio e acidente vascular cerebral isquêmico pelo escore ASCVD em 10 anos de 5 a 7,5% e 7,5 a 19,9%, respectivamente), quando o manejo clínico for incerto e tiver sido incorporado no algoritmo de decisão clínica para individualização do risco cardiovascular, com replicação desse conceito na Diretriz Americana de Prevenção Primária.^60 , 96^

Na Diretriz Brasileira de Dislipidemia e Prevenção da Aterosclerose, assim como na de Prevenção Cardiovascular,^16 , 91^ seu uso é recomendado em risco moderado pelo Escore de Risco Global (5-10% em mulheres e 5-20% em homens). Segundo as diretrizes americanas, EC > 100 unidades Agatston ou > percentil 75 para idade e sexo indica terapia com estatinas para reduzir o LDL-C. O EC 1-99 favorece o uso de estatinas (principalmente em pessoas acima de 55 anos). O EC zero indica baixo risco a médio prazo, deixando-se a opção de terapia de acordo com decisão compartilhada entre médico e paciente exceto para diabéticos, fumantes e portadores de história familiar de doença coronária precoce, em que a estatina é preconizada.^18 , 54 , 60 , 96^

#### 2.1.1.13. Pontos Pendentes e Perspectivas

Análises de custo-efetividade ainda não apresentaram uma resposta definitiva. Para qualquer exame, por si só, elas são difíceis de se realizar, uma vez que muitas variáveis estão envolvidas e mudam conforme o local. Podemos citar, entre outras, os custos do exame e do tratamento, a aderência do paciente e a intenção de pagar por isso. Uma análise no cenário americano apontou o EC como custo-efetivo em pacientes do sexo masculino, com risco intermediário de acordo com escores de risco clínicos.^97^

Mais estudos são necessários para determinar se a estratificação de risco baseada no EC melhora desfechos clínicos, apesar de sabermos que não são simples de serem realizados e até mesmo que métodos muito mais antigos e utilizados na cardiologia até hoje não trouxeram tal informação.

A medida visual da calcificação coronariana ou mesmo pelo escore de Agatston em exames de TC de tórax não gateados parece ter boa correlação com os valores obtidos em exames dedicados, e sua realização é recomendada pela Society of Cardiovascular Computed Tomography (SCCT), havendo uma classificação dedicada para tal fim: o *Coronary Artery Calcium Data and Reporting System* (CAC-DRS).^98^

Por fim, a utilização crescente da inteligência artificial vem agregando rapidez, possibilitando a leitura de exames em segundos e integrando a informação do EC com outros dados obtidos concomitantemente, como a quantificação da gordura epicárdica a fim de aumentar o poder de predição de eventos (" *Radiomics* ").^99^

#### 2.1.2. Papel do Escore de Cálcio Coronário na reestratificação do Risco Definido pelos Escores Clínicos Tradicionais

Conforme mencionado previamente neste documento, diversos escores estão disponíveis para a estratificação do risco de eventos cardiovasculares. Embora haja variações entre as sociedades de Cardiologia e grupos de prevenção à aterosclerose, os indivíduos costumam ser classificados nas seguintes categorias de acordo com a intensidade dos FR:

1. Baixo risco: < 5% de risco absoluto de eventos em 10 anos;
2. Risco intermediário: homens com risco absoluto ≥ 5% a ≤ 20% e mulheres com risco ≥ 5% a ≤ 10% de eventos em 10 anos; e
3. Alto risco: homens com risco absoluto > 20% e mulheres com risco > 10% em 10 anos.

Recentemente algumas sociedades internacionais introduziram um grupo de risco limítrofe: 5% a < 7,5% de risco absoluto de eventos em 10 anos.^60^

Apesar de sua grande utilidade, esses escores têm limitações, principalmente na predição de eventos cardiovasculares na categoria de risco intermediário. Nesse contexto, o estudo da aterosclerose subclínica por meio do EC pode trazer informações adicionais para uma melhor estratificação de risco individual.^100^

O emblemático estudo MESA avaliou o impacto da determinação do EC na predição dos eventos coronários em homens e mulheres de diversas etnias nos Estados Unidos seguidos por cerca de 4 anos.^54^ Em comparação àqueles pacientes sem calcificação coronária, o risco de morte ou IAM, ajustado para os demais fatores de risco de doença coronária aumentou 7,7 vezes para os indivíduos com EC entre 101 e 300 unidades Agatston e 9,7 vezes para aqueles com escores de cálcio > 300 unidades Agatston (p < 0,001 para ambas as comparações). Apesar da diferença na prevalência da calcificação coronária entre os diferentes grupos étnicos, o EC acrescentou capacidade prognóstica aos FR tradicionais de maneira similar entre esses grupos. Em subanálise do estudo MESA,^101^ após um seguimento de 5,8 anos, além de aprimorar a discriminação (curva ROC), o EC melhorou significativamente os índices de reclassificação de risco. O impacto foi maior naqueles indivíduos considerados previamente como de risco intermediário pelo ERF: 16% foram reclassificados como alto risco enquanto 39% foram reclassificados como baixo risco ( *NRI* : 0,55; IC95%: 0,41-0,69; p < 0,001).

Em outro estudo prospectivo, a adição do EC aos fatores de risco tradicionais melhorou a predição de eventos cardiovasculares em relação ao ERF, proporcionando um aumento da área sob a curva (AUC) ROC de 0,63 para 0,68 (p < 0,001). Entretanto, o EC não modificou de forma significativa a predição dos indivíduos na categoria de risco < 10% do ERF.^28^ Uma exceção a essa categoria de menor risco seriam os indivíduos com história familiar positiva para DAC precoce, cuja associação com EC alto (> percentil 80) identificou um grupo de maior risco e que potencialmente se beneficiaria de intensificação da terapia hipolipemiante, de acordo com subanálise do estudo *St. Francis Heart* .^102^ Já na categoria de alto risco, o EC baixo não reclassifica adequadamente os indivíduos em um risco mais baixo e, portanto, não deve indicar a redução da terapêutica voltada para esses pacientes.^28 , 101 , 103^ Desse modo, os pacientes classificados como de risco intermediário pelo ERF são aqueles que mais se beneficiam da adição do EC, pela maior possibilidade de reclassificação correta, o que poderia levar a uma modificação das metas de prevenção primária.

#### 2.1.2.1. Comparação do Escore de Cálcio com Outros Métodos de Estratificação de Risco Cardiovascular

Nos últimos anos, foram publicados diversos estudos que compararam a utilização do EC coronário a outras ferramentas para detecção de aterosclerose subclínica e avaliação prognóstica, entre elas o índice tornozelo-braquial (ITB), a espessura médio-intimal ( *intima-media thickness* [IMT]) carotídea, a micro ou macroalbuminúria e a proteína C-reativa (PCR) de alta sensibilidade.^31 , 104^

Uma subanálise do estudo MESA demonstrou que a IMT se associa com a presença e a progressão da calcificação coronária.^105^ Em outro subestudo, foi demonstrado que o ERF isoladamente apresentou uma AUC ROC de 0,77 para a predição de eventos cardiovasculares, o ERF associado à IMT > 1,0 mm apresentou área de 0,78 (1,3 mais eventos cardiovasculares) e o ERF associado ao CAC > 0 teve área de 0,81 (2,1 vezes mais eventos cardiovasculares).^106^ Já Brook et al. compararam acurácia de EC, IMT, PCR e área de placa em carótida para a identificação de DAC obstrutiva, definida como estenose luminal de pelo menos 50% na angio-TC de coronárias, verificando que a AUC ROC do EC e da área de placa carotídea eram similares para predizer a presença de aterosclerose coronária significativa e superiores à PCR e à IMT.^107^

No estudo MESA, Yeboah et al. compararam diretamente os principais marcadores de risco quanto à acurácia na predição de eventos cardiovasculares.^31^ Foram analisados 1.330 pacientes de risco intermediário pelo escore de Framingham, não diabéticos, em um seguimento médio de 7,6 anos. Foram avaliados os seguintes marcadores de risco para predição de eventos cardiovasculares: EC, IMT da carótida, ITB, dilatação fluxo-mediada da artéria braquial (DILA), PCR e história familiar de DAC precoce. Adicionalmente, procurou-se avaliar a correta reclassificação dos pacientes de acordo com os resultados desses marcadores, segundo a ferramenta estatística NRI. Após o seguimento, 94 pacientes (7,1%) tiveram eventos cardíacos (definidos como infarto ou angina seguido de revascularização, morte súbita abortada e óbito por DAC) e 123 (9,2%) sofreram eventos cardiovasculares (DAC ou acidente vascular cerebral [AVC]). O estudo observou que EC, ITB, PCR e história familiar foram preditores independentes de risco de DAC, enquanto IMT e DILA não demonstraram capacidade de predição independente de risco de DAC. Além disso, o EC foi o marcador que mais acrescentou à capacidade do escore de Framingham em predizer eventos cardiovasculares, mostrada pela área sob a curva ROC (0,623 vs. 0,784), e foi o marcador que melhor reestratificou os pacientes em maior ou menor risco – 65% dos pacientes foram corretamente reclassificados em risco mais alto ou mais baixo, comparado a 16% da história familiar, 10% do IMT e 8% da PCR.

Portanto, o EC se mostra como a ferramenta de detecção mais acurada de aterosclerose subclínica para o refinamento da estratificação de risco em pacientes assintomáticos.

#### 2.1.3. Uso do Escore de Cálcio no Suporte na Decisão de Terapia Farmacológica

O uso do EC coronário atualmente é considerado mais adequado para a estratificação adicional dos indivíduos assintomáticos com risco cardiovascular intermediário. Com base nessa reestratificação de risco, a informação do EC poderia ajudar na decisão compartilhada de não iniciar/suspender um tratamento antiaterosclerótico mais agressivo com hipolipemiantes (nos pacientes reestratificados para baixo risco) ou antiplaquetários^108^ ou iniciar/intensificar o tratamento antiaterosclerótico com mudanças de hábitos de vida, hipolipemiantes e antiplaquetários (nos pacientes reestratificados para alto risco). Ainda não foram publicados ensaios clínicos randomizados comparando essa estratégia guiada pelo EC *versus* a estratégia guiada pela estratificação habitual de risco, o que suscita críticas ao emprego do método. No entanto, devido à extensa evidência científica do EC como marcador independente de risco cardiovascular e ao reconhecido papel das estatinas na redução desse risco, essa conduta é considerada plausível e o uso do EC é sugerido em diversas diretrizes de prevenção e tratamento da aterosclerose, quando há dúvida quanto à indicação de estatinas em determinadas categorias de risco.^19 , 60 , 96^

Aplicando as Diretrizes do American College of Cardiology/ American Heart Association (ACC/AHA) de 2013 para tratamento de dislipidemia e prevenção de aterosclerose (que já recomendavam o uso de estatina quando o risco absoluto de eventos cardiovasculares [morte, infarto do miocárdio ou AVC] era ≥ 7,5% em 10 anos e consideravam o seu uso quando o risco era de 5% a < 7,5%) sobre a população do estudo MESA, Nasir et al. constataram que aproximadamente 57% dos indivíduos não diabéticos no grupo de risco limítrofe (risco de 5% a < 7,5% pelo escore clínico) tiveram EC = 0 e uma taxa de eventos muito baixa, de 1,5% em 10 anos (sem recomendação de estatinas), em contraste com aqueles com CAC (EC > 0), que tiveram uma taxa de eventos de 7,4% (podendo ser considerado o uso de estatinas).^61^ De forma semelhante, nos indivíduos do grupo intermediário (risco de 7,5% a 20% pelo escore clínico), a taxa de eventos nos pacientes com EC = 0 foi de 4,6% em 10 anos (portanto, não teriam mais a recomendação de estatinas), enquanto, naqueles com CAC (EC > 0), a taxa foi de 10,4% (reforçando a indicação de estatinas). Portanto, de acordo com essa análise, mais de 50% dos indivíduos nos grupos limítrofes e intermediário poderiam ser reclassificados como de baixo risco, sendo poupados do uso crônico de estatinas com seus custos e possíveis efeitos colaterais associados. Em outras palavras, o EC seria capaz de reduzir de forma significativa o número necessário para tratar (NNT) para evitar um evento cardiovascular nesses grupos de indivíduos, otimizando os recursos com a utilização de estatinas. De forma oposta, aqueles com calcificação coronariana demonstrada pelo EC poderiam se beneficiar do tratamento antiaterosclerótico mais intensivo. Cerca de metade dos indivíduos participantes do estudo MESA JUPITER^109^ tinham EC igual a zero, apesar de PCR elevada, e apresentavam uma baixa taxa de eventos ao longo do seguimento de 5 anos, gerando um NNT de 549 para o tratamento com rosuvastatina 20 mg prevenir um evento coronário. No entanto, a maioria dos eventos (74%) ocorreu no subgrupo de indivíduos com EC > 100 unidades Agatston. Considerando o tratamento apenas desse subgrupo, o NNT seria muito mais favorável: de apenas 24 para prevenir um evento coronário.

Em estudo retrospectivo com militares do centro médico Walter Reed do exército americano,^110^ os autores avaliaram o impacto do uso de estatinas estratificado pelo EC em 13.644 participantes, que foram seguidos por uma mediana de tempo de 9,4 anos, após análise ajustada para comorbidades. A terapia com estatinas associou-se a menor risco de eventos cardiovasculares maiores (infarto agudo do miocárdio [IAM], AVC e morte cardiovascular) apenas nos indivíduos com EC > 0 ( *subhazard ratio* ajustada de 0,76; IC95% de 0,60 a 0,95; p = 0,015), não sendo observada essa associação nos participantes sem calcificações coronarianas ( *subhazard ratio* ajustada de 1,00; IC95% de 0,79 a 1,27; p = 0,99). Além disso, o impacto do uso de estatinas sobre a redução dos eventos foi relacionado ao grau de calcificações coronarianas, sendo maior com EC mais altos. Porém, sendo um estudo retrospectivo e não um ensaio clínico randomizado, também possui muitas limitações e não pode ser considerada uma evidência definitiva do uso do EC como guia da terapia hipolipemiante.

O estudo holandês ROBINSCA ( *Risk or Benefit in Screening for Cardiovascular Disease* )^97^ é o primeiro estudo randomizado a comparar a estratégia de estratificação de risco e tratamento guiada pelo EC *versus* a estratégia guiada pela estratificação clínica. O estudo avaliou o impacto na redução de eventos coronarianos de duas estratégias de *screening* como guia de tratamento antiaterosclerótico: avaliação do escore de cálcio coronário seguido de tratamento segundo as diretrizes locais para os indivíduos com EC > 100 unidades Agatston *versus* avaliação com um escore clínico ( *Systemic Coronary Risk Evaluation –* SCORE) seguida de tratamento para os sujeitos com escore > 10%. A utilização do EC reduziu significativamente o número de indivíduos indicados para tratamento preventivo em comparação ao SCORE (redução relativa mulheres: 37,2%; homens: 28,8%).

A Tabela 2 traz os principais cenários clínicos relacionados ao emprego do escore de cálcio coronariano.

**Tabela 2:** Emprego do escore de cálcio coronariano conforme diferentes cenários clínicos

Indicações	Classe de recomendação	Nível de evidência
Reestratificação de risco em pacientes assintomáticos com escore de risco clínico intermediário^2 , 8 , 16 , 18 , 21 , 24 , 25 , 32 , 33 , 111 – 114^	I	A
Reestratificação de risco em pacientes assintomáticos com escore de risco clínico intermediário para orientação quanto à prevenção primária medicamentosa^2 , 8 , 16 , 18 , 21 , 24 , 25 , 32 , 33 , 111 – 114^	IIa	B
Reestratificação de risco em pacientes assintomáticos com diabetes melito ou síndrome metabólica e com escore de risco clínico intermediário^2 , 16 , 26 , 86^	I	B
Reestratificação de risco em pacientes assintomáticos com escore de risco clínico baixo e com história familiar de DAC precoce^2 , 16 , 25 , 64 , 87^	IIa	B
Reestratificação de risco em pacientes assintomáticos portadores de hipercolesterolemia familiar heterozigótica^67 , 115^	I	B
Triagem para pesquisa de isquemia miocárdica em pacientes assintomáticos com diabetes melito^2 , 116^	IIa	B
Realização do escore de cálcio coronariano para melhor estratificação de risco em pacientes sem DAC conhecida submetidos a testes de isquemia com resultados negativos^37 , 38 , 117 , 118^	IIa	B
Para afastar estenose coronária significativa em pacientes sintomáticos com suspeita de angina estável ou síndrome coronária aguda^68 , 119^	III	B
Uso em pacientes com DAC obstrutiva conhecida	III	C

DAC: doença arterial coronariana.

### 2.2. Angiotomografia de Coronárias na Suspeita de angina estável sem DAC Conhecida

#### 2.2.1. Como Opção de Primeira Escolha na Avaliação de Dor Torácica Não Aguda

Diversas sociedades publicaram documentos recentes sobre a melhor abordagem na investigação de dor torácica, particularmente em casos em que há suspeita de origem cardíaca e/ou DAC.^8 , 21^ A recomendação atual das sociedades americanas, europeias e brasileira é de que seja realizada uma avaliação de probabilidade pré-teste para a presença de DAC obstrutiva e que a recomendação sobre a investigação subsequente seja feita de acordo com essa probabilidade.

Existem vários escores de probabilidade pré-teste, e a concordância entre eles nem sempre é considerável.

Apesar das diferenças, as diretrizes internacionais consideram que a definição de probabilidade pré-teste deve levar em conta os sintomas, o sexo e a idade dos pacientes. Esta Diretriz recomenda que, em pacientes com probabilidade pré-teste acima de 10%,^111^ na ausência de outro diagnóstico etiológico claro, a presença de DAC obstrutiva deve ser considerada e a investigação subsequente deve ser considerada.

A forma de investigação da presença de DAC nesses casos depende também da presença de DAC prévia. Os casos de DAC obstrutiva prévia documentada são abordados em outras partes do presente documento, e, nesta sessão, discutiremos a abordagem em indivíduos sem DAC obstrutiva prévia conhecida. Nesses casos, as diretrizes consideram que métodos de provocação de isquemia como teste ergométrico, ecocardiograma de estresse, medicina nuclear com estresse ou ressonância com estresse podem ser considerados da mesma forma que a investigação pode ser realizada com a avaliação anatômica não invasiva através da angiotomografia de artérias coronárias. Para a decisão individualizada entre os métodos diagnósticos, deve-se sempre considerar a experiência local de quem trabalha com os métodos, a acessibilidade e a disponibilidade e o custo do método.

Para uma avaliação completa da performance da angio-TC de artérias coronárias na investigação de DAC obstrutiva, deve-se considerar:

1. A acurácia diagnóstica da angio-TC coronariana para a identificação de DAC obstrutiva em diferentes populações;
2. O valor prognóstico dos achados da angio-TC coronariana;
3. Os estudos de eficácia comparada da angio-TC coronariana com outros métodos de investigação para DAC; e
4. A custo-efetividade das diversas estratégias de investigação de DAC.

#### 2.2.1.1. Acurácia Diagnóstica

Desde a publicação do estudo Core-64 em 2008, diversos estudos demonstraram a alta acurácia diagnóstica da angio-TC coronariana quando comparada com a angiografia coronária invasiva.^112^ Mais recentemente, uma metanálise com mais de 5 mil indivíduos demonstrou que a angio-TC tem sensibilidade de aproximadamente 95% e especificidade de aproximadamente 79%.^113^

#### 2.2.1.2. Valor Prognóstico

Diversos estudos demonstraram o valor prognóstico dos achados de angio-TC coronariana.^120 – 122^ Esses estudos demonstraram que a presença de placas obstrutivas ou não obstrutivas é preditora de eventos, assim como a presença de características de maior risco nas placas detectadas pela tomografia e pela extensão da DAC definida pelo número de segmentos com placas ateroscleróticas na angio-TC.

#### 2.2.1.2.1. Estudos de Eficácia Comparada da Angio-TC Coronariana com Outros Métodos Diagnósticos para Investigação de DAC Obstrutiva

Ao menos dois estudos randomizados de grande porte avaliaram o uso da angio-TC coronariana na investigação de DAC não aguda. No estudo PROMISE (já abordado previamente),^23^ a avaliação por angio-TC coronariana não foi superior à avaliação por métodos de provocação de isquemia. Já no estudo SCOT-HEART, aproximadamente 4 mil participantes foram randomizados para a avaliação usual com teste ergométrico ou a inclusão adicional de angio-TC coronariana à investigação habitual. No estudo SCOT-HEART, foi demonstrada uma redução de aproximadamente 40% na taxa de infartos do miocárdio durante o seguimento de até 5 anos.^38^ Nesse estudo, não houve aumento persistente na utilização de angiografia invasiva ou revascularização no grupo TC coronária.

Em uma metanálise de quatro estudos com esse desenho, bem como um grande estudo retrospectivo na Dinamarca, foi demonstrado um achado próximo do resultado identificado no SCOT-HEART, com uma redução significativa de infarto, porém sem diferença em mortalidade com o uso da angio-TC coronária.^123 , 124^

Em resumo, esses estudos sugerem que, para a população de pacientes sem DAC prévia, a investigação inicial com angio-TC coronária resulta em menor taxa de infartos subsequente. No entanto, nenhum dos estudos avaliou de forma robusta subgrupos para identificar em quais populações existe maior ou menor benefício dessa estratégia.

#### 2.2.1.2.2. Estudos de Custo-efetividade

Até o presente momento, dados de custo-efetividade para o uso da angio-TC coronária na realidade brasileira são limitados. Ainda assim, um estudo brasileiro recente concluiu que a inclusão da angio-TC ao rol do arsenal diagnóstico do Sistema Único de Saúde (SUS) representaria uma estratégia custo-efetiva na maioria dos cenários avaliados.^125^ Apesar de estudos prévios sugerirem que a estratégia inicial com angio-TC coronária seguida de investigação com métodos de provocação de isquemia em casos de TC com alterações seja sugerida como a mais custo-eficaz para realidades dos Estados Unidos e da Holanda, não é possível afirmar que o mesmo ocorreria quando modelados os dados para a realidade brasileira.^125 , 126^ No entanto, dentro dos dados atualmente disponíveis, a angio-TC coronária parece ao menos tão custo-efetiva quanto outras estratégias utilizadas na investigação de DAC não aguda.

#### 2.2.2. Em Pacientes de Baixo Risco com Testes Funcionais Positivos

A angio-TC coronária pode ser solicitada na investigação inicial dos pacientes com suspeita de DAC ou naqueles pacientes com testes de isquemia previamente realizados. Nesse último cenário, os pacientes com testes de isquemia prévios inconclusivos, conflitantes ou com resultados discordantes da clínica apresentada podem se beneficiar da correta indicação desse exame.^2 , 3 , 8 , 25 , 127 – 129^

Em um estudo realizado por Abidov et al., 199 pacientes com testes de isquemia prévios realizaram angio-TC coronária para avaliação de DAC e foram seguidos por pelo menos 2 anos.^127^ Nos pacientes com testes de isquemia positivos, a angio-TC coronária demonstrou estenose > 50% somente em 19% dos pacientes. Dos 199 pacientes, 63% tinham indicação de cinecoronariografia antes da angio-TC coronária. Depois da angio-TC coronária, a cinecoronariografia foi realizada em apenas em 16% dos casos durante o seguimento de 2 anos. Tais achados evidenciam o importante valor diagnóstico e prognóstico nessa população com testes de isquemia prévios.

Outra indicação validada da angio-TC coronária é como alternativa à cinecoronariografia invasiva em pacientes com suspeita de DAC estável e probabilidade pré-teste intermediária de DAC, tanto por sintomas clínicos quanto por resultados alterados de outros exames cardíacos, como testes de isquemia.

No estudo CONSERVE ( *Coronary Computed Tomographic Angiography for Selective Cardiac Catheterization* ),^128^ multicêntrico e internacional, Chang randomizou 1.631 pacientes para cinecoronariografia invasiva ou angio-TC coronária (estratégia seletiva), com seguimento de 1 ano, e observou o mesmo número de eventos cardiovasculares (4,6%) nos dois grupos. No grupo da estratégia seletiva, apenas 23% dos pacientes realizaram cinecoronariografia no seguimento (redução de 77%), com diminuição no número de cinecoronariografias sem estenoses obstrutivas (24,6% vs. 61,1%), redução no número de revascularizações (13% vs. 18%) e redução nos custos totais em 57% a favor do grupo da angio-TC coronária.

No estudo DISCHARGE ( *Diagnostic Imaging Strategies for Patients with Stable Chest Pain and Intermediate Risk of Coronary Artery Disease* ),^129^ Maurovich-Horvat et al. randomizaram 3.561 pacientes com dor torácica estável para cinecoronariografia invasiva ou CTA em 26 centros europeus com seguimento de 3,5 anos. O grupo da angio-TC coronária encontrou o mesmo número de pacientes com DAC obstrutiva que o grupo da cinecoronariografia (25,7% vs. 25,7%) e o mesmo número de eventos cardiovasculares no seguimento (2,1% vs. 3,0%), mesmo realizando cinecoronariografia em apenas 22% dos pacientes (redução de 78%). Entretanto, o grupo submetido a angio-TC coronária demonstrou redução significativa nas complicações maiores relacionadas aos procedimentos (0,5% vs. 1,9%), diminuição no número de cinecoronariografias sem estenoses obstrutivas (27,5% vs. 74,3%) e redução no número de revascularizações (14,2% vs. 18,0%).^129^

A Tabela 3 apresenta os principais cenários clínicos envolvidos na utilização da angio-TC coronária na pesquisa da DAC estável.

**Tabela 3:** Angiotomografia das artérias coronárias na avaliação da DAC estável

Indicações	Classe de recomendação	Nível de evidência
Avaliação de pacientes sintomáticos com suspeita de DAC estável com probabilidade pré-teste baixa ou intermediária^2 , 3 , 8 , 23 , 25 , 112 , 130 – 132^	I	A
Adequada como opção inicial para avaliação de pacientes sintomáticos com suspeita de DAC estável com probabilidade pré-teste baixa ou intermediária e sem DAC conhecida^3 , 8 , 23 , 37 , 38 , 133^	I	A
Avaliação de pacientes com suspeita de DAC estável com testes de isquemia prévios inconclusivos ou conflitantes^2 , 3 , 8 , 25 , 127 – 129^	I	A
Avaliação de pacientes com suspeita de DAC estável com discordância entre a clínica e os resultados de testes de isquemia prévios^2 , 3 , 8 , 25 , 127 – 129^	I	A
Alternativa na avaliação de pacientes com suspeita de DAC estável com probabilidade pré-teste intermediária e indicação de cinecoronariografia invasiva^128 , 129^	I	A
Avaliação de pacientes assintomáticos portadores de hipercolesterolemia familiar homozigótica	IIa	B
Avaliação selecionada de pacientes assintomáticos com escore de risco clínico alto^3 , 92 , 134^	IIb	C
Avaliação de pacientes com suspeita de DAC estável com probabilidade pré-teste alta^2 , 20 , 25^	III	C
Avaliação de rotina de pacientes assintomáticos com escore de risco clínico baixo ou intermediário^3 , 25 , 135^	III	B
Avaliação de pacientes assintomáticos que exercem profissões de risco (exemplo: piloto de avião) com idade maior ou igual a 40 anos e com escore de risco clínico aumentado (≥ 10%)^136 , 137^	IIa	C

DAC: doença arterial coronariana.

### 2.3. Na Pesquisa de Etiologia Isquêmica de Insuficiência Cardíaca

Em pacientes com disfunção ventricular de origem indeterminada, a exclusão de DAC significativa é necessária, sobretudo naqueles com FR e/ou sintomas sugestivos de insuficiência coronária.^138^ A angiografia invasiva é o método diagnóstico de referência para a detecção de obstrução coronária significativa nesse contexto clínico,^139 , 140^ porém a angio-TC coronariana tem se mostrado bastante útil nesse cenário, explorando a sua alta sensibilidade e valor preditivo negativo global para a detecção de estenose luminal.^112 , 132^

Em pacientes com IC, estudos demonstraram que a angio-TC de coronárias apresenta sensibilidade, especificidade e valores preditivos positivo e negativo de 73–98%, 99–100%, 92–99% e 97–100% para detecção de DAC obstrutiva, respectivamente.^141 – 143^ Em uma análise com 96 pacientes com prevalência de DAC de 46%, a angio-TC coronária identificou corretamente 90% dos pacientes com etiologia isquêmica e 97% dos pacientes com coronárias sem lesões obstrutivas.^142^ Já Chow et al., em um estudo randomizado, multicêntrico e internacional, avaliaram a custo-efetividade da angiotomografia *versus* o cateterismo cardíaco na avaliação de 246 pacientes com IC de início recente, não encontrando diferença estatisticamente significativa entre os grupos no que se refere aos desfechos clínicos e custos entre as duas estratégias.^144^

Portanto, a utilização da angio-TC das artérias coronárias para o auxílio na diferenciação entre cardiopatia isquêmica e não isquêmica com IC de etiologia desconhecida é considerada apropriada, sobretudo quando a probabilidade pré-teste de DAC obstrutiva é baixa a intermediária ( Tabela 4 ).^25 , 138 , 145^

**Tabela 4:** Utilização da angiotomografia das artérias coronárias no auxílio à avaliação etiológica de insuficiência cardíaca

Indicações	Classe de recomendação	Nível de evidência
Avaliação de pacientes com insuficiência cardíaca, como auxílio na distinção entre cardiomiopatias isquêmicas e não isquêmicas^25 , 101 , 145^	I	B

### 2.4. Angiotomografia de Coronárias na Suspeita de Angina Estável com DAC Conhecida

#### 2.4.1. Portadores de *Stents*

As estruturas metálicas da malha dos *stents* podem gerar artefatos de imagem na angio-TC coronária, criando dificuldades na análise do lúmen coronariano. Esses artefatos são mais proeminentes em *stents* menos calibrosos (inferiores a 2,5 a 3,0 mm), em *stents* apostos a placas densamente calcificadas e em *stents* com malha mais espessa que 100 μm.^146^ Por essa razão, estudos funcionais são preferencialmente a primeira opção em pacientes com *stents* prévios e suspeita de DAC obstrutiva.^147^ No entanto, a angio-TC coronariana não é contraindicada nesses pacientes e pode ser realizada com boa acurácia, utilizando filtros específicos de imagem e protocolos otimizados, com especial atenção ao controle da frequência cardíaca no momento da aquisição para minimizar artefatos de movimento. Uma recente metanálise que incluiu 2.656 pacientes estudados em tomógrafos com 64 colunas de detectores ou mais (4 cm ou mais de cobertura) analisou 4.131 *stents* individualmente quanto à presença de lesões potencialmente limitantes ao fluxo coronariano (estenose ≥ 50%).^148^ Esses dados sugerem que a angio-TC coronariana é acurada para a avaliação de *stents* , especialmente tendo em vista as novas gerações de tomógrafos. Finalmente, dados funcionais derivados da perfusão miocárdica sob estresse por TC podem aumentar a acurácia diagnóstica da angio-TC coronariana em pacientes com *stents* .^149^

#### 2.4.2. Revascularizados

Em uma metanálise levando em conta a avaliação de 2.482 enxertos, a sensibilidade e a especificidade da angio-TC de coronárias com equipamentos de 64 fileiras de detectores para quaisquer estenoses maiores que 50% foi de 0,98 (IC95%, 0,97-0,99) e de 0,98 (IC95%, 0,96-0,98), com AUC de 0,99. Nem a idade dos pacientes nem o intervalo de tempo entre a realização do enxerto e o exame apresentaram qualquer efeito em relação à sensibilidade ou à especificidade para detecção de estenose significativa ou oclusão. Também não houve diferença de acurácia entre enxertos arteriais e venosos.^150^ Esses resultados para avaliação dos enxertos podem potencialmente ser ainda melhores em equipamentos mais modernos.^151^ Por outro lado, o leito nativo pode ser de difícil avaliação, dada a presença de doença ateromatosa acentuada, por vezes com grande quantidade de cálcio depositado, diminuindo a especificidade do método.^152^

Dessa forma, se o interesse clínico é de avaliar a patência dos enxertos, a angio-TC é validada e apropriada.^3^ Se o interesse é a avaliação do leito nativo, esse exame pode apresentar maiores limitações, devendo ser considerado o emprego de testes funcionais. Deve ser lembrada a capacidade da angio-TC coronariana de identificar os territórios coronarianos não protegidos, bem como estimar sua extensão. Dado que um maior número de territórios coronarianos não protegidos está associado a pior prognóstico, a informação fornecida pelo exame tem grande relevância no manejo desses pacientes.^153^

A Tabela 5 traz as indicações e os cenários clínicos relacionados à utilização da angio-TC coronária em pacientes com revascularização (percutânea ou cirúrgica) prévia.

**Tabela 5:** Angiotomografia das artérias coronárias na avaliação da doença arterial coronariana em pacientes revascularizados

Indicações	Classe de recomendação	Nível de evidência
Avaliação de pacientes sintomáticos com revascularização cirúrgica prévia, principalmente se a patência dos enxertos é o objetivo primário^2 , 3 , 25 , 150 , 154 , 155^	I	A
Avaliação de pacientes assintomáticos com revascularização cirúrgica prévia há 5 ou mais anos^25 , 150 , 154 , 155^	IIb	B
Avaliação de pacientes sintomáticos com angioplastia com *stent* ( *s* ) prévia, principalmente se o diâmetro do(s) *stent* ( *s* ) é ≥ 3 mm^2 , 3 , 25 , 47 , 156 , 157^	IIa	B
Avaliação de pacientes assintomáticos com angioplastia com *stent* do tronco de coronária esquerda, principalmente se o diâmetro do stent é ≥ 3 mm^25 , 148 , 156 , 157^	IIa	B
Avaliação de pacientes assintomáticos com angioplastia com *stent* ( *s* ) prévia há 2 ou mais anos, principalmente se o diâmetro do(s) *stent* ( *s* ) é ≥ 3 mm^25 , 148 , 156 , 157^	IIb	B
Avaliação de pacientes sintomáticos com alta suspeita clínica de angina e com revascularização cirúrgica ou percutânea prévia^2 , 20 , 25^	III	C

### 2.5. Seguimento de Coronariopatas em Tratamento Clínico

A tomografia das artérias coronárias é uma forma não invasiva de avaliar as artérias que nutrem o miocárdio e que faculta a análise da luz desses vasos, de suas paredes e, mais recentemente, também possibilita o estudo das características dos ateromas que eventualmente possam comprometer aqueles vasos.^158^ Desde os períodos iniciais da aplicação clínica desse método, ele se destacava pelo seu elevado poder preditivo negativo, mostrando-se eficaz para descartar com segurança a presença de doença coronária obstrutiva.^132 , 159^ À medida, porém, em que a tecnologia e a experiência com esse exame aumentaram, houve aumento do poder preditivo positivo, que pode ser ainda mais elevado no caso de se utilizar a análise não invasiva da reserva de fluxo coronária, o que lhe dá grande potencial de uso clínico.^158 , 160^ Além das publicações que apresentam suas potenciais contribuições, surgiram trabalhos mostrando que a tomografia pode aprimorar a avaliação de pacientes com suspeita de apresentar doença coronária, e, dessa forma, dispomos hoje de dados que norteiam o uso dessa técnica nesse subgrupo de pacientes, sendo este o tema abordado nesta seção.^158 , 160^

Considera-se que, em pacientes de baixo risco, a tomografia pode auxiliar na condução dos casos, identificar pacientes nos quais há ateromatose coronária, mesmo que não calcificada, e definir a carga aterosclerótica de placas não invasivas, elemento que cresce em poder prognóstico, em especial nos casos nos quais não houve investigação prévia para confirmar o diagnóstico de doença coronária.^38 , 121^ Por outro lado, a ausência de placas de ateroma representa um importante fator de bom prognóstico, e, mesmo no estudo PROMISE, a taxa de eventos nos casos de coronárias sem lesões obstrutivas era menor do que nos casos de provas funcionais normais (razão de chance ajustada 0,38; IC95%: 0,18–0,79; p = 0,01).

Nos casos de risco intermediário e intermediário-alto, a tomografia alcança seus melhores resultados.^21 , 38^ O exame é muito eficaz para determinar a ausência ou a presença de DAC, definir se existe ou não doença significativa no tronco da coronária esquerda, fornecer a carga aterosclerótica total e a carga de placas não calcificadas e possibilitar a análise da placa.^121 , 158 , 160 , 161^ Sua sensibilidade é elevada, e a adequação para excluir a presença de doença coronária obstrutiva nas artérias epicárdicas ainda constitui parte importante de suas indicações. Da mesma forma, quando há ateromatose nas artérias do coração à tomografia, há indicação de manejo clínico intenso, pois isso implicará em melhor prognóstico e redução de eventos. Em virtude disso, a tomografia vem assumindo o papel de exame inicial para avaliar a presença ou ausência de lesões obstrutivas em pacientes com risco pré-teste intermediário e intermediário-alto na prática clínica. Alguns estudos demonstraram que, nesse cenário, o exame se mostra efetivo para o estudo da anatomia das artérias coronárias, sendo custo-efetivo em relação ao exame invasivo e apresentando a mesma taxa de eventos adversos tardios.^128^ É importante mencionar que, mesmo em casos em que não se confirma o diagnóstico de isquemia, a presença de aterosclerose é preditor isolado de eventos, mesmo nos casos em que não seja considerada significativa.^38 , 160 , 161^ A acurácia do exame é comprovada também em estudos mais recentes, e, por esses motivos, passou a ser indicado para a análise inicial de pacientes sintomáticos no Reino Unido.^160 , 162 , 163^ Por outro lado, a presença de estenoses apenas à tomografia não pode ser utilizada para indicar procedimentos de revascularização, uma vez que essa estratégia não se mostrou eficaz para reduzir eventos, mas é importante que se analise cada caso de modo individual, considerando, além da presença ou não das placas de ateroma, elementos clínicos e funcionais e a possibilidade de se controlar ou não os sintomas com o tratamento clínico.^38 , 158 , 160 , 161^ Alguns autores fazem a ressalva de que a especificidade do exame é muito inferior à sua sensibilidade, o que seria, portanto, uma limitação do exame. Se, por um lado, alguns estudos confirmam essa análise, a associação com exames funcionais pode permitir a adoção de conduta mais adequada, e a introdução na prática clínica da análise da FFR pela tomografia pode fazer com que o exame assuma papel de ainda maior destaque no manejo de pacientes com angina estável.^158 , 160^ A análise da perfusão pode ser feita pela própria tomografia, cuja eficácia foi comprovada no estudo CORE320, no qual ficou demonstrado que a associação de análise anatômica e funcional pela tomografia possibilitava conduzir com segurança pacientes com doença coronária, sem aumento de riscos maiores para os pacientes.^4^ Essa abordagem, na prática, é limitada por necessitar de duas injeções de contraste e por exigir maior dose de radiação ionizante para que seja adquirida.^160^ Já a análise da FFR pela tomografia vem despertando grande interesse e faculta, a partir de uma única aquisição, a avaliação da anatomia e dos dados funcionais.^160^ Sua utilidade foi comprovada em estudos randomizados e em recente metanálise, sendo sua maior limitação prática ainda a disponibilidade ampla e o aspecto econômico.^158 , 160 , 164^ Há desenvolvimento de protocolos que tentam estimar os resultados da FFR a partir de tecnologias de aprendizado de máquina e inteligência artificial, mas elas ainda não se encontram disponíveis para o uso clínico.

A publicação dos estudos ORBITA e ISCHEMIA exige que se faça uma análise particular do papel dos dados funcionais obtidos por qualquer tipo de tecnologia, incluindo a tomografia, e estimulam o manejo clínico inicial de grande parte de pacientes com angina estável. Destaca-se, porém, que os dados anatômicos fornecidos pela tomografia, em especial o diagnóstico ou a exclusão de lesões no tronco da coronária esquerda, são fundamentais tanto para definir a necessidade de tratamento clínico e para identificar quais casos podem se beneficiar de tratamento intervencionista. Para essa finalidade, também, é lícito especular que os dados funcionais advindos da análise da FFR pela tomografia podem ser muito úteis também para identificar quais pacientes podem se beneficiar de procedimentos de revascularização.^158 , 160 , 161 , 165^

Entre os dados fornecidos pela tomografia em pacientes com angina estável, vem ganhando força a análise das características da placa de ateroma. Trabalhos pioneiros despertaram o interesse nessas características e sabe-se hoje que subanálises dos estudos ISCHEMIA, SCOTT-HEART e de registros como o CONFIRM demonstraram que a presença de placas não calcificadas, com sinais de remodelamento positivo, heterogeneidade de atenuação, incluindo o sinal do anel de guardanapo e áreas de calcificação puntiforme implicam pior prognóstico. Tais resultados estimulam a inclusão desses dados na análise tomográfica e levaram ao desenvolvimento de escores como o CT-Leaman e o Leiden, que incorporam essas informações ao lado de dados como a localização da lesão e o grau de obstrução (> 50% ou < 50%), que ajudam a estabelecer o risco para o paciente.^160 , 161 , 166^ Destaque especial, porém, merece a carga aterosclerótica total, que, quando elevada, indica prognóstico mais grave, mesmo em pacientes que não apresentem estenoses que diminuam a luz do vaso em mais do que 50%.^121 , 158 , 160^ Mesmo em pacientes estáveis, esse índice tem valor prognóstico e foi incorporado a alguns escores que se encontram atualmente disponíveis para a prática clínica.

Recentemente, o estudo SYNTAX-III Revolution demonstrou que a tomografia pode auxiliar também para definir quais pacientes serão melhores candidatos para tratamento percutâneo ou cirúrgico, demonstrando que a inclusão da análise da FFR é fundamental para essa finalidade. Caso estudos posteriores possam confirmar esses achados, o papel da tomografia pode ser ainda maior.^160 , 167^

Finalmente, caso exista retorno de sintomas após a realização de procedimentos de revascularização, seja com o implante de *stents* ou com o tratamento cirúrgico, a tomografia também pode ser utilizada para esclarecer sintomas. Sua eficácia é comprovada para a avaliação de enxertos e mesmo de vasos nativos no caso de pacientes submetidos ao tratamento cirúrgico. Já para a análise de *stents* , os resultados são superiores no caso de endopróteses com mais de 3,0 mm de diâmetro e que não se encontrem em segmentos arteriais muito calcificados. Contudo, a tomografia pode apresentar bons resultados em casos selecionados e nos quais se obtenham imagens de qualidade.^150 , 158 , 167^

### 2.6. Angiotomografia das Artérias Coronárias na Avaliação de Outros Cenários Relacionados à Doença Arterial Coronariana

Embora o papel primordial do exame de angio-TC coronária seja a pesquisa direta de DAC, esse exame permite avaliações de diferentes parâmetros relacionados à função ventricular esquerda e ao acometimento patológico do miocárdio. Nesse contexto, é importante ressaltar que, em se tratando de modalidade diagnóstica que se utiliza de radiação ionizante e contraste com potencial nefrotóxico, o seu emprego nas avaliações passíveis de execução por outros métodos (por exemplo, avaliação de função ventricular esquerda pelo ecocardiograma ou avaliação de viabilidade miocárdica pela RM) faz da tomografia recurso de exceção, em caso de falta de acesso ou imagens limitadas pelos outros métodos.

A Tabela 6 traz cenários de utilização da angio-TC cardíaca em outros cenários relacionados à DAC.

**Tabela 6:** Angiotomografia do coração na avaliação de outros cenários relacionados à doença arterial coronariana

Indicações	Classe de recomendação	Nível de evidência
Avaliação da função ventricular esquerda após infarto agudo do miocárdio com imagens inadequadas ou duvidosas por outros métodos não invasivos^25 , 168^	I	B
Avaliação da viabilidade miocárdica (realce tardio pela tomografia computadorizada) em pacientes com programação de revascularização miocárdica por disfunção sistólica do ventrículo esquerdo e que não podem realizar ou possuem imagens inadequadas por outros métodos não invasivos^3 , 25 , 169 – 172^	IIb	B
Triagem de doença vascular do enxerto em pacientes com transplante cardíaco prévio como alternativa à cinecoronariografia^3 , 25 , 173 , 174^	IIa	B

### 2.7. Anomalias de Artérias Coronárias

As anomalias de coronária (AC) são alterações congênitas cardíacas frequentes,^175^ muitas vezes subdiagnosticadas e desconhecidas pelos pacientes, principalmente porque boa parte delas não determina repercussão clínica. Contudo, sabe-se que alguns pacientes podem apresentar quadros de morte súbita, eventos isquêmicos e IC decorrentes de algumas variantes coronarianas.^176^

Dessa forma, cabe aos métodos de imagem não somente confirmar ou descartar o diagnóstico, como também avaliar eventuais riscos associados.^176 , 177^

A ecocardiografia transtorácica dirigida pode ser utilizada como método de rastreamento inicial, sobretudo nas anomalias de origem, desde que realizada de forma dirigida e por mãos experientes.^176 , 178^

Diante de quaisquer anormalidades ou em casos de alta suspeita clínica, é recomendável prosseguir investigação com outro método não invasivo que possa avaliar todo o trajeto coronariano e possíveis repercussões, aqui caracterizados principalmente pela angio-TC das artérias coronárias^179^ e pela RMC.^175 – 184^

Apesar de não haver muitos estudos comparando diretamente a RMC e a angio-TC, este último método costuma ser o preferido para essa avaliação, pois oferece melhor resolução temporal e espacial, sobretudo diante de frequências cardíacas mais elevadas (que são usuais nos pacientes pediátricos) e pela maior disponibilidade e experiência com o método.

Diversos autores mostram preferência pela angio-TC como o padrão-ouro para avaliação das AC em seus estudos. Sabe-se também que a utilização de reconstruções tridimensionais é util na avaliação pré-operatória e tem sido preferida por diversos cirurgiões e clínicos, tornando a angio-TC um método mais atraente.

Já está demonstrado, também, que a angio-TC consegue demonstrar fatores prognósticos relacionados às AC, ajudando na decisão clínica de intervenção, conforme o risco individual do paciente.^175 – 177 , 179 , 180 , 182 – 184^ Destaca-se, por sua vez, que a RM pode trazer informações funcionais importantes, assim como caracterizar eventuais repercussões isquêmicas ou sobre a função cardíaca, podendo ser utilizada de forma complementar.^175 , 176 , 184^

A Tabela 7 classifica a recomendação da angio-TC coronária para a investigação de anomalias das artérias coronárias.

**Tabela 7:** Angiotomografia computadorizada coronária na suspeita de anomalias das artérias coronárias

Indicações	Classe de recomendação	Nível de evidência
Avaliação de pacientes com suspeita de anomalias das artérias coronárias^2 , 3 , 25 , 185 – 187^	I	B

### 2.8. Angiotomografia de Coronárias na Suspeita de Dor Torácica Aguda

A dor torácica aguda é uma das queixas mais frequentes nos atendimentos de emergência, podendo corresponder até 10% das visitas não relacionadas ao trauma e até 40% das causas de internação hospitalar. No entanto, apenas 25% desses pacientes recebem diagnóstico de doença coronária aguda ou outro problema cardíaco significativo ao final da internação, levando a um grande volume de internações desnecessárias e a um alto custo.

Para otimizar o atendimento nas emergências, o uso da angio-CT coronariana encontra bem estabelecido na literatura. Como bem demonstrado por diversos estudos, o método apresenta excelente acurácia para o diagnóstico de estenose em pacientes de baixo a moderado risco cardiovascular, com destaque para seu alto valor preditivo negativo ( Tabela 8 ).^112 , 131 , 132 , 188^

**Tabela 8:** Sumário dos ensaios clínicos multicêntricos sobre acurácia da angiotomografia computadorizada coronariana em detectar estenose coronária (> 50% de estreitamento luminal) em pacientes de baixo a intermediário risco, sem diagnóstico prévio de doença arterial coronariana

Estudo	n	Sensibilidade %	VPN %	Especificidade %	VPP %
CATSCAN,^189^ 7 países, 11 centros	187	94 (89-100)	98 (94-100)	51 (43-59)	28 (19-36)
NIMISCAD,^190^ 20 centros na Itália	327	94 (89-97)	91 (85-95)	88 (81-93)	91 (86-95)
ACCURACY,^131^ 16 centros nos Estados Unidos	230	95 (85-99)	99 (96-100)	83 (76-88)	64(53-75)
CORE64,^112^ 7 países, 9 centros	291	85 (79-90)	83 (75-89)	90 (83-94)	91 (86-95)
Meijboom et al.,^132^ 3 centros na Holanda	360	99 (98-100)	97 (94-100)	64 (55-73)	86 (82-90)

VPN: valor preditivo negativo; VPP: valor preditivo positivo.

O uso da angio-TC coronária na avaliação da dor torácica aguda foi avaliado de forma segura em diversos estudos na estratificação, redução de custo e diminuição do tempo de permanência intra-hospitalar. Estudos prospectivos, controlados e randomizados avaliaram seu uso no contexto da dor torácica no pronto-socorro em pacientes de risco baixo a intermediário associados ao uso da troponina convencional negativa.^191^ Em destaque, há três estudos.

O primeiro é o estudo multicêntrico CT-STAT ( *Coronary Computed Tomographic Angiography for Systematic Triage of Acute Chest Pain Patients to Treatment* ), que randomizou 699 pacientes com dor torácica de baixo risco para estratégias de estratificação utilizando a angio-TC de coronárias ou a cintilografia miocárdica de repouso e estresse.^192^ A estratégia com a angio-TC coronária reduziu em 54% o tempo para o diagnóstico e em 38% os custos da internação, sem que houvesse diferença na taxa de eventos adversos com relação à estratégia com a cintilografia.

O segundo é o estudo multicêntrico ACRIN-PA ( *Angiography for Safe Discharge of Patients with Possible Acute Coronary Syndromes* ), que teve como objetivo primário avaliar a segurança da utilização da angio-TC coronária na avaliação de pacientes com dor torácica de risco baixo a intermediário (TIMI RISK 0 a 2) em comparação com a abordagem tradicional.^193^ Nenhum dos pacientes com angio-TC coronária normal apresentou o desfecho primário (morte cardíaca ou infarto nos primeiros 30 dias após a admissão). Além disso, os pacientes do grupo angio-TC tiveram maior taxa de alta das unidades de emergência (49,6% vs. 22,7%) e menor tempo de internação (18 horas vs. 24,8 horas; p < 0,0001), sem diferença no número de revascularizações ou cateterismos.

O terceiro estudo é o ROMICAT II ( *Rule Out Myocardial Ischemia/ Infarction Using Computer Assisted Tomography* ), que avaliou, em grupos semelhantes de pacientes, o tempo de permanência na emergência e os custos hospitalares.^194^ Esse estudo incluiu 1.000 pacientes com idade média de 54 anos, sendo o tempo de permanência hospitalar significativamente menor nos pacientes estratificados para angio-TC coronária quando comparados ao grupo submetido a avaliação tradicional (23,2±37,0 horas vs. 30,8±28,0 horas; p = 0,0002). O tempo até a exclusão do diagnóstico de síndrome coronariana aguda (SCA) também foi menor no grupo submetido à angio-TC (17,2±24,6 horas vs. 27,2±19,5 horas; p < 0,0001). Em relação às metas de segurança, não houve qualquer diferença entre os grupos. No grupo estratificado pela angio-TC coronária, houve aumento significativo dos pacientes que receberam alta hospitalar diretamente da emergência (46,7% vs. 12,4%; p = 0,001). Os custos globais foram muito similares entre os dois grupos devido ao menor tempo de permanência hospitalar (p = 0,65).

Em resumo, a utilização da angio-TC coronária das artérias coronárias é uma estratégia segura na avaliação de pacientes com dor torácica aguda de risco baixo a intermediário (com ECG não diagnóstico e marcadores de necrose miocárdica negativos [não ultrassensível]), reduzindo a taxa, o tempo de internação e, provavelmente, os custos. Nesse cenário, tem-se a indicação do uso da angio-TC como Classe I, com Nível de Evidência A.

O uso da angio-TC de coronárias na sala de emergência em pacientes com dor torácica associada a elevações da troponina convencional foi avaliado pelo estudo RAPID-CTCA,^195^ que testou a estratégia de uso de angiotomografia vs. manejo convencional em pacientes com diagnóstico de SCA sem supra (com elevação de troponina convencional). Os dados desse estudo mostram que a estratégia com uso de angio-TC não reduziu o desfecho primário proposto (mortalidade por todas as causas ou infarto miocárdico tipo 1 ou 4b no período de 1 ano), com incidência de 5,8% para TC vs. 6,1% para tratamento convencional (p = 0,65) ou revascularizações ( *odds ratio* 1,03, IC95% 0,87-1,21). No entanto, reduziu o número de cateterismos ( *odds ratio* 0,81, IC95% 0,72-0,92), às custas do aumento discreto do tempo de internação de 2,0 para 2,2 dias.

O uso da troponina ultrassensível (hs-Tn) tem ganhado espaço nas salas de emergência por trazer segurança para a alta hospitalar quando negativa. Poucos estudos têm avaliado o uso de angio-TC de coronárias nesse contexto. O BEACON, estudo multicêntrico randomizado, avaliou o uso da angio-TC na sala de emergência em pacientes de risco baixo a intermediário que, após hs-Tn negativa, eram randomizados para realização de angio-TC ou abordagem padrão na sala de emergência, tendo como objetivo primário avaliar o número de revascularizações em 30 dias. O uso da angio-TC de coronárias nesse cenário não resultou em diferenças no número de revascularizações ou de SCA não detectadas e no número de altas da unidade de emergência (65% vs 59%; p = 0,16), apresentando tempo similar de internação hospitalar em ambos os grupos (6,3 horas). Porém, o uso de angio-TC conseguiu reduzir os custos de atendimento (337 vs. 511 euros, p < 0,01) e os testes adicionais após alta hospitalar (4% vs. 10%, p < 0,01).^196^

Portanto, a angio-TC aplicada no início da investigação de suspeita de SCA na sala de emergência é segura e está associada a menos testes e menores custos, devido à menor necessidade de investigação complementar em nível ambulatorial. No entanto, em pacientes com hs-Tn negativas, a angio-TC coronária não identificou mais pacientes com DAC significativa requerendo revascularização coronariana nem encurtou a internação hospitalar ou permitiu maior proporção de altas a partir do pronto-socorro quando comparada à estratégia convencional. O estudo TARGET-CTCA (NCT03952351) está em andamento para avaliar se o tratamento precoce da DAC identificada pela tomografia em pacientes com elevações intermediárias de hs-Tn apresenta impacto na prevenção de eventos futuros após 36 meses da alta.

Alguns estudos têm avaliado o uso da angio-TC de coronárias no contexto de uma hs-Tn elevada. O ensaio CARMENTA^197^ demonstrou ser seguro o uso de angio-TC coronária ou ressonância antes do cateterismo, quando comparado à rotina clínica para selecionar os pacientes com hs-Tn positivas para o cateterismo e sem aumento de eventos cardiovasculares. Portanto, o uso da angio-TC coronária em pacientes com elevações de hs-Tn até níveis intermediários (média do estudo de 78 ng/mL) e sem indicadores de alto risco reduziu a necessidade de cateterismo de forma segura quando comparado à estratégia padrão ( *odds ratio* 0,66, P < 0,001), sem aumento de eventos cardiovasculares após 1 ano de acompanhamento (p = 0,265).

Estudos tentaram avaliar o uso do EC como forma de predizer estenose coronária na sala de emergência. Uma subanálise do estudo CORE64^198^ mostrou baixo valor preditivo negativo (VPN 0,62), sendo que até 39% dos pacientes de alto risco com SCA apresentavam EC zero e 46% tinham valores inferiores a 100 unidades Agaston. Portanto, o EC na sala de emergência para predizer lesões coronárias significativas não deve ser usado à luz de estudos atuais.

#### 2.8.1. Descarte Triplo (Estudo CAPTURE)

A angio-TC coronária pode também ser utilizada na sala de emergência para avaliar o diagnóstico diferencial das SCA. Por meio de protocolos de aquisição específicos, podem ser obtidas informações relativas às artérias coronárias, à aorta e às artérias pulmonares, permitindo a avaliação de síndromes aórticas agudas e tromboembolismo pulmonar, além de permitir análise de outras alterações torácicas (pneumonias, traumas etc.).^199 – 204^ Essa abordagem recebe o nome de descarte triplo ( *triple rule-out* ). Trata-se de um protocolo que inclui no campo de visão analisado não somente as artérias coronárias, mas também a integralidade da aorta torácica e toda a circulação pulmonar, exigindo uma maior quantidade de contraste iodado, bem como um aumento da dose total de radiação do exame. Entretanto, mesmo com técnicas otimizadas, o protocolo de aquisição para o descarte triplo é menos eficiente do que os protocolos individuais para avaliação das artérias coronárias, aorta e artérias pulmonares. Portanto, os protocolos de descarte triplo só devem ser utilizados em situações específicas, nas quais a avaliação clínica é incapaz de direcionar o diagnóstico.

A Tabela 9 traz os principais cenários clínicos relacionados à utilização da angio-TC coronária em vigência de potencial SCA.

**Tabela 9:** Angiotomografia das artérias coronárias na suspeita de síndrome coronária aguda (SCA)

Indicações	Classe de recomendação	Nível de evidência
Avaliação de pacientes com suspeita de SCA de risco baixo ou intermediário com ECG normal ou não diagnóstico e marcadores de necrose miocárdica normais ou alterados, porém sem definição de infarto miocárdico * ^2 , 25 , 132 , 192 – 194 , 205^	I	A
Avaliação de pacientes com dor torácica aguda pela técnica do descarte triplo ( *triple rule-out* )^2 , 25 , 203 , 206^	IIb	B
Avaliação de pacientes com suspeita de SCA de risco alto^20^	III	C
Avaliação de pacientes com diagnóstico definitivo de infarto do miocárdio^20^	III	C

* Definido com alteração situada no limite superior da referência do ensaio (~ percentil 99 do ensaio utilizado) e/ou alterações de marcador de necrose miocárdica potencialmente justificadas por outra(s) condição(ões) concomitante(s). ECG: eletrocardiograma.

### 2.9. Angiotomografia de Coronárias na Avaliação Pré-operatória

A avaliação pré-operatória de cirurgias cardíaca e não cardíaca já tem literatura estabelecida, com critérios definidos por diversas sociedades médicas.^207 – 210^ O algoritmo pode incluir o estudo por angio-TC das artérias coronárias como um dos exames que pode oferecer informações adicionais.^207^ A presença e a extensão da DAC são as principais informações a serem fornecidas.

No caso de cirurgias cardíacas, diversos estudos têm demonstrado o seu potencial como alternativa ao padrão ouro (cinecoronariografia), ressaltando-se seu alto VPN.^160 , 163^ Adicionalmente, a avaliação funcional (pela FFR-TC) vem sendo incorporada ao estudo anatômico como um incremento diagnóstico robusto, aumentando o potencial de informações do exame. O ensaio FASTTRACK CABG, publicado recentemente, analisou os resultados da estratégia e o planejamento da revascularização miocárdica somente com base na anatomia e avaliação funcional por tomografia. O desfecho primário de segurança, definido como patência de enxertos no seguimento de 30 dias, demonstrou uma patência das anastomoses de 92,6%, com uma incidência de eventos cardiovasculares maiores de 7,2% e taxa de sangramentos maiores de 2,7%. Estes dados encorajadores ampliam o potencial da angio-TC de coronárias como ferramenta suficiente para o planejamento de revascularizações miocárdicas cirúrgicas.^211^ A investigação da DAC por tomografia em pacientes com indicações de cirurgia valvar pode ser considerada nos casos de pacientes com probabilidade baixa ou intermediária, e a cinecoronariografia deve ser indicada em casos positivos ou duvidosos.^212^

O baixo risco do estudo por tomografia associado à sua alta acurácia diagnóstica comparada à cinecoronariografia não deve significar que sua utilização seja entendida como método de eleição na estratificação não invasiva de candidatos a cirurgias não cardíacas. Portanto, não há evidências, atualmente, que suportem a indicação de seu uso rotineiro na avaliação coronariana pré-operatória.^2 , 25^

As Tabelas 10 e 11 apresentam as recomendações acerca da utilização da angio-TC coronariana na avaliação pré-operatória de pacientes encaminhados a cirurgias cardíacas e não cardíacas.

**Tabela 10:** Angiotomografia das artérias coronárias na avaliação pré-operatória de cirurgia não cardíaca

Indicações	Classe de recomendação	Nível de evidência
Avaliação das artérias coronárias no pré-operatório de cirurgia vascular arterial em pacientes com estimativa de risco intermediário ou alto de complicações^2 , 3 , 25 , 207 , 213 – 215^	IIa	B
Avaliação das artérias coronárias no pré-operatório de cirurgia de risco intermediário em pacientes com estimativa intermediária ou alta de complicações e baixa capacidade funcional^2 , 3 , 25 , 207 , 213 – 215^	IIb	C

**Tabela 11:** Angiotomografia das artérias coronárias na avaliação pré-operatória de cirurgia cardíaca não coronariana

Indicações	Classe de recomendação	Nível de evidência
Avaliação das artérias coronárias no pré-operatório de cirurgia cardíaca não coronariana em pacientes com probabilidade pré-teste baixa ou intermediária de DAC^2 , 3 , 25 , 216 – 218^	I	B

### 2.10. Avaliação de Valvopatias pela Angiotomografia

A avaliação inicial das valvopatias é realizada pela ecocardiografia, exame amplamente disponível e sem radiação ionizante. Porém, nos pacientes com imagens inadequadas ou duvidosas por esse método, a angio-TC do coração direcionada pode ser uma alternativa tanto para avaliar a morfologia e a função das valvas e próteses valvares como as dimensões das câmaras cardíacas e as funções ventriculares associadas.^25 , 168 , 219 – 225^

A angio-TC do coração para avaliação das valvopatias deve ser direcionada para a dúvida clínica, e a sua programação é realizada diferentemente para cada valva cardíaca, com o objetivo de adquirir as imagens com constraste adequado nas câmaras cardíacas de interesse (direitas vs. esquerdas) e ajustar a modulação da dose de radiação nas fases específicas do intervalo R-R para se obter imagens diagnósticas para medição da área de abertura valvar (graduação da estenose) e do orifício regurgitante (graduação da insuficiência), sendo um exame mais bem realizado e interpretado por especialistas com experiência nesse cenário.

Abaixo ( Tabela 12 ), encontram-se as principais indicações relacionadas ao emprego da tomografia na avaliação das valvas cardíacas.

**Tabela 12:** Avaliação de valvopatias pela tomografia

Indicações	Classe de recomendação	Nível de evidência
Avaliação de valvas nativas em pacientes com suspeita de disfunção valvar significativa com imagens inadequadas ou duvidosas por outros métodos não invasivos^25 , 219 – 224^	I	B
Avaliação de próteses valvares em pacientes com suspeita de disfunção valvar significativa com imagens inadequadas ou duvidosas por outros métodos não invasivos^25 , 219 – 225^	I	B
Avaliação das dimensões das câmaras cardíacas e da função ventricular associada à disfunção valvar significativa com imagens inadequadas ou duvidosas por outros métodos não invasivos^25 , 168^	I	B

### 2.11. Avaliação Pré-implante Percutâneo de Valva Aórtica (TAVI/ViV)

A estenose aórtica grave acomete 2,9% dos idosos com idade entre 75 e 86 anos.^226^ Ensaios clínicos randomizados^227 – 229^ demonstraram que o implante de valva aórtica transcateter (TAVI) em pacientes com estenose aórtica grave é factível, seguro e relacionado a desfechos cardiovasculares superiores à troca valvar cirúrgica na população de alto risco cirúrgico. No Brasil, o TAVI vem apresentando crescimento expressivo no número de implantes/ano,^230^ chegando a 1.400 implantes em 2018.

A seleção dos pacientes candidatos ao TAVI, a escolha do tipo (balão ou autoexpansível) e tamanho da prótese e o acesso devem ser avaliados pela equipe composta por cardiologista clínico, cardiologista intervencionista, cirurgião cardíaco, ecocardiografista e especialista em TC cardiovascular – o chamado " *heart team* ". Nesse contexto, a TC do complexo valvar aórtico, coração, aorta total e artérias ilíacas e femorais comuns é indispensável para a melhor tomada de decisão.

As melhores técnicas de aquisição e reconstrução de imagens, assim como as medidas necessárias para o planejamento do TAVI, já foram descritas em recomendações de sociedades internacionais de TC cardiovascular.^9^ A avaliação do complexo valvar aórtico inclui necessariamente as medidas do anel valvar aórtico (eixos maior e menor, área e perímetro) em sístole, visto que há variação dessas medidas durante o ciclo cardíaco e que a seleção do tamanho da prótese baseada em medidas durante a diástole pode acarretar subdimensionamento^231^ e consequente " *leak* " paraprotético, que está relacionado a maior mortalidade.^232^ A escolha do tamanho da prótese baseada nas medidas da TC está relacionada a menor incidência de " *leak* " que a definição a partir das medidas do ecocardiograma bidimensional.^233^

A altura dos óstios das artérias coronárias também é fundamental, dado o risco de oclusão pelo dispositivo em pacientes com tronco da coronária esquerda com altura < 10 mm ou artéria coronária direita com altura < 12 mm em relação ao plano do anel valvar aórtico.^234^ Nos pacientes candidatos à intervenção " *valve-in-valve* ", a TC pode ainda projetar a posição da neoprótese em relação aos óstios das coronárias, que frequentemente estão mais próximos do plano do anel da prótese disfuncionante, e identificar pacientes com maior risco de oclusão.^235^

O grau de extensão da calcificação valvar aórtica para a via de saída do ventrículo esquerdo é outro parâmetro importante na avaliação do candidato ao TAVI. Pacientes com calcificação importante nessa topografia apresentam risco aumentado de ruptura durante o procedimento, uma intercorrência associada a elevada taxa de mortalidade.^236^

O comprimento do septo perimembranoso < 8 mm está associado a maiores taxas de bloqueios atrioventriculares avançados e a necessidade de marca-passo definitivo, podendo ser facilmente avaliado pela TC. Além disso, a avaliação pela TC ainda deve incluir a predição do melhor ângulo para fluoroscopia durante o procedimento, potencialmente reduzindo o volume total de contraste necessário para o implante.

A avaliação da anatomia coronária no candidato ao TAVI está frequentemente prejudicada por diversos fatores: condições clínicas que dificultam a apneia, impossibilidade de controle da frequência cardíaca com betabloqueadores, contraindicação ao uso de vasodilatadores e calcificação intensa dos vasos. No entanto, nos estudos com boa qualidade de imagem, o VPN para detecção de lesões obstrutivas significativas permanece alto.^237^ Nesse cenário, a realização de angio-TC coronariana concomitante com o planejamento pré-TAVI pela TC tem classe de recomendação IIa, Nível de evidência B ( Tabela 13 ).

**Tabela 13:** Tomografia no planejamento do implante percutâneo da valva aórtica (TAVI/TAVR)

Indicações	Classe de recomendação	Nível de evidência
Em pacientes candidatos ao TAVI, angiotomografia do coração, da aorta torácica e abdominal e das artérias ilíacas e femorais comuns deve ser realizada para programação do procedimento^2 , 235 , 240 – 243^	I	A
Em pacientes candidatos ao TAVI ViV, angiotomografia do coração, da aorta torácica e abdominal e das artérias ilíacas e femorais comuns deve ser realizada para programação do procedimento^2 , 9 , 235 , 244 – 246^	I	B
Em pacientes com dúvida sobre a gravidade da estenose aórtica pela ecocardiografia, a angiotomografia do coração pode ser realizada para avaliar a morfologia, a função valvar e a gravidade da estenose aórtica^25 , 219 – 223 , 225 , 247 – 250^	I	B
Em pacientes com suspeita de estenose aórtica importante de baixo fluxo, baixo gradiente e com FEVE preservada (≥ 50%) ou com FEVE reduzida e sem reserva contrátil pela ecocardiografia sob estresse com dobutamina, pode ser realizado o escore de cálcio da valva aórtica para avaliar a possibilidade de estenose aórtica importante (≥ 1.300 AU para mulheres e ≥ 2.000 AU para homens)^212 , 251 – 253^	IIa	B
Em pacientes com suspeita de trombose dos folhetos da prótese percutânea pós-TAVI, a angiotomografia do coração pode ser realizada^9 , 254 , 255^	I	B
A angiotomografia das artérias coronárias pode ser realizada em pacientes selecionados candidatos ao TAVI e interpretada em caso de qualidade adequada para o diagnóstico^256 , 257^	IIa	B

TAVI: implante de valva aórtica transcateter; TAVR: substituição percutânea da valva aórtica; VIV: valve-in-valve; FEVE: fração de ejeção do ventrículo esquerdo.

Finalmente, a TC fornece ainda informações fundamentais para a avaliação e escolha das vias de acesso para o implante: caracterização da aorta, artérias ilíacas e femorais comuns quanto à presença de ateromatose e grau de obstrução quando presente, diâmetro luminal mínimo, tortuosidades e presença de calcificações circunferenciais. A doença arterial periférica é bastante prevalente entre candidatos ao TAVI^238^, e a avaliação pela TC é superior à avaliação invasiva na predição de complicações vasculares relacionadas ao procedimento.^239^ Nos pacientes em que o acesso transfemoral é impossível, a TC permite avaliação do ápice do ventrículo esquerdo quanto à presença de trombos, calcificação e/ou afilamentos que contraindiquem a via de acesso transapical; a aorta ascendente também deve ser avaliada quanto à presença, extensão e localização de calcificação parietal para o acesso transaórtico.

Por ser fundamental no planejamento de TAVI, a TC deve ser utilizada em candidatos ao procedimento. A Tabela 13 traz as principais recomendações de seu uso nesse planejamento.

### 2.12. Planejamento Percutâneo de Outras Alterações Estruturais

Os grandes avanços no tratamento percutâneo das doenças valvares possibilitaram a abordagem de outras valvopatias além da estenose aórtica, com um amplo número de próteses específicas para cada valvopatia, algumas validadas na prática clínica e com menor risco relacionado ao procedimento do que uma cirurgia cardíaca corretiva.

A angio-TC do coração associada em alguns cenários a outras angio-TCs é fundamental para o melhor planejamento e indicação do procedimento, podendo ajudar na escolha do tamanho da prótese a ser implantada, além de prever o risco de complicações que podem ser impeditivas à sua realização.^258 – 267^ A seguir ( Tabela 14 ), encontram-se os principais cenários relacionados à utilização da TC no suporte à abordagem percutânea de outras cardiopatias estruturais.

**Tabela 14:** Angiotomografia do coração no planejamento de intervenções percutâneas para outras cardiopatias estruturais

Indicações	Classe de recomendação	Nível de evidência
Em pacientes candidatos ao implante percutâneo da valva mitral (TMVR), *valve-in-valve* , *valve-in-ring* ou *valve-in-MAC* , a angiotomografia do coração deve ser realizada para programação do procedimento e avaliação do risco de complicações^258 – 261^	I	B
Em pacientes candidatos ao implante percutâneo da valva pulmonar, a angiotomografia do coração e das artérias pulmonares pode ser realizada para programação do procedimento como alternativa à ressonância magnética, principalmente quando houver risco de compressão das artérias coronárias^262 , 263^	I	B
Em pacientes candidatos ao implante percutâneo da valva tricúspide, *valve-in-valve* ou no anel valvar, a angiotomografia do coração deve ser realizada para programação do procedimento^264 – 267^	I	B
Em pacientes candidatos ao implante percutâneo de próteses bicavais para insuficiência tricúspide, a angiotomografia do coração, venosa do tórax e venosa do abdome superior deve ser realizada para programação do procedimento^267^	I	C

### 2.13. Avaliação das Veias Cardíacas, Átrio Esquerdo e Avaliação de Veias Pulmonares (Incluindo Planejamento de Ablação de Fibrilação Atrial/Oclusão Apêndice Atrial)

A caracterização adequada da anatomia cardíaca e vascular é importante no auxílio aos procedimentos eletrofisiológicos tanto na fase de planejamento quanto no controle e monitorização de possíveis complicações, em especial na ablação das veias pulmonares para tratamento da fibrilação atrial (FA). A angio-TC, por ser um método de rápida aquisição e por fornecer imagens com amplo campo de visão, alta resolução espacial e reconstrução tridimensional, é uma excelente ferramenta para a determinação da anatomia vascular.^25 , 268 , 269^

A identificação correta da anatomia das veias pulmonares e do átrio esquerdo é fundamental para a segurança e o sucesso do procedimento de ablação da FA. A anatomia das veias pulmonares é marcada por grande variabilidade entre os indivíduos no que tange ao número, às dimensões dos óstios e ao padrão de bifurcação.^270^ As variações mais comumente encontradas são a presença de veias pulmonares supranumerárias (18-29%) e a presença de tronco comum (> 30%), principalmente à esquerda, além da presença de veia pulmonar do lobo médio e veia do topo.^271^ Pela TC, é possível avaliar o número de veias pulmonares, seus respectivos óstios e diâmetros, a presença de variações anatômicas ou anomalias de drenagem.^272 , 273^ As medidas não apenas dos diâmetros dos óstios, mas também da sua área e esfericidade, da angulação das veias pulmonares e da distância do óstio até a primeira bifurcação também podem ser úteis no planejamento da ablação quando se utiliza a técnica de crioablação.^274^ A TC também pode ser útil na definição anatômica das veias cavas, na exclusão de trombos atriais e na identificação da localização e curso do esôfago, assim como na identificação da presença da fossa oval e de qualquer anormalidade que possa interferir na punção transeptal, como hipertrofia lipomatosa do septo interatrial. Uma definição anatômica precisa permitir a escolha mais adequada da técnica de ablação a ser realizada e um planejamento mais adequado do procedimento, reduzindo o seu tempo de realização e a chance de complicações.^271^

As imagens da TC também podem ser fundidas com as imagens do mapeamento eletroanatômico ou da fluoroscopia.^275 , 276^ Vários estudos têm sugerido que essas técnicas podem reduzir o tempo de duração do procedimento, a taxa de recorrência da FA e a exposição à radiação.^271^ Entretanto, as evidências da literatura ainda são controversas no que se refere ao seu real benefício clínico.^268 , 269^

Outra utilidade da angio-TC nesse grupo de pacientes está relacionada com o acompanhamento pós-ablação.^273 , 277^ As duas principais complicações relatadas são lesão esofágica e estenose das veias pulmonares. A angio-TC apresenta alta especificidade para detectar estenose de veias pulmonares, cuja incidência reportada é de 0,29%.^278^ A angio-TC pode identificar a localização, a extensão e o grau da estenose das veias pulmonares, além de permitir a comparação do achado com as imagens pré-procedimento. Além disso, com a TC, é possível avaliar a presença de opacidades pulmonares sugestivas de infartos venosos ou alterações na gordura mediastinal adjacente ou linfadenopatias.

A angio-TC também é capaz de detectar trombos intracavitários, identificados como imagens de baixa atenuação localizadas sobretudo na aurícula esquerda.^271 , 279^ Estudos recentes têm demonstrado grande valor diagnóstico do método para exclusão de trombo auricular, sobretudo quando utilizadas técnicas de aquisição tardia (vários segundos após a infusão do meio de contraste), com metanálise demonstrando acurácia diagnóstica de 94% e VPN de 99%.^280^ Apesar de o ecocardiograma transesofágico ser o padrão-ouro para detecção de trombo atrial e a angio-TC ainda não ser recomendada de maneira rotineira para esse propósito,^268^ o método demonstrou aplicação prática durante a pandemia pelo SARS-COV-2 (coronavírus 2 da síndrome respiratória aguda grave), em que se procurou limitar a realização de procedimentos invasivos desnecessários.^281^

Além disso, a adequada caracterização morfológica e a localização da aurícula esquerda também são importantes para o procedimento de oclusão dela, em geral indicada para pacientes que têm contraindicação à utilização de anticoagulantes. Diferentes padrões morfológicos foram descritos, com o padrão " *chicken wing* " sendo o mais comum (48%), seguido pelo " *cactus* " (30%), " *windsock* " (19%), e " *cauliflower* " (3%), este último mais associado a eventos tromboembólicos.^278 , 282^ Alguns parâmetros importantes para o implante do dispositivo oclusor auricular podem ser obtidos pela TC, como a morfologia do óstio, o comprimento e angulação da aurícula.^283^

#### 2.13.1. Técnica

A técnica de aquisição da angio-TC para avaliação das veias pulmonares varia de acordo com o equipamento, mas, em linhas gerais, é semelhante à técnica utilizada para aquisição da angio-TC de coronárias. Uma fase confirmatória tardia pode ser realizada caso exista imagem sugestiva de trombo em apêndice atrial. A Tabela 15 apresenta as indicações da TC na avaliação de veias pulmonares e apêndice atrial esquerdo.

**Tabela 15:** Angiotomografia na avaliação do átrio esquerdo, das veias pulmonares e das veias cardíacas

Indicações	Classe de recomendação	Nível de evidência
Avaliação do átrio esquerdo, do apêndice atrial esquerdo e das veias pulmonares pré-ablação de fibrilação atrial^2 , 25 , 268^	I	B
Avaliação da presença de trombo em átrio esquerdo/apêndice atrial esquerdo em pacientes com fibrilação atrial como alternativa ou com imagens inconclusivas pela ecocardiografia transesofágica^2 , 3 , 25 , 268 , 269 , 280^	I	B
Em pacientes candidatos ao fechamento percutâneo do apêndice atrial esquerdo para programação do procedimento^268 , 269 , 284 – 287^	I	B
Avaliação da anatomia das veias cardíacas pré-implante de ressincronizador cardíaco^2 , 25 , 288 , 289^	IIa	B

### 2.14. Avaliação Funcional por Tomografia Computadorizada

#### 2.14.1. Perfusão Miocárdica por Tomografia Computadorizada

A avaliação de isquemia miocárdica com a utilização da TC (PMTC) tornou-se possível há alguns anos. Trata-se de uma técnica que avalia a primeira passagem do contraste iodado no miocárdio ventricular, sob ação de estresse farmacológico vasodilatador. A técnica envolve a utilização de duas aquisições; uma delas é dedicada à avaliação das artérias coronárias (TC de coronárias propriamente dita), bem como para a avaliação da perfusão do miocárdico ao repouso; a outra aquisição é voltada para a avaliação da perfusão miocárdica, sendo realizada sob estresse farmacológico (mais comumente dipiridamol ou adenosina em nosso meio).

A técnica de PMTC encontra robusta validação em relação à sua performance diagnóstica, tendo como referência a cintilografia de perfusão miocárdica,^4^ a RMC,^290^ e o FFR invasivo.^291^ Entre as técnicas empregadas, é possível se avaliar a perfusão sob estresse em um único momento da administração do contraste (perfusão estática) ou em aquisições de sequências durante a chegada do contraste no miocárdio (perfusão dinâmica). As duas técnicas apresentam acurácias semelhantes, diferindo entre si pelo fato de a perfusão dinâmica permitir avaliações quantitativas (estimativa de fluxo sanguíneo miocárdico regional e global, " *upslope* " da curva de contraste e pico de atenuação máxima), o que pode trazer discretos ganhos em relação à acurácia.^292 – 294^ Uma terceira técnica de aquisição, com a utilização de dois níveis de energia na obtenção de imagens estáticas (técnica de dupla energia), pode melhorar a acurácia da avaliação da perfusão miocárdica,^295 , 296^ tendo em vista melhorias na relação contraste-ruído entre os diferentes tecidos.

Equipamentos com 64 colunas de detectores ou superiores são necessários para a realização dessa técnica. A sequência de realização das etapas do exame (fase de estresse inicialmente ou a TC de coronárias inicialmente) privilegia a avaliação funcional ou anatômica, respectivamente, e sua escolha depende da experiência local. Um fator que pode determinar a escolha da fase de repouso (TC de coronárias) inicialmente é a possibilidade de cancelamento da fase de estresse se a TC de coronárias apresentar vasos completamente normais, sem estenoses.

#### 2.14.1.1. Acurária Diagnóstica

A PMTC é uma técnica que deve ser utilizada em conjunto com a avaliação da TC de coronárias, fornecendo informações anatômicas e perfusionais em um mesmo exame. Nesse sentido, a acurácia diagnóstica é melhor avaliada considerando-se o alinhamento anatômico-perfusional, buscando-se a identificação de estenoses associadas a evidências de isquemia. O estudo CORE320 analisou a combinação da TC de coronárias com a PMTC na identificação de estenoses fluxo-limitantes, tendo a combinação de cintilografia miocárdica e cateterismo cardíaco como referência.^1^ Considerando-se todos os pacientes, a acurácia da análise combinada (medida pela área sobre a curva ROC) foi de 0,87, chegando a 0,93 nos pacientes sem doença coronariana conhecida.

Na tentativa de comparação com demais métodos funcionais, Takx et al. avaliaram a performance diagnóstica de diversos testes funcionais, tendo a FFR invasiva como referência.^297^ Nesse sentido, a PMTC demonstrou uma acurácia de 93% na detecção de estenoses associadas à presença de isquemia miocárdica, com desempenho equivalente à TC por emissão de pósitrons e à RMC. A Tabela 16 apresenta os parâmetros de performance diagnóstica da PMTC em relação aos demais métodos não invasivos.

**Tabela 16:** Desempenho diagnóstico da perfusão miocárdica por tomografia computadorizada em relação a diferentes modalidades diagnósticas *

Referência	Ano	N	Referência	Sens.	Espec.	VPP	VPN
George et al.^298^	2012	50	CPM	72	91	81	85
Bettencourt et al.^290^	2013	101	FFR	89	83	80	90
Rochitte et al.^4^	2014	381	CATE e CPM	80	74	65	86
Cury et al.^299^	2015	110	com	90	84	36	99
Takx et al.^297^	2015	2.048	FFR	88	80	-	-
Sørgaard et al.^300^	2016	1.188	CPM, RMC, CATE, FFR	85	81	-	-
Pontone et al.^301^	2019	100	CATE e FFR	98	54	68	96

CATE: cateterismo cardíaco; CPM: cintilografia de perfusão miocárdica; Sens.: sensibilidade; Espec.: especificidade; VPP: valor preditivo positivo; VPN: valor preditivo negativo; FFR: reserva de fluxo fracionada.

* Modificado de Magalhães et al.^302^

#### 2.14.1.2. Valor Prognóstico

Embora sejam utilizadas extrapolações de vasta literatura acerca do valor prognóstico da carga isquêmica identificada por demais métodos funcionais (por exemplo, cintilografia de perfusão miocárdica e RM com estresse), são poucos os dados referentes à utilização da PMTC e correlação com desfechos clínicos. Em estudo recente, Dewey et al.^303^ apresentaram o seguimento de 5 anos do estudo CORE320. Nessa avaliação, os autores observaram um valor prognóstico equivalente da combinação da TC coronárias e PMTC e da combinação de CPM + CATE (AUC ROC para predição de eventos cardiovasculares maiores foi de 0,65 para ambas as abordagens).

#### 2.14.1.3. Aplicabilidade em Diferentes Cenários Clínicos

A possibilidade de combinação de avaliação de anatomia coronariana e repercussão funcional de estenoses coronarianas em um único exame torna a PMTC uma ferramenta muito atrativa na pesquisa de doença coronariana. Especificamente, um grupo de pacientes que se beneficia dessa abordagem é aquele com doença coronariana conhecida e/ou com risco cardiovascular aumentado, bem como pacientes portadores de *stents* .^149 , 304^ Nesses cenários, onde reconhecidamente existe uma perda de especificidade da angio-TC por limitações da avaliação luminal, a utilização da PMTC pode contribuir com informações que permitem identificar a repercussão no fluxo miocárdico de eventuais estenoses coronarianas.

Adicionalmente, os pacientes cuja anatomia apresente estenoses intermediárias pela angio-TC coronária, cuja repercussão hemodinâmica seja duvidosa, bem como aqueles com estenoses cuja extensão de isquemia precise ser quantificada podem ter informações úteis com a adição da PMTC à investigação.

#### 2.14.2. Reserva de Fluxo Fracionado por Tomografia Computadorizada (FFR-TC)

A angio-TC de coronárias tem sua grande utilidade nos grupos de pacientes com probabilidade baixa a intermediária de DAC, sobretudo por conta do seu alto VPN.^305 , 306^ Entretanto, a especificidade para a detecção de DAC hemodinamicamente significativa é limitada, principalmente no que tange às estenoses moderadas.^307^

A medida da FFR de modo invasivo é hoje o padrão-ouro para determinar a significância hemodinâmica da DAC.^308 – 310^ Com o incremento tecnológico, atualmente tem-se a possibilidade da mensuração da FFR através da TC (FFR-TC).

A FFR-TC é uma tecnologia que usa os princípios de dinâmica de fluidos para gerar um modelo tridimensional baseado nas informações derivadas da angio-TC coronariana, sem a necessidade de emprego maior de contraste, radiação ou medicação, uma vez que é uma análise de pós-processamento das imagens fornecidas pela angio-TC coronariana.^311^

Basicamente, há quatro princípios básicos que envolvem o cálculo da FFR-TC baseada em dinâmica de fluidos. O primeiro é que a resistência microvascular é inversamente relacionada com o diâmetro das coronárias epicárdicas. O segundo é que o modelo tem a capacidade de extrair o miocárdio da angio-TC de coronárias e determinar o fluxo sanguíneo miocárdico ao repouso. O terceiro princípio é que a hiperemia coronariana máxima é previsível e pode ser calculada baseada em respostas preestabelecidas à adenosina. Finalmente, através das equações de Navier-Stokes, pode-se determinar o fluxo e a pressão do sangue ao longo das artérias coronárias.^312^

Os três primeiros estudos publicados que validaram o método demonstraram boa performance diagnóstica quando comparados à FFR,^313 – 315^ utilizando-se o valor de FFR ≤ 0,8 como positivo, conforme demonstrado na Tabela 17 .

**Tabela 17:** Performance diagnóstica dos três primeiros estudos entre FFR-TC vs. FFR invasiva

Referência	Sens.	Espec.	VPP	VPN	Acurácia
DiscoverFlow^311^	93	82	85	91	88
DeFACTO^314^	90	54	67	84	73
NXT^315^	86	79	65	93	81

Sens.: Sensibilidade, Espec.: especificidade, VPP: valor preditivo positivo, VPN: valor preditivo negativo; FFR: reserva de fluxo fracionada; FFR-TC: FFR por tomografia computadorizada.

Mais recentemente, uma metanálise compilando 1.852 pacientes e 2.731 vasos demonstrou sensibilidade e especificidade do FFR-TC de, respectivamente, 89% e 71% na análise por paciente e de 85% e 82% na análise por vaso.^164^ A sensibilidade da FFR-TC não mostrou diferença estatística em relação à sensibilidade da angio-TC de coronárias. No entanto, a especificidade da FFR-TC foi significativamente maior (71% *versus* 32%, p < 0,001), traduzida como a habilidade do método em detectar estenoses coronárias que determinam restrição de fluxo. Desse modo, a baixa especificidade da angio-TC de coronárias agora é suplantada pela análise combinada com a FFR-TC que pode, acuradamente, diagnosticar a significância funcional das estenoses coronarianas.

O estudo RIPCORD avaliou a tomada de decisão, retrospectivamente, de 200 pacientes derivados do estudo NXT.^316^ Depois que os valores de FFR-TC foram revelados e o número de estenoses significativas foram analisadas, houve mudança do plano terapêutico em 36% dos casos.

No estudo prospectivo PLATFORM, que envolveu 584 pacientes com dor torácica estável e probabilidade intermediária de DAC divididos em braço invasivo e não invasivo, foi demonstrado que os pacientes avaliados com angio-TC de coronárias e FFR-TC apresentaram menores taxas de cateterismo cardíaco (CATE) com DAC não significativa.^18^ Enquanto 73% dos pacientes do grupo invasivo tinham doença não significativa ao CATE, somente 12% do grupo FFR-CT apresentaram esse resultado. Cabe ressaltar que, no grupo não invasivo, 61% dos estudos invasivos foram cancelados, e, na análise com 1 ano de seguimento, não houve evento adverso nesses pacientes em que o CATE foi cancelado.^23^

Em um subestudo do ensaio PROMISE envolvendo 67% dos pacientes desse estudo e avaliados por meio da FFR-TC, observou-se que a disponibilidade desse dado levaria a uma associação significativamente maior com eventos cardiovasculares adversos maiores (MACE, de *major adverse cardiovascular events* ) ou revascularização em comparação com a análise visual da angio-CT. Ademais, reservando a estratégia invasiva para os pacientes com FFR-TC ≤ 0,8, reduziria a taxa de DAC não obstrutiva em 44% e aumentaria a taxa de revascularização em 24%.^317^

Em resumo, esses dados consistentemente demonstram que a FFR-TC altera significativamente o diagnóstico de DAC obstrutiva com restrição de fluxo e, com isso, o manejo de cerca de 25% dos pacientes submetidos à técnica. Além disso, o cancelamento de CATE nos pacientes com FFR-TC negativa (> 0,8), mesmo naqueles com DAC na angio-CT coronariana, tem se mostrado uma estratégia segura.

Apesar de tais evidências, a análise da FFR-TC até o momento da publicação desta Diretriz permanece *off-site* e restrita a um único *core-lab* . Novos algoritmos baseados na utilização de inteligência artificial^318^ permitem a análise da FFR-TC *on-site* , porém ainda não está disponível para o uso na prática clínica.^319 – 323^

No que tange à custo-efetividade, os estudos que prospectam o impacto econômico da integração da angio-TC de coronárias com a FFR-TC têm projetado resultados promissores. Uma estimativa baseada nos resultados do DISCOVER FLOW projetam uma redução de 30% nos custos ao longo de 1 ano.^324^ No Reino Unido, uma análise retrospectiva do uso da FFR-TC em estenoses entre 10-90% chega a estimar economia de 200 libras por paciente.^325^ O estudo PLATFORM incluiu uma avaliação econômica preespecificada que demonstrou uma redução média em 1 ano por paciente, de US$ 12.145 para US$ 8.127.^5^

Embora os resultados sejam favoráveis ao emprego da FFR-TC, é importante salientar que o sucesso dessa ferramenta depende de uma exame de angio-CT coronariana de qualidade (mínima quantidade de artefatos de movimento e boa relação contraste-ruído).^326^ Embora o grau de calcificação possa impactar na acurácia dos resultados da FFR-TC, não há um valor preestabelecido que impeça a sua avaliação.^327^ Por fim, como se trata de uma análise específica da lesão, nos cenários de doença difusa, sua utilização não é recomendada, assim como em pacientes com revascularizados ou com *stents* coronarianos.

A Tabela 18 traz os principais cenários clínicos de indicação da avaliação funcional por tomografia (perfusão miocárdica por TC e FFR-TC).

**Tabela 18:** Tomografia na avaliação funcional da doença arterial coronariana (DAC)

Indicações	Classe de recomendação	Nível de evidência
Avaliação de isquemia miocárdica pela tomografia de perfusão miocárdica sob estresse farmacológico como alternativa a outros testes de isquemia por imagem^3 , 4 , 290 , 297 , 299 , 300 , 303^	I	A
Avaliação de isquemia miocárdica pela tomografia de perfusão miocárdica sob estresse farmacológico associada a angiotomografia das artérias coronárias na avaliação de pacientes sintomáticos com DAC conhecida^3 , 4 , 149 , 290 , 296 , 297 , 299 – 301 , 303 , 304^	IIa	A
Avaliação de isquemia miocárdica pela tomografia de perfusão miocárdica sob estresse farmacológico associada a angiotomografia das artérias coronárias na avaliação de pacientes sintomáticos sem DAC conhecida^3 , 4 , 290 , 297 , 299 , 300 , 303^	IIb	B
Avaliação do significado funcional (isquemia) pela reserva de fluxo fracionada (FFR) por tomografia em pacientes com estenose(s) moderada(s) do leito nativo pela angiotomografia das artérias coronárias^3 , 5 – 8 , 167 , 315^	IIa	B
Avaliação do significado funcional (isquemia) pelo FFR por tomografia em pacientes com estenose ≥ 50% do tronco de coronária esquerda, estenose importante triarterial, reestenose de *stent* ou estenose significativa de enxertos cirúrgicos pela angiotomografia das artérias coronárias^3^	III	C

### 2.15. Tomografia na Avaliação das Cardiomiopatias Não Isquêmicas

Uma das principais colaborações da TC em pacientes com cardiomiopatias com fração de ejeção reduzida consiste na exclusão de etiologia isquêmica pela técnica de angio-TC das artérias coronárias. Dessa forma, na pesquisa da etiologia da IC com fração de ejeção reduzida, a cineangiocoronariografia invasiva em pacientes sintomáticos é considerada classe I.^141 – 143 , 328 – 332^ Outros dados demonstram excelente correlação da angio-TC das artérias coronárias com a cineangiocoronariografia invasiva em pacientes sem doença arterial coronariana conhecida e disfunção sistólica global importante com fração de ejeção do ventrículo esquerdo (FEVE) < 35% (sensibilidade de 98%, [intervalo de confiança 94-99%], especificidade de 97% [intervalo de confiança 94-98%] e AUC de 0,99 [p < 00001]).^332^

Dessa maneira, a angio-TC das artérias coronárias pode ser utilizada como método não invasivo primordial na exclusão da etiologia isquêmica em pacientes com cardiomiopatias com nível de evidência I, sendo definitivamente apropriada.

#### 2.15.1. Tomografia na Avaliação de Função Ventricular

A aquisição das imagens das artérias coronárias pela TC, com protocolo retrospectivo guiado pelo eletrocardiograma, permite a reconstrução das imagens do coração em várias fases diferentes do ciclo cardíaco. Dessa forma, é possível avaliar a volumetrias das câmaras cardíacas em suas respectivas diástole e sístole máximas e, portanto, permite mensurar a função sistólica e fração de ejeção biventricular.

Uma metanálise de 12 estudos^333^ comparando a análise da função sistólica pela TC tendo a RM e o ecocardiograma transtorácico como referências demonstrou excelente correlação entre a avaliação da função ventricular esquerda pela TC e a RM, com *bias* de 0,0 (desvio padrão [DP] −3,7, 3,7, intervalo de confiança de 95% [IC95%] 1,96), bem como excelente concordância entre a avaliação da função ventricular esquerda entre a TC e o ecocardiograma transtorácico, com *bias* de 0,3 (DP −4,7, 5,7, IC95% 1,96).

Dessa forma, a avaliação da função ventricular esquerda pela TC pode ser utilizada como alternativa ao ecocardiograma e/ou RM empregados na avaliação da função ventricular nas cardiomiopatias não isquêmicas paralelamente à exclusão de doença coronariana significativa, com nível de evidência I, sendo definitivamente apropriada.

#### 2.15.2. Avaliação de Caracterização Tecidual Miocárdica pela Técnica de Realce Tardio pela Tomografia em Cardiomiopatias Não Isquêmicas

Em pacientes com alguma contraindicação à RM para pesquisa de RT (fibrose), a TC também pode ser realizada com essa finalidade, com uma aquisição tardia das imagens (7-12 min após a injeção do contraste), sem a necessidade de nova injeção de contraste, entretanto com uma segunda exposição à radiação do tomógrafo (considerando uma primeira aquisição dirigida para a avaliação das artérias coronárias). Por outro lado, otimizações recentes dos equipamentos de TC resultaram em baixas doses de radiação por exame. Com essa técnica, é possível avaliar a fibrose miocárdica pela TC, com boa correlação com o RT pela ressonância cardíaca na cardiomiopatia dilatada não isquêmica,^334^ na sarcoidose cardíaca,^335^ na miopericardite^336^ e na endomiocarfibrose.^337^

A avaliação da fibrose miocárdica pela técnica de RT pela TC pode ser utilizada como alternativa à RM nas cardiomiopatias não isquêmicas. A Tabela 21 traz as principais indicações da utilização da TC no contexto das cardiomiopatias não isquêmicas.

**Tabela 21:** Angiotomografia do coração na avaliação das cardiopatias não isquêmicas, doenças do pericárdio e massas cardíacas

Indicações	Classe de recomendação	Nível de evidência
Avaliação da função ventricular esquerda em pacientes com insuficiência cardíaca com imagens inadequadas ou duvidosas por outros métodos não invasivos^25 , 168 , 359^	I	B
Avaliação quantitativa da função ventricular direita como alternativa à ressonância magnética do coração^25 , 168 , 359 – 362^	I	B
Avaliação das artérias coronárias na insuficiência cardíaca para exclusão de doença arterial coronariana (DAC) obstrutiva em pacientes com probabilidade pré-teste baixa ou intermediária^2 , 3 , 25 , 359 , 362^	I	B
Avaliação da morfologia e função do ventrículo direito em pacientes com suspeita de cardiopatia arritmogênica do ventrículo direito como alternativa à ressonância magnética do coração^25 , 359 , 363 , 364^	I	B
Avaliação de pacientes com suspeita de cardiomiopatia hipertrófica com imagens inadequadas ou duvidosas por outros métodos não invasivos^359 , 365 – 367^	I	B
Avaliação de pacientes com suspeita de endomiocardiofibrose com imagens inadequadas ou duvidosas por outros métodos não invasivos^337^	I	C
Avaliação de pacientes com suspeita de miocárdio não compactado/trabeculação excessiva do VE com imagens inadequadas ou duvidosas por outros métodos não invasivos^359 , 366 , 368^	I	C
Avaliação das artérias coronárias para exclusão de DAC obstrutiva em pacientes com suspeita de miocardite aguda e probabilidade pré-teste baixa ou intermediária de DAC^359 , 366 , 369 – 371^	I	C
Avaliação das artérias coronárias em pacientes com suspeita de cardiomiopatia induzida por estresse para exclusão de DAC obstrutiva^359 , 366 , 372 , 373^	IIb	C
Avaliação de fibrose miocárdica (realce tardio pela TC) em pacientes com suspeitas de cardiomiopatias não isquêmicas que não podem realizar ressonância magnética^374^	IIb	B
Avaliação de doenças do pericárdio com imagens inadequadas ou duvidosas por outros métodos não invasivos^25 , 340 , 375 , 376^	IIa	B
Avaliação de massas cardíacas (suspeita de tumor ou trombo) em pacientes que não podem realizar ressonância magnética, para complementação diagnóstica em casos selecionados ou na presença de massas de pequenas dimensões^25 , 346 , 377^	I	C

#### 2.15.3. Avaliação de Volume Extracelular Miocárdico pela Tomografia

Assim como na RM, é possível fazer a avaliação de volume extracelular miocárdico e estimativa de fibrose intersticial pela TC, com aquisição tardia das imagens após contraste. Estudos iniciais demonstraram aumento do volume extracelular em cardiomiopatias não isquêmicas.^338^ Nesse sentido, surge a perspectiva da utilização do volume extracelular pela TC no auxílio diagnóstico das cardiomiopatias e doenças de depósito, bem como guia de resposta a tratamento.

### 2.16. Tomografia na Avaliação das Doenças Pericárdicas

A TC é uma valiosa modalidade de imagem complementar na avaliação do pericárdio, devendo ser considerada em cenários clínicos com apresentação complexa ou achados ecocardiográficos inconclusivos.^339^

A aquisição das imagens sincronizadas com o ECG de forma prospectiva, com baixa radiação e com cobertura da carina ao diafragma é geralmente adequada para a avaliação do pericárdio.^339^ A sincronização com o ECG ajuda na eliminação de artefato de movimento (e, no caso da aquisição retrospectiva, é capaz de fornecer informações funcionais, embora à custa de maior dose de radiação), porém não é um pré-requisito absoluto, visto que imagens razoáveis do pericárdio podem ser obtidas mesmo sem ativação do ECG.

O pericárdio normal aparece como uma estrutura hiperdensa linear delgada medindo habitualmente menos de 2 mm, que é facilmente detectável em exames com e sem contraste em virtude de sua visibilidade contra a baixa atenuação da gordura circundante.

Se etiologias inflamatórias, infecciosas ou neoplásicas forem consideradas, a administração intravenosa de material de contraste iodado é recomendada para aumentar a densidade do sangue e para definir eventual inflamação pericárdica.^339^

#### 2.16.1. Derrame Pericárdico

A TC pode ser útil na determinação da presença de loculações, inflamação pericárdica ou hemorragia.^340^ Um derrame pericárdico pode ser caracterizado pela TC medindo seu nível de atenuação. Atenuação próxima à da água (< 10 unidades Hounsfield [HU]) sugere um derrame transudativo simples. É incomum para um derrame transudativo exceder 15 HU, e isso pode ser usado como uma medida de limiar para considerar o derrame exsudativo mais provável do que o transudativo.^340^ Se a atenuação tomográfica estiver na faixa de 20 a 60 HU, o derrame pericárdico pode ser purulento, maligno ou mixedematoso. Derrames com valores de atenuação > 60 HU podem ainda sugerir hemorragia.

#### 2.16.2. Pericardite Aguda

A TC pode evidenciar a presença de espessamento pericárdico e realce do pericárdico após a administração do meio de contraste.

Ela pode ser de grande valia especialmente na pericardite de etiologia traumática (particularmente quando houver suspeita de lesões associadas em estruturas adjacentes), doenças neoplásicas (avaliação da extensão e estadiamento da doença) e também no pós-IAM (quando persistem dúvidas quanto à possibilidade de hemopericárdio secundário à ruptura da parede livre).

#### 2.16.3. Tamponamento Pericárdico

A TC não tem nenhum papel no tamponamento cardíaco agudo, tendo em vista a instabilidade do paciente, mas pode ajudar a determinar a viabilidade da pericardiocentese percutânea, especialmente em derrames loculados ou complexos, quando o tamponamento cardíaco é subagudo.^341 , 342^

Os sinais de tamponamento cardíaco na TC incluem um "coração achatado" e/ou um arqueamento septal devido à compressão das câmaras cardíacas secundária à presença de fluido, ar ou massas. Os achados indiretos incluem a dilatação da veia cava superior (VCS), com diâmetro semelhante ou maior que o da aorta torácica adjacente, dilatação da veia cava inferior (VCI), com diâmetro maior que duas vezes o da aorta abdominal adjacente, edema periportal, refluxo de material de contraste para a VCI ou veia ázigos e aumento das veias hepáticas e renais.^340^

#### 2.16.4. Pericardite Constritiva

A TC é um excelente método na avaliação da espessura do pericárdio e, portanto, desempenha um papel importante na pericardite constritiva. O pericárdio normal tem 1 a 2 mm de espessura pela TC, enquanto, na pericardite constritiva, costuma ter de 4 a 20 mm.^342 , 343^ O aumento da espessura do pericárdio entretanto é sinal de suporte em casos de suspeita clínica, mas não prova a condição, visto que há pericardite constritiva sem espessamento pericárdico e espessamento que não determina constrição.^341^ A TC é o melhor método para delinear a presença e a extensão da calcificação pericárdica, achado significativo diante da suspeita clínica, embora não patognomônico.^344 , 345^

A aquisição de imagens sincronizadas ao ECG e com controle retrospectivo permite avaliar a fisiologia e eventual colabamento das câmaras cardíacas, entretanto, tendo em vista a maior exposição à radiação ionizante, permanece como uma indicação específica para pacientes com ecocardiograma limitado e contraindicações para RMC.^345^

#### 2.16.5. Tumores Pericárdicos

A TC permite uma melhor caracterização das lesões pericárdicas tumorais, bem como das estruturas adjacentes, investigando a eventual disseminação tumoral e a presença de calcificação e linfadenopatias.^340 , 341^ A TC é mais robusta que a RMC na identificação de outras lesões torácicas, incluindo câncer de pulmão primário, metástases pulmonares e nódulos mediastinais.^342^

As características de malignidade pericárdica na TC incluem um pericárdio irregular, espessado e nodular, uma efusão pericárdica complexa e o realce pericárdico após infusão de contraste. Ruptura do saco pericárdico, presença de derrame hemorrágico, invasão do tecido adiposo epicárdico, do miocárdio ou das câmaras cardíacas e adenopatia mediastinal são características de malignidade de natureza agressiva.^342^

Tumores benignos do pericárdio incluem lipomas, que demonstram baixa atenuação na TC^340^, e teratomas, que se apresentam como massas contendo gordura e cálcio.^342^

O mesotelioma pericárdico é a doença maligna primária mais comum do pericárdio e pode se apresentar como um derrame com nódulos ou placas pericárdicas na TC ou como uma massa com realce heterogêneo.^340^ Linfoma, sarcoma e lipossarcoma aparecem como grandes massas heterogêneas com derrame pericárdico associado. Os linfomas pericárdicos se manifestam como massas com realce infiltrativo.^340^

Angiossarcomas e sarcomas sinoviais são tumores altamente vasculares que frequentemente se apresentam com hemopericárdio maciço. Na TC, esses tumores se manifestam como massas necróticas.^340^

#### 2.16.6. Cistos e Divertículos

Os cistos, normalmente situados no ângulo cardiofrênico direito, são visualizados na TC habitualmente como massas ovais e homogêneas com parede finas e com densidade de 30-40 HU e, dado seu componente líquido, não mostram nenhum realce com o contraste.^341^ O aparecimento de um divertículo pericárdico é semelhante a um cisto na TC, embora uma comunicação aberta com o saco pericárdico seja identificada.^340^

#### 2.16.7. Agenesia Congênita do Pericárdio

A TC muitas vezes diagnostica a ausência congênita do pericárdio de forma incidental. Por causa do contraste natural entre o pericárdio e a borda adiposa epicárdica, normalmente é possível a delimitação do pericárdio em TC sem contraste, exceto nos casos de pacientes com gordura epicárdica mínima, em que essa diferenciação se torna difícil.^342^

Além da visualização direta do pericárdio, existem importantes sinais morfológicos e funcionais indiretos consistentes com defeitos pericárdicos. O diagnóstico é suspeitado quando o movimento da parede posterior é exagerado ou quando o ventrículo direito (VD) parece falsamente aumentado devido ao desvio para a esquerda. O extremo deslocamento do ápice para a axila leva a uma aparência comprimida dos átrios. A interposição do tecido pulmonar entre a aorta e a artéria pulmonar ou entre a base do coração e o diafragma é um sinal específico.

#### 2.16.8. Pneumopericárdio

O pneumopericárdio é um acúmulo de ar dentro do espaço pericárdico, que geralmente ocorre no contexto de trauma e lesão pericárdica. Outras etiologias incluem ventilação com pressão positiva e cirurgia cardiotorácica.^340^

A TC é muito útil no quadro agudo e irá demonstrar o pneumopericárdio como acúmulo de ar no espaço pericárdico, podendo ainda avaliar sua repercussão hemodinâmica quando adquirida de forma sincronizada retrospectivamente com o ECG.^340^

#### 2.16.9. Corpos Estranhos Pericárdicos

A lesão no pericárdio por corpo estranho pode ocorrer devido a trauma direto ou secundário à embolização a partir de um local de penetração distal. A TC geralmente é diagnóstica por localizar o corpo estranho no pericárdio e também pode mostrar características associadas, como hemopericárdio.^340^ A Tabela 19 apresenta a performance diagnóstica da TC e da ressonância nas alterações do pericárdio. A Tabela 22 traz as principais indicações da utilização da TC na avaliação do pericárdio.

**Tabela 19:** Comparação da TC e RMC nas doenças pericárdicas

	TC	RMC
**Aspectos técnicos**		
Disponibilidade	++	+
Custo	Moderado	Alto
Duração do exame	10 minutos	30-40 minutos
Segurança	+ a	++ b
Acesso e monitoramento do paciente	++	+/-
**Pericárdio**		
Espessamento pericárdico	+++	+++
Calcificação pericárdica	+++	-
Inflamação pericárdica	++	+++
Aderências pericárdicas/mobilidade dos folhetos	+	+++
Detecção da efusão	+++	+++
Caracterização da efusão	++	++
Massas pericárdicas	+/++	++/+++
**Morfologia pericárdica**		
Incluindo caracterização tecidual	++	+++
**Função cardíaca**		
Sistólica	++ c	+++
Diastólica	-	++
Movimentação septal/acoplamento	+/-	+++
Mudanças intracardíacas com a respiração	+/-	++

RMC: ressonância magnética cardíaca; TC: tomografia computadorizada. (-): não possível ou pobre avaliação (+): moderado (++) bom (+++) excelente.

a radiação ionizante, potencial nefrotoxicidade pelo meio de contraste, reações alérgicas ao contraste.

b pacientes com implantes metálicos, claustrofobia, reações alérgicas ao contraste, restrita apenas a pacientes hemodinamicamente estáveis.

c usando aquisição de imagens com sincronização ao eletrocardiograma.

**Tabela 22:** Angiotomografia na avaliação das doenças vasculares

Indicações	Classe de recomendação	Nível de evidência
Avaliação de tromboembolismo pulmonar^2 , 393^	I	A
Avaliação de aneurismas da aorta^2 , 179 , 401 – 403^	I	B
Avaliação de dissecção crônica da aorta^2 , 403^	I	B
Avaliação de síndromes aórticas agudas (dissecção, úlcera, hematoma e ruptura)^2 , 401 , 404 – 406^	I	B
Avaliação de lesão traumática da aorta^2 , 401 , 406^	I	B
Planejamento de abordagem cirúrgica da aorta^2 , 401^	I	B
Planejamento de abordagem endovascular da aorta^2 , 401^	I	B
Avaliação pós-implante de endopróteses aórticas^2 , 401 , 407^	I	B
Avaliação das artérias carótidas e vertebrais^2 , 408^	I	B
Avaliação do tronco celíaco e das artérias mesentéricas^2 , 400 , 409^	I	B
Avaliação das artérias renais^2 , 84 , 409^	I	B
Avaliação das artérias dos membros superiores e inferiores^2 , 409 – 412^	I	B
Avaliação das arterites de grandes e médios vasos^2 , 385 , 400 , 401 , 407 – 412^	I	B
Avaliação venosa central^2 , 413 , 414^	I	B
Avaliação venosa periférica (membros)^2 , 414 – 417^	IIa	B

### 2.17. Tomografia na Avaliação de Massas/Trombos Cardíacos

A avaliação de tumores cardíacos geralmente requer a utilização de várias modalidades de imagem para se obter informações precisas sobre a localização e as características teciduais. A ecocardiografia transtorácica (ETT) é geralmente a primeira escolha devido à sua disponibilidade e alta resolução temporal, sendo ideal para identificar pequenas massas móveis. Já a ecocardiografia transesofágica (ETE) é mais acurada na avaliação de massas valvares que não são bem visualizadas na ETT.^346^

A imagem por RMC é a modalidade de escolha para uma avaliação mais detalhada de tumores cardíacos não valvares, pois oferece excelente caracterização tecidual e avaliação multiplanar das estruturas cardíacas. No entanto, a RMC pode não ser adequada em alguns pacientes devido a restrições como o tempo de aquisição prolongado, contraindicações em casos de claustrofobia, pacientes não colaborativos ou na presença de certos dispositivos implantados.^347^

Nesses cenários em que outras modalidades de imagem não são diagnósticas ou contraindicadas, a TC tem se tornado um método cada vez mais utilizada para essa avaliação. O exame tem a vantagem de um tempo de aquisição mais curto e alta resolução espacial.^348^ A sincronização eletrocardiográfica minimiza artefatos relacionados ao movimento e permite a identificação da localização do tumor, a delimitação mais precisa das margens da lesão e sua relação com os planos teciduais e estruturas circundantes, o que é especialmente valioso para o planejamento cirúrgico.^349^

A avaliação diagnóstica de massas baseada na TC envolve vários aspectos, incluindo o tamanho da massa, a localização (câmara cardíaca, envolvimento pericárdico, estruturas extracardíacas), a quantidade, a morfologia (fixação, aparência das margens, infiltração) e a correlação clínica (malignidade ou infecção conhecida, presença de cateter, síndromes associadas). Além disso, a TC tem a capacidade de caracterizar tecidos por meio da análise de densidade e perfusão, sendo útil no diagnóstico etiológico diferencial por meio da avaliação de calcificação, atenuação de gordura, distribuição vascular e componente fibroso dos tumores. Comparada a outras modalidades de imagem cardíaca, a TC destaca-se como a opção ideal para avaliar massas calcificadas, oferecendo também uma avaliação abrangente do tórax, tecido pulmonar e suas estruturas vasculares correspondentes ( Tabela 20 ).^346^

**Tabela 20:** Características das massas cardíacas pela tomografia computadorizada (TC)

MASSA/TUMOR	ACHADOS DE TC CARDÍACA
BENIGNOS 75%	
Mixoma	Pedunculado, móvel, heterogêneo com baixa atenuação. 10-20% são calcificados. Podem prolapsar através da válvula mitral.
Lipoma	Bem definido, encapsulado e hipodenso com atenuação de gordura. São homogêneos e sem realce; lesões múltiplas podem ser vistas com esclerose tuberosa.
Fibroelastoma	Difícil observação pela TC. Pequena massa homogênea aderida à valva cardíaca (10 mm), por meio de um pequeno pedículo, móvel. Podem ser formados trombos em sua superfície.
Rabdomioma	Múltiplas lesões em 60% dos casos. Atenuação homogênea semelhante ao miocárdio. > 90% em lactentes e crianças.
Fibroma	Homogêneo e intramural, com baixa atenuação e realce mínimo, geralmente com calcificação central; segundo mais comum em lactentes e crianças.
Hemangioma	Bem definido; densidade baixa ou igual ao miocárdio; realce intenso heterogêneo; "rubor vascular".
teratoma	Multicístico, realce moderado, parcialmente calcificado.
**MALIGNOS 25%**	
Angiossarcoma	Irregular, heterogêneo, baixa atenuação, infiltrativo, derrame pericárdico, metastático.
Rabdomiossarcoma	Irregular, baixa atenuação, infiltrativo; a extensão importante para miocárdio e pericárdio associa-se a mau prognostico. Comum em lactentes e crianças.
Fibrossarcoma	Grande, irregular, baixa atenuação, com extensa área de necrose central ou hemorragia, infiltrativo.
Osteossarcoma	Baixa atenuação, infiltrativo, com calcificação extensa.
Lipossarcoma	Grande, atenuação de tecido adiposo e mole, leve realce de contraste, infiltrativo.
Mesotelioma	Infiltrativo, atenuação variável, derrame pericárdico.

A TC desempenha ainda um papel crucial no estadiamento de tumores, pois possui a capacidade de detectar metástases em casos de suspeita de malignidade, especialmente quando combinada com tomografia por emissão de pósitrons (PET)/TC com 18F-fluorodesoxiglicose.^350 – 352^

O método também pode auxiliar na diferenciação entre tumores intracavitários e trombos.^353^ Demonstrou ser uma alternativa precisa e confiável ao ETE para a detecção de trombos no átrio esquerdo (AE) e apêndice atrial esquerdo (AAE) em pacientes com fibrilação atrial, com sensibilidade e especificidade médias de 96 e 92%, respectivamente.^10^ Da mesma forma, em pacientes com AVCi, a sensibilidade e a especificidade da TC para a detecção de trombos no AE/AAE são de 96 e 100%, respectivamente.^354^

A TC caracteriza trombos no ventrículo esquerdo como massas hipodensas, com atenuação significativamente menor em relação ao miocárdio adjacente, e os diagnostica com sensibilidade, especificidade e valores preditivos positivos e negativos de 94, 97, 94 e 97%, respectivamente.^353^ Atualmente, existem poucos dados validados sobre o papel da TC na detecção de trombos no ventrículo esquerdo em comparação com à RMC;^355^ no entanto, no contexto do AVCi, a TC pode ser superior à ETT.^356^

A angio-TC das artérias coronárias obtida no mesmo protocolo de aquisição possibilita a avaliação pré-cirúrgica da presença de DAC ou massas adjacentes que possam determinar obstrução, reduzindo riscos relacionados ao procedimento.^357^ A estratificação não invasiva é especialmente indicada para pacientes com baixa probabilidade pré-teste, principalmente naqueles com massas em cavidades cardíacas esquerdas, que apresentam maior risco de eventos embólicos relacionados à angio-TC coronariana invasiva.^357 , 358^

A Tabela 20 apresenta as características das principais massas cardíacas observadas pela TC.

A Tabela 21 traz as recomendações da utilização da tomografia na avaliação das cardiopatias não isquêmicas, doenças do pericárdio e massas cardíacas/trombos.

### 2.18. Doenças Vasculares

A avaliação dos territórios vasculares arteriais e venosos tem na TC um exame prático e preciso, com acurácia diagnóstica extremamente elevada. Melhorias dos equipamentos de TC, com aumento do número e do perfil de detectores, trouxeram avanços em resolução espacial e temporal, permitindo a realização de exames com maior rapidez e efetividade. Tais características são fundamentais, por exemplo, na avaliação de pacientes com condições críticas, em que o tempo de realização do exame passa a ser fator determinante.

As patologias de diferentes territórios vasculares extracardíacos serão abordadas a seguir. Esta Diretriz não tratará do diagnóstico das patologias vasculares intracranianas.

#### 2.18.1. Aorta

As doenças da aorta são diversas e incluem os aneurismas da aorta (AA), a síndrome aórtica aguda (SAA), os pseudoaneurismas, a rotura aórtica, as afecções ateroscleróticas e inflamatórias, as doenças genéticas e as anormalidades congênitas. Compõem a SAA a dissecção da aorta (DA), o hematoma intramural (HIM), a úlcera penetrante (UP) e as lesões traumáticas da aorta.

As doenças da aorta podem ser diagnosticadas após um longo período de desenvolvimento subclínico ou podem ter apresentação aguda. A SAA costuma ser o primeiro sinal da doença, que requer diagnóstico rápido e tomada de decisão para reduzir o prognóstico extremamente ruim. Os resultados do tratamento para condições estáveis, frequentemente assintomáticas, mas de alto risco, são muito melhores do que os resultados do tratamento necessário para apresentações de doenças agudas.

Dessa forma, a identificação das doenças da aorta antes de eventual fase aguda é desejável, e, nesse contexto, o diagnóstico por imagem exerce papel muito importante, especialmente a TC e a RM.

A TC desempenha papel central no diagnóstico, estratificação de risco e tratamento das doenças da aorta. Suas vantagens sobre outras modalidades de imagem incluem o curto tempo necessário para aquisição e processamento de imagens, a capacidade de obter um conjunto de dados volumétricos (3D) completo de toda a aorta, amplo campo de visão e ampla disponibilidade.

Na avaliação da aorta torácica, a aquisição de imagens sincronizadas ao eletrocardiograma são fundamentais para eliminar artefatos de movimento da raiz aórtica e da aorta ascendente, evitando erros diagnósticos relacionados.^378^

Para a obtenção de imagens volumétricas que permitam reconstruções multiplanares e tridimensionais, fundamentais na avaliação da aorta, faz-se necessário a utilização de tomografia helicoidal multidetectores (TCMD) de pelo menos 16 canais, com preferencialmente 64 canais ou mais, devido a esses equipamentos terem resolução espacial e resolução temporal mais altas.^379^ Na avaliação da aorta torácica, pela necessidade de sincronização com o ECG, é recomendado a utilização de TCMD de pelo menos 64 canais, pois a aquisição mais rápida se torna menos suscetível a artefatos de movimentação cardíaca e respiratória. Recomenda-se espessura de corte de 1 mm ou menos.

Para a obtenção de imagens angiográficas por angio-TC, é necessário o uso de contraste iodado intravenoso, com aquisição das imagens no pico da contrastação arterial. Nos quadros agudos e pós-operatórios, é recomendada a aquisição de imagens sem contraste antes da fase contrastada (fase pré-contraste), para auxiliar a detecção de hematomas e extravasamentos. Uma aquisição tardia pós-contraste é recomendada nos controles pós-operatórios, especialmente endovasculares, para a detecção de extravasamentos e na avaliação de eventuais realces parietais.

A TC permite detectar com excelente acurácia a localização e a extensão do segmento aórtico doente, os diâmetros do vaso e a presença de ateroma, trombo, dilatação, estenose, hematoma, ulceração, dissecção, espessamento parietal, calcificação e extravasamento. Permite avaliar os tecidos periaórticos, os ramos da aorta e as estruturas extravasculares, possibilitando detectar alterações em órgãos-alvo como hipoperfusão e infarto. Além disso, pode-se facilmente incluir na área de varredura os ramos do arco aórtico, as artérias ilíacas e as artérias femorais, fundamentais no planejamento de procedimentos de reparo cirúrgico e endovascular.

Nas doenças agudas da aorta, a TCMD é o método de imagem recomendado na avaliação inicial. Vários estudos demonstraram alta acurácia diagnóstica para detecção de DA e HIM (sensibilidade combinada de 100% e especificada combinada de 98%), assim como para a detecção de UP, trombo, oclusão, pseudoaneurisma e rotura.^380^

Em virtude da utilização de radiação ionizante, recomenda-se que, em crianças e mulheres jovens, o controle das doenças da aorta não seja feito exclusivamente com TC, podendo-se utilizar de outros métodos que não utilizam radiação ionizante, como a RM, o ultrassom e o ecocardiograma, a depender da localização do segmento aórtico comprometido.

A utilização de contraste iodado pode ser uma limitação nos pacientes com alergia ao iodo e naqueles com função renal comprometida.^381^ Destaca-se que o controle de dilatação da aorta pode ser feito por TC sem contraste, pois a é possível fazer a mensuração do calibre externo do vaso sem a administração de contraste, com a mesma precisão de um estudo com contraste.

#### 2.18.2. Carótidas Extracranianas

A angio-TC de carótidas é um método de imagem eficaz para a avaliação de acometimento aterosclerótico desse território vascular e sobretudo para definição de graus de obstrução decorrentes, fornecendo subsídios para o planejamento de tratamento/intervenção de doenças extravasculares.

A estenose carotídea é definida por uma estenose > 50% (sintomática ou assintomática) no segmento extracraniano da carótida interna e é uma das principais indicações clínicas para a triagem de doença arterial extracraniana por exames de imagem.^382^ Pacientes que apresentarem estenose carotídea ≥ 60 a 99% em exames de ultrassom têm indicação de realizar angio-TC ou angioressonância de carótidas para confirmação do grau de estenose e para avaliação das características das placas. Essa recomendação justifica-se pela menor acurácia do ultrassom para quantificação do grau de estenose e pela possibilidade de falsos-positivos por aquele método.^383^

A angio-TC de carótidas pode ser empregada na avaliação de dissecção, que é uma reconhecida causa de AVEi e ataque isquêmico transitório (AIT).^384^ A dissecção de carótidas pode ser espontânea, eventualmente na presença de fatores predisponentes, porém a dissecção espontânea de artérias extracranianas pode também ocorrer em pacientes sem fatores predisponentes, em associação a traumas.^384^

Traumatismos cranianos ou cervicais apresentam moderado a alto risco de lesões vasculares associadas e que exigem avaliação por meio de estudo angiotomográfico, especialmente traumatismos associados a fraturas de primeira a terceira vértebras cervicais, fraturas acometendo os forames transversos e fraturas da base do crânio. Outras indicações de angio-TC de carótidas incluem displasia fibromuscular de carótidas ou vertebrais, carotidínea, avaliação de aneurismas e pseudoaneurismas, malformações vasculares e fístulas arteriovenosas, planejamento de tratamento endovascular ou cirurgia vascular, avaliação de vascularização de tumor na região cervical ou craniocervical, vasculites e doenças do colágeno.

#### 2.18.3. Artérias Renais

A angio-TC tem grande utilidade na avaliação de estenoses das artérias renais e tem vantagens em comparação à arteriografia com subtração digital por sua facilidade de realização e menor invasividade.^385^ Em estudo prospectivo comparando a angio-TC com a arteriografia com subtração digital, os valores de sensibilidade, especificidade e acurácia para detecção de estenoses significativas foram de 100, 98,6 e 96,9%, respectivamente.^386^ Além da aterosclerose, a TC pode avaliar o acometimento das artérias renais por outras doenças, como a displasia fibromuscular, poliarterite nodosa, fístulas arteriovenosas, aneurismas e tromboses.^387^

#### 2.18.4. Doença Vascular Periférica

Aproximadamente 80% das doenças das artérias dos membros inferiores são representadas por doença arterial obstrutiva periférica (DAOP), com uma prevalência estimada em 14,5% em indivíduos com idade acima de 70 anos. A apresentação clínica pode variar desde pacientes assintomáticos, portadores de claudicação intermitente e eventualmente com quadros de oclusão arterial aguda.

Os 20% restantes das doenças arteriais dos membros inferiores, que também frequentemente causam estenoses e oclusões, compreendem doenças sistêmicas e processos inflamatórios e degenerativos locais. Nesse grupo, estão incluídos os aneurismas, as vasculites (como trombangeíte obliterante, displasia fibromuscular e arterite de Takayasu) e a síndrome do aprisionamento poplíteo.

Dentro desse contexto, os exames de imagem das artérias dos membros inferiores têm o papel de confirmar uma hipótese diagnóstica (como de estenoses e/ou oclusões por DAOP) e de retratar a anatomia e as alterações a fim de verificar a necessidade e a viabilidade de um procedimento invasivo, de escolher a melhor estratégia e de se preparar para esse procedimento, seja endovascular ou cirúrgico.

A angio-TC e a angiografia por RM (angio-RM) permitem a obtenção de imagens em alta resolução das artérias dos membros inferiores, com um tempo de execução relativamente mais curto, o que leva a uma maior tolerância dos pacientes.^388^ O desempenho da angio-RM para a avaliação da DAOP é muito semelhante ao da angio-TC, com sensibilidade de 92 a 99,5% e especificidade de 64 a 99%.^388 , 389^ Em virtude da disponibilidade, facilidade de execução e tempo reduzido de exame, a angio-TC é o exame de imagem não invasivo de escolha para a avaliação vascular, ficando a angio-RM reservada a pacientes que não podem ser submetidos à angio-TC, como aqueles alérgicos a iodo e com quadros de insuficiência renal.

A angio-TC e a angio-RM permitem o mapeamento completo das artérias dos membros inferiores, desde a aorta abdominal até as artérias dos pés. Garantem a identificação do número de lesões, extensão, diâmetro e morfologia das estenoses, calibre arterial normal adjacente e condições dos vasos distais.^389^ A angio-TC demonstra, ainda, calcificações.^388 , 390^ Essas informações orientam o planejamento do procedimento em relação à via de acesso, à escolha do material e à permeabilidade a longo prazo esperada após a intervenção.^388 , 390^

As próteses endovasculares e os *stents* são facilmente identificados aos exames de angio-TC pela sua malha metálica, que apresenta atenuação elevada.^391^ Sua perviedade e eventuais estenoses luminais também são muito bem caracterizadas, uma vez que esse método permite a visualização da coluna de contraste intraluminal.^391^ As próteses endovasculares e os *stents* em geral causam artefatos de distorção da imagem aos exames de RM e angio-RM, o que impede a avaliação da sua luz e, em alguns casos, das estruturas circundantes.

Os aneurismas das artérias periféricas apresentam-se como dilatações arteriais focais fusiformes ou saculares, frequentemente associadas a trombos murais.^392^ Na avaliação angiográfica, seja por tomografia ou ressonância, é fundamental a avaliação dos diâmetros, das extensão dos aneurismas e dos diâmetros do vaso acometido acima e abaixo da dilatação,^392^ medidas do colo do aneurisma,^392^ bem como a sua localização precisa, com descrição do eventual acometimento de outros vasos^392^ para a escolha da melhor abordagem.

#### 2.18.5. Artérias Pulmonares

Diversas patologias podem envolver as artérias pulmonares e resultar em alterações parietais, dilatação, estenose e oclusão. As duas principais patologias vasculares pulmonares são o tromboembolismo pulmonar (TEP) e a hipertensão pulmonar (HP).

A TC desempenha papel central no diagnóstico das doenças das artérias pulmonares, especialmente o TEP. Suas vantagens sobre outras modalidades de imagem incluem o curto tempo necessário para aquisição e processamento de imagens, a capacidade de obter um conjunto de dados volumétricos (3D) completo de todo o tórax, o amplo campo de visão e a ampla disponibilidade. Para avaliação das artérias pulmonares, recomenda-se a utilização de TCMD de ao menos 16 canais, preferencialmente de 64 canais ou mais, devido a esses equipamentos terem resolução espacial e resolução temporal mais altas, aumentando a qualidade das imagens e reduzindo a ocorrência de artefatos de movimentação cardíaca e respiratória. Recomenda-se espessura de corte de 1 mm ou menos.

A angio-TC de artérias pulmonares é o método de escolha para avaliação de TEP. Permite adequada visualização das artérias pulmonares e identificação de trombos até o nível subsegmentar. Ela também apresenta boa sensibilidade e especificidade para o diagnóstico de TEP,^393^ sendo considerada uma angio-TC negativa um critério adequado para exclusão de TEP em pacientes com probabilidade clínica baixa ou intermediária.^394^

Com relação à HP, a angio-TC pode identificar alterações cardíacas e extracardíacas usualmente relacionadas a HP, como dilatação do tronco pulmonar, sinais de TEP pregresso, dilatação ou hipertrofia ventricular direita, entre outros.^381 , 395^

#### 2.18.6. Artérias Viscerais

A angio-TC com contraste é eficaz para avaliação de estenoses arteriais e venosas significativas nos quadros de isquemia mesentérica aguda e crônica, bem como fornece informações adicionais das alças intestinais, inclusive para avaliação de isquemia mesentérica por alças estranguladas em hérnias internas, aderências ou bridas e também de doenças não ateroscleróticas, como arterites e displasia fibromuscular.^396 – 398^

Na isquemia mesentérica aguda, a angio-TC com contraste é o exame de escolha, pois existe a necessidade de diagnóstico rápido e, além disso, outros exames como a angio-RM, por exemplo, discutida na sessão de RM desta publicação, pode não estar disponível ou depender de mais cooperação do paciente para aquisição das sequências. Uma criteriosa revisão sistemática e metanálise calcula sensibilidade de 94% e especificidade de 95% para o diagnóstico de isquemia mesentérica aguda pela angio-TC com contraste.^397^

Na isquemia mesentérica crônica, também chamada de angina abdominal, a angio-TC com contraste pode identificar tanto as estenoses antigas, como eventuais redes colaterais vasculares que podem aparecer após estenose significativa/oclusão crônica dos principais vasos mesentéricos (tronco celíaco, artéria mesentérica superior e artéria mesentérica inferior).^399^

A TCMD pode avaliar os principais vasos mesentéricos com acurácia semelhante à da arteriografia. Além disso, o método também possui elevada acurácia para avaliação pós-operatória de angioplastias ou enxertos.^390 , 400^

Na Tabela 22 , encontram-se as recomendações de uso da angio-TC na avaliação de diferentes cenários clínicos relacionados às doenças vasculares.

## 3. Ressonância Magnética Cardiovascular

A RMC é um dos exames mais completos e abrangentes da cardiologia e, apesar de existente há décadas nessa área, apresentou crescimento exponencial do seu uso e das suas indicações após introdução da técnica do realce tardio (RT) em 1999, tornando-se um exame indispensável nos tempos atuais para os melhores cuidados aos pacientes com cardiopatias.^2 , 418 – 420^ Os estudos científicos decorrentes desde então mudaram o entendimento de várias patologias dentro da cardiologia, seja na DAC por meio da mais sensível detecção do infarto do miocárdio e de um novo conceito de viabilidade miocárdica pela transmuralidade do infarto ou pela avaliação das cardiomiopatias não isquêmicas, com o conhecimento de doenças antes não diagnosticadas de maneira adequada. Adicionalmente, a avaliação de cardiopatias congênitas encontrou na RMC maior precisão das medições volumétricas e das funções ventriculares, ajudando a melhorar os resultados no seguimento desses pacientes.^2 , 12 – 15 , 421 – 423^

A RMC é um exame que não utiliza radiação ionizante (ao contrário da TC, da medicina nuclear e da hemodinâmica), apresentando segurança para pacientes que precisam de seguimento com exames de imagem ou de pacientes com menor faixa etária. Quando se necessita do uso de contraste, são utilizados contrastes baseados em gadolínio, que não apresentam nefrotoxicidade, mas devem ser usados com cautela nos pacientes com insuficiência renal crônica e taxa de filtração glomerular < 30 mL/min pelo risco de fibrose sistêmica nefrogênica. Apesar dessa recomendação, consensos recentes mostraram a segurança de contrastes específicos nessa população.^2 , 424^

Limitações ao uso da RMC são a claustrofobia, que pode ser contornada realizando o exame sob anestesia nos pacientes em que o benefício da informação superam os riscos do procedimento, e a presença de dispositivos metálicos como clipes cerebrais com material ferromagnético e implantes cocleares.^2^ A presença de dispositivos cardíacos eletrônicos implantáveis como o marca-passo e o cardiodesfibrilador implantável (CDI) não são contraindicações ao exame de RM na atualidade. Entretanto, tais dispositivos necessitam de programação e acompanhamento por especialistas durante a realização do exame e irão apresentar artefatos metálicos no tórax, algumas vezes limitando a análise apropriada do exame, particularmente nos portadores de CDI.^2 , 425^

Uma grande vantagem desse método é a sua multimodalidade, permitindo obter, em um único exame, diversos dados de uma cardiopatia, apresentando um diagnóstico mais assertivo por meio de várias sequências de pulsos com diferentes objetivos.^2^

Uma técnica usada de rotina é a cinerressonância através da sequência de pulso de precessão livre em estado de equilíbrio (SSFP, de *steady state free precession* ), que permite a mensuração acurada e com alta reprodutibilidade dos volumes das câmaras cardíacas e das massas e funções ventriculares, sem a necessidade do uso de contraste, sendo o melhor método para pacientes que precisam dessa informação para a tomada de decisões clínicas. As sequências de cinerressonância também permitem uma excelente avaliação morfológica e funcional do coração sem a limitação pelo biotipo dos pacientes, não havendo problemas de janelas inadequadas e se podendo avaliar hipertrofias localizadas, trabeculações, aneurismas/pseudoaneurismas, doenças do pericárdio, massas e valvopatias, inclusive realizar medidas por planimetria para determinar a gravidade das estenoses valvares.

Outra sequência de pulso da RMC é o *fast spin echo* , capaz de realizar imagens estáticas do coração sem o uso de contraste, podendo ser pesadas em T1 para a avaliação morfológica e a determinação de gordura ou pesadas em T2 para a avaliação de edema.^2^ Essas sequências podem ser usadas na avaliação de massas e doenças do pericárdio, avaliação de edemas miocárdico e pericárdico e avaliação da infiltração gordurosa nas cardiomiopatias arritmogênicas e da metaplasia lipomatosa no infarto crônico, por exemplo.

Uma análise específica da RM que mudou o seguimento, o tratamento e a mortalidade da talassemia *major* foi o diagnóstico da sobrecarga de ferro miocárdico por meio da alteração do valor do T2* (lê-se T2 estrela).^2 , 426 , 427^ Essa medida é realizada por uma sequência de pulso *gradient echo* , sem o uso de contraste, e ajuda no diagnóstico da hemossiderose cardíaca, que é uma complicação das anemias hemolíticas hereditárias que recebem múltiplas transfusões como a talassemia *major* e a hemocromatose hereditária.

Recentes técnicas dentro da RMC são os mapas paramétricos T1 e T2, cada vez mais empregados na rotina clínica, porém ainda não disponíveis em todos os aparelhos de RM em nosso país.^428 , 429^ O mapa T1 permite detectar e quantificar alterações na estrutura miocárdica sem o uso de contraste, como a presença da fibrose miocárdica; entretanto, pode ter limitações para determinar a sua etiologia quando não associado à técnica do RT. O mapa T1 também está alterado nas situações de inflamação como no infarto agudo, na miocardite aguda e na pericardite e, quando há suspeita de doenças de depósito, como a amiloidose cardíaca, é considerado fundamental no seu diagnóstico pela RM. O mapa T2 permite a avaliação do edema miocárdico de forma mais objetiva e acurada, auxiliando no diagnóstico das cardiopatias agudas, como a miocardite.

O *phase contrast* é uma sequência de pulso amplamente utilizada nos pacientes com valvopatias e cardiopatias congênitas, pois possibilita a análise de fluxos com a mensuração dos volumes e das velocidades associadas, sem o uso de contraste.^2^ O *phase contrast* pode fornecer medidas altamente precisas do volume regurgitante e da fração regurgitante na graduação de uma insuficiência valvar ou da relação Qp/Qs (fluxo pulmonar/fluxo sistêmico) nos pacientes com *shunts* intra ou extracardíacos.

Outra técnica importante da RMC é a perfusão de primeira passagem do contraste baseado em gadolínio, que pode ser usada na caracterização tecidual de massas cardíacas ou, principalmente, na análise da perfusão miocárdica sob estresse farmacológico para a avaliação de isquemia. O estresse farmacológico nos exames de RM é geralmente realizado com vasodilatadores como a adenosina e o dipiridamol e possuem elevada acurácia diagnóstica. No estudo CE-MARC, que comparou a RMC e a cintilografia para avaliação de isquemia em 628 pacientes submetidos à cinecoronariografia, a RMC mostrou maior sensibilidade (RMC 86,5% vs. SPECT 66,5%) e maior VPN (RMC 90,5% vs. SPECT 79,1%) do que a cintilografia, com especificidade e valor preditivo positivo semelhantes.^10^ Outra forma de detectar isquemia miocárdica com a RMC é a análise da contratilidade segmentar sob estresse com dobutamina, também apresentando alta acurácia diagnóstica. Em estudo comparando a RMC com ecocardiografia sob estresse com dobutamina, observou-se maior sensibilidade (RMC 86,2% vs. ECO 74,3%), bem como maior especificidade (RMC 85,7% vs. ECO 69,8%) e acurácia (RMC 86% vs. ECO 72,7%) da RMC para detecção de estenoses coronárias significativas quando comparada à cinecoronariografia.^430^

**Figura 4:**
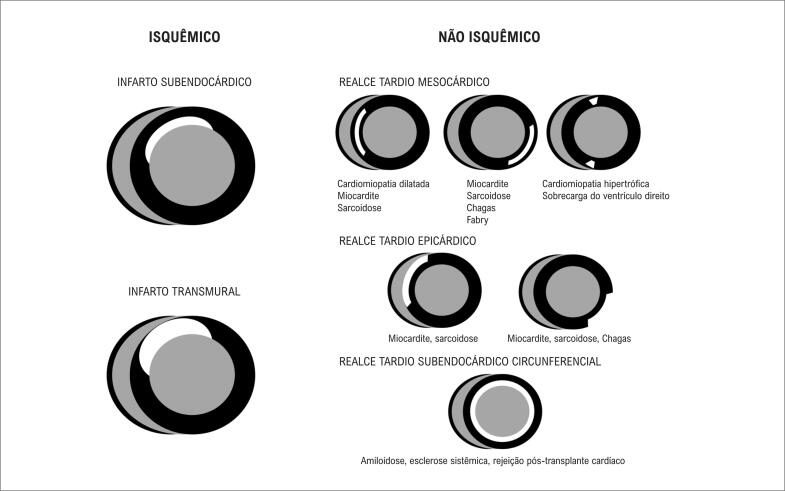
Padrões de fibrose miocárdica identificados pela ressonância magnética e correlação com as diferentes cardiomiopatias.

A técnica de RT, primeiramente demonstrada em pacientes com infarto e posteriormente em diferentes cardiomiopatias não isquêmicas, causou grande impacto na cardiologia e no uso da RMC. Essa técnica permite detectar áreas de necrose miocárdica após a injeção de contraste, qualificando-as entre o infarto e a fibrose não isquêmica. Além disso, permite predizer a viabilidade miocárdica pela transmuralidade do infarto, bem como estratificar o risco de eventos adversos nas mais diversas cardiopatias.^12 – 15 , 418 , 421 , 422^ A diferenciação entre a fibrose isquêmica e não isquêmica pode ser realizada, de forma simples, pelo acometimento do subendocárdico e por obedecer a um ou mais territórios coronarianos nas fibroses por infarto do miocárdio. A Figura 4 traz uma representação gráfica dos diferentes padrões de fibrose miocárdica e a sua correlação com o diagnóstico de cardiomiopatias isquêmicas e não isquêmicas.^14 , 15^ Em virtude de tais características, a RMC é qualificada como uma ferramenta indispensável na avaliação inicial de pacientes com cardiomiopatias sem definição de sua etiologia ( Tabela 23 ).

**Tabela 23:** Emprego da ressonância magnética cardíaca na avaliação de cardiomiopatias

Indicação	Classe de recomendação	Nível de evidência
Avaliação inicial de pacientes portadores de cardiomiopatia^2 , 418 – 420 , 431 – 434^	I	B

Nas cardiopatias congênitas, outra técnica que pode ser acrescentada à RMC é angio-RM com o uso de contraste (angio-RM da aorta torácica e das artérias pulmonares), com o objetivo de uma abordagem integral dessas cardiopatias na avaliação das lesões vasculares associadas, que impactam na escolha do melhor tratamento e no sucesso das intervenções cirúrgicas e/ou hemodinâmicas.^2^ Os exames de angio-RM das estruturas vasculares também podem ser solicitados isoladamente em patologias específicas, como a angio-RM da aorta torácica em pacientes em seguimento de aneurisma da aorta ascendente para avaliar o momento de uma possível indicação cirúrgica.^2^

Dessa forma, diante das numerosas técnicas e informações que podem ser avaliadas pelo exame de RM, recomenda-se que a realização do exame de RMC seja dirigida (utilizando-se protocolos específicos) pelas hipóteses levantadas no pedido médico pelo solicitante, propiciando aos serviços que realizam esses exames e aos médicos responsáveis por sua execução e análise que tenham os dados necessários para um melhor resultado em benefício dos pacientes.

### 3.1. Uso dos Mapas Multiparamétricos no Diagnóstico Diferencial das Miocardiopatias

Na última década, a utilização dos mapas multiparamétricos cresceu significativamente e consolidou-se como uma ferramenta útil no diagnóstico diferencial das miocardiopatias. Essa técnica permite a avaliação de processos patológicos no miocárdio, sendo útil na pesquisa de edema miocárdico, depósito de ferro e presença de fibrose intersticial e áreas de infartos, portanto, apresentando papel relevante na avaliação de resposta a tratamentos e fornecendo informações prognósticas. Esses mapas fornecem informações baseados em alterações nos parâmetros miocárdicos de T1, T2 e volume extracelular (VEC).

#### 3.1.1. Técnica do Mapa T1

O mapeamento de T1 é definido como o tempo de relaxamento magnético longitudinal do miocárdio (T1). O tempo de relaxamento longitudinal, ou " *spin-lattice* ", é a medida que avalia a rapidez com que a magnetização de um próton retorna ao seu estado de equilíbrio após serem excitados por um pulso de radiofrequência (RF) do aparelho de RM. O valor de T1 é codificado em cada *pixel* por essa sequência.

O mapeamento de T1 é tido como uma ferramenta capaz de caracterizar as diferentes estruturas presentes no coração,^435^ distinguindo miocárdio, áreas de fibrose e presença de edema de modo preciso. Tem o potencial de detectar alterações estruturais difusas do miocárdio não avaliáveis por outros métodos não invasivos, incluindo o RT. Diversas terminologias são incorporadas ao mapeamento T1, sendo T1 nativo (imagens sem o contraste paramagnético), T1 pós-contraste (imagens adquiridas após a injeção do contraste) e VEC (derivação de valores após a administração de contraste) as mais utilizadas. O T1 nativo e o VEC são as variáveis de maior impacto na prática clínica diária.

#### 3.1.2. Princípios Básicos

O princípio básico do mapeamento de T1 é a aquisição de uma sequência com múltiplas imagens com diferentes ponderações em T1 e ajuste de curva não linear, utilizando-se a intensidade de sinal e tempo após inversão de cada imagem.^428 , 436^ Os tempos T1 podem ser determinados em regiões de interesse, segmentos do miocárdio ou cada localização do *pixel* para formar um mapa T1.

Já o VEC é obtido por meio de uma equação matemática, que considera os tempos T1 dos mapas pré e pós-contraste e o valor do hematócrito. O valor do hematócrito deve ser medido preferencialmente no mesmo dia do exame.

Diversas técnicas (por exemplo, MOLLI, shMOLLI, SASHA) são capazes de quantificar os tempos de relaxamento T1 do miocárdio, cada uma com vantagens e limitações específicas. Para comparação dos valores de T1, é importante que seja o mesmo protocolo utilizado, o mesmo tipo e dose de contraste, no mesmo campo magnético (1.5 T vs. 3.0 T) e usando o mesmo método de processamento.

O mapa de T2 é uma ferramenta que se mostrou sensível na avaliação da inflamação miocárdica e lesão reversível, permitindo avaliação da inflamação aguda/ativa sem uso do contraste com gadolínio.

A identificação de edema pelo mapa de T2 mostrou-se superior quando comparadas as técnicas tradicionais de sangue escuro ponderadas em T2, sendo este mapa importante no diagnóstico de miocardiopatia inflamatórias e elevado em fases agudas de IAM.^437 – 439^

#### 3.1.3. Diagnóstico Diferencial de Miocardiopatias

Os mapas T1 e T2 mostraram-se ferramentas capazes de auxiliar no diagnóstico diferencial das miocardiopatias, uma vez que se observou que esses mapas apresentam comportamento diferente em cada patologia. O mapa T2 tem grande utilidade na identificação de edema, já o mapa T1 tem a capacidade de identificar edema, fibrose difusa e infiltração miocárdica ( Tabela 24 ).

**Tabela 24:** Padrões mais comumente encontrados de mapa T1 e volume extracelular (VEC) nas miocardiopatias.

Miocardiopatia	T1	VEC	Realce tardio (RT)
Miocardite^440 – 443^	Elevado fase aguda. Acometimento difuso.	Elevado	RT mesocárdico, epicárdico, de padrão coronariano.
Síndrome de Takotsubo^444^	Elevado nas áreas com alteração de contratilidade.	Elevado nas áreas com alteração de contratilidade.	Ausente
Infarto do miocárdio^445 , 446^	Alteração segmentar em território infartado. Elevações mais acentuadas na fase aguda.	Muito elevado nas áreas de fibrose.	RT padrão transmural/subendocárdico. Correspondência com território coronariano.
Amiloidose cardíaca	Aumento significativo do valor de T1, difuso.	Acentuada infiltração extracelular. Aumento importante do valor do VEC.	RT difuso/subendocárdico/transmural
Doença de Anderson-Fabry^447^	Reduzido difusamente.	-	Classicamente, ínfero-lateral parede média de padrão não coronariano
Miocardiopatia por depósito de ferro^448^	Reduzido difusamente.	Reduzido	Ausente
Miocardiopatia hipertrófica^449 , 450^	Elevado	Elevado	RT de padrão não coronariano, mesocárdico. Multifocal.
Miocardiopatia dilatada^449^	Elevado	Elevado	Ausente ou mesocárdico, focal, comumente localizado no septo interventricular, parede inferior ou epicárdio.

O valor do mapa T1 encontra-se prolongado nos quadros nos quais o compartimento extracelular está aumentado, sendo elevado especialmente quando há edema (por exemplo, aumento da água tissular na inflamação do infarto agudo) e aumento do espaço intersticial (por exemplo, fibrose cicatricial do infarto/cardiomiopatia e depósito amiloide). Por outro lado, apresenta redução dos valores quando há sobrecarga de lipídios (por exemplo, doença de Anderson-Fabry) e sobrecarga de ferro. Já o VEC está aumentado no excesso de depósito de colágeno e baixo na metaplasia lipomatosa. A Tabela 24 resume as principais características do mapeamento de T1 nas miocardiopatias.

#### 3.1.4. Infarto/Miocardiopatia Isquêmica

Nos pacientes na fase aguda de infarto, os valores de T1 nativo encontram-se elevados em virtude da presença de edema miocárdico.^428^ As alterações segmentares de T1 nativo em comparação às áreas de RT, apresentação correlação significativa, especialmente em paciente com infarto do miocárdio crônico, sendo adequadas para classificar áreas sem RT, áreas com RT, mas viáveis e áreas com RT, não viáveis.^451^ Os valores de VEC encontram-se elevados nas áreas de fibrose miocárdica.^452^ Nas áreas de hemorragia intramiocárdica e áreas de " *no-reflow* ", pode-se observar valores reduzidos de T1 nativo e pseudonormalização, respectivamente.^445^

#### 3.1.5. Miocardite

O diagnóstico de miocardite pela RMC é realizado com a utilização pelos critérios de Lake-Louise,^437^ que se baseiam pela identificação de necrose, edema e cicatrização/inflamação. Estudos recentes mostram que o emprego dos mapas T1 e T2 aumentam a acurácia diagnóstica quando comparadas as técnicas classicamente utilizadas.^453^ O T1 nativo é capaz de avaliar lesão e edema miocárdico de modo mais sensível que a técnica de RT precoce ( *early gadolinium ehancement* ), e T2 nativo é utilizado para avaliação de edema ao invés das técnicas classicamente utilizadas com imagens ponderadas em T2.^438 , 440^ O RT de padrão não coronariano, mesocárdico e epicárdico é usualmente encontrado. O T1 nativo e VEC encontram-se habitualmente aumentados, caracterizando fibrose miocárdica difusa.^441^ Adicionalmente, o acometimento cardíaco pelo novo coronavírus é relativamente comum. Os pacientes acometidos apresentam elevações significativas de T1 e T2 nativos, e essas elevações apresentam correlação com níveis de troponina.^454^ Os achados, entretanto, são indistinguíveis de miocardites de outras etiologias.

#### 3.1.6. Síndrome de Takotsubo

Caracterizada por redução transitória da fração de ejeção, com alteração de contratilidade segmentar, mais comumente relacionada à região apical do ventrículo esquerdo. Nos pacientes com síndrome de Takotsubo, observou-se elevações nos valores de T1 e T2 nativo, bem como do VEC, de modo mais acentuado nos segmentos com alteração de contratilidade. No seguimento tardio desses pacientes, notou-se progressiva redução desses valores, associado à recuperação da função ventricular.^444^

#### 3.1.7. Amiloidose Cardíaca

O depósito de proteína amiloide no miocárdio está associado a aumento da espessura miocárdica e fibrose miocárdica difusa, com presença de RT subendocárdico e transmural distribuídos em toda extensão ventricular. Ambos os tipos de amiloidose cardíaca mostram valores de T1 nativos marcadamente elevados, mas habitualmente a amiloidose ATTR ( *transthyretin-related cardiac amyloidosis* ) apresenta acometimento miocárdico mais extenso que a amiloidose de AL ( *light chains* ).^455^ A amiloidose cardíaca está associada a um VEC mais alto do que qualquer outra miocardiopatia secundária a infiltração extracelular ampla e substancial do depósito de proteína amiloide.^452^ Alguns estudos recentes mostraram que o mapa T1 (T1 nativo e VEC), realizado de modo seriado, podem ser uma ferramenta não invasiva para acompanhar a resposta ao tratamento e as mudanças na estrutura miocárdica.^456^ Em pacientes com disfunção renal secundária ao acometimento da amiloidose, valores elevados de modo acentuado de T1 nativo sugerem o diagnóstico de amiloidose cardíaca, sem necessidade da administração de gadolínio.^457 , 458^

#### 3.1.8. Doença de Anderson-Fabry

A doença de Anderson-Fabry é uma condição rara caracterizada pelo depósito intracelular de lipídios que resulta em hipertrofia ventricular. Nessa patologia, nota-se que paciente acometidos apresentam valores de T1 nativo reduzidos globalmente quando comparados a voluntários saudáveis e pacientes com outras comorbidades.^447^ Os valores de T1 foram inversamente proporcionais à espessura das paredes e estavam alterados mesmo em pacientes sem hipertrofia ventricular, mostrando que essa técnica é um marcador precoce de acometimento cardíaco.^459^ Nas áreas com presença de RT, os valores de T1 apresentavam-se normais ou elevados.^447^

#### 3.1.9. Miocardiopatia por Depósito de Ferro

A miocardiopatia por depósito de ferro miocárdico é manifestação de hemocromatose. O método de diagnóstico de acometimento cardíaco por depósito de ferro miocárdico é feito pela técnica de T2*. Pacientes com sobrecarga de ferro intramiocárdico (T2* < 20 ms) apresentam valores reduzidos de T1 e T2 nativos.^460 – 462^ Os valores de mapa T1 e T2 nativos foram significativamente correlacionados com os valores de T2* e apresentaram menor variabilidade intra e interobservador.^462^ O mapa T1 pode ser considerado um método alternativo para quantificação do ferro cardíaco, com o potencial para detecção sobrecarga leve de ferro, com grande reprodutibilidade. Essas características têm implicações potenciais para o desenho de ensaios clínicos e monitoramento terapêutico.^448^

#### 3.1.10. Cardiomiopatia Hipertrófica (CMH)

A cardiomiopatia hipertrófica (CMH) é uma doença genética caracterizada por hipertrofia do ventrículo esquerdo (VE). Os valores T1 nativos são aumentados na CMH e apresentam correlação com o grau de espessura miocárdica. Os valores de T1 pós-contraste estão reduzidos com a presença de fibrose intersticial difusa fora das áreas de RT.^449 , 463 , 464^ O VEC na CMH encontra-se acima dos valores normais mesmo em áreas sem a presença de RT.^452^ T1 nativo prolongado e elevação de VEC podem estar presentes mesmo em pacientes sem RT e obstrução da via de saída, sugerindo fibrose miocárdica difusa, e estão relacionados ao grau de hipertrofia ventricular esquerda, sendo marcador precoce de doença.^465^ Em uma coorte com 263 pacientes com diagnóstico de CMH, elevações do VEC, durante o seguimento médio de 28 meses, foram associadas com ocorrência de eventos cardíacos (morte, transplante cardíaco, IC, morte súbita abortada, parada cardiorrespiratória após síncope).^450^ No diagnóstico diferencial entre essa condição e o coração de atleta, os valores de T1 nativo e VEC encontram-se caracteristicamente normais ou reduzidos nas hipertrofias adaptativas às práticas desportivas.^428^

#### 3.1.11. Cardiomiopatia Dilatada (CMD)

Os valores de T1 nativo e VEC encontram-se aumentados nos pacientes com cardiomiopatia dilatada (CMD) quando comparados a controles saudáveis, e os valores de T1 pós-contraste estão reduzidos.^449 , 466^ Os valores de VEC refletem o conteúdo de colágeno do miocárdio na CMD, podendo servir como uma ferramenta não invasiva para monitorar a resposta ao tratamento e auxiliar na estratificação de risco em diferentes estágios da doença.^467^ Uma metanálise recente, com 1.242 pacientes, mostrou que o VEC e o T1 apresentaram alto valor prognóstico para um desfecho composto de mortalidade e morbidade, com HR 1,38 (IC95%, 1,18-1,61) e HR 1,20 (IC95%, 1,14-1,27), respectivamente.^468^ Esses achados sugerem que os valores de T1 nativo e VEC podem auxiliar na identificação de pacientes potencialmente graves, que irão desenvolver complicações cardiovasculares maiores.^468^

Outras patologias podem alterar os mapas miocárdicos, como cardiotoxicidade por quimioterápicos, miocardiopatia valvar (classicamente, estenose aórtica), transplante cardíaco e doenças sistêmicas com acometimento cardíaco (como lúpus, artrite reumatoide e esclerose sistêmica).^428^ A investigação diagnóstica com mapas nesses cenários auxilia na identificação precoce de atividade de doença, que normalmente não são visualizadas por outras métodos nessa fase aguda. A Tabela 25 apresenta os principais cenários clínicos envolvidos na utilização dos mapas multiparamétricos pela RMC.

**Tabela 25:** Uso dos mapas multiparamétricos na avaliação das miocardiopatias

Indicação	Classe de recomendação	Nível de evidência
Utilização de mapa T1 no diagnóstico de amiloidose cardíaca^469 , 470^	I	A
Utilização de mapa T1 na resposta terapêutica de pacientes com amiloidose cardíaca^436^	IIa	B
T2* na avaliação quantitativa de depósito de ferro miocárdico^471 – 474^	I	A
Mapa T1 na avaliação do depósito de ferro miocárdico^475^	IIb	C
Mapa T1 no diagnóstico de doença de Anderson-Fabry^476 – 478^	I	B
Pesquisa de edema miocárdico com técnica de mapa T2 nas miocardiopatias inflamatórias^437 , 439 , 479^	IIa	B
Mapa T1 na avaliação prognóstica de portadores de miocardiopatia dilatada^480 – 483^	IIa	B
Mapa T1 na avaliação de portadores de cardiomiopatia hipertrófica^442 , 443 , 465 , 484^	IIb	B

### 3.2. Pesquisa de DAC pela Ressonância Magnética – Isquemia Miocárdica

As últimas três décadas têm demonstrado um crescimento contínuo na utilização da RMC na avaliação diagnóstica e prognóstica da cardiopatia isquêmica.

A RMC com estresse farmacológico apresenta diversas vantagens tecnológicas que resultam em acurácia diagnóstica superior quando comparadas aos demais métodos usados comumente, como cintilografia do miocárdio e ecocardiograma com estresse. A RMC apresenta maior campo de visão ( *field-of-view* ), maior resolução espacial e consequente maior habilidade para diferenciar os diversos tecidos. Além disso, a elevada resolução espacial da ressonância permite identificar defeitos de perfusão em diversas camadas do subendocárdio. A identificação do defeito perfusional é feita através da primeira passagem do contraste baseado em gadolínio, o que torna a técnica menos suscetível a perda de acurácia no contexto de isquemia balanceada.

A RMC é capaz de avaliar múltiplos parâmetros da cardiopatia isquêmica, como detecção de isquemia, presença de fibrose/necrose resultante do infarto do miocárdio, determinação da viabilidade miocárdica e mais recentemente os mapas paramétricos de T1, T2 e VEC do miocárdio.

A RMC demonstra alta acurácia e reprodutibilidade para análise da função global e segmentar biventricular, independentemente da geometria ventricular e do biótipo do paciente. Na avaliação dos pacientes com sequelas do IAM, como aneurismas e pseudoaneurismas, a RMC é capaz de identificar de forma precisa os volumes e a geometria das câmaras cardíacas (além da área infartada), sendo importante na avaliação da melhora da função cardíaca e anatomia ventricular após procedimentos de revascularização miocárdica.^485 , 486^ Portanto, a RMC é um método apropriado para a avaliação da contratilidade e função ventricular global e segmentar, sendo hoje considerada o padrão-ouro para essa finalidade.^487 , 488^

#### 3.2.1. Detecção de Isquemia Miocárdica

Atualmente, a presença de isquemia do miocárdio pode ser detectada através da perfusão de primeira passagem sob efeito do estresse farmacológico e em repouso ou através da avaliação da contratilidade através da indução de isquemia com dobutamina, sendo a primeira com maior sensibilidade e a segunda com maior especificidade.^489 – 491^

#### 3.2.2. Avaliação da Perfusão Miocárdica

Atualmente, a forma mais frequente de avaliar cardiopatia isquêmica pela ressonância é através da perfusão do miocárdio. A utilização da perfusão sob estresse farmacológico usualmente é realizada em duas fases (estresse e repouso), através da infusão de gadolínio para definir áreas hipoperfundidas. Durante a infusão de fármacos vasodilatadores, como dipiridamol e adenosina, ocorre significativa hiperemia da microcirculação coronariana não associada a estenoses epicárdicas significativas. Contudo, o mesmo não ocorre nos territórios supridos por coronárias com estenoses significativas, que já apresentam vasodilatação máxima compensatória (mecanismo de reserva coronariana). Essa diferença de perfusão entre os territórios isquêmicos e remotos permite identificar defeitos de perfusão miocárdica, fornecendo importantes informações para o manejo e prognóstico desses pacientes.^492^

A avaliação da perfusão do miocárdio é mais frequentemente realizada de forma visual ou, de maneira menos frequente, através de análise semiquantitativa ou quantitativa (necessitando de *softwares* específicos). As imagens da perfusão por RMC para avaliação de isquemia são feitas através da comparação das imagens sob o estresse farmacológico comparadas às imagens em repouso, após a reversão com aminofilina do estresse farmacológico realizado com dipiridamol. As imagens do RT devem ser analisadas conjuntamente na leitura da perfusão miocárdica, devido ao fato de as áreas com isquemia intensa (obstruções acima de 90%) poderem demonstrar déficit perfusional tanto na fase de estresse quanto na fase de repouso. Todavia, como temos a possibilidade de visualizar diretamente a fibrose miocárdica pela técnica do RT, a etapa da perfusão em repouso não é obrigatória.

A avaliação de isquemia através da perfusão do miocárdio pela RMC apresenta elevada acurácia diagnóstica e já foi comparada com outros métodos diagnósticos no início dos anos 2000. Estudos unicêntricos demonstraram a superioridade da RMC em relação à cintilografia e valores semelhantes à PET.^493^ O estudo multicêntrico MR-IMPACT, publicado em 2008, demonstrou elevado poder diagnóstico da perfusão do miocárdio para detectar isquemia.^494^ Seguindo a evolução do conhecimento, metanálises foram publicadas. Nandalur et al.^495^ publicaram uma análise de 1.183 pacientes cuja sensibilidade e especificidade médias da RMC foram de 91 e 81%, respectivamente, na detecção de doença coronariana obstrutiva. Já em 2010, Hamon et al.^496^ publicaram estudo demonstrando elevada sensibilidade (89%) e especificidade (80%) no diagnóstico de DAC obstrutiva.

Estudos recentes com maior número de pacientes foram publicados, demonstrando a grande utilidade da RMC com estresse farmacológico na prática cardiológica diária. Estudos como o MR-Impact II, publicado em 2013, demonstraram a superioridade do método quando comparado com cintilografia do miocárdio.^494 , 497^ Já o estudo CE-MARC publicado em 2012^10^ avaliou 752 pacientes e comparou com angiografia como padrão ouro para definição de estenose > 70% ou lesão de tronco de coronária esquerda > 50%, com sensibilidade e especificidade de 87 e 83%, respectivamente.

O valor prognostico da RMC na cardiopatia isquêmica apresenta dados consistentes. O estudo MR-INFORM avaliou 918 pacientes sintomáticos e comparou diferentes estratégias de investigação, demonstrando que a estratégia com RMC não apresenta inferioridade em relação à estratégia com utilização do FFR invasivo (3,7% para FFR vs. 3,6% para RMC), sem diferença em 12 meses para desfechos primários.^498^ Já o estudo SPINS avaliou 2.349 pacientes com dor torácica e demonstrou que a ausência de isquemia ou RT está associada a número baixo de eventos cardiovasculares em até 5 anos após o exame de RMC.^499^

A avaliação de isquemia pela RM apresenta profunda base de estudos realizados em diversos cenários clínicos e comparados com diversos métodos de detecção de cardiopatia isquêmica. Além do mais, na última década, diversos estudos avaliaram o fator prognóstico da presença/ausência de déficit perfusional, demonstrando ser uma ferramenta útil para estratificação de risco. Diversas diretrizes internacionais ressaltam a importância desse método na avaliação da cardiopatia isquêmica.

#### 3.2.3. Avaliação da Contratilidade Segmentar/Reserva Contrátil

A ressonância com estresse farmacológico com dobutamina é a forma mais utilizada na avaliação de isquemia através da avaliação da contratilidade segmentar ou da reserva contrátil, devido às dificuldades técnicas para a realização do exercício físico no interior do aparelho de ressonância.

Dessa forma, com doses crescentes de dobutamina, a isquemia miocárdica durante o exame de estresse pode ser definida como um novo déficit contrátil segmentar decorrente da infusão de dobutamina ou a ocorrência de resposta bifásica, isto é, o aumento da contratilidade miocárdica em baixas doses e disfunção segmentar em altas doses de dobutamina.^430^

As vantagens desta técnica de avaliação de estresse com dobutamina quando comparada com o ecocardiograma se deve à alta qualidade de imagem e, consequentemente, à elevada reprodutibilidade dos resultados, já que problemas de janela acústica com RMC não existem.^500^

A avaliação da cardiopatia isquêmica com RMC com dobutamina segue o mesmo protocolo utilizado nos exames de ecocardiografia sob estresse, com doses crescentes da droga (10, 20, 30, 40 mcg/kg por 3 minutos), podendo ou não ser adicionada atropina no término da dose de 40 mcg/kg com o objetivo de atingir a frequência cardíaca submáxima do paciente.^501^ A taxa de complicações no exame de RMC é baixa, sendo inferior a 0,1%, semelhante à de estudos com ecocardiografia de estresse.^502^ O protocolo de aquisição de imagens envolve sequências para avaliação dinâmica da função (cine) em diferentes planos de corte, abrangendo os 17 segmentos miocárdicos.^501^ A análise na prática clínica é comumente realizada de forma qualitativa; entretanto, técnicas de avaliação quantitativa (por exemplo, *tagging* ) têm sido utilizadas em estudos clínicos, demonstrando facilitar a identificação de isquemia, tanto na forma qualitativa como quantitativa. Na década de 1990, foram publicados os primeiros estudos de RMC de estresse com dobutamina, que demonstraram a alta acurácia do método para diagnóstico de obstruções coronarianas ≥ 50%, com sensibilidade de 81 a 84%.^503^ A metanálise de Nadalur et al. demonstrou sensibilidade de 83% e especificidade de 86% para diagnóstico de lesões coronárias significativas em pacientes de alto risco para DAC.^495^

A RMC com dobutamina, assim como a RMC perfusional com dipiridamol, apresenta importância na avaliação prognóstica dos pacientes. O exame de RMC com dobutamina normal indica baixa taxa de eventos (< 2% em 2 anos).^504^ A presença de disfunção segmentar identifica pacientes de risco para IAM e morte de causa cardíaca.^505^ O diagnóstico de isquemia determinado pela alteração de motilidade na RMC de estresse com dobutamina é preditor independente de eventos cardíacos (HR = 5,42 em 3 anos; p < 0,001) independentemente do sexo do paciente.^506 , 507^

Contudo, há limitações da RMC com dobutamina, como monitorização adequada do paciente durante o exame (já que o traçado do eletrocardiograma pode sofrer alterações do campo magnético com impossibilidade de avaliação do segmento ST) e contraindicações próprias da infusão da dobutamina.

A Tabela 26 apresenta os principais cenários de indicação da RM na pesquisa de isquemia miocárdica.

**Tabela 26:** Pesquisa de doença arterial coronariana (DAC) pela ressonância magnética – isquemia miocárdica

Indicação	Classe de recomendação	Nível de evidência
Avaliação da perfusão miocárdica sob estresse farmacológico com dipiridamol/adenosina^10 , 297 , 494 , 497 , 498 , 508^	I	A
Avaliação da contratilidade ventricular sob estresse com dobutamina^489 , 502 , 506 , 509^	I	B
Investigação de DAC em pacientes com dor torácica aguda e probabilidade pré-teste intermediária de DAC^8 , 10 , 498^	I	B
Avaliação de angina estável/equivalente angionoso em pacientes com probabilidade pré-teste intermediária de DAC^8 , 10 , 494 , 498 , 508^	I	B
Identificação e quantificação de isquemia miocárdica em pacientes com DAC conhecida (exceto pacientes com anatomia de alto risco * )^510 , 511^	I	B
Investigação de isquemia miocárdica em pacientes revascularizados (cirurgicamente ou de forma percutânea) com sintomatologia sugestiva de DAC obstrutiva^512^	I	B
Avaliação de pacientes com DAC não obstrutiva conhecida e/ou suspeita de INOCA^513 – 515^	IIa	C

* Definida como estenose > 50% em tronco coronário esquerdo e triarteriais com acometimento coronariano proximal. INOCA: isquemia e doença arterial coronariana não obstrutiva.

### 3.3. Pesquisa de DAC pela Ressonância Magnética – Viabilidade Miocárdica

A RMC com técnica de RT miocárdico (RTM) tem sido considerada a referência clínica mais disponível para a avaliação da viabilidade miocárdica. É intuitivo que a imagem da viabilidade miocárdica ajude na avaliação diagnóstica e prognóstica, o que todavia vem sendo contestado por ensaios clínicos que indicaram falha da imagem de viabilidade miocárdica em indicar precisamente a revascularização do miocárdio para que levasse a uma redução de eventos cardíacos adversos.^516 , 517^ No entanto, um recente e novo olhar sobre a viabilidade miocárdica tem surgido e é baseado em dados de publicações utilizando técnicas avançadas como a RMC para avaliação da viabilidade miocárdica^518^ e em dados recentes com seguimento de muito longo prazo de estudo randomizado.^519^ Além disso, os grandes estudos clínicos que lançaram dúvidas iniciais sobre a efetividade da viabilidade miocárdica, em sua maioria, não utilizaram a RMC. Assim, a imagem de viabilidade tem implicações clínicas significativas, e novos estudos randomizados estão em andamento e apontam para uma utilização clínica crucial da viabilidade miocárdica.^520^ Portanto, a despeito do debate em curso, a RMC mantém potencial significativo para avaliar a viabilidade miocárdica com vistas à melhora do prognóstico do paciente.^521 – 523^

#### 3.3.1. A RMC e suas Técnicas

A RMC pode caracterizar a suspeita de miocárdio em hibernação por uma combinação de técnicas: a espessura diastólica da parede do VE (EDPVE), a reserva inotrópica da função contrátil segmentar e a extensão transmural do infarto miocárdico com imagem RTM. Outros métodos de RMC para avaliação da viabilidade miocárdica não são de uso clínico de rotina e fogem do escopo deste documento.

Na DAC crônica, o afilamento da parede ocorre como resultado da reabsorção do infarto e retração fibrótica, mas também pode ocorrer como resultado de isquemia miocárdica grave. Assim, a EDPVE isoladamente é limitada para a previsão de recuperação funcional após a revascularização. Classicamente, paredes com espessura diastólica menor que 5,5 mm seriam consideradas sem potencial de recuperação contrátil após revascularização miocárdica,^524^ com boa sensibilidade (94%), mas com muito baixa especificidade (52%). Esse uso foi questionado em recente publicação que estimou que aproximadamente 20% dos segmentos miocárdicos disfuncionais e afilados têm pouca fibrose miocárdica e demonstram melhora da contratilidade e do afilamento da parede após a revascularização coronária.^525^

Os agentes de contraste a base de gadolínio reduzem o tempo de relaxamento T1 dos tecidos proporcionalmente à sua concentração local. Constituem-se de grandes macromoléculas, que não adentram o espaço intracelular (não atravessam a membrana celular íntegra de um miócito normal) e, portanto, têm distribuição exclusivamente extracelular. No entanto, quando a membrana celular do miócito está danificada (por exemplo, infarto miocárdico agudo) ou se houver um aumento no espaço extracelular entre os miócitos, o gadolínio se acumula no espaço extracelular, e sua lavagem é lentificada após a injeção. Esse acúmulo leva a aumento do sinal na imagem de RT miocárdico (branco é morto – " *bright is dead* "). Assim, tanto nos cenários de infarto miocárdico agudo ou crônico, o RTM é resultado direto da ausência de miócitos viáveis.^526^

Na rotina clínica, os protocolos atuais realizam imagens de RTM 5 a 10 minutos após a injeção de gadolínio.^527 , 528^ Nos pacientes com DAC, a imagem de RTM que apresenta o miocárdio disfuncional com intensidade de sinal normal anulado (escuro) sugere atordoamento do miocárdio ou hibernação e ausência de infarto. O infarto miocárdico agudo ou crônico aparecerá como áreas de aumento de sinal (branco), quase sempre envolvendo a camada subendocárdica da parede do VE e, na maioria das vezes, respeitando territórios coronarianos específicos. Nos pacientes com DAC e com disfunção contrátil global ou segmentar do VE sendo avaliados quanto aos benefícios da revascularização coronária, a extensão transmural do RTM fornece uma previsão da probabilidade de melhora na contratilidade miocárdica segmentar após revascularização coronária.^422^ Os segmentos acinéticos sem ou com infarto subendocárdico mínimo (< 25%) têm uma chance > 90% de recuperação segmentar da função contrátil se a artéria coronária envolvida for revascularizada com sucesso. Os segmentos com extensão transmural do infarto > 50% têm uma chance < 10% de recuperação contrátil segmentar, apesar da revascularização coronária bem-sucedida.^422^ Em segmentos que demonstram extensão transmural do infarto entre 25 e 50%, a probabilidade da recuperação contrátil é, em geral, próxima a 50%, o que levou a considerá-los na prática clínica como segmentos viáveis e com potencial de recuperação contrátil preservado.^12 , 418 , 421 , 529 – 531^ No entanto, nesse grupo intermediário, a previsão apenas pelo critério de extensão transmural do infarto pode não ser precisa e deve levar em conta outros critérios como a extensão global do infarto (percentual do VE infartado), o número de segmentos viáveis e não viáveis, a adjacência a segmentos não viáveis, assim como a avaliação da reserva contrátil inotrópica.^532 – 535^

Uma das vantagens da RMC com técnica de RTM é a capacidade de avaliar a presença de infarto subendocárdico e delinear a extensão transmural do infarto com alta resolução espacial (1-3 mm no plano da imagem), o que permite a detecção de infartos de pequenas dimensões, não identificados por outros métodos.^421^

A reserva contrátil inotrópica em resposta à infusão de baixa dose de dobutamina (5-10 μg/kg/min) é uma avaliação fisiológica bem validada da viabilidade miocárdica por ecocardiografia ou RMC.^532 , 536 , 537^ Em comparação com outros métodos, a reserva contrátil inotrópica tende a ser mais específica que a avaliação tecidual porque avalia um parâmetro semelhante ao padrão de referência, a função contrátil segmentar, mas com uma sensibilidade mais baixa. Embora a reserva contrátil induzida pela dobutamina possa ter uma especificidade maior do que a extensão transmural da imagem de RTM para prever a recuperação funcional segmentar, especialmente em extensão transmural intermediária do RT,^532^ ela é apenas moderadamente sensível na previsão da recuperação contrátil segmentar.^538^ A combinação das técnicas tecidual e inotrópica pode melhorar a precisão na previsão da recuperação funcional segmentar após revascularização, com o RTM atingindo a maior sensibilidade de 95% e complementada pela alta especificidade de 91% oferecida pela reserva contrátil com dobutamina.^539^

#### 3.3.2. O uso na Rotina Clínica

#### 3.3.2.1. Cenário Agudo

No IAM, a RMC permite, através da determinação da extensão transmural da área infartada, diferenciar áreas com disfunção contrátil compostas por miocárdio viável (atordoadas/hibernadas) de áreas constituídas fundamentalmente de necrose ou fibrose sem potencial de recuperação funcional, com lesão irreversível.^540^ A RMC é precisa em determinar a evolução da função cardíaca, identificar o remodelamento adverso^541^ e determinar a extensão de áreas de obstrução microvascular (" *no-reflow* ").^542 – 544^

**Figura 5:**
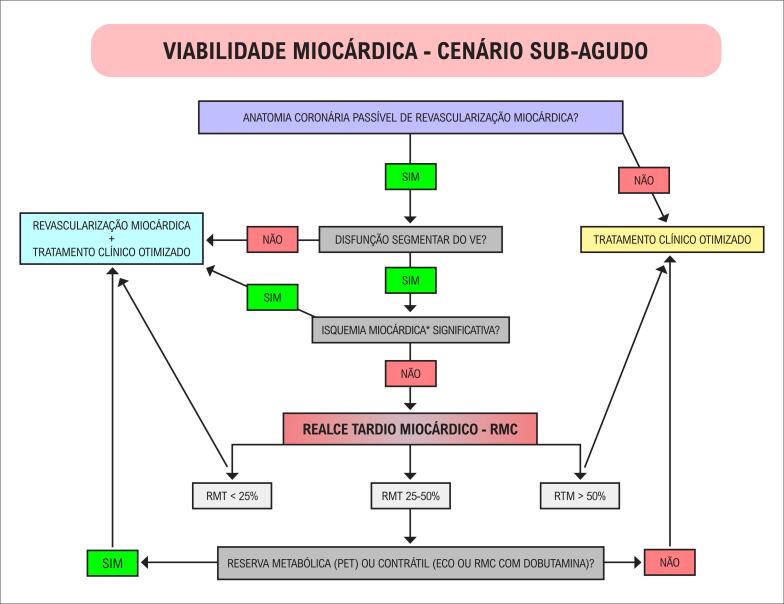
Avaliação de viabilidade miocárdica pela ressonância magnética (cenário sub-agudo). *Definida por sintomas anginosos, alterações eletrocardiográficas ou em testes de imagem. VE: ventrículo esquerdo; RMC: ressonância magnética cardíaca; RTM: realce tardio miocárdico; PET: tomografia por emissão de pósitrons.

Um algoritmo para esse cenário indicaria a utilização da RMC com RTM em pacientes com disfunção contrátil segmentar, particularmente extensa (por exemplo, parede anterior acinética) associada a DAC obstrutiva passível de revascularização miocárdica, seja cirúrgica, mas em especial por angioplastia com implante de *stent* ( Figura 5 ). O poder preditivo da recuperação contrátil na fase aguda do infarto é similar ao da fase crônica, porém, na fase aguda, a avaliação do RTM enfrenta ainda a retração do tamanho do infarto resultante do processo de cicatrização como uma variável adicional (o tamanho do infarto pode reduzir em aproximadamente 22% nos primeiros 6 meses pós-infarto).^418 , 545^

#### 3.3.2.2. Cenário Crônico

A avaliação da viabilidade miocárdica para predizer quais pacientes terão melhora da função ventricular global ou regional após revascularização do miocárdio é de extrema utilidade clínica.^546^

No infarto crônico, a RMC mostrou-se superior à cintilografia por SPECT^421 , 547^ e com mesma sensibilidade e especificidade que a PET para a identificação de áreas de fibrose,^57 , 548^ especialmente em regiões de infarto subendocárdico. A avaliação da extensão transmural ("transmuralidade") das regiões de infarto do miocárdio permite predizer, com excelente acurácia, a probabilidade de recuperação da função regional após a revascularização, seja cirúrgica ou percutânea.^422 , 518 , 549 – 551^

Alguns dados demonstraram que pacientes com cardiopatia isquêmica crônica com disfunção ventricular esquerda, com anatomia coronária passível de revascularização e com predomínio de viabilidade miocárdica nos exames de avaliação não invasiva apresentavam grande benefício em termos de redução da mortalidade total quando adequadamente revascularizados.^552^ Em contrapartida, quando esses mesmos pacientes não apresentam viabilidade miocárdica significativa, não existe benefício dos procedimentos de revascularização em termos de redução de mortalidade.^552^

Um uso comum, em nosso meio, da técnica de RTM é a avaliação de áreas acinéticas e discinéticas, incluindo na suspeita de aneurisma ventricular esquerdo, em especial se o paciente tem procedimento cirúrgico de revascularização miocárdica planejado. A ausência de viabilidade miocárdica nesses territórios pode ajudar na decisão de aneurismectomia ou reconstrução geométrica do VE, assim como no planejamento pré-operatório pelo cirurgião cardíaco.^485^ Embora o valor prognóstico dessa abordagem não tenha sido confirmado, a presença de viabilidade miocárdica indica melhor prognóstico em qualquer das abordagens terapêuticas escolhidas: tratamento clínico otimizado ou cirurgia de revascularização.^1 , 516^

Em pacientes com disfunção segmentar em repouso, a RMC de estresse com baixa dose de dobutamina mostrou-se útil na identificação da reserva contrátil.^538^ Nos casos de viabilidade miocárdica preservada, ocorre uma melhora na disfunção segmentar ventricular durante a infusão de baixa dose de dobutamina. Esses pacientes, ao serem submetidos a revascularização do miocárdio, têm maior probabilidade de melhora do espessamento sistólico.^532 , 539 , 553^

**Figura 6:**
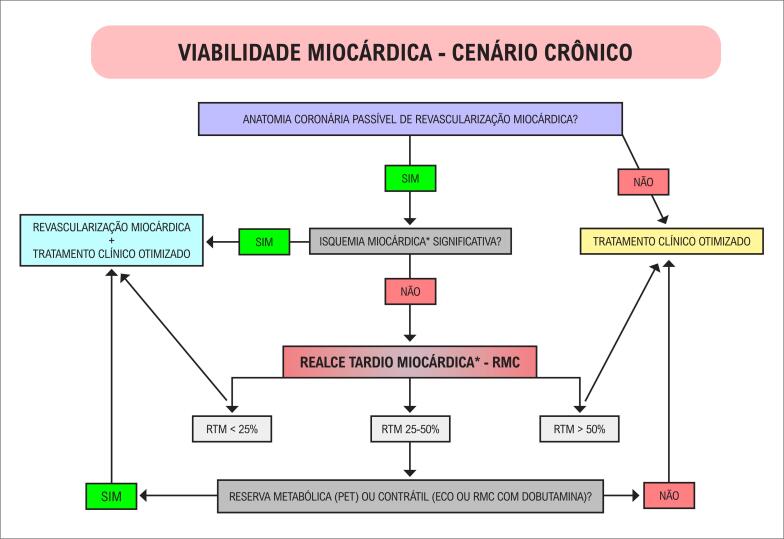
Avaliação de viabilidade miocárdica pela ressonância magnética (cenário crônico). *Definida por sintomas anginosos, alterações eletrocardiográficas ou em testes de imagem. RMC: ressonância magnética cardíaca; RTM: realce tardio miocárdico; PET: tomografia por emissão de pósitrons.

Um algoritmo proposto para o manejo de pacientes com anatomia coronária passível de revascularização miocárdica baseia-se fundamentalmente na avaliação da viabilidade miocárdica e presença de isquemia miocárdica. Diferentemente do cenário agudo, no cenário crônico, a disfunção contrátil e o afilamento parietal passam a ter menor importância, uma vez que podem ser o resultado tanto de hibernação/atordoamento miocárdico como de ausência de viabilidade miocárdica (infarto transmural).^525 , 534^ De forma similar, os casos com RTM intermediário (de 25 a 50% de transmuralidade) beneficiam-se de avaliação da reserva contrátil com dobutamina ou metabólica com PET ( Figura 6 ).^532 , 539 , 553^

#### 3.3.3. Classe de Recomendação e Nível de Evidência

A representação da RMC na avaliação da viabilidade miocárdica em diretrizes internacionais vem aumentando de forma progressiva. No cenário da síndrome coronária aguda, a RMC tem classe de recomendação I e nível de evidência B na suspeita de cardiomiopatia de estresse (Takotsubo) e no diagnóstico diferencial de infarto do miocárdio com artérias coronárias não obstrutivas (MINOCA, de *myocardial infarction with non-obstructive coronary artery* ).^554^ No espectro da viabilidade miocárdica, o quadro clínico de IC em pacientes com DAC é o mais bem representado, com classe de recomendação IIa e nível de evidência B.^139 , 555 , 556^ Similar recomendação é encontrada na avaliação de arritmia ventricular associada a DAC.^557^ Indicações similares e com alta classe de recomendação e nível de evidência foram recomendadas pelas diretrizes brasileiras de RMC e tomografia computadorizada cardíaca (TCC) da SBC/CBR anteriores, de 2006^558^ e 2014.^2^

A Tabela 27 apresenta as recomendações e representa a melhor evidência científica apurada pelo grupo de *experts* desta Diretriz no momento da sua produção.

**Tabela 27:** Pesquisa de DAC pela ressonância magnética – viabilidade miocárdica

Indicações de RMC para avaliação da viabilidade miocárdica no cenário de disfunção ventricular global ou regional (insuficiência cardíaca) em pacientes com DAC conhecida ou suspeita	Classe de recomendação	Nível de evidência
Avaliação da função ventricular regional em repouso e em estresse^559 – 561^	IIa	B
Detecção de infarto agudo e crônico do miocárdio pela técnica de realce tardio^422 , 518 , 525 , 528 , 562 , 563^	I	A
Diagnóstico diferencial com etiologia não isquêmica pela técnica de realce tardio (outras CMPs)^564 – 566^	I	B
Avaliação de viabilidade miocárdica no cenário clínico pré-revascularização pela técnica de realce tardio * ^422 , 518 , 567^	I *	B *
Detectar trombo ventricular^568 – 571^	I	B
Avaliação de aneurisma de VE^525 , 572^	I	B

* Nível de evidência (NE) e classe de recomendação (CR) referentes à escolha do **método** de ressonância cardíaca como estratégia preferencial para pesquisa de viabilidade miocárdica. A classificação apresentada não representa NE e CR da pesquisa de viabilidade miocárdica (de maneira geral) como estratégia de pré-revascularização miocárdica. DAC: doença arterial coronariana; RMC: ressonância magnética cardíaca; CMPs: cardiomiopatias; VE: ventrículo esquerdo.

### 3.4. Angiorressonância das Artérias Coronárias

O diagnóstico de DAC e a avaliação da isquemia miocárdica são primordiais para a prevenção de eventos cardíacos futuros.

A angio-RM das artérias coronárias apresenta como característica a não utilização de radiação ionizante ou quaisquer meios de contraste. Apesar do reconhecimento precoce deste potencial extraordinário, limitações técnicas significativas, incluindo resolução espacial reduzida, aquisição longa e baixa relação sinal-ruído prejudicando a qualidade da imagem, fizeram com que a angio-RM de artérias coronárias fosse substituida por outras técnicas não invasivas para avaliação de DAC. A angio-TC das artérias coronárias devido a sua praticidade e rapidez assumiu um papel mais importante na rotina da prática clínica.^573 – 575^ A angio-RM das artérias coronárias foi utilizada como método não invasivo para avaliar DAC em diferentes grupos de pacientes, em investigações clínicas em estudos unicêntricos,^576 , 577^ bem como estudos multicêntricos,^578 , 579^ embora não tenha mostrado boa reprodutibilidade fática no mundo real. No entanto, a angio-RM das artérias coronárias provou ser importante no delineamento de anomalias coronárias congênitas para as quais é recomendada como a modalidade clínica de escolha, particularmente quando há preocupação com o uso de radiação e contraste.^580 , 581^

Didaticamente, podemos citar três linhas de frente de desenvolvimento de pesquisa na avaliação das artérias coronária por RM:

1. Avaliação do grau de estenoses coronárias com a implementação de técnicas de queda significativa de sinal nas regiões com maior estenose.^582 , 583^
2. Avaliação das características e vulnerabilidade das placas.^584 , 585^
3. Análise fisiológica do fluxo das artérias coronárias, incluindo análise do fluxo sanguíneo coronariana pela técnica de *phase contrast* ,^586^ fluxo no seio coronariano,^587^ reserva de fluxo coronariano e gradiente de fluxo através da estenose coronariana.^588 , 589^

Diante das evidências atuais, podemos usar a angio-RM das artérias coronárias na prática clínica para avaliação da origem das artérias coronárias e doença de Kawasaki (segmentos proximais e com maior calibre), com nível de evidência IIa e possívelmente apropriado ( Tabela 28 ).

**Tabela 28:** Cenários clínicos para utilização da angiorresonância de artérias coronárias

Indicação	Classe de recomendação	Nível de evidência
Avaliação da origem das artérias coronárias^590 , 591^	IIa	B
Seguimento de portadores de doença de Kawasaki (avaliação de aneurismas coronarianos)^592 – 594^	IIa	B
Avaliação de doença arterial coronariana^10^	III	B

A avaliação do grau de estenose, da reserva de fluxo coronariano e do gradiente de fluxo através das áreas estenóticas ainda se encontram em desenvolvimento.

### 3.5. Diagnóstico Diferencial de Troponina Positiva com Coronárias Normais (TP-NOCA/MINOCA)

Embora o diagnóstico de IAM esteja habitualmente ligado à presença de obstrução coronária, sabemos que existe um considerável número de pacientes, entre 6 e 8%,^595^ com SCA associada a artérias coronárias angiograficamente normais.^596^ O diagnóstico de MINOCA requer documentação de um IAM e coronariografia invasiva ou angio-TC das artérias coronárias sem obstrução significativa.^597^ O diagnóstico de MINOCA, assim como o diagnóstico IAM, necessita da presença de um mecanismo isquêmico responsável pela lesão miocárdica; assim, causas não isquêmicas como miocardites ou síndrome de Takotsubo estão excluídas por definição desse diagnóstico. A última definição de IAM da Sociedade Europeia de Cardiologia^598^ define que três características necessitam estar presentes para que o diagnostico de MINOCA possa ser confirmado: 1) os mesmos critérios diagnósticos de IAM (cenário clínico com biomarcadores cardíacos alterados) na presença de lesão coronária; 2) a lesão coronária angiográfica deve ser < 50%; e 3) que não se identifique outra causa clínica que possa ser responsável pelos achados compatíveis com a presença de lesão miocárdica (por exemplo, miocardite ou embolia pulmonar). Nesta seção, embora seguindo essas considerações, discutiremos algumas diferenças em apresentações que frequentemente figuram no diagnóstico diferencial.

Muitos pesquisadores consideram MINOCA, assim como IC, um diagnóstico em andamento ( *working diagnosis* ), por muitas vezes haver dificuldade na identificação da etiologia para correta orientação terapêutica.^597^ Assim, alguns diagnósticos diferenciais se estabelecem por cumprirem os princípios característicos de IAM tipo I ou II. Incluem-se doenças isquêmicas decorrentes da placa coronariana (erosão, rutura ou ulceração), dissecção coronária, tromboembolismo, espasmo coronário microvascular, embolia coronária, além de cardiomiopatias inflamatórias (miocardite de qualquer etiologia), síndrome de Takotsubo ou mesmo embolia pulmonar.^599^ Dessa forma, o diagnóstico preciso de MINOCA é fundamental para escolher-se a melhor opção terapêutica para pacientes isquêmicos e não isquêmicos.^600^ Pela sua complexidade diagnóstica, foi sugerido englobarem-se esses diagnósticos sob a denominação TpNOCA ( ***T*** *roponin* ***p*** *ositive with* ***N*** *on* ***O*** *bstructive* ***C*** *oronaries* ***A*** *rteries* ), que seriam síndromes de elevação de troponina sem obstruções coronarianas por terem os níveis elevados de troponina como marcador comum. Essa denominação seria subcategorizada pelas causas coronárias epicárdicas (MINOCA), causas miocárdicas (por exemplo, miocardites) e causas extracardíacas (por exemplo, embolia pulmonar).^601^

A RMC consiste em uma das mais importantes ferramentas na determinação da etiologia dos casos de MINOCA, podendo definir até 74% desses casos.^602^ O RT, quando presente, permite a localização da área de lesão miocárdica, além de fornecer evidências dos mecanismos envolvidos. Adicionalmente, a RMC permite identificar os pacientes com pior prognóstico, podendo alterar a terapêutica em aproximadamente 50% dos casos,^603^ o que permite personalizar adequadamente a terapia médica (incluindo prevenção secundária). Além disso, a abordagem com a utilização da RMC pode evitar prescrição desnecessária de drogas com seus respectivos efeitos colaterais, como sangramento no caso de uso de antiagregantes plaquetários.^602^

Aproximadamente 23% dos casos diagnosticados como MINOCA são decorrentes de aterosclerose coronariana em sua forma não obstrutiva, decorrentes de erosão ou ulceração de placas com consequente trombose momentânea e recanalização do vaso comprometido, consequente a vasoespasmo prolongado.^595^ A RMC consegue confirmar o diagnóstico de infarto pela técnica de RT e permite diferenciar de outras lesões pelas técnicas que identificam edema (como em casos de miocardite), seja pelas técnicas tradicionais ponderadas em T2 ou pelos novos mapas paramétricos (mapas T1 e T2).^604^

Outra causa relevante e que se confunde com o diagnóstico de MINOCA são as miocardites. Correspondem a aproximadamente 29% dos casos,^595^ sendo a etiologia viral a mais frequente, tendo ganhado maior repercussão após a pandemia de covid-19, evidenciando alterações miocárdicas em aproximadamente 50% dos casos recuperados.^605 – 608^ A aplicação da RMC na avaliação de miocardites será tratada em tópico dirigido nesta Diretriz.

Aproximadamente 16% dos casos de MINOCA se apresentarão como síndrome de Takotsubo.^595^ A RMC se mostra como excelente ferramenta diagnóstica, uma vez que permite a identificação das áreas discinéticas em qualquer segmento do VE (embora a discinesia apical transitória seja a mais frequente manifestação na síndrome de Takotsubo). Além disso, permite caracterizar as áreas de edema miocárdico, sendo colocada como método de escolha em consenso publicado recentemente.^609^ A fase inflamatória, em que é possível fazer a detecção do edema miocárdico, costuma desaparecer em 3 meses.^610^ A RMC de controle nesses casos pode ser solicitada para verificação de reversão da área discinética, bem como do desaparecimento do edema miocárdico, com a ratificação do diagnóstico de Takotsubo. Habitualmente, não se observam áreas de RT nessa síndrome; entretanto, na fase aguda, pequenas áreas de RT podem ser visualizadas nas áreas discinéticas, pelo aumento do espaço intersticial das áreas inflamadas.

Outros diagnósticos possíveis são menos frequentes e podem ser observados como etiologia de MINOCA, como cardiomiopatia hipertrófica em aproximadamente 3% dos casos, cardiomiopatia dilatada não isquêmica em 2% dos casos e amiloidose em menos de 5% dos casos,^595 , 604^ sendo abordados em tópicos específicos desta Diretriz.

Assim, a excelente performance da RMC em realizar o diagnóstico preciso das áreas de infarto relacionadas à MINOCA faz com que a RMC seja considerada na investigação diagnóstica com classe de recomendação I e nível de evidência B^609 , 610^ ( Tabela 29 ).

**Tabela 29:** Diagnóstico diferencial de troponina positiva com coronárias não obstrutivas (TP-NOCA/MINOCA)

Indicação	Classe de recomendação	Nível de evidência
Diagnóstico diferencial de síndromes de elevação de troponina com coronárias não obstrutivas^611 – 613^	I	B

### 3.6. Cardiomiopatia Induzida por Estresse (Takotsubo)

A cardiomiopatia de Takotsubo (cardiomiopatia de estresse, síndrome do coração partido) foi descrita inicialmente por autores japoneses em 1990.^614^ É mais frequente em mulheres com idade acima de 55 anos, que apresentam quadro de dor torácica e alterações eletrocardiográficas semelhantes ao IAM, associados a disfunção segmentar médio apical do VE de padrão característico.^615 , 616^ Classicamente, não apresenta obstrução coronariana significativa e é habitualmente precedida de importante estresse emocional ou físico.^617^ Mais recentemente, entre as alterações cardíacas detectadas em pacientes com covid-19, alterações semelhantes ao Takotsubo foram descritas.^618 , 619^

Os critérios diagnósticos atualmente aceitos (InterTAK Diagnostic Criteria) incluem disfunção ventricular reversível com balonamento apical, histórico de estresse emocional ou físico, alterações eletrocardiográficas e biomarcadores moderadamente elevados (desproporcionalmente à disfunção ventricular).^619^ A presença de coronariopatia obstrutiva não é um fator excludente já que pode existir a sobreposição das duas etiologias, e a presença de miocardite ou de lesão miocárdica irreversível (infarto) deve ser excluída.^609^

A RMC tem sua utilidade na avaliação da suspeita de síndrome Takotsubo pela sua capacidade de avaliação da função global e segmentar associada à caracterização tecidual.^620 , 621^ O protocolo deve incluir técnicas de cinerressonância, imagem de sangue escuro ponderada em T2 e RT. Quando disponível, técnicas de mapas paramétricos (mapa T1 nativo, VEC e mapa T2) podem ser úteis na identificação de alteração miocárdica em segmentos normais pela avaliação visual.^622^

A presença de disfunção segmentar característica associada a dano miocárdico reversível (edema) são achados bastante sugestivos para o diagnóstico. Habitualmente, não há RT na sua forma clássica, mas há descrição de impregnação menos intensa pelo meio de contraste e de forma reversível na fase hiperaguda.^620 , 621^

A RMC também é útil para identificar outras doenças que devem ser excluídas para o diagnóstico de Takotsubo, como infarto miocárdico e miocardite, assim como a presença de fatores complicadores como trombos intracavitários.^620^

Em resumo, a RMC é útil na avaliação inicial dos casos suspeitos de Takotsubo, podendo contribuir na detecção de critérios diagnósticos e na diferenciação de outras doenças com quadro clínico semelhante.^623^ Os cenários clínicos relacionados à sua utilidade encontram-se descritos na Tabela 30 .

**Tabela 30:** Utilização da ressonância magnética cardíaca em cardiomiopatia induzida por estresse (Takotsubo)

Indicação	Classe de recomendação	Nível de evidência
Na avaliação de suspeita diagnóstica de cardiomiopatia de Takotsubo (incluindo avaliação da função ventricular global e segmentar)^609 , 624^	I	B
Avaliação de trombo apical em pacientes com diagnóstico de cardiomiopatia de Takotsubo^621 , 625^	I	B
Avaliação de suspeita diagnóstica de cardiomiopatia de Takotsubo, após ecocardiograma com imagens inadequadas/janela acústica limitada^621 , 626^	I	C

### 3.7. Miocardites/Cardiomiopatias Inflamatórias

A miocardite é uma doença inflamatória do músculo cardíaco que pode ocorrer como consequência de infecção, exposição a substâncias tóxicas ou ativação do sistema imune.^627^ A etiologia infecciosa viral é a mais prevalente, sendo o quadro clínico bastante variável (desde indivíduos assintomáticos até morte súbita) e, em geral, cursa com dor precordial, dispneia, fadiga, palpitações e síncope.^628^ Alterações eletrocardiográficas estão presentes em 85% dos casos, com elevação do segmento ST, alargamento do QRS ou arritmias associadas à elevação de marcadores de necrose miocárdica (troponina ultrassensível).^627^

O diagnóstico de miocardite inclui a associação de quadro clínico, exame físico e exames laboratoriais e de imagem. A RMC auxilia no diagnóstico, sendo um exame sensível às alterações teciduais que ocorrem pela inflamação do miocárdio.^419 , 437^ Os critérios de Lake Louise foram atualizados em 2018, associando as técnicas de mapas paramétricos e VEC, o que aumentou a acurácia diagnóstica. A inflamação miocárdica aguda pode ser detectada se ao menos um critério de cada categoria estiver presente.^437^ Uma das categorias é o edema miocárdico por meio de imagens ponderadas em T2 ou mapa de T2, e a outra categoria é a injúria miocárdica por meio de RT, aumento do T1 nativo ou do VEC.^437 , 629^ O mapa de T2 se encontra mais elevado na fase aguda da miocardite e tende a normalizar ao longo dos meses, sendo um recurso útil tanto no diagnóstico quanto no monitoramento do tratamento.^630^ O tempo de relaxamento T1 se prolonga por edema intracelular ou extracelular, hiperemia e devido à presença de áreas de fibrose, e o VEC pode aumentar em decorrência da expansão do meio extracelular pela inflamação.^437 , 630^ Se ambos os critérios forem positivos, aumentam a especificidade diagnóstica. Por outro lado, se apenas um deles estiver presente em um cenário de suspeição clínica, permite auxiliar no diagnóstico.^437^ Na ausência de RT e quadro clínico positivo, a presença de alteração nos mapas de T1 nativo e VEC pode ser indicativa de injúria miocárdica. Nessa situação, a elevação do T1 nativo em áreas sem RT mostrou elevação na sensibilidade do método sem aumentar falsos-positivos.^628^

Além da utilidade diagnóstica, a RMC também pode ser usada para prognóstico. Nesse contexto, a disfunção biventricular resultante de envolvimento miocárdico significativo é o maior preditor de mortalidade. A presença de RT também é preditor de mortalidade, estando mais relacionada ao risco de morte súbita e à evolução com dilatação ventricular esquerda e queda de fração de ejeção (FE).^628^ Os pacientes que apresentam FE ≤ 40% associada a RT positivo apresentam um aumento do risco de evento cardiovascular desfavorável em 10% ao ano.^628^

O SARS-CoV-2, vírus causador da covid-19, tem sido frequentemente associado à injúria miocárdica. No seguimento dos pacientes, o achado mais comum é a disfunção diastólica (55%), e apenas 2,8% apresentaram função ventricular esquerda reduzida. Na fase aguda da doença, os achados de RMC mais comuns são alterações nos mapas de T1 e T2, alterações pericárdicas (miopericardite) e padrões de RT não coronariano. Os pacientes em fase de convalescença da doença que apresentaram quadros moderados a graves costumam apresentar alterações na RMC, sendo mais frequente o RT pericárdico e miocárdico (principalmente subepicárdico e mesocárdico) e a discreta redução da função sistólica biventricular em relação aos grupos-controle. Adicionalmente, observou-se elevação dos mapas de T1 e T2 nos pacientes infectados. Os pacientes com sintomas leves ou assintomáticos não apresentaram alterações significativas em relação ao controle.^631^

Atualmente, a realização de RMC na suspeita de miocardite é indicação classe I na investigação diagnóstica e prognóstica de miocardite aguda, crônica e/ou suspeita de miocardite prévia ( Tabela 31 ). A incorporação recente dos dados de mapas paramétricos T1 e T2 e VEC elevam a sensibilidade do método.

**Tabela 31:** Uso da ressonância magnética cardíaca (RMC) em diferentes cenários de investigação de miocardites/cardiomiopatias inflamatórias

Indicação	Classe de recomendação	Nível de evidência
RMC na avaliação da função, geometria e morfologia ventricular na suspeita de miocardite aguda, subaguda e crônica^371 , 437 , 439 , 479^	I	B
RMC na investigação diagnóstica e prognóstica de miocardite aguda, crônica e/ou suspeita de miocardite prévia^371 , 437 , 439 , 479^	I	B
RMC no acompanhamento de 4 a 12 semanas do episódio agudo, para diferenciação de evolução complicada *versus* não complicada^371 , 437^	IIa	B
RMC na miocardite fulminante com instabilidade hemodinâmica^371 , 437^	III	B

### 3.8. Coração de Atleta

A síndrome do coração do atleta pode ser observada geralmente em atletas de alta performance e dependendo do estímulo referente ao exercício, podendo levar a um aumento dos volumes e diâmetros do coração, aumento da massa ventricular ou uma combinação dessas alterações.^632 – 635^ Esses achados podem ser avaliados pelo ecocardiograma na maioria das vezes, porém a RMC pode ser útil naqueles em que a ecocardiografia não foi capaz de fazer o diagnóstico.^636 – 640^

A diferenciação entre os achados observados no coração de atletas com cardiopatias é fundamental em alguns casos e pode evitar consequências importantes relacionadas a falhas nesses diagnósticos. A morte súbita em atletas pode ocorrer em indivíduos aparentemente saudáveis, sem diagnóstico prévio de alterações cardiovasculares.^636 , 641^

Formas iniciais de CMH, cardiomiopatia dilatada e não compactação ventricular esquerda/trabeculação excessiva podem fazer o diagnóstico diferencial com o coração de atleta,^642^ porém outras doenças associadas como cardiomiopatia arritmogênica, miocardite e até mesmo DAC quando não diagnosticadas podem aumentar o risco de desfechos desfavoráveis.^638^

A RMC é considerada padrão-ouro para a quantificação dos volumes cavitários e função biventricular, e os valores de referência normais já foram publicados para a população geral.^581 , 643^ Em revisão sistemática mais recente^18^, foram publicados valores de referência para atletas masculinos. De acordo com o tipo de esporte com maior componente de resistência ou força muscular, foi observada variação do volume diastólico final e da massa ventricular entre os atletas.

A diferenciação entre o coração de atleta para uma cardiopatia em fase inicial nem sempre é fácil, e as medidas mais precisas fornecida pela RMC podem auxiliar nesse sentido. Como subsídio adicional ao diagnóstico, a utilização da técnica do RT para avaliação da fibrose pode ajudar nesse processo. No diagnóstico diferencial entre coração de atleta e CMH, não se espera a ocorrência de fibrose miocárdica na primeira condição, sendo relativamente comum em portadores de CMH (embora seja descrita a ocorrência de fibrose miocárdica em alguns maratonistas e triatletas).^644 , 645^ Outros dados que podem auxiliar nos diagnósticos são o tipo geométrico da hipertrofia, a obstrução de via de saída do VE e o remodelamento do átrio esquerdo desproporcional ao remodelamento do VE. Essas características, quando presentes, contribuem para o diagnóstico de CMH. Em alguns casos, somente a interrupção do treinamento com avaliação evolutiva dos parâmetros como volumes, massas e FE vão poder permitir essa distinção.^646^ A maioria das alterações cardiovasculares parecem regredir após descondicionamento do exercício em 9 a 12 semanas, porém, em 20% dos casos, a dilatação do VE pode persistir.^647^

Os mapas paramétricos podem ajudar no diagnóstico diferencial do coração do atleta para outras cardiopatias. O mapa T1 e a fração de VEC geralmente estão mais baixos do que observamos em outras cardiopatias,^648^ porém são necessários mais trabalhos para melhor avaliação dessas técnicas nesses cenários.

A RMC permite medir parâmetros do coração de forma mais precisa que outros métodos, tornando o método muito apropriado para a avaliação do coração do atleta^648^ e para o diagnóstico diferencial com diversas cardiopatias. Sua recomendação na pesquisa das alterações da morfologia cardíaca em atletas é apresentada na Tabela 32 .

**Tabela 32:** Emprego da ressonância magnética na pesquisa de coração de atleta

Indicação	Classe de recomendação	Nível de evidência
Diagnóstico diferencial de alterações da morfologia cardíaca (dilatação, hipertrofia ventricular, trabeculações acentuadas) em atletas^646 , 649 – 651^	I	B

### 3.9. Cardiomiopatia Hipertrófica

A CMH é uma doença hereditária caracterizada por uma mutação genética e progressão espontânea da hipertrofia, pela ausência de estresse hemodinâmico associado, bem como pela inexistência de doenças sistêmicas relacionadas a depósito miocárdico (por exemplo, amiloidose cardíaca). Tem como potenciais complicações morte súbita, obstrução de via de saída do VE, IC e acidente cerebrovascular tromboembólico, sendo considerada a principal causa de morte súbita em atletas jovens. Apesar disso, a utilização de tratamentos convencionais, assim como o uso de desfibriladores implantáveis nos pacientes com maior risco, pode diminuir a mortalidade a menos de 1% ao ano.^652 – 656^

O diagnóstico de CMH é feito pelo ecocardiograma ou pela RMC quando, na diástole máxima, a espessura ventricular esquerda ultrapassa 15 mm na ausência de outras causas que justifiquem esse achado.^657^ Em pacientes com testes genéticos positivos ou em familiares de pacientes com CMH, a espessura do VE entre 13 a 14 mm pode ser considerada para o diagnóstico.^658 – 660^

Habitualmente, o aumento da espessura do VE se dá junto ao septo basal e parede anterior do VE. Entretanto, variações de localização e distribuição, mesmo sem aumento da massa miocárdica total, podem ocorrer de maneira variável. Outras alterações morfológicas também podem ocorrer, como hipertrofia apical, criptas miocárdicas, inserção anômala do músculo papilar, alongamento dos folhetos da válvula mitral e hipertrofia ventricular direita.^653 , 661^

A RMC é fundamental na avaliação diagnóstica dos pacientes com CMH, sobretudo naqueles em que o ecocardiograma apresentou alguma limitação (por exemplo, dificuldade de janela ecocardiográfica) ou quando há dúvida na indicação de desfibrilador implantável pela estratificação de risco de eventos arrítmicos.^419^

A fibrose miocárdica também ocorre de forma variável em pacientes com CMH, com padrão de fibrose miocárdica multifocal (algumas vezes podendo simular o padrão de fibrose miocárdica decorrente de infarto do miocárdio). A presença de síncope, taquicardia ventricular não sustentada, história familiar de morte súbita, queda da pressão arterial durante o exercício e afilamento do VE são fatores de risco independentes para a morte súbita nessa doença.^653 , 661 , 662^

A identificação de marcadores de risco como aneurismas apicais, a diminuição da função sistólica e a quantificação da fibrose miocárdica pela técnica do RT também fazem com que a RMC seja cada vez mais importante nessa avaliação. A quantificação da fibrose miocárdica torna o método essencial em muitos pacientes com CMH.^663 , 664^

Os principais critérios observados pela RMC na identificação de pacientes com pior prognóstico na CMH são: 1) FEVE menor que 50%; 2) espessura ventricular esquerda maior que 30 mm; 3) presença de aneurisma apical; e 4) fibrose miocárdica pela técnica do RT com extensão maior que 15% da massa do VE (quantificada por *software* ou estimada visualmente).^664 – 666^

A Tabela 33 traz as principais características anatômicas relacionadas a apresentação de CMH observadas pela RMC.

**Tabela 33:** Características observadas pela ressonância magnética cardíaca em pacientes com cardiomiopatia hipertrófica

Característica	Observações	Critério de mau prognóstico
Volumes cavitários	Geralmente diminuídos	
Obstrução de via de saída do ventrículo esquerdo	Geralmente observado pela cinerressonância em repouso	
Espessura	Acima de 15 mm (ou acima de 13-14 mm em pacientes com outros fatores diagnóstico)	Acima de 30 mm
Local do aumento da espessura	Geralmente localizada no septo basal do ventrículo esquerdo, mas com grande variabilidade	Pode levar à obstrução de via de saída do ventrículo esquerdo
Fração de ejeção	Muitas vezes acima de 80%	Abaixo de 50%
Volume do átrio esquerdo	Muitas vezes aumentado	Maior chance de fibrilação atrial
Presença de aneurisma apical	Associado ou não a fibrose miocárdica	Sinal de pior prognóstico
Realce tardio	Presença de realce tardio com padrão variado	Fibrose miocárdica > 15%

A RMC é uma ferramenta importante na suspeita de CMH e vem se tornando fundamental para a estratificação da morte súbita, sobretudo na caracterização tecidual e estimativa do grau de fibrose miocárdica. Os níveis de evidências e as classes de recomendação da RMC em diferentes cenários clínicos estão descritos na Tabela 34 .

**Tabela 34:** Utilização da ressonância magnética cardíaca em suspeita de cardiomiopatia hipertrófica

Indicação	Classe de recomendação	Nível de evidência
Suspeita de cardiomiopatia hipertrófica em pacientes com ecocardiograma inconclusivo ou com dados clínicos/eletrocardiográficos conflitantes mesmo com ecocardiografia normal^640 , 667 – 670^	I	A
Diagnóstico diferencial de hipertrofia ventricular esquerda^667 , 669 – 672^	I	A
Pesquisa de fibrose miocárdica para estratificação de risco em portadores de cardiomiopatia hipertrófica^669 – 673^	I	A
Pacientes com obstrução da via de saída do ventrículo esquerdo, inconclusiva ao ecocardiograma^669 , 670 , 674 – 676^	IIa	B

### 3.10. Endomiocardiofibrose

A endomiocardiofibrose (EMF) é uma doença de etiopatogenia pouco definida e atribuída a fatores como infecções e parasitose, desencadeando eosinofilia e aumento de imunoglobulina E (IgE) quando associados a susceptibilidade genética. Sua forma tropical é considerada endêmica nos países em desenvolvimento, incluindo o Brasil,^677^ enquanto a endocardite de Loeffler, forma tardia da síndrome hiperosinofílica, é mais comum em países com clima temperado. A doença se manifesta com obliteração apical dos ventrículos por conteúdo fibroso, com ou sem calcificação e trombo local, associada a disfunção diastólica e aumento atrial, podendo haver regurgitação valvar associada. O acometimento pode ser biventricular ou ventricular direito e esquerdo isolados, com essas prevalências ainda controversas na literatura, dependendo da metodologia utilizada.^678 , 679^

A RMC, devido à sua capacidade de caracterização tecidual, destaca-se como exame fundamental no diagnóstico de EMF. Através das imagens de cine-RM, pode-se confirmar os achados morfofuncionais já eventualmente vistos à ecocardiografia. É fundamental a distinção entre outras patologias que fazem diagnóstico diferencial, principalmente CMH em sua forma apical, além de doenças que podem eventualmente acometer o ápice, como aneurismas com trombo (secundários a CMH, doença de Chagas ou infarto) e tumores.^680^

No entanto, a confirmação diagnóstica e a diferenciação com outras patologias se dá primordialmente através da caracterização tecidual pelo RT. O RT classicamente descrito na EMF é subendocárdico, acometendo ápice de VE e/ou VD, podendo se estender para a via de entrada, sendo que as vias de saída são geralmente poupadas.^679^ Um achado clássico descrito por um grupo brasileiro denominado "sinal do duplo-V"^681^ caracteriza-se por uma tripla camada, composta por miocárdio normal, endomiocárdio com RT e uma terceira camada com hipossinal, consistindo de trombo com ou sem calcificação. Esses achados tiveram excelentes valores de diagnóstico e prognóstico^679^ ( Tabela 35 ). A Tabela 36 apresenta os cenários clínicos relacionados ao emprego da RMC em suspeita de EMF.

**Tabela 35:** Características morfológicas e teciduais da endomiocardiofibrose pela RM cardíaca

**Achados morfofuncionais (em uma ou em duas cavidades ventriculares)** - Obliteração apical - Redução dos volumes ventriculares (menor que 57 mL/m² ± 15 para VE e menor que 56 mL/m² ± 30 para VD), embora possa haver aumento compensatório de diâmetro de porção basal.^678^ - Fração de ejeção normal ou levemente reduzida. - Aumento atrial. - Regurgitação valvar (mitral e/ou tricúspide), com ou sem alteração da anatomia dos músculos papilares, como fusão e aderência às paredes ventriculares.
**Alterações teciduais** - Realce tardio subendocárdico, não relacionada a território coronariano e acometendo principalmente ápice ventricular. - Sinal do duplo V (com trombo e/ou calcificação apical) ou V único (apenas fibrose).
**Valor prognóstico** - Volume quantificado de tecido fibrótico maior que 19 mL/m².^679^

RM: ressonância magnética; VE: ventrículo esquerdo; VD: ventrículo direito.

**Tabela 36:** Utilização da ressonância magnética cardíaca na avaliação de pacientes com suspeita de endomiocardiofibrose

Indicação	Classe de recomendação	Nível de evidência
Suspeita de endomiocardiofibrose^419 , 679^	I	B
Diagnóstico diferencial de hipertrofia apical^419 , 667 , 670 , 671^	I	B

### 3.11. Amiloidose Cardíaca

A amiloidose é uma doença infiltrativa que acomete o miocárdio pelo depósito intersticial de proteínas, sendo as mais comuns a de cadeia leve (AL) de imunoglobulinas e a secundária ao acúmulo da transtirretina anômala (ATTR) (tanto a forma hereditária quanto a forma selvagem, ou *wild type* ).^682^ O acometimento cardíaco pode ocorrer em diferentes estágios da doença e tem implicância prognóstica. O tratamento específico é totalmente diferente, sendo bastante relevante a distinção entre as duas formas. Para o diagnóstico, as últimas recomendações das diretrizes é buscá-lo de maneira não invasiva com exames de imagem, reservando o diagnóstico por biópsia cardíaca para casos de exceção. A RMC tem grande aplicabilidade clínica no diagnóstico dessa patologia, em conjunto com a cintilografia miocárdica com pirofosfato na medicina nuclear.

O primeiro aspecto da RMC é no diagnostico diferencial etiológico da amiloidose cardíaca dentro das síndromes restritivas ou em outras etiologias que levam a uma hipertrofia miocárdica.^683^ Para isso, os protocolos de ressonância usualmente incluem diversas técnicas para fazer o diagnóstico diferencial da amiloidose.^684^ A começar pela avaliação da morfologia e função, já se observa uma hipertrofia que pode ser concêntrica ou mesmo assimétrica dos VE e VD, sendo mais pronunciado nos casos de ATTR. O acometimento do septo atrial é bem conhecido, e a distinção entre hipertrofia e degeneração lipomatosa pode ser realizada pela ressonância. O depósito de RT tem característica típica na amiloidose, sendo inicialmente subendocárdico difuso, mas podendo acometer todo o miocárdio em aspecto transmural em casos mais avançados.^685^ O RT, além de informações diagnósticas, também indica o prognóstico da doença, sendo o grau de depósito proporcional à sobrevida livre de eventos.^686^

Mais recentemente, o uso de técnica de T1 nativo e o cálculo de VEC vêm também sendo aplicados no diagnóstico da doença, especialmente no caso do T1 nativo em pacientes que não podem utilizar contraste.^687^ O T1 nativo está fortemente aumentado em casos de amiloidose, e o VEC tem se mostrado como um marcador prognóstico adicional, mesmo em pacientes com graus semelhantes de RT.

Entre as principais indicações da RMC para amiloidose, encontra-se a utilização em pacientes com hipertrofia miocárdica usualmente encontradas na ecocardiografia, mas cujo diagnóstico etiológico é incerto ou duvidoso.^688^ Em pacientes já com o diagnóstico de amiloidose em que se quer determinar o envolvimento ou não do miocárdio, pode-se utilizar a RMC mesmo antes do desenvolvimento fenotípico da hipertrofia. Finalmente, para determinação prognóstica e seleção de terapêuticas mais especificas, o uso da ressonância pode também ser útil na rotina clínica. Especialmente nos casos nos quais a cintilografia miocárdica com pirofosfato tem fraca ou nenhuma captação e os exames hematológicos são positivos, o uso da ressonância se faz ainda mais necessário para a suspeita diagnóstica de amiloidose tipo AL.

A Tabela 37 relaciona-se ao emprego da RMC na avaliação diagnóstica de pacientes com suspeita de amiloidose cardíaca.

**Tabela 37:** Emprego da ressonância cardíaca na avaliação de amiloidose cardíaca

Indicação	Classe de recomendação	Nível de evidência
Para o diagnóstico diferencial de hipertrofia ventricular em pacientes com suspeita clínica e/ou laboratorial e/ou de exames de imagem prévios para amiloidose cardíaca^667 , 671 , 689 , 690^	I	A
Pacientes com exames conflitantes para diagnóstico de amiloidose cardíaca^691 – 693^	IIa	B
Seguimento prognóstico e terapêutico de casos confirmados de amiloidose cardíaca^690 , 691 , 694 – 696^	IIa	B
Para confirmação de diagnóstico de amiloidose cardíaca AL em pacientes com alteração hematológica compatível e suspeita clínica^690 , 691 , 697^	IIa	C

### 3.12. Hemossiderose Cardíaca

O diagnóstico de ferro miocárdico e hepático tem aplicabilidade clínica direta em doenças em que ocorrem transfusões de repetição e/ou há um aumento da absorção intestinal de ferro por mecanismos patológicos. Entre as patologias principais, estão incluídas talassemias, síndromes mielodisplásicas e hemocromatose hereditária.^698^ A RM é uma ferramenta única para esse diagnóstico, e não temos substituto clínico não invasivo para a quantificação de ferro tecidual por imagem nessas doenças. A ferritina sérica é um marcador indireto e pouco correlacionado com a sobrecarga miocárdica de ferro, e a biópsia invasiva é limitada apenas ao diagnóstico hepático.

Para a realização da RM para este fim, não é necessário o uso de contraste, e a técnica mais conhecida e validada é o mapa paramétrico de T2*. Essa técnica foi validada em estudos multicêntricos e mostrou acurácia bastante precisa com a validação por biópsia miocárdica direta.^699^ A partir da medida do T2* obtém-se um valor em milissegundos, que pode ser convertido em concentração de ferro tanto no fígado quanto no coração, utilizando-se *softwares* de pós-processamento comerciais ou ferramentas gratuitas e disponibilizadas on-line.^700^ A obtenção da quantificação ao menos do fígado e coração se dá pelos diferentes ritmos de absorção de ferro nesses dois órgãos, por mecanismos distintos, fazendo com que usualmente haja disparidade entre o acúmulo de ferro entre eles.^701^

O T2* cardíaco tem correlação direta não só com o diagnóstico quantitativo da sobrecarga de ferro cardíaca, mas também traz informações prognósticas e pode ser usado para monitorização terapêutica da quelação de ferro.^702^ As diretrizes clínicas nacionais e internacionais preconizam o uso da ressonância com T2* a partir de 7 a 10 anos de idade e sua repetição anual, podendo esse intervalo variar entre 6 e 24 meses conforme o estado clínico do paciente e sua carga transfusional e sobrecarga inicial.^471^ Valores miocárdicos acima de 20 ms (< 1,16 mg/g de concentração de ferro miocárdico) estão correlacionados com melhor prognóstico e são considerados de baixo risco para IC. Valores < 10 ms (> 2,7 mg/g) indicam pior prognóstico e risco aumentado para IC e arritmias (parâmetros válidos para aparelhos de 1,5 T).

Alguns estudos mais recentes sugerem que a quantificação de ferro tecidual possa também ser feita utilizando-se mapas paramétricos de T1 e que essa medida, inclusive, pode ser mais sensível à sobrecarga de ferro em pequenas quantidades.^448^ Porém, pela menor disponibilidade e padronização do método, a técnica é ainda pouco utilizada na rotina clínica, devendo ser aplicada apenas na impossibilidade de realização do T2* por indisponibilidade da técnica. A Tabela 38 traz as principais indicações da RM cardíaca no manejo do depósito de ferro miocárdico.

**Tabela 38:** Utilização da ressonância magnética cardíaca no diagnóstico e manejo de portadores de sobrecarga de ferro miocárdico

Indicação	Classe de recomendação	Nível de evidência
Cardiomiopatia siderótica, especialmente secundária à talassemia^426 , 473 , 699^	I	A
Avaliação diagnóstica de ferro hepático e miocárdico por técnica de T2* quantitativa^426 , 427 , 473 , 703^	I	A
Seguimento evolutivo para monitorização terapêutica de sobrecarga de ferro por técnica de T2* quantitativa^474 , 703 , 704^	I	A
Diagnóstico e seguimento evolutivo de sobrecarga de ferro por técnica de T1^705^	IIb	C

### 3.13. Outras Doenças de Depósito Miocárdico

#### 3.13.1. Doença de Anderson-Fabry

A doença de Anderson-Fabry é um distúrbio lisossômico autossômico recessivo ligado ao cromossomo X, caracterizado por deficiência de alfa-galactosidase A e acúmulo progressivo de glicoesfingolipídeos complexos, predominantemente globotriaosilceramida (Gb3), nas células musculares, endoteliais e musculares lisas.^706^ Tal acúmulo no miocárdio, valvas e sistema de condução cardíaco leva a um aumento na espessura da parede ventricular, espessamento dos folhetos valvares e arritmias.^707 , 708^ Seu diagnóstico é difícil, pois suas características morfológicas e clínicas podem mimetizar outras doenças que cursam com hipertrofia, como a CMH e amiloidose.

A RMC pode ter um papel importante na avaliação de pacientes com hipertrofia do VE sem etiologia definida. O mapeamento T1 é uma técnica útil para essa finalidade, e valores baixos do T1 nativo podem caracterizar depósito de glicoesfingolipídeos precedendo o desenvolvimento de hipertrofia parietal.^709^

A sequência de RT geralmente demonstra padrão de realce mesocárdico predominantemente no segmento inferolateral basal do VE, poupando a região subendocárdica.^710 , 711^

#### 3.13.2. Doença de Depósito de Glicogênio

As doenças de depósito de glicogênio são doenças hereditárias do metabolismo do glicogênio que podem afetar sua síntese ou degradação nos tecidos musculares, hepáticos e cardíaco.^712^

A **doença de Danon** é de caráter autossômico dominante ligada ao cromossomo X devido à deficiência da enzima LAMP2 e com a tríade de IC com fenótipo de CMH, miopatia esquelética e retardo mental em pacientes do sexo masculino e apenas miocardiopatia em mulheres.^712^ O fenótipo da miocardiopatia em geral é hipertrófico, mas há também descrito o dilatado. A miopatia em geral é leve, com fraqueza proximal dos músculos dos membros e estudos de condução nervosa mostram polineuropatia sensorial e motora.^713^

A RMC demonstra fenótipos de hipertrofia massiva (podendo chegar até 4 cm) e em sua grande maioria concêntrica. Alguns casos podem ter hipertrofia assimétrica ou até padrão dilatado.^713 , 714^ O RT demonstra padrão extenso e usualmente subendocárdico e mesocárdico, com áreas praticamente transmurais. A preservação do septo é uma característica de imagem típica, observada em 88% dos pacientes, enquanto a parede lateral e o RT apical estão presentes em quase todos os pacientes.^10^ Os valores do T1 nativo e VEC são elevados e correspondem às áreas de RT.^713^

A **síndrome PRKAG2** é uma doença hereditária autossômica dominante rara, que pode ser similar à doença de Danon, porém a ausência de doença sistêmica, função hepática normal e níveis séricos de creatinoquinase normais devem alertar para o diagnóstico.^714^ A apresentação clínica é de hipertrofia ventricular e taquiarritmias que podem levar a morte súbita, doença do tecido de condução, hipertrofia miocárdica severa, miopatia esquelética e arritmias, frequentemente relacionadas com síndrome de Wolff-Parkinson-White (WPW).^714^

A hipertrofia cardíaca acomete principalmente o VE e tem caráter progressivo acompanhado de disfunção sistólica e diastólica. Alta voltagem nos complexos QRS com anormalidades de repolarização ventricular é observada mesmo na ausência de hipertrofia ventricular esquerda (HVE) ao ecocardiograma.^715 , 716^

A Tabela 39 traz a indicação da utilização da RMC no contexto de doenças de depósito miocárdico.

**Tabela 39:** Utilização da ressonância magnética cardíaca em patologias específicas que cursam com depósito miocárdico.

Indicação	Classe de recomendação	Nível de evidência
Investigação de acometimento cardíaco por patologias específicas que cursam com depósito miocárdico (Danon, Anderson-Fabry etc.)^717^	I	B

### 3.14. Cardiomiopatia Chagásica

A cardiomiopatia chagásica (CC) é a manifestação mais comum e severa da doença de Chagas. A sua patogênese deve ser entendida como uma complexa interação entre o hospedeiro e o parasita e como uma doença multifatorial. O *T. Cruzi* está presente em biópsias com evidências de inflamação moderada a importante. Além das lesões de origem inflamatória, alterações microvasculares (por exemplo, microespasmos, trombos, ativação plaquetária, disfunção endotelial e roubo de fluxo) são também descritas como causadoras dos danos miocárdicos.^718 , 719^

A RMC vem se destacando como uma ferramenta importante na estratificação de risco e prognóstico em portadores de CC, cujos achados característicos são descritos na Tabela 40 . Em 2005, Rochitte et al. avaliaram 51 pacientes em estágios diferentes da CC. Fibrose miocárdica foi observada em 68,6% dos casos, incluindo em alguns pacientes da fase indeterminada, principalmente de padrão mesocárdico e subepicárdico nos segmentos inferolateral médio/basal e apical do VE, demonstrando boa correlação da presença e quantificação da fibrose com fatores prognósticos bem estabelecidos, como FEVE e classificação funcional da New York Heart Association (CF-NYHA).^720^ Posteriormente, outras publicações descreveram a presença de fibrose em pacientes na fase indeterminada,^721 – 724^ assim como seu padrão de acometimento e repercussão na função segmentar, notadamente nos segmentos inferolateral médio/basal e na porção apical do VE.^722 , 723^ A disfunção sistólica do VD é mais comumente associada com a disfunção sistólica do VE, embora também possa ser identificada isolada e precocemente.^720^ Algumas publicações demonstraram uma prevalência significativa de RT subendocárdico, reforçando também a hipótese de lesões isquêmicas associadas (tromboembólica/microvascular), além das não isquêmicas (inflamatórias).^721 , 722^

**Tabela 40:** Achados característicos da cardiopatia chagásica crônica pela ressonância magnética

Fibrose miocárdica de padrão heterogêneo (inflamatório/microvascular/isquêmico)
Fibrose miocárdica em parede inferolateral e apical
Disfunção sistólica global e/ou segmentar
Aneurisma vorticilar ("dedo de luva")
Disfunção do ventrículo direito
Inflamação miocárdica crônica
Trombos intracavitários

A utilidade dos mapas paramétricos T1 e VEC começou a ser descrita na avaliação da CC. O VEC apresentou uma AUC ROC semelhante àquela vista pelo RT na predição de taquicardia ventricular não sustentada.^725^ Em outra publicação, a quantidade de fibrose foi o principal preditor de taquicardia ventricular (TV).^726^

A atividade inflamatória do miocárdio na CC crônica foi avaliada pela ressonância com sequências *Spin Echo* ponderado em T2, edema^723^ e o realce global precoce. A confirmação da inflamação ativa na CC crônica pode representar implicações terapêuticas interessantes: a seleção mais efetiva de pacientes para o tratamento etiológico específico e a possibilidade do uso de terapia com imunomoduladores.

A CC é um fator de risco independente para AVC, independentemente da função ventricular e da presença de arritmias cardíacas.^727 , 728^ Disfunção sistólica, defeito segmentar, aneurisma vorticilar apical, fibrose e edema no miocárdio são alterações que determinam maior risco para a formação de trombos intracavitários e são comumente descritos em portadores de CC crônica. Em uma recente publicação sobre AVC criptogênico, a RMC encontrou anormalidades com potencial embólico em mais de um quarto dos pacientes, mudando a conduta em relação ao uso de terapia anticoagulante, quase a metade deles portadores de CC crônica.^729^

A avaliação prognóstica da RMC na CC crônica foi descrita incialmente relacionando a presença e extensão da fibrose miocárdica com fatores de risco consagrados (NYHA, FEVE) e posteriormente com o escore de Rassi.^721 , 724^ Dados de estudos recentes demonstraram que a presença e a extensão de fibrose miocárdica foram fortemente associadas a eventos adversos maiores.^730 , 731^

**Figura 7:**
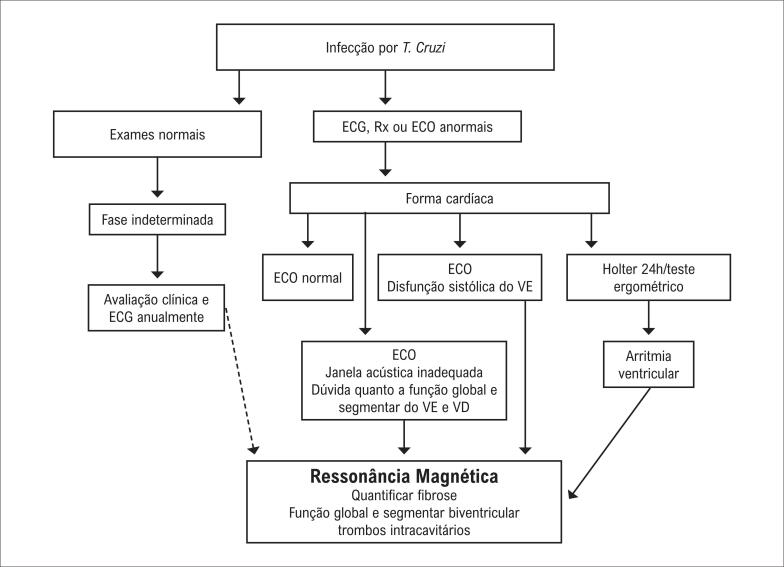
Sugestão de fluxograma diagnóstico para cardiomiopatia chagásica.*Seta tracejada representa indicação condicional. ECG: eletrocardiograma; Rx: raio X; ECO: ecocardiograma; VE: ventrículo esquerdo; VD: ventrículo direito.

Uma sugestão de fluxograma diagnóstico em pacientes com CC encontra-se ilustrado na Figura 7 .

A Tabela 41 traz as principais indicações da RMC no diagnóstico e na estratificação prognóstica em portadores de CC.

**Tabela 41:** Emprego da ressonância magnética cardíaca em pacientes com cardiomiopatia chagásica

Indicação	Classe de recomendação	Nível de evidência
Avaliação da função sistólica biventricular (global e segmentar) em portadores de cardiomiopatia chagásica^732 , 733^	IIa	B
Pesquisa de trombos intracavitários em pacientes chagásicos portadores de acidente vascular cerebral criptogênico^734 , 735^	I	B
Pesquisa de fibrose e/ou edema miocárdico em pacientes com a forma indeterminada^720 , 730 , 736^	IIa	C
Pesquisa de fibrose miocárdica para estratificação de risco em pacientes com o diagnóstico de cardiomiopatia chagásica^724 , 730 , 731^	I	B

### 3.15. Displasia/Cardiomiopatia Arritmogênica do Ventrículo Direito (D/CAVD)

A displasia/cardiomiopatia arritmogênica do VD (D/CAVD) é uma doença que acomete preferencialmente o VD e está associada a arritmias ventriculares, por vezes fatais.^737 , 738^ A D/CAVD nem sempre acomete o VD de forma isolada, e existem evidências de que o VE pode estar envolvido ou até ser predominante.^739^

Essa doença pode ser causa de até 10% dos casos de morte súbita em pacientes jovens.^740^ Os sintomas mais frequentemente observados são palpitações e síncopes e se manifestam entre a segunda e quinta décadas de vida.^737 , 738^ Os desmossomos, complexos multiproteicos estruturais da membrana celular, importantes na manutenção da interação entre células e no processo de sinalização, estão possivelmente envolvidos na etiologia da doença.^737 , 741 , 742^ Uma vez diagnosticada e tratada corretamente, considerando-se inclusive o uso de CDI, a mortalidade é relativamente baixa.^737^

As alterações morfofuncionais características dessa entidade traduzem-se em um VD com volumes aumentados, apresentando dilatações focais (também chamadas de aneurismas) ao longo da parede anterior do infundíbulo, da parede subvalvar inferior ou na região apical lateral, pontos também conhecidos como "triângulo da displasia".^737 , 742^ Parece haver uma perda gradual dos miócitos nessas regiões, com substituição por adipócitos e fibroblastos, além de variável infiltração linfocítica.

As anormalidades encontradas pela RMC refletem essas alterações: segmentos acinéticos no VD e aumento do volume ventricular direito e do diâmetro da via de saída do VD. Inicialmente, a infiltração por gordura na parede livre do VD foi considerada um sinal patognomônico.^743^ Entretanto, a infiltração gordurosa tem baixa concordância interobservador (kappa = 0,74) e, atualmente, não é considerada critério diagnóstico para a doença.^744^ A sensibilidade da RMC para o diagnóstico de D/CAVD, considerando-se infiltração gordurosa, dilatação e disfunção regional do VD, foi de 84, 68 e 78%, respectivamente; a especificidade foi de 79, 96 e 94%, respectivamente.^744^

Na fase inicial da doença, descrita como oculta, pode existir fibrose mínima com RT negativo pela RMC, e esses indivíduos podem apresentar arritmias ventriculares sustentadas com potencial risco de morte súbita.^745^ Já a demonstração de alterações fibrogordurosas em ambos os ventrículos, por meio da sequência de RT, mostra-se promissora.^746^

Em 1994, a Força Tarefa Internacional para o Diagnóstico de D/CAVD propôs um critério para o diagnóstico clínico dessa doença.^747^ Entretanto, o limitado conhecimento da doença naquela época resumia os casos principalmente aos pacientes sintomáticos e às vítimas de morte súbita – o extremo de gravidade no espectro da doença. Uma nova revisão desses critérios foi publicada em 2010, baseada em novas evidências sobre os aspectos genéticos da doença, obtidas da ampla investigação dos membros de famílias acometidas.^748^ De acordo com essa revisão, a RMC pode contribuir com informações a respeito da contratilidade e do volume do VD, cujas alterações podem constituir um critério maior para o estabelecimento do diagnóstico de CAVD (acinesia regional ou discinesia ou contração dissincrônica do VD associada ao volume diastólico final indexado pela superfície corpórea ≥ 110 mL/m^2^ em homens ou ≥ 100 mL/m^2^ em mulheres, ou FE do VD ≤ 40%) ou critério menor (acinesia regional ou discinesia ou contração dissincrônica do VD associada a volume diastólico final do VD indexado pela superfície corpórea entre 100 e 110 mL/m^2^ em homens ou entre 90 e 100 mL/m^2^ em mulheres ou FE do VD entre 40 e 45%).

Recentemente, um consenso de especialistas conhecido como "Critérios de Pádua" ampliou a definição da doença para um espectro mais amplo de "cardiomiopatia arritmogênica", incluindo no espectro dois outros fenótipos, como o esquerdo dominante e os fenótipos biventriculares. Nessa nova abordagem, a RMC emergiu como a técnica de imagem de escolha, devido à sua capacidade de análise morfofuncional detalhada e avaliação da caracterização tecidual biventricular. Sendo assim, da mesma forma que os critérios anteriores, os critérios de Pádua também precisam ser validados por estudos clínicos em grandes coortes de pacientes e não necessariamente substituem os anteriores.^749^

A Tabela 42 traz as principais indicações da RMC no diagnóstico de D/CAVD.

**Tabela 42:** Utilização da ressonância cardíaca na suspeita e estratificação de risco de cardiomiopatia arritmogênica

Indicação	Classe de recomendação	Nível de evidência
Avaliação de pacientes com suspeita de D/CAVD^746 , 747 , 750 , 751^	I	B
Estratificação de risco em portadores de arritmia ventricular e/ou alterações de eletrocardiograma, com suspeita de D/CAVD^752 – 754^	I	B
Avaliação de acometimento ventricular esquerdo em pacientes com suspeita ou diagnóstico de D/CAVD^750 , 755 – 757^	I	B
Avaliação de função e volumes ventriculares direitos^746 , 747 , 750^	I	B

D/CAVD: displasia/cardiomiopatia arritmogênica do ventrículo direito.

### 3.16. Sarcoidose

A sarcoidose é uma doença granulomatosa multissistêmica complexa, de etiologia desconhecida e apresentação clínica variada e heterogênea. Caracterizada pela formação de granulomas não caseosos em basicamente qualquer órgão do corpo, tem como principal órgão-alvo os pulmões e linfonodos mediastinais, com comprometimento cardíaco clínico em 5 a 10% dos pacientes.^758 , 759^ No entanto, estudos com autópsia relatam granulomas miocárdicos em 25 a 58% dos pacientes com sarcoidose,^760 – 763^ com o envolvimento cardiopulmonar sendo o principal responsável pela morbimortalidade da doença.

O diagnóstico de sarcoidose cardíaca é complexo, com manifestações cardíacas variadas e inespecíficas dependendo da localização, extensão e estágio do processo inflamatório miocárdico, havendo correlação da extensão das lesões com a gravidade das manifestações. Arritmias cardíacas são a manifestação mais comum, incluindo bloqueios atrioventriculares, arritmias supraventriculares, taquiarritmias ventriculares e eventualmente morte súbita. A IC pode ser a apresentação inicial quando o envolvimento miocárdico é extenso, sendo responsável por até 25% da mortalidade.^764^

Considerado padrão-ouro para o diagnóstico da sarcoidose cardíaca, a biópsia endomiocárdica, entretanto, apresenta baixa acurácia quando não guiada (25%), com recomendação de consenso para a biópsia guiada^764^ e aumento do sucesso diagnóstico para a faixa dos 50%, mesmo em pacientes sintomáticos. Na ausência de confirmação tecidual, as diretrizes recomendam a correlação de envolvimento extracardíaco com evidências de envolvimento cardíaco, principalmente por marcadores de imagem, mesmo sem evidências científicas que corroborem essas recomendações ( Tabela 43 ). Nesse sentido, dois cenários principais se impõem: pacientes com envolvimento extracardíaco definido, com ou sem sintomas cardíacos, e pacientes sem envolvimento sistêmico.

**Tabela 43:** Emprego da ressonância magnética cardíaca em pacientes com suspeita de envolvimento cardíaco por sarcoidose

Indicação	Classe de recomendação	Nível de evidência
Suspeita de envolvimento cardíaco em pacientes com diagnóstico de sarcoidose extracardíaca^768 – 770^	I	B
Avaliação de fibrose miocárdica em pacientes com suspeita de envolvimento cardíaco por sarcoidose (arritmias ventriculares, bloqueios atrioventriculares etc.)^768 , 769 , 771 , 772^	I	B

Os achados da RMC dependem do estágio do processo patológico. Na fase inflamatória aguda, observam-se espessamento da parede miocárdica com alterações da contratilidade, aumento do sinal em T2 (edema) e RT. Na fase crônica, observam-se áreas focais de afilamento miocárdico cicatricial e RT tipicamente no septo basal. O RT pode apresentar aspecto linear subepicárdico, transmural ou nodular, com distribuição heterogênea e padrão tipicamente não vascular. O comprometimento septal do lado do VD e as áreas de junção das paredes ventriculares são considerados característicos.^765 – 767^

A Tabela 43 traz as principais indicações da utilização da RMC em suspeita de casos de sarcoidose.

### 3.17. Não Compactação Ventricular Esquerda/Trabeculação Excessiva do Ventrículo Esquerdo

A não compactação ventricular esquerda, alteração caracterizada por falha embriológica no processo de compactação miocárdica, leva a mudanças da anatomia da parede ventricular caracterizada por excessivas trabeculações e profundos recessos, com apresentação clínica composta de sintomas de IC, fenômenos arrítmicos e tromboembólicos.^773^ O diagnóstico dessa condição é bastante difícil e controverso, devido à presença de trabeculação miocárdica em parte significativa da população saudável, além de outras cardiomiopatias e condições fisiológicas como gravidez.^774^

A RMC surgiu como ferramenta promissora no diagnóstico de não compactação ventricular esquerda. O primeiro critério diagnóstico amplamente difundido foi publicado por Pettersen,^775^ no qual foi atribuído um valor de 2,3 para a razão da espessura de miocárdio não compactado (trabeculado) em relação à espessura do miocárdio compactado, medido na diástole. Posteriormente, surgiram publicações em que foram apresentados critérios como massa trabeculada maior que 20% e relação > 2,0 na sístole.^776^ Esse último critério demonstrou melhor correlação com eventos cardiovasculares futuros. Há ainda estudos demonstrando presença de RT em pacientes com não compactação ventricular esquerda, embora com padrão heterogêneo,^777^ dado que, em conjunto com a FE deprimida, apresenta excelente correlação prognóstica.^778^

Recentemente, um posicionamento de especialistas recomendou a substituição do termo "não compactação ventricular esquerda" para "trabeculação excessiva" do VE. Tal recomendação resultou da falta de evidências que justifiquem o mecanismo embrionário de "compactação" miocárdica. Nesse contexto, a trabeculação excessiva deve ser encarada como uma manifestação fenotípica que pode acompanhar certas condições fisiológicas (por exemplo, gravidez, adaptações fisiológicas ao exercício), bem como cardiopatias congênitas ou adquiridas, e não está necessariamente ligada a alteração prognóstica em adultos.

A Tabela 44 apresenta a utilização da RMC em suspeita de não compactação ventricular esquerda/trabeculação excessiva do ventrículo esquerdo

**Tabela 44:** Utilização da ressonância magnética cardíaca em suspeita de não compactação ventricular esquerda/trabeculação excessiva do ventrículo esquerdo

Indicação	Classe de recomendação	Nível de evidência
Suspeita de não compactação ventricular esquerda/trabeculação excessiva do ventrículo esquerdo^773 , 779 – 781^	I	B

### 3.18. Distrofias Musculares

Distrofias musculares são desordens genéticas que afetam os músculos estriados. São causadas por mutações em genes que codificam várias proteínas responsáveis pela contração muscular, provocando enfraquecimento muscular e perda progressiva da função, podendo acometer também o músculo cardíaco e o sistema de condução do coração.

As distrofias comumente associadas ao envolvimento cardíaco são as distrofinopatias (Duchenne [DMD], Becker [DMB] e pacientes portadoras no gene da distrofina), Limb Girdle, Emery-Dreifuss e distrofia muscular miotônica. A DMD é a mais frequente das distrofias musculares.^782 – 785^

O envolvimento miocárdico nas distrofinopatias é frequente e insidioso,^786 , 787^ acometendo aproximadamente 80% dos pacientes.^788^ Atualmente, representa uma das principais causas de mortalidade (IC e arritmias), como resultado de aumento da sobrevida pela melhora do suporte respiratório (ventilação noturna mecânica não invasiva), cirurgia de estabilização da coluna e tratamento com corticoides.^789 , 790^ O ecocardiograma transtorácico ainda é o teste mais utilizado para diagnosticar o envolvimento cardíaco na DMD, mas apresenta importante limitação diagnóstica devido a pobre janela acústica ocasionada pela progressão da escoliose e obesidade desses pacientes e à limitação própria do método para diagnosticar acometimento cardíaco subclínico.^791^ A avaliação do acometimento cardíaco nas distrofias musculares se fundamenta basicamente nas imagens de cinerressonância e sobretudo na identificação de fibrose miocárdica através da técnica de RT que se apresenta com padrão subepicárdico, e acomete principalmente os segmentos anterolaterais e inferolaterais do VE.^792 – 798^

O diagnóstico precoce e subclínico do envolvimento cardíaco associado às distrofias musculares (DMD e DMB) é importante para proporcionar o tratamento cardioprotetor visando reduzir os efeitos adversos do remodelamento cardíaco e atenuar os sinais da IC, impactando na queda da mortalidade.^799 – 801^

Consensos e atualizações para DMD e DMB sugerem que a avaliação cardíaca deve ser feita desde o diagnóstico dessas mutações.^791 , 802 – 804^ O National Heart, Lung and Blood Institute (NHLBI) publicou atualização sobre o acometimento cardíaco em DMD e considerou a RMC como a modalidade não invasiva de escolha para diagnóstico do acometimento cardíaco precoce, exceto em jovens pacientes que não cooperarem com as manobras necessárias para a realização do exame. O ecocardiograma deve ser realizado até 6 a 7 anos devido à impossibilidade do uso de anestésicos nesses pacientes. Após essa idade, deve ser realizada pelo menos uma ressonância a cada 2 anos e anualmente após os 10 anos.^803^ As mulheres portadoras de DMD e DMB devem ser avaliadas caso apresentem qualquer sintoma de acometimento cardíaco. Por volta de 40 anos, essas pacientes têm demonstrado queda da FE e evidência de fibrose miocárdica.^805^

Em 2017, a AHA^806^ publicou diretrizes para o envolvimento cardíaco nas doenças neuromusculares. Entretanto, não existem consensos para a utilização da ressonância cardíaca no diagnóstico das distrofias musculares de Limb-Girdle, Emery-Dreifuss e distrofia miotônica, devendo o seu uso ser individualizado conforme evolução da doença sistêmica e a manifestação de doença cardiovascular.

A Tabela 45 descreve os principais cenários clínicos relacionados à utilização da RMC no manejo do acometimento cardíaco por distrofias musculares.

**Tabela 45:** Avaliação cardíaca pela ressonância magnética em portadores de distrofias musculares (DMD/DMB)

Indicação	Classe de recomendação	Nível de evidência
Avaliação anual de pacientes sintomáticos^806^	I	B
Avaliação de função ventricular em pacientes com janela ecocardiográfica ruim ou para pesquisa de fibrose miocárdica^797 , 801 , 806^	IIa	B
Avaliação de fibrose miocárdica em assintomáticos com dilatação ou disfunção ventricular esquerda^797 , 801^	IIa	B
Assintomáticos – avaliação bianual até os 10a, e anualmente após os 10a (recomendação controversa quanto a intervalo de tempo) ^806^	IIa	B
Avaliação de fibrose miocárdica para estratificação de risco e indicação de IECA em pacientes com DMD/DMB com função ventricular esquerda preservada^801^	I	B

DMD: distrofia muscular de Duchenne; DMB: distrofia muscular de Becker; IECA: inibidores da enzima conversora de angiotensina.

### 3.19. Cardiomiopatia Periparto

A cardiomiopatia periparto (CMPP) é uma forma de IC rara, porém com significativo impacto na morbidade e mortalidade de gestantes. Pode ser definida como IC no terceiro trimestre da gestação ou até 5 meses após o parto, com exame de imagem demonstrando dilatação e disfunção sistólica do VE (FEVE < 45%), sendo um diagnóstico de exclusão em mulheres sem doença cardíaca prévia conhecida.^720^ Pode ter incidência de 1:300 a 1:4.000 gestações, com bastante variação a depender do país e demografia.^807 , 808^

A RMC é considerada segura durante a gestação, porém o uso de contraste paramagnético (gadolínio) e o seu efeito no feto não é bem estabelecido. Dessa forma, a administração do contraste durante a gestação não é recomendada, especialmente no primeiro trimestre. Em contrapartida, não há restrição ao uso de contraste durante o período de amamentação.^809 , 810^ A RMC é o método de escolha para avaliação de volume e função ventricular, que é um preditor independente de evento na CMPP.^811 , 812^ Também pode ser útil para excluir outras etiologias, como miocardite, doenças infiltrativas e não compactação ventricular esquerda/trabeculação excessiva do ventrículo esquerdo.^813^ O RT na CMPP é de padrão não coronariano (mesocárdico), com prevalência heterogênea descrita na literatura, de 5 a 70% (provavelmente devido aos diferentes intervalos de início dos sintomas e a realização da RMC).^814 – 816^

A Tabela 46 (abaixo) traz os cenários clínicos em que a RMC pode oferecer informações úteis para o diagnóstico e o manejo clínico da CMPP.

**Tabela 46:** Utilização da ressonância magnética cardíaca na avaliação de cardiomiopatia periparto

Indicação	Classe de recomendação	Nível de evidência
Disfunção ventricular sem diagnóstico definido^817 – 819^	IIa	C
Avaliação prognóstica (acometimento do ventrículo esquerdo e avaliação com realce tardio após o parto)^812 , 814 , 817 , 820^	IIa	C

### 3.20. Cardiomiopatia Associada a Doenças Sistêmicas

Diversas doenças sistêmicas afetam o sistema cardiovascular, com apresentações muito variadas e em cenários clínicos desafiadores, muitas vezes com apresentações cardiovasculares frustas em meio ao quadro clínico global. Destacam-se aqui as doenças autoimunes como artrite reumatoide e espondiloartropatias, lupus eritematoso sistêmico, esclerodermia, vasculites sistêmicas como a granulomatose com poliangeite, doença mista do tecido conjuntivo e outras miopatias inflamatórias.^821^ Os mecanismos que levam ao dano miocárdico não estão esclarecidos, com diversos fatores envolvendo interações intracelulares e extracelulares, mutações genéticas, reações autoimunes e moduladores inflamatórios.^822^ As apresentações são muito diversas, com lesões envolvendo miocárdio, valvas, pericárdio, sistema de condução e vasos em geral. Soma-se a isso um risco de aterosclerose aumentado nas doenças reumáticas, não atribuído apenas aos fatores de risco cardiovasculares tradicionais, mas provavelmente relacionado a respostas imunológicas disfuncionais e inflamação crônica, adicionando morbimortalidade nos casos mais avançados.^822^

O papel da RMC nessas condições envolve a avaliação da apresentação específica de cada caso, mas algumas técnicas, principalmente o RT e a demonstração de dano miocárdico irreversível, já foram demonstradas.^823^ A lesão miocárdica direta com infiltrado inflamatório também já foi demonstrada na poliangeíte,^824^ além da presença de isquemia na ausência de lesão coronária no lúpus sistêmico^825^ e também da análise de mapa T1, inclusive no estudo do pericárdio.^826 , 827^ Em consenso recente,^826^ aborda-se o potencial da RMC para avaliação de pacientes com doenças sistêmicas, como a identificação precoce de lesões miocárdicas, a definição de sua natureza, isquêmica ou inflamatória, além da detecção de substratos arritmogênicos.

A Tabela 47 traz as recomendações de utilização da RMC no diagnóstico de cardiomiopatia associada a doenças sistêmicas.

**Tabela 47:** Utilização da ressonância magnética cardíaca no diagnóstico de cardiomiopatia associada a doenças sistêmicas

Indicação	Classe de recomendação	Nível de evidência
Pesquisa de fibrose miocárdica em pacientes com disfunção ventricular e portadores de doenças autoimunes (artrite reumatoide, lúpus eritematoso sistêmico, esclerodermia)^826 , 828 – 830^	I	B
Suspeita de miocardite/pericardite secundária a atividade inflamatória de doença autoimune^830 – 834^	I	B

### 3.21. Alterações Cardíacas Associadas ao Transplante Cardíaco

A doença vascular do enxerto permanece como a principal causa de morbidade e mortalidade tardia após o transplante cardíaco e pode ocorrer em até 50% dos transplantados após 10 anos. Embora o padrão-ouro para o diagnóstico seja a angiografia coronária invasiva, a RMC vem apresentando resultados animadores.^835^

Monitorar a ocorrência de rejeição celular e humoral, principalmente no primeiro ano após o transplante cardíaco, e buscar ativamente as complicações tardias, como doença vascular do enxerto, são fundamentais para o seguimento dos pacientes transplantados. O principal método diagnóstico e padrão-ouro permanece sendo a biópsia endomiocárdica (BEM). No entanto, por ser um procedimento invasivo, não é isento de riscos, e as alterações inflamatórias podem não ser contínuas no miocárdico, dificultando o diagnóstico na amostra restrita abordada pela BEM.^835 , 836^ Dessa forma, exames diagnósticos não invasivos continuam em evolução e na tentativa de detecção cada vez mais precoce da rejeição do enxerto.^822^

A RMC identifica o comprometimento da função ventricular e permite uma melhor avaliação do VD, cuja disfunção pode ocorrer no pós-operatório do transplante cardíaco. Apesar da disfunção ventricular estar presente em casos de rejeição do enxerto, ela costuma ser identificada apenas em estágios mais avançados, em que provavelmente já exista algum grau de lesão miocárdica.

A rejeição celular aguda pode ser detectada pela RMC nas sequências para caracterização tecidual, que podem mostrar inflamação/edema e necrose/fibrose miocárdica. Edema miocárdico é analisado nas sequências anatômicas ponderadas em T2 ou pode ser avaliado diretamente de forma quantitativa pelo Mapa T2. Taylor et al. mostraram que o uso de um critério combinado incluindo edema miocárdico avaliado por imagens ponderadas em T2 (sinal relativo > 2) ou presença de realce precoce (após administração de gadolínio) apresentou sensibilidade e especificidade de 100% e 73% respectivamente, comparado à BEM, além de poder identificar rejeição significativa (≥ 2R) do enxerto.^837^ Através da análise do Mapa T2, estudos retrospectivos descreveram um alto valor preditivo negativo (97%) para detecção de rejeição aguda ≥ 2R utilizando valor de T2 ≥ 56 ms e um risco relativo maior que 2 para rejeição com T2 > 60 ms. A associação de T2 > 59 ms com o volume diastólico final indexado do VD mostrou VPN de 98% para rejeição significativa ≥ 2R.^821 , 823 , 833 , 838^ Ide et al. mostraram, em um estudo prospectivo com população pediátrica, que o uso de Mapa T1 (T1 nativo e VEC) se correlacionou com volume de colágeno da amostra histológica, mostrando o remodelamento fibrótico do miocárdio acelerado em crianças.^824 , 825 , 839^

Nos últimos anos, tem se mostrado a transição das análises por RMC qualitativas para quantitativas, usando os dados acima detalhados em conjunto. Isso pode ser observado em publicações recentes,^825 – 827^ mostrando o uso combinado de T2 e VEC como potenciais biomarcadores para detectar a rejeição celular aguda.

A Tabela 48 descreve as principais indicações da RMC para avaliação das alterações cardíacas associadas ao transplante cardíaco.

**Tabela 48:** Ressonância magnética cardíaca na avaliação de alterações cardíacas associadas ao transplante cardíaco

Indicação	Classe de recomendação	Nível de evidência
Piora da função ventricular sem evidência de doença vascular do enxerto^840^	IIa	B
Piora da função ventricular e biópsia endomiocárdica negativa/inconclusiva^840^	IIa	B

### 3.22. Doenças do Pericárdio

As doenças pericárdicas são relativamente comuns em todo o mundo, podem se apresentar como um processo isolado ou associado a diferentes condições sistêmicas.^340 , 343 , 841^ Tradicionalmente, o ecocardiograma tem sido o método de escolha e, em grande parte, o único necessário para a avaliação das doenças pericárdicas.^343 , 841 , 842^ Entretanto, a TC e a RMC estão sendo cada vez mais usadas como parte de uma abordagem racional de imagem multimodalidade.^340 , 342^

#### 3.22.1. Papel da RMC

A RMC é indicada principalmente nos casos em que o ecocardiograma apresenta janela limitada ou persiste com dúvida diagnóstica, permitindo uma avaliação mais precisa do pericárdio.^342^ A RMC pode ainda ser indicada para o acompanhamento evolutivo de pacientes com doença pericárdica mais complexa e imagem ecocardiográfica limitada, tendo em vista a não utilização de radiação ionizante e contraste iodado.^341^

A investigação da doença pericárdica com RMC inclui sequências específicas de imagens morfológicas, para avaliar a estrutura pericárdica e realizar a caracterização tecidual, e imagens funcionais, para medir a função ventricular e os fluxos intracardíacos, possibilitando, inclusive, a avaliação de eventual repercussão hemodinâmica das doenças pericárdicas.^340 , 341 , 843^

#### 3.22.2. Derrame Pericárdico

O derrame pericárdico é uma entidade clínica comum, na qual há acúmulo de líquido maior que 50 mL no saco pericárdico decorrente de distúrbio que afeta seletivamente o pericárdio ou como resposta a doença sistêmica. O líquido do saco pericárdico pode ser transudato, exsudato, hemorragia (hemopericárdio), pus (piopericárdio) ou linfa (quilopericárdio).^340 , 844^

O papel da imagem é confirmar a presença de derrame pericárdico, estimar a quantidade, descrever sua distribuição e determinar qualquer efeito hemodinâmico no coração.^844^ A RMC define a distribuição e a quantidade de líquido pericárdico com mais precisão do que o ecocardiograma.^342^ O emprego de sequências de imagem direcionadas ajuda na caracterização do conteúdo do saco pericárdico – gordura, líquido com mais ou menos conteúdo proteico –e na conduta a ser adotada.^340^ Derrames transudativos tipicamente se manifestam com baixa intensidade de sinal em T1 e alto sinal em T2 na RMC. Derrames exsudativos parecem heterogêneos com uma intensidade de sinal intermediária em imagens de RMC ponderadas em T1 (com tendência a alto sinal) e T2. Um derrame hemorrágico seguirá a intensidade do sinal dos produtos sanguíneos na RM.^344^

#### 3.22.3. Pericardite Aguda

A RMC deve ser considerada na presença de achados ecocardiográficos inconclusivos, ausência de resposta à terapêutica instituída e apresentação clínica atípica.

O método é bem indicado, nesse contexto, na suspeita de pericardite constritiva – permitindo avaliação inclusive da repercussão hemodinâmica, e no pós-IAM – quando persistem dúvidas quanto à possibilidade de hemopericárdio contido secundário à ruptura da parede livre.^344^

#### 3.22.4. Tamponamento Pericárdico

A RMC não apresenta indicação no tamponamento cardíaco, entidade acompanhada de instabilidade hemodinâmica, com diagnóstico eminentemente clínico e facilmente avaliada pelo ecocardiograma. Entretanto, nos casos em que a presença de tamponamento cardíaco ainda é incerta, a RMC fornece um complemento útil para avaliar tamponamento localizado ou loculado. Os critérios na RMC para um derrame funcionalmente importante são semelhantes aos do ecocardiograma: compressão diastólica da parede livre do VD, colapso sistólico precoce do átrio direito (AD), distorção da morfologia do VE e VD e, potencialmente, deslocamento do septo interventricular para o lado esquerdo durante a inspiração inicial (interdependência ventricular), embora este último seja mais comumente visto na pericardite constritiva.^341^

#### 3.22.5. Pericardite Constritiva

A pericardite constritiva é uma condição na qual um pericárdio não complacente, espessado, inelástico e frequentemente calcificado limita o enchimento diastólico dos ventrículos ao impedir a transmissão total das alterações da pressão intratorácica respiratória para as cavidades cardíacas.^341^

Em pacientes com suspeita de constrição, os exames de imagem podem se concentrar nas informações diagnósticas, incluindo: espessura pericárdica, dependência interventricular, outras anormalidades associadas (valvular, miocárdica ou DAC) e evidências de patologias alternativas, como cardiomiopatia restritiva, disfunção do VD ou regurgitação tricúspide grave. O ecocardiograma é o exame de imagem inicial e pode fornecer um diagnóstico definitivo de pericardite constritiva para a maioria dos pacientes, embora essa seja uma boa indicação para avaliação pela RMC.^342^

A RMC permite a detecção de características hemodinâmicas de constrição, semelhantes às observadas no ecocardiograma, principalmente através do uso das imagens de cinerressonância em tempo real.^845^ Um pericárdio espessado na RMC (> 4 mm), no cenário clínico adequado, frequentemente apoia o diagnóstico de pericardite constritiva, embora a ausência de espessamento pericárdico não necessariamente a exclua.^341^

Aderências pericárdicas com mobilidade reduzida do miocárdio podem ser detectadas por imagens marcadas de cinerressonância ( *tagging* ).^342^ A RMC é valiosa, ainda, para avaliar a extensão da inflamação pericárdica, pela detecção de edema e/ou RT pericárdicos nesses pacientes, entretanto não são achados universais.^342^

#### 3.22.6. Massas Pericárdicas

Os tumores pericárdicos são muito raros e podem ser divididos em primários (benignos e malignos) e metastáticos.

As neoplasias benignas primárias do pericárdio são tumores de crescimento lento, normalmente de bordas bem definidas e frequentemente detectados incidentalmente,^340^ mas podem causar complicações cardiovasculares significativas devido ao efeito de massa com repercussão hemodinâmica.^343^ Teratoma, lipoma (o tumor benigno do pericárdio mais comum), fibroma, hemangioma, linfangioma, paraganglioma e mioblastoma de células granulares são os mais encontrados.^340 – 342^

Neoplasias pericárdicas malignas primárias também são bastante raras e incluem mesotelioma (o mais comum), sarcoma (segundo mais comum), linfoma, lipossarcoma, teratoma maligno e hemangioendotelioma.^340^ A maioria dos casos de tumores malignos primários não é ressecável no momento do diagnóstico. Quimioterapia e radioterapia oferecem poucos benefícios.^342^

As metástases pericárdicas são mais frequentes do que a neoplasia pericárdica primária e geralmente ocorrem tardiamente no processo da doença. A maioria dos tumores pericárdicos são lesões secundárias devido à disseminação local do pulmão e tumores do mediastino ou devido a lesões metastáticas de cânceres de pulmão e mama, linfomas e melanoma.^841^

A RMC permite avaliar melhor a dimensão da massa pericárdica, sua relação com as estruturas adjacentes e eventuais repercussões hemodinâmicas.^342^ Embora a RMC seja útil para a diferenciação entre tumor pericárdico benigno e maligno,^343^ a biópsia e a análise histopatológica, entretanto, continuam necessárias para alcançar um diagnóstico definitivo para a maioria dos tumores pericárdicos.^340^

Lesões pericárdicas bem definidas ou encapsuladas sem irregularidade pericárdica ou derrames têm maior probabilidade de serem benignas.^342^ Aderência pericárdica e/ou extensão direta para estruturas adjacentes são geralmente indicações de malignidade, e a marcação dinâmica ( *tagging* ) pode ajudar a identificar tais alterações. Na RMC, as metástases pericárdicas podem se apresentar como espessamento pericárdico irregular ou nodular, nódulos ou massas,^340^ podendo se apresentar como múltiplas massas pericárdicas com realce e coalescência, geralmente com um grande derrame exsudativo associado.^841^

A maioria das neoplasias tem baixo sinal nas imagens ponderadas em T1 e alto sinal nas imagens ponderadas em T2. Na perfusão de primeira passagem, os tumores pericárdicos primários e secundários – predominantemente os malignos – mostram algum grau de realce com meios de contraste a base de gadolínio. Por causa de sua vascularização aumentada, as neoplasias pericárdicas apresentam RT variável e heterogêneo após a administração de contraste, observando-se hiperintensidade mais significativa, principalmente nos tumores malignos altamente vascularizados.^340 , 342^

#### 3.22.7. Cistos e Divertículos Pericárdicos

Os cistos pericárdicos estão habitualmente localizados adjacentes à borda cardíaca, na maioria das vezes no ângulo cardiofrênico direito.^841 , 844^ O divertículo é diferenciado do cisto por apresentar comunicação com o espaço pericárdico.^343^

Os cistos e divertículos pericárdicos quase sempre aparecem com sinal de baixa e homogênea intensidade em imagens ponderadas em T1 e com intensidades altas em imagens T2 e não mostram realce após administração de contraste. Muito raramente, podem conter líquido altamente proteico, levando a alta intensidade de sinal nas imagens T1.^342 , 841^

#### 3.22.8. Abscesso Pericárdico

O abscesso pericárdico é uma coleção isolada de pus dentro do espaço pericárdico e frequentemente se apresenta como uma coleção biconvexa localizada dentro do espaço pericárdico comprimindo as câmaras cardíacas adjacentes. Na RMC, o núcleo pode ser mais bem demonstrado, parecendo isointenso a hiperintenso nas imagens ponderadas em T1 e T2.^841^

#### 3.22.9. Hematomas

A RMC é particularmente útil para o diagnóstico de hematomas pericárdicos, que têm uma intensidade de sinal característica nas imagens ponderadas em T1 e T2: hematomas agudos demonstram alta intensidade de sinal homogêneo, enquanto hematomas subagudos (1 a 4 semanas) tipicamente mostram intensidade de sinal heterogênea, com áreas de alta intensidade de sinal nas imagens ponderadas em T1 e T2. Em imagens ponderadas em T1, hematomas crônicos organizados podem mostrar uma borda periférica escura e focos internos de baixa intensidade de sinal, que podem representar calcificação, fibrose ou deposição de hemossiderina. Os hematomas não aumentam a intensidade de sinal após o contraste.^845^

#### 3.22.10. Ausência Congênita do Pericárdio

É uma doença rara, causada por um desenvolvimento embriológico anormal, que pode se manifestar isoladamente ou associada a outras desordens congênitas como valva aórtica bicúspide, persistência do canal arterial, estenose mitral e defeito do septo atrial ou tetralogia de Fallot.^845^

Na RMC, o pericárdio pode ser identificado porque é diferenciado do miocárdio adjacente pela presença das camadas de gordura epicárdica e pericárdica, que apresentam intensidade de sinal diferente do pericárdio. Em pacientes com pouco conteúdo de gordura epicárdica (jovens, magros, atletas), essa identificação pode ser difícil, especialmente porque a região habitualmente com menor conteúdo de gordura epicárdica é a mesma da localização mais comum do defeito pericárdico (paredes lateral, posterior e inferior do VE), devendo-se atentar para evitar resultados de imagem falso-positivos.^342^ Existem importantes sinais morfológicos e funcionais indiretos consistentes com defeitos pericárdicos: a interposição do parênquima pulmonar entre a aorta e a artéria pulmonar, o deslocamento significativo do coração para a esquerda e o movimento excessivo do ápice cardíaco.^344 , 841^

A Tabela 49 apresenta as principais indicações da RMC na avaliação do pericárdio.

**Tabela 49:** Indicações da RMC na avaliação do pericárdio

Indicação	Classe de recomendação	Nível de evidência
Avaliação da repercussão hemodinâmica de derrame pericárdico volumoso, com imagens inconclusivas ao ecocardiograma^341 , 342 , 845^	IIa	B
Pericardite aguda (< 3 meses)^341 , 342 , 845^	IIa	B
Pericardite crônica (> 3 meses)^341 , 342 , 845^	IIa	B
Pericardite constritiva sem suspeita de calcificação pericárdica associada^341 , 342 , 845^	IIa	B
Pericardite constritiva com suspeita de calcificação pericárdica associada^341^	IIa	C
Pesquisa de anomalias congênitas do pericárdio^340 , 342 , 344 , 841^	I	B

### 3.23. Massas Cardíacas e Trombo

As massas cardíacas são raras e possuem um amplo diagnóstico diferencial, incluindo tumores benignos, tumores malignos (primários e secundários) e condições semelhantes a tumores, como trombos, cistos pericárdicos e vegetações.^846^ A ecocardiografia transtorácica é a técnica de escolha na avaliação inicial devido, principalmente, à sua disponibilidade, ao baixo custo e à portabilidade; entretanto, o método possui limitações, como limitação na avaliação de câmaras cardíacas direitas, estruturas mediastinais e extracardíacas.^847^

A RMC constitui-se como um dos principais métodos de avaliação dessas estruturas, uma vez que fornece dados relacionados a localização, tamanho, bordas, caracterização tecidual, vascularização, relação com estruturas vizinhas, além da função cardíaca.^848^ As diferentes técnicas de aquisição de imagens possibilitam a identificação de características importantes quanto aos componentes histológicos (presença de gordura, conteúdo hemorrágico, necrótico, melanina, cálcico, mixóide e líquido), além da vascularização tecidual, que, associado a informações como a localização da massa ( Tabela 50 ), pode auxiliar na distinção entre massas não neoplásicas (trombos, cistos e vegetações) e massas neoplásicas benignas ou malignas.^849^ A despeito de sua característica celular, qualquer tumor cardíaco, mesmo que histologicamente benigno, pode ter consequências hemodinâmicas ou arrítmicas substanciais, dependendo de seu tamanho e localização.^850 , 851^

**Tabela 50:** Características dos tumores cardíacos pela ressonância magnética

MASSA CARDÍACA	LOCALIZAÇÃO	CINE-RM	IMAGEM PONDERADA EM T1	IMAGEM PONDERADA EM T2	REALCE TARDIO
LESÕES NÃO NEOPLÁSICAS					
TROMBO	Átrio esquerdo (fibrilação atrial) Ventrículo esquerdo (aneurisma) Átrio direito (cateter venoso central)	Hipo ou isointenso	Agudo: Hiperintenso Crônico: Hipointenso	Hipointenso ou hiperintenso	Sem captação
CISTO	Pericárdico (ângulo cardiofrênico direito)	Hiperintenso	Hipointenso	Hiperintenso	Sem captação
**TUMORES BENIGNOS**					
MIXOMA	Fossa oval do septo atrial (átrio esquerdo 80%, átrio direito 20%)	Lesão móvel	Isointenso, heterogêneo	Hiperintenso	Realce heterogêneo
FIBROELASTOMA PAPILAR	Válvula cárdica (geralmente a esquerda)	Móvel, hiperintensa, fluxo turbulento peritumoral	Isointenso	Isointenso	Hiperrealce
LIPOMA	Epicárdico (70%), ventrículos, septo interatrial	Borda escura, hiperintensa	Hiperintenso	Hiperintenso	Sem captação
FIBROMA	Septo interventricular, parede do ventrículo esquerdo, ventrículo direito	Hipo ou isointenso	Iso/hiperintenso	Hipointenso	Hiperrealce homogêneo
RABDOMIOMA	Ventrículo e septo interventricular	Massa intramural, levemente hiperintensa	Iso/hiperintenso	Iso/hiperintenso	Sem captação ou captação mínima
HEMANGIOMA	Ventrículo e septo interventricular	Hiperintenso	Isointenso	Hiperintenso	Realce prolongado heterogêneo
**TUMORES MALIGNOS**					
MESOTELIOMA	Pericárdico (exposição a amianto ou alterações pleurais)	Nódulo hipointenso	Isointenso	Intenso heterogêneo	Realce heterogêneo
ANGIOSSARCOMA	Átrio direito	Isointenso e heterogêneo	Hiperintenso heterogêneo	Hiperintenso heterogêneo	Realce heterogêneo
RABDOMIOSSARCOMA	Qualquer câmara	Isointenso	Isointenso	Isointenso	Realce heterogêneo
LEIOMIOSSARCOMA	Átrio esquerdo - parede posterior	Hipo ou isointenso	Isointenso	Hiperintenso	Inespecífico
SARCOMA SINOVIAL	Pericárdio - átrio direito	Hipo ou isointenso	Isointenso	Ligeiramente hiperintenso	Realce heterogêneo
LINFOMA	Átrio direito	Isointenso	Hipo/isointenso	Ligeiramente hiperintenso	Sem captação ou captação mínima
METÁSTASE	Depende do sítio envolvido Pericárdio	Depende do sítio envolvido	Hipointenso	Hiperintenso	Realce heterogêneo

Cine-RM: cinerresonância magnética; LGE: realce tardio com gadolínio.

As massas cardíacas mais frequentes são as não neoplásicas, que podem mimetizar tumores cardíacos (por exemplo, trombos, vegetações valvares, abscessos perivalvares, cistos pericárdicos, calcificação anular mitral etc.). Entre as massas não neoplásicas, os trombos intracavitários são os mais comuns, geralmente ocorrendo no AE, relacionados à fibrilação atrial ou doença valvar, podendo ser também encontrados no VE com FE reduzida.^852^ A imagem de perfusão de primeira passagem permite clara diferenciação do trombo em relação ao miocárdio adjacente, uma vez que o trombo é uma estrutura avascular. A utilização de contraste (gadolínio) aumenta a acurácia do método para identificação dos trombos. Não há captação em meio de contraste, tanto no realce precoce quanto no RT. O realce periférico pode ser observado ocasionalmente em trombos crônicos devido a componentes fibróticos.^570 , 853^

Os tumores cardíacos primários são raros (taxa de incidência inferior a 0,3%), e mais de 75% deles são benignos.^854 , 855^ O mixoma cardíaco é o tumor benigno mais frequente em adultos, representando cerca de 50% do total de casos. Ocorre mais frequentemente no sexo feminino, geralmente entre a 3ª e a 6ª décadas de vida. São mais comumente encontrados no AE como nódulos solitários, móveis, aderidos à fossa oval.^856^ Se manifestam tipicamente como isointensos ou heterogêneos em imagens ponderadas em T1 e hiperintensos ou heterogêneos em imagens ponderadas em T2, pelo alto teor de água extracelular. Cerca de metade dos mixomas cardíacos apresentam realce heterogêneo.^850^

Os lipomas constituem o segundo tipo de tumor cardíaco mais frequente (cerca de 16%), usualmente descobertos incidentalmente por serem assintomáticos. São bem definidos, homogêneos, com morfologia capsulada e, em geral, localizados no VE e AD, caracterizados à RMC por hipersinal em T1 pela presença de tecido adiposo.^857^

O fibroelastoma papilar representa cerca de 75% de todas as neoplasias valvares e 10% dos tumores cardíacos primários. Geralmente são solitários e pequenos (menores de 2 cm), ocorrem em pessoas de 60 a 80 anos, sem diferença entre os sexos e têm como principais sintomas os eventos embólicos. Podem surgir de qualquer superfície endocárdica, mas as localizações mais comuns são a face atrial da valva mitral e a superfície aórtica dos folhetos da valva aórtica.^858^ Apresentam-se hipointensos em imagens de cine e isointensos nas imagens ponderadas em T1 e T2.

O rabdomioma é o tumor primário cardíaco mais comum na infância, representando cerca de 90% dos tumores cardíacos benignos primários nessa faixa etária, sendo que 75% ocorrem em crianças menores de 1 ano. A maioria das lesões tende a regredir espontaneamente e é caracterizada à RMC por sinal intermediário a alto nas imagens ponderadas em T1 e sinal intermediário em T2, sem RT. Outros tumores cardíacos primários são fibromas, paraganglioma intrapericárdico, hemangiomas e teratomas.^859 , 860^

Dos tumores malignos primários, os sarcomas são os mais comuns, e, dos tumores secundários, as neoplasias extracardíacas mais frequentemente associadas são de pulmão, linfoma, mama e câncer de esôfago. As metástases são 20 a 40 vezes mais comuns do que tumores cardíacos primários, podendo ser resultados de invasão direta (carcinomas broncogênicos e linfoma), disseminação hematogênica (neoplasias pulmonares e de mama, melanomas, linfomas e leucemia), linfática (via mais comum dos linfomas e mais raro nos carcinomas) ou por contiguidade com estruturas vasculares venosas (carcinomas hepatocelulares, renais, endometriais e tireoidianos).^861^

O angiossarcoma é o tipo histológico mais comum na idade adulta dos tumores malignos primários cardíacos. É um sarcoma altamente agressivo que muitas vezes se origina no AD e tem alta probabilidade de metástases no momento da apresentação. Nas imagens ponderadas em T1, aparece como lesões isointensas com múltiplas áreas nodulares de alta intensidade.^357^ As características de RT com gadolínio do tumor mostram realce heterogêneo e podem mostrar um grande núcleo necrótico sem realce,^862^ além de evidências de vascularização na perfusão de primeira passagem.

O rabdomiossarcoma é o tumor primário cardíaco maligno mais comum na infância. São frequentemente volumosos (> 10 cm de diâmetro) e invasivos, sem predileção por câmara cardíaca, sendo mais comuns nas valvas. Se caracterizam na RMC por ter sinal homogêneo, geralmente isointenso ou discretamente hiperintenso nas imagens ponderadas em T1 e T2, com a necrose central do tumor sendo uma característica distintiva^863^ e RT acentuado.

O mesotelioma é raro e surge do pericárdio na maioria dos casos. É comum em homens idosos com história de exposição a amianto e alterações pleurais. Geralmente se manifesta por crescimento difuso, massas múltiplas e mal definidas dentro da cavidade pericárdica, dando origem a extenso espessamento pericárdico e volumoso derrame pericárdico hemorrágico, podendo apresentar sinais e sintomas de pericardite constritiva ou tamponamento cardíaco.^864^ A RMC mostra massa pericárdica, que é circundada pelos pericárdios visceral e parietal, apresentando-se isointensa em sequências ponderadas em T1 e sinal heterogêneo em T2.

Os linfomas cardíacos primários são extremamente raros, típicos do linfoma não Hodgkin e restritos ao coração e ao pericárdio. Geralmente ocorre em pacientes imunocomprometidos associado à infecção pelo vírus Epstein-Barr. Na RMC, são hipo/isointensos nas imagens ponderadas em T1 e levemente hiperintensos nas imagens ponderadas em T2. Associa-se, ainda, impregnação heterogênea pelo gadolínio, podendo encontrar áreas hipointensas centrais.^849^

A RMC demonstra características úteis na diferenciação entre tumores maligno e benigno. O tamanho do tumor, a infiltração local, a presença de vascularização aumentada, o envolvimento de mais de uma câmara cardíaca e o derrame pericárdico são bons indicadores para avaliar o grau de malignidade. Além de auxiliar na caracterização do tipo histológico, a RMC proporciona um melhor detalhamento do tumor e sua relação com as estruturas extracardíacas, fundamental para a avaliação pré-operatória.^865^

A Tabela 50 traz as características de diferentes tumores cardíacos identificadas pela RM.

A Tabela 51 apresenta as principais indicações e cenários clínicos da RMC na avaliação de massas/trombos cardíacos.

**Tabela 51:** Avaliação de massas/trombo cardíaco pela ressonância magnética

Indicação	Classe de recomendação	Nível de evidência
Detecção e caracterização de tumores cardíacos e pericardíacos^419 , 850 , 866 – 868^	I	B
Detecção e diagnóstico diferencial de trombos ventriculares^419 , 866 , 867 , 869^	I	B
Detecção de trombos atriais e em apêndice atrial^870^	IIa	B
Seguimento de tumores cardíacos benignos^419 , 867^	I	C
Seguimento tumoral (recorrência pós-ressecção, pós-quimio/radioterapia)^871 – 873^	I	C

### 3.24. Doenças Valvares

O exame de primeira linha na avaliação dos pacientes com doença orovalvar é o ecocardiograma transtorácico.^251 , 874 , 875^ Entretanto, a RMC possui vantagens importantes em relação ao ecocardiograma, como: (1) liberdade na escolha dos planos de imagem, sem necessidade de se limitar a cortes específicos determinados por janelas acústicas,^876^ (2) superioridade na quantificação das lesões valvares regurgitantes,^875 , 877 – 880^ (3) maior acurácia e reprodutibilidade na avaliação morfológica e funcional de ambos os ventrículos, incluindo quantificação dos volumes, FE e massa ventriculares^881 , 882^ e (4) capacidade de proporcionar caracterização tecidual do miocárdio.^418 , 883 , 884^ Consequentemente, uma proporção crescente dos pacientes com doença orovalvar tem se beneficiado da versatilidade da RMC na complementação da avaliação ecocardiográfica.^875 , 885 , 886^

Primeiramente, a avaliação por RMC deve incluir a caracterização da morfologia e estrutura da valva, assim como a análise do jato de estenose e/ou regurgitação e, quando possível, a determinação do mecanismo causador da lesão valvar. No caso da doença valvar aórtica, por exemplo, é importante avaliar se existe espessamento, calcificação, retração ou fusão dos folhetos, determinar o número de folhetos, caracterizar o jato estenótico ou regurgitante (tamanho, direção, excêntrico vs. central, único vs. fragmentado), pesquisar a presença de dilatação da raiz aórtica ou aorta ascendente etc. No caso de pacientes com regurgitação mitral, por exemplo, é importante avaliar o espessamento dos folhetos, a calcificação no anel mitral e aparelho subvalvar, a mobilidade (prolapso, " *flail* ", " *tethering* ") ou perfuração dos folhetos e a localização da anormalidade valvar e, em última análise, determinar o mecanismo da regurgitação e ser capaz de definir a insuficiência mitral como primária, secundária (funcional) ou mista.^875^ A mesma abordagem deve ser realizada nos casos de avaliação de lesões das valvas tricúspide e pulmonar.

O segundo passo é a avaliação da gravidade da lesão valvar. Os principais métodos da RMC para a graduação de tais lesões são:

- Lesões estenóticas:
  1. Estenose aórtica. Os dois principais parâmetros medidos pela RMC para quantificar o grau de estenose aórtica são (1) velocidade máxima do jato de estenose aórtica (Vmax) medida pela técnica de contraste de fase (" *phase-contrast* ") e (2) medida direta da área valvar aórtica por planimetria. Em ambos os casos (tanto na medida da Vmax como da área valvar aórtica), é fundamental que o plano de corte utilizado para a medida seja posicionado perpendicularmente ao jato de estenose aórtica.
  2. Estenose pulmonar. Os mesmos métodos descritos para a avaliação da estenose aórtica são utilizados para a avaliação da estenose pulmonar, isto é, a quantificação da velocidade máxima do jato de estenose pulmonar (Vmax) e medida direta da área valvar pulmonar por planimetria.
  3. Estenose mitral e estenose tricúspide. De uma forma geral, essas lesões valvares são avaliadas de forma adequada pelo ecocardiograma. Entretanto, nos casos em que a avaliação ecocardiográfica for limitada, a RMC permite a avaliação da gravidade da estenose mitral ou tricúspide utilizando os princípios descritos acima para as valvas aórtica e pulmonar: medida da Vmax por " *phase-contrast* " e área valvar por planimetria direta.
- Lesões regurgitantes:^878 , 879 , 887 – 889^
  1. Insuficiência aórtica. Os três principais métodos de RMC para quantificar o grau de insuficiência aórtica são: (1) medida do volume regurgitante (fluxo retrógrado aórtico na diástole) em um corte posicionado perpendicular à aorta ascendente entre o plano valvar aórtico e a junção sinotubular utilizando a técnica de " *phase-contrast* ". A fração regurgitante pode ser calculada como o volume regurgitante dividido pelo volume anterógrado total aórtico medido na sístole.^878^ (2) Na ausência de lesões concomitantes em outras valvas (lesões triviais ou leves podem ser desconsideradas), o volume regurgitante aórtico pode ser medido como a diferença entre o volume sistólico do VE e o volume sistólico do VD obtida pela técnica de Simpson nas imagens de cinerressonância magnética (cine-RM). Nesse caso, a fração regurgitante pode ser calculada como o volume regurgitante dividido pelo volume sistólico do VE. (3) Medida direta da área do orifício regurgitante aórtico por planimetria.
  2. Insuficiência mitral. Os três principais métodos de RMC para quantificar o grau de insuficiência mitral são: (1) quantificação do volume regurgitante medido como a diferença entre o volume sistólico do VE obtida pela técnica de Simpson nas imagens de cine-RM e o volume anterógrado na aorta ascendente medido na sístole pela técnica de " *phase-contrast* ". A fração regurgitante pode ser calculada como o volume regurgitante dividido pelo volume sistólico total do VE.^880 , 886^ (2) Na ausência de lesões concomitantes em outras valvas (lesões triviais ou leves podem ser desconsideradas), o volume regurgitante mitral pode ser medido como a diferença entre o volume sistólico do VE e o volume sistólico do VD obtida pela técnica de Simpson nas imagens de cine-RM. Nesse caso, a fração regurgitante pode ser calculada como o volume regurgitante dividido pelo volume sistólico do VE. (3) Medida direta da área do orifício regurgitante mitral por planimetria.
  3. Insuficiência pulmonar. A avaliação da insuficiência pulmonar pela RMC segue a mesma estratégia utilizada na avaliação da insuficiência aórtica: (1) medida do volume/fração regurgitante comparando o volume sistólico anterógrado e o volume retrógrado diastólico no tronco da artéria pulmonar utilizando a técnica de " *phase-contrast* ". (2) Na ausência de lesões concomitantes em outras valvas (lesões triviais ou leves podem ser desconsideradas), o volume/fração regurgitante pulmonar pode ser medido como a diferença entre o volume sistólico do VD e o volume sistólico do VE obtida pela técnica de Simpson nas imagens de cine-RM. (3) Medida direta da área do orifício regurgitante pulmonar por planimetria.
  4. Insuficiência tricúspide. A avaliação da insuficiência tricúspide pela RMC segue a mesma estratégia utilizada na avaliação da insuficiência mitral: (1) medida do volume/fração regurgitante comparando o volume sistólico do VD obtido pela técnica de Simpson nas imagens de cine-RM e o volume anterógrado no tronco da artéria pulmonar medido na sístole pela técnica de " *phase-contrast* ". (2) Na ausência de lesões concomitantes em outras valvas (lesões triviais ou leves podem ser desconsideradas), o volume/fração regurgitante tricúspide pode ser medido como a diferença entre o volume sistólico do VD e o volume sistólico do VE obtida pela técnica de Simpson nas imagens de cine-RM. (3) Medida direta da área do orifício regurgitante tricúspide por planimetria.

Com relação aos valores de corte utilizados para classificar as lesões valvares como leves, moderadas ou graves, já existem dados sugerindo valores de corte específicos para RMC.^876^ Entretanto, ainda existem poucos estudos validando dados específicos para RMC, e, portanto, os valores de corte oriundos da ecocardiografia ainda são os mais frequentemente utilizados.^874^

O terceiro passo consiste em avaliar a morfologia e a função de ambos os ventrículos e átrios. Como mencionado anteriormente, a RMC é mais acurada e reprodutível que o ecocardiograma nesse tipo de avaliação,^880 , 881^ o que a torna especialmente útil no acompanhamento longitudinal dos pacientes com doença orovalvar. A quantificação dos volumes cavitários e da função biventricular permite avaliar a resposta adaptativa do coração à sobrecarga pressórica e/ou volumétrica causada pelas lesões valvares. Essa informação é importante não apenas porque representa dado adicional que ajuda na avaliação da gravidade das lesões valvares,^875^ mas também porque constitui parâmetros definidores da estratégia terapêutica, incluindo o melhor momento para indicar a intervenção cirúrgica ou percutânea.^251 , 874 , 885 , 889^

Uma contribuição única da RMC na avaliação dos pacientes com doença orovalvar consiste na sua capacidade de proporcionar caracterização tecidual do miocárdio.^418 , 883 , 884^ Diversos estudos demonstraram que a avaliação do VE quanto a presença, extensão e padrão de fibrose miocárdica utilizando a técnica do RT proporciona informações prognósticas importantes^890 , 891^ que podem auxiliar no manejo dos pacientes com doença valvar aórtica e/ou mitral.^892 – 897^ Mais recentemente, alguns estudos demonstraram que técnicas de mapeamento T1^883 , 898^ também podem proporcionar informações prognósticas valiosas e complementares à avaliação pela técnica do RT.^876 , 895^

Finalmente, cabe ressaltar que a RMC permite avaliar pacientes portadores de próteses valvares,^876^ sejam biológicas ou mecânicas. A presença e extensão dos artefatos de susceptibilidade dependerão da quantidade de material ferromagnético na prótese. Em alguns casos, é possível avaliar a estrutura da prótese e a mobilidade dos folhetos (artefatos de susceptibilidade ausentes ou discretos); já em outros, essa avaliação não é possível (artefatos de susceptibilidade extensos). Não obstante, na grande maioria dos casos é possível avaliar quantitativamente lesões estenóticas (medindo a Vmax do jato estenótico em um plano de corte imediatamente distal à região de artefato) e lesões regurgitantes (utilizando as mesmas técnicas descritas acima para a avaliação quantitativa das valvas nativas).

A Tabela 52 traz os principais cenários clínicos relacionados à utilização da RMC na avaliação das doenças valvares.

**Tabela 52:** Emprego da ressonância magnética cardíaca na avaliação de doenças valvares

Indicação	Classe de recomendação	Nível de evidência
Avaliação da anatomia e da função ventricular^251 , 419^	I	A
Avaliação quantitativa complementar ao ecocardiograma de lesões regurgitantes^212 , 251 , 874 , 886^	IIa	B
Quantificação da gravidade da insuficiência valvar mitral primária em pacientes com planejamento de troca ou reparo valvar (cirúrgico ou transcateter)^212 , 251 , 874 , 880 , 886^	IIa	B
Avaliação complementar ao ecocardiograma de lesões estenóticas^251 , 419 , 874 , 899 , 900^	IIa	B
Pesquisa de fibrose miocárdica na avaliação prognóstica de estenose valvar aórtica^212 , 251 , 874 , 892 , 901^	IIa	B
Avaliação complementar ao ecocardiograma de próteses valvares^902 , 903^	IIb	C
Avaliação de massas ou vegetações suspeitas em folhetos valvares, para o diagnóstico diferencial de endocardite infecciosa^904^	III	C

### 3.25. Cardio-oncologia

A melhora da sobrevida associada com a eficácia do tratamento para o câncer tem propiciado o aumento da incidência de complicações cardíacas relacionadas ao tratamento antineoplásico. A detecção precoce da cardiotoxicidade, além de identificar precocemente lesão ao sistema cardiovascular, pode propiciar a implementação de terapias dirigidas.^905^

Apesar de diversos avanços, a detecção efetiva da cardiotoxicidade encontra vários desafios. Não infrequentemente, a disfunção cardíaca ocorre apenas muito tempo após o termino do tratamento oncológico, dificultando a associação com a terapia contra o câncer.^906^ Além disso, essa avaliação baseia-se exclusivamente no seguimento longitudinal da função sistólica do VE e da FEVE obtidas pelo ecocardiograma convencional, que apresenta diversas limitações, como reprodutividade limitada e incapacidade de avaliar a caracterização tecidual.^907^ Finalmente, reduções da FEVE ocorrem frequentemente dentro dos valores da normalidade, indicando que a lesão do músculo cardíaco pode estar presente mesmo antes da presença da disfunção do VE.^908 , 909^ Dentro desse contexto, destaca-se a capacidade da RMC em obter dados considerados "padrão-ouro" para morfologia e função cardíaca, com resultados reprodutíveis e acurados.^910^ A RMC também oferece diferentes tipos de modalidades de imagem, como sequências em cine para morfologia/função, T2 para edema, perfusão para isquemia, RT para cicatriz, *tagging* para avaliação de *strain* e mapas de T1 para caracterização tecidual.

#### 3.25.1. Avaliação da Morfologia e Função Ventricular

A RMC é considerada o método de escolha para avaliação das alterações da morfologia e função causadas pela cardiotoxicidade,^911 – 913^ fornecendo precisamente informações sobre os volumes e massa de maneira reprodutível e independente.^914^ Drafts et al.^915^ comprovaram que as imagens em cine da RMC são capazes de detectar alterações precoces da função cardíaca, mesmo quando pacientes são expostos a doses baixas e intermediárias de antracíclicos. Um outro estudo comparando diferentes técnicas de imagem em adultos que apresentaram câncer na infância tratados com antraciclinas demonstrou que o ecocardiograma convencional apresenta acurácia limitada para confirmar a presença de FEVE < 50% definida pela RMC, com sensibilidade de 25% e altas taxas de falsos-positivos (75%).^916^ Apesar da ecocardiografia 3D ter melhorado a sensibilidade para 53%, essa modalidade apresentou capacidade inferior a RMC em detectar FEVE < 50%.^916^

A avaliação da massa do VE também merece atenção, uma vez que o tratamento quimioterápico pode modificá-la.^915^ Cerca de 50% das crianças sobreviventes de câncer apresentam massa do VE < 2 desvios-padrão que os valores habituais.^916^ Neilan et al.,^917^ estudando indivíduos com disfunção do VE tratados com antracíclicos (seguimento mediano de 88 meses), evidenciaram que a massa do VE indexada avaliada pela RMC foi um importante fator prognóstico. Pacientes com massa indexada do VE < 57 g/m^2^ apresentaram risco significativamente maior de eventos cardiovasculares, incluindo morte cardiovascular, choque apropriado do CDI e admissão por IC.^917 , 918^ Apesar da disfunção do VD ser um reconhecido fator prognóstico, poucos estudos investigaram especificamente os efeitos da terapia oncológica na morfologia e função do VD. Recentemente, a capacidade da RMC em detectar alterações morfológicas e funcionais do VD mesmo com doses moderadas de antracíclicos (240 mg/m^2^) foi documentada.^919^

#### 3.25.2. Caracterização Tecidual

#### 3.25.2.1. Fibrose Miocárdica

Apesar do RT representar uma importante estratégia para investigação de cicatriz e infarto no miocárdio, essa modalidade de imagem oferece uma avaliação parcial da fibrose presente no miocárdio.^418^ Como o RT baseia-se na diferença relativa da intensidade de sinal dos tecidos após a administração de contraste a base de gadolínio, essa técnica pode falhar em identificar a fibrose intersticial.^907^ Inúmeros estudos evidenciam que o RT não ocorre de maneira uniforme após a quimioterapia nos pacientes que desenvolvem cardiotoxicidade e sobretudo após os esquemas utilizando antracíclicos.^909 , 920 , 921^ Dessa maneira, a ausência de RT em paciente sob risco de desenvolver cardiotoxicidade após o tratamento contra o câncer não deve ser interpretada como ausência de lesão miocárdica.

#### 3.25.2.2. Mapas de T1

Numerosos estudos examinaram a utilidade dos mapas de T1 pela RMC para detectar o remodelamento miocárdico após quimioterapia. Utilizando medidas de T1 no miocárdio e na cavidade antes e após a administração de contraste, com a correção para o valor do hematócrito, é possível determinar também o volume do VEC.^922^ Em um estudo pediátrico, Tham mostrou existir associação positiva entre o VEC com a dose cumulativa da quimioterapia, assim como entre a incapacidade física avaliada pelo teste cardiopulmonar.^923^ Jordan et al., estudando um corte relativamente grande de pacientes com câncer, indicou que não apenas o VEC, mas também o T1 nativo foram significantemente mais elevados nos pacientes com câncer tratados, comparado aos controles.^924^ Utilizando a técnica de quantificação do tempo de vida intracelular das moléculas de água (TVIA) e a fração do volume da matriz extracelular, após administração de gadolínio, alguns estudos demonstraram a possibilidade da investigação do remodelamento miocárdico em nível celular, com validação histológica.^907 , 918 , 925 , 926^

Mais recentemente, Thavendiranathan et al. demonstraram que os mapas de T1 e T2 são úteis na investigação da cardiotoxicidade causada pelos inibidores de *checkpoint* .^927^ Estudando 136 pacientes que desenvolveram miocardite por inibidores de *checkpoint,* os autores mostram que as alterações nos mapas de T1 apresentaram não apenas utilidade diagnóstica, como identificaram os pacientes com risco elevado de apresentaram eventos cardiovasculares subsequentes.^927^

A Tabela 53 traz os principais cenários clínicos relacionados à avaliação e ao seguimento de pacientes com suspeita de cardiotoxicidade.

**Tabela 53:** Ressonância magnética cardíaca na pesquisa e seguimento de pacientes com suspeita de cardiotoxicidade

Indicação	Classe de recomendação	Nível de evidência
Avaliação da morfologia e função cardíaca (podendo incluir *strain* miocárdico) em suspeita de cardiotoxicidade^928 – 930^	I	A
Pesquisa de fibrose miocárdica (podendo incluir mapa T1 e volume extracelular) na avaliação e seguimento de portadores de cardiotoxicidade^930 – 932^	IIa	C
Avaliação de atividade inflamatória aguda (podendo incluir mapas T1 e T2) na avaliação e seguimento de portadores de cardiotoxicidade^931 , 932^	IIb	B
Suspeita de miocardite secundária a uso de inibidores de *checkpoint* ^927 , 928 , 933 , 934^	IIa	B

### 3.26. Doenças Vasculares

O emprego da RM no diagnóstico da patologia vascular vem sendo beneficiado por avanços nos equipamentos utilizados para essa finalidade, bem como por melhorias nos métodos de aquisição. A utilização de bobinas de melhor perfil e o emprego de sequências de pulso associadas a técnicas de aceleração permitiram a realização de exames com melhor resolução e sinal, com redução no tempo de aquisição das imagens.

A possibilidade da realização de estudos angiográficos que dispensam o uso de radiação ionizante é uma vantagem oferecida pela RM. Isso pode ser benéfico para situações que exijam exames seriados para acompanhamento de determinadas patologias vasculares, com consequente redução de radiação cumulativa. A utilização de contraste paramagnético baseado em gadolínio é extremamente segura. Reações de hipersensibilidade são muito incomuns, e a ocorrência de fibrose nefrogênica sistêmica (fundamentalmente relacionada à utilização em pacientes com disfunção renal importante e dialíticos) tem se provado um fenômeno cada vez mais raro e relacionado a perfis específicos de moléculas de gadolínio. Por não exercer nefrotoxicidade direta, a utilização de angio-RM com gadolínio pode ser considerada uma alternativa em pacientes cujas reduções na taxa de filtração glomerular limitem a utilização de TC com injeção de contraste iodado.

A seguir, são apresentados os diferentes contextos de avaliação de patologias vasculares pela RM. Nesta Diretriz, não serão abordadas as indicações de angio-RM de vasos intracranianos.

#### 3.26.1. Aorta

Assim como a TC, a RM desempenha papel fundamental na avaliação das doenças da aorta.^935^ É capaz de obter imagens multiplanares e volumétricas com excelente resolução espacial, permitindo a avaliação tridimensional tanto do lúmen quanto das paredes da aorta. Por ser um exame com tempo de realização significativamente mais longo que a TC, é menos utilizada nas doenças agudas.

A RM apresenta diversos tipos de sequências que permitem a obtenção de imagens com características distintas e alta capacidade de caracterização tecidual, o que pode ser utilizado para aumentar a acurácia diagnóstica. Resumidamente, para análise da aorta, pode-se utilizar sequências angiográficas, morfológicas e de análise de fluxo. Algumas delas são adquiridas com apenas uma apneia, e outras podem demorar minutos, o que exige a utilização de técnicas de sincronização cardíaca e respiratória para a obtenção de imagens livres de artefatos.

Entre as sequências angiográficas, as mais utilizadas são aquelas que utilizam meio de contraste. Há também técnicas recentes que permitem a realização de imagens angiográficas sem contraste, sendo que, em alguns segmentos do corpo, já atingem qualidade comparável às sequências angiográficas que utilizam contraste.^936^

Como as sequências angiográficas por RM são luminografias, é fundamental que também sejam realizadas sequências morfológicas para melhor avaliação da parede vascular e dos planos perivasculares. Uma das sequências mais utilizadas são as de "sangue escuro", que anulam o sinal do sangue e destacam as paredes vasculares e podem ser ponderadas em T1 (permitindo identificação de hematoma e realce pelo contraste) ou T2 (permitindo avaliação de edema). Atualmente, vem ganhando destaque uma série de sangue escuro chamada " *vessel wall* ", que tem como características cortes finos de alta resolução espacial, permitindo análise pormenorizada da parede vascular. A RM permite ainda análise qualitativa e quantitativa de fluxo, através de técnicas específicas; entre as mais utilizadas, estão a " *phase contrast* " e a "4D-Flow".

A RM é um método baseado em radiofrequência e, dessa forma, não utiliza radiação ionizante, o que é uma vantagem para pacientes que precisam realizar exames seriados. As principais patologias da aorta foram abordadas no capítulo de avaliação da aorta por TC.

A seguir ( Tabela 54 ), encontram-se as principais recomendações da utilização de RM no diagnóstico das patologias da aorta.

**Tabela 54:** Indicações da utilização de ressonância magnética no diagnóstico da patologia da aorta

Indicação	Classe de recomendação	Nível de evidência
Aneurisma da aorta^419 , 937 – 940^	I	B
Dissecção crônica da aorta^419 , 937 , 938^	I	B
Hematoma intramural aórtico^419 , 937 – 941^	I	B
Úlceras aórticas^419 , 937 , 938^	I	C
Planejamento de abordagem cirúrgica da aorta^419 , 937^	I	B
Planejamento de endoprótese aórtica^419 , 937 , 942^	I	B
Arterites de grandes e médios vasos^943 , 944^	I	B
Dissecção aguda da aorta^419 , 937 , 941^	IIa	B
Avaliação pós-operatória de endoprótese aórtica^945 – 947^	IIb	B
Mensuração dos calibres da aorta^419 , 937^	I	C
Síndrome aórtica aguda em paciente instável^419 , 937 , 938^	III	C
Sindrome aórtica aguda em paciente estável^419 , 937 , 938 , 941^	IIa	C
Lesão traumática da aorta^937 , 938 , 948^	IIb	C

#### 3.26.2. Carótidas Extracranianas

A angio-RM de carótidas permite o estudo de diferentes aspectos da manifestação da aterosclerose carotídea, incluindo a patência de fluxo, o calibre dos vasos, a presença de estenoses e as características das paredes do vaso, além de ser capaz de avaliar uma série de doenças vasculares, a anatomia vascular e as relações entre os vasos e outras estruturas anatômicas e lesões cervicotorácicas, do pescoço e cervicocranianas.

As principais indicações para a realização de angio-RM de carótidas são semelhantes às indicações da angio-TC de carótidas.^949^ A angio-RM e a angio-TC de carótidas têm acurácias maiores do que o ultrassom na avaliação de estenose carotídea,^408 , 950^ existindo a recomendação de realização daquelas modalidades nos casos que se necessite a confirmação do grau de estenose para nortear a conduta.^951^ Como a acurácia da ressonância e da TC são semelhantes para a avaliação de estenose carotídea, a escolha entre os métodos deve levar em consideração contraindicações específicas do paciente para cada um dos métodos (por exemplo, alergia a contraste iodado, exposição a radiação ionizante), o grau de emergência da avaliação (dando-se preferência à TC em casos de emergência, pela maior rapidez de aquisição) e a disponibilidade dos métodos.

A Tabela 55 traz as principais indicações da RM na avaliação das artérias carótidas extracranianas.

**Tabela 55:** Emprego da ressonância magnética na avaliação de artérias carótidas extracranianas

Indicação	Classe de recomendação	Nível de evidência
Avaliação de estenoses de artérias carótidas^952 , 953^	I	B
Avaliação de composição de placa em artérias carótidas^953 , 954^	IIb	C
Avaliação de carótidas em vigência de acidente vascular encefálico agudo, quando a TC foi contraindicada ou inconclusiva^953 , 955 , 956^	IIa	C

#### 3.26.3. Artérias Renais

A angio-RM das artérias renais com contraste para a avaliação das artérias renais já é um método aceito há muitos anos para estudo do trajeto, calibre, variações anatômicas e estenoses das artérias renais, inclusive para planejamento pré-nefrectomias.^419^ Há também literatura recente sugerindo que sequências dinâmicas após a injeção do contraste podem ser utilizadas para quantificar a perfusão renal.^957^ A recente introdução de técnicas de denervação simpática para tratamento da hipertensão arterial persistente^958 – 960^ também trouxe interesse na imagem da anatomia das artérias renais e quantificação não invasiva da função renal e até mesmo cardiorrenal por angio-RM.^419 , 961^ Para o diagnóstico de estenoses em artérias renais, novas sequências de aquisição, inclusive sem a injeção do meio de contraste paramagnético, têm se mostrado bastante promissoras.^962 – 964^ Já as sequências com contraste estão consolidadas para o diagnóstico de estenoses nas artérias renais, com alta eficácia. Uma metanálise estabeleceu sensibilidade e especificidade de 97% e 93%, respectivamente, em comparação à arteriografia.^965^

A seguir ( Tabela 56 ), encontra-se a recomendação da utilização da RM na avaliação das artérias renais.

**Tabela 56:** Utilização da ressonância magnética cardíaca na avaliação das artérias renais

Indicação	Classe de recomendação	Nível de evidência
Avaliação de estenoses das artérias renais^965 – 967^	I	B

#### 3.26.4. Artérias Pulmonares

A RM pode ser utilizada para avaliação das artérias pulmonares, com bom desempenho para análise de anatomia, calibre e perviedade. Permite também análise qualitativa e quantitativa de fluxo, através de técnicas específicas.

No entanto, não apresenta bom desempenho para análise de TEP^968^ devido à sua baixa sensibilidade, alta proporção de exames inconclusivos, baixa disponibilidade na maioria das salas de emergência e tempo de aquisição mais longo quando comparado à TC. Na impossibilidade de realizar os métodos convencionais para pesquisa de TEP, tais como a angio-TC e a cintilografia, a RM se torna uma alternativa plausível.^969^ Para pesquisa de TEP por ressonância, recomenda-se a realização de sequências angiográficas com gadolínio, que apresentam maior acurácia para análise intravascular.

As principais patologias das artérias pulmonares foram abordadas no capítulo de avaliação das artérias pulmonares por TC.

A Tabela 57 traz os principais cenários nos quais a RM contribui para a avaliação das artérias renais.

**Tabela 57:** Avaliação das artérias pulmonares pela ressonância magnética

Indicação/cenário clínico	Classe de recomendação	Nível de evidência
Tromboembolismo pulmonar (como alternativa à tomografia)^419 , 969 , 970^	IIa	B
Calibre do tronco pulmonar e das artérias pulmonares principais^419 , 971^	I	B

#### 3.26.5. Artérias Viscerais

Estenoses do tronco celíaco e artérias mesentéricas também podem ser diagnosticadas com angio-RM com contraste. Apesar de a angio-TC ser geralmente preferida, conforme discutido na sessão de TC desta publicação, a angio-RM também pode trazer informações a respeito de estenoses e circulação colateral, bem como sobre doenças não ateroscleróticas como displasia fibromuscular e compressão do ligamento arqueado mediano pelo diafragma,^972^ porém com eficácia inferior à angio-TC com contraste, sobretudo nas porções mais distais dos vasos mesentéricos.^419^

A RM ganha papel relevante quando existe uma preocupação a respeito da radiação ionizante ou da nefrotoxicidade do contraste iodado que são usados na angio-TC. A Tabela 58 traz o papel da RM na avaliação das artérias viscerais.

**Tabela 58:** Utilização da ressonância magnética na avaliação das artérias viscerais

Indicação	Classe de recomendação	Nível de evidência
Avaliação das artérias mesentéricas e tronco celíaco^419 , 953 , 973^	IIa	C

## 4. Cardiopatias Congênitas

### 4.1. Tomografia Computadorizada na Avaliação de Cardiopatias Congênitas

A TCMD encontra na população de cardiopatas congênitos uma grande perspectiva de contribuição. Trata-se de um exame de execução rápida, muitas vezes dispensando necessidade de sedação, o que lhe confere praticidade e possibilidade de realização em basicamente todos os pacientes, mesmo naqueles instáveis. Entretanto, como demanda da utilização de radiação ionizante, deve ter seu uso criteriosamente considerado (sobretudo nos exames realizados repetidamente), haja vista o efeito cumulativo a longo prazo da utilização dessa modalidade. Além disso, a necessidade da utilização de contraste iodado deve ser avaliada no contexto de pacientes com alteração da função renal.

A TCMD é um exame que se baseia na definição anatômica das alterações relacionadas às estruturas cardiovasculares, bem como na avaliação pós-operatória dessas condições. Tem um papel fundamental na identificação da perviedade de *stents* , tubos e condutos e nas complicações a eles relacionadas. Alterações vasculares extracardíacas que frequentemente acompanham as patologias de base, bem como o resultado de anastomoses pós-cirúrgicas (por exemplo, derivações cavo-pulmonares parciais ou totais [cirurgias de Glenn/Fontan], derivações sistêmico-pulmonares [procedimento de Blalock-Taussig]), têm na TC uma excelente ferramenta de avaliação.

Embora não seja sua utilidade primária, a TC do coração com sincronização eletrocardiográfica permite avaliação da função e volume ventriculares. Em casos de dúvida ou impossibilidade de realização de imagens adequadas pelo ecocardiograma ou RMC, é possível avaliar esses parâmetros por meio da utilização da TC. Da mesma maneira, a avaliação morfologófica e funcional de valvas cardíacas (mobilidade de folhetos/calcificações/orifícios regurgitantes) é factível em casos selecionados.

Dada a especificidade de cada patologia cardíaca congênita, a aplicação da TCMD em diferentes cenários clínicos será tratada individualmente nos tópicos dedicados a cada uma das cardiopatias congênitas descritas nesta Diretriz.

#### 4.1.1. Avaliação de Shunts Intra e Extracardíacos

#### 4.1.1.1. Comunicação Interatrial e Interventricular

A avaliação de comunicação interatrial (CIA) e comunicação interventricular (CIV) é feita de forma bastante adequada e conclusiva pelo ecocardiograma, sendo a TC e RM reservadas principalmente para aqueles casos em que o ecocardiograma não foi completamente elucidativo, em especial na avaliação pré-operatória, como nos casos de CIAs do tipo seio venoso, muitas vezes associados a drenagem anômala de veias pulmonares.^974 – 976^

Em relação à RMC, a TC mostra a desvantagem de uma avaliação mais limitada nas repercussões dos defeitos, detectando somente dilatações de vasos mediastinais e câmaras cardíacas, tendo, portanto, um papel menor no seguimento desses indivíduos.^974^ Uma análise funcional contrátil é possível, mas à custa de dose mais alta de radiação. Por outro lado, a TC tem superioridade nas medidas das comunicações intercavitárias,^977^ quando houver uma complexidade geométrica septal como nos casos de comunicações interventriculares musculares múltiplas.

Após fechamento percutâneo desses *shunts* , a RMC ou TC podem ser indicadas se houver suspeita de complicações, como infecção, má posição, embolização ou *shunt* persistente.^978^

A seguir, encontram-se os principais cenários clínicos relacionados à utilização da TC na avaliação de *shunts* intracardíacos (comunicações interatrial e interventricular e defeitos do septo atrioventricular [ Tabelas 59 , 60 e 61 respectivamente]) e extracardíacos (persistência do canal arterial – Tabela 62 ).

**Tabela 59:** Comunicação interatrial (CIA)

Indicação	Classe de recomendação	Nível de evidência
Avaliação pré-operatória para correção de CIA, anatomia septal e pesquisa de aneurismas do septo^974 , 979^	IIa	C
Avaliação pré-operatória de correção de CIA do tipo seio venoso e/ou conexão anômala das veias pulmonares (CAVP) parcial e seio coronário^974 , 975 , 980^	I	B
Seguimento pós-operatório ou pós-intervenção percutânea de CIA, pacientes com *shunt* residual significativo, disfunção valvar ou ventricular, arritmias e/ou hipertensão pulmonar^981^	IIb	C
Seguimento pós-intervenção percutânea de CIA ou forame oval patente (FOP), suspeita de complicações, embolização ou *shunt* persistente^978^	IIb	C
Seguimento pós-intervenção percutânea de CIA para avaliação de má posição da prótese^978^	IIa	C

**Tabela 60:** Comunicação interventricular (CIV)

Indicação	Classe de recomendação	Nível de evidência
Avaliação pré-operatória de correção de CIV de anatomia complexa^982 , 983^	IIa	C
Seguimento pós-operatório ou pós-intervenção percutânea de CIV, pacientes com *shunt* residual significativo, disfunção valvar ou ventricular, arritmias e/ou hipertensão pulmonar^984^	IIb	C

**Tabela 61:** Defeito do septo atrioventricular (DSAV)

Indicação	Classe de recomendação	Nível de evidência
Avaliação pré-operatória de correção de DSAV^975 , 976^	IIa	C
Seguimento pós-operatório de DSAV, pacientes com *shunt* residual significativo, disfunção valvar ou ventricular, obstrução da via de saída do ventrículo esquerdo (VSVE), arritmias e/ou hipertensão pulmonar^974^	IIa	C
Seguimento pós-operatório de DSAV, pacientes com sinais de falência cardíaca^974^	IIb	C

**Tabela 62:** Persistência do canal arterial (PCA)

Indicação	Classe de recomendação	Nível de evidência
Avaliação pré-operatória de correção de PCA^983^	IIb	C
Avaliação pós-tratamento com suspeita de complicação em território aórtico ou pulmonar	I	C

#### 4.1.1.2. Conexão Venosa Anômala Parcial e Total

A avaliação da anatomia da drenagem venosa pulmonar anômala por TC é bastante comparável à RMC, permitindo uma investigação pré-operatória precisa e completa,^985^ sendo bem indicada na avaliação pré-operatória desses pacientes.^974 , 976^

Assim como nos defeitos septais, uma desvantagem da TC é a avaliação funcional limitada, em relação às repercussões cardíacas das lesões, sendo menos indicada no seguimento desses pacientes.^974^ Por outro lado, a TC mostra bem repercussões pulmonares desses defeitos, permitindo identificar sinais de congestão pulmonar, com edema intersticial ou edema alveolar, ou ainda derrames cavitários.

Nos casos de síndrome venolobar congênita ("cimitarra"), a avaliação de malformações broncopulmonares associadas é fundamental, assim como a presença de áreas de irrigação pulmonar sistêmica, ambas feitas de forma bastante precisa por TC. A seguir ( Tabela 63 ), são apresentados os cenários clínicos relacionados à utilização da TC na avaliação das conexões anômalas das veias pulmonares.

**Tabela 63:** Conexão anômala de veias pulmonares (CAVP)

Indicação/cenário clínico	Classe de recomendação	Nível de evidência
Avaliação pré-operatória de correção de CAVP^980 , 983^	I	B
Complicaçõea atribuíveis à CAVP, durante seguimento pós-tratamento^977 , 985^	I	C
Seguimento pós-operatório de conexão anômala de veias pulmonares total, pacientes assintomáticos, com pouca ou nenhuma sequela	IIa	C

#### 4.1.2. Lesões Congênitas Valvares

Inúmeros pacientes com cardiopatias congênitas necessitam de nova intervenção para reabordagem das valvas cardíacas, comumente em até mais de uma valva. Em pacientes jovens submetidos à troca da válvula mitral, 50% necessitarão de substituição dentro de 10 anos e 15% requerem a colocação de marca-passo em até 1 mês do implante de próteses. Existem vários estudos que mostram a utilidade da TC cardíaca para avaliação de estenose e insuficiência valvar nativa e mecânica, vazamento perivalvar, trombose, abscesso e endocardite. As diferenças de volume sistólico entre os ventrículos calculadas a partir de um conjunto de dados funcional podem ser usadas para quantificar a gravidade da regurgitação valvar e se correlacionam intimamente com os achados ecocardiográficos.^986 , 987^

#### 4.1.2.1. Valva Tricúspide/Anomalia de Ebstein

Os volumes ventriculares e as FE podem ser obtidos por angio-TC cardíaca com sincronização eletrocardiográfica de maneira semelhante à imagem da RMC em pacientes que não podem ser submetidos à ressonância. A morfologia e a mobilidade dos folhetos podem ser visualizadas nas reconstruções de cine. A angio-TC pode ser uma alternativa à RMC também quando for necessário o estudo das artérias coronárias. Entretanto, não é possível a quantificação de fluxos pela angio-TC e avaliação da severidade da regurgitação tricúspide.^988^

#### 4.1.2.2. Valva Pulmonar

A angio-TC tem a vantagem de cobrir todo o tórax com estudo de alta resolução e é capaz de representar detalhes anatômicos da valva pulmonar, estruturas perivalvares e ramos da artéria pulmonar. É a modalidade de escolha para avaliação das artérias pulmonares proximais e distais, bem como a proximidade da origem e trajeto da artéria coronária. Porém, como a resolução temporal é menor que a da RMC e do ecocardiograma, sua aplicação para avaliação funcional da valva pulmonar é limitada. Pode ser uma opção para avaliar a valva pulmonar em pacientes em que a RM não é viável, especialmente quando é necessária descrição da anatomia e complicações pós-cirúrgicas, avaliação do tamanho e função ventricular. Em *scanners* de dupla fonte com resolução temporal mais rápida, a qualidade da imagem é melhor reduzindo artefatos de movimento. A angio-TC também pode fornecer informações valiosas sobre as características morfológicas, dilatação pós-estenótica e a localização de uma estenose supra ou subvalvar.^989 , 990^ As vegetações que afetam a valva pulmonar são vistas ao ecocardiograma em 50% dos pacientes com endocardite. Essas vegetações podem ser acompanhadas por concomitante espessamento, encurtamento, perfurações ou destruição completa das cúspides, causando regurgitação pulmonar. Pseudoaneurismas podem se formar no local onde há um abscesso perivalvar desaguando no coração ou no lúmen do vaso. As fístulas podem causar *shunt* e vazamento perivalvar. A angio-TC com sincronização eletrocardiográfica é excelente para avaliação de todas essas anormalidades estruturais associadas à endocardite da valva pulmonar, entretanto seu uso pode ser limitado para avaliação de pequenas vegetações (< 4 mm) e pequenas perfurações valvares.^991^

Como na valva aórtica, a TC fornece informações valiosas para o implante percutâneo da válvula pulmonar. As indicações para uma abordagem transcateter são semelhantes às da cirurgia. Além disso, as características morfológicas da via de saída do ventrículo direito são um critério importante para elegibilidade para substituição da valva transcateter, uma determinação que pode ser facilmente feita em imagens de TC ou RM. Pacientes com aparência aneurismática não são elegíveis para implante de valva pulmonar transcateter. As complicações do implante da valva incluem migração do *stent* , fratura do *stent* , ruptura do conduto, compressão da artéria coronária, hemorragia pulmonar e endocardite na prótese. Tais complicações podem ser muito bem avaliadas pela angio-TC.^990^

#### 4.1.2.3. Valva Mitral

As anomalias congênitas da valva mitral incluem aquelas associadas a defeitos do septo atrioventricular (AV), valva mitral em paraquedas e mitral com duplo orifício. O espectro dos defeitos do septo AV total, que são compostos por uma valva AV comum, comunicação interatrial *ostium primum* e comunicação interventricular de via de entrada; defeitos parciais, que são constituídos por valvas AV esquerda e direita separadas, um folheto da valva AV esquerda com fenda e CIA *ostium primum* e várias categorias intermediárias entre essas duas extremidades do espectro. Uma valva mitral em paraquedas ocorre quando todas as cordoalhas surgem de um único músculo papilar fundido. Essa anormalidade está associada à estenose mitral de vários graus e à síndrome de Shone. A síndrome de Shone é caracterizada por valva mitral em paraquedas, anel supravalvar mitral, estenose subvalvar aórtica e coarctação da aorta. Uma valva mitral com duplo orifício é caracterizada por uma ponte de tecido que divide o ânulo em dois orifícios, ambos abertos para o VE. Essa condição está associada a vários graus de estenose ou regurgitação mitral e tem fortes associações com anomalias valvares adicionais e outros defeitos cardíacos congênitos.

Em geral, o ecocardiograma avalia muito bem os defeitos que acometem a valva mitral, não necessitando investigação complementar para decisão terapêutica e/ou seguimento desse grupo de pacientes. A angio-TC tem grande utilidade em pacientes com espectros variados da síndrome de Shone pelo poder de avaliação dos múltiplos achados. Nos casos em que há necessidade de troca valvar, podemos usar a TC para avaliações pós-operatórias por causa de sua resolução espacial superior e menor suscetibilidade a artefatos metálicos. A cine-TC tem demonstrado excelente utilidade na identificação de abscessos perivalvares e vegetações valvares.^992^

#### 4.1.2.4. Valva Aórtica

Para a avaliação da valva aórtica bicúspide e estenose valvar aórtica isolada, geralmente não é necessário complementação diagnóstica ao ecocardiograma. Em pacientes com estenoses sequenciais subvalvar, valvar e supravalvar aórtica, a angio TC pode acrescentar informações adicionais ao ecocardiograma, caracterizando a via de saída do ventrículo esquerdo quando é alongada, tipo "túnel" ou na presença de pequenas membranas que, muitas vezes, podem não ser visualizadas na RMC, pela menor resolução espacial. Essas informações adicionais podem contribuir para o planejamento cirúrgico.^993^ A seguir ( Tabela 64 ), são apresentadas as principais indicações da utilização da tomografia na avaliação de lesões congênitas valvares.

**Tabela 64:** Lesões congênitas valvares

Indicação	Classe de recomendação	Nível de evidência
Avaliação complementar de lesões valvares regurgitantes^987 , 994^	IIb	B
Avaliação complementar de lesões valvares estenóticas^989 , 995 , 996^	IIa	B
Avaliação pré-operatória para correção de anomalia de Ebstein (valva tricúspide e ventrículo direito e árvore pulmonar)^988^	IIa	C
Pós-correção de Ebstein/disfunção valvar ou ventricular	IIb	C

#### 4.1.3. Anomalias Conotruncais

#### 4.1.3.1. Tetralogia de Fallot

Na avaliação pré-operatória, a maioria das informações diagnósticas é fornecida pelo ecocardiograma. A TC pode fornecer dados complementares quando o eco não foi completamente conclusivo, como na associação com anomalias coronarianas e nos casos mais complexos com atresia pulmonar, em que a avaliação de colaterais e da segmentação da vasculatura pulmonar se faz necessária.^997 – 999^

Nos casos de tetralogia de Fallot com agenesia da valva pulmonar, a TC se mostra útil na avaliação de compressão de vias aéreas centrais por estruturas vasculares, decorrentes das dilatações aneurismáticas das artérias pulmonares.

No pós-operatório, identifica complicações cirúrgicas nas reconstruções da via de saída do ventrículo direito, no tronco pulmonar e na árvore vascular proximal e de seu leito mais periférico, além de ser um melhor método para avaliação de estenoses em topografia dos *stents* .^1000 , 1001^

Pode ser um método alternativo para a quantificação da volumetria e da função ventricular nos casos em que a RM é contraindicada ou limitada por dispositivos implantados, possibilitando o monitoramento para a reabordagem nos defeitos residuais. A Tabela 65 traz as indicações da TC na avaliação de pacientes com tetralogia de Fallot.

**Tabela 65:** Tetralogia de Fallot

Indicação	Classe de recomendação	Nível de evidência
Avaliação pré-operatória nos casos de anatomia desfavorável ou se houver limitação pelo ecocardiograma^999 , 1001^	I	C
Avaliação no pós-operatório antes da substituição planejada da valva pulmonar (percutânea [Melody→] ou cirúrgica)^262 , 1002 , 1003^	IIa	C
Avaliação com obstrução da via de saída do ventrículo direito, estenose do território pulmomar, arritmias ou presença de um conduto ventrículo direito-artéria pulmonar.^1004^	I	C

#### 4.1.3.2. Dupla Via de Saída de Ventrículo Direito

A dupla via de saída de VD não é uma anomalia cardíaca única, mas um grupo heterogêneo com características morfológicas variáveis. A posição anormal dos grandes vasos em associação a várias anomalias estruturais pode levar a diferentes fenótipos fisiológicos.^1005 , 1006^ Representa um espectro complexo que consiste em posição variada da comunicação interventricular em relação às valvas aórtica e pulmonar, bem como graus variados de mal posicionamento das grandes artérias. Um conjunto de dados tridimensionais pela TC podem auxiliar na localização da comunicação interventricular para um planejamento cirúrgico adequado.^1007^ Nesses pacientes, o tamanho e a posição do defeito septal interventricular em relação às grandes artérias são importantes para a compreensão da fisiologia e para a determinação do reparo cirúrgico.^1008 , 1009^

O preciso relacionamento topográfico com as artérias é fundamental para estimar dificuldades, prever ampliações da comunicação interventricular, reinserir o aparato da valva atrioventricular ou reduzir a cavidade ventricular no redirecionamento dos vasos. Imagens tridimensionais da anatomia intracardíaca nesse cenário irão determinar se um túnel intraventricular é viável e se a exposição através do AD e valva tricúspide é suficiente, de modo que uma ventriculotomia direita possa ser evitada.^1010^

Tomógrafos de gerações atuais com alta resolução espacial e temporal e algoritmos de redução de dose com ferramentas avançadas de pós-processamento tridimensional fornecem uma alternativa de baixo risco e alta qualidade ao cateterismo cardíaco diagnóstico e têm sido cada vez mais utilizados nesses arranjos anatômicos mais complexos. A seguir ( Tabela 66 ), são apresentados os principais cenários clínicos de utilização da TC na avaliação da dupla via de saída de ventrículo direito.

**Tabela 66:** Dupla via de saída de ventrículo direito

Indicação	Classe de recomendação	Nível de evidência
Avaliação pré-operatória da anatomia vascular e da relação da comunicação interventricular^1007 – 1009^	I	C
Avaliação de disfunção ventricular, obstrução da via de saída do ventrículo direito ou esquerdo, estenose de território pulmonar, arritmias ou presença de um conduto ventrículo direito-artéria pulmonar.^1010^	IIa	C

#### 4.1.3.3. Tronco Arterial Comum

O ecocardiograma é a ferramenta diagnóstica principal, mas, quando há dúvida na análise de estruturas extracardíacas, a TC pode ser muito útil para delinear a anatomia extracardiaca do tronco arterial comum.^1011^ A avaliação pré-operatória deve incluir o delineamento da anatomia vascular, principalmente do território pulmonar elucidando a distância entre as artérias pulmonares, se existe disposição vascular cruzada ou se sua emergência se dá na junção sinotubular ou abaixo, próximo do seio da valva truncal.^1012^ A proposta cirúrgica vai depender das características morfológicas, e a presença de anomalias associadas influencia no resultado cirúrgico e na mortalidade dos pacientes.

Entre as associações, podemos ter graus variáveis de coarctação até interrupção do arco aórtico. A TC contribui na avaliação vascular, identificando de maneira precisa a origem e o trajeto das artérias pulmonares e caracterizando os graus de malformação do arco aórtico.^1013^ Além disso, pela resolução espacial da TC, pode trazer informação diagnóstica a respeito da circulação coronariana com a delimitação clara de ambos os óstios coronarianos e o trajeto epicárdico. Alterações nesse território podem ocorrer e incluem coronária única, anomalias de trajeto epicárdico proximal, trajetos intramurais e anomalias ostiais (óstios puntiformes ou em fenda). A presença de anomalia coronariana consiste em fator de risco para mortalidade tardia após a cirurgia corretiva.^1014 , 1015^

Na avaliação pós-operatória, a continuidade do VD e o território pulmonar através de conduto devem ser estudados, excluindo-se pontos de redução luminal significativa ou distorções vasculares locais. Proximidades da reconstrução cirúrgica de outras estruturas, como circulação coronariana ou região retrosternal, são de importância em caso de reabordagem cirúrgica.

Nos casos em que houve correção no território aórtico, a TC é útil na caracterização no local da anatomose, excluindo estreitamento no local anastomótico, dilatações aneurismáticas ou outras complicacões locais.^1010 , 1016^ O papel da TC na avaliação do tronco arterial comum é apresentado na Tabela 67 .

**Tabela 67:** Tronco arterial comum

Indicação/cenário clínico	Classe de recomendação	Nível de evidência
Avaliação pré-operatória de anatomia para correção^1011 , 1012^	I	C
Avaliação no pós-operatório - comunicação interventricular residual conhecida, presença de estenose em conduto ventrículo direito-artéria pulmonar ou território pulmonar^1014 – 1016^	I	C
Avaliação no pós-operatório: estenose ou regurgitação da valva truncal^1017^	IIa	C

#### 4.1.3.4. Transposição das Grandes Artérias

Na avaliação pré-operatória, a TC pode ser um método útil na complementação diagnóstica para análise de anomalias coronarianas além de lesões associadas como obstruções no arco aórtico.^1018^

Após a cirurgia de Jatene, a avaliação cuidadosa dos óstios e da porção proximal da coronária é mandatória, já que são transferidas da aorta nativa para a neoaorta (pulmonar nativa) durante a troca arterial. A TC é a técnica de imagem não invasiva de escolha para a avaliação do território coronariano, incluindo a avaliação ostial e trajeto epicárdico proximal após reimplante. Angulação e tração podem ocorrer com consequente isquemia e dano miocárdico, estando a TC indicada para a avaliação de distorções ou redução luminal das artérias coronárias.^1019 – 1021^

O território pulmonar e o território sistêmico também são estudados no intuito de detectar estenose nos locais de sutura. A estenose pulmonar pode ocorrer tanto em topografia supravalvar como nas artérias pulmonares, consequência da disposição vascular após a realização da manobra de Lecompte, bem como a distorções locais pela dilatação da raiz da neoaorta.^1022^

Na correção em nível atrial, é possível delimitar bem as tunelizações venosas, tanto no território venoso pulmonar como das veias cavas superior e inferior. No caso de impossibilidade da avaliação do ventrículo sistêmico pela RM, é possível a avaliação volumétrica e funcional da cavidade ventricular direita pela TC como alternativa diagnóstica.^1023^ A Tabela 68 (a seguir) apresenta as indicações de utilização da TC na avaliação da transposição das grandes artérias.

**Tabela 68:** Transposição das grandes artérias

Indicação	Classe de recomendação	Nível de evidência
Avaliação pré-operatória de anatomia para correção^1018^	IIa	C
Pós- cirurgia de Jatene – avaliação coronariana^1019 – 1021 , 1024^	I	C
Pós-cirurgia de Jatene: disfunção valvar ou ventricular moderada, obstrução da via de saída do ventrículo direito ou esquerdo, estenose de território pulmonar ou arritmias	IIa	C
Pós-cirurgia de Jatene: raiz neoaórtica dilatada ou regurgitação neoaórtica^1022^	IIa	C
Pós-correção atrial (por exemplo, Senning): regurgitação atrioventricular sistêmica, disfunção sistêmica do ventrículo direito, obstrução da via de saída do ventrículo esquerdo ou arritmias	I	C
Pós-correção atrial (por exemplo, Senning): avaliação de tunelizações venosas^1023^	I	C

#### 4.1.3.5. Transposição Corrigida das Grandes Artérias

A TC permite a caracterização da discordância atrioventricular e ventriculoarterial no pré-operatório, bem como outros defeitos associados, e serve como uma modalidade alternativa para pacientes nos quais a RM está contraindicada, sendo particularmente útil no delineamento preciso da anatomia coronariana. Caracteriza bem a morfologia ventricular, demonstrando a inversão ventricular bem como o padrão invertido da emergência coronariana, além da relação espacial entre a aorta e o tronco pulmonar. Entre os defeitos associados, localiza espacialmente a CIA, demonstrando sua repercussão hemodinâmica. Graus variáveis de obstrução na via de saída do ventrículo esquerdo podem estar presentes, desde alterações subpulmonares ou na valva pulmonar.^1010 , 1025^

A TC pode contribuir com uma análise completa no pós-operatório em nível arterial e atrial ( *double switch* ), incluindo não só a avaliação de reimplante coronariano, anastomoses arteriais bem como os redirecionamentos venosos em nível atrial.^1026^ Quando é realizado apenas o reparo dito fisiológico, a TC pode auxiliar na avaliação da correção de pontos de obstrução do território pulmonar ou se há *shunt* residual após o fechamento da CIV. A Tabela 69 traz os principais cenários clínicos relacionados ao emprego da TC na avaliação da transposição corrigida das grandes artérias.

**Tabela 69:** Transposição corrigida das grandes artérias

Indicação	Classe de recomendação	Nível de evidência
Avaliação pré-operatória para correção da anatomia^1010 , 1025^	I	C
Pós-operatório: insuficiência da valva atrioventricular sistêmica, disfunção sistêmica do ventrículo direito	IIa	C
Pós-operatório: disfunção do conduto ventrículo esquerdo-artéria pulmonar.^1026 , 1027^	I	C

#### 4.1.4. Anomalias da Aorta Torácica

#### 4.1.4.1. Coarctação e Outras Anormalidades da Aorta

Assim como a RMC, a TC constitui um método complementar que permite uma completa visualização não invasiva da aorta no diagnóstico dessas malformações.^975 , 977 , 993 , 1028^ Numerosas anomalias podem ser diagnosticadas, como coarctação, hipoplasia, interrupção e outras anomalias do arco aórtico, assim como outras lesões associadas.^977 , 1029^ Sua precisão é um pouco superior à RMC, devido à elevada resolução espacial.

Algumas doenças estão associadas à formação de anéis vasculares. A TC tem grande importância no estudo das vias aéreas e traz informações muitas vezes não providas por outras modalidades diagnósticas, demonstrando simultaneamente a anomalia vascular e a repercussão nas estruturas aeradas.^977^ A compressão de vias aéreas centrais de origem vascular pode ser decorrente de várias situações, como anomalias de arco aórtico, *sling* pulmonar, tetralogia de Fallot com agenesia de valva pulmonar ou em situações de dilatação de território pulmonar.^977 , 1029 – 1032^ Vale lembrar que a avaliação de compressão esofágica por TC é limitada, muitas vezes sendo necessária a complementação com outros métodos, como a esofagografia.^1029^

Avaliações subsequentes, após a correção da coarctação ou interrupção, são recomendadas para visualização da geometria do arco e exclusão de complicações, como reestenose, aneurismas locais ou dissecções. Nesse seguimento, deve-se dar preferência à RMC, pela ausência de radiação ionizante.^975^ Porém, nos casos pós-implante de *stent* ferromagnético, a TC tem superioridade na avaliação por ocasionar menos artefatos locais que podem limitar a avaliação luminal.^984 , 1030 , 1033 , 1034^ Complicações locais após a implantação de endopróteses incluem estenose residual, fratura, dissecção, *endoleak* ou aneurisma local, sem a ocorrência de artefatos como na RMC. As Tabelas 70 e 71 apresentam o papel da TC na avaliação da coarctação da aorta e nas anomalias do arco aórtico.

**Tabela 70:** Coarctação da aorta (CoAo)

Indicação	Classe de recomendação	Nível de evidência
Avaliação pré-operatória de correção de CoAo^975 , 984^	I	B
CoAo em seguimento pós-tratamento, com mudança clínica atribuível ao defeito (suspeita de recoarctação?)^984 , 1033^	I	C
Seguimento no primeiro ano pós-tratamento percutâneo (6-12 meses), paciente assintomático com nenhuma ou discreta sequela^1035 , 1036^	IIa	B
Seguimento após o primeiro ano pós-tratamento, paciente assintomático com nenhuma ou discreta sequela, intervalo de 1 a 2 anos^1037^	IIa	B
Seguimento pós-tratamento, paciente assintomático, para avaliar aneurisma de arco e/ou *stent* (reestenose, fratura ou *endoleak* )^1030 , 1034^	I	B
Seguimento pós-tratamento, paciente com sintomas de falência cardíaca^975 , 977 , 984 , 993 , 1028 , 1034^	IIa	B

**Tabela 71:** Anomalias do arco aórtico

Indicação	Classe de recomendação	Nível de evidência
Avaliação de anel vascular, vias aéreas e parênquima pulmonar^1029 , 1031^	I	B
Avaliação de interrupção de arco aórtico^1038^	I	C
Avaliação de janela aortopulmonar	I	C

#### 4.1.5. Coração Univentricular

Nos últimos anos, o uso da angio-TC em pacientes pediátricos aumentou rapidamente devido à excelente resolução espacial e temporal, e exames rápidos usando tomógrafos de última geração têm sido aplicados em cardiopatias complexas tipo univentricular, condição em que, após as cirurgias, apenas um ventrículo sustenta a circulação sistêmica.^1039^ A alta resolução espacial da angio-TC permite excelente avaliação de pequenas estruturas, além da vantagem da avaliação global das vasculaturas extracardíacas e suas relações com as demais estruturas torácicas. Nesse cenário, a correção cirúrgica é univentricular, com cirurgias paliativas com 2 a 3 estágios.

O primeiro estágio, se necessário e dependente da fisiologia, é realizado no recém-nascido e geralmente envolve um procedimento de Norwood, *shunt* arterial sistêmico pulmonar ou bandagem pulmonar. Alguns centros defendem uma abordagem "híbrida" utilizando um *stent* no canal arterial colocado por cateter e bandagem seletiva das artérias pulmonares.^986 , 1040^

#### 4.1.5.1. Antes da Cirurgia de Estágio 1

Enquanto muitos pacientes com coração univentricular podem ser visualizados adequadamente usando o ecocardiograma antes do primeiro estágio, a angio-TC é ocasionalmente necessária para melhor definir a anatomia complexa particularmente quando o eco não consegue definir a anatomia vascular extracardíaca.^986^ Em algumas situações, uma descrição detalhada da anatomia do canal arterial para planejamento de implante de *stent* em anatomias mais complexas e desafiadoras.^1041^

#### 4.1.5.2. Após a Cirurgia de Estágio 1 (Norwood, Shunt Arterial Sistêmico Pulmonar, Procedimento Híbrido)

Entre os estágios 1 e 2 da cirurgia, as estenoses da artéria pulmonar são relativamente comuns e, muitas vezes, não bem visualizadas ao ecocardiograma. A presença de estenoses mais distais ou segmentares nos ramos pulmonares secundários a retração ou nos locais das anastomoses dos *shunts* ou nas bandagens seletivas é bem visualizada pela angio-TC.

Pacientes com *shunts* sistêmicos para a artéria pulmonar ocasionalmente apresentam trombose de *shunt* , resultando em cianose profunda e aguda. A angio-TC, devido à sua fácil acessibilidade e ao curto tempo de imagem, é uma excelente modalidade de imagem para identificar esse problema e identificar quando a intervenção é necessária quando há dificuldade ao eco.^986 , 1042 , 1043^

Na maioria dos centros, o cateterismo cardíaco é realizado no pré-operatório da cirurgia de Glenn. Foi realizada comparação de angio-TC e cateterismo antes do estágio paliativo 2, que revelou excelente correlação com achados cirúrgicos para ambas as modalidades, e não houve diferença no resultado cirúrgico até a alta hospitalar. O grupo de cateterismo teve maior dose de radiação e de contraste, necessitou de acesso vascular central e anestesia geral em todos os casos e teve uma taxa relativamente alta de eventos adversos.^1044^ Um estudo anterior randomizou pacientes pré-estágio 2 para RMC ou cateterismo e não encontrou diferença na cirurgia ou desfechos de médio prazo para pacientes, seguidos em uma mediana de 8 anos.^1045^ Alguns centros vêm sugerindo avaliação diagnóstica prévia aos estágios paliativos de forma não invasiva, reservando os cateterismos para procedimentos terapêuticos.^1046^

#### 4.1.5.3. Para os Estágios Glenn e Fontan

A TC cardiovascular tem demonstrado visualizar adequadamente todos os aspectos no pós-operatório das cirurgias de Glenn e de Fontan após paliação de ventrículo único. As alterações hemodinâmicas e as alterações anatômicas em pacientes após a paliação de ventrículo único promovem desafios únicos na opacificação ideal das artérias pulmonares e do circuito cavopulmonar que podem resultar em exames não diagnósticos ou interpretação errônea de imagens. É importante usar técnicas adequadas para otimizar a homogeneidade do contraste nas artérias pulmonares e reconhecer as armadilhas comuns na realização e interpretação de estudos angiográficos de TC pulmonares em cada estágio.^1047 , 1048^

A formação de trombo é relativamente comum, e eles podem ser visualizados por TC cardiovascular no conduto de Fontan, no ventrículo residual ou no coto pulmonar residual após ligadura da artéria. A embolia pulmonar também é identificada pela TC cardiovascular. Cuidados devem ser tomados, no entanto, para otimizar a técnica de injeção de contraste para opacificar de forma otimizada o circuito de Fontan e evitar um diagnóstico falso-positivo de embolia pulmonar.^986 , 1048^

Outra complicação usual nesse grupo de pacientes é a presença de estenose nas artérias pulmonares, que pode ocorrer por retração cicatricial de manipulações prévias, anastomose de *shunt* , após a retirada de bandagem seletiva dos ramos ou hipoplasia difusa de ramos após cirurgia de Norwood, por exemplo. Como já bem estabelecido pela literatura, a avaliação das artérias pulmonares é excelente pela angio-TC, assim como a avaliação de possíveis *stents* previamente implantandos nessa topografia que são mais bem visualizados pela TC do que pela RMC devido aos artefatos da malha metálica.^1033 , 1049^

A visualização de circulação colateral sistêmicopulmonar e/ou venovenosa também pode ser realizada na angio-TC, entretanto, não é possível quantificar o fluxo dessas colaterais e sua repercussão hemodinâmica como na RMC, mas a TC torna-se uma alternativa para avaliação nas situações em que não é possível o uso da RMC.^1033 , 1050 – 1053^ A quantificação da função ventricular por TC cardiovascular pode ser garantida em pacientes com implantes metálicos e contraindicações para RMC.^986 , 1048^

A angio-TC também fornece informações diagnósticas em situações clínicas mais instáveis e complexas, como nos pacientes em oxigenação por membrana extracorpórea (ECMO) ou com suporte de dispositivo de assistência ventricular, uma população em que a imagem convencional pode ser um desafio. Além da avaliação de lesões residuais, é possível avaliar posições da cânula, presença de trombos e possíveis sítios de infecção no mediastino.^986 , 1054^ A seguir ( Tabela 72 ), encontram-se os cenários clínicos relacionados à utilização da TC no contexto de coração univentricular.

**Tabela 72:** Coração univentricular

Indicação	Classe de recomendação	Nível de evidência
Avaliação pré-operatória de anatomia para correção^986^	IIa	C
Avaliação estágio I ( *shunt* S-P ou bandagem pulmonar)^1042 – 1044^	I	C
Pós-estágio 2 (Glenn): disfunção valvar ou ventricular, avaliação da anastomose da veia cava superior com o território pulmonar, circulação colateral^1045^	IIa	C
Pós-estágio 3 (Fontan): disfunção valvar ou ventricular, avaliação de trombos no circuito cavopulmonar total, circulação colateral^1046 , 1047^	IIa	C

#### 4.1.6. Miscelânea

As malformações cardíacas podem acompanhar as alterações de posicionamento dos órgãos do tórax e abdome. Heterotaxia e anormalidades de situs descrevem um arranjo anormal de órgãos viscerais na cavidade toracoabdominal ao longo do eixo esquerdo-direito normal do corpo. Esse arranjo anormal está associado a uma alta ocorrência de defeitos cardíacos e abdominais congênitos, incluindo veias pulmonares anômalas, alterações de conexões, anormalidades venosas sistêmicas, asplenia e outras alterações viscerais abdominais. Geralmente consistem em cardiopatias mais complexas que necessitam de ampla avaliação vascular em vários territórios.^1055^

A TC fornece extensos e valiosos dados cardiovasculares e extracardíacos nas síndromes heterotáxicas. Ela avalia de maneira acurada a anatomia torácica de parênquima pulmonar e via aérea e ainda elucida a disposição visceral abdominal.^1056 , 1057^

A TC, pela sua alta resolução espacial e reconstrução de imagem multiplanar, também permite uma visão abrangente de outras alterações espaciais, que incluem o cruzamento das vias de entrada com disposição súpero-inferior ventricular, ocasionada pela rotação do coração sobre o seu eixo longo, um arranjo chamado *criss-cross* . Permite avaliar as conexões atrioventriculares cruzadas e as anomalias intra e extracardíacas associadas, correlacionando as estruturas para uma melhor programação de abordagem cirúrgica.^1058^ A seguir ( Tabela 73 ), encontram-se cenários clínicos relacionados ao emprego da TC nas condições referidas neste tópico.

**Tabela 73:** Miscelânea

Indicação	Classe de recomendação	Nível de evidência
Avaliação de alterações de posicionamento no tórax e rotação nos eixos cardíacos^1058^	I	C
Anomalias de situs e síndromes heterotáxicas^1055 – 1057^	I	C

### 4.2. Ressonância Magnética Cardiovascular em Cardiopatias Congênitas

A utilização da RMC no suporte aos portadores de cardiopatias congênitas vem se consolidando como uma importante estratégia no diagnóstico dessas condições. Por se tratar de um exame com elevadas resoluções espacial e temporal, permite o detalhamento anatômico dessas patologias, bem como define com precisão o resultado cirúrgico das diferentes abordagens cirúrgicas (e eventuais complicações decorrentes) utilizadas nessas correções. Pelo fato de não utilizar radiação ionizante, apresenta um perfil adequado para aplicação na população pediátrica.

A RMC apresenta várias modalidades de avaliação em um único exame, permitindo explorar diferentes parâmetros cardiovasculares. As sequências de cinerressonância constituem-se o padrão-ouro para estimativa de função e volumes biventriculares, informações fundamentais no acompanhamento pré e pós-operatório de diversas cardiopatias congênitas. Avaliações de fluxos (pela técnica de " *phase-contrast* ") pela RM são ferramentas precisas na definição de lesões valvares e *shunts* intra ou extracardíacos. Sequências de angiografias (arteriais e venosas) permitem a adequada avaliação das circulações sistêmica e pulmonar, tanto em *status* pré e pós-operatório. Por fim, sequências de caracterização tecidual permitem a definição de estruturas que exijam diagnóstico diferencial por meio de informações da constituição histológica (por exemplo, massas tumorais e trombos).

Como exposto acima, existem inúmeros benefícios oferecidos pela utilização da RMC no diagnóstico das cardiopatias congênitas. Entretanto, por se tratar de exame de alta complexidade e disponibilidade limitada, o seu emprego deve estar restrito a cenários específicos, sobretudo nos quais as informações oferecidas pelo ecocardiograma sejam limitadas (por imagens subótimas) ou insuficientes. Além disso, a RMC demanda controle ventilatório durante a aquisição das imagens, o que é alcançado por apneia voluntária (habitualmente em pacientes com mais de 7 anos de idade) ou com anestesia e intubação orotraqueal, com apneias controladas por pausas na ventilação mecânica.

#### 4.2.1. Avaliação de *Shunts* Intra e Extracardíacos

#### 4.2.1.1. Comunicação Interatrial

Em geral, a avaliação de CIA é feita de forma conclusiva pela ecocardiografia, conduzindo a decisão clínica na faixa pediátrica.^975 , 1059^ O papel principal da RMC é como método complementar, principalmente para os adolescentes ou adultos em que o ecocadiograma não foi completamente elucidativo.^974 , 978^

Vale destacar que pode haver dificuldade de acesso ecocardiográfico especialmente na CIA do tipo seio venoso, assim como na pesquisa de defeitos associados, como a conexão anômala de veias pulmonares, com alguma frequência associada ao tipo seio venoso superior,^975 , 976 , 1060 – 1062^ sendo a RMC bem indicada na avaliação pré-operatória desses pacientes.^974^

A RMC permite também avaliar a repercussão hemodinâmica com o cálculo do Qp/Qs, o grau de sobrecarga e o desempenho ventricular.^975 , 1063^ Nos casos de avaliação pré-operatória ou pré-intervenção percutânea, a RMC é um método menos preciso na mensuração do defeito e suas bordas quando comparado ao ecocardiograma ou à TC.^979 , 981^ Pode ser usada para planejar a melhor estratégia de tratamento de CIAs do tipo *ostium secundum* .^974^

#### 4.2.1.2. Forame Oval Patente

Assim como a CIA, a avaliação de forame oval patente (FOP) por RMC é absoluta exceção; em quase todos os casos, a investigação por ecocardiograma e outras modalidades (como Dopplerfluxometria transcraniana) é conclusiva e definitiva.^978 , 1059^ A RMC ou TC podem ser usadas nos poucos casos em que há limitação na avaliação ecocardiográfica.^981^ É pesquisada a passagem de contraste intravenoso ou a presença de sinais de fluxo transeptal durante manobras de Valsalva.^1064 , 1065^

Após fechamento percutâneo, a RMC e a TC só são indicadas se houver suspeita de complicações, como infecção, má posição, embolização ou *shunt* persistente.^978^ Abaixo ( Tabela 74 ), encontram-se as principais indicações de utilização da RMC no contexto da CIA.

**Tabela 74:** Comunicação interatrial (CIA)

Indicação	Classe de recomendação	Nível de evidência
Avaliação pré-operatória para correção de CIA, anatomia septal, pesquisa de aneurismas do septo e decisão de opções terapêuticas^1062^	IIa	C
Diagnóstico e avaliação pré-operatória e diagnóstico de correção de CIA seio venoso e/ou defeito do septo atrioventricular parcial e seio coronário^976 , 1066^	I	B
Seguimento pós-operatório ou pós-intervenção percutânea de CIA, pacientes com *shunt* residual significativo, disfunção valvar ou ventricular, arritmias e/ou hipertensão pulmonar^976 , 978^	IIa	C
Seguimento pós-intervenção percutânea de CIA, suspeita de complicações, embolização ou *shunt* persistente^978^	IIb	C
Avaliação inicial de forame oval patente^1059 , 1064 , 1065^	III	C
Estimativa de relação entre fluxos pulmonar e sistêmico (Qp/Qs) para auxílio na tomada de decisão cirúrgica^1060 – 1062^	IIa	C

#### 4.2.1.3. Conexão Anômala de Veias Pulmonares Parcial e Total

A anatomia da conexão anômala de veias pulmonares (CAVP) é bem caracterizada tão bem na TC quanto na RMC, tanto na sua forma total como na parcial. Permite a avaliação detalhada tanto na determinação da confluência venosa e local de drenagem quanto na identificação de possíveis áreas de obstrução venosa, além do estudo da anatomia cardíaca com reconhecimento de lesões associadas,^971 , 1067 – 1070^ sendo bem indicada na avaliação pré-operatória desses pacientes.^974 , 976^

A RMC tem a vantagem sobre a TC de permitir uma melhor avaliação da repercussão hemodinâmica das lesões, o grau de sobrecarga cavitária e o desempenho ventricular, podendo ser indicada no seguimento de conexão anômala de veias pulmonares parcial assintomático com envolvimento de mais de uma veia, CAVP total, especialmente quando houver mudança na apresentação clínica.^974^ No pós-operatório, a RM pode ter algum papel no seguimento de pacientes com maior risco de evolução desfavorável (como obstrução venosa ou disfunção) ou CAVP total.^974^ A avaliação da CAVP por meio da RMC tem suas indicações definidas na Tabela 75 .

**Tabela 75:** Conexão anômala de veias pulmonares (CAVP)

Indicação	Classe de recomendação	Nível de evidência
Avaliação pré-operatória de correção de CAVP^976 , 1066 , 1071^	I	B
Complicações atribuíveis à CAVP durante seguimento pós-tratamento^971 , 1068^	I	C
Seguimento pós-operatório de CAVP, pacientes assintomáticos, com pouca ou nenhuma sequela^1063^	IIa	C

#### 4.2.1.4. Comunicação Interventricular (CIV)

Os casos de CIV também são muito bem avaliados pelo ecocardiograma, permitindo uma adequada decisão clínica; a RMC sendo reservada, via de regra, àqueles casos em que o ecocardiograma não conseguiu realizar uma avaliação completa, geralmente em adolescentes e adultos, em especial como parte da avaliação pré-operatória.^974^ Pode haver maior dificuldade na avaliação de defeitos musculares apicais pelo ecocardiograma. Destacam-se também os casos de dupla via de saída do ventrículo direito (DVSVD); nesses casos, a contribuição da RMC (ou da TC) é maior, permitindo uma avaliação mais precisa da posição, dimensão e localização do defeito, em especial naqueles do tipo supracristal,^1005 , 1072 – 1074^ informações fundamentais para a programação da técnica cirúrgica que será empregada na correção.

Assim como nos demais defeitos acima descritos, a RMC consegue quantificar a repercussão hemodinâmica, com o cálculo do Qp/Qs, o grau de sobrecarga e o desempenho ventricular, além de defeitos associados.^975^ Tanto no seguimento de CIVs não abordadas quanto naqueles indivíduos tratados, a RM só tem algum papel na avaliação daqueles pacientes que apresentem uma evolução clínica desfavorável.^974^ A Tabela 76 traz o papel da RMC na avaliação da CIV.

**Tabela 76:** Comunicação interventricular (CIV)

Indicação	Classe de recomendação	Nível de evidência
Avaliação pré-operatória de correção de CIV para definir localização, anatomia e repercussão funcional^976^	IIa	C
Seguimento pós-operatório ou pós-intervenção percutânea de CIV, pacientes com *shunt* residual significativo, disfunção valvar ou ventricular, arritmias e/ou hipertensão pulmonar^974 , 975^	IIa	C
Estimativa de relação entre fluxos pulmonar e sistêmico (Qp/Qs) para auxílio na tomada de decisão cirúrgica^1060 , 1061^	IIa	C

#### 4.2.1.5. Defeito de Septo Atrioventricular

Aqui também o ecocardiograma é a modalidade principal de investigação em praticamente todos os casos, fornecendo uma avaliação completa e definitiva. A RMC é reservada somente para aqueles casos em que há a necessidade de investigar lesões associadas ou quantificar de forma mais precisa as repercussões do defeito de septo atrioventricular (DSAV)^975^ bem como o seu grau de desbalanceamento ventricular.

Pode ter algum papel na avaliação pré-operatória ou na avaliação de pacientes em seguimento (com ou sem tratamento), com evolução desfavorável. Uma avaliação de rotina pós-tratamento só está indicada naqueles pacientes de maior risco (sintomas congestivos, *shunt* residual etc.).^974^ A Tabela 77 (a seguir) apresenta as indicações da RMC na avaliação dos DSAV.

**Tabela 77:** Defeito do septo atrioventricular (DSAV)

Indicação	Classe de recomendação	Nível de evidência
Avaliação pré-operatória de correção de DSAV^976^	IIa	C
Seguimento pós-operatório de DSAV, pacientes com *shunt* residual significativo, disfunção valvar ou ventricular, obstrução da via de saída do ventrículo esquerdo, arritmias e/ou hipertensão pulmonar^1063 , 1067^	IIa	C
Seguimento pós-operatório de DSAV, pacientes com sinais de falência cardíaca^974^	IIa	C

#### 4.2.1.6. Persistência do Canal Arterial

A persistência do canal arterial (PCA) também é uma condição adequadamente avaliada somente com o ecocardiograma em quase todos os pacientes, sendo a RMC e TC reservadas somente para casos duvidosos ou inconclusivos pelo ecocardiograma, como avaliação pré-tratamento.^974^

Nos casos de PCA de dimensões maiores e/ou com morfologias complexas, esses métodos têm superioridade na avaliação anatômica, permitindo mensurações e informações que podem ajudar no planejamento, principalmente nos casos de abordagem percutânea.

Uma parcela dos pacientes tratados pode evoluir com obstruções no território pulmonar ou aórtico, sendo bem avaliados por RM.^974^ De forma semelhante aos cenários anteriores, a RM pode contribuir avaliando repercussões da lesão,^975^ em especial no seguimento (sem ou com tratamento) de pacientes com evolução desfavorável.^974^ A seguir ( Tabela 78 ), encontram-se os cenários clínicos relacionados à utilização da RMC na avaliação da PCA.

**Tabela 78:** Persistência do canal arterial (PCA)

Indicação	Classe de recomendação	Nível de evidência
Avaliação pré-operatória de correção de PCA^974^	IIb	C
Avaliação pós-tratamento com suspeita de complicação em território aórtico ou pulmonar^975^	I	C

#### 4.2.2. Lesões Congênitas Valvares

As cardiopatias congênitas podem estar associadas a anormalidades valvares, sendo o ecocardiograma o método de primeira escolha para avaliar a morfologia e função das valvas. A RMC é usada principalmente para avaliar o impacto fisiológico da regurgitação valvar medindo o volume de regurgitação, a fração regurgitante e o tamanho e função ventriculares. Essas informações desempenham um papel importante na decisão do momento das intervenções terapêuticas, principalmente intervenções cirúrgicas e/ou percutâneas. Nos casos de estenose valvar, a RMC pode ser usada para definir o tamanho do orifício valvar, mas pode subestimar a velocidade de pico e o gradiente de pressão estimado.

#### 4.2.2.1. Valva Tricúspide/Anomalia de Ebstein

Já é sabido que a RMC é considerada padrão-ouro para quantificação dos volumes e função do VD, que são muitas vezes desafiadores para quantificar com precisão pelo ecocardiograma. Avaliações seriadas de volume e função do VD pela RM podem ser úteis para determinar a evolução da doença e deterioração do VD. A quantificação da regurgitação tricúspide é desafiadora especialmente nos casos de anomalia de Ebstein, em que o(s) jato(s) regurgitante(s) pode(m) ser múltiplo(s) e ter direções incomuns. Além disso, a direção do jato pode mudar em diferentes fases da sístole com o movimento anular da valva. Tudo isso pode levar a imprecisões e falta de reprodutibilidade entre os exames.^988^

Neijenhuis et al.^1075^ forneceram uma revisão retrospectiva de uma única instituição de pacientes com Ebstein que foram submetidos a reparo através da cirurgia do cone. Seus objetivos principais eram avaliar a competência a longo prazo da valva tricúspide e da função biventricular por meio de imagens de RMC. O objetivo secundário foi avaliar o remodelamento reverso biventricular após o reparo do cone. No pré-operatório, apenas o volume do VD funcional (VDf) foi incluído, ignorando o volume da porção atrializada (VDa) e, assim, diminuindo significativamente os volumes do VD medidos. Possivelmente, se tivessem medido o VD anatômico (VDf + VDa) tanto no pré quanto no pós-operatório, teria ocorrido uma diminuição mais significativa desses volumes após o reparo. Por esse motivo, alguns autores recomendam que os volumes do VD na RMC incluam o VD anatômico completo no pré-operatório.^988^ Citado por outros autores, o mesmo estudo é referido ainda com valores limitados pelo número pequeno de pacientes que tiveram imagens de RMC de acompanhamento.^1076^ Beroukhim et al.^1077^ demonstraram significativa redução dos volumes do VD usando a RMC no seguimento tardio de pacientes com a cirurgia do cone. A correção levou a redução do volume regurgitante da tricúspide e do volume sistólico do VD, aumento do volume sistólico do VE, melhora da distensão do septo basal do VE e melhor sincronia do VE. Os volumes do VD relatados nos resultados incluíram a porção atrializada do VD por ser considerado melhor para demonstrar as alterações de volume relacionadas à redução da carga de volume, visto que a cirurgia do cone reposiciona o anel funcional.

#### 4.2.2.2. Valva Mitral

Apesar de contarmos com inúmeros estudos demonstrando o valor da RMC para avaliação e quantificação de regurgitação mitral, o ecocardiograma é o método de escolha por todos os benefícios já citados, como o fácil acesso, custo e praticidade. No caso de crianças pequenas com insuficiência valvar de origem congênita, o ecocardiograma é o responsável pelo diagnóstico e seguimento desses pacientes, devendo a RMC ficar reservada para casos com resultados conflitantes, podendo auxiliar na avaliação da gravidade da regurgitação, quando a avaliação pelo ecocardiograma é considerada insatisfatória ou quando há uma discrepância entre a gravidade da insuficiência mitral e os achados clínicos. Há uma escassez de estudos comparativos, e a maioria mostrou uma modesta concordância nos valores qualitativos ou quantitativos. Isso é esperado tendo em vista os vários fatores que afetam a avaliação da regurgitação por cada modalidade. A reprodutibilidade da quantificação tem sido consistentemente maior com RMC.^875^

Cawley et al.^1078^ compararam prospectivamente ecocardiograma e RMC na avaliação da insuficiência mitral, demonstrando que a RMC tem menor variabilidade intraobservador e interobservador para volume regurgitante, sugerindo que pode ser superior para medidas seriadas.

As diretrizes da Sociedade Europeia e do AHA/ACC ao mesmo tempo enfatizam a importância de avaliar os efeitos hemodinâmicos da regurgitação mitral no VE e AE. Entretanto, as diretrizes têm recomendações limitadas sobre como realizar uma avaliação abrangente por RMC de forma padronizada.^1079^

#### 4.2.2.3. Estenose Mitral

A estenose da valva mitral raramente ocorre como uma doença congênita isolada. Apresenta-se mais comumente como uma lesão adquirida ou em associação com lesões obstrutivas sequenciais do lado esquerdo, como na síndrome de Shone e na síndrome de hipoplasia do coração esquerdo ou ainda em outras doenças cardíacas congênitas.

A maioria dos defeitos mitrais congênitos complexos, incluindo valva mitral de duplo orifício, anel supravalvar mitral, arcada mitral e valva mitral em paraquedas, está tipicamente associada à estenose da válvula mitral. Em crianças, estimar a estenose valvar mitral inclui pelo menos a avaliação do gradiente de pressão transvalvar, da área do orifício e do diâmetro anular. As diretrizes para adultos não podem simplesmente serem transcritas para as crianças. Qualquer avaliação também deve abordar a presença de defeitos associados, pois eles podem influenciar na abordagem terapêutica. A princípio, os dados obtidos pelo ecocardiograma são satisfatórios, podendo-se lançar mão de ferramentas adicionais como o ecocardiograma tridimensional. O papel de uma abordagem integrada de múltiplos métodos de imagem como a RM e a TC deve ser discutido e reservado para casos mais complexos nos quais a análise dos defeitos associados promovendo obstruções sequenciais no lado esquerdo é essencial para a definição do grau de obstrução.^1080^

#### 4.2.2.4. Valva Pulmonar

A análise de fluxos pela RMC através do mapeamento de velocidades é útil para a estimativa da gravidade da obstrução ou regurgitação pulmonar que ocorre em vários cenários de malformações congênitas ou após intervenção cirúrgica ou percutânea.

A RMC é atualmente o melhor método para quantificar a regurgitação pulmonar e avaliar em série o remodelamento e função do VD em pacientes com insuficiência pulmonar significativa. O método direto através da técnica de phase contrast obtida acima do plano da valva é o método de escolha, uma vez que permite a medição direta do refluxo pulmonar total e da fração de regurgitação.^875^

O ecocardiograma e o cateterismo cardíaco direito são os padrões-ouro para o diagnóstico de estenose da valva pulmonar e avaliação da gravidade da doença e da capacidade de resposta ao tratamento.^1081^ A RM também pode fornecer informações valiosas sobre as características morfológicas e a mobilidade da valva, a dilatação pós-estenótica, o grau de estenose e a localização de uma estenose supra ou subvalvar. Diferentes tipos de estenose podem ser distinguidos na imagem de RMC. O jato de estenose pulmonar valvar pode ser direcionado para a artéria pulmonar esquerda, resultando em dilatação e aumento do fluxo sanguíneo para o pulmão esquerdo. A valva pulmonar bicúspide estenótica está comumente associada à dilatação da artéria pulmonar pós-estenótica e à formação de aneurisma resultante do fluxo pós-estenótico, que podem ser observados em imagens de *4D flow* .^990^ O grau de estenose também pode ser avaliado usando mapeamento de velocidade, mas gradientes determinados por ecocardiograma são normalmente preferidos.

#### 4.2.2.5. Valva Aórtica

Embora a RMC possa fornecer informações importantes sobre a morfologia da valva aórtica, seu potencial completo ainda precisa ser determinado, e mais estudos de desfechos clínicos são necessários antes que os dados da RMC possam ser integrados ao manejo de pacientes com lesões valvares aórticas significativas. A cine-RM dos folhetos valvares pode ser usada para avaliar a morfologia da valva na presença de janelas ecocardiográficas inadequadas. A RMC pode fornecer uma avaliação detalhada da estrutura valvar e da anatomia da raiz da aorta, o que auxilia na identificação da causa da regurgitação. Além disso, as dimensões de toda a aorta torácica podem ser medidas. A vantagem da RMC na insuficiência aórtica é a quantificação da regurgitação e dos volumes e função do VE, principalmente para medição seriada.

A valva aórtica bicúspide é a malformação mais frequente da valva aórtica e uma das malformações cardiovasculares congênitas mais comuns, com incidência estimada em 1 a 2% da população. A regurgitação e a estenose aórtica são comuns na valvuloplastia aórtica por balão (VAB). Pode ocorrer isoladamente ou estar associada a outras malformações congênitas, como coarctação da aorta, estenose sub ou supravalvar aórtica, CIV, persistência do canal arterial e aneurisma do seio de Valsalva. Pacientes com valvas aórticas bicúspides que apresentam dilatação documentada dos seios de Valsalva ou aorta ascendente devem ter avaliação seriada das dimensões, pois a aortopatia pode progredir com o tempo. As indicações da utilização da RMC na avaliação do acometimento valvar congênito são demonstradas na Tabela 79 .

**Tabela 79:** Acometimento valvar congênito

Indicação	Classe de recomendação	Nível de evidência
Avaliação complementar de lesões valvares regurgitantes^875 , 1079 , 1082^	I	B
Avaliação complementar de lesões valvares estenóticas^974 , 976^	IIa	B
Avaliação pré-operatória para correção de anomalia de Ebstein (anatomia da valva tricúspide, ventrículo direito e árvore pulmonar)^988 , 1083^	IIa	C
Pós-correção de Ebstein: disfunção valvar ou ventricular^1075 , 1077^	IIa	C

#### 4.2.3. Anomalias Conotruncais

#### 4.2.3.1. Tetralogia de Fallot

A grande contribuição da RM se dá na avaliação pós-operatória em decorrência da presença defeitos residuais, sendo uma das importantes complicações a insuficiência pulmonar. A principal vantagem do método é a quantificação precisa do tamanho e função biventricular, sendo o padrão-ouro para avaliação de volume e fluxo na tetralogia de Fallot, avaliando as consequências hemodinâmicas da insuficiência pulmonar residual no VD após a correção total. A insuficiência pulmonar pode ser deletéria a longo prazo, ocasionando dilatação e disfunção ventricular, arritmias e eventos adversos tardios.^1084^ A regurgitação tricúspide pode ocorrer como consequência da dilatação ventricular direita e levar a aumento volumétrico cavitário adicional. Disfunção tanto do VD quanto do VE podem ocorrer devido a cianose de longa data antes do reparo e/ou proteção miocárdica inadequada durante a correção, interação interventricular/dissincronia eletromecânica ou anormalidades da artéria coronária.^1085 , 1086^

O momento mais adequado para reabordagem cirúrgica é de fundamental importância e ainda é um desafio, sendo necessário o acompanhamento do volume e da função ventricular sob o risco de se perder a possibilidade de recuperação funcional após a intervenção. As recomendações para reabordagem nos pacientes assintomáticos com insuficiência pulmonar significativa dependem dos volumes do VD, e, quando há uma dilatação progressiva do VD para volume sistólico final do VD indexado (VSFVDi) ≥ 80 mL/m^2^ e volume diastólico final do VD indexado (VDFVDi) ≥ 160 mL/m^2^, a reintervenção deve ser considerada bem como quando há disfunção ventricular associada, seja direita ou esquerda.^1087 – 1090^

Avalia-se ainda no pós-operatório de tetralogia de Fallot a presença de aneurismas e regiões acinéticas em via de saída do ventrículo direito e outras lesões residuais como estenoses ou *shunts* intracardíacos. A CIV residual pode ser por deiscência do remendo ou por fechamento incompleto durante a correção cirúrgica e pode acarretar sobrecarga volumétrica do VE. A estenose residual pode ocorrer no infundíbulo, na valva pulmonar, no tronco pulmonar ou nas artérias pulmonares. Hipertrofia ventricular direita e aumento pressórico nas cavidades direitas foram descritos como fatores de risco independentes de mau desempenho e evolução desfavorável mesmo com volumes menores de VD.^1091^

Dilatação aórtica e insuficiência valvar aórtica também podem ocorrer no seguimento desses pacientes como complicações no território aórtico, evoluindo raramente para dissecção aórtica.^1092^

Além disso, por meio do RT, se estabelece prognóstico, permitindo correlação entre quantidade de fibrose com disfunção ventricular, intolerância ao exercício e desencadeamento de eventos arrítmicos.

Mais recentemente, os mapas paramétricos têm tido um papel emergente, e o aumento do VEC calculado com medidas de T1 pré e pós-gadolínio também foi associado a sobrecarga de volume do VD e arritmias, sugerindo que medidas de fibrose difusa podem ajudar a estratificar o risco do paciente. As indicações da utilização da RMC no contexto de tetralogia de Fallot encontram-se a seguir ( Tabela 80 ).

**Tabela 80:** Tetralogia de Fallot

Indicação	Classe de recomendação	Nível de evidência
Avaliação pré-operatória nos casos de anatomia desfavorável ou se houver limitação pelo ecocardiograma	I	C
Avaliação no pós-operatório (volumetria e função do ventrículo direito) antes da substituição planejada da valva pulmonar (percutânea ou cirúrgica)^1088 , 1093 – 1097^	I	B
Avaliação com obstrução da via de saída do ventrículo direito, estenose do território pulmonar, arritmias ou presença de um conduto ventrículo direito-artéria pulmonar^1091^	I	C

#### 4.2.3.2. Dupla Via de Saída do Ventrículo Direito

A DVSVD envolve um amplo espectro de arranjos anatômicos na dependência da relação da CIV, resultando em apresentações fisiológicas variáveis.^1005^ A intervenção cirúrgica é, portanto, determinada pela fisiologia pré-operatória predominante e inclui reparos para qualquer um desses substratos anatômicos.

A RM é capaz de fornecer uma avaliação tridimensional da anatomia intracardíaca, esclarecendo a relação vascular com o defeito septal interventricular, e de avaliar de forma abrangente a função valvar e ventricular, bem como os volumes cavitários. A avaliação do aparato da valva atrioventricular, a sua relação com o defeito interventricular e a presença ou não de *straddling* são pontos importantes no diagnóstico. O reparo biventricular requer ventrículos de volumes adequados.^1008^ A criação de tunelizações em topografia intraventricular pode comprometer o volume da cavidade ventricular direita, já que parte dela é incorporada à via de saída do VE. Portanto, é importante avaliar o tamanho da cavidade do VD no pré-operatório e estimar o volume do VD remanescente após a abordagem cirúrgica pretendida.

A avaliação pré-operatória desses pacientes vai ajudar na decisão terapêutica de se o substrato anatômico é passível de reparo biventricular. A presença de infundíbulo em topografia subaórtica ou subpulmonar deve ser avaliada e ajuda na tomada de decisão pré-procedimento.

As complicações potenciais após o reparo biventricular estão na dependência da cirurgia indicada para cada grupo de fenótipo fisiológico da dupla via de saída. Podem incluir dilatação e/ou disfunção ventricular, obstrução das tunelizações das via de saída esquerda ou do VD e lesão residual de *shunt* intracavitário.^1098^ Na Tabela 81 , encontram-se as indicações de utilização da RMC na avaliação de DVSVD.

**Tabela 81:** Dupla via de saída de ventrículo direito

Indicação	Classe de recomendação	Nível de evidência
Avaliação pré-operatória de anatomia vascular e da relação da comunicação interventricular^1005 , 1008^	I	C
Avaliação de função e volumes ventriculares^1067^	I	C
Avaliação de obstrução da via de saída do ventrículo direito ou esquerdo, estenose de território pulmonar, arritmias ou presença de um conduto ventrículo direito-artéria pulmonar^1098^	IIa	C

#### 4.2.3.3. Tronco Arterioso Comum

A avaliação no pré-operatório é realizada na grande maioria das vezes pelo ecocardiograma. Em algumas situações, pode haver dúvidas em relação a emergência das artérias pulmonares a partir do tronco arterial comum ou uma melhor avaliação da valva truncal pode se fazer necessária.^1099^ A raiz da conexão ventrículo-arterial geralmente se encontra dilatada, e a valva truncal, que é quase sempre anormal, apresenta diferentes formas de anormalidades e deformidades. Pode ser displásica com várias cúspides associada a dificuldade de coaptação, gerando insuficiência na maioria das vezes e mais raramente apresentando limitação de sua abertura.^1100^

O arco aórtico consiste em outro ponto de avaliação, em que a lateralidade e a integridade do arco devem ser estabelecidas. Pode haver associação com interrupção do arco em alguns casos, mais comumente entre as artérias carótida comum esquerda e subclávia. Nesse cenário, a distância do segmento interrompido bem como o calibre e possíveis pontos de estenose no canal arterial que supre a aorta distal devem ser quantificados.^1101^

A correção desses pacientes consiste na reconstrução da via de saída do VD, criando uma continuidade com o território pulmonar. Isso pode incluir a colocação de um conduto ventrículo-artérias pulmonares, e este está propenso a desenvolver estenose ou regurgitação local. A RM pode fornecer uma avaliação anatômica e funcional precisas, avaliando não só a lesão residual desses condutos, mas também a árvore pulmonar distal e o grau de repercussão no VD, quantificando massa e volumes cavitários.^1102^ A avaliação ventricular direita se faz necessária, já que o VD pode estar submetido a insuficiência ou estenose residual na via de saída reconstruída, devendo haver um monitoramento para auxiliar na decisão de procedimentos terapêuticos adicionais.

Repercussões no VE também podem estar presentes por disfunção da antiga valva truncal, ocorrendo mais frequentemente insuficiência valvar. Nos casos em que a correção do arco foi realizada, podemos ter algum grau de obstrução nesta topografia no pós-operatório, outra causa de sobrecarga pressórica a longo prazo. A seguir ( Tabela 82 ), encontram-se as indicações de utilização da RMC na avaliação do tronco arterioso comum.

**Tabela 82:** Tronco arterioso comum

Indicação	Classe de recomendação	Nível de evidência
Avaliação pré-operatória para avaliação de anatomia para correção^1099 , 1100^	IIa	C
Avaliação no pós-operatório: comunicação interventricular residual conhecida, presença de estenose em conduto ventrículo direito-artéria pulmonar ou território pulmonar^1103^	IIa	C
Avaliação no pós-operatório: estenose ou regurgitação da valva truncal	IIa	C

#### 4.2.3.4. Transposição das Grandes Artérias

No cenário da avaliação pré-operatória da transposição das grandes artérias, o ecocardiograma consiste no melhor método e, na grande maioria dos casos, consegue definir os dados anatômicos e funcionais necessários para a correção cirúrgica. Nos casos de uma morfologia mais desafiadora, essas informações podem ser complementadas, dando-se preferência pela TC se a dúvida diagnóstica é no território coronariano e podendo se utilizar tanto a RM como a TC na suspeita de anomalias associadas, por exemplo, na avaliação de obstruções subpulmonares e no território aórtico.

Após a cirurgia de Jatene, correção cirúrgica de escolha na atualidade, as principais complicações ocorrem no plano de sutura vascular em que ocorreu a troca arterial e no reimplante coronariano.^1019^ Os territórios aórtico e pulmonar podem ser estudados com medidas de fluxo local e por angio-RM.^1104^

Complicações no território pulmonar podem ocorrer em topografia supravalvar pulmonar ou artérias pulmonares relacionadas com a manobra de Lecompte, unilateralmente ou bilateralmente por distorção vascular local. Dilatação da raiz da neoaorta com insuficiência valvar pode ocorrer e trazer complicações a longo prazo. A insuficiência aórtica pode ser avaliada pela RM de maneira quantitativa, estimando a fração regurgitante local e repercussão nas cavidades esquerdas.

Avaliação de complicações da abordagem da porção proximal das coronárias pode ser realizada pela pesquisa de isquemia e infarto com as técnicas de perfusão e viabilidade miocárdica. Disfunção ventricular esquerda e arritmias são raras, mas podem ocorrer e estar relacionadas a problemas no reimplante coronariano ou à proteção miocárdica durante o procedimento cirúrgico de troca arterial.^1105^ Avaliação da anatomia vascular do reimplante coronariano é feita de maneira mais acurada pela TC, pela sua melhor resolução espacial.

Após a correção da transposição das grandes artérias em nível atrial, em que apenas a fisiologia da circulação é corrigida, o estudo da perviedade das tunelizações se faz necessário, bem como o desempenho contrátil do VD sistêmico e a insuficiência da valva tricúspide. Podemos evoluir com disfunção e falência do VD a longo prazo, bem como com regurgitação tricúspide progressiva (valva atrioventricular sistêmica). A RM fornece dados confiáveis e robustos em relação a avaliação da função sistólica sistêmica do VD. Além disso, pode avaliar melhor o grau de hipertrofia miocárdica e fibrose miocárdica associada. *Shunts* residuais pela tunelização em nível atrial também podem ser quantificados, mas pequenas comunicações podem ser limitadas ao método, podendo ser melhor visualizadas ao ecocardiograma. A Tabela 83 apresenta as indicações de utilização da RMC na avaliação de transposição das grandes artérias.

**Tabela 83:** Transposição das grandes artérias

Indicação	Classe de recomendação	Nível de evidência
Avaliação pré-operatória de anatomia para correção^1018^	IIa	C
Pós-cirurgia de Jatene: avaliação coronariana^1019 , 1021^	IIb	C
Pós-cirurgia de Jatene: disfunção valvar ou ventricular moderada, obstrução da via de saída do ventrículo direito ou esquerdo, estenose de território pulmonar ou arritmias^1104^	I	C
Pós-cirurgia de Jatene: raiz neoaórtica dilatada ou regurgitação neoaórtica^1022^	I	C
Pós-correção atrial (por exemplo, Senning): regurgitação atrioventricular sistêmica, disfunção sistêmica do ventrículo direito, obstrução da via de saída do ventrículo esquerdo ou arritmias	I	C
Pós-correção atrial (por exemplo, Senning): avaliação de tunelizações venosas^1106^	I	C

#### 4.2.3.5. Transposição Corrigida das Grandes Artérias

A transposição congenitamente corrigida das grandes artérias pode ocorrer isoladamente ou em combinação com outras anomalias cardíacas estruturais. A RM avalia os defeitos associados que podem incluir algum grau de estenose pulmonar, acometimento da valva tricúspide além da CIV. Fornece anatomia intracardíaca e dos grandes vasos e é indicada para quantificação de volumes ventriculares, função e massa miocárdica, especialmente nos casos em que a avaliação ecocardiográfica do VD for mais difícil e menos confiável. A valva tricúspide pode ser displásica e ter graus variáveis de acolamento ( *Ebstein-like* ) ou apresentar-se insuficiente com gravidade variável no cenário de dilatação do VD sistêmico.

A avaliação funcional do VD anatômico conectado à aorta e que enfrenta pressão sistêmica deve ser avaliada e, em algum momento de sua evolução, irá desenvolver insuficiência sistólica ou regurgitação tricúspide.^1107^

Pode haver um reparo fisiológico, em que as lesões estruturais são reparadas, mas o VD é mantido como sistêmico. Isso inclui a abordagem das associações mais comuns, como o fechamento de CIV, reparo da tricúspide e correção da estenose pulmonar. Nesse cenário, além da procura de lesões residuais como *shunts* intracavitários, quantificação da insuficiência tricúspide e de estenoses residuais subpulmonares, deve-se também ter enfoque na cavidade sistêmica do VD. É essencial monitorar volumes, massa e função de maneira bem curada. A RM é o padrão-ouro para avaliação de VD sistêmico, sendo recomendada para o manejo adequado do paciente.^1108 , 1109^ Alterações de sua conformação geométrica, com dilatação e hipertrofia exuberante além da pesquisa de fibrose miocárdica em que geralmente se constata RT pontual e focal no ponto de inserção inferior do septo interventricular sem significado clinico conhecido e a presença de fibrose densa focal em outras porções do VD, ainda permanencem controversas.^1110^ Mais estudos para pesquisa de fibrose intersticial através de mapas paramétricos são necessários.

Em alguns casos, o reparo anatômico pode ser realizado como estratégia (reparo em nível atrial e arterial – *Double switch* ), em que os retornos venosos e os fluxos de saída são organizados para que o ventrículo morfológico esquerdo torne-se o ventrículo sistêmico e o VD se torne o ventrículo subpulmonar.^1025 , 1111^ A avaliação nesse contexto pela TC acaba sendo uma melhor alternativa por avaliar melhor o reimplante coronariano. Abaixo ( Tabela 84 ), encontram-se as indicações da utilização da RMC no contexto da transposição corrigida das grandes artérias.

**Tabela 84:** Transposição corrigida das grandes artérias

Indicação	Classe de recomendação	Nível de evidência
Avaliação pré-operatória de anatomia para correção^1112^	IIa	C
Pós-operatório: insuficiência da valva atrioventricular sistêmica, disfunção sistêmica do ventrículo direito^1107 – 1109^	I	C
Pós-operatório: disfunção do conduto ventrículo esquerdo-artéria pulmonar^1111^	IIa	C

#### 4.2.4. Anomalias da Aorta Torácica

#### 4.2.4.1. Coarctação de Aorta

Nos casos de crianças pequenas em avaliação pré-operatória, com janela acústica adequada, geralmente a RMC não é necessária, em que o ecocardiograma permite uma avaliação completa e precisa. A RMC (e a TC) é reservada somente para aqueles casos inconclusivos pelo ecocardiograma (pacientes maiores com janela inadequada) ou quando há necessidade de uma avaliação mais panorâmica da aorta, como nos casos de suspeita de anormalidades associadas no arco aórtico.^2 – 4^ Embora geralmente não necessária, quando realizada, a RMC é superior ao ecocardiograma em diversos aspectos, permitindo uma avaliação mais integral e precisa da anatomia no local da coarctação de aorta (CoAo), sua localização e extensão, anatomia do arco e relações espaciais com seus ramos, com medidas mais precisas das estruturas regionais, presença e extensão de colaterais, possibilitando o planejamento cirúrgico adequado mesmo em casos complexos.^419 , 1040 , 1113^ O acompanhamento com RM ou TC pode ser feito para avaliar complicações a longo prazo, como formação de aneurisma, fratura ou migração de *stents* . Abaixo ( Tabela 85 ), encontram-se os principais cenários relacionados ao emprego da RMC na avaliação de coarctação de aorta.

**Tabela 85:** Coarctação da aorta (CoAo)

Indicação	Classe de recomendação	Nível de evidência
Avaliação pré-operatória de correção de CoAo^1114 , 1115^	I	B
CoAo em seguimento pós-tratamento, com mudança clínica atribuível ao defeito^1116 , 1117^	I	C
Seguimento no primeiro ano pós-tratamento percutâneo (6-12 meses), paciente assintomático com nenhuma ou discreta sequela^419 , 1040 , 1113 , 1118^	IIa	B
Seguimento após o primeiro ano pós-tratamento, paciente assintomático com nenhuma ou discreta sequela, intervalo de 1 a 2 anos^419 , 1040 , 1113 , 1118^	IIa	B
Seguimento pós-tratamento, paciente assintomático, para avaliar aneurisma de arco e/ou *stent* (reestenose, fratura ou *endoleak* )^419 , 1040 , 1048 , 1113 , 1118^	IIa	B
Seguimento pós-tratamento, paciente com sintomas de falência cardíaca^419 , 1040 , 1113 , 1118^	IIa	B

#### 4.2.4.2. Outras Anomalias da Aorta

A aorta pode ser sede de outras doenças congênitas, algumas complexas e que demandam uma avaliação pré-operatória adequada. Temos como exemplos a hipoplasia do arco aórtico, a interrupção da aorta e a janela aortopulmonar.^1119^

A RMC é um método bem estabelecido na avaliação da aorta e de seus principais ramos, em casos complexos selecionados, permitindo uma avaliação adequada em todas essas condições. A TC permite uma avaliação comparável, mostrando superioridade nos casos de anéis vasculares, por apresentar uma melhor avaliação de estruturas aeradas como a traqueia.

O grande papel desses métodos é no acompanhamento pós-operatório, os cenários e as indicações bastante semelhantes aos da CoAo.^1119^ A Tabela 86 contém as principais indicações da RMC na avaliação das anomalias do arco aórtico.

**Tabela 86:** Anomalias do arco aórtico

Indicação	Classe de recomendação	Nível de evidência
Avaliação de anel vascular^1118 , 1119^	IIa	C
Avaliação de interrupção de arco aórtico^1119^	I	C
Avaliação de janela aortopulmonar^1119^	I	C

#### 4.2.5. Coração Univentricular

O conceito de coração univentricular compreende corações não passíveis de reparo biventricular e incluem um grupo variado de malformações cardíacas. Nesse cenário, a correção cirúrgica é univentricular com cirurgias paliativas com 2 a 3 estágios, culminando na cirurgia de Fontan.^1020^

O ecocardiograma é a principal ferramenta de imagem durante a avaliação inicial. Em alguns casos, a RMC pode ser usada para avaliar casos duvidosos de reparo uni ou biventricular no contexto de ventrículo dito *borderline* . Segundo estudo de Grosse-Wortmann et al.^1118^ avaliando o VE *borderline* pelo ecocardiograma e pela RMC, o ecocardiograma subestimou sistematicamente o volume do VE e não se correlacionou com a RM. A geometria do VE em pacientes com VE limítrofe é quase certamente o motivo da imprecisão dessa técnica quando comparada com a RM.^419 , 1113 , 1118^

Embora o ecocardiograma forneça imagens diagnósticas excelentes em muitos pacientes com fisiologia de ventrículo único, a RMC tem utilidade adicional especialmente na avaliação da anatomia extracardíaca com reconstruções multiplanares e de volume tridimensional em pacientes com janelas acústicas subótimas ou outras limitações técnicas. A RM se tornou o padrão-ouro para avaliação dos volumes ventriculares, FE e massa, uma vez que a técnica se baseia na aquisição de imagens contíguas e paralelas, adquiridas com alta resolução temporal e sem a necessidade de fazer suposições sobre a forma ventricular. Isso é particularmente verdadeiro no cenário de malformações cardíacas congênitas complexas, em que a forma ventricular é geralmente atípica e altamente variável, de modo que a avaliação ecocardiográfica dos volumes e massa ventricular é menos precisa e reprodutível do que a RM.^1120 – 1122^ A função ventricular e a geometria são parâmetros cruciais na evolução clínica dos pacientes convertidos para a circulação de Fontan.^5 , 6^ A RMC permite avaliar a funcionalidade da valva atrioventricular do ventrículo sistêmico e fornece meios para analisar os padrões de fluxo em qualquer componente do circuito de Fontan.^1048^

Estudos demonstraram que a RMC em conjunto com o ecocardiograma pode substituir o cateterismo diagnóstico de rotina em pacientes selecionados antes do procedimento de Glenn bidirecional. Na ausência de evidência de hipertensão pulmonar, a medição de rotina de resistência vascular pulmonar não é necessária antes do Glenn bidirecional. Brown et al.^1123^, comparando valores de cateterismo e RMC, encontraram valores de resistência pulmonar no grupo de cateterismo dentro dos limites aceitáveis, e os pacientes no grupo RMC não tiveram uma evolução diferente após a cirurgia de Glenn bidirecional mesmo na ausência dos dados de resistências e pressões.^1114 , 1117 , 1123^

Após o procedimento de Fontan, os pacientes permanecem sob risco de inúmeras complicações, além de disfunção ventricular e valvar, obstrução e/ou estenose do conduto de Fontan, estenose das artérias pulmonares, CoAo, formação de colaterais venosas e sistêmico-pulmonares, além de formação de trombos intracardíacos. A RMC tem um papel fundamental na vigilância dessas complicações, visto que muitas vezes o ecocardiograma tem janela limitada para esses achados.^419^

A RM possui também papel na determinação do prognóstico desses pacientes, visto que parâmetros derivados da RMC, como volume ventricular e fibrose miocárdica, mostraram estar associados a resultados adversos.^1115 , 1124^

A RM oferece a possibilidade de caracterização tecidual com mapas T1, T2 e T2* do miocárdio para avaliação de evidências de edema miocárdico, cicatrizes, fibrose difusa e deposição de ferro.^1125^ Muitas dessas novas técnicas quantitativas em RM foram utilizadas apenas em estudos menores em pediatria e ainda estão em pesquisa para fornecer dados baseados em evidências para comprovar sua utilidade.^1048^

Conexões vasculares anormais entre as artérias sistêmicas e o leito vascular pulmonar, os vasos colaterais sistêmico-pulmonares (CSP) se manifestam em pacientes com uma variedade de doenças cardíacas congênitas e adquiridas e ocorre comumente em pacientes com ventrículo único após conexão cavopulmonar superior, com significado clínico ainda obscuro. Já está bem estabelecido na literatura que é possível quantificar o fluxo da circulação CSP após conexões cavopulmonares bidirecionais e cirurgia de Fontan, usando as técnicas de análises de fluxos pela RM.^1053 , 1126 – 1130^

O aumento da pressão venosa central pode induzir a recanalização de vasos embriologicamente pré-formados e obliterados e levar ao desenvolvimento de colaterais venosas sistêmicas após procedimentos de Glenn ou Fontan e podem levar à dessaturação sistêmica e redução da função ventricular, resultando em desempenho diário prejudicado em pacientes com doença cardíaca univentricular.^1131 – 1134^

As complicações linfáticas em pacientes com ventrículo único repercutem sinais de falência no sistema de Fontan e incluem bronquite plástica, enteropatia perdedora de proteínas e quilotorax e são uma fonte de morbimortalidade significativa com opções terapêuticas historicamente limitadas. Novas técnicas de imagem linfática, como a linfangiografia por RM ponderada em T2 sem contraste e a linfangiografia dinâmica por RM com contraste, são capazes de avaliação anatômica do sistema linfático nesta população de pacientes e vêm promissoramente orientando o desenvolvimento de novas técnicas de intervenção linfática levando ao progresso do tratamento de patologias linfáticas.^1134 – 1142^ A Tabela 87 (abaixo) traz os principais cenários clínicos relacionados à utilização da RMC no manejo do coração univentricular.

**Tabela 87:** Coração univentricular

Indicação	Classe de recomendação	Nível de evidência
Avaliação pré-operatória de anatomia para correção^974^	IIa	C
Avaliação estágio I ( *shunt* S-P ou bandagem pulmonar)^974 , 976^	I	C
Pós-estágio 2 (Glenn): disfunção valvar ou ventricular avaliação da anastomose da veia cava superior com o território pulmonar, circulação colateral^419 , 1116^	I	C
Pós-estágio 3 (Fontan): disfunção valvar ou ventricular, avaliação de trombos no circuito cavopulmonar total, circulação colateral^1048 , 1117^	I	C

#### 4.2.6. Miscelânea

A RM pode ter um papel relevante na avaliação de cardiopatias mais complexas associadas a alterações espaciais e geométricas importantes cuja análise pode ser limitada ao ecocardiograma. Como consiste em um método que independe de janela acústica, a realização dessa modalidade de imagem não invasiva pode ser considerada para uma avaliação mais aprofundada, sendo possível estabelecer o diagnóstico não só das associações intracardíacas, mas também das alterações viscerais torácicas e abdominais associadas.^1143^

As heterotaxias consistem em síndromes congênitas que podem ser classificadas em isomerismo direito ou esquerdo na dependência da morfologia dos apêndices atriais do coração, em que também observamos alterações da disposição visceral dentro do tórax e abdome. Um amplo espectro de anormalidades pode ser encontrado com fígado em topografia mediana, poliespelenia ou asplenia, interrupção de VCI, alterações na drenagem venosa, além das malformações intracardícas associadas.^1144 , 1145^

Outras situações em que a ressonância pode vir em auxílio diagnóstico consistem em alteração espacial do coração ou mesmo de partes dele, como ventrículos em disposição superoinferior com rotação de suas vias de entrada ( *criss-cross heart* ).^1146 , 1147^ Em relação a este último, esclarece não só alteração de disposição da via de entrada perpendicular, mas também a dinâmica de abertura valvar e se há a presença de *straddling* ou *overriding* .^1148^

A seguir ( Tabela 88 ), encontram-se cenários clínicos relacionados ao emprego da RMC nas condições referidas neste tópico.

**Tabela 88:** Miscelânea

Indicação	Classe de recomendação	Nível de evidência
Avaliação de alterações de posicionamento no tórax e rotação nos eixos cardíacos^1149^	I	C
Anomalias de *situs* e síndromes heterotáxicas^1055 , 1145^	I	C

## Lista de abreviaturas

AA: – Aneurisma de aorta
AAE: – Apêndice atrial esquerdo
AC: – Anomalias de coronárias
ACC/AHA: – American College of Cardiology/American Heart Association
ACCURACY: – *Assessment by Coronary Computed Tomographic Angiography of Individuals Undergoing Invasive Coronary Angiography*
ACRIN-PA: – *Angiography for Safe Discharge of Patients with Possible Acute Coronary Syndromes*
AE: – Átrio esquerdo
Angio-TC: – Angiotomografia computadorizada coronariana
ATTR: – Amiloidose transtirretina
AUC: – Área sob a curva
AUC ROC: – Área sob a curva característica de operação do receptor
AVC: – Acidente vascular cerebral
AVCi: – Acidente vascular cerebral isquêmico
BEM: – Biópsia endomiocárdica
CAC: – Calcificação arterial coronariana
CAC-DRS: – *Coronary Artery Calcium Data and Reporting System*
CAD Consortium: – *Coronary Artery Disease Consortium*
CAPTURE: – *Randomised Placebo-Controlled Trial of Abciximab Before and During Coronary Intervention in Refractory Unstable Angina*
CARDIA: – *Coronary Risk Development in Young Adults*
CATSCAN: – *Coronary Assessment by Computed Tomographic Scanning and Catheter Angiography*
CBR: – Colégio Brasileiro de Radiologia
CC: – Cardiomiopatia chagásica
CDI: – Cardiodesfibrilador implantável
CIA: – Comunicação interatrial
CIV: – Comunicação interventricular
CMD: – Cardiomiopatia dilatada
CMH: – Cardiomiopatia hipertrófica
CMPP: – Cardiomiopatia periparto
CoAo: – Coarctação de aorta
CONFIRM: – *Coronary CT Angiography Evaluation for Clinical Outcomes: An International Multicenter Registry*
CONSERVE: – *Coronary Computed Tomographic Angiography for Selective Cardiac Catheterization*
CORE320: – *Coronary Artery Evaluation using 320-row Multidetector Computed Tomography Angiography and Myocardial Perfusion Study*
CORE64: – *Coronary Artery Evaluation Using 64-Row Multidetector Computed Tomography Angiography*
CT-STAT: – *Coronary Computed Tomographic Angiography for Systematic Triage of Acute Chest Pain Patients to Treatment*
CAVP: – Conexão anômala das veias pulmonares
D/CAVD: – Displasia/cardiomiopatia arritmogênica do ventrículo direito
DA: – Dissecção aórtica
DAC: – Doença arterial coronariana
DAOP: – Doença arterial obstrutiva periférica
DC: – Doença de Chagas
DHS: – *Dallas Heart Study*
DILA: – Dilatação fluxo-mediada da artéria braquial
DISCHARGE: – *Diagnostic Imaging Strategies for Patients with Stable Chest Pain and Intermediate Risk of Coronary Artery Disease*
DISCOVER-FLOW: – *Diagnosis of Ischemia-Causing Stenoses Obtained Via Noninvasive Fractional Flow Reserve (FFR) Assessment* (diagnóstico de estenoses causadoras de isquemia obtidas por avaliação não invasiva da reserva de fluxo fracionado [FFR])
DM: – Diabetes melito
DMB: – Distrofia muscular de Becker
DMD: – Distrofia muscular de Duchenne
DSVA: – Defeito do septo atrioventricular
DVSVD: – Dupla via de saída de ventrículo direito
EBCT: – *Electron beam computed tomography*
EC: – Escore de cálcio coronariano
ECG: – Eletrocardiograma
ECMO: – *Extracorporeal membrane oxygenation*
EMF: – Endomiocardiofibrose
ERF: – Escore de risco de Framingham
ETE: – Ecocardiografia transesofágica
ETT: – Ecocardiografia transtorácica
FA: – Fibrilação atrial
FASTTRACK CABG: – *Safety and Feasibility Evaluation of Planning and Execution of Surgical Revascularization Solely Based on Coronary CTA and FFRCT in Patients with Complex Coronary Artery Disease*
FFR: – *Fractional flow reserve* (reserva de fluxo fracionada)
FHS: – *Framingham Heart Study*
FOP: – Forame oval patente
FOURIER: – *Further Cardiac Outcomes Research with PCSK9 Inhibition in Subjects with Elevated Risk*
FR: – Fatores de risco (para doença arterial coronariana)
HIM: – Hematoma intramural
HNR: – *Heinz Nixdorf Recall Study*
HP: – Hipertensão pulmonar
HR: – *Hazard ratio*
IAM: – Infarto agudo do miocárdio
IC: – Insuficiência cardíaca
IC95%: – Intervalo de confiança de 95%
IMT: – *Intimal Media Thickness* (Espessura médio-intimal)
ISCHEMIA: – *International Study of Comparative Health Effectiveness with Medical and Invasive Approaches*
ITB: – Índice tornozelo-braquial
JUPITER: – *Justification for the Use of Statins in Prevention: an Intervention Trial Evaluating Rosuvastatin* (Justificativa para o uso de estatinas na prevenção: um estudo de intervenção avaliando rosuvastatina)
MACE: – *Major adverse cardiovascular events* (eventos cardiovasculares adversos maiores)
MESA: – *Multi-Ethnic Study of Atherosclerosis*
MINOCA: – *Myocardial Infarct and Non Obstructive Coronary Arteries* (infarto do miocárdio com artérias coronárias sem obstrução)
MR-INFORM: – *Magnetic Resonance Imaging for Myocardial Perfusion Assessment in Coronary Artery Disease Trial*
NHLBI: – *National Heart, Lung and Blood Institute*
NIMISCAD: – *Non-Invasive Multicenter Italian Study for Coronary Artery Disease*
NNT: – Número necessário para tratar
NRI: – *Net reclassification index*
OP: – *Ostium primum*
ORBITA: – *Objective Randomised Blinded Investigation with optimal medical Therapy of Angioplasty in stable angina*
OS: – *Ostium secundum*
PCA: – Persistência do canal arterial
PCR: – Proteína C-reativa
PLATFORM: – *A Prospective, Randomized, Multicenter Study to Compare Effectiveness and Safety of a 6-Month Versus a 12-Month Duration of Dual Antiplatelet Therapy in Subjects Undergoing Percutaneous Coronary Intervention With Zotarolimus-Eluting Coronary Stents*
PMTC: – Perfusão miocárdica por tomografia computadorizada
PREDICT: – *Prevent Disease Global Cardiovascular Risk Assessment*
PROMISE: – *Prospective Multicenter Imaging Study for Evaluation of Chest Pain*
Qp/Qs: – Relação entre fluxos pulmonar e sistêmico
RM: – Ressonância magnética
RMC: – Ressonância magnética cardíaca
ROBINSCA: – *Risk or Benefit in Screening for Cardiovascular Disease*
ROC: – *Receiver operating characteristic curve*
ROMICAT II: – *Rule Out Myocardial Ischemia/Infarction Using Computer Assisted Tomography*
RTM: – Realce tardio miocárdico
SAA: – Síndrome aórtica aguda
SARS-CoV-2: – *Severe acute respiratory syndrome coronavirus 2* (síndrome respiratória aguda grave do coronavírus 2)
SBC: – Sociedade Brasileira de Cardiologia
SCA: – Síndrome coronariana aguda
SCCT: – *Society of Cardiovascular Computed Tomography*
SCORE: – *Systemic Coronary Risk Evaluation*
SCOT-HEART: – *Scottish COmputed Tomography of the HEART*
SIS: – *Segment involvement score*
SPECT: – *Single photon emission computed tomography*
SSFP: – *Steady state free precession* (precessão livre em estado de equilíbrio)
SYNTAX III REVOLUTION: – *A Randomized Study to Evaluate the Feasibility of Heart-Team Clinical Decision Making Regarding the Optimal (Surgical or Percutaneous Based) Revascularization Strategy in Patients With Complex Coronary Artery Disease, Based on Non-invasive Coronary CT Angiography (CTA) Imaging Utilising High-definition GE RevolutionTM Multi-slice CT and HeartFlow FFRCT Compared to the Current Standard of Care With Conventional Invasive Coronary Angiography (CA)*
TARGET-CTCA: – *Troponin in Acute Chest Pain to Risk Stratify and Guide EffecTive Use of Computed Tomography Coronary Angiography*
TAVI: – *Transcatheter aortic valve implantation* (implante transcaterer valvar aórtico)
TC: – Tomografia computadorizada
TCMD: – Tomografia computadorizada de múltiplos detectores
TEP: – Tromboembolismo pulmonar
TpNOCA: - *Troponin positive with Non Obstructive Coronaries Arteries* (troponina positiva em coronárias sem obstruções)
UP: – Úlcera penetrante
VCI: – Veia cava inferior
VCS: – Veia cava superior
VD: – Ventrículo direito
VE: – Ventrículo esquerdo
VEC: – Volume de espaço extracelular
VPN: – Valor preditivo negativo
VSVD: – Via de saída do ventrículo direito
VSVE: – Via de saída do ventrículo esquerdo
