# Supplementary material for: Diretriz de Tomografia Computadorizada e Ressonância Magnética Cardiovascular da Sociedade Brasileira de Cardiologia e do Colégio Brasileiro de Radiologia – 2024
Source: Arq Bras Cardiol. 2024 Oct 17;121(9):e20240608. [Article in Portuguese] doi: 10.36660/abc.20240608 (PMC12094294; doi:10.36660/abc.20240608)
Supplement: Translation

# Translation: Cardiovascular Computed Tomography and Magnetic Resonance Imaging Guideline of the Brazilian Society of Cardiology and the Brazilian College of Radiology – 2024

**DOI:** 10.36660/abc.20240608i

**Authors:** Tiago Augusto Magalhães, Adriano Camargo de Castro Carneiro, Valéria de Melo Moreira, Henrique Simão Trad, Marly Maria Uellendahl Lopes, Rodrigo Julio Cerci, Marcelo Souto Nacif, Paulo R. Schvartzman, Antônio Carlos Palandrini Chagas, Isabela Bispo Santos da Silva Costa, André Schmidt, Afonso Akio Shiozaki, Sérgio Tavares Montenegro, Leopoldo Soares Piegas, Marcelo Zapparoli, José Carlos Nicolau, Fabio Fernandes, Marcelo Souza Hadlich, Nabil Ghorayeb, Evandro Tinoco Mesquita, Luiz Flávio Galvão Gonçalves, Felix José Alvarez Ramires, Juliano de Lara Fernandes, Pedro Vellosa Schwartzmann, Salvador Rassi, Jorge Andion Torreão, José Carlos Pachón Mateos, Luiz Beck-da-Silva, Marly Conceição Silva, Gabriela Liberato, Gláucia Maria Moraes de Oliveira, Gilson Soares Feitosa, Hilka dos Santos Moraes de Carvalho, Brivaldo Markman, Ricardo Paulo de Sousa Rocha, Clerio Francisco de Azevedo, Flávio Taratsoutchi, Otavio Rizzi Coelho-Filho, Roberto Kalil, Ludhmila Abrahão Hajjar, Walther Yoshiharu Ishikawa, Cíntia Acosta Melo, Ieda Biscegli Jatene, Andrei Skromov de Albuquerque, Carolina de Medeiros Rimkus, Paulo Savoia Dias da Silva, Thiago Dieb Ristum Vieira, Fabio Biscegli Jatene, Guilherme Sant Anna Antunes de Azevedo, Raul D. Santos, Guilherme Urpia Monte, José Antonio Franchini Ramires, Marcio Sommer Bittencourt, Alvaro Avezum, Leonardo Sara da Silva, Alexandre Abizaid, Ilan Gottlieb, Dalton Bertolim Precoma, Gilberto Szarf, Antônio Carlos Sobral Sousa, Ibraim Masciarelli Francisco Pinto, Fábio de Morais Medeiros, Bruno Caramelli, José Rodrigues Parga, Tiago Senra Garcia dos Santos, Carlos Eduardo Elias dos Prazeres, Marcelo Antonio Cartaxo Queiroga Lopes, Luiz Francisco Rodrigues de Avila, Mauricio Ibrahim Scanavacca, Luis Henrique Wolff Gowdak, Silvio Henrique Barberato, Cesar Higa Nomura, Carlos Eduardo Rochitte

**Affiliations:** 1 Curitiba PR Brazil Complexo Hospital de Clínicas da Universidade Federal do Paraná (CHC-UFPR), Curitiba, PR – Brazil; 2 São Paulo SP Brazil Hospital do Coração (HCOR), São Paulo, SP – Brazil; 3 São Paulo SP Brazil Hospital Sírio Libanês, SP, São Paulo, SP – Brazil; 4 São Paulo SP Brazil Hospital Alemão Oswaldo Cruz, São Paulo, SP – Brazil; 5 São Paulo SP Brazil Instituto do Coração (Incor) do Hospital das Clínicas da Faculdade de Medicina da Universidade de São Paulo (HCFMUSP), São Paulo SP – Brazil; 6 Ribeirão Preto SP Brazil Lotus Radiologia Ltda, Ribeirão Preto, SP – Brazil; 7 São Paulo SP Brazil Universidade Federal de São Paulo (UNIFESP), São Paulo, SP – Brazil; 8 São Paulo SP Brazil DASA – Diagnósticos da América S/A, São Paulo, SP – Brazil; 9 Curitiba PR Brazil Quanta Diagnóstico por Imagem, Curitiba, PR – Brazil; 10 Niterói RJ Brazil Universidade Federal Fluminense, Niterói, RJ – Brazil; 11 Niterói RJ Brazil Hospital Universitário Antonio Pedro, Niterói, RJ – Brazil; 12 Porto Alegre RS Brazil Hospital Moinhos de Vento, Porto Alegre, RS – Brazil; 13 Santo André SP Brazil Faculdade de Medicina do ABC, Santo André, SP – Brazil; 14 São Paulo SP Brazil Instituto do Câncer do Estado de São Paulo, São Paulo, SP – Brazil; 15 Ribeirão Preto SP Brazil Universidade de São Paulo (USP), Ribeirão Preto, SP – Brazil; 16 Maringá PR Brazil ND Núcleo Diagnóstico, Maringá, PR – Brazil; 17 Maringá PR Brazil Ômega Diagnóstico, Maringá, PR – Brazil; 18 Maringá PR Brazil Hospital Paraná, Maringá, PR – Brazil; 19 Recife PE Brazil PROCAPE – Universidade de Pernambuco, Recife, PE – Brazil; 20 Curitiba PR Brazil DAPI, Curitiba, PR – Brazil; 21 Rio de Janeiro RJ Brazil Fleury Medicina e Saúde, Rio de Janeiro, RJ – Brazil; 22 Rio de Janeiro RJ Brazil Rede D'Or RJ, Rio de Janeiro, RJ – Brazil; 23 Rio de Janeiro RJ Brazil Unimed, Rio de Janeiro, RJ – Brazil; 24 Rio de Janeiro RJ Brazil Instituto Nacional de Cardiologia (INC), Rio de Janeiro, RJ – Brazil; 25 São Paulo SP Brazil Instituto Dante Pazzanese de Cardiologia, São Paulo, SP – Brazil; 26 São Paulo SP Brazil Inspirali Educação, São Paulo, SP – Brazil; 27 São Paulo SP Brazil Anhanguera Educacional, São Paulo, SP – Brazil; 28 Rio de Janeiro RJ Brazil Universidade Federal Fluminense (UFF), Rio de Janeiro, RJ – Brazil; 29 Aracaju SE Brazil Hospital São Lucas, Rede D'Or SE, Aracaju, SE – Brazil; 30 Aracaju SE Brazil Hospital Universitário da Universidade Federal de Sergipe, Aracaju, SE – Brazil; 31 Aracaju SE Brazil Clínica Climedi, Aracaju, SE – Brazil; 32 Campinas SP Brazil Radiologia Clinica de Campinas, Campinas, SP – Brazil; 33 Ribeirão Preto SP Brazil Hospital Unimed Ribeirão Preto, Ribeirão Preto, SP – Brazil; 34 Ribeirão Preto SP Brazil Centro Avançado de Pesquisa, Ensino e Diagnóstico (CAPED), Ribeirão Preto, SP – Brazil; 35 Goiânia GO Brazil Universidade Federal de Goiás, Goiânia, GO – Brazil; 36 Salvador BA Brazil Santa Casa da Bahia, Salvador, BA – Brazil; 37 Porto Alegre RS Brazil Universidade Federal do Rio Grande do Sul (UFRGS), Porto Alegre, RS – Brazil; 38 Belo Horizonte MG Brazil Axial Medicina Diagnóstica, Belo Horizonte, MG – Brazil; 39 Rio de Janeiro RJ Brazil Universidade Federal do Rio de Janeiro, Rio de Janeiro, RJ – Brazil; 40 Salvador BA Brazil Escola Bahiana de Medicina e Saúde Pública, Salvador, BA – Brazil; 41 Recife PE Brazil Hospital das Clínicas de Pernambuco da Universidade Federal de Pernambuco (UFPE), Recife, PE – Brazil; 42 Recife PE Brazil Real Hospital Português de Pernambuco, Recife, PE – Brazil; 43 Fortaleza CE Brazil Hospital Monteklinikum, Fortaleza, CE – Brazil; 44 Fortaleza CE Brazil Hospital Oto Kora, Fortaleza, CE – Brazil; 45 Minneapolis USA Minneapolis Heart Institute, Abbott Northwestern Hospital Allina Health, Minneapolis – USA; 46 Campinas SP Brazil Universidade Estadual de Campinas (UNICAMP), Campinas, SP – Brazil; 47 São Paulo SP Brazil Hospital Beneficência Portuguesa de São Paulo, São Paulo, SP – Brazil; 48 São Paulo SP Brazil Hospital Infantil Sabará, São Paulo, SP – Brazil; 49 São Paulo SP Brazil Hospital das Clínicas da Faculdade de Medicina da Universidade de São Paulo (HCFMUSP), São Paulo SP – Brazil; 50 São Paulo SP Brazil Instituto D'Or de Pesquisa e Ensino (IDOR), São Paulo SP – Brazil; 51 Iowa City USA University of Iowa Hospitals and Clinics, Iowa City – USA; 52 Blumenau SC Brazil ECOMAX, Blumenau, SC – Brazil; 53 Blumenau SC Brazil Hospital Unimed Blumenau, Blumenau, SC – Brazil; 54 Blumenau SC Brazil Hospital São José de Jaraguá do Sul, Blumenau, SC – Brazil; 55 Blumenau SC Brazil Cliniimagem Criciúma, Blumenau, SC – Brazil; 56 Brasília DF Brazil Instituto de Cardiologia e Transplantes do Distrito Federal, Brasília, DF – Brazil; 57 Pittsburgh USA University of Pittsburgh, Pittsburgh – USA; 58 Goiânia GO Brazil CDI Premium – Clínica diagnóstica, Goiânia, GO – Brazil; 59 Rio de Janeiro RJ Brazil Fonte Imagem Medicina Diagnostica, Rio de Janeiro, RJ – Brazil; 60 Curitiba PR Brazil Sociedade Hospitalar Angelina Caron, Curitiba, PR – Brazil; 61 Aracaju SE Brazil Universidade Federal de Sergipe, Aracaju, SE – Brazil; 62 Aracaju SE Brazil Hospital São Lucas, Aracaju, SE – Brazil; 63 Aracaju SE Brazil Rede D'Or de Aracaju, Aracaju, SE – Brazil; 64 Brasília DF Brazsil Laboratório Sabin Centro de Imagem VEJA, Brasília, DF – Brazsil; 65 João Pessoa PB Brazil Hospital Alberto Urquiza Wanderley, João Pessoa, PB – Brazil; 66 Curitiba PR Brazil Cardioeco, Centro de Diagnóstico Cardiovascular, Curitiba, PR – Brazil

**Table:** 

Cardiovascular Computed Tomography and Magnetic Resonance Imaging Guideline of the Brazilian Society of Cardiology and the Brazilian College of Radiology – 2024	
The report below lists declarations of interest as reported to the SBC by the experts during the period of the development of these statement, 2021-2024.	
Expert	Type of relationship with industry
Adriano Camargo de Castro Carneiro	Nothing to be declared
Afonso Akio Shiozaki	Nothing to be declared
Alexandre Abizaid	Nothing to be declared
Alvaro Avezum	Other relationships Funding of continuing medical education activities, including travel, accommodation and registration in conferences and courses, from the brazilian or international pharmaceutical, orthosis, prosthesis, equipment and implants industry: - EMS: SBC Spirituality Symposia - DEMCA.
André Schmidt	Financial declaration B - Research funding under your direct/personal responsibility (directed to the department or institution) from the Brazilian or international pharmaceutical, orthosis, prosthesis, equipment and implants industry: - Janssen: Milvexiana; AMGEN: Olpasirana.
Andrei Skromov de Albuquerque	Nothing to be declared
Antonnio Carlos Palandri Chagas	Financial declaration A - Economically relevant payments of any kind made to (i) you, (ii) your spouse/partner or any other person living with you, (iii) any legal person in which any of these is either a direct or indirect controlling owner, business partner, shareholder or participant; any payments received for lectures, lessons, training instruction, compensation, fees paid for participation in advisory boards, investigative boards or other committees, etc. From the brazilian or international pharmaceutical, orthosis, prosthesis, equipment and implants industry: - Novo Nordisk: Semaglutide; De Vita Institute: Advisory Board. Other relationships Funding of continuing medical education activities, including travel, accommodation and registration in conferences and courses, from the brazilian or international pharmaceutical, orthosis, prosthesis, equipment and implants industry: - Novo Nordisk: Semaglutide.
Antônio Carlos Sobral Sousa	Financial declaration A - Economically relevant payments of any kind made to (i) you, (ii) your spouse/partner or any other person living with you, (iii) any legal person in which any of these is either a direct or indirect controlling owner, business partner, shareholder or participant; any payments received for lectures, lessons, training instruction, compensation, fees paid for participation in advisory boards, investigative boards or other committees, etc. From the brazilian or international pharmaceutical, orthosis, prosthesis, equipment and implants industry: - Viatris: Inspra. Other relationships Funding of continuing medical education activities, including travel, accommodation and registration in conferences and courses, from the brazilian or international pharmaceutical, orthosis, prosthesis, equipment and implants industry: - Pfizer: Sybrava.
Brivaldo Markman Filho	Nothing to be declared
Bruno Caramelli	Nothing to be declared
Carlos Eduardo Elias dos Prazeres	Nothing to be declared
Carlos Eduardo Rochitte	Nothing to be declared
Carolina de Medeiros Rimkus	Financial declaration A - Economically relevant payments of any kind made to (i) you, (ii) your spouse/partner or any other person living with you, (iii) any legal person in which any of these is either a direct or indirect controlling owner, business partner, shareholder or participant; any payments received for lectures, lessons, training instruction, compensation, fees paid for participation in advisory boards, investigative boards or other committees, etc. From the brazilian or international pharmaceutical, orthosis, prosthesis, equipment and implants industry: - Guerbet: artificial intelligence; Biogen: immunomodulators; Roche: immunomodulators.
Cesar Higa Nomura	Nothing to be declared
Cíntia Acosta Melo	Nothing to be declared
Clerio Francisco de Azevedo Filho	Nothing to be declared
Dalton Bertolim Precoma	Financial declaration A - Economically relevant payments of any kind made to (i) you, (ii) your spouse/partner or any other person living with you, (iii) any legal person in which any of these is either a direct or indirect controlling owner, business partner, shareholder or participant; any payments received for lectures, lessons, training instruction, compensation, fees paid for participation in advisory boards, investigative boards or other committees, etc. From the brazilian or international pharmaceutical, orthosis, prosthesis, equipment and implants industry: - Novo Nordisk: Ozempic; Astrazeneca: Forxiga; Novartis: Entresto; Daiichi Sankyo: Lixiana; Lilly: Tirzetapibe. B - Research funding under your direct/personal responsibility (directed to the department or institution) from the Brazilian or international pharmaceutical, orthosis, prosthesis, equipment and implants industry: - Novo Nordisk: heart failure, obesity; Lilly: heart failure, obesity; Astrazeneca: renal failure, hypertension, heart failure; MSD: dyslipidemia; Bayer: heart failure; Janssen-BMS: anticoagulation and prevention of cardioembolism. Other relationships Funding of continuing medical education activities, including travel, accommodation and registration in conferences and courses, from the brazilian or international pharmaceutical, orthosis, prosthesis, equipment and implants industry: - AstraZeneca: heart failure; Lilly: heart failure and obesity. Any economically relevant equity interest in companies in the healthcare or education industry or in any companies competing with or supplying to SBC: - Healthcare.
Evandro Tinoco Mesquita	Financial declaration A - Economically relevant payments of any kind made to (i) you, (ii) your spouse/partner or any other person living with you, (iii) any legal person in which any of these is either a direct or indirect controlling owner, business partner, shareholder or participant; any payments received for lectures, lessons, training instruction, compensation, fees paid for participation in advisory boards, investigative boards or other committees, etc. From the brazilian or international pharmaceutical, orthosis, prosthesis, equipment and implants industry: - Ache: Astra educational material and lessons. Other relationships Funding of continuing medical education activities, including travel, accommodation and registration in conferences and courses, from the brazilian or international pharmaceutical, orthosis, prosthesis, equipment and implants industry: - Pfizer: amyloidosis.
Fabio Biscegli Jatene	Nothing to be declared
Fábio de Morais Medeiros	Nothing to be declared
Fabio Fernandes	Nothing to be declared
Felix José Alvarez Ramires	Financial declaration A - Economically relevant payments of any kind made to (i) you, (ii) your spouse/partner or any other person living with you, (iii) any legal person in which any of these is either a direct or indirect controlling owner, business partner, shareholder or participant; any payments received for lectures, lessons, training instruction, compensation, fees paid for participation in advisory boards, investigative boards or other committees, etc. From the brazilian or international pharmaceutical, orthosis, prosthesis, equipment and implants industry: - Novartis; Pfizer; AstraZeneca; Amgen.
Flávio Tarasoutchi	Other relationships Funding of continuing medical education activities, including travel, accommodation and registration in conferences and courses, from the brazilian or international pharmaceutical, orthosis, prosthesis, equipment and implants industry: - Aula da Edwards.
Gabriela Liberato	Nothing to be declared
Gilberto Szarf	Nothing to be declared
Gilson Soares Feitosa Filho	Nothing to be declared
Gláucia Maria Moraes de Oliveira	Nothing to be declared
Guilherme Sant Anna Antunes de Azevedo	Financial declaration A - Economically relevant payments of any kind made to (i) you, (ii) your spouse/partner or any other person living with you, (iii) any legal person in which any of these is either a direct or indirect controlling owner, business partner, shareholder or participant; any payments received for lectures, lessons, training instruction, compensation, fees paid for participation in advisory boards, investigative boards or other committees, etc. From the brazilian or international pharmaceutical, orthosis, prosthesis, equipment and implants industry: - Canon: computed tomography.
Guilherme Urpia Monte	Nothing to be declared
Henrique Simão Trad	Nothing to be declared
Hilka dos Santos Moraes de Carvalho	Nothing to be declared
Ibraim Masciarelli Francisco Pinto	Nothing to be declared
Ieda Biscegli Jatene	Nothing to be declared
Ilan Gottlieb	Nothing to be declared
Isabela Bispo Santos da Silva Costa	Nothing to be declared
Jorge Andion Torreão	Nothing to be declared
José Antonio Franchini Ramires	Nothing to be declared
José Carlos Nicolau	Financial declaration A - Economically relevant payments of any kind made to (i) you, (ii) your spouse/partner or any other person living with you, (iii) any legal person in which any of these is either a direct or indirect controlling owner, business partner, shareholder or participant; any payments received for lectures, lessons, training instruction, compensation, fees paid for participation in advisory boards, investigative boards or other committees, etc. From the brazilian or international pharmaceutical, orthosis, prosthesis, equipment and implants industry: - Amgen; AstraZeneca; Bayer; CSL Behring; Daiichi Sankyo; Dalcor; Esperion; Janssen; Novartis; Novo Nordisk; Sanofi; Vifor; Anthos; Libbs. Other relationships Funding of continuing medical education activities, including travel, accommodation and registration in conferences and courses, from the brazilian or international pharmaceutical, orthosis, prosthesis, equipment and implants industry: - Novo Nordisk.
José Carlos Pachón Mateos	Nothing to be declared
Josá Rodrigues Parga Filho	Nothing to be declared
Juliano de Lara Fernandes	Nothing to be declared
Leonardo Sara da Silva	Nothing to be declared
Leopoldo Soares Piegas	Nothing to be declared
Ludhmila Abrahão Hajjar	Nothing to be declared
Luis Beck-da-Silva	Financial declaration A - Economically relevant payments of any kind made to (i) you, (ii) your spouse/partner or any other person living with you, (iii) any legal person in which any of these is either a direct or indirect controlling owner, business partner, shareholder or participant; any payments received for lectures, lessons, training instruction, compensation, fees paid for participation in advisory boards, investigative boards or other committees, etc. From the brazilian or international pharmaceutical, orthosis, prosthesis, equipment and implants industry: - Viatris; Pfizer; AstraZeneca; NovoNordisk. B - Research funding under your direct/personal responsibility (directed to the department or institution) from the Brazilian or international pharmaceutical, orthosis, prosthesis, equipment and implants industry: - CSL Vifor. Other relationships Funding of continuing medical education activities, including travel, accommodation and registration in conferences and courses, from the brazilian or international pharmaceutical, orthosis, prosthesis, equipment and implants industry: - Novo Nordisk; Viatris.
Luis Henrique Wolff Gowdak	Financial declaration A - Economically relevant payments of any kind made to (i) you, (ii) your spouse/partner or any other person living with you, (iii) any legal person in which any of these is either a direct or indirect controlling owner, business partner, shareholder or participant; any payments received for lectures, lessons, training instruction, compensation, fees paid for participation in advisory boards, investigative boards or other committees, etc. From the brazilian or international pharmaceutical, orthosis, prosthesis, equipment and implants industry: - Servier: chronic coronary syndrome; Novartis: hypercholesterolemia. B - Research funding under your direct/personal responsibility (directed to the department or institution) from the Brazilian or international pharmaceutical, orthosis, prosthesis, equipment and implants industry: - Servier: chronic coronary syndrome. Other relationships Funding of continuing medical education activities, including travel, accommodation and registration in conferences and courses, from the brazilian or international pharmaceutical, orthosis, prosthesis, equipment and implants industry: - Servier: chronic coronary syndrome.
Luiz Flávio Galvão Gonçalves	Nothing to be declared
Luiz Francisco Rodrigues de Avila	Nothing to be declared
Marcello Zapparoli	Other relationships Any economically relevant equity interest in companies in the healthcare or education industry or in any companies competing with or supplying to SBC: - Partner in an imaging diagnostics company.
Marcelo Antonio Cartaxo Queiroga Lopes	Nothing to be declared
Marcelo Souto Nacif	Nothing to be declared
Marcelo Souza Hadlich	Nothing to be declared
Marcio Sommer Bittencourt	Financial declaration A - Economically relevant payments of any kind made to (i) you, (ii) your spouse/partner or any other person living with you, (iii) any legal person in which any of these is either a direct or indirect controlling owner, business partner, shareholder or participant; any payments received for lectures, lessons, training instruction, compensation, fees paid for participation in advisory boards, investigative boards or other committees, etc. From the brazilian or international pharmaceutical, orthosis, prosthesis, equipment and implants industry: - Cleerly Health; Elucid.
Marly Conceição Silva	Nothing to be declared
Marly Maria Uellendahl Lopes	Nothing to be declared
Mauricio Ibrahim Scanavacca	Financial declaration A - Economically relevant payments of any kind made to (i) you, (ii) your spouse/partner or any other person living with you, (iii) any legal person in which any of these is either a direct or indirect controlling owner, business partner, shareholder or participant; any payments received for lectures, lessons, training instruction, compensation, fees paid for participation in advisory boards, investigative boards or other committees, etc. From the brazilian or international pharmaceutical, orthosis, prosthesis, equipment and implants industry: - Daiichi Sankyo: anticoagulation. B - Research funding under your direct/personal responsibility (directed to the department or institution) from the Brazilian or international pharmaceutical, orthosis, prosthesis, equipment and implants industry: - J&J: ventricular tachycardia ablation. C - Personal research funding paid by the Brazilian or international pharmaceutical, orthosis, prosthesis, equipment and implants industry: - ABBOT: catheter ablation of vagal vessel cindame. Other relationships Funding of continuing medical education activities, including travel, accommodation and registration in conferences and courses, from the brazilian or international pharmaceutical, orthosis, prosthesis, equipment and implants industry: - J&J: Industry-sponsored symposium.
Nabil Ghorayeb	Nothing to be declared
Otavio Rizzi Coelho-Filho	Financial declaration A - Economically relevant payments of any kind made to (i) you, (ii) your spouse/partner or any other person living with you, (iii) any legal person in which any of these is either a direct or indirect controlling owner, business partner, shareholder or participant; any payments received for lectures, lessons, training instruction, compensation, fees paid for participation in advisory boards, investigative boards or other committees, etc. From the brazilian or international pharmaceutical, orthosis, prosthesis, equipment and implants industry: - Pfizer: amiloidose/Tafamides; AstraZeneca: insuficiência cardíaca/Forxiga; Bayer: Firialta; Norvartis: insuficiência cardíaca/dislipidemia; EMS: insuficiência cardíaca. Other relationships Funding of continuing medical education activities, including travel, accommodation and registration in conferences and courses, from the brazilian or international pharmaceutical, orthosis, prosthesis, equipment and implants industry: - Bayer; Pfizer; AstraZeneca.
Paulo R. Schvartzman	Nothing to be declared
Paulo Savoia Dias da Silva	Nothing to be declared
Pedro Vellosa Schwartzmann	Financial declaration B - Research funding under your direct/personal responsibility (directed to the department or institution) from the Brazilian or international pharmaceutical, orthosis, prosthesis, equipment and implants industry: - MSD; Novartis; AstraZeneca; Alnylam; BridgeBio; Ionis; Lilly. Other relationships Funding of continuing medical education activities, including travel, accommodation and registration in conferences and courses, from the brazilian or international pharmaceutical, orthosis, prosthesis, equipment and implants industry: - Pfizer; AstraZeneca; Novartis; Alnylam; NovoNordisk.
Raul D. Santos	Financial declaration A - Economically relevant payments of any kind made to (i) you, (ii) your spouse/partner or any other person living with you, (iii) any legal person in which any of these is either a direct or indirect controlling owner, business partner, shareholder or participant; any payments received for lectures, lessons, training instruction, compensation, fees paid for participation in advisory boards, investigative boards or other committees, etc. From the brazilian or international pharmaceutical, orthosis, prosthesis, equipment and implants industry: - Libbs: dyslipidemia; Novartis: dyslipidemia; Novo-Nordisk: diabetes, Sanofi: dyslipidemia; Eli-Lilly: diabetes. B - Research funding under your direct/personal responsibility (directed to the department or institution) from the Brazilian or international pharmaceutical, orthosis, prosthesis, equipment and implants industry: - Amgen: dyslipidemias; Novartis: dyslipidemias; Esperion: dyslipidemias; Sanofi: dyslipidemias; Ionis: dyslipidemias; Eli-Lilly: dyslipidemias; Kowa: dyslipidemias; Amrit: dyslipidemias. Other relationships Funding of continuing medical education activities, including travel, accommodation and registration in conferences and courses, from the brazilian or international pharmaceutical, orthosis, prosthesis, equipment and implants industry: - Sanofi.
Ricardo Paulo de Sousa Rocha	Nothing to be declared
Roberto Kalil Filho	Nothing to be declared
Rodrigo Julio Cerci	Nothing to be declared
Salvador Rassi	Nothing to be declared
Sérgio Tavares Montenegro	Other relationships Participation in government-related regulatory authorities or advocacy authorities in cardiology: - Member of the Technical Chamber of Cardiology of the Ministry of Health.
Silvio Henrique Barberato	Financial declaration A - Economically relevant payments of any kind made to (i) you, (ii) your spouse/partner or any other person living with you, (iii) any legal person in which any of these is either a direct or indirect controlling owner, business partner, shareholder or participant; any payments received for lectures, lessons, training instruction, compensation, fees paid for participation in advisory boards, investigative boards or other committees, etc. From the brazilian or international pharmaceutical, orthosis, prosthesis, equipment and implants industry: - Pfizer: amyloidosis; Bristol: Camzyos; Boston: left atrial appendage occlusion.
Thiago Dieb Ristum Vieira	Nothing to be declared
Tiago Augusto Magalhães	Financial declaration A - Economically relevant payments of any kind made to (i) you, (ii) your spouse/partner or any other person living with you, (iii) any legal person in which any of these is either a direct or indirect controlling owner, business partner, shareholder or participant; any payments received for lectures, lessons, training instruction, compensation, fees paid for participation in advisory boards, investigative boards or other committees, etc. From the brazilian or international pharmaceutical, orthosis, prosthesis, equipment and implants industry: - Conjuge - Novartis; Eli-Lilly; BMS; Knight; MSD; Daichii-Sankyo; AstraZeneca; Pint Pharma; GSK; Roche; Gilead; Adium; Pfizer: breast cancer.
Tiago Senra Garcia dos Santos	Nothing to be declared
Valéria de Melo Moreira	Nothing to be declared
Walther Yoshiharu Ishikawa	Nothing to be declared

Table of contents

**1. Introduction** 12

1.1. Definition of Recommendations and Evidence 12

1.2. Definitions of Clinical Risk Score and Pretest Probability 13

**1.2.1. Clinical Risk Score** 13

**1.2.2. Pretest Probability** 13

**2. Cardiovascular Computed Tomography** 13

2.1. Coronary Calcium Score 15

**2.1.1. Current Evidence on Cardiovascular Risk Stratification by CAC Scoring** 15

**2.1.2. Role of CAC Score in Risk Stratification Defined by Traditional Clinical Scores** 20

**2.1.3. Use of CAC Score to Support Pharmacotherapy Decision-making** 21

2.2. CCTA in Suspected Stable Angina without Known CAD 21

**2.2.1. As a First-choice Option in the Assessment of Nonacute Chest Pain** 21

**2.2.2. In Low-risk Patients with a Positive Functional Test** 23

2.3. In the Investigation of Ischemic Etiology of HF 23

2.4. CCTA in Suspected Stable Angina without Known CAD 23

**2.4.1. Patients with Stents** 23

**2.4.2. Revascularized Patients** 24

2.5. Follow-up of Patients with CAD Receiving Medical Therapy 24

2.6. CCTA in the Evaluation of Other CAD-related Scenarios 26

2.7. Coronary Artery Anomalies 26

2.8. CCTA in Suspected Acute Chest Pain 27

**2.8.1. Triple Rule-out** 28

2.9. CCTA in Preoperative Assessment 28

2.10. Valvular Heart Disease Assessment by CCTA 29

2.11. Preoperative Assessment in Percutaneous Aortic Valve Implantation (TAVI/ViV) 29

2.12. Percutaneous Planning of Other Structural Changes 30

2.13. Assessment of Cardiac Veins, Left Atrium, and Pulmonary Veins (Including Planning for Atrial Fibrillation Ablation/Atrial Appendage Occlusion) 31

2.14. Functional Assessment by CT 32

**2.14.1. CT Myocardial Perfusion Imaging** 32

**2.14.2. CT-derived Fractional Flow Reserve (FFRCT)** 33

2.15. CT in the Assessment of Non-ischemic Cardiomyopathy 34

**2.15.1. CT in the Assessment of Ventricular Function** 34

**2.15.2. Myocardial Tissue Characterization by Delayed Enhancement CT in Non-ischemic Cardiomyopathy** 35

**2.15.3. Myocardial Extracellular Volume Measurement by CT** 35

2.16. CT in the Assessment of Pericardial Diseases 35

2.17. CT in the Assessment of Cardiac Masses/Thrombi 37

2.18. Vascular Diseases 38

**2.18.1. Aorta** 38

**2.18.2. Extracranial Carotid Arteries** 40

**2.18.3. Renal Arteries** 40

**2.18.4. Peripheral Vascular Disease** 40

**2.18.5. Pulmonary Arteries** 41

**2.18.6. Visceral Arteries** 41

**3. Cardiovascular Magnetic Resonance** 41

3.1. Use of Multiparametric Mapping in the Differential Diagnosis of Cardiomyopathies 44

3.2. Investigation of CAD Using MRI – Myocardial Ischemia 46

3.3. Investigation of DAC Using MRI – Myocardial Viability 47

3.4. Coronary Magnetic Resonance Angiography 51

3.5. Differential Diagnosis of Troponin-positive Nonobstructive Coronary Arteries (TP-NOCA/MINOCA) 51

3.6. Stress Cardiomyopathy (Takotsubo) 52

3.7. Myocarditis/Inflammatory Cardiomyopathy 53

3.8. Athlete's Heart 53

3.9. Hypertrophic Cardiomyopathy 54

3.10. Endomyocardial Fibrosis 55

3.11. Cardiac Amyloidosis 56

3.12. Cardiac hemosiderosis 57

3.13. Other Storage Diseases 57

3.14. Chagas Heart Disease 58

3.15. Arrhythmogenic Right Ventricular Dysplasia/Cardiomyopathy 59

3.16. Sarcoidosis 60

3.17. Left Ventricular Noncompaction/Excessive Trabeculation of the Left Ventricle 61

3.18. Muscular Dystrophy 61

3.19. Peripartum Cardiomyopathy 62

3.20. Cardiomyopathy Related to Systemic Diseases 62

3.21. Cardiac Alterations Associated with Heart Transplantation 63

3.22. Pericardial Disease 63

3.23. Cardiac Masses and Thrombus 66

3.24. Valvular Heart Disease 68

3.25. Cardio-oncology 70

3.26. Vascular Disease 70

**3.26.1. Aorta** 71

**3.26.2. Extracranial Carotid Arteries** 71

**3.26.3. Renal Arteries** 71

**3.26.4. Pulmonary Arteries** 72

**3.26.5. Visceral Arteries** 72

**4. Congenital Heart Disease** 73

4.1. CT in the Evaluation of Congenital Heart Diseases 73

**4.1.1. Evaluation of Intracardiac and Extracardiac Shunts** 73

***4.1.1.1. Atrial and ventricular septal defects*** 73

***4.1.1.2. Partial and Total Anomalous Pulmonary Venous Connection*** 73

**4.1.2. Congenital Valvular Heart Disease** 74

***4.1.2.1. Tricuspid Valve/Ebstein's Anomaly*** 75

***4.1.2.2. Pulmonary Valve*** 75

***4.1.2.3. Mitral Valve*** 75

***4.1.2.4. Aortic Valve*** 75

**4.1.3. Conotruncal Anomalies** 75

***4.1.3.1. Tetralogy of Fallot*** 75

***4.1.3.2. Double-outlet Right Ventricle*** 76

***4.1.3.3. Common Arterial Trunk*** 76

***4.1.3.4. Transposition of the Great Arteries*** 76

***4.1.3.5. Congenitally Corrected Transposition of the Great Arteries*** 77

**4.1.4. Thoracic Aorta Anomalies** 77

***4.1.4.1. Coarctation and Other Aortic Abnormalities*** 77

**4.1.5. Univentricular heart** 78

**4.1.6. Other** 79

4.2. CMR in Congenital Heart Disease 80

**4.2.1. Evaluation of Intracardiac and Extracardiac Shunts** 80

***4.2.1.1. ASDs*** 80

***4.2.1.2. Patent Foramen Ovale*** 80

***4.2.1.3. Partial and Total APVC*** 81

***4.2.1.4. VSDs*** 81

***4.2.1.5. AVSDs*** 81

***4.2.1.6. Patent Ductus Arteriosus*** 81

***4.2.2. Congenital Valve Abnormalities*** 82

***4.2.2.1. Tricuspid Valve/Ebstein's Anomaly*** 82

***4.2.2.2. Mitral Valve*** 83

***4.2.2.3. Mitral Stenosis*** 83

***4.2.2.4. Pulmonary Valve*** 83

***4.2.2.5. Aortic Valve*** 83

**4.2.3. Conotruncal Anomalies** 84

***4.2.3.1. Tetralogy of Fallot*** 84

***4.2.3.2. DORV*** 84

***4.2.3.3. Common Arterial Trunk*** 85

***4.2.3.4. Transposition of the Great Arteries*** 85

***4.2.3.5. Congenitally Corrected Transposition of the Great Arteries*** 86

**4.2.4. Anomalies of the Thoracic Aorta** 87

***4.2.4.1. CoA*** 87

***4.2.4.2. Other Aortic Anomalies*** 87

**4.2.5. Univentricular Heart** 87

**4.2.6. Other** 88

**References** 89

## Preamble

In Brazil, cardiovascular diseases represent an important cause of mortality. From 2010 to 2019, 28% of deaths recorded by the Information Technology Department of the Brazilian Unified Health System resulted from this group of diseases.^1^Within the measures involved in tackling this public health problem, strategies for rational use of resources have been boosted by advances in diagnostic imaging techniques. Notably, computed tomography and magnetic resonance imaging have expanded their potential within the diagnostic and prognostic arsenal in cardiovascular disease, in addition to serving as a basis for planning different types of therapeutic procedures (surgical and/or minimally invasive).

The field of cardiovascular imaging is growing exponentially. In recent years, the emergence and improvement of new techniques for the detection of coronary artery disease, as well as those related to the anatomic and functional study of the myocardium, have allowed the expansion of indication criteria for computed tomography and cardiac magnetic resonance in the management of patients with heart disease. Meanwhile, results from large multicenter studies have allowed for more assertive definitions in the use of these methods in specific scenarios, validating and adjusting previously inconclusive indications.

Along with developments in the diagnostic field, therapeutic procedures (percutaneous and/or surgical) in cardiology have also included the development of techniques to expand their indications in the treatment of several diseases. The indication of such procedures has been accompanied by the need for more anatomic details and diagnostic accuracy for their proper use. This scenario has also been a potentiating agent for the use of computed tomography and cardiovascular magnetic resonance as an auxiliary resource in the clinical management of patients.

The Cardiovascular Computed Tomography and Magnetic Resonance Imaging Guideline II,^2^published in 2014 jointly by the Brazilian Society of Cardiology and the Brazilian College of Radiology, brought together the most robust evidence available for the application of both methods in different clinical settings. On that occasion, it also shed light on the techniques being developed in multiple areas, even if they had not yet been widely validated by large-scale studies. Therefore, this update aims to revisit the indications proposed in the previous document and to contextualize the advances in these modalities, qualifying them with the respective levels of evidence and classes of recommendation in multiple applications. The ultimate goal of this document is to serve as an information source for cardiologists, providing updated information based on the best available evidence to be used in clinical practice aiming to address routine clinical issues.

## 1. Introduction

Since the latest cardiovascular computed tomography (CT) and magnetic resonance imaging (MRI) guideline of the Brazilian Society of Cardiology (SBC), new technologies and several scientific publications involving these diagnostic methods, including multicenter and randomized studies, have contributed to reinforcing pre-existing indications, as well as to reporting new contributions of these imaging modalities within cardiology.

Cardiovascular CT and MRI, relatively recent methods in cardiology, have brought about a revolution in the understanding and treatment of heart disease. Cardiac CT has allowed the detection of coronary atherosclerosis in its earliest stages, highlighting the important prognostic value of nonobstructive coronary artery disease (CAD), previously underestimated by various cardiology societies worldwide, and reinforcing the value of anatomy to guide the treatment of obstructive CAD, being the initial method of choice in the workup of symptomatic patients without known CAD. Despite initially providing an anatomic examination, CT is increasingly proving to be a comprehensive modality in the assessment of heart disease. The assessment of ischemia using stress CT myocardial perfusion imaging (CT-MPI) and/or CT-derived fractional flow reserve (FFR_CT_) can be a validated alternative to other tests for ischemia or may complement the anatomic information in stenoses with undetermined functional repercussions.^3 – 8^The assessment of structural heart diseases, such as valvular heart disease, has also benefited from advances in CT, enabling cardiologists to better select patients for less invasive therapeutic procedures with increased success rates and reduced complication rates.^9^

Cardiac magnetic resonance (CMR) has the advantage of not using ionizing radiation and provides comprehensive morphological and functional cardiac assessments. It has expanded the diagnostic arsenal in the assessment of CAD, with high diagnostic accuracy for detecting myocardial ischemia, and is considered the gold standard in the assessment of ventricular function, myocardial infarction, and myocardial viability, being able to assess all these parameters in a single scan.^2 , 10 – 13^MRI has become an essential imaging modality for the assessment of non-ischemic cardiomyopathy, assisting in the diagnosis and prognosis of the disease and providing information for therapeutic management.^2 , 14 , 15^

To serve as a reference for the use of these imaging modalities in routine clinical practice and based on the best available evidence, SBC and the Brazilian College of Radiology (CBR) developed this document aiming to assist physicians in recommending these modalities to improve clinical decision-making that benefits patients.

### 1.1. Definition of Recommendations and Evidence

In line with previous documents developed by different national and international medical entities and societies, the information contained in this document derives from indications based on classes of recommendation and levels of evidence. Represented by indications in different clinical settings and/or specific diseases, the use of each modality is individualized for each topic proposed in this document.

Briefly, the class of recommendation involves a consensus position on the usefulness and benefit of a certain procedure, considering the safety and effectiveness of its use based on the best available evidence. The level of evidence defines the quality of the studies supporting such recommendations, including everything from expert opinions to randomized controlled trials.

**Classes of recommendation** ^2^ **:**

**Class I:** Conditions for which there is conclusive evidence and, failing that, general agreement that a given procedure is safe and useful/effective.
**Class II:** Conditions for which there is conflicting evidence and/or a divergence of opinion about the safety and usefulness/efficacy of a procedure.
  - **Class IIa:** Weight or evidence/opinion in favor of the procedure. Most approve.
  - **Class IIb:** Safety and usefulness are less well established, and there is no predominance of opinions in favor of the method.
**Class III:** Conditions for which there is evidence and/or general agreement that a procedure is not useful/effective and, in some cases, may be harmful.

**Levels of evidence** ^2^ **:**

**Level A:** Data obtained from several large randomized studies showing concurring results and/or a robust meta-analysis of randomized controlled trials.
**Level B:** Data obtained from a less robust meta-analysis, a single randomized study, or from nonrandomized (observational) studies.
**Level C:** Data obtained from consensual expert opinions.

### 1.2. Definitions of Clinical Risk Score and Pretest Probability

#### 1.2.1. Clinical Risk Score

Clinical risk scores are tools that help assess the probability of an asymptomatic individual developing atherosclerotic cardiovascular disease (ASCVD) within a given period (typically 10 years) and are calculated based on individual risk factors and population analyses. The use of risk scores is important to identify an appropriate preventive therapy, adjusting the intensity of prescribed therapy to the patient's estimated risk. This allows us to maximize the benefit of medications in patients at higher risk and to avoid unnecessary and/or excessive medication use in those at lower risk.

The Framingham risk score (FRS) has been used for many years in several countries to estimate the 10-year risk of coronary heart disease and death. However, it is currently being replaced by other risk scores, such as the global risk score, ASCVD risk estimator, Reynolds risk score, and SCORE (Systematic COronary Risk Evaluation).^16 – 19^SBC recommends the use of the global risk score, which estimates the 10-year risk of myocardial infarction, stroke, heart failure (HF), or peripheral vascular insufficiency.^16 , 17^

#### 1.2.2. Pretest Probability

The ability of a test to correctly confirm or exclude a disease will depend on its diagnostic accuracy and the prevalence of that disease in the population under investigation. Therefore, selecting the appropriate test for a given population is essential to avoid false-negative and false-positive results.

In the workup of CAD, several tests with well-established diagnostic accuracy can be used, and their choice should be based on the prevalence of CAD, as well as on local availability and experience and on patients' specific characteristics that may limit the analysis of a test.^8 , 20 , 21^

The assessment of CAD prevalence in an investigated population includes the collection of patient data, personal history, and previous test results, physical examination, and, mainly, the characteristics of the reported symptoms, thus estimating the pretest probability of CAD. The pretest probability can also be analyzed more objectively using validated scores to assist physicians in clinical decision-making regarding the test to be ordered. An older, widely used score is the Diamond-Forrester model, but recent studies have shown an overestimation of CAD pretest probability. More current scores, such as the CAD Consortium and its variants, may provide more appropriate estimates of this prevalence.^8 , 21 – 24^

Despite these discrepancies between the different scores in pretest probability estimates, an alternative stratification of suspected CAD can be used as follows^2 , 25^:

- **Low pretest probability:** < 10% probability of CAD
- **Intermediate pretest probability:** 10% to 90% probability of CAD
- **High pretest probability:** > 90% probability of CAD

## 2. Cardiovascular CT

The applications and indications of cardiac CT have expanded greatly with the development of new technologies, the publication of studies, and the experience of the physicians involved. Initially, CT was performed without contrast to evaluate coronary artery calcium (CAC). Later, using 64-slice multidetector CT (MDCT) scanners, the noninvasive assessment of anatomy was expanded to include the coronary arteries. Currently, CT is a method that offers a multimodal assessment in cardiology, providing an alternative to other diagnostic methods in the analysis of multiple anatomic and physiologic structures and parameters, such as the assessment of cardiac chamber volumes and function, identification of myocardial ischemia using MPI (under pharmacological stress or FFR_CT_, a tool that determines the functional repercussion of coronary stenosis), assessment of myocardial infarction and viability using late gadolinium enhancement (LGE), analysis of cardiac veins and pulmonary veins, and assessment of valvular heart disease, congenital heart disease, and non-ischemic cardiomyopathy.^2 , 3 , 8 , 25^

This expansion in the use of cardiac CT is one of the reasons for updating this Guideline, which will be discussed in specific topics throughout this document and in an introductory manner below to help ordering physicians understand its applications and limitations.

One of the applications of cardiac CT is to determine the CAC score, a method validated in asymptomatic patients for further stratification of cardiovascular risk. CAC testing is a rapid noninvasive assessment, without the use of iodinated contrast and with a low dose of ionizing radiation (around 1 mSv), which aims to detect and quantify CAC, being an independent predictor of mortality, coronary events, and myocardial ischemia.^2 , 26 – 30^The information provided by the CAC score on the burden of coronary atherosclerosis makes it possible to individualize the cardiovascular risk provided by clinical risk scores based on population data, further stratifying cardiovascular risk better than any other method for this purpose in asymptomatic patients and enabling clinicians to adjust preventive therapy and improve medication adherence in patients requiring medication use.^31 – 34^A suggestion on how CAC can assist in risk stratification is illustrated in the flowchart in Figure 1 .^26 – 34^

**Figure 1:** Use of coronary artery calcium (CAC) scoring as a tool for risk stratification and clinical management support. CAD: coronary artery disease; LDL: low-density lipoprotein; ASA: acetylsalicylic acid.

The main examination included within the definition of cardiac CT is coronary CT angiography (CCTA), a well-validated examination in the workup of CAD with high diagnostic accuracy and prognostic value. CCTA uses iodinated contrast, preferably nonionic contrast agents due to the lower risk of complications, requiring peripheral venous access for high-flow injection (4 to 6 mL/s) of a low volume of contrast (approximately 60 to 70 mL) compared with other CT modalities.

CCTA also uses ionizing radiation, and in recent decades there has been a significant reduction in the radiation dose due to modern CT scanners and technological advances in conventional CT scanners. The average radiation dose in retrospective acquisition with dose modulation is about 9 mSv using 64-slice MDCT scanners. However, in more modern CT scanners, this same type of acquisition uses an average dose of 5 mSv, which can be even lower if other types of acquisition are used, such as prospective (< 3 mSv) and high-pitch acquisition (< 1 mSv).^2 , 35^

In CCTA protocols, the use of negative chronotropic medications to reduce heart rate (< 60 bpm) and the use of sublingual nitrates for coronary vasodilation are strategies that increase imaging quality and diagnostic accuracy and allow the radiation dose to be further reduced by selecting acquisition modes that can be used with lower heart rates.^2 , 36^As imaging is synchronized with the electrocardiogram (ECG) for image formation, patients with arrhythmias or high heart rates may have non-diagnostic images, especially with conventional CT scanners. Another key point is that CCTA images are acquired during inspiration breath hold (< 15 seconds), which is necessary to keep the diaphragm and the topography of the heart unchanged in the chest during scanning.^2 , 36^

Among the diagnostic tests for CAD, CCTA is a noninvasive imaging option in patients with low-to-intermediate pretest probability which has been increasingly indicated owing to the publication of multiple studies, including prospective, randomized, and multicenter studies.^2 , 3 , 8 , 23 , 25 , 37 – 39^In the Prospective Multicenter Imaging Study for Evaluation of Chest Pain (PROMISE), published in 2015, 10,003 patients with suspected stable CAD were randomized to undergo CCTA or functional testing for ischemia, showing a similar number of primary outcomes (death, myocardial infarction, hospitalization for unstable angina, and major procedure-related complication) in both groups at the end of a follow-up of 2 years (3.3% vs 3.0%, respectively). Despite the similar number of positive tests in both groups (10.7% vs 11.7%), the CCTA group referred more patients to coronary angiography within 90 days (12.2% vs 8.1%) and myocardial revascularization (6.2% vs 3.2%). Interesting data from the PROMISE study show a lower rate of coronary angiography without significant stenosis in the CCTA group (27,9 vs 52,5%). Although it was considered a neutral study (primary composite outcome did not differ between the groups), a significant reduction (34%) in the composite outcome of death and myocardial infarction was observed at the end of the first 12 months of follow-up in the CCTA group (hazard ratio [HR] 0.66; p = 0.049).^23^

Another study comparing CCTA and functional testing for ischemia was the Scottish Computed Tomography of the Heart (SCOT-HEART) trial, in which 4,146 patients with suspected stable angina were randomized to undergo CTA or standard of care using treadmill test. An important instruction in the SCOT-HEART trial protocol was that, in the CTA group, patients with obstructive and nonobstructive CAD would have to receive drug therapy, while in the functional-testing group, as this test cannot detect nonobstructive CAD, drug therapy in patients with negative results was guided by the local clinical risk score. Despite a similar number of patients referred for coronary angiography and revascularization, the CTA group detected a higher rate of patients with obstructive CAD, initiated more preventive and antianginal therapies, and significantly reduced (50%) the rates of fatal and nonfatal myocardial infarction in the first 20 months of follow-up and the rates of cardiovascular deaths and myocardial infarction (41%) at the end of 5 years of follow-up.^37 , 38^

A long-awaited study in the treatment of stable CAD was the International Study of Comparative Health Effectiveness with Medical and Invasive Approaches (ISCHEMIA), in which 5,179 patients with stable angina and moderate or severe ischemia on stress testing were randomized to an invasive strategy (coronary angiography and revascularization when feasible) or a conservative strategy (initial medical therapy and coronary angiography if medical therapy failed). The study patients underwent CCTA to evaluate left main stenosis (≥ 50%), which was an exclusion criterion, and the cardiologists who followed the remaining patients were blinded to these results. The study showed that the primary outcome of cardiovascular death, myocardial infarction and hospitalization for unstable angina, HF, or resuscitated cardiac arrest was similar in both groups, demonstrating the safety of medical therapy in this population without left main stenosis on CCTA.^39^Further analysis of data from the ISCHEMIA trial showed that the severity of stenosis on CCTA was associated with greater clinical risk, but not with the ischemia severity after adjustment for anatomic severity.^40^

Given current evidence, CCTA is a well-validated test not only for its diagnostic and prognostic value in CAD but also for improved further stratification of cardiovascular risk and guidance on clinical decision-making in patients with CAD, being an appropriate initial option in this assessment, especially in patients without known CAD, as recommended by other international guidelines.^3 , 8^Suggested flowcharts for the investigation of patients without known CAD and with known CAD are shown in Figures 2 and 3 , respectively.^3 , 8 , 41 , 42^

**Figure 2:** Flowchart for assessment of coronary artery disease (CAD) in patients without known CAD. CT: computed tomography; LMCA: left main coronary artery; FFR: fractional flow reserve.

**Figure 3:** Flowchart for assessment of coronary artery disease (CAD) in patients with known CAD. ECG: electrocardiogram; CT: computed tomography; MRI: magnetic resonance imaging; CT-MPI: CT myocardial perfusion imaging; FFR: fractional flow reserve; PET: positron emission tomography.

Cardiac CT modalities that can also be used in CAD assessment include the detection of myocardial ischemia by stress CT-MPI or FFR_CT_.^3^Stress MPI, generally combined with vasodilators such as adenosine and dipyridamole, has been validated for approximately 2 decades, with both diagnostic and prognostic value. In a Coronary Artery Evaluation using 320-row Multidetector Computed Tomography Angiography and Myocardial Perfusion (CORE320) study, CT-MPI (not combined with anatomic CTA data) in relation to myocardial scintigraphy (single photon emission CT [SPECT]) had better diagnostic accuracy than invasive coronary angiography, showing superior sensitivities for the diagnosis of significant left main stenosis (CT-MPI 92% vs SPECT 75%), 3-vessel CAD (CT-MPI 92% vs SPECT 79%), 2-vessel CAD (CT-MPI 89% vs SPECT 68%), and 1-vessel CAD (CT-MPI 83% vs SPECT 41%).^43^

Evaluation of myocardial ischemia using FFR_CT_, a more recent method in cardiology and validated in clinical studies, has the advantage of evaluating the functional significance of coronary artery stenosis without the need for pharmacological stress or extra doses of iodinated contrast and radiation, although requiring the use of software designed for this additional analysis. FFR_CT_can therefore assist in decision-making in patients, for example, with moderate stenosis, maintaining management with medical therapy if negative or ordering invasive coronary angiography if positive to continue the diagnostic workup.^5 – 7^

Other different uses of cardiac CT are better detailed in specific sections of this Guideline, showing its multimodality features within cardiology.

### 2.1. Coronary Calcium Score

#### 2.1.1. Current Evidence on Cardiovascular Risk Stratification by CAC Scoring

In 1990, Arthur Agatston used electron beam CT (EBCT) to identify and quantify calcified coronary atherosclerosis.^44^Since then, after more than 3 decades of publications and follow-up of several large population cohorts, the quantification of atherosclerotic burden using the CAC score has established itself as the best additional tool for predicting the risk of major cardiovascular events among the currently available tools in clinical practice.^45^

#### 2.1.1.1. Technique

- CAC testing is performed without the use of contrast, often on MDCT scanners, which have wider clinical utility than EBCT scanners. ECG-synchronized axial images are acquired covering the entire cardiac area, in the craniocaudal direction. Total scanning duration is about 10 minutes, and the current radiation dose is extremely low, about 0.8 to 1 mSv.^46^
- CAC testing detects the calcified atheroma plaque component, which can be quantified in several ways (Agatston, volume, and density scores, among others). The Agatston score is the most widely used one in clinical practice, as it is a reference for population databases such as the Multi-Ethnic Study of Atherosclerosis (MESA) and Framingham Heart Study (FHS).^47^

#### 2.1.1.2. Independent Risk Marker for Cardiovascular Events

Early studies helped determine the role of CAC, such as the South Bay Heart Watch Study,^46^which showed that CAC further risk-stratified patients deemed at intermediate risk by the FRS, and the St. Francis Heart Study, which, in 2005, showed a much higher cardiovascular risk when comparing groups with CAC > 400 vs CAC = 0.^48^

Larger cohort studies of asymptomatic patients in primary prevention have been conducted over the past 15 years. Studies conducted in the United States, such as the MESA,^49^which prospectively followed 6,814 patients aged 45-84 years, CAC Consortium (largest cohort in the literature with 66,363 patients),^50^and Dallas Heart Study (DHS),^51^the German Heinz Nixdorf Recall (HNR) study, including 4,814 patients aged 45-74 years,^52^and the Dutch Rotterdam Study,^53^including 7,983 slightly older patients (age > 55 years), reinforced concepts such as CAC being higher in men, there being differences across ethnicities, and differences that increase with age, thus contributing to a better understanding of the process of coronary atherosclerosis.

These studies also helped to establish the relationship between CAC scoring and cardiovascular risk. For example, Detrano et al., using data collected from the MESA cohort, showed that CAC was independently related to the incidence of cardiovascular events, and that a doubling of the CAC score resulted in a 25% increase in the risk of a major event during a follow-up of 3.8 years.^54^

#### 2.1.1.3. Basic Concepts

1. Higher atherosclerotic burden detected by the CAC score indicates greater risk of obstructive CAD and major cardiovascular events. This pattern is similar across all study populations.
2. Positive CAC scores can be classified as low (1-100), moderate (101-400), high (> 400), and very high (> 1,000) ( Table 1 ).

**Table 1:** Relative risk (RR) for cardiovascular events and degree of coronary calcification according to absolute coronary artery calcium (CAC) score

Absolute CAC score	RR for cardiovascular events	Degree of calcification
0		No calcification
1-100	1.9 (1.3-2.8)	Mild
101-400	4.3 (3.1-6.1)	Moderate
401-1,000	7.2 (5.2-9.9)	High
> 1,000	10.8 (4.2-27.7)	Very high

* Adapted from Azevedo, Rochitte, and Lima^55^and Greenland et al.^56^

Of particular clinical interest is an Agatston score greater than 100 or a 75th percentile ranking or higher for sex, age, and ethnicity (useful in young patients with absolute scores still below 100), being regarded as an aggravating factor for cardiovascular risk by further risk-stratifying patients to a risk category above that obtained with the clinical tool used (eg, global risk score, 10-year ASCVD, FRS).^16^It is worth noting that, in patients with diabetes mellitus (DM), the cutoff threshold is lower, with CAC greater than 10 being considered an additional risk factor.^57^Absolute scores are more strongly associated with major cardiovascular events than percentile ranks, at least in the short/medium term.^58^An elevated coronary calcium score, greater than 400 (Agatston method), is related to a higher incidence of detectable ischemia and a risk of events similar to that of symptomatic patients.^59^

#### 2.1.1.4. Target Population

Asymptomatic patients in primary prevention and with a marginal or intermediate clinical risk are those who benefit most from CAC testing.^60 , 61^

It should be noted that scores that use traditional risk factors tend to overestimate cardiovascular risk compared with the incidence of events in the population included in more recent studies,^62^which may lead to a categorization that will require greater use of drugs and complementary tests, with questionable clinical benefit.

Populations with risk factors not included in traditional scores, such as DM and familial hypercholesterolemia (FH), and with a positive family history of premature CAD are also associated with potential clinical benefits with the measurement of coronary plaque burden using CAC.^63 – 67^

CAC testing should not be routinely used in symptomatic patients in the emergency department, as the mechanism potentially involved (plaque rupture, thrombosis, non-calcified plaque) may not be properly characterized in this test. Furthermore, even in the outpatient setting, the use of CAC in symptomatic individuals is also not recommended. Although a CAC score of zero is known to have a very high negative predictive value and a good medium-term prognosis, we also know that up to 10% of these patients have non-calcified CAD, with approximately 2% of them presenting with lesions > 50% in a cohort of 1,753 patients.^68 , 69^

Likewise, patients in secondary prevention, where cardiovascular risk is already considered very high, are also not candidates for CAC testing.

#### 2.1.1.5. Distribution

Studies have demonstrated that, similar to coronary angiography and CCTA, in which the segment involvement score (SIS) can be calculated,^70^the location and distribution of calcified plaques in CAC scoring have prognostic implications. Thus, plaque burden concentration mostly in the left main coronary artery (especially if above 25% of total CAC) is independently associated with an increase of 6% to 9% in cardiovascular mortality, after adjusting for plaque burden in the remaining coronary arteries. Patients with 1-, 2-, and 3-vessel CAC also have a continuous and progressive worsening in the rate of cardiovascular events, as demonstrated in the FHS with a 7-year follow-up.^71 , 72^A study of the CAC Consortium population showed that integrating the regional distribution of CAC into traditional CAC improved further risk stratification.^73^

#### 2.1.1.6. Age to Start CAC Testing

There is no determined standard age, and clinical data obtained from the patient's history-taking and physical examination should be considered.

An interesting recent study using data from the CAC Consortium, including 22,346 patients aged 30 to 50 years, aimed to determine the ideal age at which a first CAC scan for subclinical atherosclerosis would have the highest utility according to the presence of ASCVD risk factors. Compared with patients without risk factors, those with DM developed a positive CAC 6.4 years earlier, whereas patients with other traditional risk factors developed CAC > 0, on average, 3.3 to 4.3 years earlier. The model used in the study indicated that the ideal age for a potential first CAC scan was approximately 37 years in men and 50 years in women with DM. At the other end, in patients without risk factors, the ideal age was approximately 42 years in men and 58 years in women.^74^

#### 2.1.1.7. Frequency

For patients with a positive CAC (> 0), the general recommendation is not to repeat the test, especially if cardiovascular risk has already been further stratified in the test in question. It should be noted that there is an inter-scan variability of 15%^56^and a natural progression of atherosclerosis of 15% to 20% per year,^75^which may also be affected by statin therapy^76^and regular physical activity,^77^as both situations lead to an increase in the calcified atheroma plaque component. Furthermore, no clinical algorithm uses CAC progression to define treatment, as such information is not clearly superior to baseline CAC as a prognostic indicator.

When CAC = 0, 20% to 25% of patients convert to a positive CAC (generally low scores) within 4 to 5 years. A recent study showed that the time to conversion from CAC = 0 was 5 to 7 years in low-risk patients (< 5% risk by the ASCVD score), 3 to 5 years in low-to-intermediate-risk patients (5% to 10% risk by the ASCVD score), and 3 years in high-risk patients with diabetes. Therefore, in general, 3 to 5 years seems to be the recommended time frame for repeat scanning in CAC = 0.^78 , 79^

Patients with 2 zero-CAC scores have the best event-free survival rate (1.4% at 10 years).^28^

#### 2.1.1.8. The Power of Zero CAC

Over the years, after the concept of atherosclerosis burden as the main marker of cardiovascular risk became established, the focus of studies shifted to the protective effect of the absence of calcified atherosclerosis, since the very low rate of events in patients with zero CAC (approximately 0.1% per year) has always attracted attention. This population represents approximately one-third of patients even in scenarios where the expected prevalence of atherosclerosis would be higher, such as in patients with chest pain and diabetes.^69 , 80^A meta-analysis including 29,312 patients with zero CAC from 13 studies showed a 4-year event rate of 0.47% in this population.^81^

Zero CAC has been shown to play a role as a "negative" risk factor in the absence of calcified atherosclerosis as detected by the Agatston score, surpassing clinical risk factors for mortality prediction. In a study of 44,052 patients, patients with no traditional risk factors and CAC > 400 had a substantially higher cardiovascular risk than those with 3 or more risk factors and CAC = 0 (16.89 vs 2.72 events per 1,000 person-years).^82^

In the CAC Consortium population, which is the study with the largest sample size available in the literature (66,363 patients, with a mean age of 54 years and 33% women), 45% had zero CAC (mean age of 45 years) and low rates of major cardiovascular events (0.32 to 0.43 per 1,000 person-years). Although still rare, cancer was the leading cause of death in this population.^83^This concept was consolidated in an unprecedented way that the 2018 American consensus on dyslipidemia included the possibility of reclassifying the cardiovascular risk of those with zero CAC to a lower level, including the discussion of the discontinuation of lipid-lowering medications (except in smokers, those with DM, those with FH, and those with a positive family history of premature CAD).^60^

#### 2.1.1.9. Very High CAC (> 1,000 Agatston Units)

The population with a considerable calcified atherosclerosis burden has also been studied more closely in recent years.

Recently, a cohort study of patients with CAC > 1,000 showed that the risk of cardiovascular events in this population surpasses even that of patients in secondary prevention with a suboptimal lipid profile, such as those evaluated in the Further Cardiac Outcomes Research With PCSK9 Inhibition in Subjects with Elevated Risk (FOURIER) trial.^84^A study of patients from the CAC Consortium found similar results,^85^suggesting a more aggressive treatment and better cost-benefit ratio in the use of functional testing for ischemia in this subgroup of patients.

#### 2.1.1.10. Special Populations

#### 2.1.1.10.1. Diabetes Mellitus (DM)

Even in the population of individuals with DM, who would theoretically be at high risk, cardiovascular risk is heterogeneous. This population may benefit from individualization with the use of CAC.^65 , 66 , 80^Raggi et al. evaluated 10,377 patients, 903 of whom had DM, and showed that 5-year survival was the same in patients with and without DM when CAC = 0.^26^In the Prospective Evaluation of Diabetic Ischemic Disease by Computed Tomography (PREDICT) study, including 589 patients with DM and without CAD, higher CAC scores indicated greater risk of unfavorable cardiovascular outcomes.^86^The use of CAC is recommended by the 2021 American Diabetes Association guidelines, starting at the age of 40.^63^

#### 2.1.1.10.2. Family History of Premature CAD

Patients with a positive family history of premature CAD (event in a first-degree relative before the age of 55 in men or before the age of 65 in women) have a higher cardiovascular risk and are not usually included in the main clinical scores.

In the Coronary Artery Risk Development in Young Adults (CARDIA) study, including patients aged 32 to 46 years, CAC > 0 was not uncommon, especially in the presence of a risk factor.^87^Furthermore, Miedema et al. showed that CAC may have prognostic value in young (< 40 years of age) low-risk patients.^64^

#### 2.1.1.10.3. Young Patients

Another scenario in which the use of CAC can be considered is in young patients (especially under 40 years of age), supposedly at low cardiovascular risk according to traditional clinical scores. As age has a major impact on scores, the risk for young patients may be underestimated, below the threshold for recommending statin use. Two prospective studies of young patients (CARDIA^87^study and Prospective Army Coronary Calcium [PACC]^88^project), with mean ages of 40.3 and 42.9 years, respectively, showed that coronary calcium was associated with a 3- to 12-fold increased risk of coronary events compared with patients with zero CAC.

#### 2.1.1.10.4. Familial Hypercholesterolemia (FH)

Even in populations with high baseline risk, such as those with FH, CAC scoring appears to help better discern cardiovascular risk. Miname et al., in a study of 206 patients with molecularly proven FH receiving statin therapy, with a mean residual low-density lipoprotein cholesterol (LDL-c) of 150 ± 56 mg/dL, showed that a zero CAC was associated with low risk of major cardiovascular events during a median follow-up of 4 years.^67^The events occurred only in those with CAC > 0 (incidence of 2.6% and 4.4% per year in those with CAC 1-100 and CAC > 100, respectively). More recently, Gallo et al. found similar results in 1,624 patients with FH on lipid-lowering therapy with statins and/or ezetimibe (LDL-c under treatment, 170 mg/dL) followed for a median of 2.7 years, with event rates of 0.47%, 2.1%, and 14.2%, respectively, for CAC = 0, 1-100, and > 100 during follow-up.^89^Likewise, Sandesara et al., in a study of patients from the MESA cohort who had LDL-c > 190 mg/dL (mean LDL-c, 215 ± 27 mg/dL), observed that individuals with zero CAC had fewer events (annual absolute incidence of events of 0.4%) than those with CAC > 100 (2% of events per year), after 14 years of follow-up.^90^

#### 2.1.1.11. Comparison of the Use of CAC vs CCTA in Risk Stratification of Cardiovascular Events

With the advent of CT scanners with new technologies, imaging radiation dose decreased substantially, reaching a 78% reduction in the Prospective Multicenter Registry on RadiaTion Dose Estimates of Cardiac CT AngIOgraphy IN Daily Practice in 2017 (PROTECTION VI) study in relation to the original study,^91^leading to proposals to extend the use of CCTA to asymptomatic populations. This aims to improve further cardiovascular risk stratification, since non-calcified plaques would become visible, in addition to small calcified plaques not detected in CAC, in which the spatial resolution proposed in the Agatston score (3 mm) is much lower than that of CCTA (0.5-0.625 mm). However, despite a lower threshold for detecting atherosclerosis, the results of the studies are controversial, either neutral or favorable to the new strategy,^92 , 93^not showing clear superiority in risk stratification in the overall asymptomatic population. Senoner et al., in a study of 6,439 patients, showed that in those with zero CAC, even when only non-calcified plaques are found, the event rate continues to be low.^94 , 95^

#### 2.1.1.12. Use in Clinical Guidelines

CAC has a class IIA indication in the American Guideline on the Management of Blood Cholesterol for patients at borderline and intermediate cardiovascular risk (calculated risk of death, myocardial infarction, and ischemic stroke at a 10-year ASCVD score of 5% to 7.5% and 7.5% to 19.9%, respectively), when clinical management is uncertain and has been incorporated into the clinical decision algorithm to individualize cardiovascular risk, with replication of this concept in the American Primary Prevention Guidelines.^60 , 96^

In the Brazilian Guidelines for Dyslipidemia and Atherosclerosis Prevention and for Cardiovascular Prevention,^16 , 91^CAC use is recommended at moderate risk according to the Global Risk Score (5%-10% in women and 5%-20% in men). According to the U.S. guidelines, a CAC score > 100 Agatston units or > 75th percentile for age and sex indicates statin therapy to reduce LDL-c. A CAC score of 1-99 favors statin therapy (especially in people aged > 55 years). A CAC score of zero indicates low risk in the medium term, leaving the option of therapy to a shared decision between physician and patient, except in those with DM, smokers, and those with a family history of premature CAD, in whom statin therapy is recommended.^18 , 54 , 60 , 96^

#### 2.1.1.13. Outstanding Issues and Perspectives

Cost-effectiveness analyses have not yet provided a definitive answer. These analyses are difficult to perform for any test, since many variables are involved and change depending on the location, testing and treatment costs, patient compliance, and intention to pay for it. An analysis in the U.S. scenario indicated CAC as cost-effective in male patients at intermediate risk according to clinical risk scores.^97^

Further studies are needed to determine whether risk stratification based on CAC improves clinical outcomes, although we know that they are not simple to perform and, to date, even much older methods used in cardiology have not been able to provide such information.

The visual measurement of coronary calcification, or even by the Agatston score in non-gated chest CT, appears to have a good correlation with values obtained with dedicated CT imaging, and its use is recommended by the Society of Cardiovascular Computed Tomography (SCCT), with a dedicated classification for this purpose, the CAC data and reporting system (CAC-DRS).^98^

Finally, the increasing use of artificial intelligence has accelerated the process, making it possible to read the results in seconds and integrate CAC information with other data obtained concomitantly, such as the quantification of epicardial adipose tissue to increase the power of predicting events (radiomics).^99^

#### 2.1.2. Role of CAC Score in Risk Stratification Defined by Traditional Clinical Scores

As previously mentioned in this document, several scores are available for cardiovascular risk stratification. Despite some variations between cardiology societies and atherosclerosis prevention groups, individuals are usually classified into the following categories according to the intensity of their risk factors:

1. Low risk: 10-year absolute event risk < 5%.
2. Intermediate risk: 10-year absolute event risk of ≥ 5% to ≤ 20% in men and of ≥ 5% to ≤ 10% in women.
3. High risk: 10-year absolute event risk > 20% in men and > 10% in women.

Recently, some international societies have introduced a borderline risk group: 10-year absolute event risk of 5% to < 7.5%.^60^

Despite their great utility, these scores have limitations mainly in predicting cardiovascular events in the intermediate risk category. In this context, the investigation of subclinical atherosclerosis using CAC can provide additional information for a better individual risk stratification.^100^

The representative MESA study evaluated the impact of CAC measurement on the prediction of coronary events in men and women of different ethnicities in the United States followed for approximately 4 years.^54^Compared with patients with no coronary calcium, the risk of death or acute myocardial infarction (AMI), adjusted for other risk factors for CAD, was increased by 7.7 times for individuals with CAC scores between 101 and 300 Agatston units and 9.7 times for those with CAC scores > 300 Agatston units (p < 0.001 for both comparisons). Despite the differences in the prevalence of coronary calcification between different ethnic groups, CAC added prognostic accuracy to traditional risk factors in a similar way across these groups. In a subanalysis of the MESA cohort,^101^after a follow-up of 58 years, in addition to improving discrimination (receiver operating characteristic [ROC] curve), CAC significantly improved risk reclassification rates. The impact was greater on those individuals previously considered to be at intermediate risk by the FRS: 16% were reclassified as high risk, while 39% were reclassified as low risk (net reclassification index [NRI] 0.55; 95% CI, 0.41-0.69; p < 0.001).

In another prospective study, the addition of CAC to traditional risk factors improved the prediction of cardiovascular events in relation to the FRS, increasing the area under the ROC curve (AUC) from 0.63 to 0.68 (p < 0.001). However, CAC did not significantly change prediction in individuals in the < 10% risk category of the FRS.^28^An exception to this lower risk category would be individuals with a positive family history of premature CAD, whose association with high CAC (> 80th percentile) identified a group at higher risk who would potentially benefit from aggressive (vs conservative) lipid-lowering therapy, according to a subanalysis of the St. Francis Heart Study.^102^In the high-risk category, low CAC does not adequately reclassify individuals to lower risk and, therefore, should not indicate a reduction in therapy aimed at these patients.^28 , 101 , 103^Therefore, patients classified at intermediate risk by the FRS are those who benefit most from the addition of CAC due to the increased possibility of a correct reclassification, which could lead to changes in primary prevention goals.

#### 2.1.2.1. Comparison of CAC Scoring with Other Cardiovascular Risk Stratification Methods

In recent years, several studies have been published comparing CAC vs other tools for subclinical atherosclerosis detection and prognostic assessment, such as the ankle-brachial index (ABI), carotid intima-media thickness (IMT), micro or macroalbuminuria, and high-sensitivity C-reactive protein (CRP).^31 , 104^

A subanalysis of the MESA study demonstrated that IMT is associated with the presence and progression of coronary calcification.^105^Another substudy demonstrated that the FRS alone had an AUC of 0.77 for the prediction of cardiovascular events, FRS combined with IMT > 1.0 mm had an AUC of 0.78 (1.3 more cardiovascular events), and FRS combined with CAC > 0 had an AUC of 0.81 (2.1 times more cardiovascular events).^106^Brook et al. compared the accuracy of CAC, IMT, CRP, and carotid plaque area for identifying obstructive CAD, defined as luminal stenosis of at least 50% on CCTA, and found that the AUCs for CAC and carotid plaque area were similar in predicting the presence of significant coronary atherosclerosis and superior to CRP and IMT.^107^

In the MESA study, Yeboah et al. directly compared the main risk markers regarding their accuracy in the prediction of cardiovascular events.^31^A total of 1,330 patients without DM classified at intermediate risk by the FRS were followed for a median of 7.6 years, and the following markers for cardiovascular risk prediction were evaluated: CAC, carotid IMT, ABI, brachial artery flow-mediated dilation (FMD), CRP, and family history of premature CAD. Additionally, they sought to evaluate the correct reclassification of patients based on the results of these markers, according to the NRI. After follow-up, 94 patients (7.1%) had cardiac events (defined as myocardial infarction or angina followed by revascularization, resuscitated cardiac arrest, or CAD death) and 123 (9.2%) had cardiovascular events (CAD or stroke). The study observed that CAC, ABI, CRP, and family history were independent predictors of CAD risk, while IMT and brachial artery FMD showed no independent ability to predict CAD risk. Furthermore, CAC was the marker that most added to the FRS ability to predict cardiovascular events, as shown by improved AUC (0.623 vs 0.784), and was the marker that best further risk-stratified patients into higher or lower risk – 65% of patients were correctly reclassified into higher or lower risk, compared to 16% for family history, 10% for IMT, and 8% for CRP.

Therefore, CAC proves to be the most accurate subclinical atherosclerosis detection tool for refining risk stratification in asymptomatic patients.

#### 2.1.3. Use of CAC Score to Support Pharmacotherapy Decision-making

The use of CAC is currently considered more appropriate for further stratification of asymptomatic individuals at intermediate cardiovascular risk. Based on this further risk stratification, information from CAC could assist in the shared decision not to initiate/discontinue more aggressive anti-atherosclerotic therapy with lipid-lowering drugs (in patients further stratified to low risk) or antiplatelet agents^108^or to initiate/intensify anti-atherosclerotic therapy with lifestyle modifications, lipid-lowering drugs, and antiplatelet agents (in patients further stratified to high risk). There are no published randomized controlled trials comparing this CAC-guided strategy vs the strategy guided by usual risk stratification, which raises criticism against the use of the method. However, given the extensive evidence of CAC as an independent marker of cardiovascular risk and the recognized role of statins in reducing this risk, this approach is considered a plausible strategy and the use of CAC is suggested in several guidelines for the prevention and treatment of atherosclerosis when the indication of statins is unclear in certain risk categories.^19 , 60 , 96^

When the 2013 American College of Cardiology/American Heart Association (ACC/AHA) dyslipidemia treatment and atherosclerosis prevention guidelines (which already recommended the use of statins when the 10-year absolute risk of cardiovascular events [death, myocardial infarction, or stroke] was ≥ 7.5% and considered their use when the risk was 5% to < 7.5%) were applied to the MESA participants, Nasir et al. found that approximately 57% of individuals without DM in the borderline risk group (risk of 5% to < 7.5% by clinical score) had CAC = 0 and a very low 10-year event rate of 1.5% (statin not recommended), in contrast to those with CAC > 0, who had a 10-year event rate of 7.4% (statin considered).^61^Likewise, individuals in the intermediate risk group (risk of 7.5% to 20% by clinical score) with CAC = 0 had a 10-year event rate of 4.6% (therefore, statin no longer recommended), whereas those with CAC > 0 had a rate of 10.4% (statin recommendation reinforced). Therefore, according to this analysis, more than 50% of individuals in the borderline and intermediate risk groups could be reclassified as low risk, being spared from long-term statin therapy and its associated costs and potential side effects. In other words, CAC would be able to significantly reduce the number needed to treat (NNT) to prevent a cardiovascular event in these groups of individuals, optimizing resources with the use of statins. Conversely, those with coronary calcification demonstrated by CAC could benefit from more aggressive anti-atherosclerotic therapy. Approximately half of the MESA Justification for the Use of Statins in Prevention: an Intervention Trial Evaluating Rosuvastatin (JUPITER) population^109^had CAC scores of 0, despite high CRP, and had a low 5-year event rate, generating an NNT of 549 for treatment with rosuvastatin 20 mg to prevent a coronary event. However, most events (74%) occurred in the subgroup of individuals with CAC > 100 Agatston units. Considering treatment only for this subgroup, the NNT would be much more favorable: only 24 to prevent a coronary event.

In a retrospective study of military personnel from the U.S. Army's Walter Reed Medical Center,^110^the impact of statin use stratified by CAC scores was evaluated in 13,644 participants, who were followed for a median of 9.4 years, after analysis adjusted for comorbidities. Statin therapy was associated with a lower risk of major cardiovascular events (AMI, stroke, and cardiovascular death) in individuals with CAC > 0 (adjusted subhazard ratio, 0.76; 95% CI, 0.60-0.95; p = 0.015), but not in participants without coronary calcification (adjusted subhazard ratio, 1.00; 95% CI, 0.79-1.27; p = 0.99). Furthermore, the impact of statin use on the reduction of events was related to the severity of coronary calcification, being greater with higher CAC scores. However, as it is a retrospective study and not a randomized controlled trial, it has several limitations and cannot provide definitive evidence of the use of CAC as a guide to lipid-lowering therapy.

The Dutch Risk or Benefit in Screening for Cardiovascular Disease (ROBINSCA) trial,^97^is the first randomized trial to compare a CAC-guided risk stratification and treatment strategy vs clinical stratification-guided strategy. The study investigated the impact on coronary event reduction of 2 screening strategies as a guide to anti-atherosclerotic therapy: assessment of the CAC score followed by treatment according to local guidelines for patients with CAC > 100 Agatston units vs assessment with a clinical score (Systemic COronary Risk Evaluation [SCORE]) followed by treatment for patients with a score > 10%. The use of CAC significantly reduced the number of individuals indicated for preventive treatment compared to SCORE (relative reduction women: 37.2%; men: 28.8%).

Table 2 presents the recommendations for the use of CAC scoring in different clinical scenarios.

**Table 2:** Recommendations for the use of the coronary artery calcium score according to different clinical scenarios

Indication	Class of recommendation	Level of evidence
Further risk stratification in asymptomatic patients with an intermediate clinical risk score.^2 , 8 , 16 , 18 , 21 , 24 , 25 , 32 , 33 , 111 – 114^	I	A
Further risk stratification in asymptomatic patients with an intermediate clinical risk score for guidance on drug therapy for primary prevention.^2 , 8 , 16 , 18 , 21 , 24 , 25 , 32 , 33 , 111 – 114^	IIa	B
Further risk stratification in asymptomatic patients with diabetes mellitus or metabolic syndrome and an intermediate clinical risk score.^2 , 16 , 26 , 86^	I	B
Further risk stratification in asymptomatic patients with a low clinical risk score and family history of premature CAD.^2 , 16 , 25 , 64 , 87^	IIa	B
Further risk stratification in asymptomatic patients with heterozygous familial hypercholesterolemia.^67 , 115^	I	B
Screening for investigation of myocardial ischemia in asymptomatic patients with diabetes mellitus.^2 , 116^	IIa	B
Further risk stratification in patients without known CAD undergoing functional testing for ischemia with negative results.^37 , 38 , 117 , 118^	IIa	B
To rule out significant coronary stenosis in symptomatic patients with suspected stable angina or acute coronary syndrome.^68 , 119^	III	B
Use in patients with known obstructive CAD.	III	C

CAD: coronary artery disease.

### 2.2. CCTA in Suspected Stable Angina without Known CAD

#### 2.2.1. As a First-choice Option in the Assessment of Non-acute Chest Pain

Several societies have recently published documents on the optimal approach to investigate chest pain, particularly in cases where cardiac origin and/or CAD are suspected.^8 , 21^American, European, and Brazilian societies currently recommend that pretest probability be determined for the presence of obstructive CAD and that a recommendation for subsequent investigation be made according to this probability.

There are several pretest probability scores, but agreement between them is not always substantial.

Despite the differences, international guidelines consider that the definition of pretest probability should take into account the symptoms, sex, and age of patients. This Guideline recommends that, in patients with a pretest probability above 10%,^111^in the absence of another clear etiological diagnosis, both the presence of obstructive CAD and subsequent investigation should be considered.

How to investigate the presence of CAD in these cases will depend on the presence of previous CAD. Cases of documented previous obstructive CAD are covered elsewhere in this document, and in this session, we will discuss management in individuals without known previous obstructive CAD. In these cases, according to the guidelines, provocative testing for ischemia, such as exercise ECG, stress echocardiography, and nuclear stress testing or stress cardiac MRI, can be considered in the same way that the investigation can be conducted with noninvasive anatomic assessment using CCTA. To make an individualized decision about which diagnostic method to use, the local experience of those who work with the methods should be considered, as well as the availability and affordability of the method.

For a complete assessment of CCTA performance in the workup of obstructive CAD, the following should be considered:

1. Diagnostic accuracy of CCTA for identifying obstructive CAD in different populations.
2. Prognostic value of CCTA findings.
3. Efficacy studies of CCTA vs other methods for the workup of CAD.
4. Cost-effectiveness of different CAD workup strategies.

#### 2.2.1.1. Diagnostic Accuracy

Since the publication of the Coronary Artery Evaluation using 64-row Multidetector Computed Tomography Angiography (CORE64) study in 2008, several studies have demonstrated the high diagnostic accuracy of CCTA compared with invasive coronary angiography.^112^More recently, a meta-analysis including more than 5,000 individuals demonstrated that CCTA has a sensitivity of approximately 95% and a specificity of approximately 79%.^113^

#### 2.2.1.2. Prognostic Value

Several studies have demonstrated the prognostic value of CCTA findings.^120 – 122^These studies have demonstrated that the presence of obstructive or nonobstructive plaques is a predictor of events, as well as the presence of higher risk characteristics in plaques detected by CT and the extent of CAD defined by the number of segments with atherosclerotic plaques on CTA.

#### 2.2.1.2.1. Efficacy Studies of CCTA vs Other Diagnostic Methods for the Investigation of Obstructive CAD

At least 2 large randomized studies have evaluated the use of CCTA in the investigation of non-acute CAD. In the PROMISE study (discussed earlier),^23^assessment by CCTA was not superior to assessment by provocative testing for ischemia. The SCOT-HEART trial, including approximately 4,000 participants randomized to either the usual assessment with exercise ECG or the addition of CCTA to standard care, showed an approximately 40% reduction in the myocardial infarction rate over the course of 5 years.^38^Also, there was no persistent increase in the use of invasive coronary angiography or revascularization in the CTA group.

In a meta-analysis of 4 studies with this design, as well as in a large retrospective study conducted in Denmark, a finding close to the result identified in the SCOT-HEART trial was demonstrated, with a significant reduction in myocardial infarction, but no difference in mortality, with the use of CCTA.^123 , 124^

In summary, these studies suggest that, for the population of patients without previous CAD, initial investigation with CCTA results in a lower rate of subsequent myocardial infarction. However, none of the studies robustly evaluated subgroups to identify in which populations there is a greater or lesser benefit from this strategy.

#### 2.2.1.2.2. Cost-effectiveness Studies

To date, cost-effectiveness data for the use of CCTA in the Brazilian setting are limited. Nevertheless, a recent Brazilian study concluded that the inclusion of CTA in the list of the Brazilian Unified Health System's diagnostic arsenal would represent a cost-effective strategy in most of the scenarios evaluated.^125^Although previous studies suggest that the initial strategy with CCTA followed by investigation with provocative testing for ischemia in cases of abnormal CT findings is the most cost-effective strategy for settings in the United States and the Netherlands, it is not possible to state that the same would occur when modeling the data for the Brazilian setting.^125 , 126^However, based on the currently available data, CCTA appears to be at least as cost-effective as other strategies used in the investigation of non-acute CAD.

#### 2.2.2. In Low-risk Patients with a Positive Functional Test

CCTA may be ordered in the initial investigation of patients with suspected CAD or in those with a previous functional test for ischemia. In the latter scenario, patients with previous inconclusive, conflicting, or clinically discordant functional test results may benefit from the correct indication of CCTA.^2 , 3 , 8 , 25 , 127 – 129^

In a study by Abidov et al., 199 patients with prior stress tests underwent CCTA for CAD and were followed for at least 2 years.^127^In patients with positive stress tests, CCTA showed stenosis > 50% in only 19% of patients. Of the 199 patients, 63% had an indication for coronary angiography before CCTA. After CCTA, coronary angiography was performed in only 16% of patients during the 2-year follow-up. Such findings highlight the important diagnostic and prognostic value in this population with a previous functional test for ischemia.

Another validated indication for CCTA is its use as an alternative to invasive coronary angiography in patients with suspected stable CAD and intermediate pretest probability of CAD, both due to clinical symptoms and abnormal results of other cardiac tests, such as functional tests for ischemia.

In the international multicenter Coronary Computed Tomographic Angiography for Selective Cardiac Catheterization (CONSERVE) trial,^128^1,631 patients were randomized to invasive coronary angiography or CCTA (selective strategy), with a 1-year follow-up, and the same number of cardiovascular events (4.6%) was observed in both groups. In the selective strategy group, only 23% of patients underwent coronary angiography during follow-up (77% reduction), with a reduction in the use of coronary angiography without obstructive stenosis (24.6% vs 61.1%), reduction in revascularization (13% vs 18%), and total diagnostic cost savings (57%) in favor of the CCTA group.

In the Diagnostic Imaging Strategies for Patients with Stable Chest Pain and Intermediate Risk of Coronary Artery Disease (DISCHARGE) trial,^129^3,561 patients with stable chest pain were randomized to invasive coronary angiography or CCTA in 26 European centers with a follow-up of 3.5 years. The CCTA group had the same number of patients with obstructive CAD as the coronary angiography group (25.7% in each group) and a similar number of cardiovascular events during follow-up (2.1% vs 3.0%), even performing coronary angiography in only 22% of patients (78% reduction). However, the CCTA group had a significant reduction in major procedure-related complications (0.5% vs 1.9%), reduction in the use of coronary angiography without obstructive stenosis (27.5% vs 74.3%), and reduction in revascularization (14.2% vs 18.0%).^129^

Table 3 presents the recommendations for the use of CCTA in the investigation of stable CAD.

**Table 3:** Recommendations for the use of coronary computed tomography angiography in the assessment of stable CAD

Indication	Class of recommendation	Level of evidence
Evaluation of symptomatic patients with suspected stable CAD and low or intermediate pretest probability.^2 , 3 , 8 , 23 , 25 , 112 , 130 – 132^	I	A
Suitable as an initial option for evaluation of symptomatic patients with suspected stable CAD and low or intermediate pretest probability without known CAD.^3 , 8 , 23 , 37 , 38 , 133^	I	A
Evaluation of patients with suspected stable CAD and previous inconclusive or conflicting functional test results for ischemia.^2 , 3 , 8 , 25 , 127 – 129^	I	A
Evaluation of patients with suspected stable CAD and previous clinically discordant functional test results for ischemia.^2 , 3 , 8 , 25 , 127 – 129^	I	A
Option for the evaluation of patients with suspected stable CAD and intermediate pretest probability with an indication for invasive coronary angiography.^128 , 129^	I	A
Evaluation of asymptomatic patients with homozygous familial hypercholesterolemia.	IIa	B
Selective evaluation of asymptomatic patients with a high clinical risk score.^3 , 92 , 134^	IIb	C
Evaluation of patients with suspected stable CAD and high pretest probability.^2 , 20 , 25^	III	C
Routine evaluation of asymptomatic patients with a low or intermediate clinical risk score.^3 , 25 , 135^	III	B
Evaluation of asymptomatic patients in high-risk professions (eg, airline pilots) aged ≥ 40 years with an increased clinical risk score (≥ 10%).^136 , 137^	IIa	C

CAD: coronary artery disease.

### 2.3. In the Investigation of Ischemic Etiology of HF

In patients with ventricular dysfunction of unknown origin, it is necessary to exclude significant CAD, especially in those with risk factors and/or symptoms suggestive of coronary insufficiency.^138^Invasive coronary angiography is the reference method for the diagnosis of significant coronary obstruction in this clinical setting.^139 , 140^However, CCTA has proven to be very useful in this scenario, with high sensitivity and global negative predictive value for detecting luminal stenosis.^112 , 132^

In patients with HF, studies have demonstrated that CCTA has a sensitivity of 73%-98%, specificity of 99%-100%, positive predictive value of 92%-99%, and negative predictive value of 97%-100% for detection of obstructive CAD.^141 – 143^In an analysis of 96 patients, with a CAD prevalence of 46%, CCTA correctly identified 90% of patients with ischemic etiology and 97% of patients with nonobstructive coronary lesions.^142^Chow et al., in an international, multicenter, randomized trial, evaluated the cost-effectiveness of CCTA vs invasive coronary angiography in 246 patients with new-onset HF and found no statistically significant difference in clinical outcomes or costs between the 2 strategies.^144^

Therefore, the use of CCTA to help differentiate between ischemic and non-ischemic heart disease in patients with HF of unknown etiology is considered appropriate, especially in those with a low-to-intermediate pretest probability of obstructive CAD ( Table 4 ).^25 , 138 , 145^

**Table 4:** Recommendation for the use of coronary computed tomography angiography to assist in the etiological assessment of heart failure

Indication	Class of recommendation	Level of evidence
Evaluation of patients with heart failure as an aid in distinguishing between ischemic and non-ischemic heart disease.^25 , 101 , 145^	I	B

### 2.4. CCTA in Suspected Stable Angina with Known CAD

#### 2.4.1. Patients with Stents

The metal structures of the stent mesh can generate image artifacts on CCTA, hindering the analysis of the coronary lumen. These artifacts are more common in small-caliber stents (< 2.5 to 3.0 mm), in stents apposed to densely calcified plaques, and in stents with a mesh thicker than 100 μm.^146^For this reason, functional tests are preferably the first option in patients with stents and suspected obstructive CAD.^147^However, CCTA is not contraindicated in these patients and can be performed with good accuracy using specific image filters and optimized protocols, with special attention to heart rate control during imaging to minimize motion artifacts. A recent meta-analysis including 2,656 patients and using ≥ 64-slice MDCT scanners (4 cm or more coverage) analyzed 4,131 stents individually for the presence of potentially flow-limiting coronary lesions (stenosis ≥ 50%).^148^The data suggest that CCTA is accurate for the evaluation of stents, especially considering new generation CT scanners. Finally, functional data derived from stress CT-MPI may increase the diagnostic accuracy of CCTA in patients with stents.^149^

#### 2.4.2. Revascularized Patients

In a meta-analysis evaluating 2,482 grafts, the sensitivity and specificity of CCTA using 64-slice MDCT scanners for any stenosis > 50% were 0.98 (95% CI, 0.97-0.99) and 0.98 (95% CI, 0.96-0.98), respectively, with an AUC of 0.99. Neither patient age nor the interval between grafting and imaging had any effect on sensitivity or specificity for detecting significant stenosis or occlusion. There was also no difference in accuracy between arterial and venous grafts.^150^When evaluating grafts, these results can potentially improve with the use of more modern CT scanners.^151^Conversely, native coronary arteries can be difficult to assess given the presence of severe atheromatous disease, sometimes with a large amount of calcium deposits, reducing the specificity of the method.^152^

Therefore, if the clinical interest is to assess graft patency, CCTA is validated and appropriate.^3^If the interest is to assess native coronary arteries, CCTA may pose major limitations, and the use of functional testing should be considered. It should be noted the ability of CCTA to identify unprotected coronary territories and to estimate their extent. Given that a larger number of unprotected coronary territories is associated with a worse prognosis, the information provided by CCTA is of great relevance in the management of these patients.^153^

Table 5 presents the recommendations for the use of CCTA in patients with previous (percutaneous or surgical) revascularization.

**Table 5:** Recommendations for the use of coronary computed tomography angiography in the assessment of coronary artery disease in revascularized patients

Indication	Class of recommendation	Level of evidence
Evaluation of symptomatic patients with previous surgical revascularization, especially if graft patency is the primary objective.^2 , 3 , 25 , 150 , 154 , 155^	I	A
Evaluation of asymptomatic patients with previous surgical revascularization performed 5 or more years ago.^25 , 150 , 154 , 155^	IIb	B
Evaluation of symptomatic patients with previous angioplasty with stent(s), especially if the diameter of the stent(s) is ≥ 3 mm.^2 , 3 , 25 , 47 , 156 , 157^	IIa	B
Evaluation of asymptomatic patients undergoing angioplasty and stenting of the left main coronary artery, especially if the diameter of the stent(s) is ≥ 3 mm.^25 , 148 , 156 , 157^	IIa	B
Evaluation of asymptomatic patients with previous angioplasty and stenting performed 2 or more years ago, especially if the diameter of the stent(s) is ≥ 3 mm.^25 , 148 , 156 , 157^	IIb	B
Evaluation of symptomatic patients with a high clinical suspicion of angina and previous surgical or percutaneous revascularization.^2 , 20 , 25^	III	C

### 2.5. Follow-up of Patients with CAD Receiving Medical Therapy

CCTA is a noninvasive method to assess the arteries that nourish the myocardium which allows the analysis of vessel lumen and walls, and more recently also the study of atheroma characteristics that may eventually compromise these vessels.^158^Since the beginning of clinical application of this method, it has stood out for its high negative predictive power, proving to be effective in safely ruling out the presence of obstructive CAD.^132 , 159^However, as technology and experience with CCTA have increased, there has been an increase in positive predictive power, which can be even higher if noninvasive analysis of FFR_CT_is used, increasing its potential for clinical use.^158 , 160^In addition to the publications reporting its potential contributions, studies have emerged showing that CCTA can improve the evaluation of patients with suspected CAD, and, therefore, there is currently evidence to guide the use of this technique in this subgroup of patients, which is the topic covered in this section.^158 , 160^

In low-risk patients, CCTA can help manage the case, identify patients with coronary atheromatosis, even if not calcified, and define the atherosclerotic burden of noninvasive plaques, an element that grows in prognostic power, especially in cases in which there was no prior investigation to confirm the diagnosis of CAD.^38 , 121^Conversely, the absence of atheroma plaques represents an important good prognostic factor, and, even in the PROMISE study, the event rate in patients with nonobstructive coronary lesions was lower than in those with normal functional test results (adjusted odds ratio [OR], 0.38; 95% CI, 0.18-0.79; p = 0.01).

In cases of intermediate and intermediate-to-high risk, CCTA achieves its best results.^21 , 38^CCTA is very effective in determining the absence or presence of CAD, defining whether or not there is significant disease in the left main coronary artery, providing total atherosclerotic burden and non-calcified plaque burden, and enabling plaque analysis.^121 , 158 , 160 , 161^CCTA has high sensitivity, and its suitability to exclude the presence of obstructive disease in the epicardial coronary arteries is an important part of its indications. Likewise, when there is coronary atheromatosis on CCTA, intensive clinical management is indicated, as this will result in better prognosis and reduction of events. As a result, CCTA has assumed the role of initial imaging method to assess the presence or absence of obstructive lesions in patients at intermediate and intermediate-to-high pretest risk in clinical practice. Some studies have demonstrated that, in this scenario, CCTA is effective in studying the coronary artery anatomy, is cost-effective in relation to invasive coronary angiography, and has the same rate of late adverse events.^128^It is important to note that, even in cases where the diagnosis of ischemia is not confirmed, the presence of atherosclerosis is a stand-alone predictor of events, even when it is not considered significant.^38 , 160 , 161^The accuracy of CCTA has also been confirmed in more recent studies, and, for these reasons, it is now recommended for the initial evaluation of symptomatic patients in the United Kingdom.^160 , 162 , 163^Conversely, the presence of stenosis on CT alone cannot be used to indicate revascularization, as this strategy has not been shown to be effective in reducing events. However, it is important to analyze each case individually, considering, in addition to the presence or absence of atheroma plaques, clinical and functional elements and the possibility or impossibility of controlling symptoms with clinical treatment.^38 , 158 , 160 , 161^Some authors point out that the CCTA specificity is much lower than its sensitivity, which would therefore be a limitation of the method. If, on the one hand, some studies confirm this analysis, on the other hand, the combination with functional testing may allow the adoption of more appropriate approaches, and the introduction of FFR_CT_analysis into clinical practice may result in CCTA assuming an even greater role in the management of patients with stable angina.^158 , 160^CT-MPI can be performed and had its effectiveness confirmed in the CORE320 study, which demonstrated that the combination of anatomic and functional analysis using CT made it possible to safely manage patients with CAD, without increasing major risks for patients.^4^In practice, this approach is limited by requiring 2 contrast injections and by requiring a higher dose of ionizing radiation for image acquisition.^160^The analysis of FFR_CT_, in turn, has aroused great interest and allows, from a single acquisition, the evaluation of anatomy and functional data.^160^Its utility has been confirmed in randomized studies and in a recent meta-analysis, where its greatest practical limitation was the lack of widespread availability and high cost.^158 , 160 , 164^Protocols are being developed in an attempt to estimate FFR results using machine learning and artificial intelligence technologies, but they are not yet available for clinical use.

The publication of the Objective Randomised Blinded Investigation with optimal medical Therapy of Angioplasty in stable angina (ORBITA) trial and the ISCHEMIA trial requires us to make a particular analysis of the role of functional data obtained by any type of technology, including CT, and encourages their use in the initial clinical management of a large proportion of patients with stable angina. It is noteworthy, however, that the anatomic data provided by CCTA, especially the diagnosis or exclusion of left main coronary artery lesions, are essential both to define the need for clinical treatment and to identify which cases can benefit from interventional treatment. For this purpose, it is also reasonable to speculate that the functional data deriving from FFR_CT_analysis may also be very useful in identifying which patients may benefit from revascularization procedures.^158 , 160 , 161 , 165^

Among the data provided by CT in patients with stable angina, the analysis of atheroma plaque characteristics has been gaining popularity. Pioneering works have sparked interest in these characteristics, and subanalyses of the ISCHEMIA trial, SCOT-HEART trial, and the Coronary CT Angiography Evaluation for Clinical Outcomes: An International Multicenter Registry (CONFIRM) registry have demonstrated that the presence of non-calcified plaques, with signs of positive remodeling, heterogeneous attenuation, including the napkin-ring sign and areas of punctate calcification, imply a worse prognosis. Such results encourage the inclusion of these data into CT analysis and have led to the development of scores such as the CT-Leaman and Leiden scores, which incorporate this information alongside data such as lesion location and degree of obstruction (> 50% or < 50 %), which help determine the patient's risk.^160 , 161 , 166^However, total atherosclerotic burden deserves special attention, as, when elevated, it suggests a more serious prognosis even in patients without stenosis that reduces the vessel lumen by more than 50%.^121 , 158 , 160^Even in stable patients, this index has prognostic value and has been incorporated into some scores that are currently available in clinical practice.

Recently, the SYNTAX III REVOLUTION trial demonstrated that CCTA can also help to define which patients will be the best candidates for percutaneous or surgical treatment, suggesting that the inclusion of FFR_CT_analysis is essential for this purpose. If future studies confirm these findings, the role of CCTA can become even more important.^160 , 167^

Finally, if symptoms return after revascularization, whether with stent implantation or surgical treatment, CCTA can also be used to clarify symptoms. Its effectiveness is confirmed for the assessment of grafts and even native vessels in patients undergoing surgical treatment. When analyzing stents, the results are superior for stents greater than 3.0 mm in diameter not located in highly calcified arterial segments. However, CCTA can yield good results in selected cases in which high-quality images are obtained.^150 , 158 , 167^

### 2.6. CCTA in the Evaluation of Other CAD-related Scenarios

Although the primary role of CCTA is the direct workup of CAD, this modality allows the assessment of different parameters related to left ventricular (LV) function and pathologic involvement of the myocardium. In this context, it is important to highlight that, as a diagnostic modality that uses ionizing radiation and contrast agents with nephrotoxic potential, its use in assessments that can be performed by other methods (for example, assessment of LV function by echocardiography or assessment of myocardial viability by MRI) makes CT an exception resource, to be used in the unavailability of other methods or their limited image quality.

Table 6 presents the recommendations for using CCTA in other CAD-related scenarios.

**Table 6:** Recommendations for the use of coronary computed tomography angiography in the evaluation of other scenarios related to coronary artery disease

Indication	Class of recommendation	Level of evidence
Assessment of left ventricular function after acute myocardial infarction with inadequate or unreliable images by other noninvasive methods.^25 , 168^	I	B
Assessment of myocardial viability (late gadolinium enhancement) in patients scheduled for myocardial revascularization due to left ventricular systolic dysfunction who cannot undergo or have inadequate images by other noninvasive methods.^3 , 25 , 169 – 172^	IIb	B
Screening for graft vascular disease in patients with previous heart transplant as an alternative to coronary angiography.^3 , 25 , 173 , 174^	IIa	B

### 2.7. Coronary Artery Anomalies

Coronary artery anomalies (CAAs) are common congenital heart abnormalities,^175^often underdiagnosed and unknown by patients, mainly because most of them do not have clinical repercussions. However, it is known that some patients may experience sudden cardiac death, ischemic events, and HF resulting from some coronary artery variants.^176^

Therefore, imaging methods should not only confirm or rule out the diagnosis but also assess any associated risks.^176 , 177^

Goal-directed transthoracic echocardiography (TTE) can be used as an initial screening method, especially for anomalies of origin, as long as it is conducted in a goal-directed manner and by experienced operators.^176 , 178^

In the event of any abnormalities or in cases of high clinical suspicion, it is recommended to continue investigation with another noninvasive method that can assess the entire coronary vessel and possible repercussions, characterized here mainly by CCTA^179^and CMR.^175 – 184^Although there are not many studies directly comparing CMR and CCTA, the latter method is usually preferred for this assessment, as it offers better temporal and spatial resolution, especially in the case of higher heart rates (which are common in pediatric patients) and due to greater availability and experience with the method.

Several authors show a preference for CCTA as the gold standard for the assessment of CAAs in their studies. It is also known that the use of 3-dimensional (3D) reconstructions is useful in preoperative assessment and has been preferred by several surgeons and clinicians, making CCTA a more attractive method.

CCTA has also been demonstrated to indicate prognostic factors related to CAAs, assisting in clinical decision-making for interventions according to individual patient risk.^175 – 177 , 179 , 180 , 182 – 184^It is worth noting that MRI can provide important functional information, as well as characterize possible ischemic repercussions or repercussions on cardiac function, and can be used as a complementary modality.^175 , 176 , 184^

Table 7 presents CCTA recommendation for CAA investigation.

**Table 7:** Recommendation for the use of coronary computed tomography angiography in suspected coronary artery anomalies

Indication	Class of recommendation	Level of evidence
Evaluation of patients with suspected coronary artery anomalies.^2 , 3 , 25 , 185 – 187^	I	B

### 2.8. CCTA in Suspected Acute Chest Pain

Acute chest pain is one of the most frequent complaints in emergency care, accounting for up to 10% of non-trauma-related visits and up to 40% of causes of hospital admission. However, only 25% of these patients are diagnosed with acute CAD or another significant cardiac problem at the end of their hospital stay, leading to a large volume of unnecessary hospitalizations at a high cost.

To optimize emergency care, the use of CCTA is well established in the literature. As demonstrated in several studies, the method has excellent accuracy for the diagnosis of stenosis in patients with low-to-moderate cardiovascular risk, with emphasis on its high negative predictive value ( Table 8 ).^112 , 131 , 132 , 188^

**Table 8:** Summary of multicenter clinical trials on the accuracy of coronary computed tomography angiography in detecting coronary stenosis (> 50% luminal narrowing) in low-to-intermediate-risk patients without a previous diagnosis of coronary artery disease

Study	n	Sensitivity %	NPV %	Specificity %	PPV %
CATSCAN,^189^ 7 countries, 11 centers	187	94 (89-100)	98 (94-100)	51 (43-59)	28 (19-36)
NIMISCAD,^190^ 20 centers in Italy	327	94 (89-97)	91 (85-95)	88 (81-93)	91 (86-95)
ACCURACY,^131^ 16 centers in the United States	230	95 (85-99)	99 (96-100)	83 (76-88)	64 (53-75)
CORE^64 , 112^ 7 countries, 9 centers	291	85 (79-90)	83 (75-89)	90 (83-94)	91 (86-95)
Meijboom et al.,^132^3 centers in the Netherlands	360	99 (98-100)	97 (94-100)	64 (55-73)	86 (82-90)

NPV: negative predictive value; PPV: positive predictive value.

The use of CCTA in the assessment of acute chest pain has been safely evaluated in several studies regarding stratification, cost reduction, and reduction of in-hospital stay. Prospective randomized controlled trials have evaluated its use in the context of chest pain in the emergency department in low-to-intermediate-risk patients associated with the use of negative conventional troponin.^191^We highlight 3 studies.

The first is the multicenter Coronary Computed Tomographic Angiography for Systematic Triage of Acute Chest Pain Patients to Treatment (CT-STAT) trial, which randomized 699 patients with low-risk chest pain to stratification strategies using CCTA or stress-rest myocardial scintigraphy.^192^The CCTA strategy reduced the time to diagnosis by 54% and hospitalization costs by 38%, with no difference in the rate of adverse events compared with the scintigraphy strategy.

The second is the multicenter Angiography for Safe Discharge of Patients with Possible Acute Coronary Syndromes (ACRIN-PA) trial, whose primary objective was to evaluate the safety of using CCTA in the evaluation of low-to-intermediate-risk patients with chest pain (thrombolysis in myocardial infarction risk score of 0 to 2) compared with traditional care.^193^None of the patients with normal findings on CCTA showed the primary outcome (cardiac death or myocardial infarction within 30 days of admission). Additionally, patients in the CCTA group were more likely to be discharged from the emergency department (49.6% vs 22.7%) and had a shorter length of stay (18.0 vs 24.8 hours; p < 0.0001), with no difference in the rate of revascularization or catheterization.

The third is the Rule Out Myocardial Ischemia/Infarction Using Computer Assisted Tomography (ROMICAT II) trial, which evaluated, in similar groups of patients, length of stay in the emergency department and hospital costs.^194^The study included 1,000 patients, with a mean age of 54 years, and found that length of hospital stay was significantly shorter in the CCTA group than in the standard evaluation group (23.2 ± 37.0 vs 30.8 ± 28.0 hours; p = 0.0002). Time to exclusion of the diagnosis of acute coronary syndrome (ACS) was also shorter in the CCTA group (17.2 ± 24.6 vs 27.2 ± 19.5 hours; p < 0.0001). There were no differences between the groups regarding safety end points. In the CCTA group, there was a significant increase in the rate of patients discharged directly from the emergency department (46.7% vs 12.4%; p = 0.001). Overall costs were very similar between the 2 groups due to the shorter hospital stay (p = 0.65).

In summary, the use of CCTA is a safe strategy in the evaluation of low-to-intermediate-risk patients with acute chest pain (with non-diagnostic ECG and negative myocardial necrosis markers [non-high sensitivity]), reducing rates and length of hospital stay, and, probably, costs. In this scenario, the indication for the use of CCTA is classified as Class I, with Level of Evidence A.

The use of CCTA in the emergency department in patients with chest pain associated with conventional troponin elevation was evaluated in the Rapid Assessment of Potential Ischaemic Heart Disease with Computerised Tomography Coronary Angiography (RAPID-CTCA) trial,^195^which tested the strategy of using CCTA vs standard care in patients with a diagnosis of ACS without ST-segment elevation (with conventional troponin elevation). Data from this study show that the CCTA strategy did not reduce the proposed primary outcome (1-year all-cause mortality or type 1 or type 4b myocardial infarction at 1 year), with an incidence of 5.8% for CCTA vs 6.1% for standard care (p = 0.65) or revascularization (OR, 1.03; 95% CI, 0.87-1.21). However, it reduced the rate of catheterization (OR, 0.81; 95% CI, 0.72-0.92) at the expense of a slight increase in hospital stay (from 2.0 to 2.2 days).

The use of high-sensitivity troponin (hs-Tn) has gained ground in emergency departments as it provides safety for hospital discharge when negative. Few studies have evaluated the use of CCTA in this context. The multicenter, randomized Better Evaluation of Acute Chest Pain with Coronary Computed Tomography Angiography (BEACON) trial evaluated the use of CCTA in the emergency department in low-to-intermediate-risk patients who, after negative hs-Tn, were randomized to receive CCTA or standard optimal care in the emergency department, with the primary objective of evaluating the number of patients requiring revascularization within 30 days. The use of CCTA in this setting resulted in no difference in the number of patients requiring revascularization, incidence of undetected ACS, or rate of direct discharge from the emergency department (65% vs 59%; p = 0.16), with similar length of stay in both groups (6.3 hours). However, the use of CCTA reduced medical costs (€337 vs €511; p < 0.01) and outpatient testing after the index emergency department visit (4% vs 10%; p < 0.01).^196^

Therefore, CCTA applied early in the workup of suspected ACS in the emergency department is safe and associated with less outpatient testing and lower costs due to the reduced need for additional workup in the outpatient setting. However, in patients with negative hs-Tn, CCTA did not identify more patients with significant CAD requiring coronary revascularization, shorten hospital stay, or allow for more direct discharge from the emergency department compared with standard optimal care. The Troponin in Acute Chest Pain to Risk Stratify and Guide EffecTive Use of Computed Tomography Coronary Angiography (TARGET-CTCA) trial (NCT03952351) is underway to evaluate whether early treatment of CT-identified CAD in patients with intermediate hs-Tn elevation has an impact on the prevention of future events 36 months after discharge.

Some studies have evaluated the use of CCTA in the setting of elevated hs-Tn levels. The CARdiovascular Magnetic rEsoNance imaging and computed Tomography Angiography (CARMENTA) trial^197^demonstrated that the use of CCTA or CMR before catheterization is safe compared with routine care to select patients with positive hs-Tn for catheterization, without an increase in cardiovascular events. Therefore, the use of CCTA in patients with hs-Tn elevation to intermediate levels (study median of 78 ng/mL) and without high-risk indicators safely reduced the need for catheterization compared with routine care (OR 0.66; p < 0.001), with no increase in cardiovascular events after 1 year of follow-up (p = 0.265).

Studies have attempted to evaluate the use of CAC to predict coronary stenosis in the emergency department. A subanalysis of the CORE64 study^198^showed low negative predictive value (0.62), with up to 39% of high-risk patients with ACS having CAC = 0 and 46% having CAC < 100 Agatston units. Therefore, CAC in the emergency department to predict significant coronary lesions should not be used in light of current studies.

#### 2.8.1. Triple Rule-out (CAPTURE Study)

CCTA can also be used in the emergency department for the differential diagnosis of ACS. Using specific imaging protocols, information can be obtained on the coronary arteries, aorta, and pulmonary arteries, allowing the assessment of acute aortic syndrome (AAS) and pulmonary embolism (PE), in addition to the analysis of other thoracic abnormalities (eg, pneumonia, trauma).^199 – 204^This approach is called triple rule-out. The triple rule-out protocol includes in the analyzed field of view not only the coronary arteries but also the entire thoracic aorta and the pulmonary arteries, requiring a greater amount of iodinated contrast material, as well as an increase in the total radiation dose for imaging. However, even with optimized techniques, the imaging protocol for triple rule-out is less efficient than individual protocols for the assessment of the coronary arteries, aorta, and pulmonary arteries. Therefore, triple rule-out protocols should only be used in specific situations, in which clinical evaluation is unable to guide the diagnosis.

Table 9 presents the recommendations for the use of CCTA in the investigation of potential ACS.

**Table 9:** Recommendations for the use of coronary computed tomography angiography in suspected acute coronary syndrome (ACS)

Indication	Class of recommendation	Level of evidence
Evaluation of low-to-intermediate-risk patients with suspected ACS with normal or non-diagnostic ECG and normal or abnormal myocardial necrosis markers, but without definition of myocardial infarction * .^2 , 25 , 132 , 192 – 194 , 205^	I	A
Evaluation of patients with acute chest pain using the triple rule-out technique.^2 , 25 , 203 , 206^	IIb	B
Evaluation of high-risk patients with suspected ACS.^20^	III	C
Evaluation of patients with a definitive diagnosis of myocardial infarction.^20^	III	C

* Defined as a change at the upper limit of the assay reference (~99th percentile of the assay used) and/or changes in myocardial necrosis markers potentially explained by other concomitant condition(s). ECG: electrocardiogram.

### 2.9. CCTA in Preoperative Assessment

Preoperative assessment of cardiac and noncardiac surgery has well-established literature, with criteria defined by several medical societies.^207 – 210^The algorithm may include CCTA as one of the modalities that can provide additional information.^207^The presence and extent of CAD are the main information to be provided.

In cardiac surgery, several studies have demonstrated the potential of CCTA as an alternative to the gold standard (coronary angiography), highlighting its high negative predictive value.^160 , 163^Additionally, functional assessment (by FFR_CT_) has been incorporated into the anatomic study as a robust diagnostic improvement, increasing the potential information to be obtained from imaging. The Safety and Feasibility Evaluation of Planning and Execution of Surgical Revascularization Solely Based on Coronary CTA and FFRCT in Patients With Complex Coronary Artery Disease (FASTTRACK CABG) study, analyzed the results of the strategy and planning of myocardial revascularization based only on anatomy and functional assessment by CT. The primary safety outcome, defined as graft patency at 30 days, demonstrated anastomotic patency of 92.6%, with an incidence of major cardiovascular events of 7.2% and a major bleeding rate of 2.7%. These encouraging data expand the potential of coronary CT angiography as a sufficient tool for planning surgical myocardial revascularization.^211^The workup of CAD using CCTA in patients with an indication for valve surgery may be considered in cases with low or intermediate probability, while coronary angiography should be indicated in positive or doubtful cases.^212^

The low risk of CCTA associated with its high diagnostic accuracy compared with coronary angiography should not mean that its use is understood as the method of choice in the noninvasive stratification of candidates for noncardiac surgery. Therefore, there is currently no evidence to support the indication of its routine use in preoperative coronary assessment.^2 , 25^

Tables 10 and 11 present recommendations for the use of CCTA in the preoperative assessment of patients referred for cardiac and noncardiac surgery.

**Table 10:** Recommendations for the use of coronary computed tomography angiography in the preoperative assessment of noncardiac surgery

Indication	Class of recommendation	Level of evidence
Preoperative assessment of coronary arteries before arterial vascular surgery in patients with an estimated intermediate or high risk of complications.^2 , 3 , 25 , 207 , 213 – 215^	IIa	B
Preoperative assessment of coronary arteries before intermediate-risk surgery in patients with an estimated intermediate or high risk of complications and low functional capacity.^2 , 3 , 25 , 207 , 213 – 215^	IIb	C

**Table 11:** Recommendations for the use of coronary computed tomography angiography in the preoperative assessment of non-coronary cardiac surgery

Indication	Class of recommendation	Level of evidence
Preoperative assessment of coronary arteries before non-coronary cardiac surgery in patients with low or intermediate pretest probability of coronary artery disease.^2 , 3 , 25 , 216 – 218^	I	B

### 2.10. Valvular Heart Disease Assessment by CCTA

The initial assessment of valvular heart disease is performed using echocardiography, a widely available test that does not use ionizing radiation. However, in patients with inadequate or unreliable images using this method, targeted CCTA imaging may be an alternative to assess the morphology and function of heart valves and prosthetic valves, as well as cardiac chamber dimensions and associated ventricular functions.^25 , 168 , 219 – 225^

CCTA for the assessment of valvular heart disease should be directed toward clinical doubt and programmed differently for each heart valve with the aim of acquiring images with adequate contrast in the cardiac chambers of interest (right vs left) and adjusting the modulation of the radiation dose in the specific phases of the R-R interval to obtain diagnostic images for measurement of valve opening area (stenosis grading) and regurgitant orifice area (insufficiency grading). CCTA imaging is best performed and interpreted by specialists with experience in this setting.

Table 12 presents the recommendations for the use of CT in the assessment of heart valves.

**Table 12:** Recommendations for the use of computed tomography in the assessment of valvular heart disease

Indication	Class of recommendation	Level of evidence
Assessment of native valves in patients with suspected significant valve dysfunction with inadequate or unreliable images by other noninvasive methods.^25 , 219 – 224^	I	B
Assessment of prosthetic valves in patients with suspected significant valve dysfunction with inadequate or unreliable images by other noninvasive methods.^25 , 219 – 225^	I	B
Assessment of cardiac chamber dimensions and ventricular function associated with significant valvular dysfunction with inadequate or unreliable images by other noninvasive methods.^25 , 168^	I	B

### 2.11. Preoperative Assessment in Percutaneous Aortic Valve Implantation (TAVI/ViV)

Severe aortic stenosis affects 2.9% of older people aged 75 to 86 years.^226^Randomized clinical trials^227 – 229^have demonstrated that transcatheter aortic valve implantation (TAVI) in patients with severe aortic stenosis is feasible, safe, and associated with superior cardiovascular outcomes compared with surgical valve replacement in the high-surgical-risk population. In Brazil, TAVI has grown substantially in the number of implants per year,^230^reaching 1,400 implants in 2018.

The selection of patients who are candidates for TAVI, the choice of type (balloon or self-expanding) and size of the valve, and access should be evaluated by a team consisting of a clinical cardiologist, an interventional cardiologist, a cardiac surgeon, an echocardiographer, and a cardiac imaging specialist – the so-called "heart team." In this context, CT of the aortic valve complex, heart, total aortic arch, and iliac and common femoral arteries is essential for optimal decision-making.

The best image acquisition and reconstruction techniques, as well as the measures necessary for TAVI planning, have already been described in recommendations from international cardiovascular imaging societies.^9^Assessment of the aortic valve complex necessarily includes measurements of the aortic annulus (long and short axes, area, and perimeter) during systole, since these measurements vary throughout the cardiac cycle and valve size selection based on measurements during diastole may result in undersizing^231^and consequent paravalvular leak, which is related to higher mortality.^232^Valve size selection based on CT measurements is associated with a lower incidence of paravalvular leak than selection based on assessment by 2D echocardiography.^233^

The coronary artery ostia height is also essential, given the risk of obstruction by the device in patients with a left coronary artery height < 10 mm or a right coronary artery height < 12 mm in relation to the aortic annulus plane.^234^In patients who are candidates for valve-in-valve (ViV) implantation, CT can also anticipate the position of the new valve in relation to the coronary ostia, which are often closer to the annulus plane of the dysfunctional valve, and identify patients at increased risk of coronary occlusion.^235^

The extent of aortic valve calcification into the LV outflow tract (LVOT) is another important parameter in the evaluation of candidates for TAVI. Patients with significant calcification located in this topography have an increased risk of rupture during the procedure, a complication associated with a high mortality rate.^236^

Perimembranous ventricular septal defect < 8 mm is associated with an increased rate of advanced atrioventricular block and need for permanent pacemaker, and it can be easily assessed by CT. CT assessment should also include prediction of the best fluoroscopic projection angle during the procedure, potentially reducing the total contrast volume required for implantation.

The assessment of coronary anatomy in TAVI candidates is often hampered by several factors: clinical conditions that make apnea difficult, impossibility of controlling heart rate with beta-blockers, contraindication to the use of vasodilators, and severe vessel calcification. However, in studies with good image quality, the negative predictive value for detecting significant obstructive lesions remains high.^237^In this scenario, performing CCTA concomitantly with pre-TAVI planning by CT has a Class of Recommendation IIa, with Level of Evidence B ( Table 13 ).

**Table 13:** Recommendations for the use of computed tomography in planning percutaneous aortic valve implantation/replacement (TAVI/TAVR)

Indication	Class of recommendation	Level of evidence
In patients who are candidates for TAVI, CTA of the heart, thoracic and abdominal aorta, and iliac and common femoral arteries should be performed for procedure planning.^2 , 235 , 240 – 243^	I	A
In patients who are candidates for ViV TAVI, CTA of the heart, thoracic and abdominal aorta, and iliac and common femoral arteries should be performed for procedure planning.^2 , 9 , 235 , 244 – 246^	I	B
In patients with uncertain severity of aortic stenosis by echocardiography, cardiac CTA can be performed to assess morphology, valve function, and aortic stenosis severity.^25 , 219 – 223 , 225 , 247 – 250^	I	B
In patients with suspected low-flow, low-gradient severe aortic stenosis with preserved LVEF (≥ 50%) or reduced LVEF and no contractile reserve by dobutamine stress echocardiography, aortic valve calcium scoring may be performed to assess the possibility of severe aortic stenosis (≥ 1,300 AU for women and ≥ 2,000 AU for men).^212 , 251 – 253^	IIa	B
In patients with suspected valve leaflet thrombosis after TAVI, cardiac CTA can be performed.^9 , 254 , 255^	I	B
In selected patients who are candidates for TAVI, coronary CTA can be performed and interpreted if it shows adequate quality for diagnosis.^256 , 257^	IIa	B

CTA: computed tomography angiography; ViV: valve-in-valve; LVEF: left ventricular ejection fraction; AU: Agatston units.

Finally, CT also provides essential information for the evaluation and choice of access routes for implantation: characterization of the aorta, iliac arteries, and common femoral arteries regarding the presence of atheromatosis and degree of obstruction (if present), minimum luminal diameter, tortuosity, and presence of circumferential calcification. Peripheral artery disease is quite prevalent among TAVI candidates,^238^and CT assessment is superior to invasive assessment in predicting procedure-related vascular complications.^239^In patients in whom transfemoral access is not feasible, CT allows assessment of the LV apex for the presence of thrombi, calcification, and/or thinning that contraindicate the transapical approach. The ascending aorta should also be assessed for the presence, extent, and location of parietal calcifications for transaortic access.

Because CT is essential in TAVI planning, it should be used in candidates for the procedure. Table 13 presents the main recommendations for CT use in TAVI planning.

### 2.12. Percutaneous Planning of Other Structural Changes

Major advances in the percutaneous treatment of valvular heart diseases have made it possible to address valve diseases other than aortic stenosis, with a large number of prosthetic valves specific to each valve disease, some validated in clinical practice and with a lower procedure-related risk than corrective cardiac surgery.

In some scenarios, cardiac CTA combined with other CTAs is essential for optimal procedure planning and indication and can assist in valve size selection, in addition to predicting the risk of complications that may prevent the procedure.^258 – 267^ Table 14 presents the recommendations for the use of CT to support a percutaneous approach to other structural heart diseases.

**Table 14:** Recommendations for the use of cardiac computed tomography angiography (CTA) in planning percutaneous interventions for other structural heart diseases

Indication	Class of recommendation	Level of evidence
In patients who are candidates for transcatheter mitral valve replacement (valve-in-valve, valve-in-ring, or valve-in-MAC), cardiac CTA should be performed for procedure planning and assessment of the risk of complications.^258 – 261^	I	B
In patients who are candidates for percutaneous pulmonary valve implantation, CTA of the heart and pulmonary arteries can be performed for procedure planning as an alternative to magnetic resonance imaging, especially when there is a risk of coronary artery compression.^262 , 263^	I	B
In patients who are candidates for transcatheter tricuspid valve replacement (valve-in-valve or valve-in-ring), cardiac CTA should be performed for procedure planning.^264 – 267^	I	B
In patients who are candidates for percutaneous implantation of bicaval valve prosthesis for tricuspid insufficiency, CTA of the heart, thoracic veins, and upper abdominal veins should be performed for procedure planning.^267^	I	C

### 2.13. Assessment of Cardiac Veins, Left Atrium, and Pulmonary Veins (Including Planning for Atrial Fibrillation Ablation/Atrial Appendage Occlusion)

It is important to adequately characterize cardiac and vascular anatomy to assist both in the planning of electrophysiological procedures and in the control and monitoring of possible complications, especially in pulmonary vein ablation for the treatment of atrial fibrillation (AF). CTA is an excellent tool for determining vascular anatomy because it is a fast imaging method that provides images with a wide field of view, high spatial resolution, and 3D reconstruction.^25 , 268 , 269^

Correct identification of pulmonary vein and left atrial anatomy is critical to the safety and success of the AF ablation procedure. Pulmonary vein anatomy is marked by great variability among individuals regarding their number, ostia dimensions, and bifurcation pattern.^270^The most common variations are the presence of supernumerary pulmonary veins (18%-29%) and of a common trunk (> 30%), mainly on the left, in addition to the presence of a middle lobe pulmonary vein and a top vein.^271^CT allows the assessment of the number of pulmonary veins, their respective ostia and diameters, the presence of anatomical variations or drainage anomalies.^272 , 273^Measurements not only of the ostia diameter but also of their area and sphericity, pulmonary vein angle, and distance from the ostium to the first bifurcation can also be useful in planning the ablation when using the cryoablation technique.^274^CT can also be useful to anatomically define the vena cava, rule out atrial thrombus, and identify the location and course of the esophagus, as well as to identify the presence of the fossa ovalis and any abnormalities that may interfere with transseptal puncture, such as lipomatous hypertrophy of the interatrial septum. An anatomical definition should allow the optimal choice of ablation technique to be performed and most appropriate planning of the procedure, reducing procedure time and risk of complications.^271^

CT images can also be fused with electroanatomical mapping or fluoroscopic images.^275 , 276^Several studies have suggested that these techniques may reduce procedure time, AF recurrence rate, and radiation exposure.^271^However, evidence from the literature is still controversial regarding its real clinical benefit.^268 , 269^

Another use of CTA in this group of patients is related to post-ablation monitoring.^273 , 277^The 2 main complications reported are esophageal injury and pulmonary vein stenosis. CTA has high specificity for detecting pulmonary vein stenosis, which has a reported incidence of 0.29%.^278^CTA can identify the location, extent, and degree of pulmonary vein stenosis. It also allows comparison of findings with pre-procedure images. CT also allows assessment of the presence of pulmonary opacities suggestive of venous infarction or changes in adjacent mediastinal fat or lymphadenopathy.

CTA can also detect intracavitary thrombi, identified as low attenuation images located mainly in the left atrium (LA).^271 , 279^Recent studies have demonstrated great diagnostic value of the method for excluding atrial thrombus, especially when using late acquisition techniques (several seconds after contrast infusion), with a meta-analysis demonstrating diagnostic accuracy of 94% and negative predictive value of 99%.^280^Although transesophageal echocardiography (TEE) is the gold standard for detecting atrial thrombus and CTA is not yet routinely recommended for this purpose,^268^the method demonstrated potential practical application and utility, especially during the SARS-CoV-2 pandemic, when attempts were made to limit unnecessary invasive procedures.^281^

Furthermore, adequate morphological characterization and location of the LA are also important for atrial occlusion procedures, often indicated for patients who have contraindications to the use of anticoagulants. Multiple morphological patterns have been described: the "chicken wing" pattern is the most common (48%), followed by "cactus" (30%), "windsock" (19%), and "cauliflower" (3%), the latter being more closely associated with thromboembolic events.^278 , 282^Some important parameters for the implantation of the atrial occlusion device can be obtained by CT, such as ostial morphology and leaflet length and angle.^283^

#### 2.13.1. Technique

The CTA imaging technique for assessment of pulmonary veins varies according to the equipment, but, in general terms, it is similar to the technique used for CCTA imaging. A late confirmatory phase may be performed if there is an image suggestive of atrial appendage thrombus.

Table 15 presents the recommendations for the use of CTA in the assessment of pulmonary veins and left atrial appendage (LAA).

**Table 15:** Recommendations for the use of computed tomography angiography in the assessment of the left atrium, pulmonary veins, and cardiac veins

Indication	Class of recommendation	Level of evidence
Assessment of the left atrium, left atrial appendage, and pulmonary veins pre-ablation of atrial fibrillation.^2 , 25 , 268^	I	B
Assessment of the presence of thrombus in the left atrium/left atrial appendage in patients with atrial fibrillation as an alternative or in those with inconclusive transesophageal echocardiographic images.^2 , 3 , 25 , 268 , 269 , 280^	I	B
In patients who are candidates for percutaneous closure of the left atrial appendage for procedure planning.^268 , 269 , 284 – 287^	I	B
Assessment of cardiac vein anatomy before implantation of a cardiac resynchronization device.^2 , 25 , 288 , 289^	IIa	B

#### 2.14. Functional Assessment by CT

##### 2.14.1. CT Myocardial Perfusion Imaging

Assessment of myocardial ischemia with CT-MPI became possible a few years ago. This technique evaluates the first pass of iodinated contrast in the ventricular myocardium under the action of pharmacological vasodilatory stress. CT-MPI involves the use of 2 acquisitions: one is dedicated to the assessment of the coronary arteries (coronary CT itself), as well as to the assessment of myocardial perfusion at rest; the other is aimed at assessing myocardial perfusion, being performed under pharmacological stress (most commonly dipyridamole or adenosine in our setting).

The CT-MPI technique has been robustly validated in relation to its diagnostic performance, using as a reference myocardial perfusion scintigraphy,^4^CMR,^290^and invasive FFR.^291^The techniques used allow the assessment of stress perfusion from a single imaging of contrast administration (static imaging) or in sequential slices during the delivery of contrast to the myocardium (dynamic imaging). Both techniques are similarly accurate, differing from each other in that stress MPI allows quantitative assessments (estimation of regional and global myocardial blood flow, contrast upslope, and maximum attenuation peak), which can provide slight gains in terms of accuracy.^292 – 294^A third acquisition technique, using dual-energy imaging to obtain static images, can improve the accuracy of myocardial perfusion assessment,^295 , 296^given the improvements in the contrast-to-noise ratio between different tissues.

Scanners with 64 or more detector rows are required to perform this technique. The sequence in which the imaging steps are performed (stress-first or coronary CT-first protocol) prioritizes functional or anatomical assessment, respectively, and the choice depends on local experience. One factor that may determine the selection of a rest-first protocol (coronary CT first) is the possibility of canceling the stress test if coronary CT shows completely normal vessels, without stenosis.

##### 2.14.1.1. Diagnostic Accuracy

CT-MPI is a technique that should be used in conjunction with coronary CT imaging, providing anatomical and perfusion information in a single examination. In this respect, diagnostic accuracy is best assessed by considering the anatomical-perfusion alignment, aiming to detect stenosis associated with evidence of ischemia. The CORE320 study analyzed the combination of coronary CT with CT-MPI in the identification of flow-limiting stenosis, using the combination of myocardial scintigraphy and cardiac catheterization as a reference.^1^Considering all patients, the accuracy of the combined coronary CT and CT-MPI (measured by AUC) was 0.87, reaching 0.93 in patients without known CAD.

In an attempt to compare MPI with other functional methods, Takx et al. evaluated the diagnostic performance of several functional tests, using invasive FFR as a reference.^297^CT-MPI had an accuracy of 93% in detecting stenosis associated with the presence of myocardial ischemia, with performance comparable to that of positron emission tomography (PET) and CMR. Table 16 presents the diagnostic performance parameters of CT-MPI compared with other noninvasive methods.

**Table 16:** Diagnostic performance of computed tomography myocardial perfusion imaging compared with different diagnostic modalities *

Reference	Year	N	Reference	Sens.	Spec.	PPV	NPV
George et al.^298^	2012	50	MPS	72	91	81	85
Bettencourt et al.^290^	2013	101	FFR	89	83	80	90
Rochitte et al.^4^	2014	381	CC and MPS	80	74	65	86
Cury et al.^299^	2015	110	SPECT	90	84	36	99
Takx et al.^297^	2015	2.048	FFR	88	80	-	-
Sørgaard et al.^300^	2016	1.188	MPS, CMR, CC, FFR	85	81	-	-
Pontone et al.^301^	2019	100	CC and FFR	98	54	68	96

* Modified from Magalhães et al.^302^CC: cardiac catheterization; MPS: myocardial perfusion scintigraphy; Sens.: sensitivity; Spec.: specificity; FFR: fractional flow reserve; PPV: positive predictive value; NPV: negative predictive value; SPECT: single photon emission computed tomography; CMR: cardiac magnetic resonance.

##### 2.14.1.2. Prognostic Value

Although extrapolations from extensive literature are used regarding the prognostic value of ischemic burden identified by other functional methods (eg, myocardial perfusion scintigraphy and stress MRI), data are scarce on the use of CT-MPI and correlation with clinical outcomes. In a recent study, Dewey et al.^303^showed the 5-year follow-up of the CORE320 study and reported a comparable prognostic value of the combination of coronary CT and CT-MPI and the combination of myocardial perfusion scintigraphy + cardiac catheterization (AUC for prediction of major cardiovascular events was 0.65 for both approaches).

##### 2.14.1.3. Applicability in Different Clinical Settings

The possibility of combining assessment of coronary anatomy and functional repercussions of coronary stenosis in a single examination makes CT-MPI a very attractive tool in CAD workup. Specifically, a group of patients who benefit from this approach are those with known CAD and/or increased cardiovascular risk, as well as patients with stents.^149 , 304^In these settings, where there is a recognized loss of specificity of CTA due to limited luminal assessment, the use of CT-MPI can contribute with information that allows the identification of the impact of possible coronary stenosis on myocardial blood flow.

Furthermore, patients whose anatomy shows intermediate stenosis by CCTA, whose hemodynamic repercussion is doubtful, as well as those with stenosis whose extent of ischemia needs to be quantified may obtain useful information with the addition of CT-MPI to the workup.

##### 2.14.2. CT-derived Fractional Flow Reserve (FFR_CT_)

CCTA is very useful in patients with a low-to-intermediate probability of CAD, mainly due to its high negative prognostic value.^305 , 306^However, the specificity for detecting hemodynamically significant CAD is limited, especially with regard to moderate stenosis.^307^

Invasive FFR measurement is currently the gold standard for determining the hemodynamic significance of CAD.^308 – 310^With technological advances, it is now possible to measure FFR using CT.

FFR_CT_is a technology that uses the principles of fluid dynamics to generate a 3D model based on information derived from CCTA, without the need for additional contrast, radiation, or medication administration, since it is a post-processing analysis of the images provided by CCTA.^311^

Basically, there are 4 basic principles involved in the calculation of FFR_CT_based on fluid dynamics. The first is that microvascular resistance is inversely related to the diameter of the epicardial coronary arteries. The second is that the model has the ability to extract the myocardium from CCTA to determine the resting myocardial blood flow. The third is that maximal coronary hyperemia is predictable and can be calculated based on pre-established responses to adenosine. Finally, using the Navier-Stokes equations, blood flow and pressure along the coronary arteries can be determined.^312^

The first 3 published studies validating the method demonstrated good diagnostic performance compared with FFR,^313 – 315^using an FFR of 0.8 or less as positive, as shown in Table 17 .

**Table 17:** Diagnostic performance in the first 3 studies of FFRCT vs invasive FFR

Reference	Sens. %	Spec. %	PPV %	NPV %	Accuracy %
Discover-Flow^311^	93	82	85	91	88
DeFACTO^314^	90	54	67	84	73
NXT^315^	86	79	65	93	81

Sens.: sensitivity, Spec.: specificity, PPV: positive predictive value, NPV: negative predictive value; FFR: fractional flow reserve; FFR_CT_: computed tomography-derived FFR.

More recently, a meta-analysis including 1,852 patients and 2,731 vessels demonstrated FFR_CT_sensitivity and specificity, respectively, of 89% and 71% in the analysis per patient and 85% and 82% in the analysis per vessel.^164^The sensitivity of FFR_CT_showed no statistical difference from the sensitivity of CCTA. However, the specificity of FFR_CT_was significantly higher (71% vs 32%; p < 0.001), translated into the ability of the method to detect coronary stenosis that can determine blood flow restriction. Thus, the low specificity of CCTA is now overcome by a combined analysis with FFR_CT_, which can accurately diagnose the functional significance of coronary stenosis.

The RIPCORD study retrospectively evaluated decision-making in 200 patients from the NXT trial.^316^After disclosure of FFR_CT_data and analysis of the rate of significant stenosis, there was a change in the treatment plan in 36% of cases.

In the Prospective LongitudinAl Trial of FFRct: Outcome and Resource IMpacts (PLATFORM), which involved 584 patients with stable chest pain and intermediate probability of CAD divided into invasive and noninvasive arms, those evaluated with CCTA and FFR_CT_had lower rates of cardiac catheterization with non-significant CAD.^18^While 73% of patients in the invasive arm had cardiac catheterization with non-significant CAD, only 12% of those in the FFR_CT_group showed this outcome. It is worth noting that, in the noninvasive arm, 61% of patients had a planned invasive catheterization canceled, and in the 1-year follow-up analysis, there were no adverse events in these patients who had their invasive procedure canceled.^23^

In a substudy of the PROMISE study, involving 67% of the patients in this study evaluated by FFR_CT_, the availability of these data would lead to a significantly greater association with major adverse cardiovascular events (MACE) or revascularization compared with visual analysis by CTA. Furthermore, reserving the invasive strategy for patients with an FFR_CT_of 0.8 or less could decrease the nonobstructive CAD rate by 44% and increase the revascularization rate by 24%.^317^

In summary, these data consistently demonstrate that FFR_CT_significantly changes the diagnosis of obstructive CAD with blood flow restriction and, consequently, the management of approximately 25% of patients subjected to the technique. Furthermore, cancellation of cardiac catheterization in patients with a negative FFR_CT_(> 0.8), even in those with CAD on CCTA, has been shown to be a safe strategy.

Despite such evidence, FFR_CT_analysis, at the time of publication of this Guideline, remains off-site and limited to a single core laboratory. New artificial intelligence-based algorithms^318^allow on-site FFR_CT_analysis, but they remain unavailable for use in clinical practice.^319 – 323^

Regarding cost-effectiveness, studies that examine the economic impact of combining CCTA with FFR_CT_have projected promising results. Estimates based on the results of the Diagnosis of Ischemia-Causing Stenoses Obtained Via Noninvasive Fractional Flow Reserve (DISCOVER-FLOW) trial project a 30% reduction in costs over the course of 1 year.^324^In the United Kingdom, a retrospective analysis of the use of FFR_CT_in patients with stenosis of 10% to 90% estimated a saving of £200 per patient.^325^The PLATFORM trial included a prespecified economic evaluation that demonstrated a 1-year reduction in mean costs per patient from $12,145 to $8,127.^5^

Although the results favor the use of FFR_CT_, it is important to note that the success of this tool depends on a high-quality CCTA imaging (minimum amount of motion artifacts and good contrast-to-noise ratio).^326^Although the degree of calcification may impact the accuracy of FFR_CT_results, there is no pre-established value that prevents its evaluation.^327^Finally, because this is a lesion-specific analysis, its use is not recommended in settings of diffuse disease, in revascularized patients, or in patients with coronary stents.

Table 18 presents the recommendations for functional assessment by CT (CT-MPI and FFR_CT_).

**Table 18:** Recommendations for the use of computed tomography (CT) in the functional assessment of coronary artery disease (CAD)

Indication	Class of recommendation	Level of evidence
Assessment of myocardial ischemia by stress CT myocardial perfusion imaging as an alternative to other imaging tests for ischemia.^3 , 4 , 290 , 297 , 299 , 300 , 303^	I	A
Assessment of myocardial ischemia by stress CT myocardial perfusion imaging combined with coronary CT angiography in symptomatic patients with known CAD.^3 , 4 , 149 , 290 , 296 , 297 , 299 – 301 , 303 , 304^	IIa	A
Assessment of myocardial ischemia by stress CT myocardial perfusion imaging combined with coronary CT angiography in symptomatic patients without known CAD.^3 , 4 , 290 , 297 , 299 , 300 , 303^	IIb	B
Assessment of functional significance (ischemia) by FFR_CT_in patients with moderate stenosis in native vessels by coronary CT angiography.^3 , 5 – 8 , 167 , 315^	IIa	B
Assessment of functional significance (ischemia) by FFR_CT_in patients with left main stenosis ≥ 50%, significant 3-vessel stenosis, in-stent restenosis, or significant graft stenosis by coronary CT angiography.^3^	III	C

FFR_CT_: CT-derived fractional flow reserve.

#### 2.15. CT in the Assessment of Non-ischemic Cardiomyopathy

One of the main contributions of CT in patients with cardiomyopathy with reduced ejection fraction (EF) is the exclusion of ischemic etiology using CCTA. While there is a Class I recommendation for the use of invasive coronary angiography in the investigation of the etiology of HF with reduced EF in symptomatic patients,^141 – 143 , 328 – 332^other data demonstrate excellent correlation of CCTA with invasive coronary angiography in patients without known CAD and significant global systolic dysfunction with LVEF < 35%, showing a sensitivity of 98% (95% CI, 94%-99%), specificity of 97% (95% CI, 94%-98%), and AUC of 0.99 (p < 00001).^332^

Therefore, CCTA can be used as a primary noninvasive method to exclude ischemic etiology in patients with cardiomyopathy, with a level of evidence I, and is considered definitely appropriate.

#### 2.15.1. CT in the Assessment of Ventricular Function

The acquisition of images of the coronary arteries by CT, with a retrospective ECG-guided protocol, allows the reconstruction of images of the heart in several different phases of the cardiac cycle. This allows the assessment of cardiac chamber volumes at their respective maximum diastolic and systolic potential and, therefore, the measurement of systolic function and biventricular EF.

A meta-analysis of 12 studies,^333^comparing the assessment of systolic function by CT with MRI and transthoracic ECG as reference methods, showed excellent correlation between LV function assessment by CT and MRI with a bias of 0.0 (−3.7, 3.7 [SD 1.96], with 95% CI), as well as excellent agreement between left LV function assessment by CT and transthoracic ECG, with a bias of 0.3 (−4.7, 5.7 [SD 1.96], with 95% CI).

Therefore, LV function assessment by CT can be used as an alternative to ECG and/or MRI in non-ischemic cardiomyopathy in parallel with the exclusion of significant CAD, with a level of evidence I, and is considered definitely appropriate.

#### 2.15.2. Myocardial Tissue Characterization by Delayed Enhancement CT in Non-ischemic Cardiomyopathy

In patients with a contraindication to delayed enhancement MRI (fibrosis), CT can also be performed for this purpose with delayed image acquisition (7-12 minutes after contrast injection), without the need for a second contrast injection, but with a second exposure to CT scanner radiation (considering a first acquisition aimed at assessing the coronary arteries). However, recent optimization of CT scanners has resulted in low radiation doses per imaging session. This technique allows the assessment of myocardial fibrosis by CT with good correlation with delayed enhancement CMR in non-ischemic dilated cardiomyopathy (DCM),^334^cardiac sarcoidosis (CS),^335^myopericarditis,^336^and endomyocardial fibrosis (EMF).^337^

Assessment of myocardial fibrosis using delayed enhancement CT may be an alternative to MRI in non-ischemic cardiomyopathy. Table 21 shows the indications for the use of CT in the context of non-ischemic cardiomyopathies.

**Table 21:** Recommendations for the use of cardiac computed tomography angiography in the assessment of non-ischemic cardiomyopathy, pericardial diseases, and cardiac masses

Indication	Class of recommendation	Level of evidence
Assessment of left ventricular function in patients with heart failure with inadequate or unreliable images by other noninvasive methods.^25 , 168 , 359^	I	B
Quantitative assessment of right ventricular function as an alternative to cardiac magnetic resonance.^25 , 168 , 359 – 362^	I	B
Assessment of coronary arteries in heart failure to exclude obstructive CAD in patients with low or intermediate pretest probability.^2 , 3 , 25 , 359 , 362^	I	B
Assessment of right ventricular morphology and function in patients with suspected arrhythmogenic right ventricular cardiomyopathy as an alternative to cardiac magnetic resonance.^25 , 359 , 363 , 364^	I	B
Evaluation of patients with suspected hypertrophic cardiomyopathy with inadequate or unreliable images by other noninvasive methods.^359 , 365 – 367^	I	B
Evaluation of patients with suspected endomyocardial fibrosis with inadequate or unreliable images by other noninvasive methods.^337^	I	C
Evaluation of patients with suspected left ventricular non-compaction/excessive trabeculation of the left ventricle with inadequate or unreliable images by other noninvasive methods.^359 , 366 , 368^	I	C
Assessment of coronary arteries to exclude obstructive CAD in patients with suspected acute myocarditis and low or intermediate pretest probability of CAD.^359 , 366 , 369 – 371^	I	C
Assessment of coronary arteries in patients with suspected stress-induced cardiomyopathy to exclude obstructive CAD.^359 , 366 , 372 , 373^	IIb	C
Assessment of myocardial fibrosis (delayed enhancement CT) in patients with suspected non-ischemic cardiomyopathy who cannot undergo magnetic resonance imaging.^374^	IIb	B
Evaluation of patients with pericardial diseases with inadequate or unreliable images by other noninvasive methods.^25 , 340 , 375 , 376^	IIa	B
Assessment of cardiac masses (suspected tumor or thrombus) in patients who cannot undergo magnetic resonance imaging to complement the diagnosis in selected cases or in the presence of small masses.^25 , 346 , 377^	I	C

CAD: coronary artery disease; CT: computed tomography.

#### 2.15.3. Myocardial Extracellular Volume Measurement by CT

As with MRI, it is possible to measure myocardial extracellular volume (ECV) and estimate interstitial fibrosis by CT, with delayed image acquisition after contrast injection. Initial studies have demonstrated increased ECV in non-ischemic cardiomyopathy.^338^In this respect, there is the possibility of using CT-measured ECV to assist in the diagnosis of cardiomyopathies and storage diseases, as well as to guide treatment response.

### 2.16. CT in the Assessment of Pericardial Diseases

CT is a valuable complementary imaging modality in the assessment of the pericardium and should be considered in limited clinical scenarios with complex presentation or inconclusive echocardiographic findings.^339^

Prospective ECG-synchronized image acquisition, with low radiation and extending from the carina to the diaphragm, is generally adequate for the assessment of the pericardium.^339^ECG synchronization helps eliminate motion artifacts and, in the case of retrospective acquisition, can provide functional information, although at the cost of a higher radiation dose. However, it is not an absolute prerequisite, since reasonable images of the pericardium can be obtained even without ECG synchronization.

Normal pericardium appears as a thin linear hyperdense structure usually measuring less than 2 mm, which is easily detectable on both contrast-enhanced and non-contrast scans because of its visibility against the low attenuation of the surrounding fat.

If inflammatory, infectious, or neoplastic etiologies are considered, intravenous administration of iodinated contrast material is recommended to increase blood density and define possible pericardial inflammation.^339^

#### 2.16.1. Pericardial Effusion

CT may be useful in determining the presence of loculations, pericardial inflammation, or hemorrhage.^340^On CT, pericardial effusion can be characterized by measuring its attenuation value. Near-water attenuation (< 10 Hounsfield units [HU]) suggests a simple transudative effusion. It is unusual for a transudative effusion to exceed 15 HU, and this can be used as a threshold measurement to consider exudative effusion more likely than transudative effusion.^340^Attenuation values ranging from 20 to 60 HU suggest that the pericardial effusion may be purulent, malignant, or myxedematous. Effusions with attenuation > 60 HU may suggest hemorrhage.

#### 2.16.2. Acute Pericarditis

CT may demonstrate the presence of pericardial thickening or pericardial enhancement after contrast administration.

CT can be of great value especially in pericarditis of traumatic etiology (particularly when there is suspicion of associated lesions in adjacent structures), neoplastic diseases (assessment of disease extent and staging), and post-AMI (when doubt persists concerning the possibility of hemopericardium secondary to free wall rupture).

#### 2.16.3. Pericardial Tamponade

CT has no role in acute cardiac tamponade, due to patient instability, but it may help determine the feasibility of percutaneous pericardiocentesis, especially in loculated or complex effusions, when cardiac tamponade is subacute.^341 , 342^

CT signs of cardiac tamponade include a "flattened heart" and/or septal bounce due to compression of the cardiac chambers secondary to the presence of fluid, air, or masses. Indirect findings include dilation of the superior vena cava, with a caliber greater than or equal to that of the adjacent thoracic aorta, dilation of the inferior vena cava, with a caliber greater than twice that of the adjacent abdominal aorta, periportal edema, reflux of contrast into the inferior vena cava or azygos vein, and enlargement of the hepatic and renal veins.^340^

#### 2.16.4. Constrictive Pericarditis

CT is an excellent method for assessing pericardial thickness and therefore plays an important role in constrictive pericarditis. On CT, the normal pericardium is 1 to 2 mm thick, whereas in constrictive pericarditis it is usually 4 to 20 mm thick.^342 , 343^Increased pericardial thickness is a supportive sign in clinically suspected cases, but it does not confirm the condition, since constrictive pericarditis may occur without pericardial thickening and thickening may not determine constriction.^341^CT is the best method to delineate the presence and extent of pericardial calcification, a significant finding in view of clinical suspicion, although not pathognomonic.^344 , 345^

ECG-synchronized image acquisition with retrospective control allows the assessment of the physiology and possible chamber collapse. However, given the greater exposure to ionizing radiation, its indication remains restricted to patients with limited echocardiography and contraindications to CMR.^345^

#### 2.16.5. Pericardial Tumors

CT allows better characterization of pericardial tumors and adjacent structures, as well as the investigation of possible tumor dissemination and the presence of calcification or lymphadenopathies.^340 , 341^CT is more robust than CMR in the identification of other thoracic lesions, including primary lung cancer, lung metastases, and mediastinal nodules.^342^

On CT, features of pericardial malignancy include an irregular, thickened, nodular pericardium, complex pericardial effusion, and post-contrast pericardial enhancement. Rupture of the pericardial sac, presence of hemorrhagic effusion, invasion into epicardial adipose tissue, myocardium, or cardiac chambers, and mediastinal adenopathy are characteristics of an aggressive malignancy.^342^

Benign pericardial tumors include lipomas, which show low attenuation on CT,^340^and teratomas, which appear as fat- and calcium-containing masses.^342^

Pericardial mesothelioma is the most common primary malignancy of the pericardium and may appear as an effusion with pericardial nodules or plaques on CT or as a heterogeneously enhancing mass.^340^Lymphoma, sarcoma, and liposarcoma appear as large heterogeneous masses with associated pericardial effusion. Pericardial lymphomas appear as infiltrative enhancing masses.^340^

Angiosarcomas and synovial sarcomas are highly vascular tumors that often present with massive hemopericardium. On CT, these tumors appear as necrotic masses.^340^

#### 2.16.6. Cysts and Diverticula

Cysts, usually located in the right cardiophrenic angle, often appear on CT as oval, homogeneous masses with thin walls and a density of 30-40 HU. Given their liquid component, they do not show contrast enhancement.^341^On CT, a pericardial diverticulum resembles a cyst, although an open communication with the pericardial sac can be identified.^340^

#### 2.16.7. Congenital Absence of the Pericardium

CT often diagnoses congenital absence of the pericardium incidentally. Because of the natural contrast between the pericardium and epicardial fat rim, it is usually possible to delimit the pericardium on non-contrast CT, except in patients with minimal epicardial fat in whom this differentiation becomes difficult.^342^

In addition to the direct visualization of the pericardium, there are important indirect morphologic and functional signs consistent with pericardial defects. The diagnosis is suspected when posterior wall motion is accentuated or when the right ventricle (RV) appears falsely enlarged due to leftward shift. The marked displacement of the apex toward the axilla leads to the appearance of compressed atria. Interposition of lung tissue in spaces between the aorta and pulmonary artery or between the base of the heart and the diaphragm is a specific sign.

#### 2.16.8. Pneumopericardium

Pneumopericardium is an accumulation of air within the pericardial space, which usually occurs in the setting of traumatic pericardial injury. Other etiologies include positive pressure ventilation and cardiothoracic surgery.^340^

CT is very useful in acute conditions and will show pneumopericardium as an accumulation of air in the pericardial space, and can also assess its hemodynamic repercussions when acquired retrospectively with ECG synchronization.^340^

#### 2.16.9. Pericardial Foreign Bodies

Pericardial foreign body injury may occur due to direct trauma or secondary to distal embolic penetration. CT is usually diagnostic because it localizes the foreign body in the pericardium and may also show associated features, such as hemopericardium.^340^ Table 19 presents the diagnostic performance of CT and MRI in pericardial abnormalities.

**Table 19:** Comparison of CT and CMR in pericardial diseases

	CT	CMR
**Technical aspects**		
Availability	++	+
Cost	moderate	high
Scan duration	10 minutes	30-40 minutes
Safety	+ a	++ b
Patient access and monitoring	++	+/-
**Pericardium**		
Pericardial thickening	+++	+++
Pericardial calcification	+++	-
Pericardial inflammation	++	+++
Pericardial adhesions/leaflet mobility	+	+++
Effusion detection	+++	+++
Effusion characterization	++	++
Pericardial masses	+/++	++/+++
**Pericardial morphology**		
Including tissue characterization	++	+++
**Cardiac function**		
Systolic	++ c	+++
Diastolic	-	++
Septal movement/coupling	+/-	+++
Intracardiac abnormalities with breathing	+/-	++

CT: computed tomography; CMR: cardiac magnetic resonance. (-): not possible or poor assessment; (+): moderate; (++) good; (+++) excellent.

a Ionizing radiation, potential nephrotoxicity due to contrast agent, allergic reactions to contrast agent.

b Patients with metal implants, claustrophobia, potential nephrotoxicity due to contrast agent, allergic reactions to contrast agent, restricted to hemodynamically stable patients only.

c Using electrocardiogram-synchronized image acquisition.

### 2.17. CT in the Assessment of Cardiac Masses/Thrombi

Assessment of cardiac tumors often requires the use of multiple imaging modalities to obtain accurate information about location and tissue characteristics. TTE is usually the first choice due to its availability and high temporal resolution, being ideal for identifying small mobile masses. TEE can more accurately assess valve masses that are not well visualized on TTE.^346^

CMR is the imaging modality of choice for a more detailed assessment of nonvalvular cardiac tumors, as it provides excellent tissue characterization and multiplanar reconstruction of cardiac structures. However, CMR may not be suitable for some patients due to restrictions such as prolonged acquisition time, contraindication in cases of claustrophobia, uncooperative patients, or in the presence of certain implanted devices.^347^

In these scenarios where other imaging modalities are not diagnostic or are contraindicated, CT has become an increasingly used method for this assessment. Advantages include shorter acquisition time and high spatial resolution.^348^ECG synchronization minimizes motion-related artifacts and allows the identification of tumor location and a more precise delimitation of lesion margins and their relationship with surrounding tissue planes and structures, which is especially valuable for surgical planning.^349^

CT-based diagnostic assessment of masses involves several aspects, including mass size, location (cardiac chamber, pericardial involvement, extracardiac structures), quantity, morphology (fixation, margin appearance, infiltration), and clinical correlation (known malignancy or infection, presence of catheter, associated syndromes). Furthermore, CT has the ability to characterize tissues via density and perfusion analysis, being useful in the etiological differential diagnosis by assessing calcification, fat attenuation, vascular distribution, and fibrous component of tumors. Compared to other cardiac imaging modalities, CT stands out as the optimal option for assessing calcified masses, also providing a comprehensive assessment of the thorax, lung tissue, and their corresponding vascular structures ( Table 20 ).^346^

**Table 20:** Characteristics of cardiac masses on CT

MASS / TUMOR	CARDIAC CT FINDINGS
BENIGN 75%	
Myxoma	Pedunculated, mobile, heterogeneous with low attenuation. 10%-20% are calcified. Mitral valve prolapse may occur.
Lipoma	Well defined, encapsulated, and hypodense with fat attenuation. Homogeneous and nonenhancing; multiple lesions may be seen with tuberous sclerosis.
Fibroelastoma	Difficult visualization on CT. Small homogeneous mass attached to the heart valve (10 mm) by means of a small, mobile pedicle. Thrombus formation may occur on the surface.
Rhabdomyoma	Multiple lesions in 60% of cases. Homogeneous attenuation similar to the myocardium. > 90% in infants and children.
Fibroma	Homogeneous and intramural, with low attenuation and minimal enhancement, often showing central calcification; second most common in infants and children.
Hemangioma	Well defined; density is low or equal to the myocardium; marked heterogeneous enhancement; "vascular flushing."
Teratoma	Polycystic, moderate enhancement, partially calcified.
**MALIGNANT 25%**	
Angiosarcoma	Irregular, heterogeneous, low attenuation, infiltrative, pericardial effusion, metastatic.
Rhabdomyosarcoma	Irregular, low attenuation, infiltrative; extends significantly into the myocardium and pericardium; associated with a poor prognosis. Common in infants and children.
Fibrosarcoma	Large, irregular, low attenuation, with an extensive area of central necrosis or hemorrhage, infiltrative.
Osteosarcoma	Low attenuation, infiltrative with extensive calcification.
Liposarcoma	Large, adipose and soft tissue attenuation, slight contrast enhancement, infiltrative.
Mesothelioma	Infiltrative, variable attenuation, pericardial effusion.

CT: computed tomography.

A CT also plays a crucial role in tumor staging, as it has the ability to detect metastases in cases of suspected malignancy, especially when combined with 18F-fluorodeoxyglucose PET/CT.^350 – 352^

It can also help differentiate between intracavitary tumors and thrombi.^353^It is an accurate and reliable alternative to TEE for the detection of thrombi in the LA and LAA in patients with AF, with mean sensitivity and specificity of 96% and 92%, respectively.^10^Likewise, in patients with ischemic stroke, CT sensitivity and specificity for detecting LA/LAA thrombi are 96% and 100%, respectively.^354^

CT characterizes LV thrombi as hypodense masses, with significantly lower attenuation compared with the adjacent myocardium, and diagnoses them with a sensitivity of 94%, specificity of 97%, positive predictive value of 94%, and negative predictive value of 97%.^353^Currently, there are few validated data on the role of CT in the detection of LV thrombi compared with CMR,^355^but in the setting of ischemic stroke, CT may be superior to TTE.^356^

CCTA, obtained with the same acquisition protocol, allows preoperative assessment of the presence of CAD or adjacent masses that may determine obstruction, reducing procedure-related risks.^357^Noninvasive stratification is particularly indicated for patients with a low pretest probability, especially those with masses in the left cardiac chambers who are at increased risk of embolic events associated with invasive coronary angiography.^357 , 358^

Table 20 presents the characteristics of the main cardiac masses seen on CT.

Despite its recognized utility, CT has some limitations, such as radiation exposure, risk of contrast-related adverse events, lower temporal resolution compared with echocardiography or CMR, and lower soft tissue resolution compared with CMR.^346^ Table 21 presents the recommendations for the use of CT in the assessment of non-ischemic cardiomyopathy, pericardial diseases, and cardiac masses/thrombi.

### 2.18. Vascular Diseases

Arterial and venous territories are assessed using CT, which is a practical and precise examination with extremely high diagnostic accuracy. Improvements in CT scanners, with an increase in the number and profile of detectors, have brought advances in spatial and temporal resolution, allowing imaging to be performed more quickly and effectively. Such characteristics are essential, for example, in the evaluation of critically ill patients, where imaging time becomes a determining factor.

Different extracardiac vascular diseases will be discussed below. This Guideline will not address the diagnosis of intracranial vascular diseases.

#### 2.18.1. Aorta

Diseases of the aorta are diverse and include aortic aneurysm (AA), AAS, pseudoaneurysms, aortic rupture, atherosclerotic and inflammatory conditions, genetic diseases, and congenital abnormalities. AAS includes aortic dissection (AD), intramural hematoma (IMH), penetrating ulcer, and traumatic aortic injury.

Aortic diseases may be diagnosed after a long period of subclinical development or may have an acute presentation. AAS is often the first sign of the disease, which requires rapid diagnosis and decision-making to reduce the extremely poor prognosis. Treatment outcomes for stable, often asymptomatic, but high-risk conditions are much better than the outcomes of treatment required for acute disease.

Therefore, the identification of aortic diseases before a possible acute phase is desirable, and in this context, diagnostic imaging plays a very important role, especially CT and MRI.

CT plays a central role in the diagnosis, risk stratification, and treatment of aortic diseases. Its advantages over other imaging modalities include the short time required for image acquisition and processing, the ability to obtain a complete (3D) volumetric dataset of the entire aorta, a wide field of view, and broad availability.

In the assessment of the thoracic aorta, the acquisition of ECG-synchronized images is essential to eliminate motion artifacts from the aortic root and ascending aorta, avoiding testing-related diagnostic errors.^378^

To obtain volumetric images that allow 3D multiplanar reconstruction, which is essential for assessing the aorta, it is necessary to use at least 16-slice MDCT scanners, preferably with 64 or more detector rows, as these devices have higher spatial and temporal resolution.^379^When assessing the thoracic aorta, given the need for ECG synchronization, it is recommended to use at least 64-slice MDCT scanners, as faster acquisition is less susceptible to cardiac and respiratory motion artifacts. A slice thickness of 1 mm or less is recommended.

Intravenous administration of iodinated contrast material is required to obtain angiographic images by CTA, with image acquisition at arterial peak contrast enhancement. In acute and postoperative conditions, it is recommended to obtain unenhanced images before contrast injection (pre-contrast phase) to assist in the detection of hematoma and extravasation. A late post-contrast acquisition is recommended in postoperative control imaging, especially endovascular, to detect extravasation and to assess possible parietal enhancement.

CT allows the detection with excellent accuracy of the location and extent of the diseased aortic segment, vessel diameters, presence of atheroma, thrombus, dilation, stenosis, hematoma, ulceration, dissection, parietal thickening, calcification, and extravasation. It allows the assessment of periaortic tissue, aortic branches, and extravascular structures, making it possible to detect changes in target organs such as hypoperfusion and infarction. Furthermore, the branches of the aortic arch, the iliac arteries, and the femoral arteries, which are essential for planning surgical and endovascular repair procedures, can be easily included in the scanning area.

In acute aortic disease, MDCT is the recommended imaging modality in the initial assessment. Several studies have demonstrated high diagnostic accuracy for the detection of AD and IMH (combined sensitivity of 100% and combined specificity of 98%), as well as for the detection of penetrating ulcer, thrombus, occlusion, pseudoaneurysm, and rupture.^380^

Due to the use of ionizing radiation, it is recommended that children and young women should not be monitored for aortic diseases exclusively with CT, and other methods that do not require ionizing radiation can be used, such as MRI, ultrasound, and ECG, depending on the location of the affected aortic segment.

The use of iodinated contrast may be a limitation in patients with iodine allergy and in those with compromised renal function.^381^It is worth noting that non-contrast CT can be used to monitor aortic dilation, since the external vessel caliber can be measured without contrast administration, with the same precision as that with contrast administration.

#### 2.18.2. Extracranial Carotid Arteries

Carotid CTA is an effective imaging modality for assessing atherosclerotic involvement of this vascular territory, and especially for defining the resulting degrees of obstruction, providing support for the planning of treatment/intervention of extravascular diseases.

Carotid stenosis is defined by stenosis > 50% (symptomatic or asymptomatic) in the extracranial segment of the internal carotid artery, and is one of the main clinical indications for imaging screening for extracranial arterial disease.^382^Patients with carotid stenosis ≥ 60% to 99% on ultrasound are referred for carotid CTA or MR angiography to confirm the degree of stenosis and to assess plaque characteristics. This recommendation is supported by the lower accuracy of ultrasound for quantifying the degree of stenosis and the possibility of false positives using that method.^383^

Carotid CTA can be used in the assessment of dissection, which is a recognized cause of ischemic stroke and transient ischemic attack.^384^Carotid artery dissection may be spontaneous, occurring occasionally in the presence of predisposing factors, but spontaneous extracranial carotid artery dissection may also occur in patients without predisposing factors in association with trauma.^384^

Head or neck trauma presents a moderate-to-high risk of associated vascular injuries that require CTA assessment, especially trauma associated with fractures of the first to third cervical vertebrae, fractures extending into the transverse foramina, and skull base fractures. Other indications for carotid CTA include fibromuscular dysplasia of the carotid or vertebral arteries, CAD, assessment of aneurysms and pseudoaneurysms, vascular malformations and arteriovenous fistulas, planning of endovascular treatment or vascular surgery, assessment of tumor vascularization in the cervical or craniocervical region, vasculitis, and collagen diseases.

#### 2.18.3. Renal Arteries

CTA is very useful in the assessment of renal artery stenosis and has advantages over digital subtraction arteriography due to its ease of operation and less invasiveness.^385^In a prospective study comparing CTA vs digital subtraction arteriography, the sensitivity, specificity, and accuracy values for detecting significant stenosis were 100%, 98.6%, and 96.9%, respectively.^386^In addition to atherosclerosis, CT can assess the involvement of the renal arteries by other diseases, such as fibromuscular dysplasia, polyarteritis nodosa, arteriovenous fistula, aneurysm, and thrombosis.^387^

#### 2.18.4. Peripheral Vascular Disease

Approximately 80% of lower extremity artery diseases are represented by peripheral arterial occlusive disease (PAOD), with an estimated prevalence of 14.5% in individuals over 70 years of age. The clinical presentation may range from asymptomatic patients or patients with intermittent claudication to cases of acute arterial occlusion.

The remaining 20% of lower extremity arterial diseases, which also often cause stenosis and occlusion, include systemic diseases and local inflammatory and degenerative processes. This group includes aneurysms, vasculitis (such as thromboangiitis obliterans, fibromuscular dysplasia, and Takayasu arteritis), and popliteal entrapment syndrome.

Within this context, imaging of the lower extremity arteries has the role of confirming a diagnostic hypothesis (such as stenosis and/or occlusion due to PAOD) and displaying the anatomy and abnormalities to determine the need and feasibility of an invasive procedure, to choose the optimal strategy, and to prepare for an endovascular or surgical procedure.

CTA and MR angiography allow obtaining high-resolution images of lower extremity arteries with a relatively shorter turnaround time, which improves patient tolerance.^388^The performance of MR angiography in the assessment of PAOD is very similar to that of CTA, with a sensitivity of 92% to 99.5% and specificity of 64% to 99%.^388 , 389^Due to its availability, ease of operation, and reduced imaging time, CTA is the noninvasive imaging modality of choice for vascular assessment, with MR angiography being reserved for patients who cannot undergo CTA, such as those allergic to iodine and those with renal failure.

Both CTA and MR angiography allow complete mapping of the lower extremity arteries, from the abdominal aorta to the foot arteries. They ensure the identification of the number of lesions, stenosis extent, diameter, and morphology, adjacent normal arterial caliber, and status of the distal vessels.^389^CTA also shows calcifications.^388 , 390^This information guides the procedure planning regarding access route, choice of material, and expected long-term permeability after the intervention.^388 , 390^

Endografts and stents are easily identified on CTA by their metal mesh, which shows high attenuation.^391^Their patency and potential luminal stenosis are also very well characterized, since this method allows visualization of the intraluminal contrast column.^391^Endografts and stents, in general, cause image distortion artifacts on MRI and MR angiography, which prevents the evaluation of their lumen and, in some cases, of the surrounding structures.

Peripheral artery aneurysms appear as focal fusiform or saccular arterial dilations, often associated with mural thrombi.^392^In the angiographic evaluation, whether by CT or MRI, it is essential to assess aneurysm diameters and extent, the diameters of the affected vessel above and below the dilation,^392^as well as aneurysm neck measurements^392^and precise location, with description of possible involvement of other vessels,^392^to choose the optimal approach.

#### 2.18.5. Pulmonary Arteries

Several diseases can involve the pulmonary arteries and result in parietal abnormalities, dilation, stenosis, and occlusion. The 2 main pulmonary vascular diseases are PE and pulmonary hypertension (PH).

CT plays a key role in the diagnosis of pulmonary artery diseases, especially PE. Its advantages over other imaging modalities include the short time required for image acquisition and processing, the ability to obtain a complete (3D) volumetric dataset of the entire thorax, a wide field of view, and broad availability. To assess the pulmonary arteries, it is recommended to use at least 16-slice MDCT scanners, preferably with 64 or more detector rows, as these devices have higher spatial and temporal resolution, thus increasing image quality and reducing the occurrence of cardiac and respiratory motion artifacts. A slice thickness of 1 mm or less is recommended.

CTA of the pulmonary arteries is the method of choice for assessing PE. It allows adequate visualization of the pulmonary arteries and identification of thrombi down to the subsegmental level. It has good sensitivity and specificity for the diagnosis of PE,^393^and a negative CTA is considered an adequate criterion for excluding PE in patients with low or intermediate pretest probability.^394^

Regarding PH, CTA can identify cardiac and extracardiac abnormalities usually related to PH, such as dilated pulmonary trunk, signs of previous PE, and RV dilation or hypertrophy, among others.^381 , 395^

#### 2.18.6. Visceral Arteries

Contrast-enhanced CTA is effective in assessing significant arterial and venous stenosis in cases of acute and chronic mesenteric ischemia, as well as in providing additional information on intestinal loops, including data to assess mesenteric ischemia due to strangulated loops in internal hernia, adhesions or bands, and non-atherosclerotic diseases, such as arteritis and fibromuscular dysplasia.^396 – 398^

In acute mesenteric ischemia, contrast-enhanced CTA is the imaging modality of choice because there is a need for rapid diagnosis. In addition, other modalities such as MR angiography, for example, discussed in the MRI section of this Guideline, may not be available or may require more patient cooperation for acquisition of the sequences. A careful systematic review and meta-analysis calculated a sensitivity of 94% and a specificity of 95% for the diagnosis of acute mesenteric ischemia using contrast-enhanced CTA.^397^

In chronic mesenteric ischemia, also called abdominal angina, contrast-enhanced CTA can identify both old stenoses and potential collateral vascular networks that may appear after significant stenosis/chronic occlusion of the main mesenteric vessels (celiac trunk, superior mesenteric artery, and inferior mesenteric artery).^399^

MDCT can assess the main mesenteric vessels with an accuracy similar to that of arteriography. Furthermore, MDCT also has high accuracy for postoperative evaluation of angioplasty or grafting.^390 , 400^

Table 22 presents the recommendations for the use of CTA in the evaluation of different clinical scenarios related to vascular diseases.

**Table 22:** Recommendations for the use of computed tomography angiography in the assessment of vascular diseases

Indication	Class of recommendation	Level of evidence
Assessment of pulmonary embolism.^2 , 393^	I	A
Assessment of aortic aneurysms.^2 , 179 , 401 – 403^	I	B
Assessment of chronic aortic dissection.^2 , 403^	I	B
Assessment of acute aortic syndrome (dissection, ulcer, hematoma, and rupture).^2 , 401 , 404 – 406^	I	B
Assessment of traumatic aortic injury.^2 , 401 , 406^	I	B
Planning of surgical approach to the aorta.^2 , 401^	I	B
Planning of endovascular approach to the aorta.^2 , 401^	I	B
Assessment after aortic endografing.^2 , 401 , 407^	I	B
Assessment of the carotid and vertebral arteries.^2 , 408^	I	B
Assessment of the celiac trunk and mesenteric arteries.^2 , 400 , 409^	I	B
Assessment of the renal arteries.^2 , 84 , 409^	I	B
Assessment of the upper and lower extremity arteries.^2 , 409 – 412^	I	B
Assessment of medium- and large-vessel arteritis.^2 , 385 , 400 , 401 , 407 – 412^	I	B
Central vein assessment.^2 , 413 , 414^	I	B
Peripheral vein assessment (extremities).^2 , 414 – 417^	IIa	B

### 3. Cardiovascular Magnetic Resonance

CMR is one of the most complete and comprehensive examinations in cardiology. Although it has existed for decades in this field, its use and indications have grown exponentially after the development of myocardial delayed enhancement technique in 1999, being currently considered an essential imaging test for the optimal care of patients with heart disease.^2 , 418 – 420^The studies conducted since then have changed the understanding of several diseases within cardiology, whether in CAD through the more sensitive detection of myocardial infarction and a new concept of myocardial viability by infarct transmurality, or through the assessment of non-ischemic cardiomyopathy, with knowledge of diseases that were previously not properly diagnosed. In addition, in the assessment of congenital heart disease, CMR increased the accuracy of volumetric measurements and ventricular functions, helping to improve patient outcomes during follow-up.^2 , 12 – 15 , 421 – 423^

CMR does not use ionizing radiation (unlike CT, nuclear medicine, and hemodynamics), offering safety for patients who need follow-up imaging or younger patients. When the use of contrast is required, gadolinium-based contrast agents are used, which do not present nephrotoxicity. However, they should be used with caution in patients with chronic renal failure and glomerular filtration rate < 30 mL/min due to the risk of nephrogenic systemic fibrosis. Despite this recommendation, recent consensus statements have shown the safety of specific contrast agents in this population.^2 , 424^

Limitations to the use of CMR are claustrophobia, which can be overcome by performing the examination under anesthesia in patients in whom the benefits of the information outweigh the risks of the procedure, and the presence of metal devices such as ferromagnetic cerebral aneurysm clips and cochlear implants.^2^The presence of cardiac implantable electronic devices, such as pacemakers and implantable cardioverter-defibrillators (ICDs), is not currently a contraindication to MRI. However, such devices require programming and monitoring by specialists during imaging and will present metal artifacts in the chest, sometimes limiting the appropriate analysis of imaging findings, particularly in patients with ICDs.^2 , 425^

A major advantage of this method is its multimodality features, allowing for the collection of various data on a heart condition in a single examination, thus providing a more assertive diagnosis through various pulse sequences with different objectives.^2^

A routinely used technique is cine-MRI using the steady-state free precession (SSFP) pulse sequence, which allows accurate and highly reproducible measurement of cardiac chamber volumes and ventricular masses and functions, without the need for contrast agents, and is the best method for patients who need this information for clinical decision-making. Cine-MRI sequences also allow an excellent morphological and functional evaluation of the heart without the limitation of the patient's biotype and without problems of inadequate windows, thus allowing the assessment of localized hypertrophies, trabeculations, aneurysms/pseudoaneurysms, pericardial diseases, masses, and valvular heart diseases and planimetric measurements to determine the severity of valve stenosis.

Another CMR pulse sequence is fast spin echo, capable of producing static images of the heart without the use of contrast agents, which can be T1-weighted for morphological evaluation and fat determination, or T2-weighted for the assessment of edema.^2^These sequences can be used in the assessment of pericardial masses and diseases, myocardial and pericardial edema, fatty infiltration in arrhythmogenic cardiomyopathy, and lipomatous metaplasia in chronic myocardial infarction, among others.

A specific MRI analysis that changed the follow-up, treatment, and mortality of thalassemia major was the diagnosis of myocardial iron overload through changes in the T2-star (T2*) value.^2 , 426 , 427^This measurement is performed using a gradient-echo pulse sequence, without the use of contrast agents, and assists in the diagnosis of cardiac hemosiderosis, a complication of hereditary hemolytic anemia that requires multiple transfusions, such as thalassemia major and hereditary hemochromatosis.

Recent techniques within CMR include T1 and T2 parametric mapping, increasingly used in clinical practice, but which are not yet available on all MRI scanners in our country.^428 , 429^T1 mapping allows the detection and quantification of changes in the myocardial structure without the use of contrast agents, such as the presence of myocardial fibrosis, but it may have limitations in determining the etiology when not combined with the delayed enhancement technique. The T1 map also appears altered in conditions of inflammation, such as AMI, acute myocarditis, and pericarditis, and in cases of suspected storage diseases, such as cardiac amyloidosis (CA), being considered essential in the diagnosis by MRI. T2 mapping allows for a more objective and accurate assessment of myocardial edema, assisting in the diagnosis of acute heart diseases, such as myocarditis.

Phase-contrast pulse sequences are widely used in patients with valvular heart disease and congenital heart disease, as they allow blood flow analysis with the measurement of volumes and associated velocities, without the use of contrast agents.^2^Phase-contrast pulse sequences can provide highly accurate measurements of regurgitant volume and regurgitant fraction in the grading of valve insufficiency, or of the pulmonary-to-systemic flow ratio (Qp/Qs) in patients with intracardiac or extracardiac shunts.

Another important CMR technique is first-pass MPI of gadolinium-based contrast agents, which can be used in tissue characterization of cardiac masses or, particularly, in stress CT-MPI for the assessment of ischemia. Stress MRI is usually performed with vasodilators, such as adenosine and dipyridamole, and has high diagnostic accuracy. In the Clinical Evaluation of MAgnetic Resonance imaging in Coronary heart disease (CE-MARC) study, which compared CMR and scintigraphy for the assessment of ischemia in 628 patients undergoing coronary angiography, CMR showed higher sensitivity (CMR 86.5% vs SPECT 66.5%) and higher negative predictive value (CMR 90.5% vs SPECT 79.1%) than scintigraphy, with similar specificity and positive predictive value.^10^Another way to detect myocardial ischemia with CMR is the analysis of segmental contractility under dobutamine stress, which also presents high diagnostic accuracy. A study comparing CMR with dobutamine stress echocardiography found higher sensitivity (CMR 86.2% vs ECHO 74.3%), higher specificity (CMR 85.7% vs ECHO 69.8%), and higher accuracy (CMR 86.0% vs ECHO 72.7%) for CMR in detecting significant coronary stenosis compared with coronary angiography.^430^

The delayed enhancement technique, first demonstrated in patients with myocardial infarction and later in different non-ischemic cardiomyopathies, had a great impact on cardiology and the use of CMR. This technique allows the detection of areas of myocardial necrosis after contrast injection, discriminating between myocardial infarction and non-ischemic fibrosis. Furthermore, it allows predicting myocardial viability based on infarct transmurality, as well as stratifying the risk of adverse events in multiple heart diseases.^12 – 15 , 418 , 421 , 422^Ischemic and non-ischemic fibrosis can be differentiated in a simple way by the involvement of the subendocardium and by obeying one or more coronary territories in fibrosis following myocardial infarction. Figure 4 provides a graphical representation of the different patterns of myocardial fibrosis and their correlation with the diagnosis of ischemic and non-ischemic cardiomyopathy.^14 , 15^In view of these features, CMR is considered an indispensable tool for the initial evaluation of patients with cardiomyopathy of unknown etiology ( Table 23 ).

**Figure 4:** Patterns of myocardial fibrosis identified by magnetic resonance imaging and correlation with different cardiomyopathies.

**Table 23:** Recommendation for the use of cardiac magnetic resonance in the assessment of cardiomyopathy

Indication	Class of recommendation	Level of evidence
Initial evaluation of patients with cardiomyopathy.^2 , 418 – 420 , 431 – 434^	I	B

In congenital heart diseases, another technique that can be added to CMR is contrast-enhanced MR angiography (MR angiography of the thoracic aorta and pulmonary arteries) aiming at a comprehensive approach to these heart diseases in the assessment of associated vascular lesions, which impact the choice of optimal treatment and the success of surgical and/or hemodynamic interventions.^2^MR angiography of vascular structures can also be ordered alone in specific conditions, for example: MR angiography of the thoracic aorta in patients followed up for ascending AA to assess the timing of a possible surgical indication.^2^

Therefore, given the numerous techniques and data that can be assessed by MRI, it is recommended that CMR be guided (using specific protocols) by the hypotheses raised by the ordering physician, providing imaging services and the physicians responsible for image acquisition and analysis with the necessary data for a better outcome for the benefit of patients.

### 3.1. Use of Multiparametric Mapping in the Differential Diagnosis of Cardiomyopathies

In the last decade, the use of multiparametric mapping has grown significantly and has become a useful tool in the differential diagnosis of cardiomyopathies. This technique allows the assessment of pathological processes in the myocardium, being useful in the investigation of myocardial edema, iron deposition, and presence of interstitial fibrosis and areas of infarction, thus playing a relevant role in the evaluation of treatment response and providing prognostic information. These maps provide information based on changes in myocardial parameters of T1, T2, and ECV.

#### 3.1.1. T1 Mapping Technique

T1 mapping is defined as the myocardial (T1) relaxation time for recovery of longitudinal magnetization. The longitudinal or spin-lattice relaxation time is a measure that evaluates how fast the proton magnetization returns to its equilibrium state after a radiofrequency (RF) pulse in the MRI scanner. The T1 value is encoded in each pixel by this sequence.

T1 mapping is considered a tool capable of characterizing the different structures present in the heart,^435^accurately distinguishing the myocardium, areas of fibrosis, and presence of edema. It has the potential to detect diffuse structural changes in the myocardium that cannot be assessed by other noninvasive methods, including LGE. Terms commonly used in T1 mapping include native T1 (images without paramagnetic contrast agents), post-contrast T1 (images acquired after contrast injection), and ECV (values derived after contrast administration), among others. Native T1 and ECV are the variables with the greatest impact on daily clinical practice.

#### 3.1.2. Basic Principles

The basic principle of T1 mapping is the acquisition of a sequence of multiple T1-weighted images and nonlinear curve fitting, using the signal intensity and time after inversion of each image.^428 , 436^T1 times can be determined for regions of interest, myocardial segments, or at each pixel location to form a T1 map.

ECV is obtained through a mathematical equation, which considers the pre-contrast and post-contrast T1 times and hematocrit level. Hematocrit levels should preferably be measured on the same day as imaging.

Several techniques can quantify myocardial T1 relaxation times, each with specific advantages and limitations, such as modified Look-Locker sequence (MOLLI), short MOLLI (shMOLLI), and saturation recovery single-shot acquisition (SASHA). To compare T1 values, it is important that the protocol used is the same, that is, T1 values should be obtained with the same type and dose of contrast agent at the same field strength (1.5 T vs 3.0 T) using the same post-processing method.

T2 mapping is a tool that has proven to be sensitive in the assessment of myocardial inflammation and reversible myocardial injury, allowing the assessment of acute/active inflammation without gadolinium enhancement.

The identification of edema by T2 mapping was superior to traditional T2-weighted dark blood imaging, with T2 mapping being important in the diagnosis of inflammatory cardiomyopathy and elevated in the acute phases of AMI.^437 – 439^

#### 3.1.3. Differential Diagnosis of Cardiomyopathies

T1 and T2 mapping can assist in the differential diagnosis of cardiomyopathies because these maps present different behaviors in each disease. T2 maps are very useful in identifying edema, while T1 maps have the ability to identify edema, diffuse fibrosis, and myocardial infiltration ( Table 24 ).

**Table 24:** T1 mapping and extracellular volume (ECV) patterns most commonly found in cardiomyopathy

Cardiomyopathy	T1	ECV	LGE
Myocarditis^440 – 443^	Elevated acute phase. Diffuse involvement.	Elevated	Midmyocardial, epicardial, coronary LGE pattern.
Takotsubo syndrome^444^	Elevated in areas of abnormal contractility.	Elevated in areas of abnormal contractility.	Absent
Myocardial infarction^445 , 446^	Segmental abnormalities in the infarcted territory. More pronounced elevations in the acute phase.	Highly elevated in areas of fibrosis.	Transmural/subendocardial LGE pattern. Corresponding to coronary territory.
Cardiac amyloidosis	Significant increase in T1 values, diffuse.	Marked extracellular infiltration. Important increase in ECV values.	Diffuse/subendocardial/transmural LGE.
Anderson-Fabry disease^447^	Diffusely reduced.	-	Classically, inferolateral mid-wall non-coronary pattern.
Iron overload cardiomyopathy^448^	Diffusely reduced.	Decreased	Absent
Hypertrophic cardiomyopathy^449 , 450^	Elevated	Elevated	Midmyocardial, non-coronary LGE pattern. Multifocal.
Dilated cardiomyopathy^449^	Elevated	Elevated	Absent or midmyocardial, focal, commonly located in the IVS, inferior wall, or epicardium.

LGE: late gadolinium enhancement; IVS: interventricular septum.

T1 values are prolonged in cases where the extracellular compartment is increased, being elevated especially when there is edema (eg, increased tissue water in inflammation following AMI) and increased interstitial space (eg, scar fibrosis following myocardial infarction/cardiomyopathy and amyloid deposition). Conversely, T1 values are reduced when there is lipid overload (eg, Anderson-Fabry disease) and iron overload. ECV increases with excessive collagen deposition and decreases in lipomatous metaplasia. Table 24 summarizes the main characteristics of T1 mapping in cardiomyopathy.

#### 3.1.4. Myocardial Infarction/Ischemic Cardiomyopathy

In patients in the acute phase of infarction, native T1 values are elevated due to the presence of myocardial edema.^428^Elevated segmental native T1 values, when comparing LGE areas, showed significant correlation especially in patients with chronic myocardial infarction, being suitable for classifying segments with no LGE, LGE positive but viable, and LGE positive but non-viable.^451^ECV values are elevated in segments of myocardial fibrosis.^452^Reduced native T1 values and pseudonormalization are observed in areas of intramyocardial hemorrhage and no-reflow, respectively.^445^

#### 3.1.5. Myocarditis

Myocarditis is diagnosed by CMR using the Lake Louise criteria,^437^which are based on the identification of necrosis, edema, and scarring/inflammation. Recent studies have shown that the use of T1 and T2 mapping increases diagnostic accuracy compared with classically used techniques.^453^Native T1 mapping can assess myocardial injury and edema more sensitively than the early gadolinium enhancement technique, and native T2 mapping is used to assess edema instead of the classically used T2-weighted imaging techniques.^438 , 440^Non-coronary, midmyocardial, and epicardial LGE patterns are usually found. Native T1 and ECV values are usually increased, characterizing diffuse myocardial fibrosis.^441^Furthermore, SARS-CoV-2 cardiac involvement is relatively common. Affected patients have significantly elevated native T1 and T2 values, and these elevations correlate with troponin levels.^454^However, the findings are indistinguishable from those of myocarditis of other etiologies.

#### 3.1.6. Takotsubo Syndrome

Characterized by a transient reduction in EF, with abnormal segmental contractility, most commonly related to the apical region of the LV. Patients with Takotsubo syndrome showed elevated native T1 and T2 values and ECV, more pronounced in segments with abnormal contractility. At late follow-up, the patients had a progressive reduction in these values associated with the recovery of ventricular function.^444^

#### 3.1.7. Cardiac Amyloidosis

Amyloid protein deposition in the myocardium is associated with increased myocardial thickness and diffuse myocardial fibrosis, with the presence of subendocardial and transmural LGE distributed throughout the ventricular wall. Both types of CA have markedly elevated native T1 values, but transthyretin-related CA usually shows more extensive myocardial involvement than light-chain CA.^455^CA is associated with a higher ECV than any other cardiomyopathy secondary to extensive and substantial extracellular infiltration of amyloid protein deposition.^452^Recent studies have shown that T1 mapping (native T1 and ECV values), performed serially, can be a noninvasive tool to monitor treatment response and changes in myocardial structure.^456^In patients with renal dysfunction secondary to amyloidosis, markedly elevated native T1 values suggest the diagnosis of CA, without the need for gadolinium administration.^457 , 458^

#### 3.1.8. Anderson-Fabry Disease

Anderson-Fabry disease is a rare condition characterized by intracellular lipid deposition resulting in ventricular hypertrophy. In this condition, affected patients had globally reduced native T1 values compared with healthy volunteers and patients with other comorbidities.^447^T1 values were inversely proportional to wall thickness and were altered even in patients without ventricular hypertrophy, indicating that this technique is an early marker of cardiac involvement.^459^In areas with the presence of LGE, T1 values were normal or elevated.^447^

#### 3.1.9. Iron Overload Cardiomyopathy

Iron overload cardiomyopathy is a manifestation of hemochromatosis. Cardiac involvement due to myocardial iron deposition is diagnosed using the T2* technique. Patients with myocardial iron overload (T2* < 20 ms) have reduced native T1 and T2 values.^460 – 462^Native T1 and T2 values were significantly correlated with T2* values and showed lower intra- and inter-observer variability.^462^T1 mapping can be considered an alternative method for quantifying cardiac iron, with the potential for detecting mild iron overload, with high reproducibility. These characteristics have potential implications for clinical trial design and treatment monitoring.^448^

#### 3.1.10. Hypertrophic Cardiomyopathy

Hypertrophic cardiomyopathy (HCM) is a genetic disease characterized by LV hypertrophy (LVH). Native T1 values are increased in HCM and correlate with the degree of myocardial thickness. Post-contrast T1 values are reduced in the presence of diffuse interstitial fibrosis outside the areas of LGE.^449 , 463 , 464^ECV in HCM is above normal values even in areas without LGE.^452^Prolonged native T1 values and elevated ECV may be present even in patients without LGE and outflow tract obstruction, suggesting diffuse myocardial fibrosis, and are related to the degree of LVH, being an early marker of disease.^465^In a cohort of 263 patients with HCM, elevated ECV values during a mean follow-up of 28 months were associated with the occurrence of cardiac events (death, heart transplant, HF, resuscitated cardiac arrest, and cardiac arrest after syncope).^450^In the differential diagnosis between HCM and athlete's heart, native T1 and ECV values were characteristically normal or reduced in adaptive hypertrophy in athletes.^428^

#### 3.1.11. DCM

In patients with DCM, native T1 and ECV values are increased and post-contrast T1 values are reduced compared with healthy controls.^449 , 466^ECV values reflect the myocardial collagen content in DCM and can serve as a noninvasive tool to monitor treatment response and to assist in risk stratification at different stages of the disease.^467^A recent meta-analysis of 1,242 patients showed that ECV and native T1 had high prognostic value for a composite outcome of mortality and morbidity, with HR 1.38 (95% CI, 1.18-1.61) and HR 1.20 (95% CI, 1.14-1.27), respectively.^468^These findings suggest that native T1 and ECV values may help identify potentially severe patients who will develop major cardiovascular complications.^468^

Other conditions can change myocardial mapping, such as chemotherapy-induced cardiotoxicity, valvular cardiomyopathy (classically, aortic stenosis), heart transplant, and systemic diseases with cardiac involvement (eg, systemic lupus erythematosus, rheumatoid arthritis, and systemic sclerosis).^428^Diagnostic investigation with mapping in these scenarios assists in the early identification of disease activity, which is normally not visualized by other methods in the acute phase. Table 25 presents the recommendations for the use of multiparametric mapping in CMR.

**Table 25:** Recommendations for the use multiparametric mapping in the assessment of cardiomyopathy

Indication	Class of recommendation	Level of evidence
Use of T1 mapping in the diagnosis of cardiac amyloidosis.^469 , 470^	I	A
Use of T1 mapping in the treatment response of patients with cardiac amyloidosis.^436^	IIa	B
Use of T2* in the quantitative assessment of myocardial iron deposition.^471 – 474^	I	A
Use of T1 mapping in the assessment of myocardial iron deposition.^475^	IIb	C
Use of T1 mapping in the diagnosis of Anderson-Fabry disease.^476 – 478^	I	B
Use of T2 mapping in the investigation of myocardial edema in inflammatory cardiomyopathy.^437 , 439 , 479^	IIa	B
Use of T1 mapping in the prognostic evaluation of patients with dilated cardiomyopathy.^480 – 483^	IIa	B
Use of T1 mapping in the evaluation of patients with hypertrophic cardiomyopathy.^442 , 443 , 465 , 484^	IIb	B

### 3.2. Investigation of CAD Using MRI – Myocardial Ischemia

The last 3 decades have shown a steady increase in the use of CMR for the diagnostic and prognostic assessment of ischemic heart disease.

Stress CMR presents several technological advantages that result in superior diagnostic accuracy compared with other commonly used methods, such as myocardial scintigraphy and ECG stress test. CMR provides a wider field of view, higher spatial resolution, and consequently greater ability to differentiate between multiple tissues. Furthermore, the high spatial resolution of CMR allows the identification of perfusion defects in several subendocardial layers. Perfusion defects are identified through the first pass of gadolinium-based contrast agents, which makes the technique less susceptible to loss of accuracy in the setting of balanced ischemia.

CMR can assess multiple parameters of ischemic heart disease, such as detection of ischemia, presence of fibrosis/necrosis following myocardial infarction, determination of myocardial viability, and more recently, myocardial T1 and T2 parametric mapping and ECV.

CMR has high accuracy and reproducibility for the analysis of global and segmental biventricular function, regardless of ventricular geometry and patient biotype. In the evaluation of patients with sequelae of AMI, such as aneurysms and pseudoaneurysms, CMR can accurately identify cardiac chamber volumes and geometry (in addition to the infarcted area), being important in the evaluation of improvements in cardiac function and ventricular anatomy after myocardial revascularization procedures.^485 , 486^Therefore, CMR is an appropriate method for assessing global and segmental ventricular contractility and function, and is currently considered the gold standard for this purpose.^487 , 488^

#### 3.2.1. Detection of Myocardial Ischemia

Currently, the presence of myocardial ischemia can be detected by first-pass stress-rest MPI or by assessing contractility through dobutamine-induced myocardial ischemia, where the former has higher sensitivity and the latter has higher specificity.^489 – 491^

#### 3.2.2. Assessment of Myocardial Perfusion

Currently, MPI is the most common method to assess ischemic heart disease. MPI is usually performed in 2 phases (stress and rest), with gadolinium infusion to define hypoperfused areas. Infusion of vasodilators, such as dipyridamole and adenosine, induces significant hyperemia of the coronary microcirculation not associated with significant epicardial stenosis. However, the same does not occur in territories supplied by coronary arteries with significant stenosis, which already present maximal compensatory vasodilation (coronary reserve mechanism). This difference in perfusion between ischemic and remote territories allows the identification of myocardial perfusion defects, providing important information for patient management and prognosis.^492^

Myocardial perfusion is often assessed visually or, less frequently, by semi-quantitative or quantitative analysis (requiring specific software). CMR MPI assesses ischemia by comparing stress images vs rest images, which are obtained after reversal of dipyridamole stress by aminophylline. LGE images should be analyzed together when reading myocardial perfusion images because areas of severe ischemia (obstruction > 90%) may have perfusion deficits in both the stress and rest phases. However, as myocardial fibrosis can be visualized directly by the LGE technique, the rest phase is not mandatory.

The assessment of ischemia using CMR MPI has high diagnostic accuracy and was compared with other diagnostic methods in the early 2000s. Single-center studies have demonstrated that CMR is superior to scintigraphy, with values similar to those of PET.^493^The multicenter MR-IMPACT study, published in 2008, showed high diagnostic power of CMR MPI to detect ischemia.^494^

Following the evolution of knowledge, meta-analyses have been published. Nandalur et al.,^495^in a meta-analysis of 1,183 patients, reported that CMR had a mean sensitivity of 91% and specificity of 81% to detect obstructive CAD. In 2010, Hamon et al.^496^published a study showing high sensitivity (89%) and specificity (80%) in the diagnosis of obstructive CAD.

Recent studies with larger sample sizes have been published and demonstrated the great utility of stress CMR in daily cardiological practice. Studies such as MR-IMPACT II, published in 2013, demonstrated the superiority of the method compared with myocardial scintigraphy.^494 , 497^The CE-MARC study, published in 2012,^10^evaluated 752 patients and compared CMR with angiography as the gold standard for defining stenosis > 70% or left main stenosis > 50%, with sensitivity and specificity of 87% and 83% respectively.

The prognostic value of CMR in ischemic heart disease presents consistent data. The Magnetic Resonance Imaging for Myocardial Perfusion Assessment in Coronary Artery Disease (MR-INFORM) trial evaluated 918 symptomatic patients and compared different investigation strategies, demonstrating that the CMR strategy is not inferior to the invasive FFR strategy (3.7% for FFR vs 3.6% for CMR), with no difference at 12 months for primary outcomes.^498^The Stress CMR Perfusion Imaging in the United States (SPINS) study evaluated 2,349 patients with chest pain and showed that the absence of ischemia or LGE was associated with a low incidence of cardiovascular events within 5 years of CMR.^499^

The assessment of ischemia by MRI is strongly supported by studies conducted in multiple clinical scenarios comparing it with different methods to detect ischemic heart disease. Furthermore, in the last decade, several studies have evaluated the prognostic factor of the presence/absence of perfusion deficits, proven to be a useful tool for risk stratification. Also, several international guidelines highlight the importance of this method in the assessment of ischemic heart disease.

#### 3.2.3. Assessment of Segmental Contractility/Contractile Reserve

Dobutamine stress MRI is the most widely used method for ischemia by assessing segmental contractility or contractile reserve, given the technical difficulties involved in performing physical exercise inside the MRI scanner.

Therefore, with increasing doses of dobutamine, myocardial ischemia during the stress test can be defined as a new segmental contractile deficit resulting from dobutamine infusion or the occurrence of a biphasic response, that is, increased myocardial contractility at low doses and segmental dysfunction at high doses of dobutamine.^430^

The advantages of dobutamine stress CMR over ECG are due to the high image quality and, consequently, high reproducibility of results, since acoustic window problems do not occur with CMR.^500^

The protocol to assess ischemic heart disease with dobutamine stress CMR follows the same protocol used in stress echocardiography, with increasing doses of the drug (10, 20, 30, and 40 mcg/kg for 3 minutes), with or without the addition of atropine after the 40 mcg/kg dose, with the aim of reaching the patient's submaximal heart rate.^501^The complication rate in CMR is low, less than 0.1%, similar to that of stress echocardiography.^502^The image acquisition protocol involves sequences for dynamic assessment of function (cine) in different cutting planes, covering the 17 myocardial segments.^501^Qualitative analysis is commonly performed in clinical practice, but quantitative assessment techniques (eg, tagging) have been used in clinical studies, demonstrating that they facilitate the identification of ischemia both qualitatively and quantitatively.

The first dobutamine stress CMR studies were published in the 1990s and showed the high accuracy of the method for diagnosing coronary obstructions ≥ 50%, with a sensitivity of 81% to 84%.^503^A meta-analysis conducted by Nadalur et al. demonstrated high sensitivity (83%) and specificity (86%) for the diagnosis of significant CAD in high-risk patients.^495^

Dobutamine stress CMR, as well as CMR MPI with dipyridamole stress, is important in the prognostic evaluation of patients. A normal dobutamine stress CMR indicates a low event rate (< 2% at 2 years).^504^The presence of segmental dysfunction identifies patients at risk for AMI and death from cardiac causes.^505^Ischemia diagnosed by motility abnormalities in dobutamine stress CMR is an independent predictor of cardiac events (HR 5.42 at 3 years; p < 0.001), regardless of patient sex.^506 , 507^

However, dobutamine stress CMR has limitations, such as adequate patient monitoring during imaging (since the magnetic field can alter the ECG tracing, making it impossible to assess the ST segment), and contraindications specific to dobutamine infusion.

Table 26 presents the recommendations for the use of MRI in the investigation of myocardial ischemia.

**Table 26:** Recommendations for the use of magnetic resonance imaging in the investigation of CAD – myocardial ischemia

Indication	Class of recommendation	Level of evidence
Assessment of myocardial perfusion with dipyridamole/adenosine stress.^10 , 297 , 494 , 497 , 498 , 508^	I	A
Assessment of ventricular contractility with dobutamine stress.^489 , 502 , 506 , 509^	I	B
Investigation of CAD in patients with acute chest pain and intermediate pretest probability of CAD.^8 , 10 , 498^	I	B
Assessment of stable angina/anginal equivalent in patients with intermediate pretest probability of CAD.^8 , 10 , 494 , 498 , 508^	I	B
Identification and quantification of myocardial ischemia in patients with known CAD (except patients with high-risk anatomy * ).^510 , 511^	I	B
Investigation of myocardial ischemia in revascularized patients (surgically or percutaneously) with symptoms suggestive of obstructive CAD.^512^	I	B
Evaluation of patients with known nonobstructive CAD and/or suspected MINOCA.^513 – 515^	IIa	C

* Defined as left main stenosis > 50% and proximal 3-vessel coronary disease.

CAD: coronary artery disease; MINOCA: myocardial infarction with nonobstructive coronary arteries.

### 3.3. Investigation of DAC Using MRI – Myocardial Viability

CMR with LGE is considered the most clinically accessible method for assessing myocardial viability. Imaging for myocardial viability can help in both diagnostic and prognostic evaluation. However, clinical trials have challenged its effectiveness, indicating that imaging for myocardial viability may fail to accurately predict which patients will benefit from myocardial revascularization in terms of reduction of adverse cardiac events.^516 , 517^Nevertheless, a recent reevaluation of myocardial viability based on advanced CMR techniques and^518^long-term follow-up data from randomized studies has emerged.^519^Moreover, most of the major clinical studies that initially questioned the effectiveness of imaging for myocardial viability did not use CMR. Thus, imaging for myocardial viability still has significant clinical implications, and new ongoing randomized clinical trials suggest that myocardial viability plays a crucial role in clinical assessment.^520^Therefore, despite ongoing debate, CMR still has significant potential for the assessment of myocardial viability with the aim of improving patient outcomes.^521 – 523^

#### 3.3.1. CMR Techniques

CMR is able to characterize suspected hibernating myocardium using a combination of techniques: LV end-diastolic wall thickness (EDWT), inotropic reserve of segmental contractile function, and transmural extent of myocardial LGE. Other CMR methods for evaluating myocardial viability are not routinely used in clinical practice and are beyond the scope of this Guideline.

In patients with chronic CAD, wall thinning develops as a result of infarct resorption and fibrotic contracture, but it may also occur as a result of severe ischemia. Thus, EDWT alone has limited prediction of functional recovery after revascularization. Classically, an EDWT < 5.5 mm had no potential for segmental functional recovery after revascularization,^524^with a sensitivity of 94% but a specificity of only 52%. This cutoff has been questioned in a recent study estimating that approximately 20% of dysfunctional and thinned myocardial segments have limited scar burden and demonstrate improved contractility and resolution of wall thinning after coronary revascularization.^525^

Gadolinium-based contrast agents (GBCAs) shorten the T1 relaxation time of surrounding tissues proportionally to local gadolinium concentration. Gadolinium is a large, high-density element that enters the extracellular space after intravascular injection but cannot cross the cell membrane of a normal myocyte. However, when the myocyte cell membrane is damaged (eg, acute MI) or if there is an increase in the extracellular space between myocytes, GBCA accumulates in the extracellular space, and its washout is delayed after the injection. Thus, areas of abnormal myocardium will have a bright signal on images relative to the surrounding normal myocardium. Myocardial LGE in both acute and chronic MI is believed to result directly from an absence of viable myocytes ("bright is dead").^526^

In clinical routine, current protocols recommend performing LGE imaging 5 to 10 minutes after GBCA injection.^527 , 528^In patients with CAD, dysfunctional myocardium with normal nulled signal intensity suggests myocardial stunning or hibernation and an absence of infarction. LGE in acute and chronic MI will appear brighter (white) and will almost always involve the LV subendocardial layer, being often restricted to a specific coronary artery territory. In patients with CAD and global or segmental LV dysfunction being evaluated for benefits of coronary revascularization, the transmural extent of myocardial LGE provides a prediction of the stepwise decreasing likelihood of improvement in segmental myocardial contractility after revascularization.^422^Akinetic segments with no or minimal subendocardial infarction (< 25%) have a > 90% chance of segmental recovery of contractile function if the involved coronary artery is successfully revascularized. Segments with > 50% transmural extent of infarction have a < 10% chance of segmental contractile recovery irrespective of successful coronary revascularization.^422^Finally, segments showing 25%–50% transmural extent of infarction typically have a likelihood of contractile recovery close to 50% and are, therefore, considered viable segments with preserved potential for contractile recovery.^12 , 418 , 421 , 529 – 531^However, prediction of functional recovery based on LGE transmural extent alone in segments with 25%–50% extent may not be accurate, and other criteria such as infarct size (percentage of LV with infarction), number of viable and nonviable segments, adjacency to nonviable segments, and assessment by inotropic contractile reserve should be considered.^532 – 535^

One advantage of CMR with LGE is the ability to assess the presence of subendocardial MI and to delineate the transmural extent of MI at high spatial resolution (typically 1-3 mm in-plane resolution), allowing the detection of small infarcts that would be missed by other imaging methods.^421^

Inotropic contractile reserve in response to low-dose dobutamine infusion (5-10 μg·kg−1·min−1) is a well-validated physiological parameter of myocardial viability assessed by either echocardiography or CMR.^532 , 536 , 537^Compared with other parameters, inotropic contractile reserve tends to achieve a higher specificity because it examines a similar endpoint of segmental contractile function, but with lower sensitivity. Although dobutamine-induced contractile reserve has a higher specificity than the transmural extent of myocardial LGE for predicting segmental functional recovery, especially in intermediate cases (25%-50%),^532^it is only moderately sensitive in the prediction of segmental contractile recovery.^538^The combination of these techniques may make prediction of segmental functional recovery after revascularization more accurate. In this setting, LGE provides the highest sensitivity of 95%, which is complemented by a high specificity of 91% offered by dobutamine contractile reserve.^539^

#### 3.3.2. Routine Clinical Use

#### 3.3.2.1. Acute Setting

In patients with AMI, by determining the transmural extent of the infarcted area, CMR helps to differentiate stunned or hibernating segments (viable myocardium) from fundamentally necrotic or fibrotic tissue with no potential for functional recovery (irreversible injury).^540^CMR is an accurate tool to measure cardiac function, adverse remodeling,^541^and the extent of microvascular obstruction (no-reflow).^542 – 544^

In this setting, CMR with LGE should be performed in patients with extensive segmental contractile dysfunction (eg, akinetic anterior wall) in association with obstructive CAD amenable to either surgical or, preferably, percutaneous revascularization. An algorithm for the assessment of myocardial viability in AMI using MRI is presented in Figure 5 . In patients with MI, the predictive power of contractile recovery in the acute stage is similar to that in the chronic stage. However, in the acute stage, the reduction in infarct size promoted by the healing process (overall LGE may decrease by approximately 22% in the first 6 months after MI) should also be considered when evaluating LGE extent.^418 , 545^

**Figure 5:** Assessment of myocardial viability using magnetic resonance imaging (acute setting). *Defined by anginal symptoms, electrocardiographic monitoring, or imaging testing. CMR: cardiac magnetic resonance imaging; ECHO: echocardiography; LGE: late gadolinium enhancement; LV: left ventricular; PET: positron emission tomography.

#### 3.3.2.2. Chronic Setting

The assessment of myocardial viability to predict which patients will have improved global or regional ventricular function after myocardial revascularization is of great clinical utility.^546^

In chronic MI, CMR was shown to be superior to SPECT scintigraphy^421 , 547^and to have similar sensitivity and specificity to PET in the identification of areas of fibrosis,^57 , 548^especially subendocardial infarction. The assessment of the transmural extent of myocardial LGE allows for highly accurate prediction of regional function recovery after either surgical or percutaneous revascularization.^422 , 518 , 549 – 551^

A study demonstrated a strong association between myocardial viability on noninvasive testing and improved survival after revascularization in patients with chronic CAD, LV dysfunction, and coronary anatomy amenable to revascularization.^552^Conversely, absence of viability was associated with no significant difference in mortality in these patients.^552^

A common use of LGE in our practice is for the investigation of akinetic and dyskinetic segments, including for suspected LV aneurysm, particularly if the patient is scheduled for surgical revascularization. The absence of myocardial viability in these segments can help guide the decision on whether to perform aneurysmectomy or correction of LV geometry, as well as assist the cardiac surgeon in preoperative planning.^485^Although the prognostic value of this approach has not yet been confirmed, the presence of myocardial viability portends a better prognosis irrespective of the chosen therapeutic approach: optimal medical therapy or surgical revascularization.^1 , 516^

In patients with regional dysfunction at rest, low-dose dobutamine stress CMR has proven useful in the assessment of contractile reserve.^538^Patients with preserved myocardial viability experience improvement in segmental ventricular dysfunction during low-dose dobutamine infusion. In these patients, systolic thickening is more likely to improve after myocardial revascularization.^532 , 539 , 553^

An algorithm developed to guide management of patients with coronary anatomy amenable to myocardial revascularization is fundamentally based on the assessment of myocardial viability and the presence of myocardial ischemia. Contractile dysfunction and wall thinning are less important in chronic than in acute cases, as they may result from myocardial hibernation/stunning or absence of myocardial viability (transmural infarction).^525 , 534^Similarly, patients showing 25%-50% transmural extent of myocardial LGE benefit the most from assessment of contractile reserve with dobutamine and of metabolic reserve with PET imaging ( Figure 6 ).^532 , 539 , 553^

**Figure 6:** Assessment of myocardial viability using magnetic resonance imaging (chronic setting). *Defined by anginal symptoms, electrocardiographic monitoring, or imaging testing. CMR: cardiac magnetic resonance imaging; ECHO: echocardiography; LGE: late gadolinium enhancement; PET: positron emission tomography.

#### 3.3.3. Class of Recommendation and Level of Evidence

International guidelines have been progressively increasing the role of CMR in the assessment of myocardial viability. In ACS, CMR has a Class I recommendation and Level of Evidence B for suspected stress cardiomyopathy (Takotsubo) and for the differential diagnosis of myocardial infarction with nonobstructive coronary arteries (MINOCA).^554^The assessment of myocardial viability has a Class IIa recommendation and a Level of Evidence B for patients with known CAD presenting with HF,^139 , 555 , 556^and a similar recommendation is observed for the evaluation of ventricular arrhythmia in CAD.^557^Similar indications with high Classes of Recommendation and Levels of Evidence were endorsed by the 2006^558^and 2014^2^SBC/CBR guidelines on CMR and CCTA, respectively.

Table 27 presents recommendations from the III CMR and CCTA Guidelines, based on the best scientific evidence compiled by the expert group at the time of its development.

**Table 27:** Recommendations for the use of magnetic resonance imaging in the assessment of CAD – myocardial viability

Indications for cmr in the assessment of myocardial viability in the setting of global or regional ventricular dysfunction (heart failure) in patients with known or suspected CAD	Class of recommendation	Level of evidence
Evaluation of regional ventricular function at rest and under stress.^559 – 561^	IIa	B
Detection of acute and chronic myocardial infarction by LGE.^422 , 518 , 525 , 528 , 562 , 563^	I	A
Differential diagnosis of nonischemic cardiomyopathy by LGE (other cardiomyopathies).^564 – 566^	I	B
Assessment of myocardial viability prior to revascularization by LGE * .^422 , 518 , 567^	I *	B *
Detection of LV thrombus.^568 – 571^	I	B
Investigation of LV aneurysm.^525 , 572^	I	B

CAD: coronary artery disease; MRI: magnetic resonance imaging; LGE: late gadolinium enhancement; LV: left ventricular.

* This Level of Evidence and Class of Recommendation refer to the choice of CMR as the preferred method for assessing myocardial viability. This classification does not represent the general Level of Evidence and Class of Recommendation for myocardial viability assessment as a pre-revascularization strategy.

### 3.4. Coronary Magnetic Resonance Angiography

The diagnosis of CAD and the assessment of myocardial ischemia are critical for the prevention of future cardiovascular events.

Coronary magnetic resonance angiography (CMRA) does not require the use of ionizing radiation or contrast agents. Despite early recognition of its potential, significant technical limitations, such as low spatial resolution, longer acquisition times, and low signal-to-noise ratios that compromise image quality, have led to the replacement of CMRA with other noninvasive techniques for the assessment of CAD. Due to its technical advantages over CMRA and a faster acquisition time, the role of CCTA has increased in routine clinical practice.^573 – 575^CMRA has been used as a noninvasive method for assessing CAD in different patient populations, in both single-center^576 , 577^and multicenter studies,^578 , 579^but it has not shown good reproducibility in real-world settings. However, CMRA was shown to be valuable in the characterization of congenital CAAs, for which it is considered the clinical method of choice, particularly when there are concerns about the use of radiation and contrast agents.^580 , 581^

In summary, there are 3 main lines of research development in coronary artery evaluation by CMR:

1. Assessment of the degree of coronary stenosis with the implementation of techniques that significantly reduce signal intensity in regions with greater stenosis.^582 , 583^
2. Characterization of plaque features and vulnerability.^584 , 585^
3. Physiologic assessment of coronary artery flow, including coronary blood flow analysis using phase-contrast techniques,^586^coronary sinus blood flow,^587^coronary flow reserve, and flow gradient across coronary stenoses.^588 , 589^

Current evidence indicates that CMRA may be used in clinical practice to investigate the origin of coronary arteries and the presence of Kawasaki disease (proximal segments with larger diameters), with a Level of Evidence IIa ( Table 28 ).

**Table 28:** Recommendations for the use of coronary magnetic resonance angiography

Indication/Clinical setting	Class of Recommendation	Level of Evidence
Evaluation of coronary artery origin.^590 , 591^	IIa	B
Follow-up of patients with Kawasaki disease (evaluation of coronary aneurysms).^592 – 594^	IIa	B
Evaluation of coronary artery disease.^10^	III	B

The assessment of the degree of stenosis, coronary flow reserve, and flow gradient across stenoses is still being investigated.

### 3.5. Differential Diagnosis of Troponin-positive Nonobstructive Coronary Arteries (TP-NOCA/MINOCA)

Although the diagnosis of AMI is typically associated with the presence of coronary obstruction, there is a significant number of patients (~6%-8%)^595^with ACS but no angiographic obstructive CAD.^596^The diagnosis of MINOCA requires documentation of an AMI and invasive coronary angiography or CCTA showing no significant obstruction.^597^In addition, like the diagnosis of MI, it indicates that there is an ischemic mechanism responsible for the myocyte injury, meaning that nonischemic causes such as myocarditis and Takotsubo syndrome are excluded. The latest definition of MI from the European Society of Cardiology^598^specifies 3 criteria that must be met to confirm MINOCA: 1) the usual criteria for MI (abnormal cardiac biomarkers in the setting of evidence of AMI); 2) no stenosis ≥ 50% on coronary angiography; and 3) no other clinically overt specific cause for myocardial injury (eg, myocarditis or PE). In this section, we will discuss some differences in clinical presentation commonly seen in differential diagnoses of MINOCA.

Many investigators consider MINOCA, like HF, a working diagnosis due to the difficulty in determining its etiology to guide clinical treatment.^597^Therefore, some conditions sharing features with type I or II AMI should be considered in the differential diagnosis of MINOCA. These include ischemic diseases resulting from coronary plaque erosion, rupture, or ulceration, coronary dissection, thromboembolism, microvascular coronary spasm, coronary embolism, as well as inflammatory cardiomyopathies (myocarditis of any etiology), Takotsubo syndrome, or even PE.^599^Thus, the accurate diagnosis of MINOCA is crucial for choosing the best therapeutic approach for both ischemic and nonischemic cases.^600^Due to their diagnostic complexity, it has been suggested to encompass the aforementioned conditions under the term "troponin-positive with nonobstructive coronaries arteries" (TP-NOCA), which would refer to syndromes characterized by troponin elevation in the absence of coronary obstruction, with troponin elevation being the common marker. The causes of TP-NOCA could then be subcategorized into epicardial coronary (eg, MINOCA), myocardial (eg, myocarditis), and extracardiac (eg, PE).^601^

CMR is one of the most important tools for determining the etiology of MINOCA, being able to identify a cause in 74% of cases.^602^When present, LGE allows localization of the area of myocardial injury and provides evidence of the mechanisms involved. Additionally, CMR helps identify patients with a poor prognosis, potentially altering clinical treatment in approximately 50% of cases,^603^and allows for personalized medical treatment (including secondary prevention). Moreover, CMR can prevent the unnecessary prescription of medications and their side effects, such as bleeding in the case of antiplatelet agents.^602^

Approximately 23% of MINOCA cases are associated with nonobstructive coronary atherosclerosis. These cases are commonly caused by plaque erosion or ulceration, which leads to acute thrombosis and vessel recanalization, resulting from prolonged vasospasm.^595^CMR can confirm the diagnosis of MI with the use of LGE and allows its differentiation from other lesions, such as myocarditis, by evaluating the presence of edema using either conventional T2-weighted imaging or parametric mapping (T1 and T2 maps).^604^

Another relevant cause of myocardial injury that is commonly confused with MINOCA is myocarditis, which corresponds to approximately 29% of cases.^595^Viral infection is the most common cause of myocarditis, having gained greater repercussion after the Covid-19 pandemic, and is associated with myocardial changes in approximately 50% of recovered patients.^605 – 608^The use of CMR in the evaluation of myocarditis will be addressed in a dedicated section of this Guideline.

Approximately 16% of MINOCA cases will present as Takotsubo syndrome.^595^CMR is an excellent diagnostic tool in this setting, as it allows the identification of dyskinetic areas in any segment of the LV, although transient apical dyskinesia is the most frequent manifestation of Takotsubo syndrome. Furthermore, it can identify areas of myocardial edema, being regarded as the method of choice in recently published consensuses.^609^The inflammatory phase, in which myocardial edema can be detected, usually disappears within 3 months.^610^Control CMR may be requested in these cases to assess for reversal of dyskinesia, as well as disappearance of myocardial edema, thus confirming the Takotsubo diagnosis. LGE is not commonly seen in this syndrome. However, in the acute phase of inflammation, small patches of LGE may be seen in dyskinetic areas due to an increase in interstitial space.

HCM, nonischemic DCM, and amyloidosis are less common and may even be the cause of MINOCA in 3%, 2%, and less than 5% of cases, respectively.^595 , 604^They will be addressed in specific sections of this Guideline.

Thus, due to its excellent performance in accurately diagnosing infarcted areas related to MINOCA, CMR is highly recommended in the diagnostic investigation of TP-NOCA/MINOCA, with a Class I recommendation and Level of Evidence B^609 , 610^( Table 29 ).

**Table 29:** Recommendations for the use of cardiac magnetic resonance imaging in the evaluation of positive-troponin with nonobstructive coronary arteries

Indication	Class of recommendation	Level of evidence
Differential diagnosis of troponin elevation in nonobstructive coronary artery disease.^611 – 613^	I	B

### 3.6. Stress Cardiomyopathy (Takotsubo)

Takotsubo cardiomyopathy (also known as stress cardiomyopathy or broken heart syndrome) was first described by Japanese authors in 1990.^614^It most commonly affects women over 55 years of age and is characterized by chest pain and ECG changes mimicking AMI, in association with characteristic dysfunction of the mid and apical segments of the LV.^615 , 616^Classically, it does not cause significant coronary obstruction and is preceded by major physical or emotional stress.^617^More recently, cardiac alterations similar to Takotsubo cardiomyopathy have been described in patients with Covid-19.^618 , 619^

The current diagnostic criteria (InterTAK Diagnostic Criteria) include reversible LV dysfunction presenting as apical ballooning; history of emotional or physical stress; ECG abnormalities; and moderately elevated cardiac markers (disproportionately to ventricular dysfunction).^619^The presence of CAD does not rule out Takotsubo syndrome, as both conditions may coexist, and the presence of myocarditis or irreversible myocardial injury (infarction) should be excluded.^609^

CMR is useful in the evaluation of suspected Takotsubo syndrome because of its ability to assess global and segmental function and characterize myocardial tissue.^620 , 621^The CMR protocol should include techniques such as cine MRI, dark-blood T2-weighted imaging, and LGE. When available, parametric mapping techniques (native T1 map, ECV, and T2 mapping) may be useful in the identification of myocardial changes in normal segments by visual assessment.^622^

The presence of characteristic segmental dysfunction associated with reversible myocardial damage (edema) is highly suggestive of Takotsubo cardiomyopathy. LGE does not typically occur in the classic form of Takotsubo syndrome, but LGE with a lower signal intensity has been described in hyperacute settings.^620 , 621^

CMR is also useful in the evaluation of other differential diagnoses that should be ruled out to confirm Takotsubo syndrome, such as MI and myocarditis, as well as in the identification of complications such as intracardiac thrombi.^620^

In summary, CMR is useful in the initial evaluation of suspected Takotsubo syndrome, as it can contribute to the detection of diagnostic features and differentiation from other conditions with similar clinical characteristics.^623^ Table 30 presents the recommendations for the use of CMR in Takotsubo cardiomyopathy.

**Table 30:** Recommendations for the use of cardiac magnetic resonance imaging in the assessment of stress cardiomyopathy (Takotsubo syndrome)

Indication	Class of recommendation	Level of evidence
Evaluation of suspected Takotsubo cardiomyopathy (including assessment of global and segmental ventricular function).^609 , 624^	I	B
Investigation of apical thrombus in patients with Takotsubo cardiomyopathy.^621 , 625^	I	B
Evaluation of suspected Takotsubo cardiomyopathy after an inconclusive echocardiogram or with poor acoustic window.^621 , 626^	I	C

### 3.7. Myocarditis/Inflammatory Cardiomyopathy

Myocarditis is an inflammatory disease of the heart that may occur due to infections, exposure to toxic substances, or immune system activation.^627^Viral infection is the most common cause, and clinical presentation ranges from asymptomatic to sudden death. Common symptoms include precordial pain, dyspnea, fatigue, palpitations, and syncope.^628^ECG changes are seen in 85% of cases, including ST-segment elevation, QRS widening, and arrhythmias associated with elevated biomarkers of myocardial necrosis (high-sensitivity troponin).^627^

Myocarditis is diagnosed based on a combination of clinical presentation, physical examination, laboratory tests, and imaging studies. CMR is useful because it is sensitive to tissue changes that occur during myocardial inflammation.^419 , 437^The diagnosis of myocarditis by CMR relies on the Lake Louise Criteria, revised in 2018 to incorporate parametric mapping techniques and ECV measurement, thereby increasing diagnostic accuracy. Acute myocardial inflammation may be detected if at least 1 criterion from each category is present,^437^including myocardial edema by T2-weighted imaging or T2 mapping and myocardial injury by LGE, increased native T1, or increased ECV.^437 , 629^T2 relaxation times are most elevated during the acute phase of myocarditis and gradually normalize over the following months, and this feature may be useful for both diagnosis and monitoring recovery.^630^T1 relaxation times may be prolonged due to intracellular or extracellular edema, hyperemia, and the presence of areas of fibrosis, while ECV may be expanded due to extracellular edema.^437 , 630^Having both criteria positive increases diagnostic specificity, while having only one may still support a diagnosis of acute myocardial inflammation in an appropriate clinical setting.^437^In the absence of LGE and positive clinical features, changes in native T1 mapping and ECV may be indicative of myocardial injury. In these cases, increased native T1 values in non-LGE areas showed a greater sensitivity with T1 mapping without increasing false positives.^628^

In addition to diagnostic utility, CMR also provides prognostic insight. Biventricular dysfunction resulting from significant myocardial involvement is a major predictor of death. The presence of LGE also predicts mortality and is correlated with the risk of sudden death and the development of LV dilatation and reduced EF.^628^An EF ≤ 40% in association with LGE increases the risk of MACE by 10% per year.^628^

Covid-19 has been frequently associated with myocardial injury. At follow-up, the most common finding is diastolic dysfunction (55%), but only 2.8% have reduced LVEF. In patients with acute Covid-19, the most common CMR findings include abnormal T2 and native T1 times, pericardial abnormalities (myopericarditis), and a nonischemic pattern of LGE. Recovered patients who had moderate-to-severe COVID-19 often have abnormal CMR findings, the most common being pericardial and myocardial LGE (especially subepicardial and mid-wall) and lower LV and RV EFs compared with controls. Native T1 and T2 maps were also higher in these patients. Asymptomatic patients or those with mild symptoms did not have significant changes compared with controls.^631^

CMR in suspected myocarditis has a Class I indication for the diagnostic and prognostic investigation of acute and chronic myocarditis and/or suspected prior myocarditis ( Table 31 ). The recent incorporation of parametric T1 and T2 mapping and ECV data increases diagnostic sensitivity.

**Table 31:** Recommendations for the use of cardiac magnetic resonance imaging in the assessment of myocarditis/inflammatory cardiomyopathy

Indication	Class of recommendation	Level of evidence
Evaluation of ventricular function, geometry, and morphology when acute, subacute, or chronic myocarditis is suspected.^371 , 437 , 439 , 479^	I	B
Diagnostic and prognostic evaluation of acute, chronic, and/or suspected prior myocarditis.^371 , 437 , 439 , 479^	I	B
Follow-up at 4 and 12 weeks after an acute event to differentiate between complicated or uncomplicated progression.^371 , 437^	IIa	B
Evaluation of fulminant myocarditis with hemodynamic instability.^371 , 437^	III	B

### 3.8. Athlete's Heart

Athlete's heart syndrome is typically observed in elite athletes and, depending on the type of training, may lead to increased heart volumes and diameters, increased ventricular mass, or a combination of both.^632 – 635^These findings are typically assessed by echocardiography, but CMR can be useful in cases where echocardiography is inconclusive.^636 – 640^

Differentiating between athlete's heart and other cardiomyopathies that share similar features is crucial to avoid significant consequences related to misdiagnosis. Sudden death in athletes may occur in seemingly healthy individuals with no previous known cardiovascular abnormalities.^636 , 641^

Early forms of HCM, DCM, and left ventricular non-compaction/excessive trabeculation of the left ventricle should be included in the differential diagnosis of athlete's heart.^642^In addition, associated conditions such as arrhythmogenic cardiomyopathy, myocarditis, and even CAD may increase the risk of adverse outcomes if undiagnosed.^638^

CMR is the gold standard for the quantification of ventricular volumes and function. Normal reference values have already been established for the general population,^581 , 643^and a recent systematic review^634^provided reference values for male athletes. End-diastolic volume and ventricular mass varied between endurance-trained and resistance-trained male athletes and according to the type of sport.

Differentiating between athlete's heart and early-stage cardiomyopathy is not always straightforward, and the accurate measurements provided by CMR may assist in this process. LGE imaging for assessing fibrosis can help support the diagnosis. For example, myocardial fibrosis is not expected in patients with athlete's heart but is a common finding in patients with HCM (although myocardial fibrosis has been described in some marathon runners and triathletes).^644 , 645^Data on the geometric pattern of hypertrophy, LVOT obstruction, and left atrial remodeling also provide further diagnostic insight. If present, they suggest the diagnosis of HCM. In some cases, interruption of training with monitoring of parameters such as mass, volume, and EF may be the only way to resolve the differential diagnosis.^646^Most cardiovascular changes seem to regress after 9 to 12 weeks of detraining, but LV dilation may persist in 20% of cases.^647^

Parametric mapping can assist in the differentiation between athlete's heart and other cardiomyopathies. T1 mapping and ECV fraction are typically lower in athlete's heart than in other cardiomyopathies,^648^but more studies are needed to better evaluate the role of these techniques in the differential diagnosis.

CMR allows for a more accurate measurement of heart parameters than other methods, making it highly suitable for the evaluation of athlete's heart^648^and to differentiate it from other cardiomyopathies. Table 32 presents the recommendations for the use of CMR to assess changes in cardiac morphology in athletes.

**Table 32:** Recommendations for the use of cardiac magnetic resonance imaging in the assessment of athlete's heart

Indication	Class of recommendation	Level of evidence
Differential diagnosis of changes in cardiac morphology (dilation, ventricular hypertrophy, prominent trabeculations) in athletes.^646 , 649 – 651^	I	B

### 3.9. Hypertrophic Cardiomyopathy

HCM is a hereditary disease characterized by progressive LVH in the absence of associated hemodynamic stress or systemic diseases related to myocardial deposition (eg, CA). Potential complications include sudden death, LVOT obstruction, HF, and thromboembolic stroke, making it the leading cause of sudden death in young athletes. Nevertheless, the use of conventional treatment as well as an ICD in high-risk patients can reduce mortality to less than 1% per year.^652 – 656^

The diagnosis of HCM through echocardiography or CRM is based on the presence of a maximal LV wall thickness of ≥ 15 mm in the absence of any other causes of LVH.^657^In family members of a patient with HCM or in conjunction with a positive genetic test, a LV wall thickness of 13-14 mm can be diagnostic.^658 – 660^

The basal anterior septum in continuity with the anterior free wall is the most common location for LVH. However, the pattern and location of LVH can vary widely even without an increase in total myocardial mass. Other morphologic abnormalities may also occur in HCM, such as apical hypertrophy, myocardial crypts, anomalous papillary muscle insertion, mitral valve leaflet elongation, and RV hypertrophy.^653 , 661^

CMR is crucial in the diagnostic evaluation of patients with HCM, particularly those in whom echocardiography presented some limitation (eg, poor acoustic window) or when there is doubt about the indication of an ICD due to the risk stratification of arrhythmic events.^419^

The pattern of myocardial fibrosis also varies in patients with HCM, but typically presents as multifocal fibrosis (sometimes mimicking the pattern of myocardial fibrosis due to MI). Syncope, nonsustained ventricular tachycardia, a family history of sudden death, a drop in blood pressure during exercise, and LV wall thinning are independent risk factors for sudden death in HCM.^653 , 661 , 662^

The identification of risk markers such as apical aneurysms, decreased systolic function, and the quantification of myocardial fibrosis by LGE also makes CMR increasingly important in the evaluation of HCM. The quantification of myocardial fibrosis in particular makes CMR essential in many patients with HCM.^663 , 664^

The identification of the following risk factors by CMR indicates a worse prognosis in patients with HCM: 1) LVEF < 50%; 2) LV wall thickness > 30 mm; 3) apical aneurysm; and 4) LGE, representing fibrosis, comprising > 15% of LV mass (either quantified by software or estimated by visual inspection).^664 – 666^

Table 33 presents the main anatomic features associated with HCM on CMR.

**Table 33:** Anatomic features associated with hypertrophic cardiomyopathy on cardiac magnetic resonance imaging

Feature	Observations	Marker of worse prognosis
Ventricular volume	Typically reduced	
LV outflow tract obstruction	Typically observed by cine CMR at rest	
LV thickness	> 15 mm (or > 13-14 mm in patients with other diagnostic factors)	> 30 mm
Location of increased LV thickness	Typically located at the basal anterior septum, but may vary	May lead to LV outflow tract obstruction
Ejection fraction	Often > 80%	< 50%
Left atrial volume	Often increased	Higher chance of atrial fibrillation
Apical aneurysm	Associated or not with myocardial fibrosis	Sign of poor prognosis
LGE	Varied LGE pattern	Myocardial fibrosis > 15%

LGE: late gadolinium enhancement; LV: left ventricular.

CMR is an important tool in suspected HCM and has become essential in sudden death stratification, especially for tissue characterization and estimation of the degree of myocardial fibrosis. Table 34 presents the recommendations for the use of CMR in suspected HCM.

**Table 34:** Recommendations for the use of cardiac magnetic resonance imaging in the assessment of suspected hypertrophic cardiomyopathy (HCM)

Indication	Class of recommendation	Level of evidence
Evaluation of suspected HCM in patients with inconclusive echocardiography, or with normal echocardiography but conflicting clinical/electrocardiographic findings.^640 , 667 – 670^	I	A
Differential diagnosis of left ventricular hypertrophy.^667 , 669 – 672^	I	A
Investigation of myocardial fibrosis for risk stratification in carriers of HCM mutations.^669 – 673^	I	A
Patients with left ventricular outflow tract obstruction in whom echocardiography was inconclusive.^669 , 670 , 674 – 676^	IIa	B

### 3.10. Endomyocardial Fibrosis

The pathogenesis and etiology of EMF are largely unknown, but are commonly attributed to parasitic infections (eg, helminths) and genetic susceptibility, leading to eosinophilia and elevated immunoglobulin E. Tropical EMF is considered endemic in developing countries, including Brazil,^677^while Loeffler's endocarditis, the late form of hypereosinophilic syndrome, usually occurs in temperate zones. EMF is characterized by ventricular apical obliteration with or without thrombus and/or calcification, in association with diastolic dysfunction, atrial enlargement, and possibly valve regurgitation. It may affect one or both ventricles, but the rates of ventricular involvement reported in the literature vary according to the method used.^678 , 679^

Due to its ability for tissue characterization, CMR is fundamental in the diagnosis of EMF. Cine CMR images can confirm morphofunctional findings that may have already been seen on echocardiography. It is extremely important to rule out differential diagnoses, especially apical HCM and conditions that may occasionally affect the apex, such as aneurysms with thrombus (secondary to HCM, Chagas disease, or infarction) and tumors.^680^

However, diagnostic confirmation and differentiation from other conditions are primarily made via tissue characterization by LGE. Classically, LGE in EMF is subendocardial, involving the LV and/or RV apex and potentially extending to the inflow tract, while the outflow tracts are usually spared.^679^A Brazilian group identified another typical finding termed "double V",^681^characterized by a hyper and hypointense double-layered image corresponding to fibrosis plus thrombus/calcification. These findings showed excellent diagnostic and prognostic value^679^( Table 35 ). Table 36 presents the recommendations for the use of CMR in suspected EMF.

**Table 35:** Morphological and tissue features of endomyocardial fibrosis on cardiac magnetic resonance imaging

**Morphofunctional findings (in one or both ventricles)** - Apical obliteration - Reduction in ventricular volumes (< 57 mL/m² ± 15 for LV and < 56 mL/m² ± 30 for RV), although there may be a compensatory increase in the diameter of the basal portion.^678^ - Normal or slightly reduced ejection fraction. - Atrial enlargement. - Valve regurgitation (mitral and/or tricuspid), with or without papillary muscle anomalies, such as fusion or adhesion to the ventricular walls.
Tissue alterations - Subendocardial late gadolinium enhancement not related to the coronary territory, mostly affecting the ventricular apex. - Double V (with thrombus and/or apical calcification) or single V sign (fibrosis).
Prognostic value - Fibrous tissue volume > 19 mL/m².^679^

**Table 36:** Recommendations for the use of cardiac magnetic resonance imaging in the assessment of suspected endomyocardial fibrosis

Indication	Class of recommendation	Level of evidence
Evaluation of suspected endomyocardial fibrosis.^419 , 679^	I	B
Differential diagnosis of apical hypertrophy.^419 , 667 , 670 , 671^	I	B

### 3.11. Cardiac Amyloidosis

CA is an infiltrative disease caused by amyloid fibril deposition in the myocardium. The most common variants leading to CA are amyloid light chain (AL) and amyloid transthyretin (ATTR, hereditary, or wild type) amyloidosis.^682^Cardiac involvement may occur in different stages of the disease, has prognostic implications, and requires different treatments depending on its severity. This is why it is extremely important to differentiate between the 2 forms. Current guidelines recommend that diagnosis be made using noninvasive imaging tests and that cardiac biopsy be reserved for exceptional cases. CMR has great clinical applicability in the diagnosis of CA, together with^99m^technetium pyrophosphate (^99m^Tc-PYP) scintigraphy.

To assist in the differential diagnosis of CA etiology, which includes both restrictive cardiomyopathy and other causes of myocardial hypertrophy,^683^CMR protocols currently include several techniques.^684^Morphological and functional assessment shows concentric or even asymmetric hypertrophy of the LV and RV, which is more pronounced in ATTR amyloidosis. Involvement of the interatrial septum is common, and CMR can help differentiate between hypertrophy and lipomatous degeneration. LGE in CA has a typical pattern of diffuse subendocardial enhancement, but more advanced cases may display a transmural pattern affecting the entire myocardium.^685^LGE not only provides diagnostic information but also has prognostic value, with the chance of event-free survival being inversely proportional to the degree of deposition.^686^

More recently, the measurement of native T1 map and ECV has been used to assist in the diagnosis of CA, especially in patients who cannot receive contrast.^687^Patients with CA have markedly increased native T1 values, and ECV has proven to be an additional prognostic marker even in patients with similar degrees of LGE.

The main indications for CMR in CA include patients with myocardial hypertrophy detected by echocardiography whose etiology is uncertain.^688^In patients already diagnosed with amyloidosis where myocardial involvement is to be determined, CMR can be performed even before the phenotypic development of hypertrophy. Finally, CMR can also be useful in clinical practice to establish a prognosis and assist in choosing the most appropriate therapies. Especially in cases where^99m^Tc-PYP scintigraphy shows weak or no myocardial uptake and hematological tests are positive, CMR is essential for the diagnosis of suspected AL amyloidosis.

Table 37 presents the recommendations for the use of CMR in the diagnostic evaluation of patients with suspected CA.

**Table 37:** Recommendations for the use of cardiac magnetic resonance imaging in the assessment of cardiac amyloidosis

Indication	Class of recommendation	Level of evidence
Differential diagnosis of ventricular hypertrophy in patients with suspected cardiac amyloidosis according to clinical, laboratory, and/or previous imaging findings.^667 , 671 , 689 , 690^	I	A
Diagnosis of cardiac amyloidosis in patients with conflicting test results.^691 – 693^	IIa	B
Prognostic follow-up and therapeutic management of patients with confirmed cardiac amyloidosis.^690 , 691 , 694 – 696^	IIa	B
Diagnostic confirmation of light chain amyloidosis in patients with compatible hematological abnormalities and clinical suspicion.^690 , 691 , 697^	IIa	C

### 3.12. Cardiac Hemosiderosis

The diagnosis of myocardial and hepatic iron overload has direct clinical applicability in diseases characterized by repeated transfusions and/or increased intestinal iron absorption due to pathological mechanisms. The main conditions include thalassemias, myelodysplastic syndromes, and hereditary hemochromatosis.^698^MRI is the only noninvasive technique available for quantification of tissue iron in these conditions. Serum ferritin is an indirect marker with poor correlation with myocardial iron overload, and invasive biopsy is restricted to the diagnosis of liver iron overload.

MRI assessment of iron overload does not require the use of contrast agents. The most well-known and validated technique is parametric T2* mapping, which was validated in multicenter studies and shown to correlate well with endomyocardial biopsy (EMB).^699^T2* is measured in milliseconds and can be used to estimate iron concentrations in both the liver and heart using commercial post-processing software or freely available online tools.^700^Iron is quantified at least in the liver and heart because these organs present different absorption rates and mechanisms, often resulting in a disparity in iron accumulation between them.^701^

In addition to being directly correlated with quantitative cardiac iron overload, cardiac T2* also provides prognostic information and can be used to monitor iron chelation therapy.^702^National and international clinical guidelines recommend starting T2* MRI screening at 7 or 10 years of age and repeating it annually; however, this interval may vary from 6 to 24 months depending on the patient's clinical condition and baseline transfusion and iron overload status.^471^Myocardial T2* values > 20 ms (< 1.16 mg/g of myocardial iron concentration) are associated with a better prognosis and are considered low risk for HF. Values < 10 ms (> 2.7 mg/g) portend a worse prognosis and are associated with increased risk of HF and arrhythmias (parameters referring to 1.5T MRI scanners).

Recent studies suggest that quantification of tissue iron may also be performed using T1 mapping, which may be more sensitive to detecting mild iron overload.^448^However, due to the lower availability and standardization of T1 mapping, it is not routinely used and should be reserved for situations where T2* is unavailable. Table 38 presents the main recommendations for the use of CMR in the imaging of myocardial iron overload.

**Table 38:** Recommendations for the use of cardiac magnetic resonance imaging in the diagnosis and management of myocardial iron overload

Indication	Class of recommendation	Level of evidence
Evaluation of siderotic cardiomyopathy, especially secondary to thalassemia.^426 , 473 , 699^	I	A
Diagnostic evaluation of hepatic or myocardial iron overload using quantitative T2* mapping.^426 , 427 , 473 , 703^	I	A
Longitudinal monitoring of iron overload management using quantitative T2* mapping.^474 , 703 , 704^	I	A
Diagnosis and longitudinal monitoring of iron overload using T1 mapping.^705^	IIb	C

### 3.13. Other Storage Diseases

#### 3.13.1. Anderson-Fabry Disease

Anderson-Fabry disease is an X-linked lysosomal disorder characterized by a deficiency in alpha-galactosidase A, leading to the progressive accumulation of complex glycosphingolipids, predominantly globotriaosylceramide (Gb3), in muscle cells, endothelial cells, and smooth muscle cells.^706^This accumulation in the myocardium, valves, and cardiac conduction system results in increased ventricular wall thickness, thickening of the valve leaflets, and arrhythmias.^707 , 708^The diagnosis of Anderson-Fabry disease is challenging because its morphological and clinical features can mimic other hypertrophic conditions, such as HCM and amyloidosis.

CMR may play an important role in evaluating patients with LVH of unknown origin. T1 mapping is particularly useful in this setting, as low native T1 values may indicate glycosphingolipid deposition even before the onset of parietal hypertrophy.^709^

The pattern of LGE is typically mid-mural, predominantly in the basal inferolateral segment of the LV, sparing the subendocardial region.^710 , 711^

#### 3.13.2. Glycogen Storage Disease

Glycogen storage diseases are hereditary metabolic disorders affecting the synthesis or degradation of glycogen within muscles, liver, and heart tissue.^712^

**Danon disease** is an X-linked dominant genetic disorder caused by a deficiency of the LAMP2 protein. In male patients, it manifests with the clinical triad of cardiomyopathy, skeletal myopathy, and intellectual disability, while in female patients it only manifests as cardiomyopathy.^712^The cardiomyopathy phenotype is generally hypertrophic, but a dilated phenotype has also been described. Myopathy is usually mild, with proximal muscle weakness, and nerve conduction studies reveal sensorimotor polyneuropathy.^713^

CMR shows massive hypertrophy (up to 4 cm), usually concentric. Some cases may exhibit asymmetric hypertrophy or a dilated pattern.^713 , 714^LGE is extensive and typically subendocardial and midmural, with nearly transmural involvement. Septal preservation is seen in 88% of patients, while lateral wall and apical LGE are present in nearly all cases.^10^Native T1 and ECV values are elevated and correlate with areas of LGE.^713^

**PRKAG2 syndrome** is a rare autosomal dominant hereditary disorder similar to Danon disease, but with normal liver function, normal serum creatine kinase levels, and no systemic disease.^714^PRKAG2 syndrome presents with ventricular hypertrophy and tachyarrhythmias, which may lead to sudden cardiac death, conduction disorders, severe myocardial hypertrophy, skeletal myopathy, and arrhythmias, often associated with Wolff-Parkinson-White syndrome.^714^

Cardiac hypertrophy primarily affects the LV and is progressive, accompanied by both systolic and diastolic dysfunction. High-voltage QRS complexes with ventricular repolarization abnormalities are observed even in the absence of LVH on echocardiography.^715 , 716^

Table 39 presents the recommendations for the use of CMR in myocardial storage diseases.

**Table 39:** Recommendations for the use of cardiac magnetic resonance imaging in the evaluation of myocardial storage diseases

Indication	Class of recommendation	Level of evidence
Evaluation of cardiac involvement in specific storage diseases (eg, Danon, Fabry disease, etc.).^717^	I	B

### 3.14. Chagas Heart Disease

Chagas heart disease (CHD) is the most common and severe expression of Chagas disease, a neglected tropical disease caused by the protozoan parasite *Trypanosoma cruzi* . The pathogenesis of CHD involves a complex interaction between the host and the parasite and is considered multifactorial. Moderate-to-severe inflammation is typically seen in biopsies, as well as microvascular changes, such as spasms, microthrombus formation, platelet activation, endothelial dysfunction, and decreased blood flow ("steal" phenomenon), which contribute to myocardial damage.^718 , 719^

CMR has become a key tool in the risk stratification and prognosis of patients with CHD. CMR features of CHD are described in Table 40 . In 2005, Rochitte et al. evaluated 51 patients at different stages of CHD and observed myocardial fibrosis in 68.6% of them, including some in the indeterminate phase. LGE was mostly observed on apical and inferolateral segments of the LV, with a primarily midwall and subepicardial pattern, and showed good correlation with established prognostic factors such as LVEF and NYHA class.^720^Subsequent studies have also described the presence of fibrosis in patients in the indeterminate phase,^721 – 724^as well as its pattern of involvement and impact on segmental function, notably on the inferolateral and apical segments of the LV.^722 , 723^RV systolic dysfunction is more commonly associated with LV systolic dysfunction, although isolated and early RV dysfunction can also be identified.^720^Some studies have found a significant prevalence of subendocardial LGE, which supports the hypothesis that myocardial involvement may be due to both ischemic (thromboembolic/microvascular) and nonischemic lesions (inflammatory).^721 , 722^

**Table 40:** Cardiac magnetic resonance imaging features of Chagas heart disease

Heterogeneous myocardial fibrosis (inflammatory/microvascular/ischemic)
Myocardial fibrosis on the inferolateral and apical walls
Global and/or segmental systolic dysfunction
Apical aneurysm ("finger in glove" sign)
Right ventricular dysfunction
Chronic myocardial inflammation
Intracardiac thrombi

T1 mapping and ECV have been shown to be useful in the evaluation of CHD. ECV showed an AUC similar to that of LGE in predicting nonsustained ventricular tachycardia.^725^In another study, the degree of fibrosis was the main predictor of ventricular tachycardia.^726^

In chronic CHD, myocardial inflammation was evaluated by T2-weighted spin-echo sequences, presence of edema,^723^and early gadolinium enhancement. Confirmation of active inflammation in chronic CHD may have important therapeutic implications, such as more effective patient selection for cause-specific treatment and the potential use of immunomodulatory therapy.

CHD is an independent risk factor for stroke regardless of ventricular function and the presence of cardiac arrhythmias.^727 , 728^Systolic dysfunction, segmental defects, apical aneurysms, fibrosis, and myocardial edema are associated with increased risk of intracardiac thrombus and are commonly described in patients with chronic CHD. In a recent study on cryptogenic stroke, CMR identified abnormalities with embolic potential in more than a quarter of patients, leading to changes in anticoagulant therapy management, with nearly half of these patients having chronic CHD.^729^

The prognostic evaluation of CMR in chronic CHD was initially described in relation to the presence and extent of myocardial fibrosis with established risk factors (NYHA class, LVEF) and later with the Rassi score.^721 , 724^Recent studies have shown that the presence and extent of myocardial fibrosis are strongly associated with MACE.^730 , 731^

A suggested diagnostic flowchart for patients with CHD is illustrated in Figure 7 .

**Figure 7:** Suggested diagnostic flowchart for patients with Chagas heart disease. *The dashed arrow represents a conditional recommendation. ECG: electrocardiography; ECHO: echocardiography; LV: left ventricular; MRI: magnetic resonance imaging; RV: right ventricular.

Table 41 presents the main recommendations for the use of CMR in the diagnosis and prognostic stratification of patients with CHD.

**Table 41:** Recommendations for the use of cardiac magnetic resonance imaging in the evaluation of Chagas heart disease (CHD)

Indication	Class of recommendation	Level of evidence
Assessment of biventricular systolic function (global and segmental) in patients with CHD.^732 , 733^	IIa	B
Investigation of intracardiac thrombi in patients with CHD and cryptogenic stroke.^734 , 735^	I	B
Investigation of fibrosis and/or myocardial edema in patients in the indeterminate phase.^720 , 730 , 736^	IIa	C
Evaluation of myocardial fibrosis for risk stratification of patients with CHD.^724 , 730 , 731^	I	B

### 3.15. Arrhythmogenic Right Ventricular Dysplasia/Cardiomyopathy

Arrhythmogenic RV dysplasia/cardiomyopathy (ARVD/C) is a disease that primarily affects the RV and is associated with ventricular arrhythmias, which sometimes may be fatal.^737 , 738^ARVD/C is not solely confined to the RV; evidence indicates that the LV may also be affected and, in some cases, may even be the predominant site of involvement.^739^

ARVD/C may account for up to 10% of sudden death cases in young patients.^740^The most common symptoms are palpitations and syncope and typically manifest between the second and fifth decades of life.^737 , 738^Desmosomes, which are multiprotein complexes that mediate cell-cell contact and signaling processes, are potentially involved in the etiology of ARVD/C.^737 , 741 , 742^With accurate diagnosis and appropriate treatment, including the consideration of an ICD, the mortality rate becomes relatively low.^737^

Characteristic morphofunctional changes in ARVD/C include increased RV volume with focal dilations (also called aneurysms) along the anterior wall of the infundibulum, the subvalvular inferior wall, or the lateral apical region, collectively referred to as the "triangle of dysplasia."^737 , 742^There appears to be a gradual loss of myocytes in these regions, replaced by adipocytes and fibroblasts, together with variable lymphocytic infiltration.

These changes are supported by CMR findings, which include akinetic segments in the RV, increased RV volume, and RV outflow tract (RVOT) enlargement. Initially, fat infiltration in the RV free wall was considered a pathognomonic sign.^743^However, fat infiltration has low interobserver agreement (kappa = 0.74) and is no longer considered a diagnostic criterion for the disease.^744^Sensitivity of fat infiltration, RV enlargement, and regional RV dysfunction for diagnosing ARVD/C was 84%, 68%, and 78%, and specificity was 79%, 96%, and 94%, respectively.^744^

In the early, or "concealed," phase of the disease, minimal fibrosis with negative LGE on CMR may be present, and these patients may experience sustained ventricular arrhythmias with a potential risk of sudden death.^745^The demonstration of fibrofatty changes in both ventricles via LGE imaging is promising.^746^

In 1994, an International Task Force proposed criteria for the diagnosis of ARVD/C.^747^However, due to limited knowledge about ARVD/C at the time, positive cases were considered mainly those that were symptomatic or those in which sudden death occurred—the most severe presentation of the disease. A new revision of the diagnostic criteria was published in 2010, based on new genetic insights from comprehensive studies of affected family members.^748^According to this revision, CMR can provide valuable information about RV contractility and volume, which can constitute either a **major criterion** for diagnosing ARVD/C (regional akinesia, dyskinesia, or dyssynchronous contraction of the RV associated with an indexed RV end-diastolic volume ≥ 110 mL/m² in men or ≥ 100 mL/m² in women, or an RVEF ≤ 40%) or a **minor criterium** (regional akinesia, dyskinesia, or dyssynchronous contraction of the RV associated with an indexed RV end-diastolic volume between 100 and 110 mL/m² in men or between 90 and 100 mL/m² in women, or an RVEF between 40 and 45%).

Recently, an expert consensus known as the Padua Criteria expanded the definition of ARVD/C to include a broader spectrum of "arrhythmogenic cardiomyopathy," including left-dominant and biventricular phenotypes. In this new approach, CMR has emerged as the imaging modality of choice due to its detailed morphofunctional analysis capabilities and biventricular tissue characterization. Like the Task Force criteria, the Padua Criteria also need to be validated in clinical studies with large patient cohorts and do not necessarily replace the previous criteria.^749^

Table 42 presents the main recommendations for the use of CMR in ARVD/C.

**Table 42:** Recommendations for the use of cardiac magnetic resonance imaging in the evaluation of suspected arrhythmogenic cardiomyopathy (ARVD/C) and risk stratification

Indication	Class of recommendation	Level of evidence
Evaluation of patients with suspected ARVD/C.^746 , 747 , 750 , 751^	I	B
Risk stratification in patients with ventricular arrhythmia and/or electrocardiographic abnormalities with suspected ARVD/C.^752 – 754^	I	B
Evaluation of left ventricular involvement in patients with suspected or confirmed ARVD/C.^750 , 755 – 757^	I	B
Assessment of right ventricular function and volume.^746 , 747 , 750^	I	B

### 3.16. Sarcoidosis

Sarcoidosis is a complex, multisystem granulomatous disease of unknown etiology and heterogeneous presentation. It is characterized by the formation of noncaseating granulomas in virtually any organ, although the lungs and mediastinal lymph nodes are the primary targets. Clinically manifest cardiac involvement has been reported in 5% to 10% of patients.^758 , 759^However, myocardial granulomas have been described in 25% to 58% of autopsy studies,^760 – 763^with cardiopulmonary involvement accounting for most of the morbidity and mortality associated with this disease.

The diagnosis of CS is challenging because it presents nonspecific cardiac manifestations that depend on location, extent, and stage of the inflammatory process, with greater involvement resulting in more symptoms. The most common cardiac manifestations are arrhythmias, including atrioventricular blocks, supraventricular arrhythmias, and ventricular tachyarrhythmias, which can occasionally lead to sudden death. HF may be the first manifestation if myocardial involvement is extensive, accounting for up to 25% CS-related mortality.^764^

EMB is considered the gold standard for the diagnosis of CS, but the reported accuracy of unguided myocardial biopsy is low (25%). Guided biopsy procedures increase the success rate to almost 50%, including in symptomatic patients, and are recommended by consensus guideline.^764^In the absence of confirmatory tissue diagnosis, clinical guidelines recommend combining proof of extracardiac disease with evidence of cardiac involvement (particularly through imaging biomarkers), although they lack supportive scientific data ( Table 43 ). Consequently, patients may either present with established extracardiac disease, with or without cardiac involvement, or no systemic involvement.

**Table 43:** Recommendations for the use of cardiac magnetic resonance imaging in the assessment of sarcoidosis with suspected cardiac involvement

Indication	Class of recommendation	Level of evidence
Evaluation of suspected cardiac involvement in patients with extracardiac sarcoidosis.^768 – 770^	I	B
Evaluation of myocardial fibrosis in patients with sarcoidosis with suspected cardiac involvement (ventricular arrhythmias, atrioventricular blocks, etc.).^768 , 769 , 771 , 772^	I	B

CMR findings depend on the stage of disease. The acute inflammatory phase is characterized by myocardial wall thickening with contractility abnormalities, increased T2 signal (edema), and LGE, while the chronic phase demonstrates focal areas of myocardial thinning and LGE, typically in the basal septum. LGE may be linear subepicardial, transmural, or nodular, with a heterogeneous distribution and a typically nonvascular pattern. LGE involving the RV septum and septal insertion points is also a classic sign of CS.

Table 43 shows the main recommendations for CMR in suspected sarcoidosis.

### 3.17. Left Ventricular Non-compaction/Excessive Trabeculation of the Left Ventricle

LV noncompaction cardiomyopathy (LVNC) results from failure of the embryonic myocardial compaction process, leading to changes in the ventricular wall anatomy consisting of excessive trabeculations and deep recesses. Clinical presentation includes symptoms of HF, arrhythmias, and thromboembolic events.^773^The diagnosis of LVNC is challenging and controversial, partly because myocardial trabeculation is present in a significant portion of the healthy population and in other cardiomyopathies and physiological conditions, such as pregnancy.^774^

CMR has emerged as a promising tool in the diagnosis of LVNC. The first widely recognized diagnostic criterion was published by Pettersen,^775^which assigned a value of 2.3 for the ratio of noncompacted to compacted myocardium (NC/C) in diastole. Later publications introduced criteria such as trabeculated LV mass > 20% and NC/C > 2.0 in systole,^776^with the latter showing a better correlation with the occurrence of future cardiovascular events. Some studies also demonstrated the presence of LGE in patients with LVNC, although with a heterogeneous pattern.^777^LGE, combined with depressed EF, provides excellent prognostic correlation.^778^

Recently, an expert panel recommended replacing the term "left ventricular noncompaction" with "excessive trabeculation" of the LV. This recommendation stems from a lack of evidence supporting the embryonic mechanism of myocardial "compaction". In this context, excessive trabeculation should be interpreted as a phenotypic manifestation of certain physiological conditions (eg, pregnancy, physiological adaptations to exercise), as well as congenital or acquired heart diseases, and is not necessarily linked to adverse prognostic outcomes in adults.

Table 44 presents the recommendations for the use of CMR in suspected LVNC/excessive trabeculation of the left ventricle.

**Table 44:** Recommendations for the use of cardiac magnetic resonance imaging in the assessment of suspected left ventricular non-compaction/excessive trabeculation of the left ventricle

Indication	Class of recommendation	Level of evidence
Evaluation of suspected left ventricular non-compaction/excessive trabeculation of the left ventricle.^773 , 779 – 781^	I	B

### 3.18. Muscular Dystrophy

Muscular dystrophy (MD) comprises a group of genetic diseases that affect muscle function. They are caused by mutations in genes encoding several proteins essential for muscle contraction, leading to muscle weakness and progressive loss of function, and may also affect the cardiac muscle and conduction system.

MDs commonly associated with cardiac involvement include dystrophinopathies (Duchenne [DMD], Becker [BMD], and carriers of dystrophin gene mutations), Limb-Girdle, Emery-Dreifuss, and myotonic dystrophy. DMD is the most common muscular dystrophy.^782 – 785^

Cardiac involvement in dystrophinopathies is frequent and insidious,^786 , 787^affecting approximately 80% of patients.^788^As overall survival has increased due to improved ventilatory support (noninvasive mechanical ventilation), spinal stabilization surgery, and corticosteroid treatment, cardiac involvement (HF and arrhythmia) is currently one of the main causes of mortality in DMD.^789 , 790^TTE is the most frequently used test to diagnose cardiac involvement in DMD. However, it is significantly limited by poor acoustic windows due to scoliosis and obesity in these patients and has limited capacity to diagnose subclinical cardiac involvement.^791^The assessment of cardiac involvement in MD is primarily based on cine CMR images, and particularly on the identification of myocardial LGE, which typically has a subepicardial pattern and mostly affects the anterolateral and inferolateral segments of the LV.^792 – 798^

Early and subclinical diagnosis of cardiac involvement related to MDs (DMD and BMD) is crucial for providing adequate cardioprotection with the aim of reducing the adverse effects of cardiac remodeling and alleviate HF symptoms, thus reducing mortality rates.^799 – 801^

DMD and BMD consensuses and updates recommend that cardiac evaluation be performed from the moment these mutations are identified.^791 , 802 – 804^The National Heart, Lung, and Blood Institute (NHLBI) published an update on cardiac involvement in DMD in which CMR is considered the noninvasive method of choice for early diagnosis of cardiac involvement, except in young patients who cannot cooperate with the maneuvers required for the examination. In patients aged up to 6-7 years, echocardiography should be performed instead of CMR, as it does not require sedation. After this age, CMR should be performed every 2 years and annually after age 10.^803^Female carriers of DMD and BMD mutations should be evaluated if they show any symptoms of cardiac involvement. At around 40 years of age, women tend to show a decrease in EF and evidence of myocardial fibrosis.^805^

In 2017, the AHA^806^published guidelines for the management of cardiac involvement in neuromuscular diseases. However, there is no consensus on the use of CMR in the diagnosis of Limb-Girdle, Emery-Dreifuss, and myotonic dystrophies. Its use should be individualized based on systemic disease progression and the manifestation of CVD.

Table 45 presents the recommendations for the use of CMR in the management of cardiac involvement in MDs.

**Table 45:** Recommendations for the use of cardiac magnetic resonance imaging in carriers of Duchenne or Becker muscular dystrophy

Indication	Class of recommendation	Level of evidence
Annual evaluation in symptomatic patients.^806^	I	B
Evaluation of ventricular function in patients with poor acoustic windows or for investigation of myocardial fibrosis.^797 , 801 , 806^	IIa	B
Evaluation of myocardial fibrosis in asymptomatic patients with left ventricular dysfunction or dilation.^797 , 801^	IIa	B
Evaluation of asymptomatic patients every 2 years until age 10 and then annually (recommended time interval remains controversial).^806^	IIa	B
Evaluation of myocardial fibrosis for risk stratification and indication of angiotensin-converting enzyme inhibitors in patients with Duchenne/Becker muscular dystrophy with preserved left ventricular function.^801^	I	B

### 3.19. Peripartum Cardiomyopathy

Peripartum cardiomyopathy (PPCM) is a rare form of HF that occurs in the third trimester of pregnancy or up to 5 months postpartum. It is characterized by LV dilation and systolic dysfunction (LVEF < 45%) and significant morbidity and mortality. PPCM is a diagnosis of exclusion in women without known prior heart disease.^720^Its incidence ranges from 1:300 to 1:4000 live births, depending on the country and demographics.^807 , 808^

CMR is considered safe during pregnancy; however, the use of paramagnetic contrast (gadolinium) and its effects on the fetus are not well established. Therefore, administering contrast during pregnancy is not recommended, especially in the first trimester. Conversely, there is no restriction on the use of contrast during breastfeeding.^809 , 810^CMR is the method of choice for evaluating ventricular volumes and function, which are independent predictors of events in PPCM.^811 , 812^It may also be useful for excluding other etiologies, such as myocarditis, infiltrative diseases, and left ventricular non-compaction/excessive trabeculation of the left ventricle.^813^The pattern of LGE in PPCM is typically midmural, with a prevalence ranging from 5% to 70% (likely due to different intervals between symptom onset and CMR).^814 – 816^

Table 46 presents the recommendations for the use of CMR in the diagnosis and management of PPCM.

**Table 46:** Recommendations for the use of cardiac magnetic resonance imaging in the assessment of peripartum cardiomyopathy

Indication	Class of recommendation	Level of evidence
Evaluation of ventricular dysfunction without an established diagnosis.^817 – 819^	IIa	C
Prognostic evaluation (right ventricular involvement and assessment of late gadolinium enhancement postpartum).^812 , 814 , 817 , 820^	IIa	C

### 3.20. Cardiomyopathy Related to Systemic Diseases

Several systemic diseases affect the cardiovascular system, with varied clinical presentations and challenging clinical settings. Cardiovascular manifestations in systemic diseases are often subtle. Notable among these are autoimmune disorders such as rheumatoid arthritis and spondyloarthropathies, systemic lupus erythematosus, scleroderma, systemic vasculitides such as granulomatosis with polyangiitis, mixed connective tissue disease, and other inflammatory myopathies.^821^The mechanisms leading to myocardial damage are not well understood, but are known to involve factors such as intracellular and extracellular interactions, genetic mutations, autoimmune reactions, and inflammatory modulators.^822^Clinical presentation is widely diverse, and lesions may involve the myocardium, valves, pericardium, conduction system, and vessels. Additionally, there is an increased risk of atherosclerosis in rheumatic diseases, not solely attributed to traditional cardiovascular risk factors but also likely related to dysfunctional immune responses and chronic inflammation, accounting for increased morbidity and mortality in more advanced cases.^822^

The role of CMR in systemic diseases involves assessing the specific presentation of each case, but the use of LGE to detect irreversible myocardial damage has also been demonstrated.^823^Direct myocardial injury with inflammatory infiltrate has been demonstrated in polyangiitis,^824^and ischemia in the absence of obstructive CAD has been demonstrated in systemic lupus erythematosus^825^and T1 mapping analysis, including in the study of the pericardium.^826 , 827^A recent consensus^826^discussed the potential of CMR in the evaluation of patients with systemic diseases, such as in the early identification of myocardial lesions, definition of their nature (ischemic or inflammatory), and detection of arrhythmogenic substrates.

Table 47 presents the recommendations for the use of CMR in the diagnosis of cardiomyopathy associated with systemic diseases.

**Table 47:** Recommendations for the use of cardiac magnetic resonance imaging in the diagnosis of cardiomyopathy associated with systemic diseases

Indication	Class of recommendation	Level of evidence
Investigation of myocardial fibrosis in patients with ventricular dysfunction and autoimmune disease (rheumatoid arthritis, systemic lupus erythematosus, scleroderma).^826 , 828 – 830^	I	B
Investigation of suspected myocarditis/pericarditis secondary to autoimmune inflammatory activity.^830 – 834^	I	B

### 3.21. Cardiac Alterations Associated with Heart Transplantation

Cardiac allograft vasculopathy is the leading cause of late morbidity and mortality after heart transplantation, occurring in up to 50% of transplant recipients after 10 years. Although the gold standard for diagnosis is invasive coronary angiography, CMR has shown promising results.^835^

Monitoring cellular and humoral rejection, especially in the first year after heart transplantation, and actively investigating late complications such as cardiac allograft vasculopathy is crucial in the follow-up of transplant patients. The primary diagnostic method and gold standard is EMB. However, it is an invasive method and therefore not free from complications. In addition, inflammatory infiltration of the heart may not be uniform, making the identification of graft rejection by EMB more challenging.^835 , 836^Therefore, noninvasive diagnostic tests continue to evolve in attempts to detect graft rejection at an increasingly earlier stage.^822^

CMR can identify ventricular dysfunction and allows for better assessment of the RV, whose dysfunction may occur in the postoperative period of heart transplantation. Although ventricular dysfunction is present in cases of graft rejection, it is usually detected only in more advanced stages, probably when some degree of myocardial damage already exists.

Acute cellular rejection can be detected using tissue characterization by CMR, which can show inflammation/edema and myocardial necrosis/fibrosis. Myocardial edema can be assessed by T2-weighted imaging or directly by quantitative T2 mapping. Taylor et al. showed that a combined CMR criteria for acute rejection including myocardial edema assessed by T2-weighted images (relative signal > 2) or early enhancement (after gadolinium administration) had a sensitivity of 100% and a specificity of 73% compared with EMB and could identify significant (grade ≥ 2R) rejection.^837^Retrospective studies using T2 mapping described a high negative predictive value (97%) for detecting acute rejection ≥ 2R with T2 values ≥ 56 ms and a relative risk greater than 2 for rejection with T2 > 60 ms. The association of T2 > 59 ms with the RV end-diastolic volume index showed a negative predictive value of 98% for significant rejection ≥ 2R.^821 , 823 , 833 , 838^In a prospective study with a pediatric population, Ide et al. demonstrated that native T1 time and ECV correlate with histologically determined collagen volume, with accelerated myocardial fibrotic remodeling in children.^824 , 825 , 839^

In recent years, there has been a shift from qualitative to quantitative CMR evaluation, using the detailed data described above in combination. This can be observed in recent studies^825 – 827^showing the combined use of T2 and ECV as a potential biomarker for detecting acute cellular rejection.

Table 48 presents the main recommendations for the use of CMR in the evaluation of cardiac alterations associated with heart transplantation.

**Table 48:** Recommendations for the use of cardiac magnetic resonance imaging in the evaluation of cardiac alterations associated with heart transplantation

Indication	Class of recommendation	Level of evidence
Worsening of ventricular function without evidence of cardiac allograft vasculopathy.^840^	IIa	B
Worsening of ventricular function and negative/inconclusive endomyocardial biopsy.^840^	IIa	B

### 3.22. Pericardial Disease

Pericardial diseases are relatively common in clinical practice and may present either as an isolated process or in association with various systemic disorders.^340 , 343 , 841^Traditionally, echocardiography has been the method of choice in patients with suspected pericardial disease and, most of the times, it is the only imaging modality needed.^343 , 841 , 842^However, CT and CMR are increasingly used as part of a multimodality imaging approach.^340 , 342^

#### 3.22.1. The Role of CMR

CMR is primarily indicated in cases where echocardiography is limited by poor acoustic windows or is inconclusive, allowing for a more accurate evaluation of the pericardium.^342^It is also indicated for the follow-up of patients with more complex pericardial disease and contraindications to echocardiography, as CMR does not involve ionizing radiation or the use of iodinated contrast agents.^341^

CMR employs specific sequences for morphological imaging of pericardial structures and tissue characterization, along with functional imaging to measure ventricular function and intracardiac flows, allowing the assessment of the hemodynamic effects of pericardial diseases.^340 , 341 , 843^

#### 3.22.2. Pericardial Effusion

Pericardial effusion refers to the accumulation of more than 50 mL of fluid in the pericardial sac, resulting from disorders affecting the pericardium directly or as a response to systemic disease. This fluid may include transudate, exudate, pus (pyopericardium), blood (hemopericardium), or lymphatic fluid (chylopericardium).^340 , 844^

The role of imaging is to confirm the presence of pericardial effusion, estimate its amount, describe its distribution, and determine any hemodynamic effect on the heart.^844^CMR provides a more accurate definition of the distribution and amount of pericardial fluid than echocardiography.^342^Targeted imaging sequences help to characterize the contents of the pericardial sac—fat, fluid with varying protein content—and determine the best treatment approach.^340^Transudative effusions typically have low signal on T1-weighted and high signal on T2-weighted CMR images. Exudative effusions appear heterogeneous with intermediate signal (tending toward high) on T1-weighted and T2-weighted CMR images. Hemorrhagic effusions will have the same signal intensity of that of blood products.^344^

#### 3.22.3. Acute Pericarditis

CMR should be considered when echocardiographic findings are inconclusive, there is nonresponse to treatment, and the clinical presentation is atypical. CMR is well indicated especially in suspected constrictive pericarditis—allowing for the evaluation of hemodynamic compromise—and post-myocardial infarction free-wall rupture when there are still doubts regarding the presence of hemopericardium.^344^

#### 3.22.4. Pericardial Tamponade

CMR is not indicated for cardiac tamponade, a condition associated with hemodynamic instability, whose diagnosis is primarily clinical and easily evaluated by echocardiography. However, in cases where the presence of cardiac tamponade is still uncertain, CMR is a useful complementary test to exclude localized or loculated tamponade. The CMR criteria for a functionally important effusion are similar to that of echocardiography: diastolic collapse of the RV free wall, right atrium (RA) collapse, LV and RV morphology abnormalities, and abnormal ventricular septal motion to the left during early inspiration (ventricular interdependence), although this is more common in constrictive pericarditis.^341^

#### 3.22.5. Constrictive Pericarditis

Constrictive pericarditis (CP) is a condition caused by a thick noncompliant inflamed, fibrotic, and/or calcified pericardium, which inhibits cardiac filling by preventing full transmission of respiratory intrathoracic pressure changes to cardiac cavities.^341^

In patients with suspected CP, imaging studies aim to obtain diagnostic information such as pericardial thickness, ventricular interdependence, associated abnormalities (valvular, myocardial, or CAD), and evidence of differential diagnoses such as restrictive cardiomyopathy, RV dysfunction, or severe tricuspid regurgitation. Although echocardiography is the initial imaging method of choice for CP in most patients, CMR can be helpful in the establishment of a definitive diagnosis.^342^

CMR can detect hemodynamic signs of constriction, similar to those observed in echocardiography, especially with real-time cine imaging.^845^Pericardial thickness on CMR (> 4 mm) in the appropriate clinical context often supports the diagnosis of CP, although the absence of pericardial thickening does not necessarily exclude it.^341^

Tagged cine CMR imaging can be used to detect pericardial adhesions with reduced mobility of the myocardium.^342^CMR is also valuable for assessing the extent of pericardial inflammation by detecting pericardial edema and/or LGE, although this finding is not universal.^342^

#### 3.22.6. Pericardial Tumors

Pericardial tumors are very rare and divided into primary (benign or malignant) and secondary (metastatic).

Primary benign neoplasms of the pericardium are slow-growing tumors with well-defined borders, often detected incidentally,^340^whose hemodynamic effects may cause significant cardiovascular complications.^343^The most common benign pericardial tumor is lipoma, but teratoma, fibroma, hemangioma, lymphangioma, paraganglioma, and granular cell myoblastoma are commonly found as well.^340 – 342^

Primary malignant pericardial neoplasms are also rare and include mesothelioma (the most common), sarcoma (the second most common), lymphoma, liposarcoma, malignant teratoma, and hemangioendothelioma.^340^Most primary malignant tumors are not resectable at diagnosis. Chemotherapy and radiotherapy offer limited benefits.^342^

Metastatic pericardial tumors are more common than primary pericardial neoplasms and tend to occur late in the disease process. Most pericardial tumors are secondary to local invasion from lung and mediastinal tumors or metastases from lung and breast cancers, lymphomas, and melanoma.^841^

CMR provides better assessment of pericardial mass size, involvement of adjacent structures, and potential hemodynamic complications.^342^Although CMR is useful for differentiating between benign and malignant pericardial tumors,^343^biopsy and histopathological analysis are necessary to achieve a definitive diagnosis for most pericardial tumors.^340^

Well-defined or encapsulated pericardial lesions without pericardial irregularity or effusions are more likely to be benign.^342^Pericardial tethering and/or direct invasion of adjacent structures are generally indications of malignancy, and dynamic tagging may help identify such physical connections. Pericardial metastases may manifest on CMR as irregular or nodular pericardial thickening, nodules, or masses,^340^which in turn tend to present as multiple enhancing and coalescing pericardial masses usually with a large associated exudative effusion.^841^

Most neoplasms exhibit low signal intensity on T1-weighted and high signal intensity on T2-weighted CMR images. In first-pass perfusion, primary and secondary pericardial tumors—predominantly malignant—show some degree of gadolinium enhancement. Because of their increased vascularity, pericardial neoplasms usually enhance heterogeneously after contrast administration. Hyperintense signal is observed mainly in highly vascularized malignant tumors.^340 , 342^

#### 3.22.7. Pericardial Cysts and Diverticula

Pericardial cysts are typically located adjacent to the heart, particularly in the right cardiophrenic angle.^841 , 844^Pericardial diverticula are differentiated from pericardial cysts by the presence of communication with the pericardial space.^343^

Pericardial cysts almost always exhibit low and homogeneous intensity on T1-weighted and high and homogeneous intensity on T2-weighted images. They do not enhance with gadolinium-based contrast administration. Very rarely, pericardial cysts may contain highly proteinaceous fluid, leading to high signal intensity on T1-weighted images.^342 , 841^

#### 3.22.8. Pericardial Abscess

Pericardial abscess is a walled off collection of pus within the pericardial space, appearing as a localized biconvex collection within the pericardial space compressing the adjacent cardiac chambers. The core may be better demonstrated on CMR, appearing isointense to hyperintense on both T1- and T2-weighted images.^841^

#### 3.22.9. Hematomas

CMR is particularly useful in the diagnosis of pericardial hematomas, which have a characteristic signal intensity on T1-weighted and T2-weighted images. Acute hematomas exhibit homogeneous high signal intensity, while subacute (1-4 weeks old) hematomas typically demonstrate heterogeneous signal intensity, with areas of high signal intensity on both T1-weighted and T2-weighted images. Particularly on T1-weighted images, chronic organized hematomas may show a dark peripheral rim and low-signal-intensity internal foci that may represent fibrosis, calcification, or hemosiderin deposition. Hematomas do not enhance after contrast administration.^845^

#### 3.22.10. Congenital Absence of Pericardium

Congenital absence of pericardium (CAP) is a rare condition caused by abnormal embryological development, which can manifest in isolation or be associated with other congenital disorders such as bicuspid aortic valve, patent ductus arteriosus (PDA), mitral valve stenosis, atrial septal defect (ASD), and tetralogy of Fallot.^845^

On CMR, the pericardium can be identified because it is differentiated from the adjacent myocardium by the presence of epicardial and pericardial fat layers, which demonstrate different signal intensities than the epicardium. However, in patients with minimal epicardial and pericardial fat (younger, lean, athletic), differentiation becomes difficult because the area typically with less epicardial fat lies almost immediately on the most common site of pericardial defects (the lateral, posterior, and inferior LV walls), requiring caution to avoid false positives.^342^There are important indirect morphological and functional signs consistent with pericardial defects, including interposition of lung tissue between the aorta and pulmonary artery, leftward cardiac displacement, and excessive levorotation.^344 , 841^

Table 49 shows the main recommendations for the use of CMR in the evaluation of the pericardium.

**Table 49:** Recommendations for the use of cardiac magnetic resonance imaging in the assessment of the pericardium

Indication	Class of recommendation	Level of evidence
Evaluation of hemodynamic consequences of large pericardial effusion when echocardiography is inconclusive.^341 , 342 , 845^	IIa	B
Evaluation of acute pericarditis (< 3 months).^341 , 342 , 845^	IIa	B
Evaluation of chronic pericarditis (> 3 months).^341 , 342 , 845^	IIa	B
Evaluation of constrictive pericarditis in patients without suspected associated pericardial calcification.^341 , 342 , 845^	IIa	B
Evaluation of constrictive pericarditis in patients with suspected associated pericardial calcification.^341^	IIa	C
Investigation of congenital pericardial anomalies.^340 , 342 , 344 , 841^	I	B

### 3.23. Cardiac Masses and Thrombus

Cardiac masses are rare and include a variety of diagnoses, such as benign tumors, malignant tumors (primary and secondary), and tumor-like conditions such as thrombi, pericardial cysts, and vegetations.^846^TTE is the initial diagnostic method of choice because it is widely available, portable, and has low cost.^847^However, its ability to evaluate right heart chambers, mediastinal structures, and extracardiac structures is limited.

CMR is one of the main methods for evaluating these structures, as it provides data related to location, size, borders, tissue characterization, vascularization, involvement of adjacent structures, and cardiac function.^848^Different acquisition techniques allow the characterization of important histological components (eg, presence of fat, hemorrhagic, necrotic, melanotic, calcified, myxoid, and fluid content), as well as tissue vascularization. This information, combined with the location of the tumor ( Table 50 ), may help differentiate between non-neoplastic lesions (thrombi, cysts, and vegetations) and benign or malignant tumors.^849^Regardless of its cellular characteristics, any cardiac tumor, even if histologically benign, may have substantial hemodynamic or arrhythmic consequences depending on its size and location.^850 , 851^

**Table 50:** Magnetic resonance imaging features of different cardiac tumors

CARDIAC MASS	LOCATION	CINE MRI	T1-WEIGHTED IMAGING	T2-WEIGHTED IMAGING	LGE
NON-NEOPLASTIC LESIONS					
THROMBUS	Left atrium (atrial fibrillation) Left ventricle (aneurysm) Right atrium (central venous catheter)	Hypo or isointense	Acute: hyperintense Chronic: hypointense	Hypointense or hyperintense	No uptake
CYST	Pericardial (right cardiophrenic angle)	Hyperintense	Hypointense	Hyperintense	No uptake
**BENIGN TUMORS**					
MYXOMA	Fossa ovalis (left atrium 80%, right atrium 20%)	Mobile lesion	Isointense, heterogeneous	Hyperintense	Heterogeneous enhancement
PAPILLARY FIBROELASTOMA	Cardiac valve (typically the left)	Mobile, hyperintense, peritumoral turbulent flow	Isointense	Isointense	Hyperenhancement
LIPOMA	Epicardial (70%), ventricles, interatrial septum	Dark border, hyperintense	Hyperintense	Hyperintense	No uptake
FIBROMA	Interventricular septum, left ventricular wall, right ventricle	Hypo or isointense	Iso/hyperintense	Hypointense	Hyperenhancement Homogeneous
RHABDOMYOMA	Ventricle and interventricular septum	Intramural mass, slightly hyperintense	Iso/hyperintense	Iso/hyperintense	No uptake or minimal uptake
HEMANGIOMA	Ventricle and interventricular septum	Hyperintense	Isointense	Hyperintense	Prolonged heterogeneous enhancement
**MALIGNANT TUMORS**					
MESOTHELIOMA	Pericardial (asbestos exposure or pleural disorders)	Hypointense nodule	Isointense	Heterogeneous and intense	Heterogeneous enhancement
ANGIOSARCOMA	Right atrium	Isointense and heterogeneous	Hyperintense Heterogeneous	Hyperintense Heterogeneous	Heterogeneous enhancement
RHABDOMYOSARCOMA	Any chamber	Isointense	Isointense	Isointense	Heterogeneous enhancement
LEIOMYOSARCOMA	Left atrium - posterior wall	Hypo or isointense	Isointense	Hyperintense	Nonspecific
SYNOVIAL SARCOMA	Pericardium Right atrium	Hypo or isointense	Isointense	Slightly hyperintense	Heterogeneous enhancement
LYMPHOMA	Right atrium	Isointense	Hypo/isointense	Slightly hyperintense	No uptake or minimal uptake
METASTASIS	Depends on site of involvement Pericardium	Depends on site of involvement	Hypointense	Hyperintense	Heterogeneous enhancement

The most common cardiac masses are non-neoplastic (eg, thrombi, vegetations, perivalvular abscesses, pericardial cysts, mitral annulus calcification), which are potentially mimicking cardiac tumors. Intracardiac thrombi are the most common cardiac masses, usually located in the LA due to AF or valvular heart disease, but may also be found in the LV with reduced EF.^852^MRI with first-pass perfusion allows clear differentiation of thrombus from surrounding myocardium because thrombus is avascular. Although the use of GBCAs increases the accuracy of CMR in identifying thrombi, they are characterized by the absence of contrast material uptake (including both early and late enhancement). Rarely, thrombi may enhance peripherally due to fibrous content.^570 , 853^

Primary cardiac tumors are rare (incidence rate < 0.3%), and more than 75% are benign.^854 , 855^Myxoma is the most frequent benign cardiac tumor in adults, accounting for approximately 50% of cases. It most commonly affects women, usually between the third and sixth decades of life. It is typically located in the LA as solitary, mobile nodules attached to the fossa ovalis.^856^Myxomas tend to present as isointense or heterogeneous on T1-weighted images and hyperintense or heterogeneous on T2-weighted images because of their high extracellular water content. About half of cardiac myxomas show heterogeneous enhancement.^850^

Lipomas are the second most common type of cardiac tumor (approximately 16%) and are mostly discovered incidentally because they are often asymptomatic. Lipomas tend to be well-circumscribed, homogeneous, and encapsulated and are typically located in the LV and RA. Because they are composed of adipose tissue, they appear hyperintense on T1-weighted images.^857^

Papillary fibroelastomas account for approximately 75% of all valvular neoplasms and 10% of primary cardiac tumors. They are usually solitary and small (less than 2 cm) and affect both sexes equally. They tend to occur more frequently in people aged 60 to 80 years, with embolic events being the main symptoms. They can arise from any endocardial surface, but are most commonly found on the atrial surface of the mitral valve and the aortic surface of the mitral valve leaflets.^858^They appear hypointense on cine images and isointense on T1- and T2-weighted images.

Rhabdomyoma is the most common cardiac tumor in children, accounting for approximately 90% of primary benign cardiac tumors in this age group, with 75% occurring in children under 1 year of age. Most lesions tend to regress spontaneously and are characterized by intermediate-to-high signal intensity on T1-weighted images and intermediate signal intensity on T2-weighted images, with absence of LGE after contrast administration. Other primary cardiac tumors include fibromas, intrapericardial paragangliomas, hemangiomas, and teratomas.^859 , 860^

Among primary malignant tumors, sarcomas are the most common. Among secondary tumors, the most common extracardiac neoplasms are lung, lymphoma, breast, and esophageal cancer. Cardiac metastasis is 20–40 times more common than primary cardiac tumors and may result from direct spread from adjacent structures (bronchogenic carcinoma, lymphoma), hematogenous seeding (lung and breast cancer, melanoma, lymphoma, and leukemia), lymphatic spread (most common route in lymphomas and rarer in carcinomas), or venous extension (hepatocellular, renal, endometrial, and thyroid carcinomas).^861^

Angiosarcoma is the most common histological subtype in adulthood among primary malignant cardiac tumors. It is a highly aggressive sarcoma that often originates in the RA and has a high likelihood of metastasis at the time of presentation. On T1-weighted images, angiosarcomas appear as isointense lesions with multiple nodular areas of high intensity.^357^LGE characteristics of the tumor show heterogeneous enhancement and may show a large necrotic core without enhancement,^862^as well as evidence of vascularization on first-pass perfusion imaging.

Rhabdomyosarcoma is the most common primary malignant cardiac tumor in childhood. It is typically large (> 10 cm in diameter), invasive, and may originate anywhere in the heart, but is more commonly found in the cardiac valves. On CMR, it demonstrates homogeneous and iso-to-hyperintense signal intensity on T1- and T2-weighted images, characteristic central necrosis,^863^and marked LGE.

Mesothelioma is a rare neoplasm that, in most cases, develops in the pericardium. It commonly affects older men with a history of asbestos exposure and pleural disorders. It usually presents with diffuse growth, multiple poorly defined masses within the pericardial cavity, causing extensive pericardial thickening and a large hemorrhagic pericardial effusion, potentially presenting signs and symptoms of CP or cardiac tamponade.^864^CMR shows a pericardial mass surrounded by the visceral and parietal pericardium appearing isointense on T1-weighted and heterogeneous on T2-weighted images.

Primary cardiac lymphomas are extremely rare, typical of non-Hodgkin lymphoma, and restricted to the heart and pericardium. They typically occur in immunocompromised patients in association with Epstein-Barr virus infection. On CMR, they are hypo/isointense on T1-weighted images and slightly hyperintense on T2-weighted images. They also show heterogeneous gadolinium uptake, with possible central hypointense areas.^849^

CMR provides useful information for differentiating between benign and malignant tumors. Tumor size, local invasion, increased vascularization, involvement of more than 1 cardiac chamber, and pericardial effusion are good indicators of malignancy. In addition to assisting in the characterization of histological type, CMR provides better detailing of the tumor and its involvement of extracardiac structures, which is essential for preoperative evaluation.^865^

Table 50 describes MRI features of different cardiac tumors.

Table 51 presents the main recommendations for CMR in the evaluation of cardiac masses/thrombi.

**Table 51:** Recommendations for the use of cardiac magnetic resonance imaging in the assessment of cardiac masses/thrombi

Indication	Class of recommendation	Level of evidence
Detection and characterization of cardiac and pericardial tumors.^419 , 850 , 866 – 868^	I	B
Detection and differential diagnosis of ventricular thrombi.^419 , 866 , 867 , 869^	I	B
Detection of atrial and atrial appendage thrombi.^870^	IIa	B
Follow-up of patients with benign cardiac tumors.^419 , 867^	I	C
Evaluation following tumor treatment (recurrence after resection, chemo/radiotherapy).^871 – 873^	I	C

### 3.24. Valvular Heart Disease

TTE is the first-line imaging modality for patients with valvular heart disease.^251 , 874 , 875^However, CMR offers significant advantages over echocardiography, including (1) free choice of imaging planes, unrestricted by acoustic windows,^876^(2) enhanced quantification of heart valve regurgitation,^875 , 877 – 880^(3) higher accuracy and reproducibility in the morphological and functional assessment of both ventricles, including quantification of ventricular volume, EF, and mass,^881 , 882^and (4) ability to provide myocardial tissue characterization.^418 , 883 , 884^Therefore, a growing number of patients with valvular heart disease benefit from the versatility of CMR as a complementary test to TTE.^875 , 885 , 886^

First, CMR assessment should include characterization of valvular morphology and structure, assessment of the stenotic and/or regurgitant jet, and, when possible, determination of the mechanism of dysfunction. In aortic valve disease, it is essential to assess for leaflet thickening, calcification, retraction, or fusion, determine the number of leaflets, characterize the stenotic or regurgitant jet (size, direction, eccentric vs central, single vs multiple), and investigate the presence of aortic root or ascending aorta dilation. In patients with mitral regurgitation, it is important to evaluate leaflet thickening, mitral annular calcification involving the subvalvular apparatus, mitral valve leaflet mobility (prolapse, flail, tethering) or perforation, and the location of regurgitation. It is crucial to determine the mechanism and etiology (primary, secondary, of mixed) of mitral regurgitation.^875^The same approach should be applied to the evaluation of tricuspid and pulmonary regurgitation.

The second step involves assessing the severity of the valvular lesion. The main CMR methods for grading such lesions are:

- Stenotic lesions:
  1. Aortic stenosis. The 2 main parameters measured by CMR to grade the severity of aortic stenosis are (1) maximum velocity of the aortic stenosis jet (Vmax) measured by phase-contrast; and (2) aortic valve area (AVA) measured by direct planimetry. For both Vmax and AVA, the measurement plane should be perpendicular to the aortic stenosis jet.
  2. Pulmonary stenosis. The same methods described for aortic stenosis are used for evaluating pulmonary stenosis, ie, quantification of the maximum velocity of the pulmonary stenosis jet (Vmax) and measurement of pulmonary valve area by direct planimetry.
  3. Mitral and tricuspid stenosis. In general, these valvular lesions can be adequately evaluated by echocardiography. However, in cases where echocardiography is limited, CMR can assess the severity of mitral or tricuspid stenosis using the same principles described above: measurement of Vmax by phase-contrast technique and valve area by direct planimetry.
- Regurgitant lesions.^878 , 879 , 887 – 889^
  1. Aortic regurgitation. There are 3 main CMR parameters used to grade the severity of aortic regurgitation: (1) regurgitant volume (diastolic forward flow in the aorta) measured by phase-contrast in a plane perpendicular to the ascending aorta between the aortic valve plane and the sinotubular junction plane. Regurgitant fraction is calculated as the regurgitant volume divided by the total aortic forward stroke volume in systole.^878^(2) In the absence of other concomitant regurgitant lesions (trivial or mild lesions can be disregarded), the aortic regurgitant volume may be measured as the difference between LV stroke volume and RV stroke volume measured by cine imaging using Simpson's method. In this case, the regurgitant fraction may be calculated as the regurgitant volume divided by the LV stroke volume. (3) Aortic regurgitant orifice area measured by direct planimetry.
  2. Mitral regurgitation. There are 3 main CMR parameters used to grade the severity of mitral regurgitation: (1) quantification of regurgitant volume measured as the difference between LV stroke volume by cine MRI using Simpson's method and the aortic forward stroke volume in systole by phase-contrast. Regurgitant fraction is calculated as the regurgitant volume divided by the total LV stroke volume.^880 , 886^(2) In the absence of other concomitant regurgitant lesions (trivial or mild lesions can be disregarded), the mitral regurgitant volume can be measured as the difference between the LV and RV stroke volumes obtained by cine MRI imaging using Simpson's method. In this case, the regurgitant fraction may be calculated as the regurgitant volume divided by the LV stroke volume. (3) Mitral regurgitant orifice area measured by direct planimetry.
  3. Pulmonary regurgitation. The assessment of pulmonary regurgitation by CMR follows the same strategy as that of aortic regurgitation: (1) regurgitant volume/fraction measured as the difference between antegrade stroke volume and retrograde stroke volume measured by phase-contrast in the pulmonary trunk. (2) In the absence of other concomitant regurgitant lesions (trivial or mild lesions can be disregarded), the pulmonary regurgitant volume/fraction may be measured as the difference between the LV and RV stroke volumes obtained by cine MRI imaging using Simpson's method. (3) Pulmonary regurgitant orifice area measured by direct planimetry.
  4. Tricuspid regurgitation. The assessment of tricuspid regurgitation by CMR follows the same strategy as that of mitral regurgitation: (1) regurgitant volume/fraction measured as the difference between RV stroke volume by cine MRI using Simpson's method and the anterograde volume in the pulmonary artery in systole by phase-contrast. (2) In the absence of other concomitant regurgitant lesions (trivial or mild lesions can be disregarded), the tricuspid regurgitant volume/fraction can be measured as the difference between the LV and RV stroke volumes obtained by cine MRI imaging using Simpson's method. (3) Tricuspid regurgitant orifice area measured by direct planimetry.

Specific cutoff values for CMR have been suggested to classify valvular lesions as mild, moderate, or severe.^876^However, there is still a dearth of studies validating specific data for CMR, so cutoff values derived from echocardiography are more commonly used.^874^

The third step involves assessing the morphology and function of both ventricles and atria. As previously mentioned, CMR is more accurate and reproducible than echocardiography in this type of evaluation,^880 , 881^making it especially useful in the longitudinal follow-up of patients with valvular heart disease. The quantification of ventricular volumes and function allows to assess the associated remodeling of cardiac chambers in response to volume overload. This information is important not only because it provides additional data to help assess the severity of valvular lesions^875^but also because it constitutes a parameter to help guide the therapeutic approach, including the optimal timing for surgical or percutaneous intervention.^251 , 874 , 885 , 889^

A unique contribution of CMR in the evaluation of patients with valvular heart disease is its ability to provide myocardial tissue characterization.^418 , 883 , 884^Several studies have shown that assessing the presence, extent, and pattern of myocardial LGE provides important prognostic information^890 , 891^that can assist in the management of patients with aortic and/or mitral valve disease.^892 – 897^More recently, some studies showed that T1 mapping techniques^883 , 898^also provide valuable prognostic information and may be used complementary to LGE imaging.^876 , 895^

Finally, it is worth noting that CMR may be used in the evaluation of patients with either biological or mechanical prosthetic heart valves.^876^The presence and extent of susceptibility artifacts depend on the amount of ferromagnetic material in the prosthesis. In cases where susceptibility artifacts are absent or minimal, it is possible to evaluate the structure of the prosthesis and the mobility of the leaflets. However, in cases with extensive susceptibility artifacts, such evaluation becomes challenging or impossible. Nonetheless, in most cases, it is possible to quantitatively evaluate stenotic lesions (by measuring the Vmax of the stenotic jet in a plane immediately distal to the artifact region) and regurgitant lesions (using the same techniques described above for the quantitative evaluation of native valves).

Table 52 presents the recommendations for the use of CMR in the evaluation of valvular heart disease.

**Table 52:** Recommendations for the use of cardiac magnetic resonance imaging in the assessment of valvular heart disease

Indication	Class of recommendation	Level of evidence
Evaluation of ventricular anatomy and function.^251 , 419^	I	A
Quantitative evaluation of regurgitant lesions complementary to echocardiography.^212 , 251 , 874 , 886^	IIa	B
Quantification of the severity of primary mitral regurgitation in patients scheduled for transcatheter valve replacement or heart valve repair.^212 , 251 , 874 , 880 , 886^	IIa	B
Evaluation of stenotic lesions complementary to echocardiography.^251 , 419 , 874 , 899 , 900^	IIa	B
Investigation of myocardial fibrosis in the prognostic evaluation of aortic valve stenosis.^212 , 251 , 874 , 892 , 901^	IIa	B
Evaluation of prosthetic heart valves complementary to echocardiography.^902 , 903^	IIb	C
Evaluation of suspected masses or vegetations in the mitral valve leaflets in the differential diagnosis of infective endocarditis.^904^	III	C

### 3.25. Cardio-oncology

Improved survival associated with effective cancer treatment has led to an increase in cardiac complications related to antineoplastic therapy. Early detection of cardiotoxicity can not only identify cardiovascular damage early but also allow for the implementation of targeted therapies.^905^

Despite several advances, effective detection of cardiotoxicity is still extremely challenging. Cardiac dysfunction often manifests long after the completion of cancer treatment, making it difficult to establish a direct correlation.^906^Additionally, this assessment typically relies solely on longitudinal monitoring of LV systolic function and LVEF by conventional echocardiography, which has several limitations, such as limited reproducibility and inability to provide tissue characterization.^907^Furthermore, reductions in LVEF often occur within normal reference ranges, indicating that myocardial injury may be present even before LV dysfunction is evident.^908 , 909^In this context, CMR is the gold standard for obtaining data on cardiac morphology and function with accuracy as well as reproducible results.^910^CMR also offers a wide range of imaging methods, such as cine sequences for morphology/function, T2 sequences for edema, perfusion for ischemia, LGE for scar detection, tagging for strain assessment, and T1 mapping for tissue characterization.

#### 3.25.1. Evaluation of Ventricular Morphology and Function

CMR is considered the method of choice for evaluating morphological and functional changes caused by cardiotoxicity,^911 – 913^providing accurate information on ventricular volumes and mass in a reproducible and independent manner.^914^Drafts et al.^915^demonstrated that cine CMR imaging can detect early cardiac function abnormalities, even in patients receiving low or intermediate doses of anthracyclines. Another study comparing different imaging techniques in adults who had childhood cancer and were treated with anthracyclines found that conventional echocardiography had limited accuracy in confirming LVEF < 50% as defined by CMR, with a sensitivity of 25% and high false positive rates (75%).^916^Although 3D echocardiography improved sensitivity to 53%, this modality was still inferior to CMR in detecting LVEF < 50%.^916^

The assessment of LV mass is also important, as chemotherapy can alter it.^915^Approximately 50% of childhood cancer survivors have LV mass < 2 standard deviations below normal values.^916^In a study involving patients with LV dysfunction treated with anthracyclines (median follow-up of 88 months), Neilan et al.^917^found that indexed LV mass assessed by CMR was an important prognostic factor. Patients with indexed LV mass < 57 g/m² had a significantly higher risk of cardiovascular events, including cardiovascular death, appropriate ICD therapy, and admission for HF.^917 , 918^Despite RV dysfunction being a recognized prognostic factor, few studies have specifically investigated the effects of cancer treatment on RV morphology and function. Recently, the ability of CMR to detect RV morphological and functional changes even at moderate doses of anthracyclines (240 mg/m²) has been documented.^919^

#### 3.25.2. Tissue Characterization

#### 3.25.2.1. Myocardial Fibrosis

Although LGE is an important tool in the investigation of myocardial scar and infarction, it only provides partial assessment of myocardial fibrosis.^418^As LGE relies on the differences in signal intensity between scarred and normal myocardium to generate contrast, it may fail to identify interstitial fibrosis.^907^In several studies, LGE was not uniformly detected in patients who developed cardiotoxicity after chemotherapy, particularly anthracycline-based regimens.^909 , 920 , 921^Thus, the absence of LGE in patients at risk of developing cardiotoxicity after cancer treatment should not be interpreted as the absence of myocardial injury.

#### 3.25.2.2. T1 Mapping

Several studies have examined the usefulness of CMR T1 mapping to investigate myocardial tissue remodeling after cancer therapy. T1 mapping before and after the administration of contrast agents, with correction of measurements to the patient's hematocrit, can be used to quantify ECV.^922^Tham et al. found a positive association between ECV and cumulative chemotherapy dose as well as exercise capacity assessed by cardiopulmonary exercise testing in a pediatric population.^923^In a relatively large cohort of cancer patients, Jordan et al. found that not only ECV but also native T1 were significantly higher in treated cancer patients compared with controls.^924^Some studies have demonstrated that CMR measurements of intracellular lifetime of water and ECV fraction may be used to assess myocardial remodeling at the cellular level, with histological validation.^907 , 918 , 925 , 926^

More recently, Thavendiranathan et al. demonstrated that T1 and T2 mapping are useful in the evaluation of cardiotoxicity caused by checkpoint inhibitors.^927^In 136 patients who developed myocarditis as a complication of checkpoint inhibitors, the authors showed that changes in T1 mapping not only have diagnostic utility but can also identify patients at high risk of subsequent cardiovascular events.^927^

Table 53 shows the recommendations for the use of CMR in the evaluation and follow-up of patients with suspected cardiotoxicity.

**Table 53:** Recommendations for cardiac magnetic resonance imaging in the assessment and follow-up of patients with suspected cardiotoxicity

Indication	Class of recommendation	Level of evidence
Evaluation of cardiac morphology and function (which may include myocardial strain) in patients with suspected cardiotoxicity.^928 – 930^	I	A
Investigation of myocardial fibrosis (which may include T1 mapping and extracellular volume) in the evaluation and monitoring of patients with cardiotoxicity.^930 – 932^	IIa	C
Evaluation of acute inflammatory activity (which may include T1 and T2 mapping) in the evaluation and monitoring of patients with cardiotoxicity.^931 , 932^	IIb	B
Evaluation of suspected immune checkpoint inhibitor myocarditis.^927 , 928 , 933 , 934^	IIa	B

### 3.26. Vascular Disease

Recent advances in imaging equipment and acquisition methods have benefited the use of MRI in the diagnosis of vascular diseases. Improved coil designs and new pulse sequences combined with imaging acceleration techniques have improved image resolution and signal, as well as reduced acquisition times.

Another advantage of CMR is that it does not expose patients to ionizing radiation. This can be beneficial in situations that require serial examinations to monitor certain vascular diseases, thereby reducing cumulative radiation exposure. The use of gadolinium-based paramagnetic contrast is extremely safe. Hypersensitivity reactions are very uncommon, and nephrogenic systemic fibrosis (primarily associated with GBCA use in patients with impaired renal function and on dialysis) has proven to be an increasingly rare phenomenon, related to specific gadolinium molecule profiles. As it does not cause direct nephrotoxicity, the use of GBCAs in MRA may be an alternative for patients with contraindications for iodine-based contrast agents due to low glomerular filtration rates.

The following section presents the different settings in which MRI may be used to evaluate vascular diseases. This Guideline does not address indications for MRA of the intracranial vessels.

#### 3.26.1. Aorta

Like CT, MRI plays a critical role in the evaluation of aortic diseases.^935^MRI provides multiplanar and volumetric imaging with excellent spatial resolution, allowing 3D assessment of the aortic lumen and walls. However, because it takes significantly longer than CT, it is less commonly used in patients with acute conditions.

MRI provides various sequences for aortic depiction and has high tissue characterization capability, improving diagnostic accuracy. In aortic evaluation, MRA, morphological sequences, and flow quantification may be performed. Some images are acquired during just one breath-hold, while others may take minutes, requiring the use of cardiac and respiratory synchronization techniques to obtain artifact-free images.

The most frequently used MRA techniques are contrast-enhanced, but modern sequences allow for noncontrast-enhanced techniques as well, with similar quality in some segments.^936^

As MRA is based on luminography, morphological sequences should also be performed to better assess the vascular wall and perivascular spaces. One of the most commonly used sequences is "black-blood," which nullifies the blood signal and highlights the vascular walls, and may be T1-weighted (allowing identification of hematoma and contrast enhancement) or T2-weighted (allowing edema assessment). Recently, MR vessel wall imaging using black-blood techniques has gained prominence, characterized by thin slices with high spatial resolution, allowing detailed analysis of the vascular wall. MRI also allows qualitative and quantitative flow analysis using specific techniques, with phase-contrast and 4D-flow being the most commonly used.

MRI is based on RF and thus does not use ionizing radiation, which is beneficial for patients requiring serial examination. The main aortic diseases were discussed in the chapter on aortic evaluation by CT.

Table 54 shows the main recommendations for the use of MRI in the diagnosis of aortic diseases.

**Table 54:** Recommendations for the use of magnetic resonance imaging in the diagnosis of aortic diseases

Indication	Class of recommendation	Level of evidence
Aortic aneurysm.^419 , 937 – 940^	I	B
Chronic aortic dissection.^419 , 937 , 938^	I	B
Aortic intramural hematoma.^419 , 937 – 941^	I	B
Aortic ulcers.^419 , 937 , 938^	I	C
Planning of aortic surgery approach.^419 , 937^	I	B
Planning of aortic endograft.^419 , 937 , 942^	I	B
Large- and medium-vessel arteritis.^943 , 944^	I	B
Acute aortic dissection.^419 , 937 , 941^	IIa	B
Postoperative evaluation of aortic endograft.^945 – 947^	IIb	B
Measurement of aortic diameters.^419 , 937^	I	C
Acute aortic syndrome in unstable patients.^419 , 937 , 938^	III	C
Acute aortic syndrome in stable patients.^419 , 937 , 938 , 941^	IIa	C
Traumatic aortic injury.^937 , 938 , 948^	IIb	C

#### 3.26.2. Extracranial Carotid Arteries

MRA of the carotid arteries allows for the examination of different aspects of carotid atherosclerosis, including blood flow patency, vessel diameter, the presence of stenoses, and vessel wall characteristics. It can also assess various vascular diseases, vascular anatomy, and the relationships between vessels and other anatomical structures and lesions in the cervicothoracic, neck, and cervicocranial regions.

The main indications for MRA of the carotid arteries are similar to those for CTA of the carotid arteries.^949^MRA and CTA of the carotid arteries are more accurate than ultrasound in the assessment of carotid stenosis,^408 , 950^and these modalities are recommended for cases requiring confirmation of the degree of stenosis to guide treatment approach.^951^As MRA and CTA offer similar accuracy in the evaluation of carotid stenosis, the choice between these methods should consider specific contraindications (eg, iodine contrast allergies, exposure to ionizing radiation), the urgency of the evaluation (favoring CT in emergencies due to faster acquisition), and the availability of the methods.

Table 55 shows the main recommendations for the use of MRI in the evaluation of extracranial carotid arteries.

**Table 55:** Recommendations for the use of magnetic resonance imaging in the assessment of extracranial carotid arteries

Indication	Class of recommendation	Level of evidence
Evaluation of carotid artery stenoses.^952 , 953^	I	B
Assessment of plaque composition in carotid arteries.^953 , 954^	IIb	C
Evaluation of carotid arteries after acute stroke when computed tomography is contraindicated or inconclusive.^953 , 955 , 956^	IIa	C

#### 3.26.3. Renal Arteries

Contrast-enhanced MRA of the kidneys has long been used for investigating the course, diameter, anatomical variations, and stenosis of renal arteries, as well as for surgical planning of nephrectomy.^419^Dynamic contrast-enhanced sequences have recently been suggested as capable of measuring renal perfusion.^957^The introduction of renal denervation as a potential treatment for resistant hypertension^958 – 960^has also increased interest in evaluation of renal artery anatomy and noninvasive quantification of renal and cardiorenal function by MRA.^419 , 961^New imaging sequences, including noncontrast-enhanced ones, have shown promise in the detection of renal artery stenosis.^962 – 964^However, contrast-enhanced sequences are already well-established for the diagnosis of renal artery stenosis, with a sensitivity of 97% and specificity of 93% compared with arteriography.^965^

Table 56 shows the recommendations for the use of MRI in the evaluation of renal arteries.

**Table 56:** Recommendations for the use of magnetic resonance imaging in the assessment of renal arteries

Indication	Class of recommendation	Level of evidence
Evaluation of renal artery stenosis.^965 – 967^	I	B

#### 3.26.4. Pulmonary Arteries

MRA is useful in the assessment of pulmonary artery anatomy, diameter, and patency. It also allows for qualitative and quantitative flow analysis with the use of specific techniques.

However, it is not ideal for assessing pulmonary thromboembolism (PTE)^968^due to its lower sensitivity, higher rate of inconclusive results, limited availability in most emergency settings, and longer acquisition times compared with CT. If conventional methods for PTE evaluation, such as CTA and scintigraphy, are not feasible, MRA becomes a plausible alternative.^969^For PTE assessment, gadolinium-enhanced MRA is recommended for higher accuracy in intravascular analysis.

The main anomalies of the pulmonary arteries were addressed in the chapter of this Guideline on evaluation of pulmonary arteries by CT.

Table 57 presents the main recommendations for the use of MRI in the evaluation of pulmonary arteries.

**Table 57:** Recommendations for the use of magnetic resonance imaging in the evaluation of pulmonary arteries

Indication	Class of recommendation	Level of evidence
Evaluation of pulmonary thromboembolism (as an alternative to computed tomography).^419 , 969 , 970^	IIa	B
Measurement of the pulmonary trunk and main pulmonary arteries.^419 , 971^	I	B

#### 3.26.5. Visceral Arteries

Stenosis of the celiac trunk and mesenteric arteries can also be diagnosed using contrast-enhanced MRA. Although CTA is generally preferred, as discussed in the CT section of this Guideline, MRA can provide valuable information about stenosis and collateral circulation, as well as nonatherosclerotic diseases such as fibromuscular dysplasia and median arcuate ligament compression.^972^However, MRA is less effective than CTA, especially in the distal portions of the mesenteric vessels.^419^

MRA is particularly useful when there are concerns about the use of ionizing radiation or the nephrotoxicity associated with iodine contrast. Table 58 shows the recommendations for the use of MRI in the evaluation of visceral arteries.

**Table 58:** Recommendations for the use of magnetic resonance imaging in the assessment of visceral arteries

Indication	Class of recommendation	Level of evidence
Evaluation of the mesenteric arteries and celiac artery.^419 , 953 , 973^	IIa	C

**Table 59:** Recommendations for the use of computed tomography in the assessment of atrial septal defects (ASDs)

Indication	Class of recommendation	Level of evidence
Evaluation prior to ASD repair to assess septal anatomy and investigate atrial septal aneurysms.^974 , 979^	IIa	C
Evaluation prior to repair of sinus venosus ASD and/or partial APVC and coronary sinus.^974 , 975 , 980^	I	B
Evaluation following percutaneous closure of ASD in patients with significant residual shunt, valvular or ventricular dysfunction, arrhythmias, and/or pulmonary hypertension.^981^	IIb	C
Evaluation following percutaneous closure of ASD of PFO to assess for complications, embolization, or persistent residual shunt.^978^	IIb	C
Evaluation following percutaneous closure of ASD to assess for device malposition.^978^	IIa	C

## 4. Congenital Heart Disease

### 4.1. CT in the Evaluation of Congenital Heart Diseases

MDCT offers significant contributions to the evaluation of patients with congenital heart disease. This imaging modality is fast, often obviating the need for sedation, which adds practicality and makes it feasible for virtually all patients, including those in unstable conditions. However, the use of ionizing radiation requires careful consideration, particularly for repeated examination, of long-term cumulative effects. Additionally, the need for iodinated contrast media should be evaluated in patients with renal dysfunction.

MDCT is primarily used for the anatomical characterization of cardiac malformations and the postoperative evaluation of these conditions. It plays a crucial role in the determination of stent, tube, and conduit patency, as well as associated complications. MDCT is also valuable for the assessment of extracardiac vascular anomalies often associated with an underlying disease, as well as the outcomes of surgical anastomosis, such as partial or total cavopulmonary shunt (Glenn-Fontan procedure) and pulmonary-to-systemic arterial shunt (Blalock-Taussig procedure).

Although not primarily intended for this purpose, ECG-synchronized MDCT may also be used to assess ventricular function and volumes. In cases where echocardiography or CMR is inconclusive or not feasible, these parameters can be evaluated using CT. Similarly, morphological and functional assessment of cardiac valves (leaflet mobility/calcifications/regurgitant orifices) may also be performed in selected cases.

Given the specificity of each congenital heart disease, the application of MDCT in different clinical settings will be discussed individually in sections dedicated to each congenital heart disease described in this Guideline.

#### 4.1.1. Evaluation of Intracardiac and Extracardiac Shunts

#### 4.1.1.1. Atrial and Ventricular Septal Defects

The assessment of atrial (ASDs) and ventricular (VSDs) septal defects is typically performed adequately and conclusively via echocardiography. CT and CMR are primarily reserved for cases where echocardiography is inconclusive, especially in preoperative evaluations, such as in cases of sinus venosus ASDs often associated with anomalous pulmonary venous drainage.^974 – 976^

Compared with CMR, CT has the disadvantage of offering a more limited assessment of the impact of septal defects. It only detects dilation of mediastinal vessels and cardiac chambers, thereby playing a smaller role in the follow-up of these patients.^974^Functional contractile analysis is possible with CT but at the cost of higher radiation doses. Conversely, CT is superior in the assessment of intracardiac shunts^977^with complex septal geometry, such as multiple muscular VSDs.

After percutaneous shunt closure, CMR or CT may be indicated if complications such as infection, malposition, embolization, or persistent shunt are suspected.^978^

The following tables describe the main recommendations for the use of CT in the evaluation of intracardiac shunts — ASDs, VSDs, and atrioventricular septal defects (AVSDs) ( Tables 59 , 60 , and 61 , respectively) — and extracardiac shunts — PDA ( Table 62 ).

**Table 60:** Recommendations for the use of computed tomography in the evaluation of ventricular septal defects (VSDs)

Indication	Class of recommendation	Level of evidence
Evaluation prior to VSD repair in patients with complex anatomy.^982 , 983^	IIa	C
Evaluation following surgical or percutaneous closure of VSD in patients with significant residual shunt, valvular or ventricular dysfunction, arrhythmias, and/or pulmonary hypertension.^984^	IIb	C

**Table 61:** Recommendations for the use of computed tomography in the evaluation of atrioventricular septal defects (AVSDs)

Indication	Class of recommendation	Level of evidence
Evaluation prior to AVSD repair.^975 , 976^	IIa	C
Evaluation following surgical closure of AVSD in patients with significant residual shunt, valvular or ventricular dysfunction, arrhythmias, and/or pulmonary hypertension.^974^	IIa	C
Evaluation following surgical closure of AVSD in patients with signs of heart failure.^974^	IIb	C

**Table 62:** Recommendations for the use of computed tomography in the evaluation of patent ductus arteriosus (PDA)

Indication	Class of recommendation	Level of evidence
Evaluation prior to correction of PDA.^983^	IIb	C
Post-treatment evaluation of suspected aortic or pulmonary artery complications.	I	C

#### 4.1.1.2. Partial and Total Anomalous Pulmonary Venous Connection

The anatomical evaluation of anomalous pulmonary venous connection (APVC) using CT is comparable to that of CMR,^985^allowing for an accurate and comprehensive preoperative assessment.^974 , 976^

As with septal defects, a disadvantage of CT is its limited functional assessment of the cardiac repercussions of APVC, making it less suitable for the follow-up of these patients.^974^Conversely, CT is effective in the visualization of pulmonary repercussions, including signs of pulmonary congestion, such as interstitial or alveolar edema, and pleural effusions.

In patients with congenital pulmonary venolobar syndrome (scimitar syndrome), the evaluation of associated bronchopulmonary malformations is crucial, as is the identification of areas of systemic pulmonary blood supply, both of which can be accurately assessed by CT. Table 63 shows the main recommendations for the use of CT in the evaluation of APVC.

**Table 63:** Recommendations for the use of computed tomography in the assessment of anomalous pulmonary venous connection (APVC)

Indication	Class of recommendation	Level of evidence
Evaluation prior to correction of APVC.^980 , 983^	I	B
Post-treatment evaluation of APVC-related complications.^977 , 985^	I	C
Evaluation following correction of total APVC in asymptomatic patients with no or mild sequelae.	IIa	C

#### 4.1.2. Congenital Valvular Heart Disease

Many patients with congenital heart disease require reoperation, often involving multiple valves. In young patients undergoing mitral valve replacement, 50% will require reoperation within 10 years and 15% may require a pacemaker within 1 month. Several studies have shown the utility of cardiac CT in the evaluation of native and mechanical valve stenosis and insufficiency, paravalvular leak, thrombosis, abscesses, and endocarditis. Differences in stroke volume between ventricles, calculated from functional data sets, can be used to quantify the severity of valve regurgitation and are closely correlated with echocardiographic findings.^986 , 987^

#### 4.1.2.1. Tricuspid Valve/Ebstein's Anomaly

Ventricular volumes and EFs can be obtained by retrospective ECG-gated CCTA in a manner similar to CMR in patients who cannot undergo CMR. The morphology and mobility of the leaflets can be assessed on cine reconstructions. CCTA is also an alternative to CMR when coronary artery assessment is required. However, CCTA does not allow quantification of flow or assessment of the severity of tricuspid regurgitation.^988^

#### 4.1.2.2. Pulmonary Valve

CTA has the advantage of high-resolution whole-chest coverage and is able to depict anatomic detail of the pulmonary valve, perivalvular structures, and pulmonary artery branches. CTA is the modality of choice for assessing the proximal and distal pulmonary arteries, as well as the proximity of the origin and course of the coronary artery to the implantation site. However, because the temporal resolution of CTA is lower than that of MRI and echocardiography, its application for functional assessment of the pulmonary valve is limited. CTA is an alternative for assessing the pulmonary valve when CMR is not feasible, especially when depiction of anatomy, complications, and ventricular size and function is desired. Dual-source scanners with faster temporal resolution improve image quality by reducing motion artifacts. CTA also provides valuable information on morphological features, post-stenotic dilation, and the location of supravalvular or subvalvular stenosis.^989 , 990^Vegetations that affect the pulmonary valve are seen at echocardiography in 50% of patients with endocarditis. These vegetations may be accompanied by concomitant thickening, shortening, perforations, or complete destruction of the cusps, causing pulmonary regurgitation. Pseudoaneurysms may form at the site where a paravalvular abscess empties into the heart or vessel lumen. Fistulas may cause shunting and paravalvular leakage. ECG-gated CCTA is excellent for evaluation of structural abnormalities associated with pulmonary valve endocarditis. However, its use may be limited for the assessment of small vegetations (< 4 mm) and small valve perforations.^991^

As with the aortic valve, CT provides valuable information for percutaneous pulmonary valve implantation. Indications for a transcatheter approach are similar to those for surgery. Furthermore, the morphological characteristics of the RVOT are a major criterion for transcatheter valve replacement eligibility, a determination that may easily be made at CT or MRI. Patients with an aneurysmal appearance are not eligible for transcatheter pulmonary valve implantation. Complications of valve implantation include stent migration, stent fracture, conduit rupture, coronary artery compression, and pulmonary hemorrhage, which can be well evaluated by CTA.^990^

#### 4.1.2.3. Mitral Valve

Congenital mitral valve abnormalities include those associated with AV septal defects, parachute mitral valve, and double-orifice mitral valve. The spectrum of total AVSDs encompasses complete defects, which are composed of a common AV valve, ostium primum ASD, and membranous ventricular septal defect; partial defects, which are constituted by separate tricuspid and mitral valves, a cleft anterior mitral leaflet, and an ostium primum ASD; and various transitional or intermediate categories that fall between these two ends of the spectrum. A parachute mitral valve occurs when all the chordae arise from a single, fused papillary muscle. This abnormality is associated with mitral stenosis of various degrees and with Shone syndrome.

Shone syndrome is characterized by a parachute mitral valve, supravalvular mitral ring, subvalvular aortic stenosis, and coarctation of the aorta (CoA). A double-orifice mitral valve is characterized by a bridge of leaflet tissue that divides the annulus into 2 orifices, both of which open into the LV. This condition is associated with varying degrees of mitral stenosis or regurgitation and has strong associations with additional valvular anomalies and other congenital cardiac defects.

Echocardiography is typically sufficient for the assessment of mitral valve defects, without the need for additional investigation to support therapeutic decision-making and/or follow-up of this patient group. CTA is extremely useful in patients with Shone's syndrome with a broad spectrum of symptoms because it can assess multiple parameters simultaneously. When valve replacement is required, CT can be used for postoperative evaluations because it has higher spatial resolution and is less susceptible to artifacts. Cine CT has demonstrated excellent utility for identifying paravalvular abscesses and valvular vegetations.^992^

#### 4.1.2.4. Aortic Valve

For the evaluation of bicuspid aortic valve and isolated aortic valve stenosis, echocardiography generally does not require supplementary diagnostic imaging. However, in patients with sequential subvalvular, valvular, and supravalvular stenoses, CTA can provide additional information, particularly regarding leaflet elongation, tunnel-like LVOT obstruction, or the presence of discrete membranes that may not be visualized on CMR due to lower spatial resolution. This additional information can contribute to surgical planning.^993^ Table 64 presents the main recommendations for the use of CT in the evaluation of congenital mitral valve abnormalities.

**Table 64:** Recommendations for the use of computed tomography in the assessment of congenital valve abnormalities

Indication	Class of recommendation	Level of evidence
Complementary evaluation of regurgitant valvular lesions.^987 , 994^	IIb	B
Complementary evaluation of stenotic valvular lesions.^989 , 995 , 996^	IIa	B
Evaluation prior to correction of Ebstein's anomaly (tricuspid valve, right ventricle, and pulmonary tree).^988^	IIa	C
Evaluation of valvular or ventricular dysfunction following correction of Ebstein's anomaly.	IIb	C

#### 4.1.3. Conotruncal Anomalies

#### 4.1.3.1. Tetralogy of Fallot

In preoperative evaluation, most diagnostic information is provided by echocardiography. CT can provide complementary data when echocardiography is inconclusive, such as in cases involving coronary anomalies or more complex settings such as pulmonary atresia, in which the evaluation of collateral vessels and pulmonary vasculature segmentation is necessary.^997 – 999^

In patients with tetralogy of Fallot with pulmonary valve agenesis, CT is useful to assess central airway compression due to aneurysmal dilations of the pulmonary arteries.

Postoperatively, CT can identify surgical complications of reconstructions of the RVOT, pulmonary trunk, and proximal vascular tree, as well as peripheral vascular beds. It is also a superior method for evaluating in-stent restenosis.^1000 , 1001^

CT may be an alternative method for quantifying ventricular volumes and function in cases where MRI is contraindicated or limited by implanted devices, allowing for the monitoring of residual lesions. Table 65 presents the recommendations for the use of CT in the evaluation of patients with Tetralogy of Fallot.

**Table 65:** Recommendations for the use of computed tomography in the assessment of Tetralogy of Fallot

Indication	Class of recommendation	Level of evidence
Evaluation prior to repair in patients with unfavorable anatomy or when echocardiography is limited.^999 , 1001^	I	C
Postoperative evaluation prior to planned pulmonary valve replacement (percutaneous [Melody→] or surgical).^262 , 1002 , 1003^	IIa	C
Evaluation in patients with right ventricular outflow tract obstruction, pulmonary stenosis, arrhythmias, or the presence of a right ventricle-to-pulmonary artery conduit.^1004^	I	C

#### 4.1.3.2. Double-outlet Right Ventricle

Double-outlet RV (DORV) is not a single cardiac anomaly but a heterogeneous group with variable morphological features. The abnormal position of the great vessels in association with various structural anomalies can lead to different physiological phenotypes.^1005 , 1006^DORV represents a complex spectrum consisting of varied position of the VSD in relation to the pulmonary and aortic valves, as well as varying degrees of malposition of the great arteries. A 3D printed model based on CT datasets can help accurately understand VSD location to guide surgical approach.^1007^In patients with DORV, the size and position of the VSD relative to the LV and the great arteries is important for understanding the physiology and for determining surgical repair.^1008 , 1009^

An accurate understanding of the intracardiac anatomy is crucial to estimate difficulties, predict VSD enlargement, and detect interposition of the atrioventricular valve apparatus or reduced ventricular cavity. 3D imaging of the intracardiac anatomy in this setting helps determine whether an intraventricular tunnel is viable and whether sufficient exposure can be obtained through the RA and tricuspid valve so that a right ventriculotomy can be avoided.^1010^

Because modern CT scanners offer the benefit of improved temporal and spatial resolution, algorithms for lower radiation doses, and advanced 3D postprocessing tools, they are a low-risk and high-quality alterative to diagnostic cardiac catheterization. They have been increasingly used in the evaluation of more complex anatomical arrangements. Table 66 presents the main recommendations for the use of CT in the evaluation of DORV.

**Table 66:** Recommendations for the use of computed tomography in the evaluation of double-outlet right ventricle

Indication	Class of recommendation	Level of evidence
Evaluation of ventricular septal defect relationship and vascular anatomy prior to repair.^1007 – 1009^	I	C
Evaluation of ventricular dysfunction, right or left ventricular outflow tract obstruction, pulmonary stenosis, arrhythmias, or the presence of a right ventricle-to-pulmonary artery conduit.^1010^	IIa	C

#### 4.1.3.3. Common Arterial Trunk

Echocardiography is the primary modality for evaluation of common arterial trunk. However, when visualization of extracardiac structures is limited, CT may be useful for delineating the extracardiac anatomy of common arterial trunk.^1011^Preoperative evaluation should include delineation of vascular anatomy, particularly the pulmonary component and the distance between the pulmonary arteries. Whether the pulmonary arteries originate from the sinotubular junction, a sinus of the truncal root, or if there is crossing of their origins should also be determined.^1012^The morphological features dictate the surgical approach, and the presence of associated anomalies influences the surgical outcome and patient mortality.

These anomalies may range from varying degrees of coarctation to interruption of the aortic arch. CT contributes to vascular assessment by accurately identifying the origin and course of the pulmonary arteries and characterizing the degree of aortic arch malformation.^1013^Additionally, the spatial resolution of CT provides diagnostic information regarding coronary circulation by allowing a clear delineation of the coronary ostia and epicardial course. CAAs may occur, such as a single coronary artery, abnormal proximal epicardial course, intramural course, and slit-like or pinpoint ostia. The presence of a coronary anomaly is a risk factor for late mortality after surgical repair.^1014 , 1015^

Postoperative evaluation should assess if continuity between the RV and pulmonary artery was established via a conduit, excluding areas of significant luminal narrowing or local vascular distortions. Determining the proximity of the surgical repair to other structures, such as coronary circulation and the retrosternal space, is essential when reoperation is required.

For surgical repairs in the aortic region, CT is useful for characterization of the anastomotic site, excluding anastomotic narrowing, aneurysmal dilations, or other local complications.^1010 , 1016^ Table 67 presents the recommendations for the use of CT in the evaluation of common arterial trunk.

**Table 67:** Recommendations for the use of computed tomography in the evaluation of common arterial trunk

Indication	Class of recommendation	Level of evidence
Evaluation of anatomy prior to repair.^1011 , 1012^	I	C
Postoperative evaluation of known residual vascular septal defect, right ventricle-to-pulmonary artery conduit stenosis, or pulmonary stenosis.^1014 – 1016^	I	C
Postoperative evaluation of truncal valve stenosis or regurgitation.^1017^	IIa	C

#### 4.1.3.4. Transposition of the Great Arteries

Preoperatively, CT can be a useful complementary method in the diagnosis of coronary anomalies and associated lesions, such as aortic arch obstruction.^1018^

After a Jatene procedure, careful evaluation of the ostia and proximal part of the coronary arteries is mandatory, as they are transferred from the native aorta to the neoaorta (native pulmonary valve) during arterial switch. CT is the noninvasive modality of choice to evaluate the coronary region, including the ostia and the presence of an epicardial course after coronary reimplantation. Angulation and traction may occur and lead to ischemia and myocardial damage. In these cases, CT is indicated for the evaluation of coronary distortions and luminal reduction.^1019 – 1021^

The pulmonary and systemic venous baffles are also assessed for stenosis at suture sites. Pulmonary stenosis may occur at the supravalvular level or involve the pulmonary arteries as a consequence of their repositioning following the LeCompte maneuver or local distortions due to dilation of the neoaortic root.^1022^

In atrial-level repair, it is possible to clearly delineate both the pulmonary venous baffle and the superior and inferior vena cava baffles. When MRI evaluation of the systemic ventricle is not feasible, CT is an alternative for assessing RV volume and function.^1023^ Table 68 presents recommendations for the use of CT in the evaluation of transposition of the great arteries.

**Table 68:** Recommendations for the use of computed tomography in the assessment of transposition of the great arteries

Indication	Class of recommendation	Level of evidence
Evaluation of anatomy prior to repair.^1018^	IIa	C
Coronary evaluation following a Jatene procedure.^1019 – 1021 , 1024^	I	C
Evaluation of moderate valvular or ventricular dysfunction, right or left ventricular outflow tract obstruction, pulmonary stenosis, or arrhythmias following a Jatene procedure.	IIa	C
Evaluation of dilated neoaortic root or neoaortic regurgitation following a Jatene procedure.^1022^	IIa	C
Evaluation of systemic AV regurgitation, systemic RV dysfunction, LVOT obstruction, or arrhythmias following atrial repair (eg, Senning procedure).	I	C
Evaluation following atrial repair (eg, Senning procedure): evaluation of venous baffles.^1023^	I	C

AV: atrioventricular; LVOT: left ventricular outflow tract; RV: right ventricular.

#### 4.1.3.5. Congenitally Corrected Transposition of the Great Arteries

CT allows for the preoperative characterization of atrioventricular and ventriculoarterial discordance, as well as other associated defects. It is as an alternative for patients in whom MRI is contraindicated and is particularly useful for accurate delineation of coronary anatomy. CT accurately demonstrates inversion of the ventricles and coronary arteries, as well as the spatial relationship between the aorta and pulmonary trunk. Among associated defects, CT can locate VSDs and evaluate hemodynamic effects. Varying degrees of LVOT may be present and may include alterations in either the subpulmonary or pulmonary valve.^1010 , 1025^

CT can provide a comprehensive postoperative evaluation at both the arterial and atrial levels (double switch), including assessment of coronary reimplantation, arterial anastomosis, and venous redirections at the atrial level.^1026^When only physiological repair is performed, CT can assist in assessing corrections of pulmonary venous baffle obstructions or residual shunting after VSD closure. Table 69 presents the recommendations for the use of CT in the evaluation of congenitally corrected transposition of the great arteries

**Table 69:** Recommendations for the use of computed tomography in the assessment of congenitally corrected transposition of the great arteries

Indication	Class of recommendation	Level of evidence
Evaluation of anatomy prior to repair.^1010 , 1025^	I	C
Postoperative evaluation in patients with systemic atrioventricular valve regurgitation or systemic right ventricular dysfunction.	IIa	C
Postoperative evaluation in patients with left ventricle-to-pulmonary artery conduit dysfunction.^1026 , 1027^	I	C

#### 4.1.4. Thoracic Aorta Anomalies

#### 4.1.4.1. Coarctation and Other Aortic Abnormalities

Like CMR, CT is a complementary noninvasive method that provides visualization of the aorta in the investigation of aortic anomalies.^975 , 977 , 993 , 1028^These include coarctation, hypoplasia, aortic arch interruption, among others, as well as associated lesions.^977 , 1029^The accuracy of CT is slightly superior to CMR due to its high spatial resolution.

Some conditions are associated with the formation of vascular rings. CT is crucial for visualization of the airways and often provides information that are not attainable with other diagnostic methods, simultaneously demonstrating the vascular anomaly and its impact on the airways.^977^Vascular compression of the central airways may result from several conditions, such as aortic arch anomalies, pulmonary sling, tetralogy of Fallot with absent pulmonary valve, or pulmonary artery dilation.^977 , 1029 – 1032^It should be noted that CT evaluation of esophageal compression is limited and should often be complemented by other methods, such as esophagography.^1029^

Follow-up of correction of aortic arch coarctation or interruption is recommended to assess aortic arch geometry and to rule out complications such as restenosis, local aneurysms, or dissections. This follow-up should preferably be conducted using CMR, as it does not require the use of ionizing radiation.^975^However, in patients with a ferromagnetic implant, CT is preferred because it causes fewer local artifacts that may limit luminal assessment.^984 , 1030 , 1033 , 1034^Device-related complications after endograft implantation include residual stenosis, fracture, dissection, endoleak, or local aneurysm, without the occurrence of artifacts as in CMR. Tables 70 and 71 present recommendations for the use of CT in the evaluation of CoA and aortic arch anomalies, respectively.

**Table 70:** Recommendations for the use of computed tomography in the assessment of coarctation of the aorta (CoA)

Indication	Class of recommendation	Level of evidence
Evaluation prior to CoA repair.^975 , 984^	I	B
Evaluation following CoA repair due to changes in clinical status.^984 , 1033^	I	C
Evaluation (6-12 months) within a year following percutaneous repair in asymptomatic patients with no or mild sequelae.^1035 , 1036^	IIa	B
Evaluation (1-2 years) after the first year following CoA repair in asymptomatic patients with no or mild sequelae.^1037^	IIa	B
Post-treatment evaluation in asymptomatic patient to assess for aortic arch aneurysm, in-stent stenosis, stent fracture, or endoleak.^1030 , 1034^	I	B
Post-treatment follow-up of patients with heart failure symptoms.^975 , 977 , 984 , 993 , 1028 , 1034^	IIa	B

**Table 71:** Recommendations for the use of computed tomography in the assessment of aortic arch anomalies

Indication	Class of recommendation	Level of evidence
Evaluation of vascular rings, airways, and lung parenchyma.^1029 , 1031^	I	B
Evaluation of aortic arch interruption.^1038^	I	C
Evaluation of aortopulmonary window.	I	C

#### 4.1.5. Univentricular Heart

In recent years, the use of CTA in pediatric patients has rapidly increased due to its excellent spatial and temporal resolution. Fast examinations using the latest generation CT scanners have been applied in univentricular heart conditions, in which only 1 ventricle is fully developed.^1039^The high spatial resolution of CTA allows excellent evaluation of small structures, as well as a comprehensive assessment of extracardiac vasculature and its relationships with other thoracic structures.

Correction of univentricular heart involves 3 stages of univentricular palliative surgery. The first stage, if necessary and dependent on the physiology, is performed in neonates and usually involves a Norwood procedure, systemic-to-pulmonary arterial shunt, or pulmonary artery banding. Some centers advocate a "hybrid" approach consisting of catheter-placed ductal stent and pulmonary artery bands.^986 , 1040^

#### 4.1.5.1. Prior to Stage 1 Surgery

While many patients with single-ventricle anatomy can be adequately assessed using echocardiography prior to stage 1 surgery, CTA is occasionally needed to define complex anatomy, particularly when echocardiography fails to delineate extracardiac vascular anatomy.^986^In some cases, a detailed description of the arterial duct anatomy is required for planning stent implantation in more complex and challenging anatomies.^1041^

#### 4.1.5.2. After Stage 1 Surgery (Norwood, Systemic-to-pulmonary Arterial Shunt, Hybrid)

Between stage 1 and 2 surgery, systemic and pulmonary artery stenoses are relatively common, and are often insufficiently visualized with echocardiography. Distal and segmental pulmonary branch stenosis secondary to retraction and stenosis at anastomotic or selective banding sites are well visualized by CTA.

Patients with systemic-to-pulmonary artery shunts occasionally experience shunt thrombosis, resulting in acute, profound cyanosis. Shunt thrombosis can be challenging to identify with echocardiography, but CTA, given its wide accessibility and fast imaging acquisition times, is an excellent alternative and may also help determine the need for intervention.^986 , 1042 , 1043^

In most centers, cardiac catheterization is performed in preparation for the Glenn procedure. A comparison of CTA and cardiac catheterization prior to stage 2 palliation revealed excellent correlation with surgical findings for both modalities and no difference in surgical outcomes to hospital discharge. The catheterization group had increased radiation and contrast exposure, required central vascular access and general anesthesia in all cases, and had a relatively high rate of adverse events.^1044^A previous study randomly assigned pre-stage 2 patients to either CMR or catheterization and found no differences in surgical and medium-term outcomes after a mean follow-up period of 8 years.^1045^Some centers now propose that noninvasive diagnostic evaluation be performed before palliation and that catheterization be reserved for therapeutic procedures.^1046^

#### 4.1.5.3. After Stage 2 and 3 Surgery (Glenn and Fontan Procedures)

Cardiovascular CT has been shown to adequately visualize all aspects of the Glenn or Fontan circuit after single-ventricle palliation. Changes in hemodynamics and anatomy after single-ventricle palliation cause unique challenges in optimal opacification of the pulmonary arteries and cavopulmonary circuit that can result in nondiagnostic CTA studies or erroneous image interpretation. It is important to use techniques to optimize enhancement of the pulmonary arteries and recognize common pitfalls in performing and interpreting pulmonary CTA studies at each stage.^1047 , 1048^

Thrombus formation is relatively common, and thrombi can be visualized by CTA in the Fontan conduit, residual ventricle or in the residual pulmonary artery stump after ligation of the pulmonary artery. PE may also be identified by CTA. However, care should be taken to optimize the contrast injection technique both to optimally opacify the Fontan circuit and to avoid a false positive diagnosis of PE.^986 , 1048^

Another common complication in this group of patients is pulmonary artery stenosis, which may occur due to scarring from previous manipulations, shunt anastomosis, diffuse branch hypoplasia after Norwood surgery, or after removal of pulmonary bands. CTA is excellent for the evaluation of pulmonary arteries, as well as previous pulmonary stents that may be poor visualized on CMR due to imaging artifacts.^1033 , 1049^

CTA can also visualize systemic-to-pulmonary and/or venovenous collateral vessels but, contrary to CMR, cannot quantify their flows or hemodynamic effects. However, CTA remains an alternative for cases where CMR is contraindicated.^1033 , 1050 – 1053^Quantification of ventricular function by CTA may be warranted in patients with metallic implants and contraindications to CMR.^986 , 1048^

CTA also provides diagnostic information in more unstable and complex clinical situations such as patients on extracorporeal membrane oxygenation or ventricular assist device support, a population in which conventional imaging may be challenging. In addition to evaluating residual lesions, it is possible to evaluate cannula positioning, presence of thrombi, and possible sites of infection in the mediastinum.^986 , 1054^ Table 72 presents the recommendations for the use of CT in the evaluation of univentricular heart.

**Table 72:** Recommendations for the use of computed tomography in the assessment of univentricular heart

Indication	Class of recommendation	Level of evidence
Evaluation of anatomy prior to repair.^986^	IIa	C
Evaluation of systemic-to-pulmonary artery shunt or pulmonary banding following stage 1 surgery.^1042 – 1044^	I	C
Evaluation of valvular or ventricular dysfunction, superior vena cava to pulmonary artery anastomosis, or collateral circulation following stage 2 surgery (Glenn procedure).^1045^	IIa	C
Evaluation of valvular or ventricular dysfunction, thrombosis after total cavopulmonary connection, or collateral circulation following stage 3 surgery (Fontan procedure).^1046 , 1047^	IIa	C

#### 4.1.6. Other

Cardiac malformations can be associated with abnormal arrangement of the thoracic and abdominal organs. Heterotaxy and situs abnormalities consist of an abnormal arrangement of visceral organs in the thoracoabdominal cavity across the normal left-right axis of the body. They are associated with a high occurrence of congenital heart and abdominal defects, including APVCs, systemic venous abnormalities, asplenia, and other visceral abnormalities. They often involve more complex heart defects that require comprehensive vascular assessment across various regions.^1055^

CT provides extensive and valuable data on cardiovascular and extracardiac structures in heterotaxy. It accurately assesses thoracic anatomy, including lung parenchyma and airways, and clarifies the arrangement of abdominal viscera.^1056 , 1057^

Due to its high spatial resolution and multiplanar image reconstruction capabilities, CT allows for a comprehensive evaluation of other anomalies, such as crisscross heart, in which abnormal rotation of the long axis of the ventricles results in crossing of the ventricular inflows. It also allows evaluation of twisted atrioventricular connections and associated intra and extracardiac anomalies to help choose the best surgical approach.^1058^ Table 73 presents the recommendations for the use of CT in the evaluation of heterotaxy and situs abnormalities.

**Table 73:** Recommendations for the use of computed tomography in the assessment of heterotaxy and situs abnormalities

Indication	Class of recommendation	Level of evidence
Evaluation of thoracic organ arrangement and long axis rotation.^1058^	I	C
Evaluation of situs anomalies and heterotaxy.^1055 – 1057^	I	C

### 4.2. CMR in Congenital Heart Disease

The use of CMR has become an important strategy in the diagnosis of congenital heart diseases. Because of its high spatial and temporal resolutions, CMR allows detailed anatomical assessment of congenital heart diseases and accurate evaluation of surgical outcomes, including potential complications from different surgical approaches. CMR is particularly suitable for pediatric populations as it does not require the use of ionizing radiation.

CMR provides several imaging modalities in a single examination, allowing for the assessment of different cardiovascular parameters. Cine CMR sequences are considered the gold standard for estimating ventricular function and volumes, which is essential for the pre and postoperative management of several congenital heart diseases. Flow assessments using phase-contrast techniques can accurately define valvular lesions and intra or extracardiac shunts. Angiographic studies (arterial and venous) allow for the adequate evaluation of systemic and pulmonary circulations in both pre and postoperative settings. Finally, tissue characterization helps to differentiate structures based on their histological composition (eg, tumors and thrombi).

While CMR offers several benefits in the diagnosis of congenital heart diseases, its use should be limited to specific settings due to its complexity and limited availability. CMR is particularly useful when echocardiography is limited (due to suboptimal images) or insufficient. In addition, breath-holding is usually required during image acquisition with CMR; in patients who are unable to cooperate and breath-hold (typically patients aged < 7 years), anesthesia and endotracheal intubation are required, with controlled apnea using a ventilator.

#### 4.2.1. Evaluation of intracardiac and Extracardiac Shunts

#### 4.2.1.1. ASDs

In general, ASDs are conclusively evaluated by echocardiography to guide clinical decisions in pediatric patients.^975 , 1059^CMR is a complementary test, particularly in adolescents and adults, when echocardiography is inconclusive.^974 , 978^

Sinus venosus ASDs and associated defects such as anomalous pulmonary venous return, which is often associated with superior sinus venosus ASD, may be difficult to visualize on echocardiography.^975 , 976 , 1060 – 1062^In these cases, CMR is a valuable tool for preoperative evaluation.^974^

CMR also allows the evaluation of hemodynamic effects by calculating the Qp/Qs ratio and the degree of ventricular overload and performance.^975 , 1063^In patients scheduled for surgical or percutaneous intervention, prior evaluation with CMR is less accurate in the delineation of ASD and its borders compared with echocardiography and CT.^979 , 981^However, it may be used to plan the most appropriate treatment strategy for secundum ASDs.^974^

#### 4.2.1.2. Patent Foramen Ovale

Like ASD, the evaluation of patent foramen ovale (PFO) by CMR is rarely necessary, as echocardiography and other modalities such as transcranial Doppler are usually conclusive.^978 , 1059^CMR or CT may be used in rare cases where echocardiography is limited.^981^The assessment involves checking for intravenous contrast medium extravasation or signs of transseptal flow during Valsalva maneuvers.^1064 , 1065^

After percutaneous closure of PFO, CMR and CT are only indicated if complications such as infection, malposition, embolization, or persistent shunt are suspected.^978^ Table 74 presents the main recommendations for the use of CMR in the evaluation of ASDs.

**Table 74:** Recommendations for the use of cardiac magnetic resonance imaging in the assessment of atrial septal defects (ASDs)

Indication	Class of recommendation	Level of evidence
Evaluation prior to ASD repair to assess septal anatomy, investigate atrial septal aneurysm, and guide therapeutic decision-making.^1062^	IIa	C
Diagnosis and preoperative assessment of sinus venosus ASD and/or anomalous pulmonary venous return.^976 , 1066^	I	B
Evaluation following surgical or percutaneous closure of ASD in patients with significant residual shunt, valvular or ventricular dysfunction, arrhythmias, and/or pulmonary hypertension.^976 , 978^	IIa	C
Evaluation following percutaneous closure of ASD in patients with suspected complications, embolization, or persistent residual shunt.^978^	IIb	C
Initial evaluation of patent foramen ovale.^1059 , 1064 , 1065^	III	C
Estimation of the pulmonary-to-systemic flow ratio to assist in surgical decision-making.^1060 – 1062^	IIa	C

#### 4.2.1.3. Partial and Total APVC

The anatomy of both partial and total APVC is well characterized by both CT and CMR. CMR provides detailed evaluation of venous confluence and the drainage site, identifying potential areas of venous obstruction. It also assess cardiac anatomy to identify associated lesions,^971 , 1067 – 1070^being particularly indicated for preoperative assessment in these patients.^974 , 976^

CMR has the advantage over CT of providing a better evaluation of hemodynamic effects, degree of chamber overload, and ventricular performance. It may be used for follow-up in asymptomatic partial APVC cases involving more than 1 vein or total APVC, especially when there is a change in clinical presentation.^974^Postoperatively, CMR may play a role in the follow-up of patients at higher risk of unfavorable outcomes, such as venous obstruction or dysfunction, or total APVC.^974^ Table 75 presents the main recommendations for the use of CMR in the evaluation of APVC.

**Table 75:** Recommendations for the use of cardiac magnetic resonance imaging in the assessment of anomalous pulmonary venous connection (APVC)

Indication	Class of recommendation	Level of evidence
Evaluation prior to correction of APVC.^976 , 1066 , 1071^	I	B
Post-treatment follow-up of APVC-related complications.^971 , 1068^	I	C
Postoperative evaluation of APVC repair in asymptomatic patients with no or mild sequelae.^1063^	IIa	C

#### 4.2.1.4. VSDs

VSDs are also well assessed by echocardiography, which typically allows for adequate clinical decision-making. CMR is reserved for cases where echocardiography cannot provide a complete evaluation, usually in adolescents and adults, particularly as part of the preoperative assessment.^974^Apical muscular VSDs may be more difficult to visualize on echocardiography. In DORV, both CMR and TC are valuable for accurately determining VSD position, dimension, and location, particularly in supracristal VSDs.^1005 , 1072 – 1074^This information is crucial for planning the adequate approach for VSD repair.

As with the other defects described above, CMR is able to quantify hemodynamic effects, including the Qp/Qs ratio, degree of chamber overload, and ventricular performance, as well as associated defects.^975^In the follow-up of untreated VSDs or in treated individuals, CMR is only relevant in patients with unfavorable clinical evolution.^974^ Table 76 presents the recommendations for the use of CMR in the evaluation of VSDs.

**Table 76:** Recommendations for the use of cardiac magnetic resonance imaging in the assessment of ventricular septal defects

Indication	Class of recommendation	Level of evidence
Evaluation prior to ventricular septal repair to evaluate for location, anatomy, and functional status.^976^	IIa	C
Evaluation following surgical or percutaneous repair in patients with significant residual shunt, valvular or ventricular dysfunction, arrhythmias, and/or pulmonary hypertension.^974 , 975^	IIa	C
Estimation of the pulmonary-to-systemic flow ratio to assist in surgical decision-making.^1060 , 1061^	IIa	C

#### 4.2.1.5. AVSDs

In most cases, echocardiography remains the primary modality for the assessment of AVSDs, as it provides comprehensive and conclusive assessments. CMR is typically reserved for cases where there is a need to investigate associated lesions or to more accurately quantify the hemodynamic effects of AVSDs,^975^as well as to assess the degree of ventricular imbalance.

CMR may play a role in preoperative evaluation or in the follow-up of patients (whether treated or not) with unfavorable clinical evolution. Routine post-treatment evaluation with CMR is indicated primarily for high-risk patients (eg, those with congestive symptoms, residual shunts, etc.).^974^ Table 77 presents the recommendations for the use of CMR in the evaluation of AVSDs.

**Table 77:** Recommendations for the use of cardiac magnetic resonance imaging in the assessment of atrioventricular septal defects (AVSD)

Indication	Class of recommendation	Level of evidence
Evaluation prior to AVSD repair.^976^	IIa	C
Follow-up of AVSD repair in patients with significant residual shunt, valvular or ventricular dysfunction, left ventricular outflow tract obstruction, arrhythmias, and/or pulmonary hypertension.^1063 , 1067^	IIa	C
Follow-up of AVSD repair in patients showing signs of heart failure.^974^	IIa	C

#### 4.2.1.6. Patent Ductus Arteriosus

PDA is also adequately assessed by echocardiography in most patients. CMR and CT are reserved for cases where echocardiography is inconclusive or ambiguous, particularly in pretreatment evaluations.^974^

In cases with larger or complex PDA morphologies, CMR and CT offer superior anatomical assessment, providing accurate measurements and critical information for treatment planning, especially for percutaneous interventions.

Some patients may develop obstructions in the pulmonary or aortic territories after treatment, which are well-evaluated by CMR.^974^As in other scenarios, CRM can help assess the repercussions of PDA,^975^particularly during follow-up of patients with unfavorable outcomes, whether treated or not.^974^ Table 78 presents the recommendations for the use of CMR in the evaluation of PDA.

**Table 78:** Recommendations for the use of cardiac magnetic resonance imaging in the assessment of patent ductus arteriosus (PDA)

Indication	Class of recommendation	Level of evidence
Evaluation prior to PDA correction.^974^	IIb	C
Post-treatment evaluation in patients with suspected aortic or pulmonary artery complications.^975^	I	C

#### 4.2.2. Congenital Valve Abnormalities

Congenital heart diseases may be associated with valvular abnormalities. Echocardiography is the modality of choice for assessing valve morphology and function. CMR is primarily used to evaluate the physiological impact of valvular regurgitation by measuring the regurgitant volume, regurgitant fraction, and ventricular size and function. This information plays a crucial role in deciding the timing of therapeutic interventions, particularly surgical and/or percutaneous procedures. In patients with valvular stenosis, CMR can be used to determine valve orifice size, although it may underestimate peak velocity and the estimated pressure gradient.

#### 4.2.2.1. Tricuspid Valve/Ebstein's Anomaly

CMR is considered the gold standard for quantifying RV volumes and function, which are often challenging to assess accurately with echocardiography. Serial evaluations of RV volume and function by CMR can be useful for the determination of disease progression and RV deterioration. Quantification of tricuspid regurgitation is particularly challenging in patients with Ebstein's anomaly, where the regurgitant jet(s) may be multiple and have unusual directions. Additionally, the direction of jet may change in different phases of systole due to annular motion of the tricuspid valve, potentially leading to inaccuracies and different findings between examinations.^988^

Neijenhuis et al.^1075^conducted a retrospective review of patients with Ebstein's anomaly from a single institution who underwent cone reconstruction. The primary objectives were to assess late-term tricuspid valve competence and biventricular function using CMR. The secondary objective was to evaluate biventricular reverse remodeling after cone reconstruction. The authors chose to not include the atrialized RV in the RV volume preoperatively, which significantly reduced measured RV volumes. If the authors had measured the "anatomical RV" (functional RV + atrialized RV) both preoperatively and postoperatively, an even more significant decrease in these volumes might have occurred after cone repair. This is why some authors suggest including the total "anatomical RV" in the RV volume preoperatively.^988^Other authors also suggest that the study by Neijenhuis et al.^1075^is limited by the small number of patients who had follow-up CMR imaging.^1076^Beroukhim et al.^1077^retrospectively reviewed pre and postoperative CMR studies of patients with Ebstein's anomaly and found significantly reduced RV volumes. Cone reconstruction led to reduced tricuspid regurgitation and RV stroke volume, increased LV stroke volume, improved LV basal septal strain, and improved LV synchrony. The authors included the atrialized RV in the RV volumes as they believed this would better demonstrate volume changes related to volume load reduction, given that the cone reconstruction repositions the functional annulus.

#### 4.2.2.2. Mitral Valve

Although several studies have demonstrated the value of CMR in the evaluation and quantification of mitral regurgitation, echocardiography remains the method of choice due to its accessibility, low cost, and convenience. In young children with congenital mitral valve regurgitation, echocardiography is used for diagnosis and follow-up, while CMR is reserved for cases where echocardiography is inconclusive or clinical findings do not correspond to the severity of mitral regurgitation. There is a dearth of comparative studies, and most have shown a modest agreement between qualitative and quantitative values. This is reasonable given the several factors affecting the evaluation of mitral valve regurgitation by each imaging modality. Quantification reproducibility has been consistently higher with CMR.^875^

Cawley et al.^1078^prospectively compared echocardiography and CMR in the evaluation of mitral valve regurgitation and showed that CMR has lower intraobserver and interobserver variabilities for regurgitant volume, suggesting CMR may be superior for serial measurements.

Guidelines from the European Society and AHA/ACC emphasize the importance of assessing the hemodynamic effects of mitral regurgitation on the LV and LA. However, these guidelines offer limited recommendations on how to conduct a comprehensive and standardized CMR evaluation.^1079^

#### 4.2.2.3. Mitral Stenosis

Mitral valve stenosis rarely occurs as an isolated defect. It more commonly occurs as an acquired lesion or in association with left-sided obstructive lesions, such as Shone's syndrome and hypoplastic left heart syndrome, or other CHDs.

Most of the complex congenital mitral valve lesions, including double-orifice MV, supravalvular mitral ring, mitral arcade, and parachute MV, are typically associated with MV stenosis. In children, estimation of MV stenosis includes at least the evaluation of transvalvular pressure gradient, orifice area, and annular diameter. Recommendations for adults cannot be directly applied to children without adequate modulation according to age. Evaluations should also include associated defects, as they may influence therapeutic approach. Typically, data obtained via echocardiography are satisfactory, but additional tools such as 3D echocardiography may sometimes be used as well. The role of an integrated multi-modality imaging approach, including CMR and CT, should be discussed and reserved for more complex cases where the analysis of associated defects causing left-sided obstructive lesions is essential for determining their severity.^1080^

#### 4.2.2.4. Pulmonary Valve

Flow measurement by CMR using velocity mapping is useful for estimating the severity of pulmonary regurgitation or obstruction due to congenital malformations or after percutaneous intervention.

CMR is currently the best method for quantification of pulmonary regurgitation and serial assessment of RV remodeling and function in patients with significant pulmonary regurgitation. The direct method based on the use of phase-contrast imaging with acquisition obtained from above the pulmonary valve is the preferred method, as it allows direct measurement of total pulmonic forward stroke volume and regurgitant fraction.^875^

Echocardiography and right heart catheterization are the gold standards for diagnosing pulmonary valve stenosis and assessing disease severity and treatment response.^1081^MRI may also provide valuable information about valve morphology and mobility, poststenotic dilatation, the degree of stenosis, and the location of supra or subvalvular stenosis. Different types of stenosis may be distinguished by CMR. The valvular pulmonary stenosis jet may be directed toward the left pulmonary artery, resulting in enlargement of the left pulmonary artery and increased blood flow to the left lung. Stenotic bicuspid pulmonary valve is commonly associated with poststenotic pulmonary arterial dilatation and aneurysm formation resulting from separated poststenotic flow, which may be seen at 4D phase-contrast MRI.^990^The degree of stenosis may also be evaluated using velocity mapping, although echocardiography is typically preferred.

#### 4.2.2.5. Aortic Valve

Although CMR can provide important information about aortic valve morphology, its full potential has yet to be determined, and further studies of clinical outcomes are needed before CMR data can be integrated into the management of patients with significant aortic valvular lesions. Cine MRI of the aortic valve leaflets can be used to assess aortic valve morphology in the presence of poor echocardiographic windows. CMR can provide a detailed assessment of the aortic valve structure and aortic root anatomy, which assists in identifying the cause of regurgitation. In addition, the dimensions of the entire thoracic aorta can be measured. The advantages of CMR in aortic regurgitation are quantification of the regurgitation and of LV volumes and function, particularly for serial measurement.

Bicuspid aortic valve (BAV) is the most common malformation of the aortic valve and one of the most common congenital cardiovascular malformations, with an estimated incidence of 1% to 2% of the population. Aortic regurgitation and aortic stenosis are common in BAV. BAVs may occur alone or in association with other congenital malformations, such as CoA, sub or supravalvular aortic stenosis, VSD, PDA, and sinus of Valsalva aneurysm. Patients with BAVs who have documented dilation of the sinuses of Valsalva or ascending aorta should undergo serial assessment of the dimensions, as the aortopathy may progress with time. Table 79 presents the recommendations for the use of CMR in the evaluation of congenital valve abnormalities.

**Table 79:** Recommendations for the use of cardiac magnetic resonance imaging in the assessment of congenital valve abnormalities

Indication	Class of recommendation	Level of evidence
Complementary evaluation of regurgitant valvular lesions.^875 , 1079 , 1082^	I	B
Complementary evaluation of stenotic valvular lesions.^974 , 976^	IIa	B
Evaluation prior to correction of Ebstein's anomaly (anatomy of the tricuspid valve, right ventricle, and pulmonary tree).^988 , 1083^	IIa	C
Evaluation of valvular or ventricular dysfunction following correction of Ebstein's anomaly.^1075 , 1077^	IIa	C

#### 4.2.3. Conotruncal Anomalies

#### 4.2.3.1. Tetralogy of Fallot

The major contribution of MRI lies in the postoperative evaluation of residual defects, with pulmonary insufficiency being one of the most significant complications. The main advantage of MRI is its ability to accurately quantify ventricular size and function. It is the gold standard for evaluating ventricular volumes and flow in patients with tetralogy of Fallot, as it can assess the hemodynamic effects of residual pulmonary regurgitation on RV function after repair of tetralogy of Fallot. Untreated pulmonary regurgitation is detrimental in the long term, causing ventricular dilation and dysfunction, arrhythmias, and late adverse events.^1084^Tricuspid regurgitation may occur as a consequence of RV dilation, causing even more RV dilation. Both RV and LV dysfunction may be due to longstanding cyanosis before repair and/or inadequate myocardial protection during repair, adverse ventricular–ventricular interactions, electromechanical dyssynchrony, and coronary artery abnormalities.^1085 , 1086^

The timing of surgical reintervention is crucial and remains a challenge, requiring monitoring of ventricular volumes and function to avoid missing the chance of functional recovery after the intervention. Recommendations for reintervention in asymptomatic patients with significant pulmonary regurgitation depend on RV volumes. When there is progressive RV dilation to RV indexed end-systolic volume (RVESVi) ≥ 80 mL/m² and/or RV indexed end-diastolic volume (RVEDVi) ≥ 160 mL/m², reintervention should be considered, especially when there is associated dysfunction of either ventricle.^1087 – 1090^

The postoperative follow-up of tetralogy of Fallot repair should also include evaluation of aneurysms and akinetic regions in the RVOT and other residual lesions such as stenoses or intracardiac shunts. Residual VSD after surgical repair usually results from patch dehiscence or incomplete closure and may lead to LV volume overload. Residual stenosis may be present in the infundibulum, pulmonary valve, pulmonary trunk, or pulmonary arteries. RV hypertrophy and pressure overload have been described as independent risk factors for poor RV performance and unfavorable evolution, even in patients with smaller RV volumes.^1091^

Complications such as aortic dilation and aortic valve regurgitation may also occur in the follow-up of these patients, but they rarely progress to AD.^1092^

Additionally, LGE can be used to establish prognosis by correlating the amount of fibrosis with ventricular dysfunction, exercise intolerance, and arrhythmia onset.

More recently, the use of parametric mapping has been growing, and increased ECV measured by pre- and post-LGE T1 values has also been associated with RV volume overload and arrhythmias, suggesting that the measurement of diffuse fibrosis can help stratify patient risk. Table 80 presents the recommendations for the use of CMR in the evaluation of tetralogy of Fallot.

**Table 80:** Recommendations for the use of cardiac magnetic resonance imaging in the assessment of tetralogy of Fallot

Indication	Class of recommendation	Level of evidence
Evaluation prior to repair in patients with unfavorable anatomy or when echocardiography is limited.	I	C
Postoperative evaluation prior to planned pulmonary valve replacement (percutaneous or surgical).^1088 , 1093 – 1097^	I	B
Evaluation in patients with right ventricular outflow tract obstruction, pulmonary stenosis, arrhythmias, or the presence of a right ventricle-to-pulmonary artery conduit.^1091^	I	C

#### 4.2.3.2. DORV

DORV involves a wide spectrum of anatomical arrangements depending on the relationship of the VSD, resulting in variable physiological presentations.^1005^Therefore, surgical intervention depends on the prevailing physiology preoperatively.

MRI provides 3D assessment of intracardiac anatomy, characterizing the vascular relationship with the VSD, as well as a comprehensive assessment of valvular and ventricular function and volumes. Evaluation of the atrioventricular valve apparatus, its relationship with the VSD, and the presence or absence of straddling are important diagnostic information. Biventricular repair may only be performed in the presence of 2 adequately sized ventricles.^1008^The creation of an intraventricular tunnel compromises RV volume because a part of the RV is incorporated into the LVOT. Therefore, it is important to assess preoperative RV cavity size and estimate the volume of the remaining RV after the intended surgical approach.

Preoperative evaluation of these patients will help determine whether the anatomical substrate is suitable for biventricular repair. The presence of a subaortic or subpulmonary infundibulum should be assessed, as it assists in preoperative decision-making.

Potential complications after biventricular repair depend on the surgery indicated for each DORV phenotype. These may include ventricular dilation and/or dysfunction, ventricular outflow tract obstruction, and residual VSD.^1098^ Table 81 presents the recommendations for the use of CMR in the evaluation of DORV.

**Table 81:** Recommendations for the use of cardiac magnetic resonance imaging in the assessment of double-outlet right ventricle

Indication	Class of recommendation	Level of evidence
Evaluation of ventricular septal defect relationship and vascular anatomy prior to repair.^1005 , 1008^	I	C
Evaluation of ventricular function and volumes.^1067^	I	C
Evaluation of right/left ventricular outflow tract obstruction, pulmonary stenosis, arrhythmias, or the presence of a right ventricle-to-pulmonary artery conduit.^1098^	IIa	C

#### 4.2.3.3. Common Arterial Trunk

Preoperative evaluation in patients with common arterial trunk is typically performed with echocardiography. In some cases, the origin of the pulmonary arteries from the common arterial trunk may not be clear, or a better assessment of the truncal valve may be necessary.^1099^The root of the ventriculoarterial junction is often dilated, and the truncal valve almost always exhibits some abnormality, such as dysplasia or an abnormal number of cusps with poor coaptation, often leading to insufficiency. In rare cases, there is limited valve opening.^1100^

Evaluation of the aortic arch should also be conducted to establish its laterality and integrity. Common arterial trunk may sometimes be associated with aortic arch interruption, most commonly between the left common carotid artery and subclavian arteries. In this case, the length of the interrupted segment, the diameter of the aorta distal to the interrupted segment, and areas of stenosis should be quantified.^1101^

The surgical repair of patients with common arterial trunk involves reconstructing the RVOT to restore continuity between the RV and pulmonary artery. This may include the placement of an RV-to-pulmonary artery (RV-PA) conduit, which is prone to develop stenosis and regurgitation. MRI provides accurate anatomic and functional assessment of residual lesions in the RV-PA conduit and the distal pulmonary arterial tree, as well quantification of RV volumes and mass to assess for RV sequelae.^1102^RV assessment is crucial to assess for RV insufficiency or residual RVOT obstruction after surgical repair, and monitoring should be performed to assist in decision-making regarding additional therapeutic procedures.

Repercussions of truncal valve dysfunction on the LV may occur, the most common being valve regurgitation. Patients with a repaired aortic arch may exhibit some degree of obstruction postoperatively, which is another cause of long-term pressure overload. Table 82 presents the recommendations for the use of CMR in the evaluation of common arterial trunk.

**Table 82:** Recommendations for the use of cardiac magnetic resonance imaging in the assessment of common arterial trunk

Indication	Class of recommendation	Level of evidence
Evaluation of anatomy prior to repair.^1099 , 1100^	IIa	C
Postoperative evaluation of known residual ventricular septal defect, right ventricle-to-pulmonary artery conduit stenosis, or pulmonary stenosis.^1103^	IIa	C
Postoperative evaluation of truncal valve stenosis or regurgitation.	IIa	C

#### 4.2.3.4. Transposition of the Great Arteries

Echocardiography is the imaging modality of choice for preoperative evaluation of patients with transposition of the great arteries and, in the vast majority of cases, can provide sufficient anatomical and functional information for surgical correction. In patients with more complex morphology, preoperative assessment may be complemented by CT for coronary delineation or by either CMR or CT for suspected subpulmonary obstruction or aortic delineation.

The Jatene procedure (arterial switch operation) is currently the surgical procedure of choice. The main postoperative complications are related to the anastomotic and coronary reimplantation sites.^1019^The pulmonary valve and aortic valve may be assessed by local flow measurements or MRA.^1104^

Pulmonary artery or supravalvular pulmonary artery stenosis may occur after the LeCompte maneuver, either unilaterally or bilaterally, due to vascular displacement. Neoaortic root dilation and neoaortic valve regurgitation are common after surgery, leading to long-term complications. Aortic regurgitation may be quantitatively assessed by CMR by estimating the local regurgitant fraction and its impact on the left chambers.

Complications such as ischemia and infarction can be evaluated by myocardial perfusion and myocardial viability assessment. LV dysfunction and arrhythmias are rare but can occur and may be related to problems with coronary reimplantation or myocardial protection during the arterial switch procedure.^1105^Evaluation of the vascular anatomy of coronary reimplantation is more accurately assessed by CT due to its better spatial resolution.

After atrial-level repair for transposition of the great arteries, in which only physiological correction is achieved, the patency of the venous baffle, as well as systemic RV contractile performance and the presence of tricuspid valve regurgitation, should be evaluated. In the long-term, there may be RV dysfunction and failure as well as progressive tricuspid regurgitation (systemic atrioventricular valve). MRI provides reliable and robust data on systemic RV systolic function. Additionally, it can better assess the degree of myocardial hypertrophy and associated fibrosis. It can also quantify residual shunting at the atrial level, although small communications may be better visualized by echocardiography. Table 83 presents the recommendations for the use of CMR in the evaluation of transposition of the great arteries.

**Table 83:** Recommendations for the use of cardiac magnetic resonance imaging in the assessment of transposition of the great arteries

Indication	Class of recommendation	Level of evidence
Evaluation of anatomy prior to repair.^1018^	IIa	C
Coronary evaluation following a Jatene procedure.^1019 , 1021^	IIb	C
Evaluation of moderate valvular or ventricular dysfunction, right or left ventricular outflow tract obstruction, pulmonary stenosis, or arrhythmias following a Jatene procedure.^1104^	I	C
Evaluation of dilated neoaortic root or neoaortic regurgitation following a Jatene procedure.^1022^	I	C
Evaluation of systemic AV regurgitation, systemic LV dysfunction, RVOT obstruction, or arrhythmias following atrial correction (eg, Senning procedure).	I	C
Evaluation of venous baffles following atrial correction (eg, Senning procedure).^1106^	I	C

#### 4.2.3.5. Congenitally Corrected Transposition of the Great Arteries

Congenitally corrected transposition of the great arteries may occur alone or in combination with other structural cardiac anomalies. MRI provides assessment of associated lesions, which may include pulmonary stenosis, tricuspid valve anomalies, and VSD. It can also characterize intracardiac and large vessel anatomy and is indicated for the quantification of ventricular volumes, function, and myocardial mass, especially in cases where echocardiography of the RV is more challenging and less accurate. The tricuspid valve may be dysplastic and exhibit variable degrees of coaptation (Ebstein-like) or regurgitation due to systemic RV dilation.

Functional assessment of the anatomical RV, which is connected to the aorta and is under systemic load, should be conducted, as it will eventually develop systolic or tricuspid regurgitation.^1107^

Physiological correction of structural lesions may be performed, but the RV will remain supporting the systemic circulation. These include VSD closure, tricuspid valve repair, and pulmonary stenosis correction. In addition to investigating residual lesions such as intracardiac shunts and quantifying tricuspid regurgitation and residual subpulmonary stenosis, systemic RV volumes, mass, and function should also be accurately monitored. MRI is the gold standard for evaluating the systemic RV and is recommended for adequate patient management.^1108 , 1109^Changes in RV geometry such as significant dilation and hypertrophy, as well as the investigation of myocardial fibrosis, where LGE is typically seen at the inferior insertion point of the interventricular septum with no known clinical significance, and the presence of dense focal fibrosis in other portions of the RV remain controversial.^1110^Larger studies investigating interstitial fibrosis using parametric mapping are necessary.

A double-switch operation may be performed in some patients with congenitally corrected transposition of the great arteries to reroute venous returns and ventricular outflows to convert the morphological LV into a systemic ventricle and the RV into a subpulmonary ventricle.^1025 , 1111^In this setting, CT is often the best method for evaluating coronary reimplantation. Table 84 presents the recommendations for the use of CMR in the evaluation of congenitally corrected transposition of the great arteries

**Table 84:** Recommendations for the use of cardiac magnetic resonance imaging in the assessment of congenitally corrected transposition of the great arteries

Indication	Class of recommendation	Level of evidence
Evaluation of anatomy prior to repair.^1112^	IIa	C
Postoperative evaluation in patients with systemic AV valve regurgitation or systemic RV dysfunction.^1107 – 1109^	I	C
Postoperative evaluation in patients with left ventricle-to-pulmonary artery dysfunction.^1111^	IIa	C

#### 4.2.4. Anomalies of the Thoracic Aorta

#### 4.2.4.1. CoA

In young children with adequate acoustic windows, echocardiography is often sufficient to provide accurate and comprehensive preoperative information. CMR and CT are reserved for cases where echocardiography is inconclusive (older patients with poor acoustic windows) or when there is a need for a panoramic view of the aorta, as in cases of suspected associated lesions in the aortic arch.^2 – 4^Although CMR is not routinely performed, it is superior to echocardiography in several aspects. CMR allows for a more comprehensive and accurate visualization of CoA anatomy, location, and severity, as well as of aortic arch anatomy and spatial relationship with its vessels. It also provides more accurate assessment of regional structures and the presence and extent of collaterals, allowing for adequate surgical planning even in complex cases.^419 , 1040 , 1113^Follow-up MRI or CT may be performed to assess for long-term complications such as aneurysm formation, fracture, or stent migration. Table 85 presents the main recommendations for the use of CMR in the evaluation of CoA.

**Table 85:** Recommendations for the use of magnetic resonance imaging in the assessment of coarctation of the aorta (CoA)

Indication	Class of recommendation	Level of evidence
Evaluation prior to planned repair.^1114 , 1115^	I	B
Post-treatment evaluation in patients with change in clinical status.^1116 , 1117^	I	C
Follow-up within a year (6-12 months) after percutaneous repair in asymptomatic patients with no or mild sequelae.^419 , 1040 , 1113 , 1118^	IIa	B
Follow-up (1-2 years) after the first year following repair in asymptomatic patients with no or mild sequelae.^419 , 1040 , 1113 , 1118^	IIa	B
Follow-up in asymptomatic patients to assess for aortic arch aneurysm and/or stent (restenosis, fracture, or endoleak).^419 , 1040 , 1048 , 1113 , 1118^	IIa	B
Follow-up in patients with heart failure symptoms.^419 , 1040 , 1113 , 1118^	IIa	B

#### 4.2.4.2. Other Aortic Anomalies

The aorta can be affected by other congenital diseases, some of which are complex and require adequate preoperative evaluation. Some of these include aortic arch hypoplasia, interrupted aortic arch, and aortopulmonary window.^1119^

CMR is a well-established method for evaluating the aorta and its main branches in selected complex cases, allowing for an appropriate assessment of these conditions. CT provides a comparable evaluation and is superior in the evaluation of vascular rings, offering a better assessment of air-filled structures such as the trachea.

These methods are especially useful in postoperative follow-up. The clinical settings and recommendations are similar to those of CoA.^1119^ Table 86 presents the main recommendations for the use of CMR in the evaluation of aortic arch anomalies.

**Table 86:** Recommendations for the use of computed tomography in the assessment of aortic arch anomalies

Indication	Class of recommendation	Level of evidence
Evaluation of vascular rings.^1118 , 1119^	IIa	C
Evaluation of aortic arch interruption.^1119^	I	C
Evaluation of aortopulmonary window.^1119^	I	C

#### 4.2.5. Univentricular Heart

Univentricular heart refers to a wide spectrum of cardiac malformations that preclude biventricular surgical repair. Correction of univentricular heart involves 3 stages of univentricular palliative surgery.^1020^

Echocardiography remains the primary modality for initial evaluation. In patients with a borderline ventricle, CMR may be used to help decide between uni or biventricular repair. Grosse-Wortmann et al.^1118^compared the usefulness of echocardiography and CMR in the evaluation of borderline LV. Echocardiography consistently underestimated LV volume and did not correlate with CMR. LV geometry in patients with borderline LV is almost certainly the reason for the inaccuracy of echocardiography when compared with CMR.^419 , 1113 , 1118^

Although echocardiography remains a mainstay of diagnosis for patients with single-ventricle physiology, CMR has additional utility, especially in the evaluation of extracardiac anatomy by multiplanar and 3D reconstructions in patients with suboptimal acoustic windows or other technical limitations. CMR has become the gold standard for evaluating ventricular volumes, EF, and mass, using contiguous and parallel imaging with high temporal resolution, without the need for assumptions about ventricular shape. This is particularly true in the context of complex CHDs, in which the ventricular shape is typically unusual and highly variable, making echocardiographic assessment of volumes and mass less accurate and reproducible than CMR.^1120 – 1122^Ventricular function and geometry are crucial parameters in the clinical evolution of patients with Fontan circulation.^5 , 6^CMR allows for functional evaluation of the AV valve of the systemic ventricle and provides analysis of flow patterns through the Fontan circuit.^1048^

Studies have demonstrated that CMR combined with echocardiography may replace routine diagnostic catheterization in selected patients before the bidirectional Glenn procedure. In the absence of evidence of PH, routine measurement of pulmonary vascular resistance is not necessary before bidirectional Glenn operation. Brown et al.^1123^compared catheterization and CMR in patients considered for bidirectional Glenn operation and found pulmonary resistance values within acceptable limits in the catheterization group, while patients in the CMR group did not fare differently after bidirectional Glenn operation as a result of the absence of resistance and pressure data.^1114 , 1117 , 1123^

After the Fontan procedure, patients remain at risk for numerous complications, including ventricular and valvular dysfunction, obstruction and/or stenosis of the Fontan conduit, pulmonary artery stenosis, CoA, formation of venous and systemic pulmonary collaterals, and intracardiac thrombus formation. CMR plays a fundamental role in monitoring these complications, as the limited acoustic window of echocardiography often precludes their detection.^419^

CMR also plays a role in determining the prognosis of these patients, as parameters derived from CMR, such as ventricular volume and myocardial fibrosis, were shown to be associated with adverse outcomes.^1115 , 1124^

CMR offers the possibility of tissue characterization with T1, T2, and T2* mapping of the myocardium to assess evidence of myocardial edema, scars, diffuse fibrosis, and iron deposition.^1125^Many of these new quantitative techniques have only been used in small pediatric populations and are still under investigation to provide evidence-based data proving their utility.^1048^

Abnormal vascular connections between systemic arteries and the pulmonary vascular bed, known as systemic-to-pulmonary collateral (SPC) vessels, manifest in patients with a variety of congenital and acquired heart diseases, commonly occurring in patients with single-ventricle anatomy after superior cavopulmonary connection. However, their clinical significance remains unknown. It is well established that SPC flow can be quantified after bidirectional cavopulmonary connection and Fontan surgery using MRI flow analysis techniques.^1053 , 1126 – 1130^

After Glenn or Fontan operations, the increased central venous pressure may induce recanalization of embryologically preformed and obliterated vessels, with the development of systemic-to-pulmonary venous collaterals that can lead to systemic desaturation and worsening of ventricular function. This results in impaired daily performance in patients with univentricular heart.^1131 – 1134^

Lymphatic complications in patients with single-ventricle physiology may lead to Fontan failure and severe Fontan complications, such as plastic bronchitis, protein-losing enteropathy, and chylothorax. These complications are a significant source of morbidity and mortality, with historically limited treatment options. New lymphatic imaging techniques, such as non-contrast T2-weighted MR lymphography and dynamic contrast enhanced MR lymphangiography, can assess the anatomy of the lymphatic system in this patient population. In addition, these techniques have been instrumental in guiding the development of new lymphatic intervention methods, thereby advancing the treatment of lymphatic diseases.^1134 – 1142^ Table 87 presents the main recommendations for the use of CMR in the management of univentricular heart.

**Table 87:** Recommendations for the use of computed tomography in the assessment of univentricular heart

Indication	Class of recommendation	Level of evidence
Evaluation of anatomy prior to repair.^974^	IIa	C
Evaluation after stage 1 surgery (systemic-to-pulmonary artery shunt or pulmonary banding).^974 , 976^	I	C
Evaluation of valvular or ventricular dysfunction, superior vena cava to pulmonary artery anastomosis, and collateral circulation after stage 2 surgery (Glenn procedure).^419 , 1116^	I	C
Evaluation of valvular or ventricular dysfunction, total cavopulmonary connection thrombosis, or collateral circulation after stage 3 surgery (Fontan procedure).^1048 , 1117^	I	C

#### 4.2.6. Other

MRI may be useful in the evaluation of more complex heart diseases associated with significant spatial and geometric changes when echocardiography is limited. As MRI is not limited by poor acoustic windows, it can provide a more in-depth assessment, making it possible to detect not only intracardiac lesions but also associated thoracic and abdominal viscera abnormalities.^1143^

Heterotaxy is a group of congenital syndromes characterized by abnormal arrangement of thoracic and abdominal viscera. It can be classified as right or left isomerism depending on the morphology of atrial appendages. Heterotaxy involves a wide spectrum of anomalies, such as midline liver, polysplenia or asplenia, interruption of the inferior vena cava, and anomalous pulmonary venous drainage, as well as associated intracardiac malformations.^1144 , 1145^

CMR may also assist in the diagnosis of spatial anomalies such as crisscross heart, characterized by superoinferior ventricles and crossing of the ventricular inflow streams,^1146 , 1147^in which it can visualize not only changes in perpendicular inflow tract disposition but also valve opening dynamics and the presence of straddling or overriding.^1148^

Table 88 presents the recommendations for the use of CMR in the evaluation of other cardiac malformations.

**Table 88:** Recommendations for the use of cardiac magnetic resonance imaging in the evaluation of other cardiac malformations

Indication	Class of recommendation	Level of evidence
Evaluation of thoracic organ arrangement and long axis rotation.^1149^	I	C
Evaluation of situs anomalies and heterotaxy.^1055 , 1145^	I	C

## List of abbreviations

AA: – aortic aneurysm
AAS: – acute aortic syndrome
ABI: – ankle-brachial index
ACC/AHA: – American College of Cardiology/American Heart Association
ACCURACY trial: – Assessment by Coronary Computed Tomographic Angiography of Individuals Undergoing Invasive Coronary Angiography
ACRIN-PA trial: – Angiography for Safe Discharge of Patients with Possible Acute Coronary Syndromes
ACS: – acute coronary syndrome
AD: – aortic dissection
AF: – atrial fibrillation
AMI: – acute myocardial infarction
APVC: – anomalous pulmonary venous connection
ARVD/C: – arrhythmogenic right ventricular dysplasia/cardiomyopathy
ASCVD: – atherosclerotic cardiovascular disease
ASD: – atrial septal defect
ATTR: – transthyretin amyloidosis
AUC: – area under the curve
AVSD: – atrioventricular septal defect
BEACON trial: – Better Evaluation of Acute Chest Pain with Coronary Computed Tomography Angiography
BMD: – Becker muscular dystrophy
CA: – cardiac amyloidosis
CAA: – coronary artery anomaly
CAC: – coronary artery calcium
CAC-DRS: – coronary artery calcium data and reporting system
CAD: – coronary artery disease
CAPTURE study: – Randomised Placebo-Controlled Trial of Abciximab Before and During Coronary Intervention in Refractory Unstable Angina
CARDIA study: – Coronary Artery Risk Development in Young Adults
CARMENTA trial: – CARdiovascular Magnetic rEsoNance imaging and computed Tomography Angiography
CATSCAN study: – Coronary Assessment by Computed Tomographic Scanning and Catheter Angiography
CBR: – Brazilian College of Radiology
CCTA: – coronary computed tomography angiography
CE-MARC study: – Clinical Evaluation of MAgnetic Resonance imaging in Coronary heart disease
CHD: – Chagas heart disease
CMR: – cardiac magnetic resonance
CoA: – coarctation of the aorta
CONFIRM registry: – Coronary CT Angiography Evaluation for Clinical Outcomes: An International Multicenter Registry
CONSERVE trial: – Coronary Computed Tomographic Angiography for Selective Cardiac Catheterization
CORE320 study: – Coronary Artery Evaluation using 320-row Multidetector Computed Tomography Angiography and Myocardial Perfusion
CORE64 study: – Coronary Artery Evaluation using 64-row Multidetector Computed Tomography Angiography
CP: – constrictive pericarditis
CRP: – C-reactive protein
CS: – cardiac sarcoidosis
CT: – computed tomography
CT-MPI: – CT myocardial perfusion imaging
CT-STAT trial: – Coronary Computed Tomographic Angiography for Systematic Triage of Acute Chest Pain Patients to Treatment
DCM: – dilated cardiomyopathy
DHS: – Dallas Heart Study
DISCHARGE trial: – Diagnostic Imaging Strategies for Patients with Stable Chest Pain and Intermediate Risk of Coronary Artery Disease
DISCOVER-FLOW trial: – Diagnosis of Ischemia-Causing Stenoses Obtained Via Noninvasive Fractional Flow Reserve
DM: – diabetes mellitus
DMD: – Duchenne muscular dystrophy
DORV: – double-outlet right ventricle
EBCT: – electron beam computed tomography
ECG: – electrocardiogram
ECV: – extracellular volume
EDWT: – end-diastolic wall thickness
EMB: – endomyocardial biopsy
EMF: – endomyocardial fibrosis
FASTTRACK CABG study: – Safety and Feasibility Evaluation of Planning and Execution of Surgical Revascularization Solely Based on Coronary CTA and FFR_CT_in Patients With Complex Coronary Artery Disease
FFR: – fractional flow reserve
FFR_CT_: – CT-derived fractional flow reserve
FH: – familial hypercholesterolemia
FHS: – Framingham Heart Study
FMD: – flow-mediated dilation
FOURIER trial: – Further Cardiac Outcomes Research With PCSK9 Inhibition in Subjects with Elevated Risk
FRS: – Framingham risk score
GBCA: – gadolinium-based contrast agent
HCM: – hypertrophic cardiomyopathy
HF: – heart failure
HNR study: – Heinz Nixdorf Recall
HR: – hazard ratio
hs-Tn: – high-sensitivity troponin
HU: – Hounsfield units
ICD: – implantable cardioverter-defibrillator
IMH: – intramural hematoma
IMT: – intima-media thickness
ISCHEMIA trial: – International Study of Comparative Health Effectiveness with Medical and Invasive Approaches
JUPITER trial: – Justification for the Use of Statins in Prevention: an Intervention Trial Evaluating Rosuvastatin
LA: – left atrium
LAA: – left atrial appendage
LDL-c: – low-density lipoprotein cholesterol
LGE: – late gadolinium enhancement
LV: – left ventricle
LVEF: – left ventricular ejection fraction
LVH: – left ventricular hypertrophy
LVNC: – left ventricular noncompaction cardiomyopathy
LVOT: – left ventricular outflow tract
MACE: – major adverse cardiovascular events
MDCT: – multidetector computed tomography
MESA: – Multi-Ethnic Study of Atherosclerosis
MINOCA: – myocardial infarction with nonobstructive coronary arteries
MRI: – magnetic resonance imaging
MR-INFORM trial: – Magnetic Resonance Imaging for Myocardial Perfusion Assessment in Coronary Artery Disease
NHLBI: – National Heart, Lung, and Blood Institute
NIMISCAD: – Non Invasive Multicenter Italian Study for Coronary Artery Disease
NNT: – number needed to treat
NRI: – net reclassification index
OR: – odds ratio
ORBITA trial: – Objective Randomised Blinded Investigation with optimal medical Therapy of Angioplasty in stable angina
PACC project: – Prospective Army Coronary Calcium
PAOD: – peripheral arterial occlusive disease
PDA: – patent ductus arteriosus
PE: – pulmonary embolism
PET: – positron emission tomography
PFO: – patent foramen ovale
PH: – pulmonary hypertension
PLATFORM trial: – Prospective LongitudinAl Trial of FFRct: Outcome and Resource IMpacts
PPCM: – peripartum cardiomyopathy
PREDICT study: – Prospective Evaluation of Diabetic Ischemic Disease by Computed Tomography
PROMISE: – Prospective Multicenter Imaging Study for Evaluation of Chest Pain
PROTECTION study: - Prospective Multicenter Registry on RadiaTion Dose Estimates of Cardiac CT AngIOgraphy IN Daily Practice
PTE: – pulmonary thromboembolism
Qp/Qs: – pulmonary-to-systemic flow ratio
RAPID-CTCA trial: – Rapid Assessment of Potential Ischaemic Heart Disease with Computerised Tomography Coronary Angiography
RF: – radiofrequency
ROBINSCA trial: – Risk or Benefit in Screening for Cardiovascular Disease
ROC: – receiver operating characteristic
ROMICAT II trial: – Rule Out Myocardial Ischemia/Infarction Using Computer Assisted Tomography
RA: – right atrium
RV: – right ventricle
RVEF: – right ventricular ejection fraction
RVOT: – right ventricular outflow tract
SARS-CoV-2: – severe acute respiratory syndrome coronavirus 2
SBC: – Brazilian Society of Cardiology
SCCT: – Society of Cardiovascular Computed Tomography
SCORE: – Systemic COronary Risk Evaluation
SCOT-HEART trial: – Scottish COmputed Tomography of the HEART
SIS: – segment involvement score
SPC: – systemic-to-pulmonary collateral
SPECT: – single-photon emission computed tomography
SPINS study: – Stress CMR Perfusion Imaging in the United States
SSFP: – steady-state free precession
SYNTAX III REVOLUTION trial: – A Randomized Study to Evaluate the Feasibility of Heart-Team Clinical Decision Making Regarding the Optimal (Surgical or Percutaneous Based) Revascularization Strategy in Patients With Complex Coronary Artery Disease, Based on Non-invasive Coronary CT Angiography (CTA) Imaging Utilising High-definition GE Revolution™ Multi-slice CT and HeartFlow FFR_CT_Compared to the Current Standard of Care With Conventional Invasive Coronary Angiography (CA)
TARGET-CTCA trial: – Troponin in Acute Chest Pain to Risk Stratify and Guide EffecTive Use of Computed Tomography Coronary Angiography
TAVI: – transcatheter aortic valve implantation
TEE: – transesophageal echocardiography
TP-NOCA: - Troponin positive with nonobstructive coronary arteries
TTE: – transthoracic echocardiography
ViV: – valve-in-valve
VSD: – ventricular septal defect
